# α-Synuclein oligomers potentiate neuroinflammatory NF-κB activity and induce Ca_v_3.2 calcium signaling in astrocytes

**DOI:** 10.1186/s40035-024-00401-4

**Published:** 2024-02-21

**Authors:** Emmanouela Leandrou, Ioanna Chalatsa, Dimitrios Anagnostou, Christina Machalia, Maria Semitekolou, Vicky Filippa, Manousos Makridakis, Antonia Vlahou, Ema Anastasiadou, Kostas Vekrellis, Evangelia Emmanouilidou

**Affiliations:** 1 Department of Chemistry, School of Sciences, National and Kapodistrian University of Athens, Panepistimioupolis Zografou, 15772 Athens, Greece; 2 Center for Basic Research, Biomedical Research Foundation of the Academy of Athens, Soranou Efesiou 4, 11527 Athens, Greece; 3 Center for Systems Biology, Biomedical Research Foundation of the Academy of Athens, Soranou Efesiou 4, 11527 Athens, Greece; 4 Present Address: School of Medicine, University of Crete, 71003 Heraklion, Greece

**Keywords:** α-Synuclein, Oligomers, Neuroinflammation, p38^MAPK^ signaling, Astrocytes, Ca_v_3.2 calcium channel, NF-κB, IGFBPL1

## Abstract

**Background:**

It is now realized that Parkinson’s disease (PD) pathology extends beyond the substantia nigra, affecting both central and peripheral nervous systems, and exhibits a variety of non-motor symptoms often preceding motor features. Neuroinflammation induced by activated microglia and astrocytes is thought to underlie these manifestations. α-Synuclein aggregation has been linked with sustained neuroinflammation in PD, aggravating neuronal degeneration; however, there is still a lack of critical information about the structural identity of the α-synuclein conformers that activate microglia and/or astrocytes and the molecular pathways involved.

**Methods:**

To investigate the role of α-synuclein conformers in the development and maintenance of neuroinflammation, we used primary quiescent microglia and astrocytes, post-mortem brain tissues from PD patients and A53T α-synuclein transgenic mice that recapitulate key features of PD-related inflammatory responses in the absence of cell death, i.e., increased levels of pro-inflammatory cytokines and complement proteins. Biochemical and -omics techniques including RNAseq and secretomic analyses, combined with 3D reconstruction of individual astrocytes and live calcium imaging, were used to uncover the molecular mechanisms underlying glial responses in the presence of α-synuclein oligomers in vivo and in vitro.

**Results:**

We found that the presence of SDS-resistant hyper-phosphorylated α-synuclein oligomers, but not monomers, was correlated with sustained inflammatory responses, such as elevated levels of endogenous antibodies and cytokines and microglial activation. Similar oligomeric α-synuclein species were found in post-mortem human brain samples of PD patients but not control individuals. Detailed analysis revealed a decrease in Iba1^Low^/CD68^Low^ microglia and robust alterations in astrocyte number and morphology including process retraction. Our data indicated an activation of the p38/ATF2 signaling pathway mostly in microglia and a sustained induction of the NF-κB pathway in astrocytes of A53T mice. The sustained NF-κB activity triggered the upregulation of astrocytic T-type Ca_v_3.2 Ca^2+^ channels, altering the astrocytic secretome and promoting the secretion of IGFBPL1, an IGF-1 binding protein with anti-inflammatory and neuroprotective potential.

**Conclusions:**

Our work supports a causative link between the neuron-produced α-synuclein oligomers and sustained neuroinflammation in vivo and maps the signaling pathways that are stimulated in microglia and astrocytes. It also highlights the recruitment of astrocytic Ca_v_3.2 channels as a potential neuroprotective mediator against the α-synuclein-induced neuroinflammation.

**Graphical Abstract:**

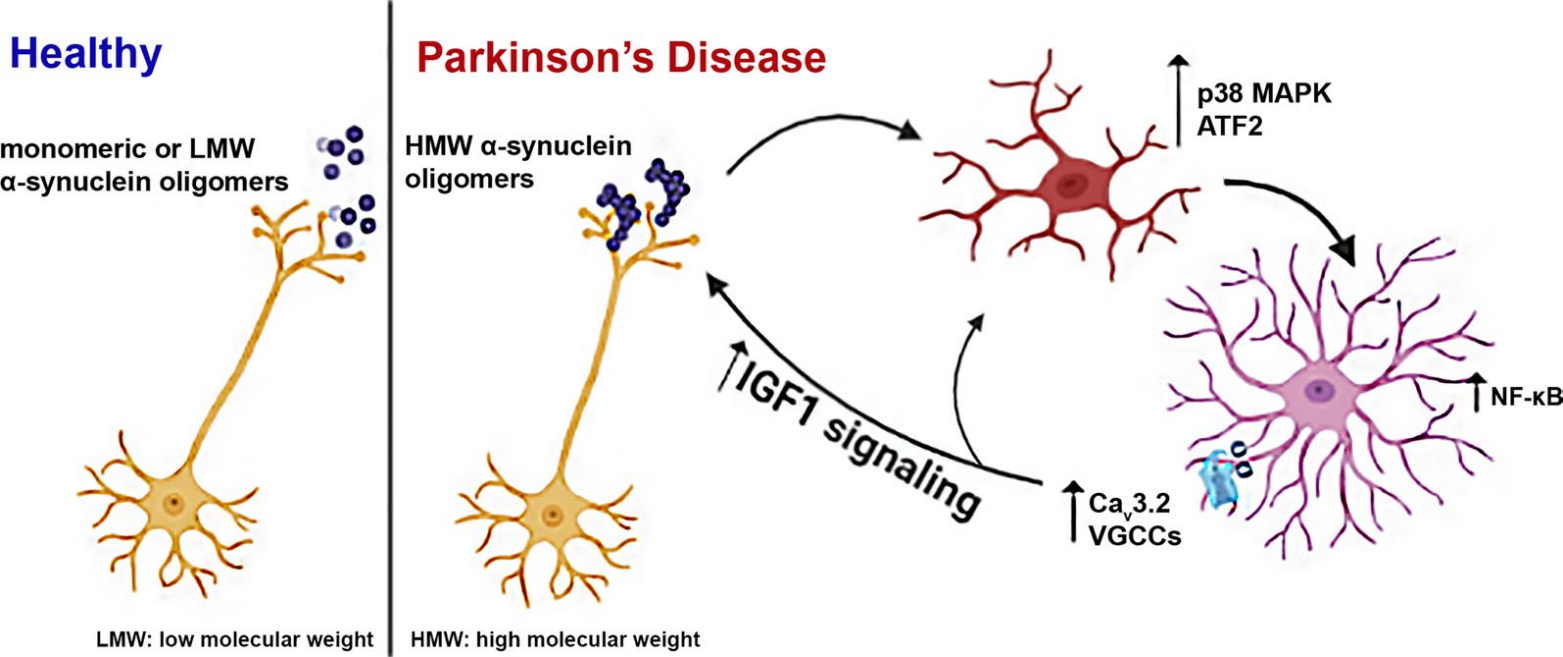

**Supplementary Information:**

The online version contains supplementary material available at 10.1186/s40035-024-00401-4.

## Introduction

Parkinson’s disease (PD) is a multifactorial movement disorder characterized by progressive neurodegeneration in certain brain areas and deposits of aggregated protein materials at the soma and axons of neurons, termed Lewy bodies (LBs) and Lewy neurites [1]. Despite the pivotal role of neuronal cell death in the central nervous system (CNS), PD is now recognized as a multi-system disorder characterized by notable neuroinflammation and immune dysfunction underlying many of the non-motor PD deficits such as sleep and gastrointestinal abnormalities, which in most cases, precede the onset of motor symptoms [2]. In this context, neuroinflammation and glial cell activation are not considered a response to cell death but rather a significant contributor to the pathogenic microenvironment [2, 3]. Post-mortem analysis of brain tissues of PD patients has shown widespread inflammatory manifestations as indicated by the sustained activation of CNS resident immune cells (microglia and astrocytes) and the infiltration of peripherally derived immune cells such as mononuclear phagocytes, neutrophils and lymphocytes. These features possibly initiate from intestinal dysbiosis and gut inflammation that lead to an increase in the circulating pro-inflammatory cytokines [2]. Both the innate and the adaptive peripheral systems are reported to be activated by cytokines and contribute to the generation and maintenance of immune responses in the PD brain. As a result of the extensive gliosis, the levels of pro-inflammatory cytokines are found to be elevated in PD brain and cerebrospinal fluid samples as well as in animal models of PD [4, 5].

At the cellular level, under pathological conditions in PD, damage-associated molecular patterns (DAMPs) are released from neurons and activate microglia and astrocytes through distinct molecular pathways including toll-like receptor (TLR)-mediated pathways and endocytosis. Activation of microglia dysregulates their phagocytic activity and induces activation of the inflammasome and complement proteins, secretion of pro-inflammatory cytokines and ROS production. The microglia-released cytokines induce a neurotoxic phenotype in astrocytes, which stop providing neuronal support, secrete neurotoxins and further amplify neuroinflammation. However, the exact trigger of glial reactivity and its contribution to neuronal loss and disease progression in the PD brain remain unclear.

Being the major constituent of LBs, aggregated α-synuclein has been extensively studied as a causative factor in PD etiology and pathogenesis [6]. Under normal conditions, α-synuclein is predominantly localized at the presynaptic nerve terminals where it possibly regulates synaptic plasticity, membrane remodeling and neurotransmitter release [7, 8]. Apart from acting in the cytoplasm of cells, neuron-derived α-synuclein species are secreted into the extracellular space where they can be taken up by nearby neurons, thereby promoting disease propagation along interconnected neuronal networks [9]. In addition, extracellular α-synuclein can trigger the NOD-, LRR- and pyrin domain-containing 3 (NLRP3) inflammasome signaling in microglia in a conformation-specific manner [10]. As a result of this activation, the phagocytic capacity of microglia is increased, ultimately leading to α-synuclein degradation through an autophagy-like process that requires the receptors TLR2 and p62 [11, 12]. Studies using pre-formed fibrils (PFFs) have confirmed that PFF treatment induces microglial activation towards the M1 phenotype and triggers the release of pro-inflammatory cytokines [13]. Such microglial activation is thought to subsequently induce a neurotoxic astrocytic phenotype, the A1 neurotoxic reactive astrocytes, further promoting neuroinflammation [14]. In the same line, exogenously added PFFs can be internalized by human or rodent astrocytes, promoting neurotoxic astrocyte activation [15]. Although the resulting neuroinflammation can initially act as a dynamic mechanism to eliminate the potential toxic insult, the sustained inflammation can gradually induce glial-dependent synaptic loss and neuronal death. However, the exact cell-produced α-synuclein conformers that contribute to glial activation in vivo and the molecular pathways they stimulate to establish an immune response are still unknown.

In the current work, we set out to address the roles of α-synuclein conformers in the development, maintenance and progression of non-motor symptoms that appear early in the disease and are mostly related to glial activation and neuroinflammation prior to neurodegeneration. We focused on the striatum, a brain area that has a pivotal role early in the disease and exhibits neuroinflammatory responses in the absence of cell death. The significance of the striatum in PD is also underlined by experimental and clinical evidence supporting a retrograde degeneration of dopaminergic neurons starting from the striatal axon terminals [16, 17]. In fact, dopaminergic denervation is greater in the striatum than in the SNpc in early disease, suggesting that the neocortex can be the area of initial neuronal dysfunction in some PD cases [18, 19]. In support of this mechanism, automatic and habitual performance that depends on the caudal putamen is greatly impaired in PD patients, often preceding PD diagnosis for several years [20].

To clarify the specific α-synuclein species that serve as the inflammatory signals and elucidate the molecular mechanisms through which these signals are transduced to immune and neuronal responses, we performed a series of genetic and biochemical analyses in the striatum of adult A53T α-synuclein transgenic mice (A53T Tg), a PD model with neuron-specific moderate expression of the human A53T α-synuclein variant under the control of the prion promoter [21, 22]. In these mice, the onset of α-synuclein expression starts during development, reaching a 2–3-fold increased expression in early adulthood (2–3 months of age). This pattern of increase of α-synuclein monomer allows the gradual oligomerization and accumulation of various conformers in vivo in a time-dependent manner.

We show here that these mice are characterized by sustained inflammation and intense astrocyte activation that is correlated with the presence of aberrant α-synuclein oligomeric conformers also found in post-mortem brain tissue samples from PD patients. Our data indicate that such α-synuclein oligomers, but not monomers, can activate microglia through induction of the p38^MAPK^/ATF2/7 pathway, which in turn results in an unconstrained activation of the NF-κB pathway in astrocytes. These cellular responses potently drive an up-regulation of astrocytic low-threshold T-type Ca_v_3.2 voltage gated Ca^2+^ channels (VGCCs) leading to the Ca^2+^-dependent release of the neuroprotective protein, insulin growth factor (IGF) binding protein like 1 (IGFBPL1). Our work suggests that such Ca_v_3.2 induction and the subsequent IGFBPL1 secretion from astrocytes could act as a compensatory mechanism against the damaging immune responses triggered by α-synuclein oligomers, thus highlighting the role of astrocytes as neuroprotective mediators in neurodegenerative disorders.

## Materials and methods

### Study design

This study was designed to determine the role of α-synuclein in neuroinflammation in mouse and human brains. We performed experiments to address: (i) the specific α-synuclein species associated with neuroinflammatory responses, (ii) the signaling pathways involved in α-synuclein-induced activation of microglia and astrocytes, and (iii) the changes in astrocyte calcium signaling and secretion upon neuroinflammation. For in vivo studies, age-matched homozygous A53T Tg mice and their wild-type (WT) littermates were randomly used for all experiments. To establish the numbers of mice required for each technique used, three basic parameters were taken into consideration, ethical limitations (homozygous A53T mice exhibit low fertility and are reproduced through crossbreeding of heterozygous A53T mice), method accuracy and reproducibility, and potential requirement for age-grouping. Animal numbers were determined by the investigators based on previous experience. Mice were humanely euthanized, and all experimental procedures were carried out in accordance with local institutional animal ethics approvals. Whenever possible, a brain tissue was used for multiple techniques. Cell culture experiments were performed using at least three biological replicates and each experimental condition was assessed in triplicate in addition to pilot optimization studies for dose and time-point determination.

### Human brain samples

The use of human brain material was approved under the protocol number 46/07-01-2020 by the Bioethics Committee of Biomedical Research Foundation Academy of Athens. Post-mortem tissues from 8 PD patients and 8 non-PD control individuals (Additional file 1: Table S1) corresponding to the putamen and the caudate nucleus were obtained from the PD UK Brain Bank. Human samples were stored at − 80 °C until further use for protein and RNA extraction.

### Mice

Adult homozygous A53T Tg C57BI/C3H mice (line M83-RRID: IMSR_JAX:004479) and WT littermates were used at 4–11 months of age. The generation and phenotypes of these mice have been described previously [22]. The release of α-synuclein does not show sex difference as indicated by our previous in vivo study using microdialysis to monitor α-synuclein secretion in living mice of either sex [23]. Considering this, we have used mice of mixed sex (61% and 50% females in WT and A53T Tg mice, respectively) throughout our study. The characteristics of the mouse groups are described in Additional file 1: Table S2. Animals were housed in the animal facility of the Biomedical Research Foundation of the Academy of Athens in a room with a controlled light–dark cycle (12 h light–12 h dark) with continuous access to food and water. All animal procedures were approved by the National Ethics Committee for Animal Welfare (Protocol Numbers 2143/14-05-18 and 656899/03-08-21).

### Cell lines

Human neuroblastoma SH-SY5Y cells were cultured in the RPMI 1640 medium and maintained at 37 °C in a humidified 5% CO_2_ environment. The medium was supplemented with 10% (*v*/*v*) heat-inactivated fetal bovine serum (FBS), 1% antibiotic/antimycotic (10,000 units/ml of penicillin, 10,000 µg/ml of streptomycin, and 25 µg/ml of amphotericin B) and 1% L-glutamine. Stable tet-off SH-SY5Y cells inducibly over-expressing the WT human α-synuclein were generated as previously described [24]. Cells were maintained in 250 μg/ml G418 and 50 μg/ml Hygromycin B. Over-expression of α-synuclein was achieved by culturing cells in the absence of doxycycline (Dox) (− Dox cells). Basal levels of α-synuclein expression (control cells) were maintained by the addition of 1 μg/ml Dox in the culture medium (+ Dox cells). All media and supplements were purchased from Life Technologies, Gibco™ (NY, USA).

### Collection of conditioned medium (CM)

CM was collected from SH-SY5Y cells maintained in the presence or absence of Dox as previously described [25]. Briefly, cells were cultured in medium containing 1% FBS for 24 h, then culture supernatant was removed and centrifuged at 4000×*g* for 10 min at 4 °C. When indicated, CM was shaken with 0.4 μM Congo Red (Sigma-Aldrich) or vehicle for 4 h at 4 °C. CM was concentrated using 3-kDa cut-off Amicon filters.

Immunodepletion of CM was performed as described [25]. In brief, 1 μg of mouse monoclonal anti-α-synuclein (BD Transductions, NJ, USA) was used to immunodeplete 1 ml of CM from SH-SY5Y cells cultured in the absence of Dox. The immunodepleted CM was 2.5 × concentrated using 3-kDa cut-off Amicon filters and applied to mouse primary microglia. Control immunodepletion was performed with mouse monoclonal anti-dopamine β-hydroxylase (DBH) antibody (Santa Cruz, TX, USA).

### Quiescent primary astrocytes

Quiescent primary astrocytes were isolated from 1-day-old pups and cultured as previously described [26]. Briefly, brains were dissected in ice-cold Hank's Balanced Salt Solution (HBSS) (14180, ThermoFisher, Gibco™) and the olfactory bulbs, brainstem, cerebellum and meninges were removed. Tissue was digested in 10 μg/ml DNase (DN25, Sigma-Aldrich, MA, USA) and 0.0625 g/l trypsin (T4674, Sigma) in Dulbecco’s Modified Eagle’s medium (DMEM) supplemented with 1% penicillin/streptomycin for 20 min at 37 °C. The medium was removed and the tissue was washed twice with DMEM supplemented with 10% FBS and 1% penicillin/streptomycin. The tissue was triturated in DMEM/10%FBS and the homogenate was centrifuged at 400×*g* for 10 min. The pellet was resuspended in DMEM and the cells were plated in a 25 cm^2^ flask coated with 0.01 mg/ml Poly-*L*-Lysine (P4707, Sigma-Aldrich) and maintained at 37 °C in a humidified 5% CO_2_ environment for 1 h. The DMEM was then replaced by Astrocyte Base Medium (ABM) consisting of 50% DMEM with low glucose (without pyruvate, glutamine and phenol red) and 50% Neurobasal-A (without sodium pyruvate and *D*-glucose) supplemented with 0.055 mg/ml sodium pyruvate (11360070, ThermoFisher, Gibco™), 0.1 mg/ml bovine serum albumin (BSA) (A9418, Sigma-Aldrich), 2 mM Glutamax (35050061, ThermoFisher, Gibco™), 20 μg/ml transferrin (T8158, Sigma-Aldrich), 16 μg/ml Putrescine Hydrochloride (P5780, Sigma-Aldrich), 40 ng/ml Sodium Selenite (S5261, Sigma-Aldrich), 5 μg/ml *N*-Acetyl Cysteine (A9165, Sigma-Aldrich), 0.5 mg/ml *D*-glucose (158,968, Sigma-Aldrich), 60 ng/ml progesterone (P8783, Sigma-Aldrich), 5 ng/ml fibroblast growth factor-2 (FGF2) (100-18B, Peprotech, NJ, USA) and 5 ng/ml epidermal growth factor (EGF) (GMP100-15, Peprotech). The cells were maintained in ABM at 37 °C in a humidified 5% CO_2_ environment. The medium was half changed every 3 days. For astrocytic culture, when cells reached ~ 80% confluency, the flask was shaken in an orbital incubator at 200 rpm for 2 h at 37 °C to remove resident microglial cells. For mixed glial cultures, microglial cells were not removed. For transient transfection, astrocytes were transfected using the calcium phosphate method. For each 25 cm^2^ flask, 6 μg of a1Ha-pcDNA3 plasmid, a gift from Edward Perez-Reyes (Addgene plasmid #45809; http://n2t.net/addgene:45809; RRID: Addgene_45809), was used for transfection. The cells and the CM were collected 48 h post transfection.

### Primary microglia

Mouse primary microglia were isolated from mixed glial cultures maintained in DMEM supplemented with 10% FBS for 10 days. T75 flasks containing mixed glia were shaken in an orbital incubator at 280 rpm for 5 h at 37 °C to detach resident microglial cells. The medium was collected and centrifuged at 400×*g* for 10 min and the pellet was resuspended in DMEM/10% FBS. The cells were plated in coverslips coated with 0.01 mg/ml Poly-*D*-Lysine.

### Cell treatments

Primary astrocytes were treated with 5 ng/ml interleukin 1β (IL-1β) (ΑF-211-1113, Peprotech) with or without 20 μM Bay 11-7085 (14795, Cayman) or 20 ng/ml tumor necrosis factor α (TNFα) (AF-315-01A, Peprotech), or 1 ng/ml interferon γ (IFNγ) (AF-315-05, Peprotech) in ABM without FGF2/EGF for the indicated time points. Prior to treatment, the cells were deprived of growth factors for 6 h. Mixed astroglial cultures were treated with 300 ng/ml PFFs in ABM medium for 3 h. The PFFs were prepared as previously described [27].

Primary microglia were treated with SH-SY5Y CM (concentrated 2.5 ×) for 4 h at 37 °C. Prior to treatment, microglia were cultured in DMEM containing 1% FBS for 24 h. SH-SY5Y cells were treated with 50 nM pituitary adenylate-cyclase-activating polypeptide (PACAP) (AS-22519, Anaspec) or 100 ng/ml IFNα for the indicated time points.

### RNA extraction, RNA sequencing and Quantitative PCR

Total RNA extraction was carried out using the TRIzol Reagent according to the manufacturer’s instructions. qPCR was performed using specific primers for each gene (Additional file 1: Table S3) and analyzed using the comparative Cycle threshold (CT) method 2^−ΔΔCT^. RNASeq analysis was performed in duplicates in the Greek Genome Center of the Biomedical Research Foundation of the Academy of Athens (BRFAA). The evaluation of the differentially expressed genes (DEGs) was performed by filtering as: {log2FC > 1 or log2FC < − 1} and {*P* value < 0.01}, in which FC = fold-change of reads per kilobase per million. A detailed description of methods for RNA sequencing, bioinformatic analysis and qPCR is included in Additional file 1: Supplementary Methods.

### Immunofluorescence labelling

All experimental procedures used for immunocytochemistry, immunohistochemistry and immune-electron microscopy are thoroughly described in Additional file 1: Supplementary Methods. The antibodies and appropriate dilutions used for these methods are listed in Additional file 1: Table S4.

### Immunoblotting

Denaturing SDS-PAGE electrophoresis and immunoblotting were performed according to standard protocols. Modifications and antibodies used are described in detail in Additional file 1: Supplementary Methods and Table S4.

### Live calcium imaging

For live-calcium imaging, all manipulations were performed in darkness at 37 °C. Briefly, primary astrocytes were incubated with 2 μΜ Fura-2AM (F1201, Invitrogen) in ABM medium for 30 min. Fura-2AM was removed and cells were cultured in their conditioned medium for 3 h. Next, the cells were incubated in the low K^+^/Ca^2+^ buffer (129 mM NaCl, 5 mM KCl, 1 mM MgCl_2_, 30 mM glucose,1% BSA, and 25 mM HEPES) for 45 min. Before Ca^2+^ measurements, the cells were washed once with the low K^+^/Ca^2+^ buffer and positioned in an inverted microscope (Nikon TE 2000U fluorescence microscope with flat stage) coupled to an intensified CCD camera (PTI-IC200) carrying the Image Master software package (SN 41N50199-21056). To assess the effects of IL-1β, astrocytes were treated with 5 ng/ml IL-1β for 5 min with or without prior exposure to 10 μM NiCl_2_ for 2 min, followed by 50 mM KCl. Fluorescence images were obtained at 350 and 380 nm excitation and 510 nm emission and imaging analysis was performed using the FIJI software (MD, USA).

### Confocal microscopy

Fluorescent images were obtained with a Leica SP5-II confocal microscope and processed by the FIJI [28] or the IMARIS (Bitplane, UK) software. Confocal images were captured using a 20 × or 63 × water immersion objective by sequential scanning of each channel with a screen resolution of 1024 × 1024 pixels. Image settings were adjusted over a negative control section that was incubated only with secondary antibodies to subtract non-specific signals and tissue auto-fluorescence.

### Fluorescence intensity measurements

#### Single-cell fluorescence

Fluorescence intensity was quantified using the FIJI [28] and the IMARIS softwares. FIJI was used to subtract the image background by using the rolling ball algorithm [29]. Next, the image stacks were imported to IMARIS for 3D reconstruction. For Ca_v_ fluorescence quantification, the IMARIS surface tool was used and the channel that represented GFAP^+^ astrocytes was chosen to create 3D surfaces. Using the mask tool, a new masked channel was created that consisted only of Ca_v_^+^ staining, present in GFAP^+^ surfaces. The mean fluorescent intensity of the masked channel was obtained from the statistics tab of IMARIS for each GFAP^+^ surface. The quantification of single-cell fluorescence was performed by assessing one Wt–A53T Tg pair per experiment. For every such pair, at least 5 sections spanning the striatum per genotype were stained in parallel and subjected to confocal imaging. Four different images were captured per section (≥ 20 images per genotype per pair) and the Ca_v_ fluorescence intensity was measured in all astrocytes present in each image. In a similar way, for phospho-p38 quantification, DAPI was used to create surfaces in IMARIS and phospho-p38 fluorescence intensity was quantified in the nucleus (stained with DAPI) of Iba1^+^ cells. CD68 fluorescence intensity was measured by building a surface of CD68-positive staining only in Iba1^+^ cells.

#### Total cell fluorescence

Iba1^+^ cells were counted in all z-stacks using the cell counter feature of FIJI [28]. To determine the size of the cell soma of Iba1^+^ cells, the major and minor axes of microglial cells were measured manually in FIJI [28]. For the quantification of mean Iba1^+^ intensity, the IMARIS software was used to measure the total mean fluorescence intensity upon background subtraction.

### 3D imaging and morphometric analysis of astrocytes

Astrocytic morphometric analysis was performed using the IMARIS software. Astrocytes were randomly selected from two different sections of either WT or A53T Tg mice. Immunofluorescence staining was performed using an antibody against GFAP to mark astrocytes in the absence of α-synuclein labeling. Image stacks from mouse striatum were imported to the IMARIS software and GFAP-immunostained cells across the 30-μm thick section were chosen for 3D astrocyte reconstruction. The astrocytic cellular processes were traced using a semi-automatic filament tracing tool followed by manual correction to achieve total length filament reconstruction. Total branches, filament length, the number of primary processes leaving the soma and the number of Scholl intersections at 25 μm were quantified for each reconstructed astrocyte.

### Protein extraction

Mice were sacrificed by isoflurane and brains were quickly collected. Tissues of the cortex, midbrain, striatum, hippocampus, and brainstem were carefully dissected and washed extensively with PBS to remove residual blood. Mouse and human brain tissues were homogenized with a Teflon glass homogenizer in 50 mM Tris/HCl (pH 6.8) and 1 mM EDTA buffer supplemented with a mixture of protease and phosphatase inhibitors (1 μΜ pepstatin A, 1 μΜ leupeptin and 0.15 μΜ aprotinin, and phosphatase inhibitor cocktail (A32957, Roche, Basel, Switzerland)). Protein extraction was accomplished by the addition of 1% (*w*/*v*) 3-[(3-cholamidopropyl) dimethylammonio]-1-propane-sulphonate (CHAPS) (A1099, AppliChem, Darmstadt, Germany) in the lysis buffer and incubation for 15 min on ice. For the extraction of Ca_v_ subunits and proteins from cell lysates, tissues or cell pellets were homogenized in RIPA buffer (150 mM NaCl, 50 mM Tris pH 7.4, 1% NP-40, 0.5% deoxycholic acid, 0.1% SDS) supplemented with protease and phosphatase inhibitors and incubated for 30 min on ice. For the extraction of Ca_v_ subunits from cell lysates, the homogenization buffer contained 1% SDS-Triton. The cell lysate was incubated for 30 min at room temperature and centrifuged at 13,000 rpm for 30 min at 18 °C. Protein concentration was determined using the Bradford method or using the DC assay kit. BSA was used for the preparation of the standard curve.

### Cytokine measurement

The brain was dissected, and the striatum and cortex were isolated. Fifty milligrams of tissue per 1 ml of sterile HBSS containing protease inhibitors (Sigma-Aldrich) were homogenized using a T-8 homogenizer (IKA-WERKE, Staufen, Germany). The homogenates were centrifuged at 400×*g* for 15 min at 4 °C and the supernatants were collected and stored at − 80 °C. The levels of TNF-α, IL-1β and IL-10 in the mouse striatum homogenates were measured using commercially available ELISA kits (R&D Systems, MN, USA) according to the manufacturer’s instructions. TNFα levels in microglial CM and in the mouse cortex were measured using a commercially available ELISA Kit (900-M54, Peprotech) according to manufacturer’s instructions except that HRP chemiluminescence was assessed by adding the Luminata Crescendo chemiluminescent substrate (ELLUR0100, Millipore).

### Secretomics

#### Sample preparation

All procedures for sample preparation were performed at room temperature. CM (9 ml per sample) was concentrated with 3 kDa MWCO Amicon Ultra Centrifugal filter devices (Merck Millipore) to a final volume of 30 μl. Protease inhibitors were added, and protein concentration was determined with the Bradford Assay. The concentrated samples were processed with the filter-aided sample preparation (FASP) method as described previously [30], with minor modifications [31]. Briefly, 200 μg of each sample was mixed with lysis buffer (0.1 M Tris–HCl pH 7.6 supplemented with 4% SDS and 0.1 M DTE) and buffer exchange was performed in Amicon Ultra Centrifugal filter devices (0.5 ml, 30 kDa MWCO; Merck Millipore) at 14,000×*g* for 15 min. Each sample was then diluted with urea buffer (8 M urea in 0.1 M Tris–HCl pH 8.5) and centrifuged. The concentrate was diluted again with urea buffer and centrifugation was repeated. Alkylation of proteins was performed with 0.05 M iodoacetamide in urea buffer for 20 min in the dark followed by centrifugation at 14,000×*g* for 10 min. Additional series of washes were conducted with urea buffer (2 times) and 50 mM NH_4_HCO_3_ pH 8.5 (2 times). Tryptic digestion was performed overnight in the dark, with a trypsin-to-protein ratio of 1:100. Peptides were eluted by centrifugation at 14,000×*g* for 10 min, lyophilized and stored at –80 °C until further use.

#### Liquid chromatography-mass spectrometry (LC–MS)/mass spectrometry (MS) analysis

Samples were resuspended in 200 μl mobile phase A (0.1% FA in water). A 5 μl volume was injected into a Dionex Ultimate 3000 RSLS nano flow system (Dionex, Camberly, UK) configured with a Dionex 0.1 × 20 mm, 5 μm, 100 Å C18 nano trap column with a flow rate of 5 µl/min. The analytical column was an Acclaim PepMap C18 nano column 75 μm × 50 cm, 2 μm 100 Å with a flow rate of 300 nl/min. The trap and analytical columns were maintained at 35 °C. Mobile phase B was 0.1% formic acid in acetonitrile. The column was washed and re-equilibrated prior to each sample injection. The eluent was ionized using a Proxeon nano spray ESI source operating in the positive ion mode. For MS analysis, a Q Exactive Orbitrap (Thermo Finnigan, Bremen, Germany) was operated in the MS/MS mode. The peptides were eluted under a 240 min gradient from 2% (B) to 80% (B). Gaseous phase transition of the separated peptides was achieved with positive ion electrospray ionization applying a voltage of 2.5 kV. For every MS survey scan, the top 10 most abundant multiply charged precursor ions between m/z ratio 300 and 2200 and intensity threshold 500 counts were selected with FT mass resolution of 70,000 and subjected to HCD fragmentation. Tandem mass spectra were acquired with FT resolution of 35,000. Normalized collision energy was set to 33 and already targeted precursors were dynamically excluded for further isolation and activation for 30 s with 5 ppm mass tolerance.

#### MS data processing, quantification and statistical analysis

Raw files were analyzed with the Proteome Discoverer 1.4 software package (Thermo Finnigan), using the Sequest search engine and the Uniprot mouse (*Mus musculus*) reviewed database, downloaded on May 10, 2021, including 17,073 entries. The search was performed using carbamidomethylation of cysteine as static and oxidation of methionine as dynamic modifications. Two missed cleavage sites, a precursor mass tolerance of 10 ppm and fragment mass tolerance of 0.05 Da were allowed. False discovery rate (FDR) validation was based on *q* value (target FDR: 0.01). Label-free quantification was performed by utilizing the precursor ion area values exported from the total ion chromatogram as defined by the Proteome Discoverer 1.4 software package. Output files from Proteome Discoverer were processed with R programming language for statistical computing (version 4.0.3). Raw protein intensities for each sample were subjected to normalization according to X’ = X/Sum(Xi) ∗ 106. Statistical analysis was performed with the non-parametric Mann–Whitney test. Proteins with ratio ≥ 1.5 (up-regulated) or ≤ 0.67 (down-regulated) were considered differentially expressed.

### Statistical analysis

Data analysis was carried out using the GraphPad Prism 4 software. All measurements were analyzed with descriptive statistics and results are presented as mean ± SEM. Shapiro–Wilk test was used to determine whether the variables were normally distributed. For *N* ≥ 30, no normality test was performed, and distributions were considered normal. For normal distributions, a two-tailed Student’s *t* test was used and for non-normally distributed variables (Shapiro–Wilk test, *P* < 0.05), Mann–Whitney U test was performed. The *P* value threshold was set at < 0.05.

## Results

### α-Synuclein oligomers are correlated with persistent neuroinflammation in mouse brains

**Fig. 1:**
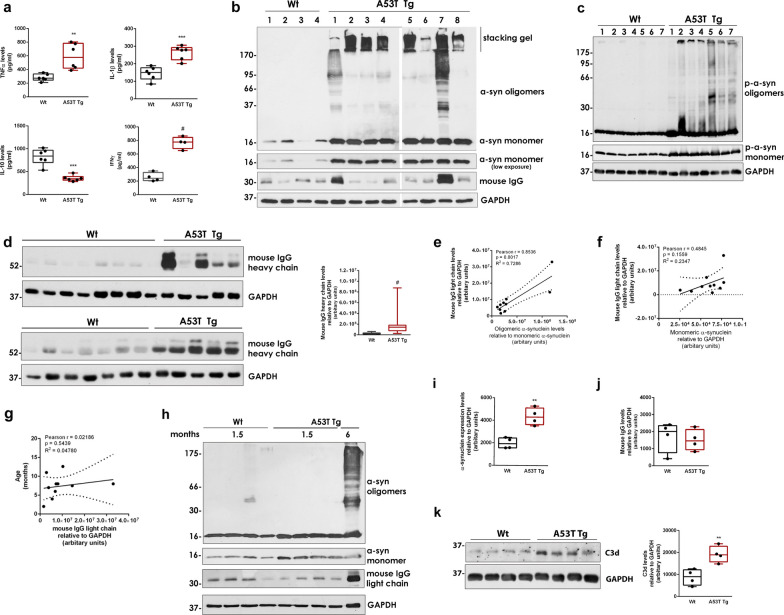
Pathological α-synuclein oligomers are associated with inflammatory responses in A53T mouse striatum. **a** TNFα (**P = 0.0029), IL-1β (***P = 0.0003), IL-10 (***P = 0.0001) and IFNγ (^#^*P* < 0.0001) levels in striatal homogenates determined using cytokine-specific ELISA assays (*N* ≥ 4 per genotype). **b**, **c** Representative western blots of CHAPS-homogenized striatum using antibodies against total (anti-Syn1) and phosphorylated α-synuclein. **d** Immunoblots of mouse IgG heavy chain and densitometric quantification in WT (*n* = 16) and A53T Tg (*n* = 14) mice, ^#^*P* < 0.0001. **e–g** Correlation analysis of mouse IgG light chain in A53T Tg mice with α-synuclein oligomers (**e**), α-synuclein monomers (**f**), and animal age (**g**) after immunoblotting and densitometric quantitation (*n* = 10). **h–j** Western blotting of homogenized striatal tissues from 1.5-month-old WT and A53T Tg mice (*n* = 4 mice per genotype) using antibodies against total α-synuclein (anti-Syn1) and densitometric quantification of monomeric α-synuclein levels (**i**, **P = 0.0019) and IgG levels (**j**, *P* = 0.7219). A six-month-old A53T Tg homogenate was used as reference for the presence of α-synuclein oligomers. **k** Representative immunoblots and quantification of C3d (*n* = 4 mice per genotype, **P = 0.0074). In **b–d**,** h** and** k**, GAPDH was used as a loading control. Statistics by Mann–Whitney test in (**d**) and unpaired Student’s *t* test in (**a**, **i**, **j**, **k**)

To understand the contribution of cell-produced α-synuclein to the neuroinflammatory responses in vivo, we used the A53T Tg mouse model [22] and focused our experimental design mainly on the investigation of the striatum, a brain area that is severely affected in PD and plays a pivotal role early in the disease without the involvement of cell death. In contrast to other α-synuclein-based mouse models that are characterized by neuronal deficits in the striatum [32], these mice develop advanced synucleinopathy in the brainstem (and spinal cord), eventually leading to an age-dependent lethal locomotor phenotype albeit at older ages ≥ 12 months. As such, the markers of advanced synucleinopathy in the A53T Tg model remain largely restricted to the brainstem and there is no evidence of synuclein pathology spreading [33, 34]. One advantage of this model is that the moderate expression levels of α-synuclein are comparable with those in human patients. Indeed, qPCR and western blotting analysis showed that the adult homozygous A53T Tg mice express high levels of human *SNCA* mRNA irrespective of their age or sex, which results in a moderate (three-fold) elevation of monomeric α-synuclein protein level in the striatum of A53T Tg mice compared with their WT littermates (Additional file 1: Fig. S1a–d). Further assessment of cytokine levels by ELISA showed a reduction in the anti-inflammatory cytokine interleukin (IL) 10, as well as elevated levels of the pro-inflammatory cytokines TNFα, IL-1β and IFN-γ, indicating an established neuroinflammation in the A53T brain (Fig. 1a).

Considering the intrinsic propensity of α-synuclein to self-aggregate, elevated levels of monomers enhance the probability of local accumulation and oligomerization of the protein towards β-sheet-rich oligomeric multimers of high molecular weight. To understand the role of the different α-synuclein conformers in the establishment of neuroinflammation, we assessed the levels of α-synuclein using the zwitterionic detergent CHAPS to preserve the native structure of all α-synuclein assemblies in the striatal tissue. CHAPS homogenization and immunoblotting revealed the presence of α-synuclein oligomers with variable levels and aggregation status, as indicated by their partial retainment into the stacking gel (Fig. 1b). The presence of these oligomers in A53T Tg but not WT mice was further verified using a different α-synuclein antibody (Additional file 1: Fig. S1e). We also found that the α-synuclein oligomers in A53T Tg mice were intensively phosphorylated, suggesting that they might possess pathological activity (Fig. 1c). In contrast, the levels of monomeric α-synuclein remain unaltered in all A53T Tg mice tested (Fig. 1b, c and Additional file 1: Fig. S1d). Interestingly, we observed that the formation of α-synuclein oligomers coincided with a significant increase in endogenous mouse IgG antibodies, indicative of active immune reaction (Fig. 1b, d). Quantification of the α-synuclein oligomers relative to the most abundant monomeric form revealed a positive correlation between oligomeric α-synuclein and IgG levels, implying a causative link of these conformers with the observed immune response (Fig. 1e). We found no correlation between monomeric α-synuclein or animal age and IgG levels (Fig. 1f, g). To further support an association between α-synuclein oligomers and immune activity in the striatum of A53T Tg mice, we analyzed A53T Tg mice of young age (1.5 months old) when the mice already exhibit a more than two-fold increase in total α-synuclein levels but an absence of α-synuclein oligomers (Fig. 1h, i). Assessment of the endogenous IgG levels in these mice did not reveal any differences between WT and A53T Tg genotypes, suggesting that the immune response observed in the older mice could be triggered or enhanced by the accumulation of α-synuclein oligomers (Fig. 1h, j).

Neuroinflammation can be the result of neuronal cell loss. We and others have addressed the issue of degeneration in the striatum of A53T Tg mice, not only in relation to neuronal loss but also in terms of synaptic integrity [11, 23, 34]. Neuronal death is partly triggered by complement C1q and C3 tagging, an established mechanism for the selective elimination of unwanted or damaged synapses by phagocytic cells [35, 36]. Even though neuronal C1q and C3 are normally downregulated in the adult CNS, the complement-dependent synaptic removal is thought to be aberrantly activated in Alzheimer’s disease by Aβ oligomers, thereby contributing to early synaptic loss [37]. Consistent with this observation, we detected increased levels of C3d in the striatum of A53T Tg mice by immunoblotting (Fig. 1k). Subsequent immunofluorescence experiments indicated that the C3 and C1q complement proteins were indeed localized in some striatal neurons, suggesting activation of the synapse-tagging mechanism in our mouse model (Additional file 1: Fig. S1f, g). Despite this activation, quantification of tyrosine hydroxylase (TH), a marker for dopaminergic neuronal terminals, synaptobrevin-2 (SYB2), a marker of synaptic integrity, and the pan-neuronal protein, neurofilament (NFL), showed no evidence of neurodegeneration at all ages assessed (5–11 months old) (Additional file 1: Fig. S1h). Further, we found no differences in the levels of PSD95, an established marker of denervation [38] between WT and A53T Tg mice, suggesting that the observed inflammation does not originate from or lead to synaptic loss or cell death (Additional file 1: Fig. S1i).

Overall, our data suggest that the accumulation of pathology-related α-synuclein oligomers, but not monomers, is associated with neuroinflammation and neuronal complement tagging in the striatum of A53T Tg mice.

### Cell-produced α-synuclein oligomers, but not monomers, can induce microglial activation

**Fig. 2:**
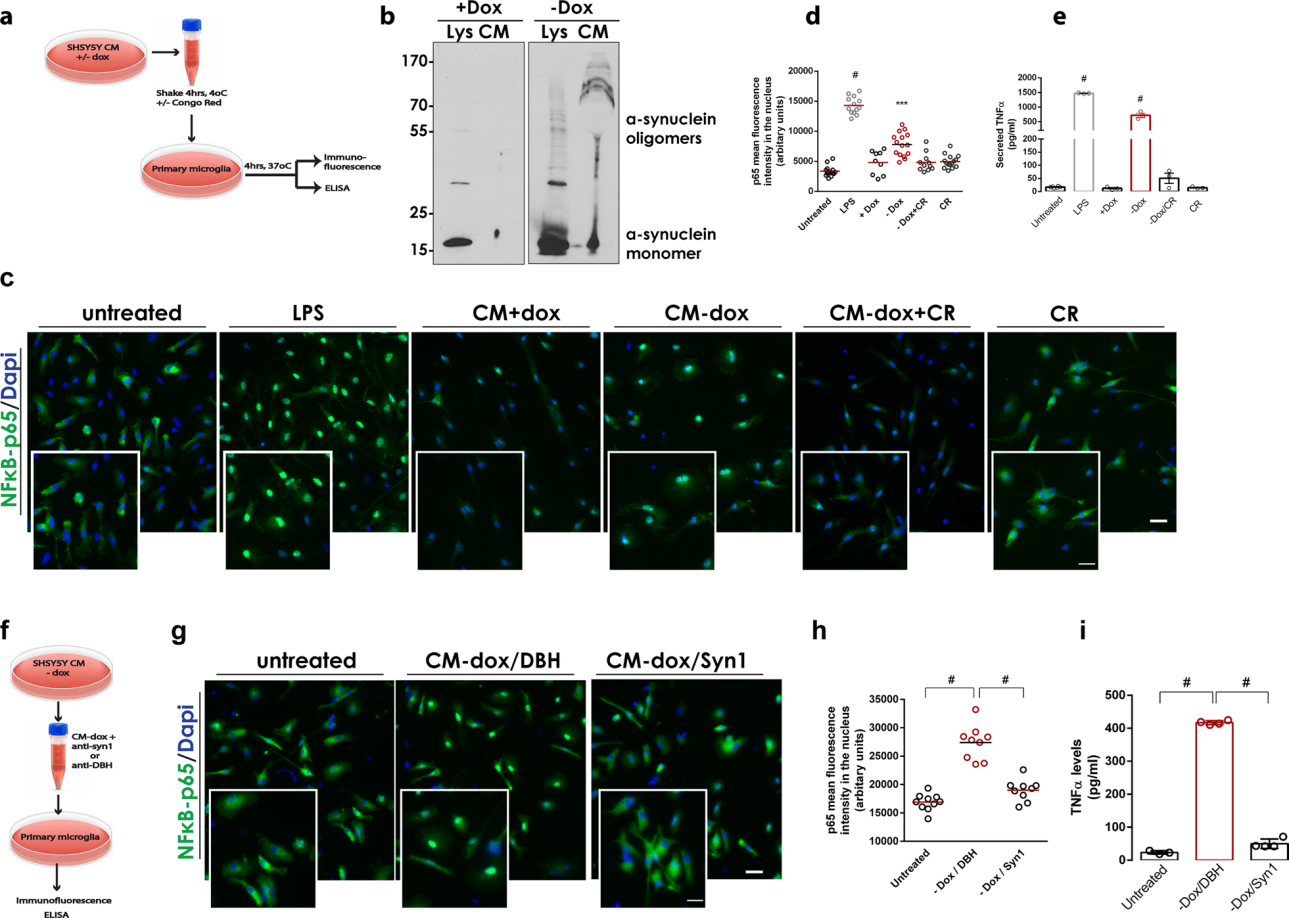
Cell-produced α-synuclein oligomers, but not monomers, can activate primary microglia. **a, f** Schematic representations of the experiments with primary microglia treated with CM from SH-SY5Y α-synuclein expressing cells. **b** Immunoblot showing the presence of cell-secreted (CM) and intracellular (Lys) α-synuclein conformers in the absence of doxycycline (− Dox). **c, g** Representative images of primary microglia immunostained with a specific antibody against NF-κB. DAPI was used as a marker for nuclei. Magnification of the boxed area is shown for each merged image. Scale bar: 20 μm (50 μm for the magnified images). **d, h** Quantification of mean fluorescence intensity of nuclear NF-κB (*N* ≥ 9 for all treatments). **e, i** Measurement of secreted TNFα from primary microglia upon indicated treatments (*n* = 3 biological replicates). In **d**, **e**, **h**, and **i**, statistics was performed by one-way ANOVA followed by Tukey’s multiple comparisons test. In **d**, ^#^*P* < 0.0001 for untreated vs LPS, ***P = 0.0001 for + Dox versus − Dox. In **e**, ^#^*P* < 0.0001 for untreated versus LPS and + Dox versus  − Dox. In **h** and **i**, ^#^*P* < 0.0001

To address the causal role of aberrant α-synuclein oligomers in neuroinflammation beyond the observed correlation in vivo, we investigated whether these conformers can directly activate primary mouse microglia. For this, we used a well-established SH-SY5Y cell system in which the expression of human WT α-synuclein results in the production of soluble oligomers that gradually accumulate in the cell interior, compromising cell homeostasis and viability [24]. In this cellular system, the expression of α-synuclein is induced by the removal of Dox from the culture medium of the cells (− Dox condition). SH-SY5Y cells cultured in the presence of Dox retain only basal levels of α-synuclein expression (+ Dox condition) [24]. We have previously shown that both oligomeric and monomeric α-synuclein are secreted from these cells and can be toxic when applied to healthy neuronal cells [25]. This toxicity was diminished when the secreted α-synuclein conformers were treated with Congo Red, an organic compound that selectively binds to and disrupts β-sheet aggregates [25]. We collected CM from these α-synuclein-expressing SH-SY5Y cells containing secreted α-synuclein conformers and applied the CM onto non-activated primary microglia for 4 h (Fig. 2a). Western blotting analysis confirmed the presence of secreted α-synuclein species (oligomers and monomers) in the CM (Fig. 2b).

To assess microglial activation, we assessed the stimulation of the NF-κB pathway by measuring the p65 protein localized in the nucleus of microglial cells by quantifying single-cell immunofluorescence. Liposaccharide (LPS) was used as a potent inducer of microglial reactivity. Our results clearly showed that the CM containing secreted α-synuclein conformers could activate the NF-κB pathway in microglia (Fig. 2c). To exclude the possibility that other secreted factors contribute to microglial activation, CM from sister SH-SY5Y cultures that express only basal levels of α-synuclein (+ Dox cells) was also applied to microglia but did not induce their activation. Importantly, pre-treatment of the CM with Congo Red abolished the observed microglial activation, suggesting that the β-sheet-enriched α-synuclein multimers are responsible, at least to a great extent, for the observed NF-κB activity (Fig. 2d). Addition of Congo Red alone was used as negative control. Microglial reactivity was further confirmed by measuring the levels of secreted TNFα in microglia CM following treatment with cell-secreted α-synuclein. Our results indicated a robust increase in TNFα levels only following treatment with CM containing secreted α-synuclein conformers (Fig. 2e). Again, pre-treatment with Congo red greatly abolished the TNFα increase, suggesting that the α-synuclein oligomers induce microglial activation (Fig. 2e).

To further verify that microglial activation is not due to other factors secreted along with α-synuclein conformers, we immunodepleted the CM obtained from the α-synuclein-expressing cells (− Dox cells) using the monoclonal Syn1 antibody which can bind both oligomeric and monomeric α-synuclein (Fig. 2f). An antibody of the same type against DBH, a protein which is highly expressed in SH-SY5Y cells, was used as a control of immunodepletion. Immunodepleted CM was applied to mouse primary microglia and activation was assessed by p65 nuclear translocation and TNFα secretion, as above. Our results clearly showed that the selective removal of α-synuclein conformers from the CM largely attenuated microglial activation. In contrast, DBH immunodepletion had no effect on microglial reactivity (Fig. 2g–i).

### A53T microglia and astrocytes exhibit distinct biochemical and morphological alterations

**Fig. 3:**
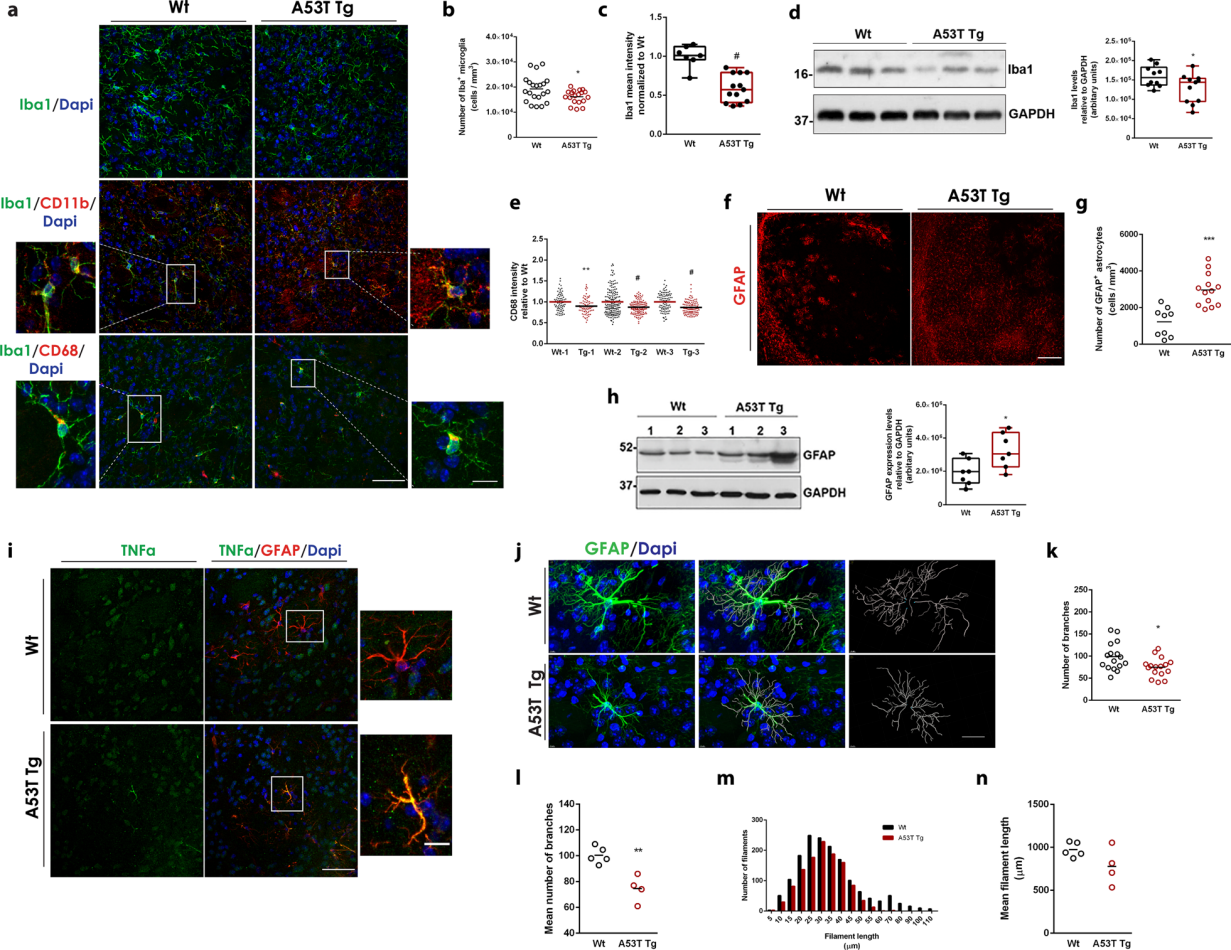
A53T microglia and astrocytes exhibit biochemical and morphological alterations. **a** Representative confocal images of WT and A53T Tg striatal sections stained with antibodies against Iba1, CD11b and CD68. DAPI (blue) was used as a nuclei dye. Scale bar: 50 μm (10 μm for the magnified images). **b** Quantification of the number of Iba1^+^ microglia cells (*n* = 21 and *n* = 19 images from WT and A53T Tg mice, respectively, ^#^*P* = 0.0239) upon immunostaining of coronal brain sections in the striatum of WT and A53T Tg mice with an Iba1-specific antibody. **c** Quantification of the total mean fluorescence intensity of Iba1^+^ cells (*n* = 7 and *n* = 13 images from WT and A53T Tg mice, respectively, ^#^*P* < 0.0001) stained as above. **d** Immunoblotting analysis of striatum homogenates from WT and A53T Tg mice using a specific antibody to Iba1 and densitometric quantification (*n* = 10 and *n* = 11 for WT and A53T Tg mice, respectively, *P = 0.0438). GAPDH was used as a loading control. **e** Quantification of the mean fluorescence intensity of CD68 in individual Iba1^+^ cells in three independent pairs (1, 2, 3) of WT and A53T Tg mice; **P = 0.0018 for pair 1, ^#^*P* < 0.0001 for pairs 2 and 3, *n* ≥ 74 astrocytes for each genotype. **f** Representative confocal images of WT and A53T Tg striatal sections stained with a specific antibody for GFAP. Scale bar, 200 μm. **g** Quantification of the number of GFAP^+^ astrocytes in the striatum following immunostaining and confocal imaging (*n* = 9 and *n* = 13 images for WT and A53T Tg mice, respectively, ***P = 0.0001). **h** Western blot and GFAP quantification in striatal homogenates (*n* = 7 mice per genotype, *P = 0.219). **i** Representative confocal images of WT and A53T Tg striatal sections stained with specific antibodies for TNFα and GFAP. Scale bar: 50 μm (10 μm for the magnified images). **j** Representative images of 3D reconstructed GFAP-immunoreactive astrocytes from WT and A53T Tg striatum, using the IMARIS software. DAPI was used for nuclei staining. Scale bar, 20 μm. **k, l** Quantification of the number of branches; **k** from 16 astrocytes per genotype, *P = 0.0174, and (**l**) pre-averaged per animal (*n* = 5 and *n* = 4 for WT and A53T Tg mice, respectively, **P = 0.0024). **m, n** Quantification of filament length; (**m**) length distribution from 16 astrocytes per genotype and (**n**) pre-averaged per animal (*n* = 5 and *n* = 4 for WT and A53T Tg mice, respectively, *P* = 0.1058). For all comparisons statistics were performed by unpaired Student’s* t* test

To further characterize neuroinflammation associated with α-synuclein, we investigated the activation of the resident glial cells, microglia and astrocytes, that mediate immune responses in the brain. Upon activation, microglia proliferate and rapidly change their morphology from a resting ramified state to an amoeboid-like phenotype with enlarged cell soma and phagocytic capacity [5]. Several protein markers have been used to determine microglial activation; however, associating one single marker with a distinct phenotype is difficult since most of the markers are present in most of the reactive states of microglial cells [39]. We investigated the presence of three protein markers, ionized calcium binding adapter molecule 1 (Iba1), CD11b and CD68, all of which were found to be expressed in the microglia (Fig. 3a). We initially assessed the expression of Iba1, a microglial marker present in both resting and activated microglial cells and found that the number of Iba1^+^ cells was significantly reduced in the striatum of A53T Tg mice (Fig. 3a, b). Subsequent measurement of single-cell Iba1 fluorescence intensity revealed that the A53T microglial cells also expressed lower levels of Iba1 protein at a cellular level (Fig. 3c). The lower levels of Iba1 were further confirmed by western blotting (Fig. 3d). This was consistent with a previous report showing a reduction in Iba1^+^ microglia in a double mutant α-synuclein transgenic mouse model at ages > 6 months [40]. Using Iba1 immunofluorescence, we also assessed the size of the soma of microglia that appeared elliptic in shape. By measuring its major and minor axes we found a wide range of soma sizes which, however, showed similar distributions in both genotypes (Additional file 1: Fig. S2a).

It has been shown that α-synuclein can be engulfed by activated microglia through a TLR4-dependent process termed “synucleinphagy”, during which the autophagy receptor, p62/SQSTM1, is upregulated to promote the lysosomal degradation of the phagocytosed α-synuclein in autophagosomes [12]. To address whether the phagocytic capacity of microglia is advanced in the A53T mice, we quantified the expression of the phagocytic activity marker CD68 in single Iba^+^ cells, and found significantly reduced CD68 in A53T microglia (Fig. 3e). We next detected p62 in striatal sections of WT and A53T Tg mice using immunofluorescence. We observed selective punctate p62 staining in the striatal neurons, but not in Iba1^+^ microglia, in either genotype, indicating that the autophagic degradation process is not enhanced in our setting (Additional file 1: Fig. S2b). To examine whether α-synuclein is internalized by glial cells in vivo, we performed immuno-electron microscopy in striatal sections where the antibody against α-synuclein, Syn1, was labeled with gold nanoparticles and microglia were visualized through an Iba1-DAB reaction. α-Synuclein-positive presynaptic terminals were observed in the vicinity of microglial cells; however, nanogold particles could not be detected in the interior of the DAB-stained microglia in WT or A53T Tg mice (Additional file 1: Fig. S2c). Given the advanced synuclein pathology in the brainstem of A53T Tg mice, we lastly investigated whether the microglial responses could be stimulated by changes in norepinephrine (NE) regulation. NE release controls the phagocytic capacity and modulates the motility of microglia by the selective modulation of adrenergic receptors [41]. Locus coeruleus is the major source of NE in the CNS and dysregulation of the noradrenergic system has been shown to potentiate neuroinflammation in various animal models of neurodegeneration. α-Synuclein can directly interfere with NE production by inhibiting the CRE-mediated transcription of DBH, the rate-limiting enzyme that converts dopamine to NE [42]. To exclude the possibility of microglial activation via attenuation of DBH transcription, we measured DBH levels in the brainstem by immunoblotting. Densitometric quantification showed no changes in the levels of DBH expression between WT and A53T Tg mice (Additional file 1: Fig. S2d).

Collectively, our data show that, in the presence of high levels of oligomeric and monomeric α-synuclein, microglia are characterized by lower Iba1 and CD68 expression levels, suggestive of decreased phagocytosis activity.

Since microglia can induce astrocyte reactivity to exacerbate or attenuate inflammation in response to CNS injury, infection or disease, we next wanted to examine whether astrocytes exhibit signs of activation in the A53T Tg model [43]. To address astrocyte reactivity, we initially measured glial fibrillary acidic protein-positive (GFAP^+^) cells in striatal sections of WT and A53T Tg mice. Using this approach, we found an elevated number of GFAP^+^ astrocytes in the A53T striatum, indicative of astrocyte proliferation (Fig. 3f, g). Western blotting confirmed the increased levels of GFAP (Fig. 3h). To directly examine astrocyte reactivity in our in vivo setting, we performed immunolabelling experiments to detect TNFα in GFAP^+^ astrocytes in striatal sections from WT and A53T Tg mice. Our results showed increased TNFα levels in A53T astrocytes, indicating their activation under conditions of elevated α-synuclein levels (Fig. 3i). Human astrocytes have been shown to possess pathological α-synuclein accumulation in the PD brain [44]. In addition, mutant α-synuclein can be found in astrocytes upon prion promoter-mediated expression [45]. To assess whether mutant α-synuclein is expressed or incorporated in astrocytes in our mouse model, we performed immunolabeling experiments using antibodies against α-synuclein (recognizing both mouse and human α-synuclein) and the astrocyte marker, GFAP. Using this approach, we did not detect α-synuclein in the interior of astrocytic cells either in WT or A53T Tg mice, suggesting that the astrocytes are not activated due to endogenously expressed or accumulated α-synuclein in our setting (Additional file 1: Fig. S2e).

We next performed a detailed comparative morphometric analysis based on the 3D reconstruction of individual GFAP-immunoreactive astrocytes residing within the area of striatum to visualize morphological alterations in the adult astrocytes of A53T Tg mice (Fig. 3j). Since the complexity of astrocytes increases with radial distance from the soma, Sholl analysis [46] was applied to 16 individual astrocytes from WT and A53T Tg mice and the number of filaments, the number of branches, the number of primary processes originating from the soma and the number of process intersections were assessed. Our data indicated that the A53T Tg astrocytes possessed lower numbers of filaments, especially shorter (< 25 μm) and longer (> 50 μm) filaments, and completely lacked the longest (> 60 μm) filaments (Fig. 3m), suggesting retraction of processes. They also had a lower number of branches (Fig. 3k), whereas the number of branches emanating from the soma and the number of process intersections (i.e., the number of intersections between a process and the sphere of 25 μm radius) were similar to WT astrocytes (Additional file 1: Fig. S2f, h). Our analysis was based on the assumption that astrocytes from the same genotype should share similar morphometric features. Indeed, considering that cells were selected in an unbiased way from 5 WT and 4 A53T Tg mice, individual values showed no outliers in the distributions, suggesting the presence of morphologically homogeneous populations of astrocytes in each genotype. Grouping individual values per animal showed similar results, further supporting our initial observations (Fig. 3l, n and Additional file 1: Fig. S2g, i). Taken together, our data suggested that α-synuclein expression in A53T Tg mice induces astrocyte proliferation and activation and can compromise the arbor complexity of astrocytes, leading to a partial retraction of their processes.

### High levels of α-synuclein motivate neuropeptide-, mitogen-activated protein (MAP) kinase (MAPK)- and Ca^2+^-dependent signaling pathways

Our results suggest that α-synuclein oligomers are related to consistent neuroinflammation characterized by increased cytokine release and distinct biochemical and morphological alterations in microglia and astrocytes. To dissect the molecular pathways underlying these changes, we performed RNA sequencing (RNAseq) analysis in WT and A53T Tg mice. Striatum was collected in duplicate for each mouse genotype at 6 months of age and processed for RNA extraction and sequencing using the Illumina HiSeq platform. We assessed the quality and purity of our RNAseq profiles by mapping quality (> 70% of reads for all our samples were mapped). Our analysis revealed 526 DEGs in A53T Tg compared with WT mice (≥ 1.5 fold) (Additional file 2), including 304 up-regulated and 222 down-regulated DEGs. *Prnp* and *SNCA* genes were found to be up-regulated with the highest significance, as expected. Six basic clusters were identified in the heat map (Additional file 1: Fig. S3a). Enrichr-based KEGG pathway enrichment analysis (https://maayanlab.cloud/Enrichr/) highlighted the “neuroactive ligand-receptor interaction”, the “MAPK signaling”, the “calcium signaling” and the “cAMP signaling” pathways as the most affected in the presence of high α-synuclein levels (Additional file 1: Fig. S3b). Cholinergic, dopaminergic and GABAergic synaptic pathways were also significantly altered, indicating changes at the level of neuronal circuits and distorted neurotransmitter release (Additional file 1: Fig. S3b). Of the DEGs that were most significantly altered in the A53T Tg striatum (Additional file 1: Fig. S3c), *Mylk3*, encoding for myocin light chain kinase (MYLK), *Shox2*, encoding for a transcription homeobox regulator, and *Pappa-2*, encoding for pappalysin-2 protease implicated in IGF-1 signaling, were most up-regulated. Nine of these DEGs were selected for qPCR validation. Assessment of the *Adcyap* gene encoding PACAP verified its up-regulation in the A53T mice (Additional file 1: Fig. S3d). PACAP is up-regulated in neurons and astrocytes in response to inflammation and can exert potent anti-inflammatory actions that are protective for neurons [47]. Its mechanism of action is mediated by cAMP signaling, which could probably explain why this pathway was underscored in the RNAseq. Two other DEGs that belong to the “neuroactive ligand-receptor interaction” pathway, *NpY* and *Npsr1*, were also up-regulated in the A53T Tg mice, further supporting our RNAseq data (Additional file 1: Fig. S3e, f). Like PACAP, NpY signaling is suggested to inhibit microglial activation and has a neuroprotective role against neurodegeneration in PD [48]. *Pappa-*2 gene was also found significantly up-regulated (Additional file 1: Fig. S3g). Further, the up-regulation of MYLK, a kinase that phosphorylates myocin to allow the calcium-dependent myocin-actin complex formation and facilitate cell motility, was confirmed at both the mRNA and the protein levels (Additional file 1: Fig. S3h, i). We also assessed four down-regulated DEGs, *Wnt8b*, *Nr4a3*, *Gpx6* and *Scarf2* that were found to be significantly decreased (Additional file 1: Fig. S3l, m). In sum, our RNAseq approach aided in the identification of biological pathways enriched in the list of genes differentially regulated in A53T Tg mice vs WT mice and highlighted the importance of α-synuclein in the regulation of molecular pathways related to synaptic functioning and neuropeptide-, MAPK- and Ca^2+^ signaling in the striatum.

### α-Synuclein conformers are associated with activation of the p38^MAPK^ signaling pathway and advancement of the ATF2/7-dependent transcription in microglia

We next investigated the signaling pathways that could be involved in microglial and astrocytic activation in the presence of α-synuclein conformers. Since our RNAseq data indicated that PACAP mRNA is up-regulated in A53T striatum, we initially examined whether PACAP signaling is activated in vivo. We found that PACAP was robustly expressed in striatal neurons of both WT and A53T Tg, as expected, and was absent from astrocytes (Additional file 1: Fig. S4a). PACAP receptor, PAC1, was observed in both neurons and astrocytes, suggesting that both cell types could be acceptors of PACAP signaling (Additional file 1: Fig. S4b). Astrocytes are responsible for glutamate uptake, and previous studies suggested that increased PACAP activity induces the astrocyte-specific glutamate transporter, Glt-1 or EAAT2 [49]. Assessment of Glt-1 levels in striatal homogenates did not reveal any differences between genotypes, suggesting that the PACAP activity is not significantly enhanced in vivo in A53T mice (Additional file 1: Fig. S4c). To verify this finding, we examined the cAMP/CREB pathway that mediates PACAP signaling. Even though the levels of total CREB were significantly elevated, the lack of CREB phosphorylation in the striatum confirmed that the PACAP signaling was not enhanced in A53T Tg mice (Additional file 1: Fig. S4d, e). In a similar fashion, the contribution of the STAT3 pathway, one of the main signaling pathways in inflammation, was addressed. Again, STAT3 was found to be increased but the phosphorylated STAT3 protein could not be detected (Additional file 1: Fig. S5a, b).

**Fig. 4:**
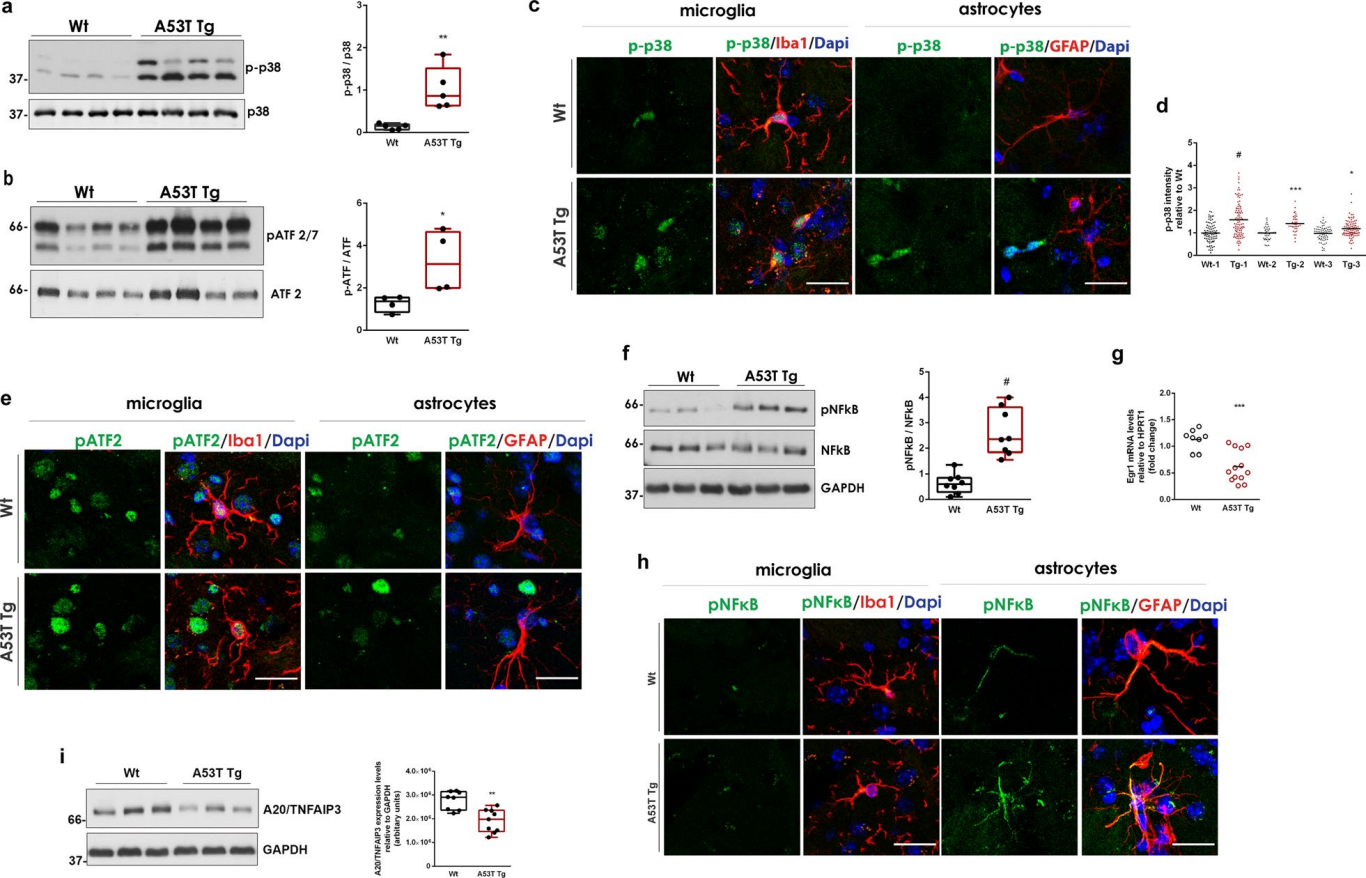
The p38^MAPK^ and the NF-κB pathways are activated in A53T Tg striatum. **a** Immunoblotting analysis and quantification of phospho-p38^MAPK^ levels relative to total p38^MAPK^ expression (*n* = 5 mice per genotype, ^#^*P* < 0.0001). **b** Immunoblotting analysis and quantification of phospho-ATF2/7 levels relative to total ATF2 expression (*n* = 4 mice per genotype, *P = 0.0378). **c, e, h** Representative magnified confocal images of striatal sections co-stained with antibodies against phospho-p38 (**c**), phospho-ATF2/7 (**e**) and phospho-NF-κB (**h**) and Iba1 or GFAP. DAPI (blue) was used for nuclei staining. Scale bar: 20 μm. **d** Measurement of phospho-p38 mean fluorescence intensity in Iba1^+^ cell surfaces (*n* ≥ 35 cells per mouse for three independent Wt-A53T Tg animal pairs, ^#^*P* < 0.0001, ***P = 0.0004, *P = 0.0239). **f** Representative immunoblot and quantification of phospho-NF-κB relative to total NF-κB in striatal homogenates (*n* = 8 mice per genotype, ^#^*P* < 0.0001). **g** qPCR measurement of Egr1 expression (*n* ≥ 13 per genotype, ***P = 0.0004). **(i)** Immunoblotting detection and quantification of A20/TNFAIP3 (*n* ≥ 8 per genotype, **P = 0.0011). GAPDH was used as a loading control. All statistics by unpaired Student’s *t* test

Since our RNAseq data underscored alterations in MAPK cascades, we next evaluated the activation of the p38 group of MAPKs (p38^MAPKs^) and the c-Jun N-terminal kinases (JNKs) as central elements. The phosphorylation status of multiple members of the p38^MAPK^ pathway, including phosphorylated MSK1, p38^MAPK^, MKK3/MKK6, activating transcription factor 2 (ATF-2/7) and HSP27, was analyzed. Interestingly, we found a profound increase in p-p38 and p-ATF-2/7 levels in the A53T Tg mice compared with their WT littermates (Fig. 4a, b). The levels of p-MSK1 and p-MKK3/6 s were not altered whereas p-HSP27 was undetectable in both WT and A53T Tg mice (Additional file 1: Fig. S5c–e). Finally, detection of phospho-JNK2 showed that the JNK pathway was not activated (Additional file 1: Fig. S5f).

The p38^MAPK^-dependent stimulation of ATF2/7 transcription is a well-established signaling cascade leading to the transcription and release of TNFα and IL-1β as mediators of immune responses in neurons, microglia and astrocytes [50]. Given the western blotting findings, we next performed a series of immunofluorescence experiments to understand which glial cell type mostly exhibits activation of these pathways. We found that the phosphorylation of p38 was induced predominately in the nucleus of Iba1^+^ microglia in the A53T striatum whereas p-ATF2/7, which is activated downstream of p38^MAPK^, was localized in the nucleus of both microglial and non-microglial cells but not astrocytes (Fig. 4c, e and Additional file 1: Fig. S6a–d). Both phosphorylated proteins showed very low expression in GFAP^+^ astrocytic cells (Fig. 4c, e and Additional file 1: Fig. S6a–d). Since p38 is a key regulator of microglia-dependent proinflammatory cytokine up-regulation [51], we quantified p-p38 immunofluorescence in the nucleus of individual microglial cells in sections across the striatum. Supporting our initial observation, single-cell fluorescence intensity showed increased p-p38 levels in the nucleus of microglial cells in vivo (Fig. 4d). Collectively, our results so far suggest that the presence of high levels of α-synuclein conformers can trigger p38^MAPK^-dependent microglial activation, which stimulates the ATF2/7-mediated transcription possibly to mediate the production of immune mediators.

### A53T astrocytes exhibit an unconstrained activation of the NF-κB pathway

Activation of the p38^MAPK^-ATF2/7 axis leads to the stimulation of several distinct molecular cascades including the NF-κB pathway, which is a central mediator of pro-inflammatory gene induction and a hallmark of chronic inflammatory diseases [52]. NF-κB pathway dysregulation has been implicated in neurodegenerative disorders. Of note, nuclear translocation of NF-κB is observed in post-mortem brain tissues of PD patients and in mouse models of PD in which overactivation of this pathway is implicated in the promotion of survival, activation and differentiation of innate immune cells, thereby prolonging inflammation. Given that the proinflammatory NF-κB responses can be acutely triggered by TNFα and IL-1β cytokines, we examined whether this pathway is dysregulated in A53T Tg mice in which these cytokines are elevated. Interestingly, we found a robust increase in phospho-NF-κB by western blotting (Fig. 4f). At the transcriptional level, NF-κB is specifically repressed by Early Growth Response 1 (Egr-1), a member of the early growth response family of transcription factors, through a physical interaction between the NF-κB DNA-binding domain and the Egr-1 zinc finger domain [53]. At the protein level, termination of NF-κB signaling is tightly regulated by A20/TNFAIP3 which utilizes both ubiquitin ligase and deubiquitinase activities to disrupt the protein scaffold that leads to NF-κB translocation to the nucleus [54]. We found significantly reduced expression of both *Egr-1* and A20/TNFAIP3, suggesting a limited ability for NF-κB repression in A53T Tg mice (Fig. 4g, i). Further investigation of the downstream of the NF-κB pathway did not reveal any differences in IκΒα activation as shown by the similar levels of p-IκΒα or total IκΒα (Additional file 1: Fig. S5g).

Recent evidence suggested that PFFs can produce transcriptional changes in human midbrain astrocytes that depend on NF-κB stimulation [15]. Using immunostaining of striatal sections with a specific p65 antibody, we observed increased levels of phospho-p65 in A53T astrocytes but not microglial cells, suggesting that the NF-κB pathway is preferably upregulated in A53T astrocytes in vivo (Fig. 4h and Additional file 1: Fig. S6e, f).

Considering that the activated microglia can trigger astrocyte reactivity, our results indicated that, in A53T Tg mice, p-p38-mediated microglial activation can induce a strong NF-κB activity in astrocytes.

### L- and T-type Ca^2+^ channels are differentially expressed in A53T reactive astrocytes

**Fig. 5:**
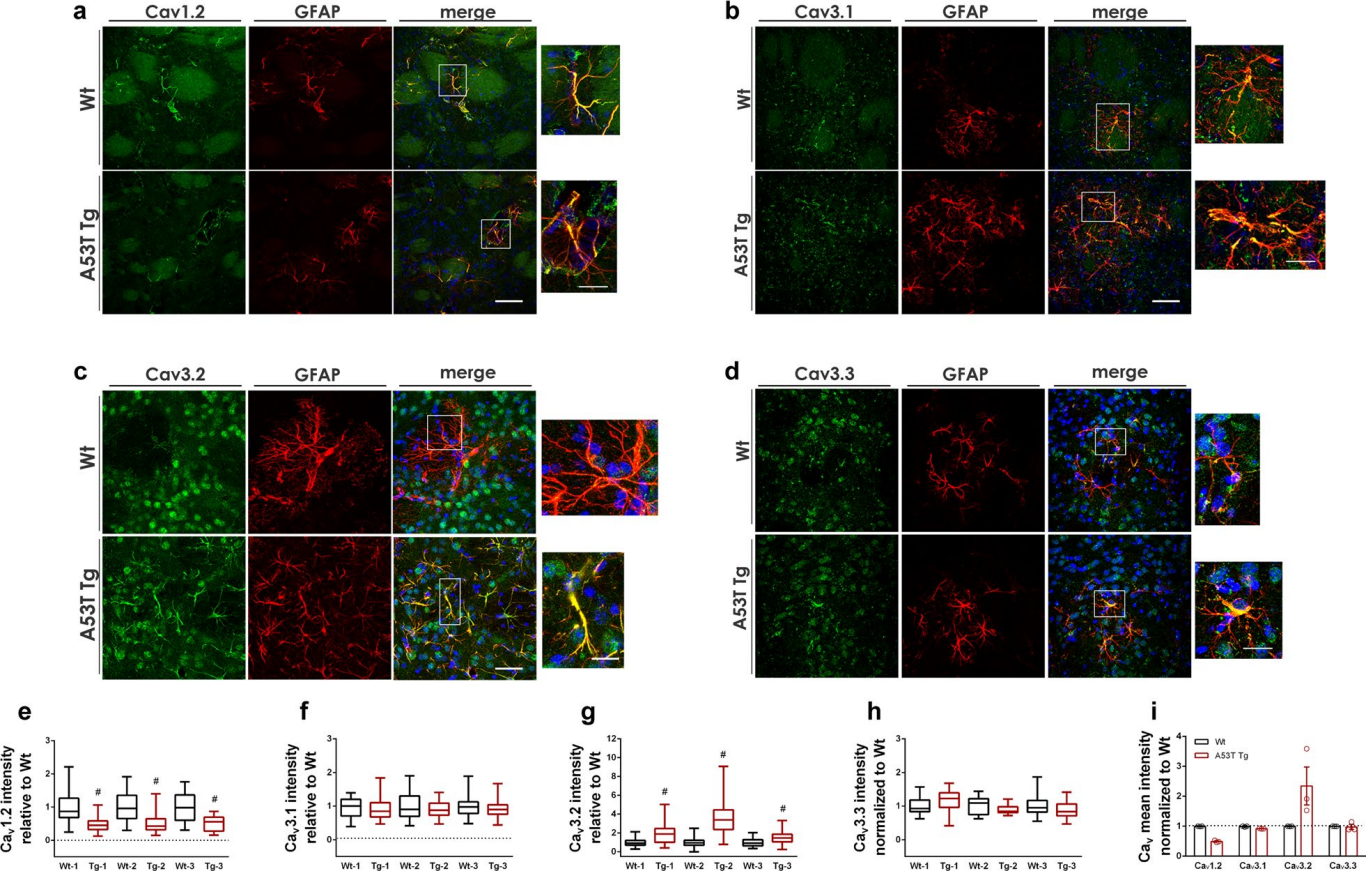
Ca_v_1.2 and Ca_v_3.2 are differentially expressed in A53T striatal astrocytes. **a–d** Representative confocal images of striatal sections co-stained with antibodies against Ca_v_1.2 (**a**), Ca_v_3.1 (**b**), Ca_v_3.2 (**c**), and Ca_v_3.3 (**d**) and the astrocyte marker, GFAP. DAPI (blue) was used for nuclei staining. Images on the right represent magnification of the boxed area. Scale bar: 50 μm (10 μm for the magnification images). **e–h** Quantification of mean fluorescence intensity of Ca_v_ VGCCs in individual GFAP^+^ astrocytes following 3D reconstruction in three independent pairs (1, 2, 3) of WT and A53T Tg mice; (**e**) for Ca_v_1.2, ^#^*P* < 0.0001, *n* ≥ 68 astrocytes for each genotype, **f** for Ca_v_3.1, *P* = 0.3298, 0.1878, 0.0950 for pairs 1, 2, 3 respectively and *n* ≥ 44 astrocytes for each genotype, **g** for Ca_v_3.2, ^#^*P* < 0.0001, *n* ≥ 129 astrocytes, and **h** for Ca_v_3.3, *P* = 0.2434, 0.2479, 0.0955, *n* ≥ 16 for each genotype. **i** Pre-averaged values of Ca_v_ mean fluorescence intensity relative to WT for 3 WT and A53T animal pairs. Data are presented as means ± SEM. All statistics by unpaired Student’s* t* test

Our RNAseq analysis in A53T mice indicated alterations in Ca^2+^ signaling and up-regulation of MYLK, a kinase required for increased astrocytic Ca^2+^ wave propagation and astrocyte motility. Ca^2+^ signaling is fundamental for astrocyte functioning since intracellular Ca^2+^ transients and intercellular Ca^2+^ waves allow intercellular communication between astrocytes or between astrocytes and neurons. We found that astrocytes exhibit strong NF-κΒ activity which can be regulated by Ca^2+^ signaling. Calcium can activate DREAM, a Ca^2+^ sensor protein that acts as a transcriptional repressor of A20/TNFAIP3, which negatively regulates the NF-κB activity to resolve inflammation. In addition, NF-κB-produced cytokines are released in a Ca^2+^-dependent manner. To investigate changes in Ca^2+^ signaling in A53T astrocytes, we first performed a series of immunolabeling experiments using antibodies specific for all the Ca_v_ subunits to understand which VGCCs are normally expressed in striatal GFAP^+^ astrocytes. We found that only the L-type Ca_v_1.2^+^ and the T-type (Ca_v_3.1^+^, Ca_v_3.2^+^ or Ca_v_3.3^+^) VGCCs were expressed in the GFAP^+^ astrocytes in mouse striatum (Fig. 5a–d). Co-localization experiments using the neuronal marker TUJ1 showed that these VGCCs were also expressed in neurons, as expected (Additional file 1: Fig. S7a–d). The Ca_v_2.1, Ca_v_2.2 and Ca_v_1.3 did not co-localize with GFAP, suggesting their expression predominately in neurons (Additional file 1: Fig. S8a–c).

Since the L-type Ca_v_1.2^+^ and the T-type VGCCs are expressed in both astrocytes and neurons, to visualize changes in the expression of astrocytic VGCCs at the single-cell level, we measured the Ca_v_ immunofluorescence in individual 3D-reconstructed GFAP-labeled astrocytes in five sections spanning the striatum using the IMARIS software. Such detailed comparison of Ca_v_ immunofluorescence in single astrocytes between three pairs of WT and A53T Tg mice unveiled a significant reduction of Ca_v_1.2^+^ and a significant induction of Ca_v_3.2^+^ VGCCs in A53T astrocytes, whereas Ca_v_3.1^+^ and Ca_v_3.3^+^ VGCCs remained unaltered (Fig. 5e–i).

Taken together, our results indicate that in A53T Tg mice, the astrocytic Ca_v_1.2 and Ca_v_3.2 VGCCs are differentially regulated, thereby disturbing Ca^2+^ homeostasis in the striatum.

### T-type Ca_v_3.2 Ca^2+^ channels are up-regulated by cytokines in a NF-κB-dependent manner

Our data from the striatum of A53T Tg mice support an induction of astrocytic T-type Ca_v_3.2 VGCCs in the presence of α-synuclein oligomers. In the context of neuroinflammation, this finding was of particular interest since the T-type Ca_v_3.2 VGCCs are upregulated by a variety of inflammatory mediators and have been implicated in the modulation of inflammatory and chronic pain in primary sensory neurons [55, 56]. Ca_v_3.2 channel dysregulation associated with either gain-of-function, such as in the cases of epilepsy and neuropathic pain, or loss-of-function mechanisms, such as in autism spectrum disorders, schizophrenia, and ALS, could be a contributor to the pathogenesis of various CNS diseases. However, the role of astrocytic Ca_v_3.2 VGCCs in neuroinflammation is currently unknown.

To investigate the molecular mechanisms involved in Ca_v_3.2 induction in astrocytes, we established a mouse primary culture of astrocytes with high purity that mimic the quiescent astrocytic phenotype. Since FBS-cultured primary astrocytes are enriched in markers for reactive gliosis [57], we used a serum-free culture medium based on nutrients and growth factors to achieve sufficient astrocyte maturation and functionality [26]. The purity of this culture was confirmed by the total absence of Iba1^+^ microglial cells as compared with a mixed glial culture grown in FBS-based medium (Additional file 1: Fig. S9a). Further characterization showed that almost all primary astrocytes expressed the pan-astrocytic marker ALDHL1 (Aldehyde Dehydrogenase 1 Family Member L1), whereas 40% of cells were positive for GFAP expression (Additional file 1: Fig. S9b, c). To assess the functional properties of cultured astrocytes, we analyzed their ability to respond to exogenous stimuli such as LPS, KCl and ATP. LPS is known to activate astrocytes preferentially towards the A1 activation phenotype [58]. Treatment with 0.5 μg/ml LPS for 24 h resulted in the upregulation of the A1-type marker, *Iigp1*, but did not affect the level of the A2-type marker, *S100a10* (Additional file 1: Fig. S9d, e), consistent with previous studies [14, 58]. Fura-2AM-based live Ca^2+^ imaging showed that the cultured astrocytes were able to respond to both KCl and ATP excitation by producing Ca^2+^ fluctuations, which were propagated to a network of neighboring cells, suggesting the generation of a Ca^2+^ wave (Additional file 1: Fig. S9f, g). The KCl- and ATP-induced Ca^2+^ transients showed similar [Ca^2+^]i peak but differed in the response time and duration (Additional file 1: Fig. S9h, i), as expected.

We next examined the responses of these primary astrocytes to cytokine-induced signaling. For this, we treated astrocytes with TNFα (20 ng/ml), IFNγ (1 ng/ml) or IL-1β (5 ng/ml) for 24 h and evaluated the transcriptional induction of *Iigp1* and *S100a10* genes and their functional effects on the p38/NF-κB pathway and Ca^2+^ influx. Treatments were performed in the absence of growth factors since both EGF and FGF2 are known to activate several signaling pathways including the p38^MAPK^ pathway, interfere with Ca^2+^ signaling [59] and suppress astrocytic activation in culture [14]. In our hands, growth factor deprivation induced both the A1 and A2 type markers (Additional file 1: Fig. S9j, k) but did not activate the NF-κB pathway (Additional file 1: Fig. S9l). Our qPCR analysis indicated that TNFα and IFNγ promoted the activation of astrocytes towards the A1 state, but not the A2 state, with IFNγ being the strongest inducer, while IL-1β did not affect either A1 or A2 activation state (Additional file 1: Fig. S9m, n). To address the ability of cytokines to trigger the p38/NF-κB pathway, the phosphorylation of p38 and the nuclear translocation of NF-κB were examined. Both TNFα and IL-1β, but not IFNγ, strongly induced the translocation of NF-κB to the nucleus of astrocytes, suggesting a potent activation of the NF-κΒ pathway (Additional file 1: Fig. S9o). None of the cytokines triggered the p38 signaling as suggested by the lack of p38 phosphorylation upon cytokine treatment (Additional file 1: Fig. S9o).

**Fig. 6:**
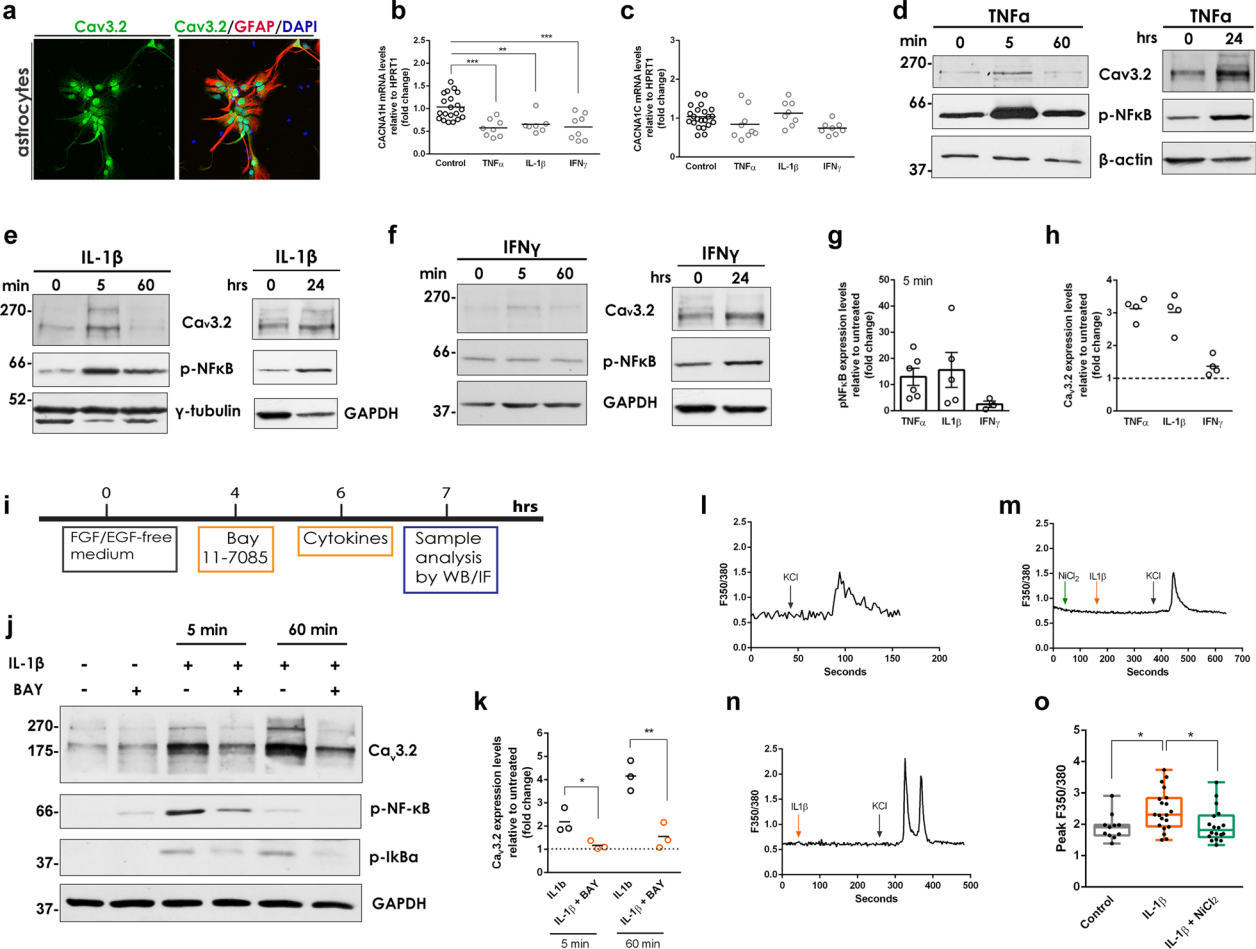
T-type Ca_v_3.2 Ca^2+^ channels are up-regulated by cytokines in a NF-κB-dependent manner. **a** Representative confocal images of primary astrocytes co-stained with specific antibodies for Ca_v_3.2 and GFAP. **b** qPCR measurement of CACNA1H mRNA levels following treatment with TNFα, ***P = 0.0003, IL-1β, **P = 0.0042 and IFNγ, ***P = 0.0005 (*n* ≥ 8 per condition). **c** CACNA1C mRNA levels assessed as above (TNFα; *P* = 0.2536, IL-1β; *P* = 0.8093, IFNγ; *P* = 0.0537) (*n* ≥ 8 per condition). Statistics in (**b, c**) by one-way ANOVA followed by Dunnett’s multiple comparisons test. **d–f** Representative western blots showing Ca_v_3.2 and p-NF-κB levels following treatment with TNFα, IL-1β or IFNγ for the indicated time points. **g, h** Fold induction of pNFκB (**g**, *n* ≥ 3 independent replicates per treatment) and Ca_v_3.2 (**h**, *n* = 4 independent replicates per treatment) expression levels relative to untreated controls following cytokine treatment for 5 min. **i** Experimental timeline explaining the treatment of primary astrocytes with IL-1β in the absence or presence of the NF-κB pathway inhibitor, BAY 11-7083. **j** Representative western blot showing the levels of Ca_v_3.2, p-NF-κB and p-IκB in the absence or presence of IL-1β and BAY 11-7083. **k** Fold induction of Ca_v_3.2 expression levels following treatment with IL-1β in the absence or presence of BAY 11-7083 for 5 min (*P = 0.0354) and 60 min (**P = 0.0060). Values are estimated relative to untreated from three independent biological replicates. Statistics by unpaired Student’s* t* test. **l–n** Representative plots from live calcium imaging of primary astrocytes upon exposure to IL-1β with or without pre-treatment with the Ca_v_3.2 inhibitor, NiCl_2_. **o** Quantification of F350/380 peak ratios from primary astrocytes after addition of IL-1β alone (*P = 0.0431 vs control) or IL-1β and NiCl_2_ (*P = 0.0364 vs IL-1β alone). Statistics were performed by one-way ANOVA followed by Tukey’s multiple comparisons test (*n* ≥ 12 images per condition in triplicates)

Following their molecular and functional characterization, we used quiescent primary astrocytes as a cellular model to address the molecular mechanism underlying Ca_v_3.2 induction, which was readily expressed in this cellular system (Fig. 6a). We initially assessed whether cytokines could interfere with the transcriptional regulation of Ca_v_3.2 channels. Cytokines were applied in primary astrocytes as above for 24 h, cells were collected, and RNA was extracted. qPCR analysis revealed that all cytokines have the potential to downregulate CACNA1H mRNA by approximately 50%, suggesting that astrocytic Ca_v_3.2 transcription depends on the presence of cytokines albeit in a negative manner (Fig. 6b). In contrast, the mRNA levels of Ca_v_1.2 remained stable in the presence of cytokines, indicating that the cytokine-dependent reduction in Ca_v_3.2 transcription is a specific effect (Fig. 6c).

Our initial results suggest that the up-regulation of Ca_v_3.2 protein observed in vivo could not be attributed to the cytokine-induced changes directly at the transcriptional level. To further exclude this possibility, we measured in A53T Tg mice the mRNA levels of Ca_v_3.2 transcriptional regulation elements. Ca_v_3.2 expression is normally regulated by the transcription factor Egr1 that binds to the *Cacna1h* promoter. Upon binding, EGR1 can induce its own repressor, NGFI-A-binding protein 2 (NAB2), which controls the EGR1-mediated Ca_v_3.2 expression. EGR1 action is antagonized by the transcriptional repressor RE1 Silencing Transcription Factor (REST) which counteracts the stimulatory effect of EGR1 on *Cacna1h* promoter [60]. We found that in A53T mice, *Egr1* expression was significantly reduced (Fig. 4g) whereas *Nab2* and *Rest* mRNA expression remained unchanged compared to the WT mice (Additional file 1: Fig. S10), further supporting that Ca_v_3.2 induction is not due to transcriptional dysregulation of the *Cacna1h* gene.

Ca_v_3.2 level is also functionally regulated by inflammatory mediators, such as IL-1β, that destabilize the ubiquitination of Ca_v_3.2 thereby blocking its proteasomal degradation and promoting its accumulation in the plasma membrane [61, 62]. To address the potential effects of cytokines at such protein level, TNFα, IL-1β, and IFNγ were applied to quiescent astrocytes for 5 min, 60 min or 24 h. Western blotting analysis revealed a reproducible three-fold increase in Ca_v_3.2 levels after the 5-min treatment with TNFα and IL-1β, but not IFNγ (Fig. 6d–f). Interestingly, the increase in Ca_v_3.2 levels correlated with the induction of the NF-κB pathway (Fig. 6d–h). In support of this, IFNγ, which did not trigger NF-κB, did not modulate Ca_v_3.2 expression (Fig. 6f–h). After 24-h treatment, all three cytokines increased p-NF-κB and Ca_v_3.2 albeit at a lower extent compared to the 5-min treatment (Fig. 6d–f).

In sum, our results indicated that the stimulation of the NF-κB pathway by TNFα or IL-1β induces the upregulation of T-type Ca_v_3.2 Ca^2+^ channels in primary astrocytes, a mechanism that could explain our in vivo data. To confirm this mechanism, we used the NF-κB pathway inhibitor, BAY 11-7085, which acts by irreversibly blocking the cytokine-induced IκBα phosphorylation in this pathway (Fig. 6i). BAY 11-7085 was administered in primary astrocytes in the presence or absence of IL-1β for 5 or 60 min and the effects of the compound treatments on Ca_v_3.2 expression levels were assessed by western blotting (Fig. 6j). Our results showed that BAY 11-7085 potently inhibited IκBα and NF-κB phosphorylation. Interestingly, co-administration of BAY 11-7085 and IL-1β totally reversed the IL-1β-induced Ca_v_3.2 increase in astrocytes, suggesting that IL-1β can induce Ca_v_3.2 VGCCs in a NF-κB-dependent manner (Fig. 6k).

Finally, we examined whether the cytokine-induced Ca_v_3.2 VGCCs are functionally active to potentiate elevations in astrocytic Ca^2+^ influx. To test this, we measured Ca^2+^ influx in living primary astrocytes following IL-1β addition in the presence or absence of the selective Ca_v_3.2 channel blocker, NiCl_2_. As before, KCl addition was used to fully depolarize all VGCCs (Fig. 6l–n). We found that IL-1β stimulated Ca^2+^ influx, which was greatly inhibited by NiCl_2,_ suggesting that Ca_v_3.2 channels can be functionally induced by IL-1β (Fig. 6o).

To confirm the importance of our findings in vivo, we analyzed low-magnification images of striatum following co-labelling with Ca_v_3.2 and Tuj1 antibodies. Such analysis revealed that the majority of Ca_v_3.2 immunofluorescence was predominately localized in astrocytes of the A53T Tg mice in contrast to WT mice in which Ca_v_3.2 was mostly localized in neurons (Additional file 1: Fig. S11a), thus indicating that Ca_v_3.2 induction was not locally restricted but was a generalized biological process in A53T astrocytes. To investigate whether the pathway connecting α-synuclein-associated neuroinflammation with Ca_v_3.2 upregulation in astrocytes was specific for the striatum, we analyzed three other brain areas, the cortex, hippocampus and SNpc, for the presence of α-synuclein oligomers and endogenous IgG antibodies. We found that all three areas that express the transgene at high levels, contained SDS-soluble oligomeric α-synuclein conformers, the levels of which were variable among the A53T Tg mice tested (Additional file 1: Fig. S11b). As in the striatum, α-synuclein oligomers were absent from WT tissues. Further, we found increased levels of endogenous IgG antibodies in the cortex, hippocampus and SNpc of A53T Tg mice, indicating an active immune response (Additional file 1: Fig. S11c). In accordance with our previous findings, further analysis of the cortex, an area that is synaptically connected to the striatum and provides the majority of α-synuclein through the glutamatergic afferents [23], showed increased levels of TNFα, significant astrocytosis and activation of the p38 pathway in A53T Tg mice compared with their WT littermates (Additional file 1: Fig. S11d–f). Finally, we repeated the Ca_v_3.2 immunostaining in the cortex using antibodies against Ca_v_3.2 and the astrocytic marker GFAP, and clearly showed that Ca_v_3.2 expression was induced in the astrocytes of A53T Tg mice in accordance with our findings in the striatum (Additional file 1: Fig. S11g). To put our work in the context of PD pathology, similar immunostaining experiments in the SNpc in midbrain sections revealed that Ca_v_3.2 levels were robustly elevated not only in striatal but also in nigral astrocytes, further supporting a generalized molecular link between α-synuclein-induced neuroinflammation and astrocytic Ca_v_3.2 upregulation (Additional file 1: Fig. S11h).

Our in vivo data support a role of α-synuclein oligomers in NF-κB activation, leading to Ca_v_3.2 induction in astrocytes. To test this hypothesis in vitro, we used synthetic PFFs that were previously shown to activate primary microglia as documented by increased Iba1 expression and elevated transcription and secretion of cytokines [11, 63]. Co-cultures of primary microglia and quiescent astrocytes were treated with 300 ng/ml human PFFs for 3 h to allow efficient internalization of α-synuclein as shown by immunostaining experiments following PFF application (Additional file 1: Fig. S12a). However, internalization of PFFs did not activate the p38^MAPK^ or NF-κB pathways in either microglia or astrocytes (Additional file 1: Fig. S12b, c). At the same time, we detected no changes in Ca_v_3.2 levels in either glial cell type (Additional file 1: Fig. S12d). We verified our results using immunoblotting following PFF treatment. Again, PFF addition to mixed glial cells resulted in α-synuclein uptake and oligomer formation but did not activate the p38^MAPK^ or NF-κB pathway and did not cause Ca_v_3.2 accumulation (Additional file 1: Fig. S12e).

Collectively, these data suggest that the activation of the NF-κB pathway can result in the accumulation of functional Ca_v_3.2^+^ VGCCs in astrocytic membranes, providing a rationale for our in vivo data in A53T Tg mice in which high levels of TNFα and IL-1β are combined with sustained NF-κB activity and Ca_v_3.2 up-regulation in reactive astrocytes. Exogenously added synthetic PFFs could not activate p38 or NF-κB pathways and did not affect Ca_v_3.2 expression.

### Induction of Ca_v_3.2 T-type channels induces alterations of astrocyte secretome, promoting the release of the neuroprotective protein IGFBPL1

**Fig. 7:**
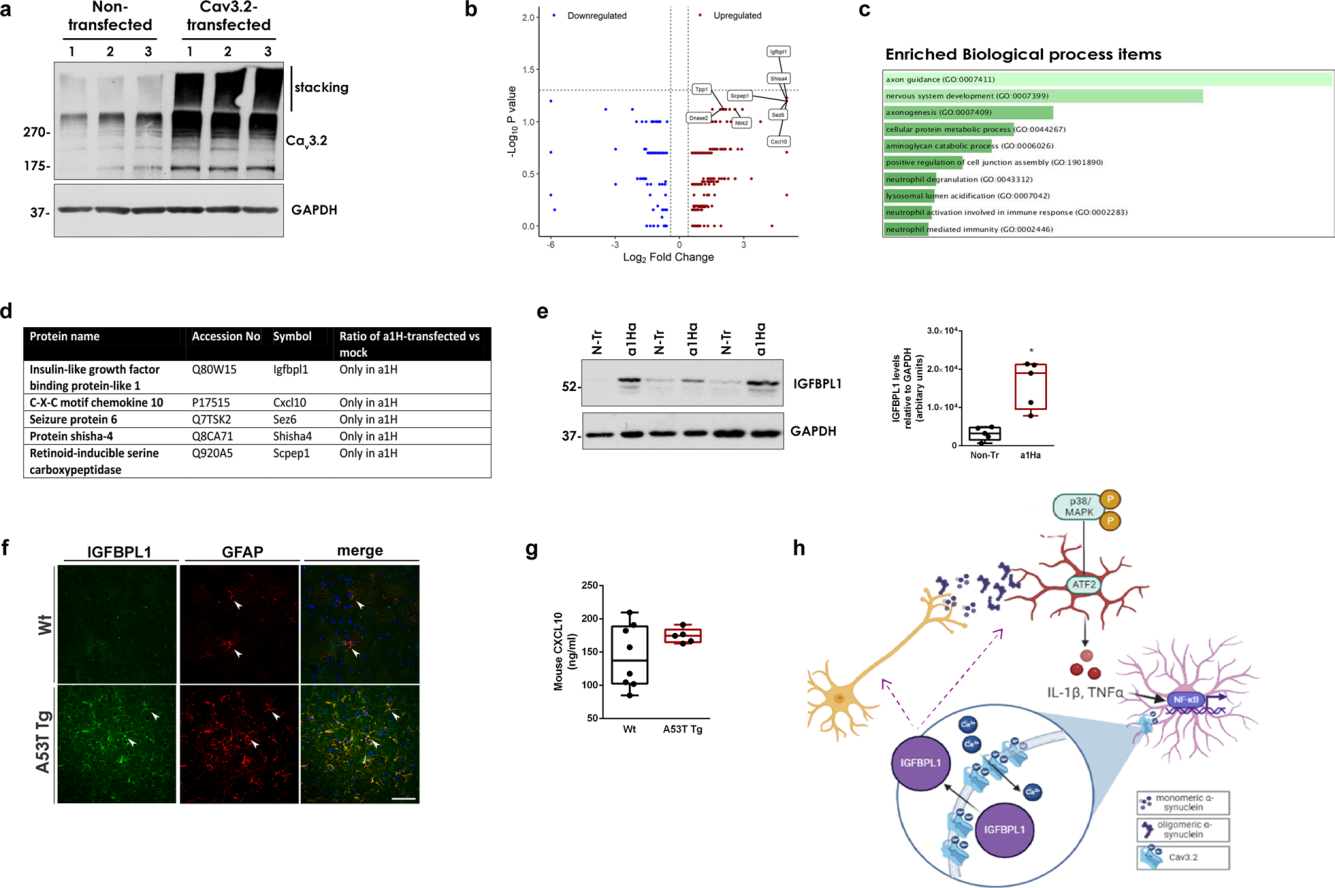
The induction of Ca_v_3.2 VGCCs alters astrocyte secretome promoting the release of the neuroprotective protein IGFBPL1. **a** Representative immunoblot of mock- and a1H-transfected primary quiescent astrocytes using an antibody against Ca_v_3.2. **b** Volcano plot showing the up- and down-regulated proteins identified by SignalP as secreted proteins identified by the LC–MS/MS analysis from the CM of mock- and a1H-transfected astrocytes (*n* = 3 independent replicates). **c** List of the top ten pathways as classified by Enrich-r. **d** List of the top five secreted proteins identified in the CM of astrocytes after a1H overexpression. **e** Immunoblot from mock and a1H-transfected astrocytes using an antibody against IGFBPL1 and densitometric quantification (*n* = 5 per condition, *P = 0.0203). **f** Representative confocal images of striatal sections co-stained with antibodies against IGFBPL1 and GFAP. DAPI (blue) was used to stain nuclei. Scale bar: 50 μm. **g** Quantification of mouse CXCL-10 levels in striatal homogenates (*n* ≥ 5, *P* = 0.1864). **h** Proposed mechanism through which α-synuclein oligomers activate microglia and astrocytes and induce Ca_v_3.2 channels that mediate IGFBPL1 secretion. GAPDH was used as a loading control. In **e**, **g**, statistics were performed by Unpaired Student’s *t* test

Astrocytes are secretory cells and utilize Ca^2+^ influx to facilitate the release of a variety of signaling molecules that enable their communication with neurons and other glial cells in the CNS. These molecules are released via several secretory pathways including Ca^2+^-regulated exocytosis and can be protective or neurotoxic depending on the disease condition in the brain [64]. Previous studies have reported that the Ca_v_3.2 channels can mediate exocytosis events through the Ca_v_3.2-SNARE interaction [65]. To investigate which proteins are released by astrocytes through Ca_v_3.2-mediated exocytosis, we transiently transfected primary quiescent astrocytes with a1Ha-pcDNA3 plasmid containing the *Cacna1h* gene and collected the CM from a1Ha- and mock-transfected cells 48 h after transfection. Immunoblotting analysis confirmed efficient overexpression of Ca_v_3.2 in cultured astrocytes following transfection (Fig. 7a). The CM of three individual experiments (*n* = 3 for each a1Ha- and mock-transfected cells, respectively) were concentrated and analyzed by LC–MS/MS. Results were processed by Sequest algorithm to generate a list of 2314 detected unique proteins (Additional File 3). From this list, SignalP prediction analysis identified 143 up-regulated and 92 down-regulated secreted proteins in a1H-transfected astrocytes versus controls (Fig. 7b and Additional File 4). Enrichr-based classification indicated that the up-regulated secreted proteins were mostly involved in axon guidance and regeneration, regulation of cell junction assembly and neutrophil activation (Fig. 7c). To identify specific proteins that are secreted through Ca_v_3.2, we focused on the top five proteins that were present in the a1Ha-transfected CM and totally absent from the mock-transfected CM (Fig. 7d). From these proteins, the first two were of particular interest; IGFBPL1 has been implicated in IGF-dependent axon growth and could be neuroprotective against inflammation [66], and C-X-C motif chemokine 10 (CXCL10), a small IFNγ-induced neutrophil chemoattractant, could exacerbate inflammation and trigger adaptive immunity in the CNS [67]. IGFBPL1, an IGF-1 binding protein, is emerging as a critical co-factor of IGF to facilitate the activation of IGF-triggered axon growth cascades and regulate microglial homeostasis to resolve inflammation [68, 69]. The induction of IGFBPL1 was verified in the homogenates of mock- and a1Ha-transfected astrocytes in which IGFBPL1 was up-regulated almost five times upon *Cacna1h* transfection. (Fig. 7e). Immunofluorescence assessment of IGFBPL1 expression showed a robust increase of IGFBPL1 in A53T GFAP^+^ astrocytes (Fig. 7f), suggesting that the astrocytic Ca_v_3.2 VGCCs could mediate the release of IGFBPL1 to induce IGF signaling in vivo. Finally, we tested whether the soluble chemokine CXCL10 is increased in A53T Tg mice. Previous work has shown that under conditions of inflammation, the expression and secretion of CXCL10 by astrocytes is specifically triggered by IFNγ in a NF-κB-dependent manner [70]. CXCL10 acts by binding to the CXR3 receptor to mediate neutrophil recruitment [71] or promote astrocyte proliferation [63]. We measured CXCL10 using a mouse specific ELISA kit and found increased levels of CXCL10 in A53T homogenates although this increase did not reach statistical significance (Fig. 7g).

Collectively, our findings suggest that the accumulation of α-synuclein oligomers activates p38^MAPK^-ATF2/7 in microglia, which strongly induces the NF-κB pathway in astrocytes. The NF-κΒ induction can drive the up-regulation of Ca_v_3.2 VGCCs in astrocytes, mediating the release of IGFBPL1 as a protective compensatory mechanism against α-synuclein-associated neuroinflammation (Fig. 7h).

### Human PD brains are also characterized by α-synuclein oligomers, C3 complement activation and increased Ca_v_3.2 mRNA levels

**Fig. 8:**
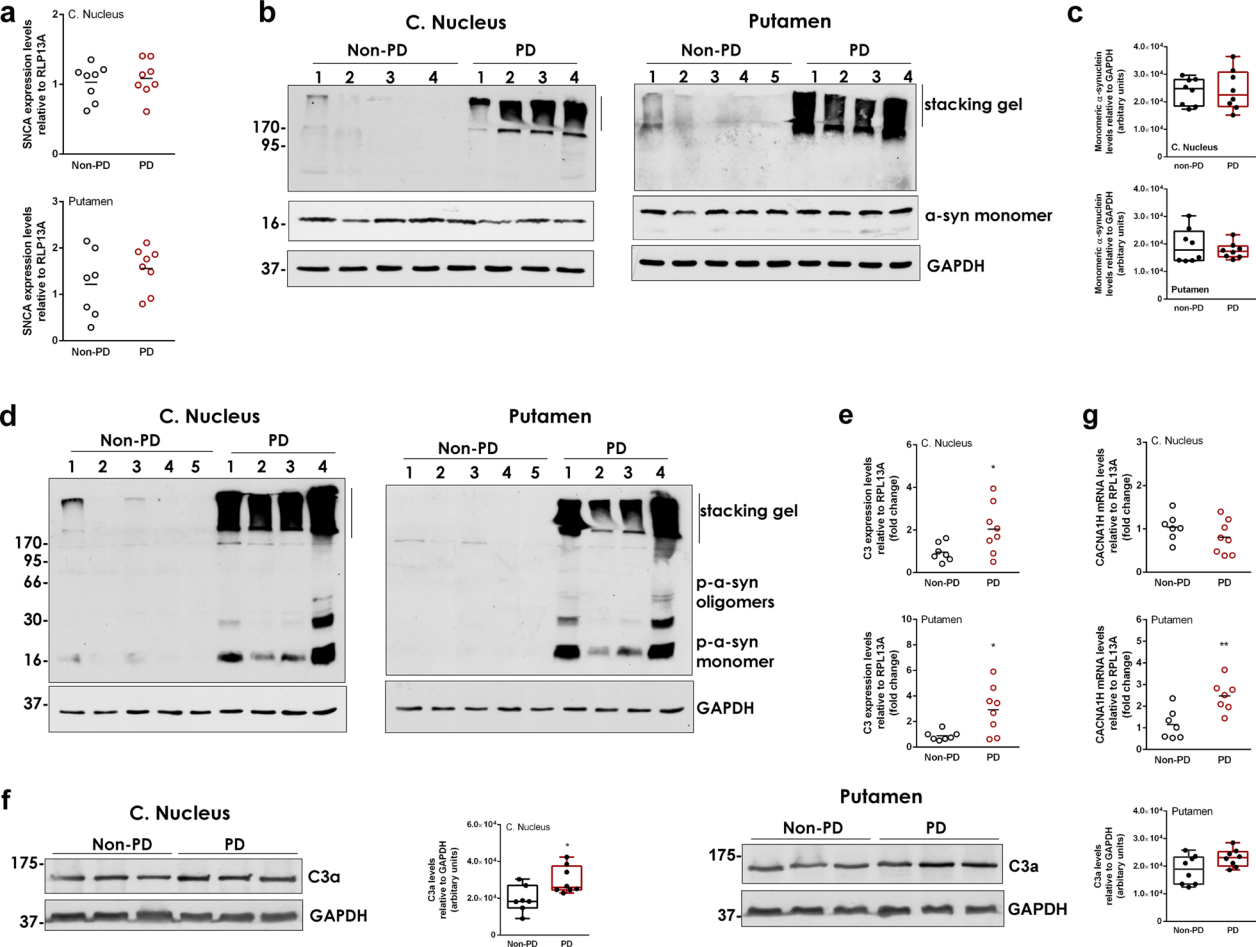
Human PD brain is characterized by α-synuclein oligomers, C3 complement activation and altered Ca_v_3.2 mRNA levels. **a** Comparison of the *SNCA* mRNA levels in the caudate nucleus (*n* = 8 per group, *P* = 0.6926) and putamen tissue (*n* = 7 Non-PD and *n* = 8 PD, *P* = 0.2917) of PD vs non-PD subjects. **b** Representative western blots depicting oligomeric and monomeric α-synuclein in the two brain areas. **c** Densitometric quantifications of monomeric α-synuclein in the caudate nucleus, *P* = 0.8613 and putamen, *P* = 0.4783 (*n* = 8 per group per brain area). **d** Representative western blots depicting phosphorylated oligomeric and monomeric α-synuclein in the two brain areas. **e** qPCR assessment of C3 mRNA in caudate nucleus, *P = 0.0373 and putamen, *P = 0.0140 (*n* = 8 per group, per brain area). **f** Representative western blots showing the levels of C3a in caudate nucleus (*n* = 7 non-PD and *n* = 8 PD, *P = 0.0168) and putamen (*n* = 8 per group, *P = 0.0651). **g** qPCR assessment of CACNA1H mRNA in caudate nucleus (*n* = 7 Non-PD and *n* = 8 PD, *P* = 0.2184) and putamen (*n* = 7 per group, **P = 0.0044). In all western blots, GAPDH was used as a loading control. All statistics were performed by unpaired Student’s *t* test

The caudal putamen, which is mainly involved in movement coordination and habitual behavior, is the site of greatest dopaminergic loss in early disease, possibly underlying the impairment of automatic and habitual performance, one of the most common symptoms of PD patients often antedating diagnosis for several years [20]. To address the human relevance of our findings, we analyzed post-mortem brain tissues of the caudate nucleus and the putamen, two brain areas comprising human striatum, from 8 PD patients and 8 individuals with no neuropathological evidence of synucleinopathy (non-PD samples) (Additional file 1: Table S1). Although the α-synuclein mRNA expression was similar in both areas between non-PD and PD groups (Fig. 8a), CHAPS homogenization and immunoblotting analysis revealed high-order α-synuclein multimers of limited SDS solubility as indicated by their partial retention in the stacking gel (Fig. 8b). These aggregated forms of α-synuclein were found only in PD tissues and were completely absent from the tissues of non-PD individuals, indicating a direct association with the disease. Comparison of the levels of monomeric α-synuclein did not show any differences in PD vs non-PD samples irrespective of the area (Fig. 8b, c). Importantly, our analysis revealed the presence of intensively phosphorylated α-synuclein oligomers exclusively in the PD samples (Fig. 8d). We next assessed complement activation and found increased *C3* mRNA in the PD samples in both caudate nucleus and putamen (Fig. 8e). In the case of caudate nucleus, the C3a protein level was also significantly elevated (Fig. 8f). We did not measure tissue cytokines since gliosis was reported as a microscopic neuropathological finding at autopsy possibly due to aging for the majority of human samples. Interestingly, the *CACNA1H* gene, which encodes for Ca_v_3.2 channels, was found significantly up-regulated in the putamen, but not caudate nucleus, of PD brains (Fig. 8g).

We conclude that the human PD brains are characterized by the presence of aberrant hyperphosphorylated oligomeric α-synuclein, increased complement C3 levels and altered Ca_v_3.2 expression, suggesting that our findings in A53T Tg mice can reflect, at least in part, the pathological features of human disease.

## Discussion

Extracellular aberrant α-synuclein is considered a major trigger of neuroinflammation since it can be phagocytosed by microglia, inducing their prolonged activation into a pro-inflammatory phenotype. Despite this established notion, the exact α-synuclein conformers that cause microglial activation in vivo are not known. Our work elaborates on the causative link between neuron-produced α-synuclein oligomers and sustained neuroinflammation in vivo by mapping the activation of specific signaling pathways in microglia and astrocytes. Using an α-synuclein over-expressing mouse model and human post-mortem brain material, we have demonstrated here for the first time that the accumulation of pathological oligomeric, but not monomeric, α-synuclein in the brain can trigger inflammatory responses by activating the p38^MAPK^-ATF2 axes in microglia and potentiating NF-κB signaling in astrocytes through the brain-circulating cytokines TNFα and IL-1β. In the presence of α-synuclein conformers (oligomers and monomers), we found persistent biochemical or morphological alterations in glial cells. Microglia are characterized by an Iba1^Low^/CD68^Low^ phenotype, whereas reactive astrocytes show increased TNFα and appear to have fewer branches and lose their distal processes. Microglia activity results in a persistent NF-κB activation that induces astrocytic T-type Ca_v_3.2 Ca^2+^ channels, facilitating the secretion of the neuroprotective protein IGFBPL1 (Fig. 8h). We propose that the Ca_v_3.2-mediated release of IGBPL1 by astrocytes could act as a compensatory mechanism to resolve the α-synuclein-induced inflammation via promoting IGF1 signaling.

### α-Synuclein oligomers but not monomers trigger immune responses in the brain

The generation of aberrant oligomeric and/or fibrillar forms of α-synuclein stems from the intrinsic propensity of α-synuclein to self-aggregate and bind to membranous structures. The process of aggregation is thought to initiate from a local accumulation of monomers, possibly structurally distorted, that gradually oligomerize to generate a repertoire of β-sheet-rich fibrillar and oligomeric multimers of high molecular weight. In this sense, the selective removal of oligomeric α-synuclein in vivo, in the presence of high amounts of monomers, as happens in the case of transgenic mouse models, is technically challenging and remains a limitation of the current study. Such an idea has been put forward by El Agnaf and colleagues who administered oligomer-specific antibodies produced in-house to transgenic mice that over-express WT α-synuclein [72]. Indeed, in this study, a decrease in α-synuclein oligomers/fibrils was associated with a lower level of phosphorylated, oligomeric and total α-synuclein and alleviated both neurodegeneration and neuroinflammation.

Our results from several brain areas including the striatum, cortex, hippocampus and midbrain showed that the generation and accumulation of α-synuclein oligomers in A53T Tg mice were correlated with active immune responses in vivo. Such responses were absent from young A53T Tg mice that express high levels of monomeric but lack oligomeric α-synuclein. To further provide a causative link between α-synuclein oligomers and microglial activation, we used primary mouse microglia and a neuroblastoma cell line inducibly expressing α-synuclein, which readily secrete both oligomeric and monomeric α-synuclein. This in vitro approach verified that the cell-secreted α-synuclein oligomers, but not monomers, have the potential to activate primary quiescent microglia, stimulating the NF-κB pathway and inducing TNFα release. Even though previous studies showed that microglia can recognize and interact with exogenously supplied recombinant monomeric α-synuclein or PFFs [12, 73, 74], we have not observed p38^MAPK^ microglial activation upon PFF treatment despite α-synuclein internalization in vitro [75]. Further, PFFs did not trigger the NF-κB pathway in vitro and therefore, did not induce Ca_v_3.2 VGCCs in astrocytes. This difference could be due to the low amount of PFFs used in our study, since we selected the lowest amount of PFFs in which efficient uptake of α-synuclein could be observed (i.e., 0.3 μg/ml vs. 70 μg/ml used in [75]), in order to approximate the relatively low abundance of cell-secreted oligomers. Previous studies have demonstrated that the cell-produced α-synuclein displays different biochemical properties than recombinant α-synuclein, probably due to alterations in conformation, post-translational modifications, etc., further suggesting that the synthetic α-synuclein could trigger different immune responses. Thus, our data support the idea that cell-produced α-synuclein oligomers and PFFs could possess distinct immunogenic properties and, as such, can activate microglia and astrocytes via different molecular pathways.

### Specific signaling pathways underlie the unconstrained neuroinflammation induced by α-synuclein oligomers

A major finding of our work is the elucidation of a microglia-to-astrocyte crosstalk mechanism through which α-synuclein aggregation prolongs the inflammatory signaling in vivo. We propose that this is mediated by the initial activation of the p38^MAPK^/ATF2 pathway in microglia, resulting in cytokine secretion. Unexpectedly, microglia in the presence of aberrant α-synuclein were characterized by reduced levels of Iba1 and CD68. A loss of Iba1 and other microglial markers has been recently described in neurodegenerative disorders such as Alzheimer’s and Huntington diseases, correlating in some cases with disease severity [39, 76, 77]. Our data show that such a loss in Iba1 and CD68 proteins is also evident in the A53T model of synucleinopathy, suggesting a common theme in microglial responses in neurodegeneration.

We found that the p38 microglial activation is combined with a profound up-regulation of the NF-κB signaling pathway in astrocytes. Such reactivity has been associated with the cytokine-induced transformation of astrocytes to the A1 neurotoxic reactive phenotype as a means to amplify neuroinflammation [14] and α-synuclein propagation [11, 15]. Interventions that reverse the A1 astrocyte reactivity can be neuroprotective in PD models [73]. Due to its fundamental importance in the regulation of immune cell responses, the NF-κB activation status is tightly controlled. In contrast, we found that in A53T Tg mice, increased activity of NF-κB is maintained through downregulation of its transcriptional repressor EGR-1 and the NF-κΒ cascade terminator A20/TNFAIP3. Under these conditions, the activation of NF-κB is not resolved and further contributes to a neurotoxic environment.

### Ca_v_3.2-mediated IGFBPL1 secretion as a means to counterbalance the α-synuclein-induced neuroinflammation

Unexpectedly, we found that the NF-κB activity in astrocytes promotes the up-regulation of astrocytic T-type Ca_v_3.2 Ca^2+^ channels in vitro and in vivo. Despite the documented expression of almost all the VGCC subtypes in cultured astrocytes [78], we found that at least in striatum, only the L-type Ca_v_1.2 and the T-type Ca_v_3 VGCCs are expressed in astrocytes in vivo. From these subtypes, the expression and function of T-type Ca_v_3.2 Ca^2+^ channels are modulated by inflammatory mediators [61, 62]. The functional regulation of Ca_v_3.2 depends on the coordinated action of specific ubiquitinases and de-ubiquitinases that regulate its proteasomal degradation. In neurons, IL-1β-mediated enhancement of the interaction between Ca_v_3.2 and the deubiquitinase USP5 inhibits the proteasome degradation of Ca_v_3.2, thereby promoting its accumulation in the plasma membrane, a mechanism that contributes to the maintenance of chronic pain [61, 62, 79]. Here, using quiescent primary astrocytes, we showed that when TNFα and IL-1β trigger the NF-κB pathway, Ca_v_3.2 can accumulate in the membranes of astrocytes within minutes, indicating a rapid blockage of its proteasomal degradation. This leads to an enhancement of the Ca_v_3.2 VGCC-mediated Ca^2+^ influx in astrocytes as demonstrated using the selective Ca_v_3.2 channel blocker, NiCl_2_. Ca_v_3.2 induction was dependent on NF-κB activity as confirmed by using the specific NF-κB inhibitor, BAY-11-708, that reversed the IL-1β-induced Ca_v_3.2 up-regulation. Respectively, we can assume that Ca_v_3.2 upregulation in A53T astrocytes could be due to the chronic NF-κB activation.

Neuronal Ca_v_3.2 channels are established contributors to the development of seizures and neuropathic and inflammatory pain [80]. However, the role of Ca_v_3.2 channels in astrocytes has not been studied. Our work highlights a novel function of astrocytic Ca_v_3.2 channels to mediate the secretion of the chemokine CXCL10 and the IGF-1 binding protein IGFBPL1. Even though we did not find a significant elevation of CXCL10 in vivo at the time point of our analysis, our results showed that IGFBPL1 was highly up-regulated in the striatal astrocytes of A53T Tg mice, in which the sustained NF-κB activation results in Ca_v_3.2 induction. IGFBPL1 has recently emerged as a molecular switch, turning inflammatory microglia to their homeostatic state to limit neuroinflammation [68]. The induction of IGFBPL1 via operation of astrocytic Ca_v_3.2 channels reveals a novel neuroprotective mechanism through which astrocytes safeguard neuronal integrity under conditions of chronic inflammation. Since we found a significant induction of pappalysin-2 (encoded by *PAPP-A2*) expression, a protease that specifically cleaves the IGF/IGFBP complex to potentiate IGF signaling [81], it is tempting to speculate that pappalysin-2 and IGFBPL1 synergistically promote IGF-1 signaling to protect from α-synuclein-produced inflammatory damage. Further experimental work is required to test whether IGFBPL1 targets neurons, microglia or astrocytes in our model.

### Relevance to human disease

Several genetic mouse models expressing the human WT or the mutant variant of α-synuclein have been generated, in which α-synuclein is expressed under the control of various promoters. Most of them can recapitulate, albeit to different extents, some characteristic biochemical and pathological features of PD, and have proven useful for identifying potential neuroprotective strategies for human disease [34]. The A53T Tg mice used in this study exhibit a moderate increase in α-synuclein expression, reflecting changes that can be found in human patients, i.e., when duplication or triplication of *SNCA* gene locus occurs. In this model, inflammation is documented by elevated levels of cytokines such as TNFα and IL-1β, which are also consistently increased in the PD brain. Even though we and others show no evidence of progressive neurodegeneration in the striatum and SNpc of A53T Tg mice, we found activation of the synapse-tagging mechanism, suggesting that defects related to synaptic maturation and function could be present in these areas as was recently shown for A53T-BAC-SNCA mice using high-resolution electron microscopy [82].

Even though our work was mainly focused on the striatum, the presence of similar α-synuclein oligomers and the profound increase of astrocytic Ca_v_3.2 VGCCs in the cortex and SNpc relate our findings also in the context of PD pathology. Striatum is a brain region severely affected in PD, mostly due to the loss of its dopaminergic innervation caused by neurodegeneration in the SNpc and to some extent from the denervation of striatal axon terminals. Our investigation was focused on neuroinflammation that is established early in the diseased brain, and is related with the development, maintenance and progression of the non-motor symptoms that precede cell death. As such, we selected an area in which neuronal viability and synaptic integrity are maintained and a transgenic mouse model (A53T Tg) that exhibits a moderate increase in α-synuclein expression (up to three-fold). In this model, the spontaneously generated oligomeric hyperphosphorylated α-synuclein conformers gradually accumulate in the striatum and are associated with elevated C3 complement, pro-inflammatory cytokines and increased levels of endogenous IgG antibodies. Similar species were found in other brain areas in mouse brain such as the cortex, midbrain and hippocampus, and also in human PD brain tissue, where they were also associated with C3 elevation.

Our data suggest that the α-synuclein-induced neuroinflammation in A53T Tg mice is linked with an up-regulation of Ca_v_3.2 VGCCs in astrocytes. Even though we could not directly assess astrocyte-specific Ca_v_3.2 expression in the human tissue, we found that Ca_v_3.2 mRNA was significantly increased in the putamen of PD patients compared to control individuals. The T-type Ca^2+^ channels have emerged as therapeutic targets for PD, particularly to attenuate the burst discharges in subthalamic neurons and improve the parkinsonian locomotor symptoms [55]. In fact, due to the growing evidence linking Ca_v_3.2 channels with neurological conditions, the T-type channels are now considered one of the most highly regarded druggable targets of the past decade, with > 40 patents (since 2012) for new small organic blockers of T-type channels [83, 84]. Our study adds to the functional properties of the T-type Ca_v_3.2 channels and underscores their involvement in the resolution of neuroinflammation in neurodegenerative disorders.

The finding that astrocytic Ca_v_1.2 channels are expressed in astrocytes in vivo is also important based on the use of isradipine, an L-type Ca^2+^ channel (LTCC) blocker currently approved as a drug for the treatment of high blood pressure, as a potential therapeutic approach for PD. However, despite the encouraging findings in pre-clinical models showing alleviation of the LTCC-mediated Ca^2+^ load in the dopaminergic neurons, isradipine failed to confer neuroprotection in a phase III clinical trial on early PD patients [85]. Our study revealed that Ca_v_1.2 channels are significantly decreased in the A53T Tg mouse model of synucleinopathy. In this paradigm, the use of selective LTCC agonists to restore Ca_v_1.2 activity will allow us to investigate the involvement of this channel in astrocyte function in future studies.

## Conclusions

We have defined the molecular mechanisms by which aberrant α-synuclein oligomers prolong neuroinflammation in vivo by sequentially activating specific signaling pathways in microglia and astrocytes. We have shown that these species are also present in the human PD brain. Further, we present a novel function of astrocytic T-type Ca_v_3.2 channels to counterbalance the α-synuclein-produced inflammation by mediating the secretion of the neuroprotective protein IGFBPL1. Even though the neuroprotective potential of IGFBPL1 has to be further verified in synucleinopathy models, this protein could represent a novel molecular target against α-synuclein-induced neuroinflammation.

Targeting this molecular mechanism could provide an alternative anti-inflammatory strategy in diseases associated with unconstrained activation of the NF-κB pathway such as synucleinopathies. However, the direct targeting of NF-κB signaling for therapy is challenging due to the vast cell-type heterogeneity of the brain tissue and the wide distribution of pleiotropic NF-κB activity in all cell types. In this context, our work highlights Ca_v_3.2 as a novel druggable molecular target to alleviate the damaging effects of microglial and astrocytic activation. Ca_v_3.2 regulation has been extensively studied in the context of inflammatory and neuropathic pain. Considering the ubiquitous expression of Ca_v_3.2 VGCCs and the structural similarities among T-type Ca^2+^ channels, the design of astrocyte-selective agonists or the indirect targeting of the ubiquitinases and de-ubiquitinases that regulate the turnover of Ca_v_3.2 channels could represent new neuroprotective approaches for synucleinopathies.

### Supplementary Information

**Additional file 1**: Supplementary Materials and Methods. **Figure S1**. Moderate overexpression of α-synuclein in A53T transgenic mice results in α-synuclein oligomerization and complement neuronal tagging in the absence of neuronal death. **Figure S2**. Morphological and biochemical alterations in A53T microglia and astrocytes in vivo.** Figure S3**. α-Synuclein expression motivates neuropeptide-, MAPK- and Ca^2+^ -dependent signaling pathways. **Figure S4**. PACAP activity is not induced in A53T transgenic mice. **Figure S5**. Investigation of the signalling pathways potentially involved in inflammatory responses in A53T transgenic mice. **Figure S6**. The p38, ATF-2 and NF-κB pathways are selectively activated in A53T glial cells. **Figure S7**. Localization of L- and T-type VGCCs in striatal neurons. **Figure S8**. Ca_v_2.1, Ca_v_2.2 and Ca_v_1.3 VGCCs are not expressed in mouse astrocytes in vivo. **Figure S9**. Primary quiescent astrocytes recapitulate biochemical and functional characteristics of mature astrocytes including responsiveness to cytokines. **Figure S10**. Nab2 and Rest mRNA levels are not altered in A53T Tg mice. **Figure S11**. Neuroinflammation and astrocytic Ca_v_3.2 upregulation is not restricted to the striatum of A53T mice. **Figure S12**. p38/NF-κB pathway and Ca_v_3.2 levels are not induced in microglia and astrocytes upon PFFs treatment. **Table S1**. Demographic information of non-PD and PD individuals. **Table S2**. Characteristics of mice groups used in the study. **Table S3**. Primer sequences used in qPCR analysis of mouse and human tissue. **Table S4**. List of antibodies used in the study.

**Additional file 2**: DEGs up- and down-regulated in WT and A53T Tg mice. Analysis of RNAseq data. Differentially up- and down-regulated genes in the striatum of WT and A53T Tg mice.

**Additional file 3**: Proteins detected in the CM of a1H- and mock-transfected astrocytes. Detection of all the proteins that are secreted from astrocytes 48 hours following transfection. Proteins identified in 3 independent experiments are listed.

**Additional file 4**: Sorting of the proteins detected in the CM of a1H- and mock-transfected astrocytes using the SignalP and SecretomeP databases. List of differentially secreted proteins from mock- and a1H-transfected astrocytes identified in three independent experiments.

## Data Availability

All data generated or analyzed during this study are included in this published article and its supplementary information files. The datasets produced during the current study can be also available from the corresponding author on reasonable request.

## Abbreviations

PD: Parkinson’s disease
GFAP: Glial fibrillary acidic protein
JNK: C-Jun N-terminal kinase
NF-κB: Nuclear factor-κB
IGFBPL1: Insulin like growth factor binding protein like 1
IGF1: Insulin-like growth factor 1
LB: Lewy bodies
CNS: Central nervous system
NLRP3: NOD-, LRR- and pyrin domain-containing 3
TLR2: Toll-like receptor 2
A53T Tg: A53T α-Synuclein transgenic mice
ATF2/7: Activating transcription factor 2/7
VGCC: Voltage-gated Ca^2+^ channel
WT: Wild type
Dox: Doxycycline
FGF2: Fibroblast growth factor-2
EGF: Epidermal growth factor
TNFα: Tumor necrosis factor α
IL-1β: Interleukin 1β
IFNγ: Interferon γ
PFFs: Preformed fibrils
Hprt1: Hypoxanthine–guanine Phosphoribosyltransferase 1
RPL13A: 60S ribosomal protein L13a
NGS: Normal goat serum
PFA: Paraformaldehyde
Iba1: Ionized calcium-binding adapter molecule 1
CHAPS: 3-[(3-Cholamidopropyl) dimethylammonio]-1-propane-sulphonate
ECL: Electrogenerated chemiluminescence
CM: Conditioned medium
FASP: Filter aided sample preparation
FDR: False discovery rate
TH: Tyrosine hydroxylase
SYB2: Synaptobrevin-2
NFL: Neurofilament
NE: Norepinephrine
DBH: Dopamine β-hydroxylase
PACAP: Pituitary adenylyl cyclase-activating peptide
VIP: Vasoactive intestinal peptide
DEG: Differentially expressed gene
MYLK: Myocin light chain kinase
IFNα: Interferon-α
LPS: Lipopolysaccharides
NAB2: NGFI-A-binding protein 2
REST: RE1 silencing transcription factor
Egr-1: Early growth response 1
IκΒα: Nuclear factor of kappa light polypeptide gene enhancer in B-cells inhibitor, alpha
CXCL10: C-X-C motif chemokine 10
PAPP-A: Pappalysin-1
