# Supplementary material for: α-Synuclein oligomers potentiate neuroinflammatory NF-κB activity and induce Cav3.2 calcium signaling in astrocytes
Source: Transl Neurodegener. 2024 Feb 21;13:11. doi: 10.1186/s40035-024-00401-4 (PMC10880263; doi:10.1186/s40035-024-00401-4)
Supplement: Supplementary file 1 — Additional file 1: Supplementary Materials and Methods. Figure S1. Moderate overexpression of α-synuclein in A53T transgenic mice results in α-synuclein oligomerization and complement neuronal tagging in the absence of neuronal death. Figure S2. Morphological and biochemical alterations in A53T microglia and astrocytes in vivo. Figure S3. α-Synuclein expression motivates neuropeptide-, MAPK- and Ca2+ -dependent signaling pathways. Figure S4. PACAP activity is not induced in A53T transgenic mice. Figure S5. Investigation of the signalling pathways potentially involved in inflammatory responses in A53T transgenic mice. Figure S6. The p38, ATF-2 and NF-κB pathways are selectively activated in A53T glial cells. Figure S7. Localization of L- and T-type VGCCs in striatal neurons. Figure S8. Cav2.1, Cav2.2 and Cav1.3 VGCCs are not expressed in mouse astrocytes in vivo. Figure S9. Primary quiescent astrocytes recapitulate biochemical and functional characteristics of mature astrocytes including responsiveness to cytokines. Figure S10. Nab2 and Rest mRNA levels are not altered in A53T Tg mice. Figure S11. Neuroinflammation and astrocytic Cav3.2 upregulation is not restricted to the striatum of A53T mice. Figure S12. p38/NF-κB pathway and Cav3.2 levels are not induced in microglia and astrocytes upon PFFs treatment. Table S1. Demographic information of non-PD and PD individuals. Table S2. Characteristics of mice groups used in the study. Table S3. Primer sequences used in qPCR analysis of mouse and human tissue. Table S4. List of antibodies used in the study. [file 40035_2024_401_MOESM1_ESM.docx]

**Additional file 1.**

**A. Supplementary Materials and Methods**

**RNA extraction and Quantitative PCR**

Total RNA was extracted from 20 mg of striatal mouse tissue using TRIzol Reagent (15596026, ThermoFisher, Invitrogen™) according to the manufacturer’s instructions. Mice were euthanized by isoflurane exposure and the striatum was dissected and homogenized in TRIzol Reagent. Before homogenization, tissue was extensively washed with PBS to remove residual blood. Total RNA was extracted from 20 mg of human tissue (putamen and caudate nucleus) in a similar manner. Following DNase I (2270B, TaKaRa) treatment, 1.5 μg of RNA was used for cDNA synthesis using PrimeScript reverse transcriptase (2680A, Takara). qPCR was performed using Kapa SYBR FAST qPCR Master Mix (5515, Kapa Biosystems) using specific primers for each gene and Hypoxanthine-guanine Phospho-ribosyltransferase 1 (*Hprt1*) or 60S ribosomal protein L13a (RPL13A) as housekeeping genes for mouse and human cDNA samples, respectively (Supplementary Table 3). All samples were amplified in triplicates and analyzed using the comparative Cycle threshold (CT) method 2^−ΔΔCT^. Primers were designed with the help of Primer designing tool (https://www.ncbi.nlm.nih.gov/tools/primer-blast) against mouse and human genomic sequences.

**RNA sequencing and bioinformatic analysis**

RNASeq analysis was performed in duplicates in the Greek Genome Center (GGC) of the Biomedical Research Foundation of the Academy of Athens (BRFAA). RNASeq libraries were prepared with the illumina TruSeq RNA v2 kit, with 1 μg of total high-quality total RNA input from striatum of Wt and A53T Tg mice. Libraries were checked with the Agilent bioanalyzer DNA1000 chip, quantitated with the qubit HS spectrophotometric method and pooled in equimolar amounts for Sequencing. Approximately 25Million, 100 bp long, Single-End reads were allocated per sample in the illumina NextSeq 500 sequencer. Bioinformatic analysis involved the steps of quality improvement, estimation of transcript abundance and differential expression analysis ^[27]^. RNA-seq raw sequencing data were aligned to mouse genome version GRCm38/mm10 using tophat (version 2.0.9). Samtools (version 0.1.19) were used for data filtering and file format conversion while HT-seq count (version 0.6.1p1) algorithm was performed for assigning aligned reads into exons. Genes with length < 500bp or average read counts per 100bp < 0.25 quantile of the average normalized read count distribution per 100bp in the gene body, were excluded to avoid possible statistical artifacts. The evaluation of the differentially expressed genes (DEGs) was performed by filtering as: {log2FC >1 or log2FC < -1} and {p.value < 0.01}, FC = fold-change of reads per kilobase per million.

**Immunocytochemistry**

5 x 10^4^ cells were plated in coverslips and fixed with 3.7% formaldehyde (104002, Millipore) in PBS for 15 min at 4^o^C. Cells were washed twice with PBS, blocked with 10% Normal Goat Serum (NGS) (G6767, Sigma-Aldrich) supplemented with 0.1% Triton-X100 (A4975,1000, AppliChem) and incubated with primary antibodies (Supplementary Table 4) in blocking buffer containing 2% NGS and 0.1% Triton-X100 in PBS for 16 hours at 4^o^C. Cells were washed and incubated with Alexa Fluor-488/594/647 goat anti-rabbit or anti-mouse IgG (H+L) (Invitrogen) at a dilution of 1:2000 and the nuclear marker DAPI (268298 Merck Millipore) (1:2000), in blocking buffer, for 2 hours in the dark. Coverslips were mounted with Vectashield medium and cells were visualized using SP5-II confocal microscope (Leica) or a Cytation 5 Cell Imaging Multimode Reader (Agilent).

**Immunohistochemistry**

Mice were anesthetized by isoflurane and transcardially perfused with 50 ml ice cold PBS for blood clearance, followed by 50 ml 4% paraformaldehyde (PFA) (D6148, Sigma-Aldrich). For free-floating cryosections, brains were isolated, post-fixed with 4% PFA, for 6 hours at 4^o^C and dehydrated by sequential incubations in 15% and 30% sucrose. Dehydrated brains were frozen in isopentane at -45^o^C and sectioned using a Bright cryostat at a 25 μm thickness. For paraffin-embedded sections, brains were post-fixed with 4% PFA for 4 hours, washed extensively with water, incubated for 48 hours in 70% Ethanol at 4^o^C, and further proceeded for paraffinization. Brains were sectioned using a vibratome at a thickness of 7 μm. Prior to immunolabeling, sections were de-paraffinized by heating at 60^o^C for 10 min and re-hydrated through a series of ethanol and water baths.

When required, antigen retrieval was performed by incubating sections in 10 mM citrate buffer, pH 6, for 30 min at 80^o^C. After antigen retrieval, sections were washed three times with PBS and blocked in 5% NGS supplemented with 0.1% Triton-X100 in PBS. Primary antibodies (Supplementary Table 3) were added in blocking buffer for 16 hours (paraffin sections) or 48 hours (free-floating cryosections). Sections were washed with PBS and incubated with Alexa Fluor-488/594/647 goat anti-rabbit or anti-mouse IgG (H+L) at a dilution of 1:2000 and the nuclear marker DAPI (1:2000), in blocking buffer, for 2 hours in the dark. Following mounting, sections were visualized using a Leica SP5-II confocal microscope.

**Immuno-Electron Microscopy**

Mice were anesthetized by isoflurane and transcardially perfused with 4% PFA, 0.5% glutaraldehyde in 100 mM Na_2_HPO_4_ for 20 min. Brains were dissected and immersed at 4% PFA and kept overnight at 4^o^C. Brains were sectioned at a thickness of 70 μm using a vibratome, and free-floating sections were stored at -20^o^C in cryoprotectant buffer (40 mM Na_2_HPO_4_, 8% v/v glycerol, 20% sucrose). The immunoreactions were performed as previously described ^[28]^. In brief, free-floating sections were thoroughly washed in 0.1M phosphate buffer (pH 7.1) and incubated with pairs of primary antibodies (36-48 h, 0-4^o^C) in 50 mM Tris-buffer saline (pH 7.4). After washing in the same buffer, the sections were incubated with the respective nanogold and biotin labeled antibodies (36-48 h, 0-4^o^C). The gold signal was then silver enhanced, whereas an ABC-DAB reaction was used for the biotinylated antibodies. Subsequently, the sections were contrasted with osmium tetroxide and uranyl acetate, dehydrated in a series of ethanol, soaked in Durcupan epoxy resin and flat-embedded on microscope slides. After resin polymerization, regions of interest were re-embedded in resin blocks, ultrathin sections (70 nm) were cut in a Leica UCT6 microtome and collected on copper slot grids. Ultrathin sections were stained with lead and examined in the HR-Jeol transmission electron microscope (JEM-2100).

**Immunoblotting**

50 μg of total protein were analyzed by denaturing gel electrophoresis (SDS-PAGE) in Tris-glycine buffer. Proteins were normally denatured at 95^o^C for 3 minutes or 45^o^C for 15 minutes (in the case of Ca_v_3.2 immunodetection). Immunoblotting was carried out following protein transfer to nitrocellulose membranes and blocking with 5% (w/v) skimmed milk or 5% (w/v) BSA in 50 mM Tris-HCl, pH 7.5, 150 mM NaCl, 0.1% Tween 20 (TBS-T) buffer. For Ca_v_s immunodetection, blots were first incubated with 0.2 M Glycine, 0.5 M NaCl, pH 2.8 for 20 minutes to allow epitope exposure, and then blocked with 5% BSA. Primary antibodies (Supplementary Table 4) were applied for 16 hours at 4^o^C, washed in TBS-T buffer and incubated with a peroxidase-conjugated anti-mouse (1:10,000 dilution; ab205719, Abcam) or anti-rabbit (1:10,000 dilution; ab205718, Abcam) secondary antibody for 1 hour. Protein signals were detected using electrogenerated chemiluminescence (ECL) reagents according to manufacturer’s recommendations (Thermo Fisher Scientific Inc.). The intensity of the immunoreactive bands was quantified by densitometric quantification using the GelQuant.NET software provided by biochemlabsolutions.com. GAPDH or β-actin was used as loading control for normalization.

**B. Supplementary figures**

**
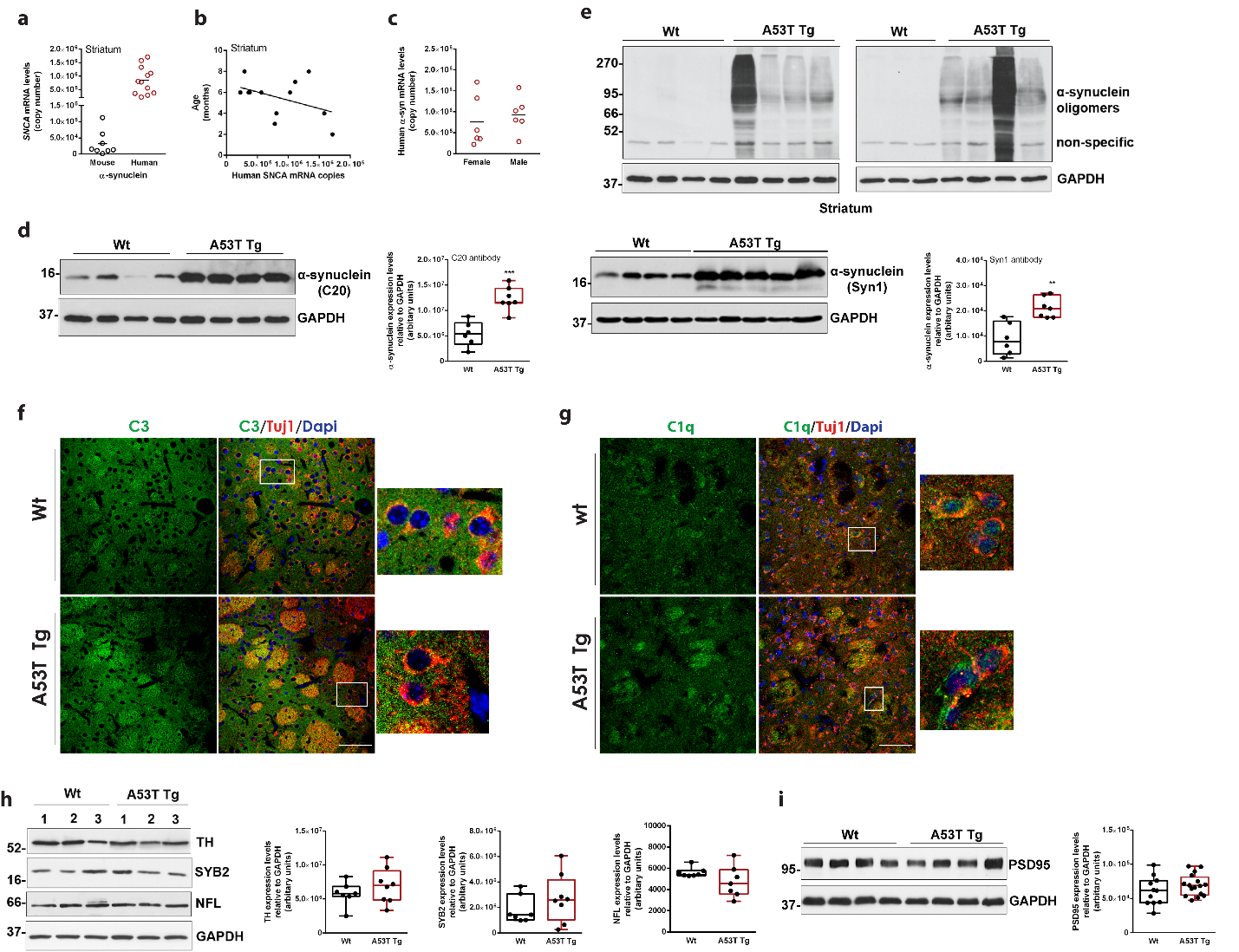
**

**Figure S1. Moderate overexpression of α-synuclein in A53T transgenic mice results in α-synuclein oligomerization and complement neuronal tagging in the absence of neuronal death. (a)** mRNA levels of mouse (*n=*8) and human (*n=*12) SNCA in the striatum of A53T Tg mice. **(b)** Correlation analysis of human SNCA mRNA levels in the striatum of A53T Tg (*n=*12) mice with the animal age. **(c)** mRNA levels of human SNCA in the striatum of male and female A53T Tg mice (*n=*6 per gender, *P=*0.5836) **(d)** Representative western blots of CHAPS-generated striatum homogenates from Wt and A53T Tg mice showing the levels of monomeric α-synuclein and densitometric quantification of antibodies against α-synuclein, Syn1, ***P=*0.0015 and C20, ****P=*0.0003 antibodies (*n=*7 per group). GAPDH was used as loading control. **(e)** Representative western blots of CHAPS-homogenized striatum using antibodies against total α-Synuclein (anti-Syn1) showing the presence of α-synuclein oligomers. **(f-g)** Representative confocal images of Wt and A53T Tg striatal sections immunostained with specific antibodies against C3, C1q and the neuronal marker β3-tubulin (tuj1). Scale bar 50 μm. **(h)** Representative immunoblot of striatum homogenates from Wt and A53T Tg mice and densitometric analysis using antibodies against the neuronal markers TH (*P=*0.3349), SYB2 (*P=*0.2982) and NFL (*P=*0.1632) of Wt (*n=*7) and A53T Tg (*n=*8) mice. **(i)** Representative immunoblot and densitometric quantification of PSD95 (*P=*0.2199) in Wt (*n=*11) and A53T Tg (*n=*16) mice. GAPDH was used as loading control. For all graphs data are represented as means ± SEM. Statistics by Unpaired Student’s t test.

**
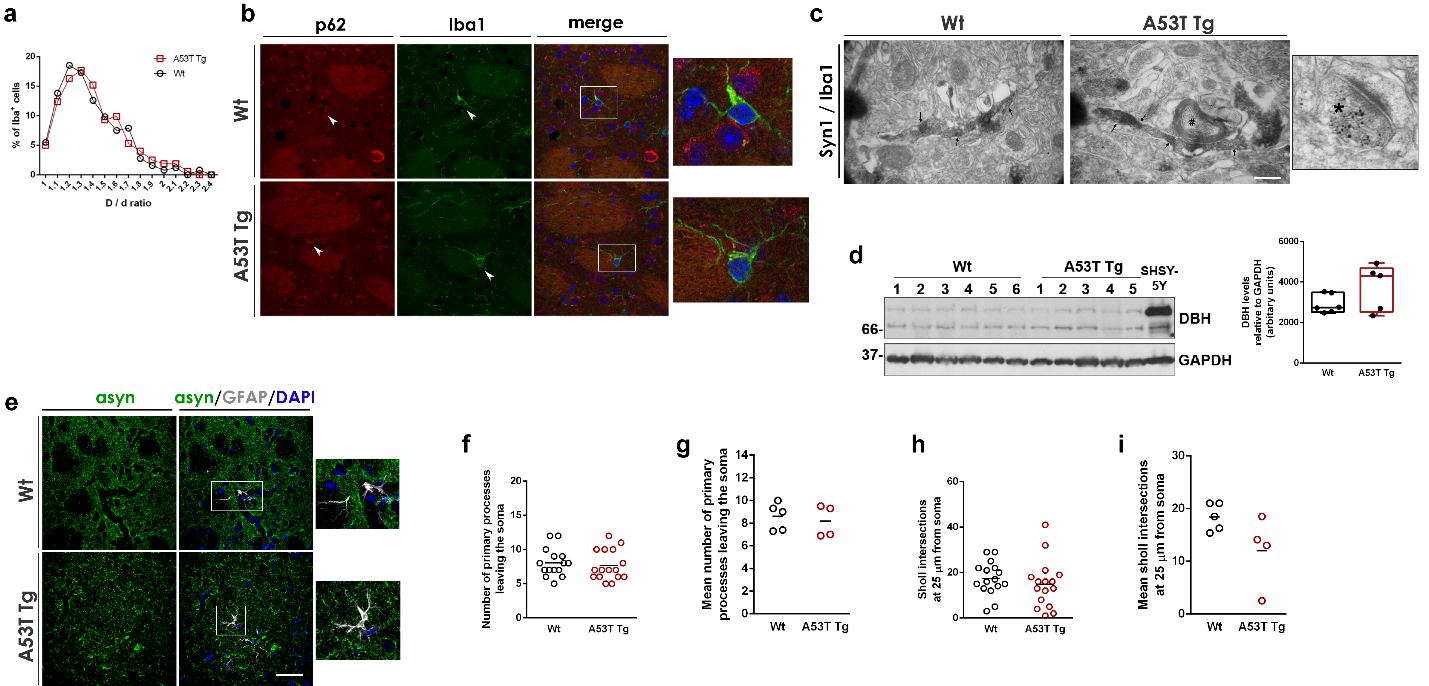
**

**Figure S2. Morphological and biochemical alterations in A53T microglia and astrocytes *in vivo*. (a)** Distribution of the size of Iba1^+^ cell soma by measuring the major and minor axes (D/d ratio) (N≥3 mice per genotype). **(b)** Representative confocal images depicting immunostaining against p62 and Iba1 in Wt and A53T Tg striatal sections. DAPI (blue) was used for nuclei staining. Scale bar: 50 μm. **(c)** Representative immuno-EM images of striatal sections from Wt and A53T Tg mice showing Syn1 antibody labeled with gold nanoparticles co-stained with Iba1-DAB. α-synuclein localization at presynaptic terminals is marked with an asterisk. **(d)** Immunoblotting analysis of striatum homogenates from Wt and A53T Tg mice using an antibody against DBH. SH-SY5Y cell lysates were used as a positive control of DBH expression. Densitometric quantification in Wt (*n=*6) and A53T Tg (*n=*5) mice is shown on the right, *P=*0.1397. **(e)** Representative confocal images depicting immunostaining against α-synuclein and GFAP in Wt and A53T Tg striatal sections. DAPI (blue) was used for nuclei staining. Scale bar: 50 μm. (**f-h**) Morphometric features measured in 16 astrocytes per genotype from 5 Wt and 4 A53T Tg mice; (**f**) number of primary processes leaving the soma (*P=*0.6252, df=29), (**g**) mean number of primary processes leaving the soma pre-averaged per animal (*P=*0.64, df=7), (**h**) number of intersections at 25 μm from the soma (*P=*0.4338, df=30), (**i**) mean number of intersections at 25 μm from the soma pre-averaged per animal (*P=*0.0894, df=7). Statistics in all graphs were performed by Unpaired Student’s t test.


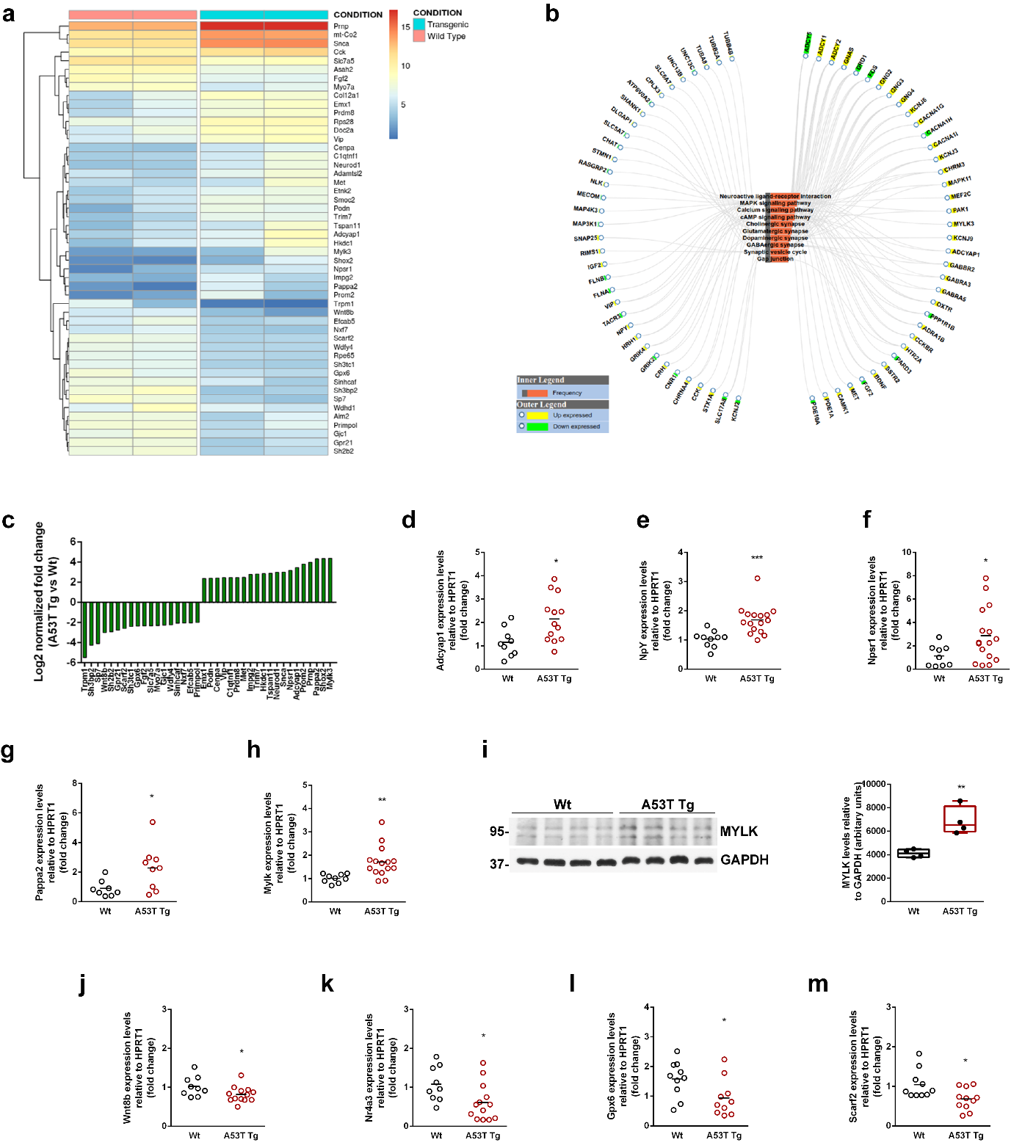


**Figure S3. α-Synuclein expression motivates neuropeptide-, MAPK- and Ca^2+^ -dependent signalling pathways. (a)** Expression heatmap of the top 50 DEGs in A53T Tg mice compared with Wt mice by RNAseq. Samples are grouped by genotype. **(b)** Enrichment analysis showing the top 10 most significant molecular pathways changed in A53T Tg mice. **(c)** Log2 fold change of the genes exhibiting the most significant differences in expression. **(d-h)** Transcript expression of Adcyap1 (**P=*0.0142), NpY (****P=*0.0009), Npsr1 (**P=*0.0456), Pappa2 (**P=*0.026) and Mylk (***P=*0.0052) (*N*≥7 and *N*≥9 for Wt and A53T Tg mice, respectively). **(i)** Western blot detection and quantification of MYLK protein (*n=*4 mice per genotype, ***P=*0.0046). GAPDH was used as loading control. **(j-m)** Transcript expression of Wnt8b (**P=*0.0462), Nr4a3 (**P=*0.0302), Gpx6 (**P=*0.0325) and Scarf2 (**P=*0.0169) (*N*≥9 and *N*≥10 for Wt and A53T Tg mice, respectively). Data are presented as means ± SEM. Statistics by Unpaired Student’s t test.

**
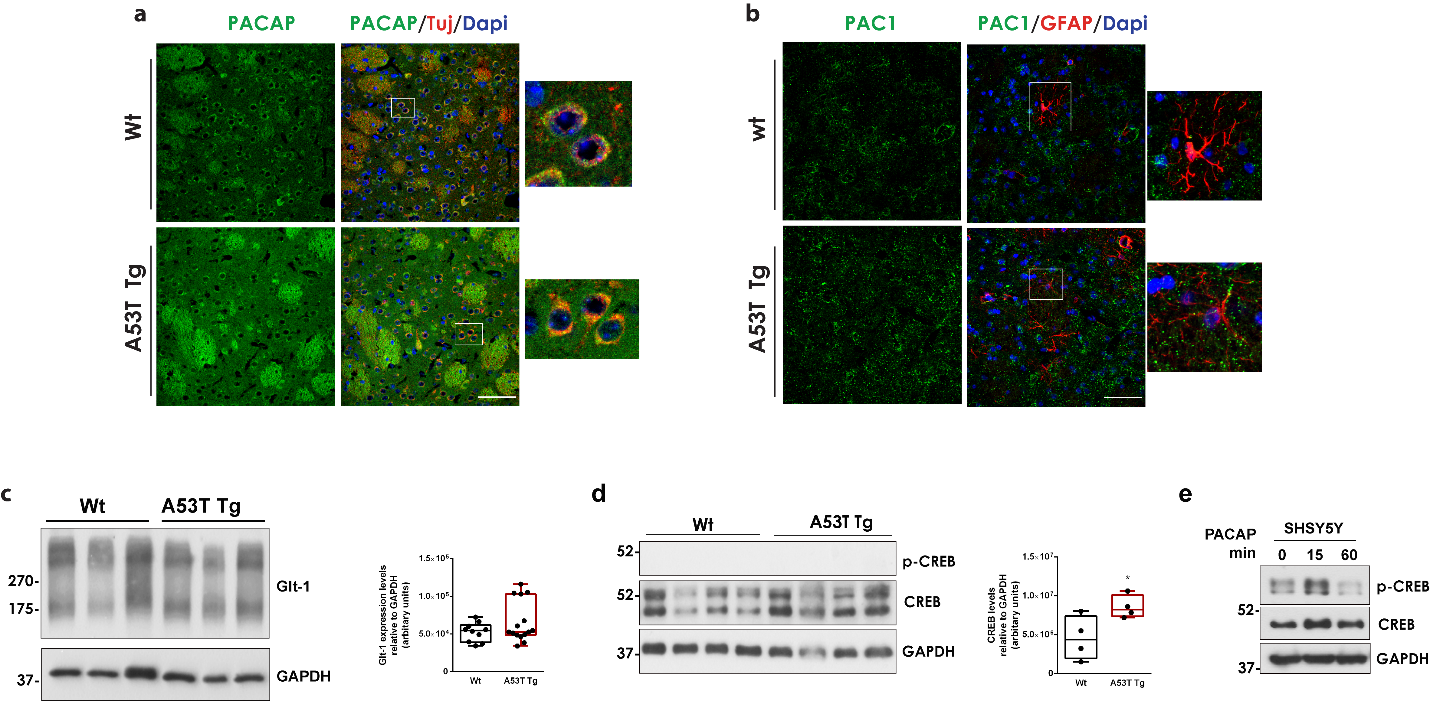
**

**Figure S4. PACAP activity is not induced in A53T transgenic mice. (a, b)** Representative confocal images from striatal sections of Wt and A53T Tg mice using antibodies against (a) PACAP and Tuj1 and (b) PAC1 and GFAP. **(c)** Western blotting and densitometric analysis of striatum homogenates from Wt (*n=*10) and A53T Tg (*n=*15) mice using a specific antibody against Glt-1 (*P=*0.1671). **(d)** Western blotting analysis of Wt and A53T Tg striatal lysates for the assessment of CREB pathway activation using phosphorylated CREB antibody. Densitometric quantification for non-phosphorylated CREB (*n=*4 per group, **P=*0.0457). **(e)** Appropriate antibody binding (p-CREB) was verified by immunoblotting of SH-SY5Y cell lysates treated with synthetic PACAP. GAPDH was used as loading control. For all graphs data are represented as means ± SEM. Statistics by Unpaired Student’s t test.

**
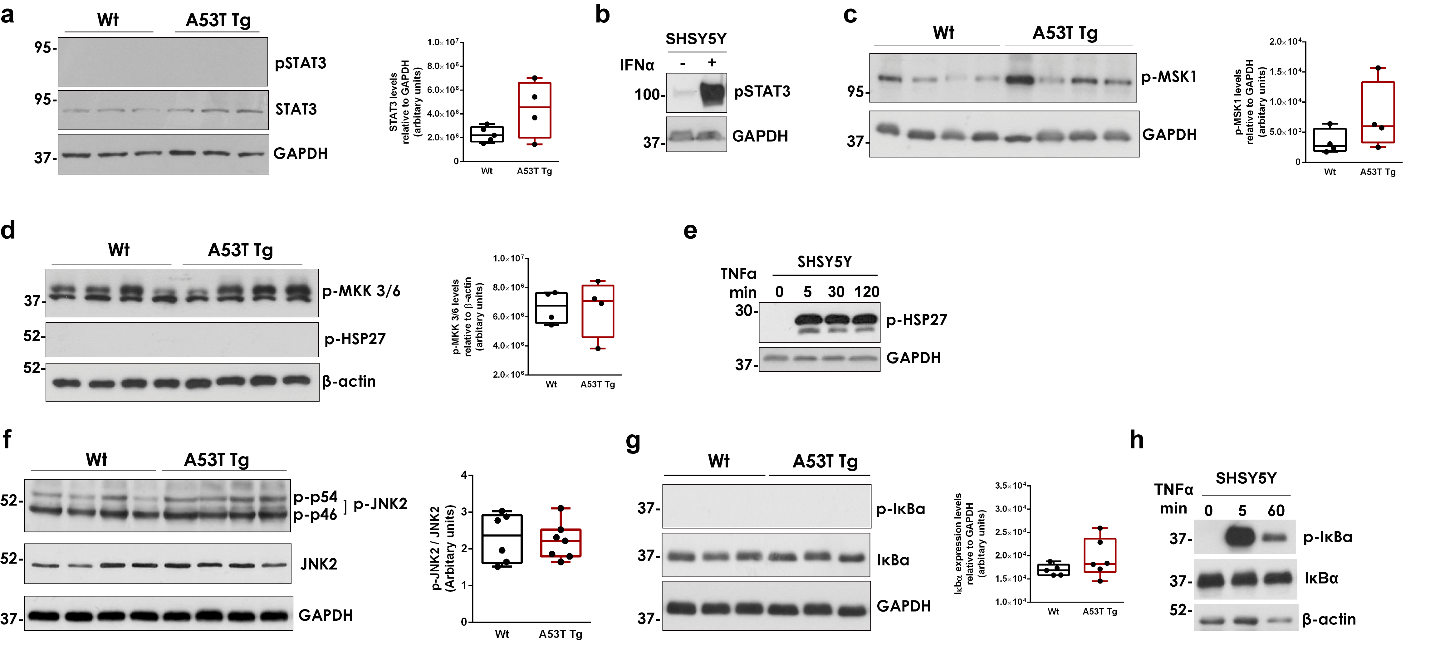
**

**Figure S5. Investigation of the signalling pathways potentially involved in inflammatory responses in A53T transgenic mice.** Western blotting analysis of Wt and A53T Tg (N≥4 per genotype) striatal lysates for the assessment of **(a)** phospho-STAT3 and total STAT3 (*P=*0.0956) **(c)** phospho-MSK1 (*P=*0.2181), **(d)** phospho-HSP27 and phospho-MKK3/6 (*P=*0.9649), **(f)** phospho-JNK2 relative to total JNK2 (*P=*0.7865) levels and **(g)** phospho-IκΒα and total IκΒα (*P=*0.2168). Appropriate antibody binding was verified by immunoblotting of SH-SY5Y cell lysates treated with **(b)** IFNα and **(e, h)** TFNα. GAPDH was used as a loading control. For all graphs data are represented as means ± SEM. Statistics by Unpaired Student’s t test.

**
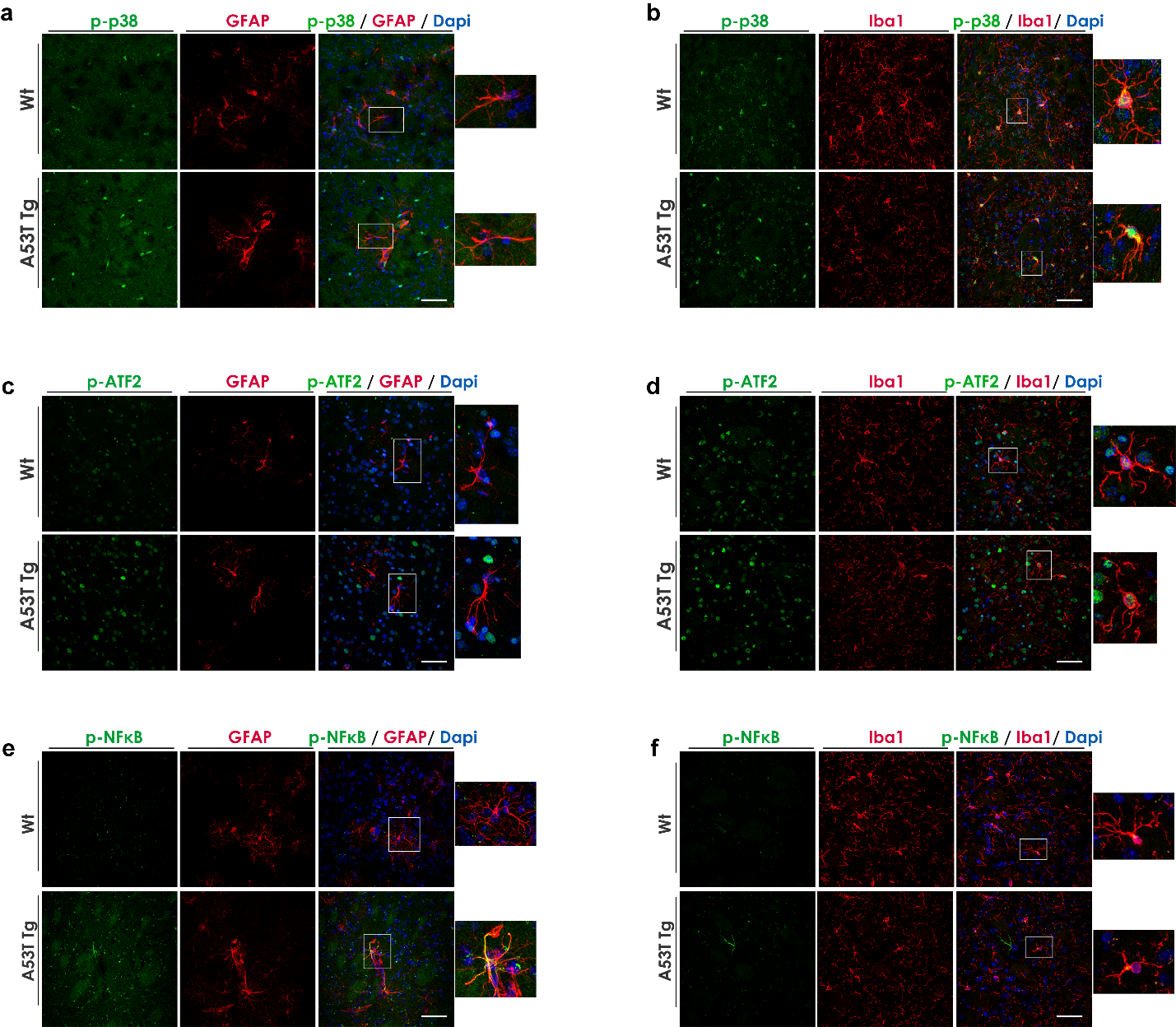
**

**Figure S6. The p38, ATF-2 and NF-κB pathways are selectively activated in A53T glial cells.** Representative confocal images of striatal sections from Wt and A53T Tg mouse brains co-stained with antibodies against (**a, b**) phospho-p38, (**c, d**) phospho-ATF2/7, (**e, f**) phospho-NF-κB and the microglial marker, Iba1 or the astrocytic marker, GFAP. DAPI (blue) was used for nuclei staining. Scale bar: 50 μm.

**
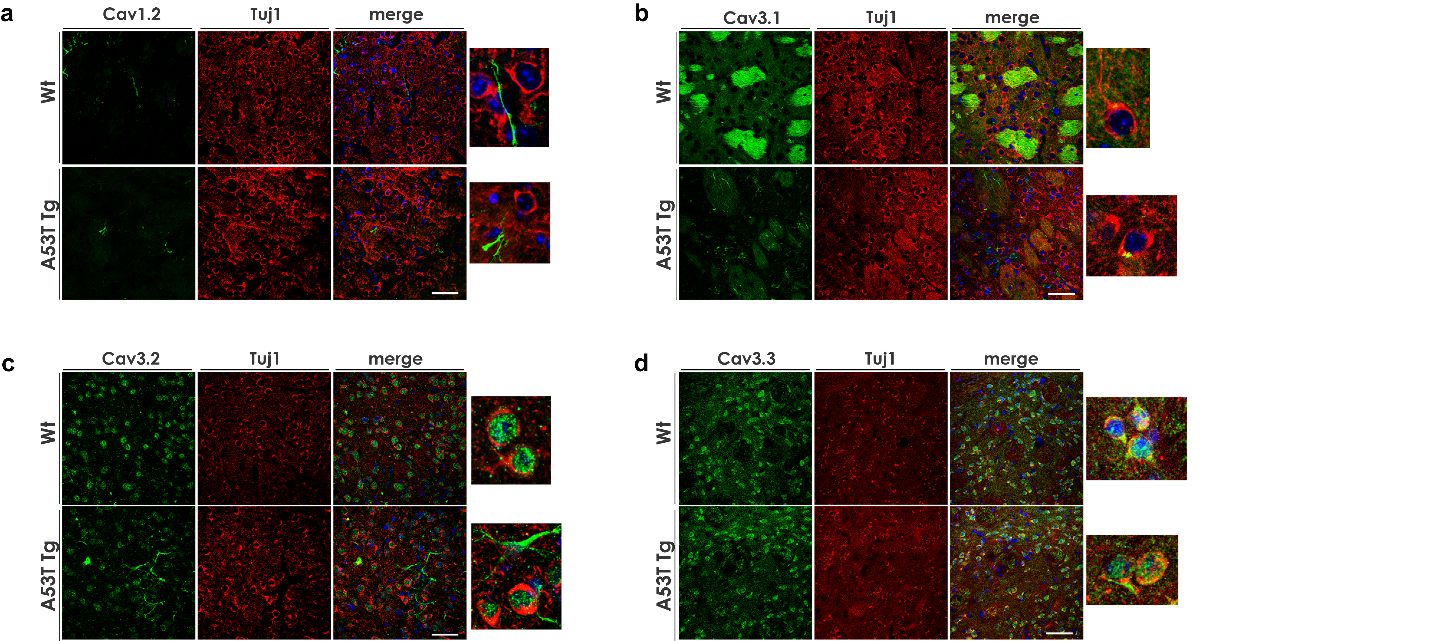
**

**Figure S7. Localization of L- and T-type VGCCs in striatal neurons.** Representative confocal images of striatal sections from Wt and A53T Tg mouse brains co-stained with antibodies against (**a**) Ca_v_1.2, (**b**) Ca_v_3.1, (**c**) Ca_v_3.2 and (**d**) Ca_v_3.3 and the neuronal marker b3-tubulin (Tuj1). DAPI (blue) was used for nuclei staining. Scale bar: 50 μm**.**

**
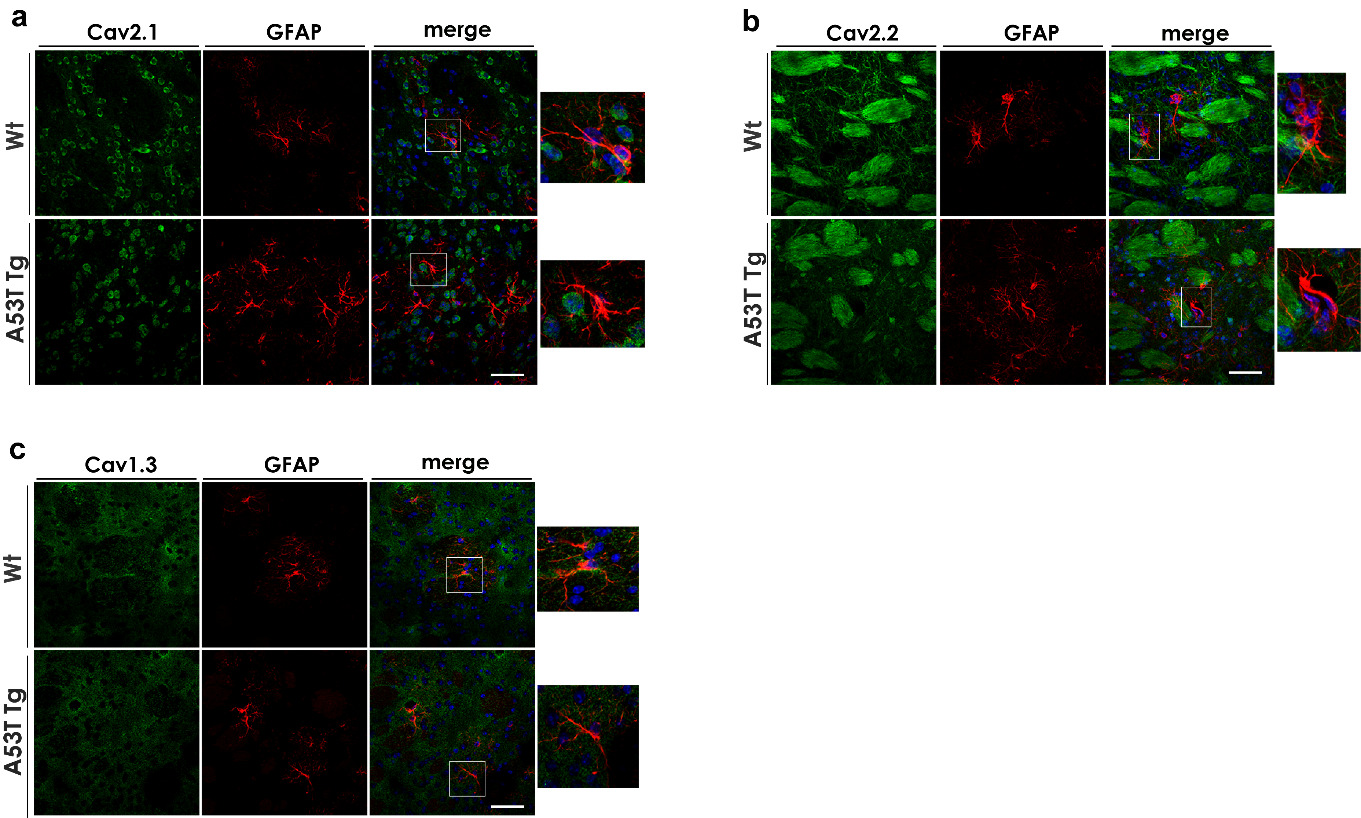
**

**Figure S8. Ca_v_2.1, Ca_v_2.2 and Ca_v_1.3 VGCCs are not expressed in mouse astrocytes *in vivo*.** Representative confocal images of striatal sections from Wt and A53T Tg mouse brains co-stained with antibodies against (a) Ca_v_2.1, (b) Ca_v_2.2 and (c) Ca_v_1.3 and the astrocytic marker GFAP. DAPI (blue) was used for nuclei staining. Scale bar: 50 μm**.**

**
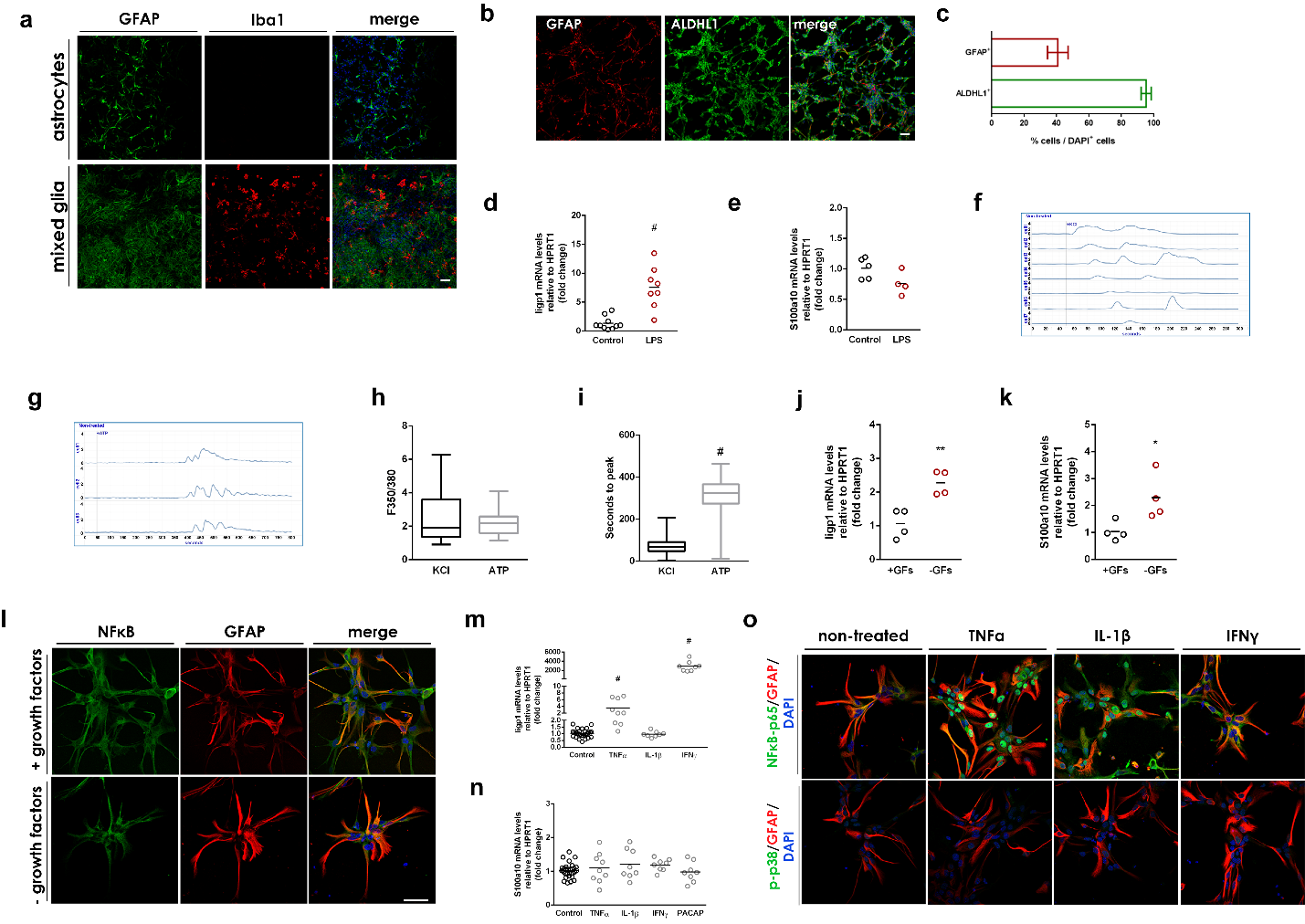
**

**Figure S9. Primary quiescent astrocytes recapitulate biochemical and functional characteristics of mature astrocytes including responsiveness to cytokines. (a)** Representative confocal images of primary quiescent astrocytes and mixed glial culture co-stained with antibodies against the astrocytic marker GFAP and the microglial marker Iba1. **(b)** Representative confocal images of primary quiescent astrocytes co-stained with the astrocytic markers GFAP and ALDH1L1 and **(c)** quantification of the number of GFAP^+^ and ALDH1L1^+^ cells using FIJI cell counter. **(d, e)** Measurement of (d) Ιigp1 (^#^*P*< 0.0001, N≥8 per group) and (e) S100a10 (*P=*0.0762, N≥4) mRNA levels by qPCR in control and LPS-treated astrocytes. **(f, g)** [Ca^2+^]i measurements showing the response of clustered astrocytes to (f) KCl and (g) ATP, using Fura-2AM and live calcium imaging. Tableau Software was used for data visualization. **(h)** Quantification of fluorescence ratio intensity F350/F380 in KCl and ATP (*P=*0.2090, *n=*47 astrocytes per group) stimulated astrocytes. **(i)** Quantification of the astrocytic response time (in seconds) to reach [Ca^2+^]i peak after KCl and ATP (^#^*P*< 0.000, N≥46 astrocytes per group) stimulation. **(j, k)** Measurement of (j) Iigp1, ***P=*0.0052 and (k) S100a10, **P=*0.0346 (*n=*4 per group) mRNA levels by qPCR in astrocytes cultured in the presence (+GF) or in the absence (-GF) of growth factors. **(i)** Representative confocal images from astrocytes cultured with or without growth factors co-stained with specific antibodies against NFκB-p65 and the astrocytic marker GFAP. **(m, n)** qPCR measurement of (**m**) Iigp1 mRNA levels following treatment with TNFα (^#^*P*< 0.0001), IL-1β (*P=*0.6206) and IFNγ (^#^*P*< 0.0001) (N≥9 per condition) and of (**n**) S100a10 mRNA levels following treatment with TNFα (*P=*0.9201), IL-1β (*P=*0.3312) and IFNγ (*P=*0.4541) (N≥9 per condition). **(o)** Representative confocal images of primary astrocytes treated with 20 ng/ml TNFα, 5 ng/ml IL1β or 1 ng/ml IFNγ for 5 minutes co-stained with antibodies against the astrocytic marker GFAP and an antibody specific to NFκB-p65 or phospho-p38. DAPI (blue) was used for nuclei staining. Scale bar: 50 μm. For all graphs data are represented as means ± SEM. Statistics by Unpaired Student’s t test.

**
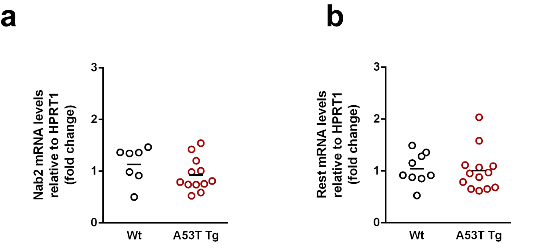
**

**Figure S10. Nab2 and Rest mRNA levels are not altered in A53T Tg mice.** **(a, b)** mRNA measurement in Wt and A53T Tg striatum; *P=*0.2053 and *P=*0.8302 for Nab2 and Rest, respectively (N≥7 per genotype). Statistics by Unpaired Student’s t test.

**
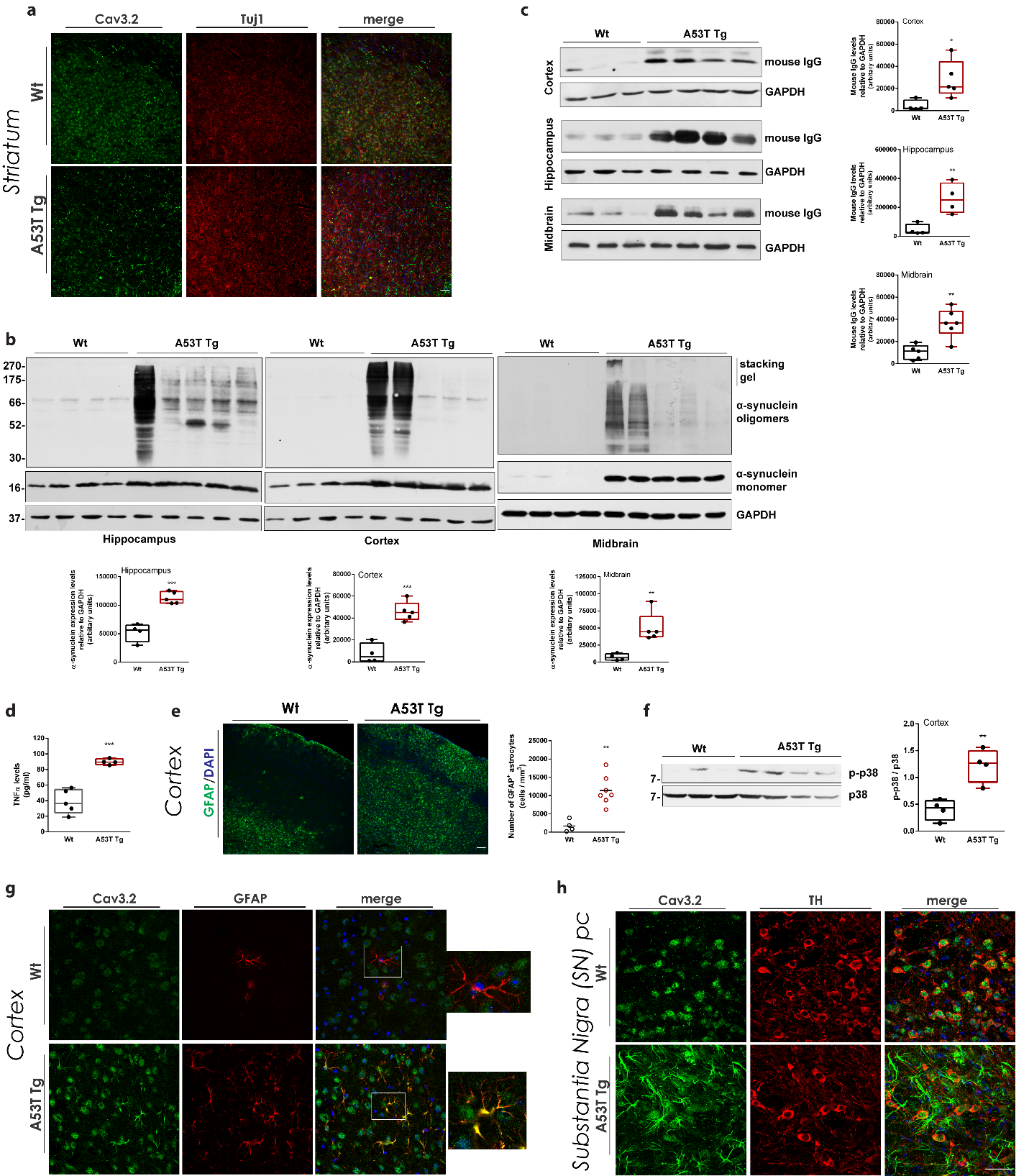
**

**Figure S11. Neuroinflammation and astrocytic Ca_v_3.2 upregulation is not restricted to the striatum of A53T mice. (a)** Representative confocal images of striatal sections from Wt and A53T Tg mouse brains co-stained with antibodies against Ca_v_3.2 and b3-tubulin (Tuj1), or Tyrosine-Hydroxylase (TH), respectively. Scale bar, 50 μm**. (b)** Representative western blots of CHAPS-homogenized hippocampus (****P=*0.0002), cortex (****P=*0.0004) and midbrain (***P=*0.0065) (N≥4, per group) using antibodies against total α-Synuclein (anti-Syn1) and densitometric quantification. **(c)** Immunoblots of mouse endogenous IgG and densitometric quantification in Wt and A53T Tg (N≥4, per group) mice in cortex (**P=*0.0292), hippocampus (***P=*0.0083) and midbrain (***P=*0.0028). **(d)** TNFα levels in cortical homogenates using cytokine-specific ELISA assays (N≥4 per genotype, ****P=*0.0004). **(e)** Representative low magnification confocal images of Wt and A53T Tg cortical sections stained with a GFAP antibody and quantification of the number of GFAP^+^ astrocytes in the cortex (Scale bar: 200 μm, N≥4 per genotype, ***P=*0.0015). DAPI (blue) was used to stain the nucleus. **(f)** Immunoblotting analysis and quantification of phospho-p38^MAPK^ levels relative to total p38^MAPK^ expression (*n=*4 mice per genotype, ^**^*P=*0.0042) in cortex. **(g, h)** Representative confocal images of Wt and A53T Tg cortical and SNpc sections stained with antibodies for Ca_v_3.2 and GFAP or TH, respectively. DAPI (blue) was used to stain the nucleus Scale bar: 50 μm.

**
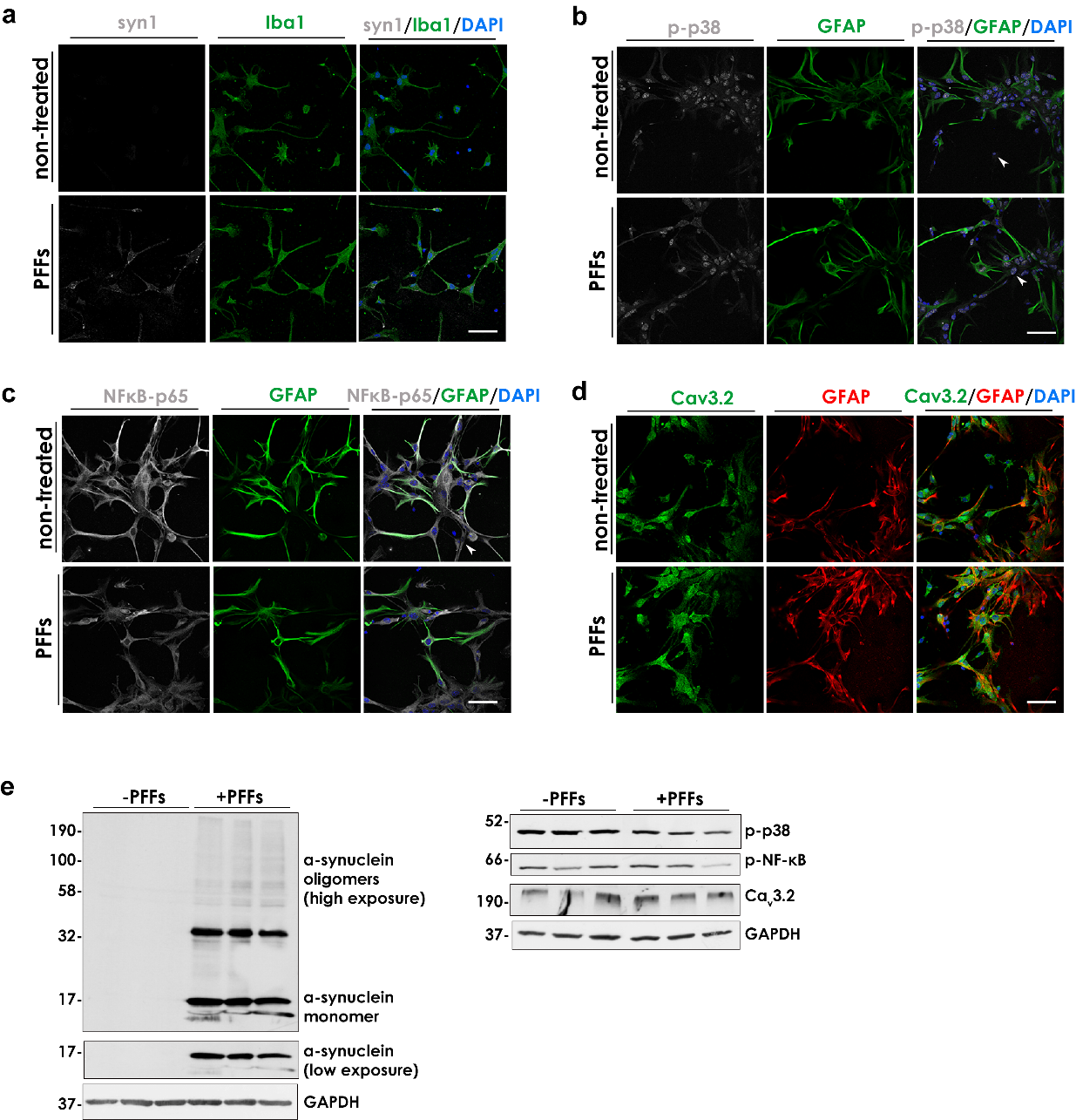
**

**Figure S12. p38/NF-κB pathway and Ca_v_3.2 levels are not induced in microglia and astrocytes upon PFFs treatment.** (**a-d**) Control and PFF-treated co-cultures of quiescent astrocytes and primary microglial cells co-stained with antibodies against α-synuclein, Iba1, phospho-p38, NFκB-p 65, GFAP and Ca_v_3.2. DAPI (blue) was used for nuclei staining. Scale bar: 50 μm. (**e**) Western blot analysis of control (-PFFs) and PFF-treated (+PFFs) co-cultures of quiescent astrocytes and primary microglial cells for the detection of α-synuclein, phospho-NF-κB, phospho-p38 and Ca_v_3.2 using specific antibodies. GAPDH was used as loading control.

**C. Supplementary tables**

**Table S1.** Demographic information of non-PD and PD individuals.

| Subjects | Non-PD | PD |
| --- | --- | --- |
| Age of death (years ± STD) | 83 ± 9 | 75 ± 5 |
| Gender (% Male) | 50% | 87% |
| Disease duration (years ± STD) | - | 14 ± 8 (range 5-26) |
| Age of onset (years ± STD) | - | 61 ± 11 (range 43-75) |

**Table S2.** Characteristics of mouse groups used in the study.

| Genotype | hu A53T SNCA^-/-^ (Wt) | hu A53T SNCA^+/+^ (A53T Tg) |
| --- | --- | --- |
| Number of mice used | 59 | 54 |
| Sex (%Female) | 39% | 50% |
| Age (months) | 6.4 ± 2.3 (range 4-11) | 7.1 ± 2.5 (range 4-11.5) |

**Table S3.** Primer sequences used in qPCR analysis of mouse and human tissue.

| Species | Primer  (Forward, F or Reverse, R) | Nucleotide sequence | Product  (bp) |
| --- | --- | --- | --- |
| Mus musculus *Hprt1* | F | AGTCCCAGCGTCGTGATTAG | 88 |
|  | R | TTTCCAAATCCTCGGCATAATGA |  |
| Mus musculus *Iigp1* | F | ACCGAGGGCTATTCCTCTCA | 100 |
|  | R | GTGAAGAGAACAGCTGACCCA |  |
| Mus musculus *Gbp2* | F | AGGCCAATTGAGAGTGAGGC | 166 |
|  | R | CCTCCAGCAAGTCTGAGCAA |  |
| Mus musculus *S100a10* | F | CTAGCCTCATCGTGGTGTGC | 156 |
|  | R | TCAAGTGGTCTTTGTCGCCT |  |
| Mus musculus *Adcyap1* | F | CAGTGTCTCCTGTTCACCTGC | 174 |
|  | R | TCCGCTGGATAGTAAAGGGC |  |
| Mus musculus *Npy* | F | TCATCACCAGACAGAGATATGGC | 212 |
|  | R | CATTGGTGGGACAGGCAGAC |  |
| Mus musculus *Mylk* | F | GCCAGGATTTTGTGCTACGG | 81 |
|  | R | TCCTTCACCTTTCTTCTCTTGG |  |
| Mus musculus *Wnt8b* | F | TTATGAAGCCCGTGTGCGT | 107 |
|  | R | GTAGACCAGGTAAGCCTTTGGA |  |
| Mus musculus *Scarf2* | F | CCAAATGTGACCAACCCTGC | 182 |
|  | R | ACAGTCCTCACCGTAAGTGC |  |
| Mus musculus *Egr1* | F | CACCTGACCACAGAGTCCTTTT | 121 |
|  | R | GGGAGAAGCGGCCAGTATAG |  |
| Mus musculus *Rest* | F | CGGTGTAAGCCTTGCCAGTA | 228 |
|  | R | CAGGTGTGCCATGTAGTGGT |  |
| Mus musculus *Nr4a3* | F | CGCAGACTTATGGCTCGGAA | 184 |
|  | R | GGCATTTGGTACAGGCAGGA |  |
| Mus musculus *Nab2* | F | CAGAGGGGATAACACACGCC | 110 |
|  | R | GTTGCAGGACCCGATACAGC |  |
| Mus musculus *Pappa2* | F | GTGACCAGTTTGGACCGCTA | 183 |
|  | R | CATCTGCTGCACTTGGATGC |  |
| Mus musculus *Gpx6* | F | TCAACGTGGCATCCTTCTGT | 100 |
|  | R | GGAAAGCCCAAAACCGTGAC |  |
| Mus musculus *Snca* | F | GCAAGGGTGAGGAGGGGTA | 90 |
|  | R | CCTCTGAAGGCATTTCATAAGCC |  |
| Homo sapiens *Cacna1h* | F | GTACTCGTTGGACGGACACA | 161 |
|  | R | AGAAGCACAGCAGAAGGACG |  |
| Homo sapiens *C3* | F | ATTTGCGAGGAGCAGGTCAA | 192 |
|  | R | CCAGCGGTTCTTATCTTTGGC |  |
| Homo sapiens *SNCA* | F | CAACAGTGGCTGAGAAGACCA | 163 |
|  | R | GCTCCTTCTTCATTCTTGCCCA |  |
| Homo sapiens *RPL13A* | F | CCTGGAGGAGAAGAGGAAAGAGA | 126 |
|  | R | TTGAGGACCTCTGTGTATTTGTCAA |  |

**Table S4.** List of antibodies used in the study.

| Antigen | Supplier | Product number | Dilution used in WB | Dilution used in IF |
| --- | --- | --- | --- | --- |
| α-synuclein (C-20) | Santa-Cruz | sc-7011-R | 1:1000 | 1:1000 |
| α-synuclein (Syn1) | BD Transductions | 610787 | 1:1000 | 1:500 |
| phospho-a-synuclein | Abcam | ab51253 | 1:1000 | N/A |
| Tyrosine hydroxylase | Millipore | MAB318 | 1:1000 | 1:2000 |
| Synaptobrevin-2 | Synaptic Systems | 104111 | 1:5000 | N/A |
| GAPDH | Santa Cruz | sc-365062 | 1:4000 | N/A |
| Ca_v_1.2 | GeneTex | GTX54754 | N/A | 1:250 |
| Ca_v_2.1 | GeneTex | GTX5475 | N/A | 1:500 |
| Ca_v_2.2 | GeneTex | GTX54812 | N/A | 1:500 |
| Ca_v_3.1 | Abcam | ab134269 | N/A | 1:500 |
| Ca_v_3.2 | GeneTex | GTX54813 | 1:1000 | 1:500 |
|  | StressMarq Biosciences | SMC-303 | 1:500 | 1:500 |
|  | Alomone Labs | ACC-025 | 1:500 | 1:500 |
| Ca_v_3.3 | Saint John's Laboratory | STJ97556 | N/A | 1:500 |
| β-actin | Sigma | A1978 | 1:5000 | N/A |
| GFAP | Invitrogen | PA3-16727 | 1:4000 | N/A |
|  | DAKO | Z0334 | N/A | 1:1000 |
|  | Cell Signaling | 3670 | N/A | 1:750 |
| ALDH1L1 | Abcam | ab87117 | N/A | 1:500 |
| IBA1 | Millipore | MABN92 | N/A | 1:750 |
|  | Abcam | Ab108539 | 1:500 | 1:750 |
| phospho-p65-NF-κB | Cell signaling | 3033 | 1:500 | 1:400 |
| p65-NF-κB | Cell signaling | 6956 | 1:500 | 1:800 |
| phospho-STAT3 | Cell signaling | 9145 | 1:500 | N/A |
| STAT3 | Santa Cruz | sc-482 | 1:1000 | N/A |
| NFL | Saint John's Laboratory | STJ96461 | 1:1000 | N/A |
| phospho-CREB | Cell signaling | 9196S | 1:500 | N/A |
| CREB/ATF1 | Cell signaling | 9197T | 1:500 | N/A |
| phospho-IkBα | Cell signaling | 2859T | 1:500 | N/A |
| IkBα | Cell signaling | 4814T | 1:500 | N/A |
| phospho-p38-MAPK (Thr180/Thr182) | Cell signaling | 9913 | 1:500 | 1:500 |
| P38-MAPK | Santa Cruz | sc-535 | 1:500 | N/A |
| phospho-MSK1(Thr581) | Cell signaling | 9595 | 1:500 | N/A |
| phospho-ATF-2 (Thr71)/ATF-7(Thr53) | Cell signaling | 15411 | 1:500 | 1:500 |
| ATF2/7 | Cell signaling | 35031T | 1:500 | N/A |
| phospho-MKK3 (Ser189) / MKK6 (Ser207) | Cell signaling | 12280 | 1:500 | N/A |
| A20/TNFAIP3 | Cell signaling | 5630T | 1:500 | 1:500 |
| phospho-JNK | Santa Cruz | sc-6254 | 1:1000 | N/A |
| phospho-HSP27 | Cell signaling | 9709T | 1:500 | N/A |
| JNK2 | Santa Cruz | sc827 | 1:500 | N/A |
| C3a | Bioss | bs-2934R-TR | 1:1000 | 1:200 |
|  | Genetex | GTX101316 | N/A | 1:500 |
| C1qA | Proteintech | 11602-1-AP | N/A | 1:150 |
| p62 | Cell signaling | 2314S | N/A | 1:1000 |
| MYLK | Santa Cruz | sc515020 | N/A | 1:100 |
| IGFBPL1 | Santa Cruz | sc398875 | 1:500 | 1:200 |
| PACAP | Santa Cruz | sc166180 | N/A | 1:150 |
| TNFα | Peprotech | 500-P64-EDK | N/A | 1:200 |
| β3-Tubulin | Cell signaling | 5568 | N/A | 1:500 |
|  | Cell signaling | 4466 | N/A | 1:250 |
| cd11b | BIO-RAD | MCA74GA | N/A | 1:250 |
| cd68 | BIO-RAD | MCA1957GA | N/A | 1:250 |
| PSD95 | Cell signaling | 3450S | 1:1000 | N/A |
| Glt-1/EAAT2 | Cell signaling | 3838S | 1:1000 | N/A |
| DBH | Bioss | bs-0596R | 1:500 | N/A |
| ADCYAP1R1 (PAC1) | Saint John's Laboratory | STJ115798 | N/A | 1:200 |

N/A: non-applicable
