# Supplementary material for: α-Synuclein oligomers potentiate neuroinflammatory NF-κB activity and induce Cav3.2 calcium signaling in astrocytes
Source: Transl Neurodegener. 2024 Feb 21;13:11. doi: 10.1186/s40035-024-00401-4 (PMC10880263; doi:10.1186/s40035-024-00401-4)
Supplement: Supplementary file 2 — Additional file 2: DEGs up- and down-regulated in WT and A53T Tg mice. Analysis of RNAseq data. Differentially up- and down-regulated genes in the striatum of WT and A53T Tg mice. [file 40035_2024_401_MOESM2_ESM.pdf]

down-regulated DEGs

baseMean	log2FoldChange	lfcSE	stat	pvalue	padj	
**Trpm1**	26.01413855	-5.480084	1.321283238	-4.147547027	3.36E-05	0.0030906
**Sh3bp2**	161.5910599	-4.249636	0.483734898	-8.785051176	1.56E-18	3.32E-15
**Sp7**	140.0131634	-4.090614	0.488456413	-8.374573424	5.54E-17	9.15E-14
**Wnt8b**	31.06718495	-3.006554	0.868505559	-3.461755373	0.0005367	0.0230425
**Sh2b2**	108.2812779	-2.914076	0.476648505	-6.113678445	9.74E-10	5.56E-07
**Gpr21**	107.7501641	-2.747877	0.517401586	-5.310916831	1.09E-07	2.66E-05
**Scarf2**	78.46713512	-2.590187	0.485232252	-5.338035568	9.40E-08	2.33E-05
**Sh3tc1**	58.89719437	-2.404253	0.535041507	-4.493583023	7.00E-06	0.0008743
**Gpx6**	96.29556878	-2.342897	0.585948397	-3.998470498	6.38E-05	0.0050648
**Fgf2**	285.6072638	-2.323163	0.397644695	-5.842309084	5.15E-09	2.07E-06
**Slc7a5**	1264.564786	-2.319142	0.222581525	-10.41929059	2.02E-25	7.52E-22
**Myo7a**	208.2106134	-2.297077	0.414322676	-5.544173409	2.95E-08	8.78E-06
**Gjc1**	111.6021574	-2.262136	0.485067179	-4.663552049	3.11E-06	0.0004664
**Wdfy4**	69.43674913	-2.236139	0.498232456	-4.48814387	7.18E-06	0.0008821
**Asah2**	418.9547835	-2.226425	0.238662725	-9.328752476	1.07E-20	3.18E-17
**Wdhd1**	161.9355362	-2.091096	0.703379767	-2.972925817	0.0029498	0.0758304
**Sinhcaf**	90.76759901	-2.072553	0.623441849	-3.324373199	0.0008862	0.0326675
**Nxf7**	37.89791868	-2.053966	0.69626648	-2.949971617	0.003178	0.0793493
**Efcab5**	60.44059348	-2.039515	0.659724637	-3.091464776	0.0019917	0.0584762
**Primpol**	139.4726611	-2.008304	0.556451031	-3.609130356	0.0003072	0.0156843
**Aim2**	107.9234756	-2.006427	0.61686487	-3.252619474	0.0011435	0.0393225
**Rpe65**	60.96648154	-1.990683	0.537121425	-3.706206398	0.0002104	0.0120071
**Mad2l1**	214.8621921	-1.95225	0.337402034	-5.786123526	7.20E-09	2.55E-06
**Mecom**	66.95342935	-1.940169	0.497246895	-3.901823028	9.55E-05	0.0067219
**Lrrc32**	115.325776	-1.872741	0.492386848	-3.803393543	0.0001427	0.0087982
**Col6a4**	152.7759068	-1.828139	0.397708377	-4.596682165	4.29E-06	0.0005851
**Slc26a10**	269.8554749	-1.823146	0.413354949	-4.410605266	1.03E-05	0.001178
**Dennd3**	101.8917136	-1.660749	0.568831669	-2.919578914	0.003505	0.0846682
**Npas4**	422.8349899	-1.559508	0.26372631	-5.913357493	3.35E-09	1.51E-06
**Slc4a11**	124.8194914	-1.497601	0.432936434	-3.459171633	0.0005418	0.0231976
**Per2**	1063.54716	-1.449582	0.180045193	-8.051209394	8.20E-16	1.11E-12
**Mroh7**	140.3082992	-1.445721	0.371029092	-3.89651742	9.76E-05	0.0067745
**Crybg3**	683.1376367	-1.409763	0.23966677	-5.882179164	4.05E-09	1.67E-06
**Papln**	120.8585202	-1.398141	0.400505002	-3.490945988	0.0004813	0.0213445
**Fam117a**	168.5011942	-1.364696	0.340841444	-4.003901808	6.23E-05	0.0049823
**Egr4**	1365.895291	-1.356837	0.228629294	-5.934657664	2.94E-09	1.41E-06
**Nrros**	115.2705979	-1.352037	0.386096056	-3.501814181	0.0004621	0.0206781
**Dmkn**	230.619134	-1.345443	0.366577115	-3.67028746	0.0002423	0.0133307
**Stk26**	711.1439498	-1.318012	0.22748283	-5.793896966	6.88E-09	2.55E-06
**Map3k1**	273.675819	-1.317557	0.327435207	-4.023872113	5.72E-05	0.004725
**Casp8ap2**	404.1693947	-1.303726	0.458722901	-2.842077536	0.0044821	0.0990855
**Nexn**	1401.224201	-1.297711	0.17121014	-7.579639041	3.47E-14	4.29E-11
**Cnot6l**	1154.254713	-1.283569	0.323229261	-3.971080341	7.15E-05	0.0054783
**Scube3**	645.7510004	-1.281806	0.286057602	-4.480938063	7.43E-06	0.0009049
**Tacr3**	159.8175453	-1.276207	0.390816649	-3.26548898	0.0010928	0.0381975
**Nsun6**	133.5876816	-1.271035	0.362054159	-3.510621583	0.0004471	0.0203728

Up-regulated DEGs

baseMean	log2FoldChange	lfcSE	stat	pvalue	padj	
**Mylk3**	43.20092346	4.3868356	1.18303499	3.708119907	0.000208804	0.0119768
**Shox2**	19.99307268	4.3483662	1.41906394	3.064249628	0.002182167	0.0617491
**Pappa2**	20.07707275	4.3269335	1.26009978	3.43380228	0.000595178	0.0247754
**Prnp**	76981.57491	3.9796046	0.13608067	29.24445142	5.28E-188	7.85E-184
**Prom2**	27.67704219	3.8029756	1.23460243	3.080324052	0.002067755	0.0597272
**Adcyap1**	171.4871274	3.4642549	0.74989808	4.619634267	3.84E-06	0.0005388
**Npsr1**	22.99728544	3.168092	1.06094069	2.986115975	0.002825455	0.0735779
**Snca**	12477.44334	3.0002843	0.15231995	19.69725081	2.28E-86	1.69E-82
**Neurod1**	58.40333333	2.9824479	0.75115442	3.970485696	7.17E-05	0.0054783
**Tspan11**	92.71838078	2.8946039	0.89588845	3.230986955	0.001233636	0.041938
**Hkdc1**	131.4911391	2.8627857	0.84081618	3.404770045	0.000662198	0.0269271
**Trim7**	63.16171903	2.8258949	0.57874815	4.882771381	1.05E-06	0.0001919
**Impg2**	27.34604466	2.7699987	0.8875794	3.120846132	0.001803322	0.0541215
**Fam19a1**	227.6961469	2.5653612	0.65816401	3.89775372	9.71E-05	0.0067716
**Met**	90.81755012	2.486467	0.8581749	2.897389619	0.003762821	0.0892191
**Prdm8**	153.2796276	2.4748653	0.51937473	4.765085983	1.89E-06	0.0003082
**C1qtnf1**	64.02895771	2.4714686	0.73051572	3.383183291	0.000716508	0.0281599
**Vip**	246.6872659	2.4679816	0.41281376	5.978438454	2.25E-09	1.12E-06
**Cenpa**	40.26770623	2.4288921	0.70736567	3.43371503	0.00059537	0.0247754
**Podn**	69.4100887	2.3945592	0.81478237	2.938894225	0.003293855	0.0810157
**Emx1**	167.4255044	2.374673	0.61695488	3.849022226	0.00011859	0.0076933
**mt-Co2**	8325.562003	2.3614367	0.19727322	11.97038692	5.08E-33	2.52E-29
**Rps28**	386.6420421	2.3022405	0.26032696	8.843650166	9.26E-19	2.29E-15
**Col12a1**	189.9482988	2.2935473	0.62654175	3.66064556	0.000251581	0.0136503
**Adamtsl2**	94.7942534	2.2610936	0.60624862	3.729647369	0.000191748	0.0110841
**Smoc2**	84.10586503	2.214574	0.55266453	4.007085395	6.15E-05	0.0049632
**Doc2a**	298.1551397	2.2046055	0.49930555	4.41534338	1.01E-05	0.0011705
**Etnk2**	70.7551162	1.9940091	0.65957582	3.023168937	0.002501426	0.0678124
**Cck**	2016.776002	1.9617042	0.28923175	6.782464978	1.18E-11	1.03E-08
**Rtn4r**	629.120907	1.9448194	0.45134967	4.308897386	1.64E-05	0.0016581
**Bhlhe22**	247.3705934	1.9208954	0.43392205	4.426821445	9.56E-06	0.0011187
**Neurod2**	634.6375267	1.9178412	0.43900054	4.368653377	1.25E-05	0.0013361
**P4ha3**	129.3513054	1.9166323	0.39577207	4.842767855	1.28E-06	0.0002281
**Nwd2**	392.759076	1.909466	0.34980316	5.458687043	4.80E-08	1.34E-05
**Pgk1-rs7**	1249.890437	1.9077539	0.29870347	6.386781927	1.69E-10	1.14E-07
**Crh**	74.9649257	1.900171	0.58147682	3.267836138	0.001083731	0.0380612
**Glt8d2**	91.81272225	1.8777454	0.61172679	3.069581829	0.002143587	0.0608893
**Wnt9a**	170.7431089	1.8731827	0.59653113	3.140125656	0.001688754	0.0516217
**Neurod6**	565.3212568	1.8621682	0.31584852	5.895763593	3.73E-09	1.58E-06
**Scube1**	357.1724709	1.857907	0.5014941	3.704743508	0.000211605	0.0120071
**Cplx3**	320.3250001	1.8357945	0.51703638	3.550609873	0.00038434	0.0182129
**Ipcef1**	1141.520206	1.8229529	0.36983935	4.929039942	8.26E-07	0.0001554
**Trabd2b**	55.48203132	1.8086157	0.58763025	3.077812495	0.002085261	0.0599723
**Prss12**	243.4998377	1.8079994	0.31046661	5.823490791	5.76E-09	2.25E-06
**Rtn4rl2**	239.8930932	1.8053229	0.45015301	4.01046495	6.06E-05	0.0049195
**Itga8**	61.23145621	1.798886	0.62149074	2.894469596	0.003797997	0.0894185

**Rem2**	919.1096817	-1.266386	0.273291621	-4.633826069	3.59E-06	0.0005128
**Soat1**	685.2675299	-1.259172	0.308876403	-4.076620989	4.57E-05	0.0039014
**Baiap3**	279.4396373	-1.221092	0.359229979	-3.399194284	0.0006758	0.0269679
**Junb**	1594.717067	-1.211106	0.295923418	-4.092632288	4.27E-05	0.0037053
**N4bp2**	231.6855643	-1.205439	0.404707824	-2.978541409	0.0028962	0.0749591
**Gpr101**	123.3069286	-1.203779	0.379634929	-3.170885013	0.0015198	0.0479351
**Cobll1**	303.7857625	-1.187098	0.275121976	-4.314806212	1.60E-05	0.0016366
**Klf4**	116.1728548	-1.183569	0.400764476	-2.953278622	0.0031442	0.0788303
**Ciart**	355.6693068	-1.18319	0.351343004	-3.36762006	0.0007582	0.0289705
**Rpl10-ps1**	895.8486918	-1.182709	0.386759764	-3.057994639	0.0022282	0.0625759
**Nr4a3**	831.81361	-1.180273	0.374435684	-3.15213793	0.0016208	0.0500594
**Rasgrp2**	3936.764022	-1.163039	0.19263825	-6.037424806	1.57E-09	8.02E-07
**Tiparp**	587.5268487	-1.155575	0.31634595	-3.652884535	0.0002593	0.0139052
**Plekha1**	2350.44051	-1.144839	0.283828311	-4.033561385	5.49E-05	0.0045851
**Zbtb20**	1797.422866	-1.134103	0.247614139	-4.580122282	4.65E-06	0.000627
**Abhd11os**	170.2708343	-1.121169	0.32547333	-3.444735067	0.0005716	0.0239886
**Fosb**	393.3881123	-1.120501	0.312991429	-3.579972953	0.0003436	0.0171308
**Dlk1**	208.8423726	-1.120421	0.374154082	-2.99454499	0.0027485	0.0725264
**Krt9**	512.410962	-1.112487	0.295370022	-3.766419508	0.0001656	0.0099352
**Rnf207**	233.8106984	-1.104237	0.308999407	-3.57358979	0.0003521	0.0173345
**Arhgap27**	662.1238689	-1.09211	0.30622401	-3.566377524	0.000362	0.0175723
**Rnf169**	864.5000564	-1.090165	0.188213711	-5.792167038	6.95E-09	2.55E-06
**Id3**	506.8664264	-1.087907	0.320499204	-3.394415135	0.0006878	0.0271736
**Prrg1**	201.0972161	-1.077406	0.373025059	-2.888294028	0.0038734	0.0903342
**Actn2**	1909.082982	-1.075441	0.206168232	-5.216327214	1.83E-07	4.24E-05
**Crlf3**	287.7537301	-1.075434	0.282541794	-3.806282809	0.0001411	0.0087443
**Fos**	365.2555633	-1.072119	0.333097652	-3.218633408	0.001288	0.0431847
**Rpl22l1**	560.0749056	-1.066078	0.268073667	-3.976811194	6.98E-05	0.0054564
**Unc13c**	3573.792053	-1.05649	0.187176611	-5.64434829	1.66E-08	5.35E-06
**Flna**	1705.745403	-1.046399	0.20051898	-5.21845343	1.80E-07	4.24E-05
**Pard3**	981.9393925	-1.044298	0.217248042	-4.806936979	1.53E-06	0.0002617
**Mrpl48**	920.4804927	-1.026479	0.205823029	-4.987191085	6.13E-07	0.0001198
**St8sia2**	251.4513706	-1.005189	0.308872237	-3.254383466	0.0011364	0.0391698
**Nab2**	840.0560451	-1.001889	0.218828678	-4.578416424	4.69E-06	0.000627
**Kcnh4**	808.1907875	-0.999947	0.192959113	-5.182168251	2.19E-07	4.94E-05
**Arl4d**	799.0975031	-0.996055	0.290803149	-3.425185189	0.0006144	0.0253601
**Gcnt2**	1834.997854	-0.994984	0.186359929	-5.339043516	9.34E-08	2.33E-05
**Rgs2**	2425.465417	-0.994854	0.225782214	-4.406253114	1.05E-05	0.0011927
**Slc5a7**	699.5674931	-0.986523	0.250208192	-3.942807297	8.05E-05	0.0058788
**Fosl2**	913.2521027	-0.983949	0.340964288	-2.885782075	0.0039044	0.0906269
**Dcun1d3**	388.7603797	-0.977677	0.281060402	-3.478528783	0.0005042	0.0220295
**Tchp**	223.0761832	-0.967201	0.292943932	-3.301658318	0.0009612	0.0345735
**Adgre5**	221.4854319	-0.966823	0.323026373	-2.993014545	0.0027624	0.0727619
**Ido1**	358.8411948	-0.965279	0.28549168	-3.381110622	0.0007219	0.0282456
**Svil**	323.3658459	-0.960553	0.255757137	-3.7557232	0.0001728	0.01023
**Rnpc3**	522.1988468	-0.943865	0.295422432	-3.19496812	0.0013985	0.045646
**Sgk1**	3364.293141	-0.929249	0.283347304	-3.279542222	0.0010398	0.0367777
**Ice2**	182.5675311	-0.925227	0.315973677	-2.928177797	0.0034096	0.0831729

**Wnt10a**	81.33880471	1.7957269	0.46285881	3.879642933	0.00010461	0.0071617
**Rpl17**	433.3812855	1.7774373	0.39459104	4.504504965	6.65E-06	0.0008376
**Kcnj6**	134.8446486	1.7749424	0.48640559	3.649099513	0.000263161	0.0139626
**Ccn3**	465.6765461	1.6964004	0.35311338	4.80412388	1.55E-06	0.0002624
**Cacna1g**	814.8240227	1.6933859	0.27881552	6.073499233	1.25E-09	6.89E-07
**Cfap100**	75.73018607	1.687082	0.5563693	3.032306161	0.002426929	0.0663986
**Cnih3**	734.6161267	1.6817732	0.39423698	4.265894112	1.99E-05	0.0019589
**Medag**	263.7274886	1.6795389	0.44003581	3.816823123	0.000135181	0.0085457
**Gfra2**	702.8954727	1.6664297	0.36823483	4.525453786	6.03E-06	0.0007785
**C1ql3**	437.2490212	1.6586406	0.2986891	5.553067106	2.81E-08	8.51E-06
**Pdzrn3**	310.8140862	1.6527259	0.41875512	3.946759792	7.92E-05	0.0058788
**Galnt9**	812.5892275	1.6459065	0.38543302	4.27027892	1.95E-05	0.0019335
**Cbln2**	254.1057064	1.6359832	0.38195062	4.283232143	1.84E-05	0.0018365
**Zbtb37**	431.0688199	1.63551	0.270791	6.039750082	1.54E-09	8.02E-07
**Pde5a**	145.1080454	1.5805509	0.39754579	3.975770702	7.02E-05	0.0054564
**Nxph3**	364.1006907	1.5607985	0.27842852	5.60574239	2.07E-08	6.55E-06
**Bdnf**	203.3273386	1.5397973	0.40457714	3.805942375	0.000141265	0.0087443
**Mical2**	3688.349722	1.5336116	0.22312888	6.873209986	6.28E-12	5.83E-09
**Dtl**	87.14610102	1.497002	0.46822509	3.197184516	0.001387761	0.045411
**Car4**	328.5812015	1.4938781	0.36051784	4.143700924	3.42E-05	0.0031147
**Pter**	773.9207436	1.4912462	0.27390905	5.444311778	5.20E-08	1.43E-05
**Nptxr**	6561.775327	1.4660293	0.27212978	5.387243026	7.15E-08	1.90E-05
**Mapk11**	440.1976115	1.4532336	0.33384717	4.35299049	1.34E-05	0.0014149
**Oxtr**	65.98594372	1.4506364	0.50404471	2.877991502	0.004002159	0.0917532
**Cux2**	697.0628782	1.4470256	0.37858339	3.822211056	0.00013226	0.0084329
**Tuba8**	137.6287231	1.4447639	0.41037285	3.520612849	0.000430551	0.0198027
**Olfm1**	8233.113239	1.4375827	0.26686882	5.386851442	7.17E-08	1.90E-05
**Lrfn2**	216.0363917	1.4335228	0.3485416	4.112917455	3.91E-05	0.0034611
**Bmp3**	179.0094319	1.432122	0.3802722	3.766044376	0.000165854	0.0099352
**Bag2**	100.2204161	1.4311849	0.44367361	3.225760659	0.001256384	0.0424201
**Rspo2**	118.4452059	1.4201432	0.41794055	3.397954924	0.000678916	0.0269679
**Syt13**	2474.149385	1.3679773	0.31790999	4.303033414	1.68E-05	0.0016911
**Ccn2**	587.8929461	1.3489091	0.29276919	4.607414747	4.08E-06	0.000564
**Tmem178**	669.1903567	1.347111	0.40006545	3.367226549	0.000759283	0.0289705
**Relt**	282.1854217	1.3471107	0.37302967	3.611269469	0.000304702	0.0156632
**Ccdc3**	118.2965967	1.3468864	0.4049417	3.32612419	0.000880627	0.0325438
**Adra1b**	162.0535624	1.3454561	0.46600318	2.887225079	0.003886561	0.0904996
**Nxph4**	97.98116502	1.3343353	0.44086381	3.026638299	0.002472897	0.0672845
**Nptx1**	3719.717582	1.3247036	0.28985029	4.570302924	4.87E-06	0.000646
**Zbtb16**	190.4999555	1.3212517	0.37730548	3.501808968	0.000462111	0.0206781
**Fst**	164.6026568	1.317999	0.35190207	3.745357257	0.000180137	0.0105182
**Ptpn3**	472.9036516	1.317881	0.39068274	3.373276747	0.000742793	0.0287368
**Kcnc4**	637.0696663	1.3150926	0.36855192	3.568269713	0.000359346	0.0175554
**Sema6c**	272.8717171	1.3137425	0.44450249	2.955534691	0.003121276	0.0784772
**Ak5**	1563.544926	1.3100018	0.27058531	4.841363461	1.29E-06	0.0002281
**Fam81a**	2053.614345	1.3065094	0.27093797	4.822171678	1.42E-06	0.0002482
**Fkbp1b**	238.2494771	1.2943929	0.39519982	3.275287092	0.001055546	0.0371592
**Rprm**	400.2048742	1.2940137	0.43103625	3.002099488	0.002681245	0.0716413

**Sh2b3**	542.2713616	-0.920826	0.210248795	-4.37969843	1.19E-05	0.0012887
**Rarb**	1645.37905	-0.918824	0.241803559	-3.799877634	0.0001448	0.0088871
**Nol8**	622.5805245	-0.911064	0.300512736	-3.0316989	0.0024318	0.06641
**Chat**	431.5039934	-0.910044	0.232408434	-3.915707796	9.01E-05	0.0065005
**Fam241a**	195.7349711	-0.905906	0.319289188	-2.837259483	0.0045503	0.0996582
**Pign**	402.8372281	-0.89749	0.238665626	-3.760450431	0.0001696	0.0100788
**Lmo2**	907.6837207	-0.897076	0.20215557	-4.437552674	9.10E-06	0.0010728
**Clk1**	2920.040509	-0.891518	0.267612454	-3.331378835	0.0008642	0.0320152
**Rpn2**	2683.000537	-0.882881	0.178420122	-4.948326628	7.49E-07	0.0001426
**Isyna1**	950.9164658	-0.879924	0.22142595	-3.973897891	7.07E-05	0.0054709
**Lrrc10b**	5345.966673	-0.877165	0.245519379	-3.572691565	0.0003533	0.0173345
**Rxrg**	2285.040853	-0.877029	0.286518602	-3.060983488	0.0022061	0.062072
**Amy1**	359.4375884	-0.868366	0.302979093	-2.866093243	0.0041557	0.0939568
**Zfp276**	504.1487419	-0.855686	0.25335531	-3.377413326	0.0007317	0.0284562
**Zc3h12c**	421.2890329	-0.854281	0.258033282	-3.310741259	0.0009305	0.033798
**Cacna1h**	2017.745116	-0.852767	0.159625694	-5.34229437	9.18E-08	2.33E-05
**Slc10a4**	332.4514897	-0.845867	0.274579563	-3.08059144	0.0020659	0.0597272
**Per1**	2306.436439	-0.835847	0.163923062	-5.09902061	3.41E-07	7.25E-05
**Fam184b**	462.7800331	-0.834232	0.274455678	-3.039588166	0.002369	0.0655353
**Dnajc1**	514.9679179	-0.829778	0.248035257	-3.345405104	0.0008216	0.0309798
**Grb10**	976.1692671	-0.827122	0.277014806	-2.98583893	0.002828	0.0735779
**Drd1**	4597.265595	-0.825979	0.185641929	-4.44931097	8.61E-06	0.0010238
**Tpm1**	5889.561583	-0.825437	0.226978466	-3.636629853	0.0002762	0.0145431
**Dbp**	3533.927983	-0.825097	0.187023959	-4.411717722	1.03E-05	0.001178
**Flnb**	941.1924193	-0.822611	0.245453927	-3.351387721	0.0008041	0.0304729
**Slc17a8**	319.4345436	-0.821447	0.289344587	-2.838991024	0.0045256	0.0996582
**Bhlhe41**	1197.230487	-0.8211	0.188466652	-4.356737226	1.32E-05	0.0014009
**Clk4**	1469.267559	-0.813301	0.27630439	-2.943495173	0.0032453	0.0800732
**Scn3a**	1340.769115	-0.804909	0.231861893	-3.471502554	0.0005176	0.0224163
**Slc25a25**	1581.507141	-0.804142	0.183130536	-4.391087042	1.13E-05	0.0012504
**Serpinb1a**	310.676419	-0.801399	0.273521476	-2.929930236	0.0033904	0.0828413
**Cxadr**	609.3598815	-0.801121	0.219408737	-3.651271216	0.0002609	0.0139052
**Anapc7**	679.5084974	-0.800299	0.211115443	-3.790812271	0.0001502	0.0091799
**mt-Atp8**	91030.01301	-0.79458	0.134083976	-5.92598978	3.10E-09	1.44E-06
**Cd4**	1381.961948	-0.7921	0.209464585	-3.781547759	0.0001559	0.0094506
**Hlcs**	396.5823861	-0.787719	0.236434132	-3.331664277	0.0008633	0.0320152
**Egr1**	7081.129901	-0.785498	0.190863517	-4.115497357	3.86E-05	0.0034611
**Ddit4l**	598.6972096	-0.785337	0.254306602	-3.088149051	0.0020141	0.0588998
**Ccdc187**	583.2519508	-0.784783	0.232939739	-3.369039098	0.0007543	0.028956
**Kcna4**	1260.996273	-0.783914	0.186502357	-4.203236865	2.63E-05	0.0025058
**Stxbp2**	860.906844	-0.780004	0.231453234	-3.37002988	0.0007516	0.0289269
**Plekha6**	2237.088492	-0.777997	0.203411269	-3.824751018	0.0001309	0.0083824
**Arpp21**	25339.35434	-0.773719	0.225939956	-3.424443671	0.0006161	0.0253601
**Gprin3**	1058.636802	-0.771686	0.21434255	-3.600246592	0.0003179	0.01601
**Reln**	2134.026012	-0.771517	0.260406439	-2.962743005	0.0030491	0.0775644
**Scn4b**	26940.9743	-0.771366	0.164286295	-4.69525563	2.66E-06	0.0004036
**Dgkb**	8413.271375	-0.766088	0.211159041	-3.628014701	0.0002856	0.014784
**Ano2**	537.4359896	-0.765463	0.216404816	-3.537180665	0.0004044	0.0187753

**Pafah2**	127.6936797	1.2939353	0.37938416	3.41062013	0.000648153	0.0264532
**Stx1a**	2020.370326	1.2837879	0.33673441	3.812464246	0.000137588	0.0086429
**Unc5d**	417.005264	1.2811316	0.39920364	3.209218126	0.001330965	0.0440374
**Lsm11**	283.0932071	1.2762244	0.38135714	3.34653325	0.000818289	0.0309326
**St8sia5**	582.093361	1.2737776	0.33068461	3.851941073	0.000117185	0.0076933
**Sowahb**	216.5973395	1.2707674	0.41745164	3.044106837	0.002333722	0.0648033
**Fah**	468.8811649	1.2676314	0.24975055	5.075590111	3.86E-07	8.08E-05
**Satb1**	1298.153295	1.2455023	0.34273436	3.634016551	0.000279043	0.0145455
**Nectin3**	340.6036164	1.2420155	0.38100388	3.259850116	0.001114711	0.0387825
**Zfp7**	145.1354501	1.2386621	0.37543665	3.29925741	0.00096941	0.0346174
**Gabbr2**	4050.683614	1.236849	0.27297838	4.530941174	5.87E-06	0.000772
**Syt17**	276.124775	1.23661	0.33380731	3.704562286	0.000211756	0.0120071
**Mpped1**	2401.977908	1.2311569	0.22547588	5.460259852	4.75E-08	1.34E-05
**Trp53i11**	874.7906614	1.231117	0.19373272	6.35471918	2.09E-10	1.35E-07
**Chrna4**	601.7151303	1.2309176	0.26104427	4.715359531	2.41E-06	0.0003731
**Fezf2**	248.000773	1.2284661	0.39881206	3.080313222	0.00206783	0.0597272
**Shank1**	4013.176659	1.2248921	0.23505011	5.21119558	1.88E-07	4.29E-05
**Ccdc120**	197.4279328	1.2225014	0.39553927	3.09072064	0.001996714	0.0585073
**Cckbr**	397.85726	1.222062	0.34315098	3.561295334	0.00036903	0.0177997
**Tmem132d**	564.6574152	1.2155505	0.34344159	3.539322496	0.000401155	0.0187717
**Olfm2**	1171.83903	1.2115545	0.35477775	3.414967485	0.000637896	0.0261784
**Rpl23a**	910.5950333	1.2105546	0.23990811	5.045909654	4.51E-07	9.31E-05
**Adgra1**	632.8687975	1.208132	0.26770891	4.512856714	6.40E-06	0.0008121
**Vsnl1**	5499.953508	1.1995912	0.3250326	3.690679692	0.000223656	0.0124911
**Ncald**	5754.768234	1.1899023	0.31774839	3.744794142	0.000180542	0.0105182
**Sphkap**	1484.24865	1.1894522	0.32210152	3.692786858	0.00022181	0.0124347
**Kcnj16**	374.3816864	1.1867751	0.3005978	3.948049812	7.88E-05	0.0058788
**Pcsk5**	119.8914639	1.1863675	0.38562757	3.076459357	0.002094749	0.0600183
**Crb2**	137.9216577	1.1859859	0.39071519	3.035423122	0.002401985	0.0658374
**Gng4**	672.5231188	1.1787025	0.33307147	3.538887538	0.000401817	0.0187717
**Golga7b**	1516.263807	1.1731667	0.25469107	4.606234072	4.10E-06	0.000564
**Boc**	174.9406367	1.1677701	0.40112806	2.911215062	0.003600261	0.0866864
**Slitrk1**	1027.946439	1.1583162	0.34222667	3.384646236	0.0007127	0.0280846
**Nr2f1**	728.677164	1.1490076	0.37351853	3.076173045	0.002096761	0.0600183
**Zdhhc22**	550.8510457	1.1475903	0.29599779	3.877023014	0.000105742	0.0071731
**Coro2a**	405.9647289	1.1438964	0.327445	3.493400249	0.000476911	0.0212682
**Sstr2**	355.2684811	1.1376805	0.31845249	3.572528141	0.000353551	0.0173345
**Rps29**	909.2995026	1.1302194	0.21627584	5.225823825	1.73E-07	4.15E-05
**Nfix**	2630.961808	1.1296548	0.26964192	4.189462804	2.80E-05	0.0026126
**Pde1a**	1893.995877	1.1235785	0.24246515	4.633979507	3.59E-06	0.0005128
**Stac2**	1411.82806	1.1215463	0.35123329	3.193166396	0.001407218	0.0457454
**Grik4**	322.7630934	1.1136173	0.34408252	3.236483259	0.001210123	0.041423
**Sel1l3**	741.378788	1.1124569	0.35050339	3.173883463	0.001504141	0.0476352
**Disp3**	390.2301936	1.103195	0.28226351	3.908387044	9.29E-05	0.0066045
**B3galt2**	634.507636	1.0992941	0.29767161	3.692975822	0.000221645	0.0124347
**Kcnj3**	1348.866529	1.0903215	0.38106389	2.861256481	0.004219655	0.0948369
**Fxyd7**	524.1458476	1.0854456	0.2866902	3.786127335	0.000153013	0.0093163
**Efhd2**	2798.353822	1.0818534	0.26381618	4.100784635	4.12E-05	0.0036031

**Cdc42ep3**	626.9635923	-0.764151	0.257616186	-2.966239683	0.0030147	0.0769514
**Cd46**	607.557035	-0.763612	0.234004877	-3.263233075	0.0011015	0.0384125
**Tnrc6b**	1370.736977	-0.757375	0.256964353	-2.947393731	0.0032046	0.0797458
**Traip**	673.2487594	-0.751165	0.217427422	-3.454783192	0.0005507	0.0235107
**Tctn1**	652.6172749	-0.749416	0.240046573	-3.12196108	0.0017965	0.0540526
**Pigq**	1846.729333	-0.748945	0.168070012	-4.456147065	8.34E-06	0.0009997
**Aktip**	2565.030762	-0.747269	0.231082803	-3.23376946	0.0012217	0.0416268
**Suz12**	882.8076821	-0.746458	0.210004314	-3.554488922	0.0003787	0.0180326
**Rgs9**	8347.341181	-0.744625	0.206246635	-3.610361317	0.0003058	0.0156639
**Acvr1c**	1330.978476	-0.740669	0.192295685	-3.851721417	0.0001173	0.0076933
**Pfas**	462.3071311	-0.739189	0.260525606	-2.837297611	0.0045497	0.0996582
**Id4**	4319.355288	-0.739053	0.151881047	-4.86599913	1.14E-06	0.0002063
**Celsr3**	1417.353288	-0.737092	0.188984382	-3.900278394	9.61E-05	0.006733
**Atp6v0a2**	1856.261897	-0.735663	0.222926913	-3.300017398	0.0009668	0.0346174
**Agfg2**	1119.131502	-0.732588	0.189077447	-3.874538176	0.0001068	0.0072137
**Frmd4b**	893.0658954	-0.727074	0.221901171	-3.276565238	0.0010508	0.0370793
**Rrp1b**	400.6993132	-0.726424	0.246035422	-2.952517974	0.0031519	0.0788303
**Adgrf5**	840.339897	-0.722161	0.206762871	-3.492700176	0.0004782	0.0212682
**Fras1**	728.453312	-0.720297	0.224343797	-3.210682043	0.0013242	0.0439115
**Zeb1**	2113.898047	-0.719731	0.171355849	-4.200211989	2.67E-05	0.0025233
**Ndufa3**	835.8412508	-0.719476	0.186413567	-3.859571208	0.0001136	0.0076011
**Pbx3**	1073.562951	-0.718516	0.242496821	-2.962989822	0.0030467	0.0775644
**Cc2d1b**	606.1896195	-0.716544	0.210744575	-3.400059676	0.0006737	0.0269679
**Fbxl16**	37262.46892	-0.714676	0.145664235	-4.906324806	9.28E-07	0.0001723
**Tbc1d8**	2020.7631	-0.714116	0.230886377	-3.092931247	0.0019819	0.0583691
**Ccdc88c**	1994.987543	-0.712951	0.250942904	-2.841088022	0.004496	0.0992458
**Lbh**	825.8340003	-0.70949	0.199557692	-3.55531231	0.0003775	0.0180326
**Rap1gap**	10192.59407	-0.708897	0.191799491	-3.696032002	0.000219	0.0123703
**Mtcl1**	3040.10166	-0.70542	0.148842893	-4.739359939	2.14E-06	0.0003388
**Ccnd2**	1746.351354	-0.700793	0.176899601	-3.961530712	7.45E-05	0.0055876
**Sv2c**	2950.839111	-0.696705	0.170462794	-4.087135659	4.37E-05	0.0037503
**Tspyl2**	2161.044672	-0.694494	0.154685756	-4.489706113	7.13E-06	0.0008821
**Pcp4**	12455.10006	-0.692506	0.173741946	-3.985831053	6.72E-05	0.0053137
**Akap9**	3773.260012	-0.692065	0.239827431	-2.885680261	0.0039057	0.0906269
**Tmem267**	462.2202233	-0.691387	0.230566537	-2.998643333	0.0027118	0.0720169
**Necab2**	3170.949018	-0.691359	0.181512078	-3.80888533	0.0001396	0.0087135
**Ppp1r16b**	6722.853167	-0.691282	0.207232024	-3.335788342	0.0008506	0.0318292
**Ablim2**	5459.994512	-0.690511	0.220886374	-3.126093283	0.0017715	0.0535982
**Ankrd12**	1589.134285	-0.690049	0.225702799	-3.057336847	0.0022331	0.0625951
**Ppp1r1b**	33651.51189	-0.688475	0.182450945	-3.773480034	0.000161	0.009722
**Rragd**	4459.498419	-0.688427	0.211395518	-3.256580517	0.0011276	0.0390014
**Dach1**	1252.115715	-0.683216	0.170649499	-4.003623358	6.24E-05	0.0049823
**Shb**	692.872406	-0.681433	0.239578499	-2.844301258	0.0044509	0.0986903
**Thsd7a**	1777.022511	-0.680793	0.162428687	-4.191337562	2.77E-05	0.0026075
**Srrt**	2688.126991	-0.674331	0.200046136	-3.370876717	0.0007493	0.028913
**mt-Nd4l**	45790.07166	-0.674152	0.14284243	-4.719548133	2.36E-06	0.0003696
**Slc9a5**	1222.62655	-0.670319	0.236294039	-2.836801766	0.0045568	0.0996582
**Selenoi**	1572.114882	-0.657087	0.188703776	-3.482108158	0.0004975	0.0218012
**Hs3st2**	680.3856766	1.0801797	0.29556627	3.654610968	0.000257572	0.0138641
**Slc18b1**	368.9948548	1.079639	0.31730548	3.402522375	0.000667669	0.0269679
**Sez6l**	1808.944502	1.076292	0.33439797	3.218596054	0.001288198	0.0431847
**Serpini1**	2971.700407	1.0710134	0.30411138	3.521780063	0.00042866	0.0197769
**Fam19a2**	406.8139867	1.068541	0.34752319	3.074732964	0.002106911	0.0601928
**Slc6a7**	639.2446564	1.0659044	0.2981838	3.574655607	0.000350689	0.0173345
**Chrm3**	573.9069233	1.0537006	0.2812642	3.746302032	0.000179461	0.0105182
**Cd34**	833.7185128	1.0516972	0.33396047	3.149166791	0.001637367	0.0503618
**Rai14**	200.4367545	1.0469058	0.36659063	2.855789898	0.004292991	0.0963394
**Tanc1**	597.7948524	1.0457913	0.34661379	3.017166	0.0025515	0.0690439
**Ndst3**	208.1501437	1.0317532	0.33283123	3.099929023	0.00193567	0.0573429
**Ak4**	524.1205479	1.0309487	0.22261384	4.631107731	3.64E-06	0.0005146
**Ank1**	1001.304858	1.0287663	0.28930608	3.555978723	0.000376575	0.0180326
**Rnasel**	149.1951559	1.0283024	0.35572015	2.89076246	0.003843085	0.0899347
**Gprin1**	1189.89355	1.0257347	0.23416476	4.380397433	1.18E-05	0.0012887
**Arhgef25**	1769.695747	1.0150554	0.2333748	4.349464639	1.36E-05	0.0014178
**Unc13b**	511.5853964	1.0136361	0.21373708	4.742443553	2.11E-06	0.0003388
**Lingo1**	3725.618694	1.0134609	0.28353742	3.574346231	0.000351104	0.0173345
**Wnt4**	241.657262	1.0116059	0.32403647	3.121888901	0.001796947	0.0540526
**Fxyd6**	860.000222	1.0067659	0.21645145	4.651231931	3.30E-06	0.0004902
**Hrh1**	174.3276249	1.0036826	0.3457685	2.902758796	0.003698914	0.0883458
**Kcnj9**	1696.95976	1.0004493	0.30170629	3.315970911	0.000913253	0.0334017
**Ptprk**	684.0693408	1.0001591	0.28035382	3.567488781	0.000360419	0.0175554
**Skida1**	181.6241128	0.9971895	0.33107201	3.012001758	0.002595311	0.0698477
**Gpd2**	1959.533522	0.9949373	0.31330711	3.175597694	0.001495282	0.0475299
**Fam155a**	1196.608128	0.9946974	0.27293306	3.644473848	0.000267939	0.0141655
**Mast4**	931.3585834	0.9941816	0.27023194	3.678993636	0.000234156	0.0129799
**Cib2**	257.0371475	0.9887057	0.32806887	3.013713984	0.00258071	0.0697073
**Mef2c**	5765.01711	0.9886622	0.30880019	3.201624214	0.001366551	0.044962
**Mast1**	810.2858051	0.9882573	0.33395739	2.959231658	0.003084072	0.0780528
**Srrm4**	462.3585236	0.9880926	0.28444601	3.473743902	0.000513251	0.0222949
**Sertm1**	238.0086959	0.9778038	0.34326945	2.848502157	0.004392556	0.0979817
**Foxp4**	577.4633222	0.9771978	0.21608811	4.522219034	6.12E-06	0.0007837
**Cpne6**	1072.786119	0.9743215	0.24204932	4.025301594	5.69E-05	0.0047226
**Epop**	807.2940103	0.9729197	0.22172207	4.388014825	1.14E-05	0.0012588
**Synj2**	1127.127803	0.9723521	0.30534273	3.184461151	0.001450238	0.0469384
**Rps13**	398.4812934	0.9720334	0.30613246	3.17520525	0.001497306	0.0475299
**Agps**	890.5877593	0.9707817	0.29115421	3.334252664	0.000855289	0.0318451
**Basp1**	5851.599979	0.9565293	0.19310362	4.953450656	7.29E-07	0.0001407
**Smyd2**	516.8952901	0.9525979	0.309531	3.077552331	0.002087082	0.0599723
**Stmn1**	5140.651899	0.9464862	0.23264844	4.068311218	4.74E-05	0.00402
**Ube2ql1**	2297.8142	0.9378887	0.22616684	4.146888534	3.37E-05	0.0030906
**Myrip**	924.9489127	0.9335641	0.23026163	4.054362298	5.03E-05	0.0042434
**Igf2**	846.5032687	0.9324378	0.28635181	3.256266415	0.001128878	0.0390014
**Tmem42**	189.9903821	0.9314167	0.31461327	2.960513128	0.00307127	0.0778614
**Ntn3**	186.2587473	0.9309854	0.32607633	2.855114853	0.004302127	0.0963431
**Sprn**	2133.896412	0.9292585	0.28889183	3.216631144	0.001297052	0.0432465
**Ly6e**	2058.609341	0.9290666	0.21379438	4.34560834	1.39E-05	0.0014329

**Smpdl3a**	640.0438924	-0.654121	0.223111305	-2.931814857	0.0033699	0.0824759
**Bex1**	1002.604941	-0.653601	0.205702926	-3.17740271	0.001486	0.0474095
**Usp28**	972.0792489	-0.648636	0.186596381	-3.476146197	0.0005087	0.0221609
**Akap8**	1650.046284	-0.643894	0.191249523	-3.366772536	0.0007605	0.0289705
**Ppp4r4**	1411.245035	-0.64116	0.218475653	-2.934695536	0.0033388	0.081849
**Jcad**	5549.305583	-0.636541	0.144931867	-4.392004193	1.12E-05	0.0012504
**Rasd2**	13500.75847	-0.633978	0.212009762	-2.990326017	0.0027868	0.0730171
**Slco1a4**	825.9974335	-0.631841	0.201768891	-3.131508066	0.0017391	0.052727
**Kcnj2**	945.7637643	-0.630103	0.188429848	-3.343965661	0.0008259	0.0310622
**Uggt2**	474.3431251	-0.629431	0.218346094	-2.882723285	0.0039425	0.0909477
**Ppp1r7**	3546.736795	-0.629292	0.210138709	-2.994650495	0.0027476	0.0725264
**Coq10b**	531.299209	-0.629095	0.216876377	-2.90070883	0.0037232	0.0886305
**Dgat2**	1974.249662	-0.627975	0.181289259	-3.463940075	0.0005323	0.0229224
**Actn1**	3564.668637	-0.627913	0.213283889	-2.944024548	0.0032397	0.0800732
**Dock7**	774.1728702	-0.626122	0.196004348	-3.194426675	0.0014011	0.045646
**Pcsk7**	560.4500093	-0.623142	0.20730315	-3.005944985	0.0026476	0.070869
**Pde10a**	22317.4207	-0.622142	0.193079788	-3.222201612	0.0012721	0.0428532
**Arid5b**	706.2851069	-0.621283	0.214926575	-2.890676288	0.0038441	0.0899347
**Spock3**	7187.938674	-0.616802	0.185876371	-3.31834602	0.0009055	0.0332159
**Cnst**	2043.897797	-0.616669	0.17545217	-3.514742067	0.0004402	0.0201831
**Smad4**	987.4232061	-0.615093	0.207987162	-2.957361894	0.0031028	0.0783941
**Crebrf**	1205.732433	-0.614466	0.180036557	-3.413008156	0.0006425	0.0262947
**Cebpz**	944.9225982	-0.610274	0.200697836	-3.040759646	0.0023598	0.0654058
**Mbnl1**	2319.012602	-0.609403	0.172475931	-3.533262331	0.0004105	0.0189965
**B3gnt2**	1711.672511	-0.60826	0.192052646	-3.167149914	0.0015394	0.0482813
**Lzts3**	8257.011753	-0.607891	0.211672823	-2.871842843	0.0040809	0.0931263
**Plekha5**	2014.211363	-0.606285	0.157342118	-3.853292615	0.0001165	0.0076933
**Grik2**	2441.228346	-0.603165	0.212691859	-2.835862503	0.0045702	0.0996991
**Adcy5**	25299.64335	-0.590956	0.197284082	-2.995455982	0.0027403	0.0725264
**Tbc1d16**	2300.47161	-0.590481	0.173846137	-3.396572066	0.0006824	0.0270322
**Kcnab1**	7524.587923	-0.588629	0.181377145	-3.245331461	0.0011731	0.0402498
**Smarca1**	881.2347235	-0.587179	0.185345488	-3.168025529	0.0015348	0.0482813

**Ntng2**	443.739316	0.9227547	0.27313992	3.378322449	0.000729295	0.0284368
**Tmem151b**	1596.663347	0.918618	0.25601189	3.588185077	0.000332988	0.0166561
**Hccs**	768.6547761	0.9180967	0.19763095	4.645510452	3.39E-06	0.000499
**Ephb3**	229.7034747	0.9168319	0.30834246	2.973420723	0.002945005	0.0758304
**Eif2s3y**	1216.643223	0.915555	0.18277286	5.009250478	5.46E-07	0.000109
**Khdrbs3**	1920.764093	0.9094463	0.15858843	5.734632341	9.77E-09	3.38E-06
**Gng2**	2309.816478	0.9065507	0.30673701	2.955465754	0.003121974	0.0784772
**Gabra3**	1522.480268	0.9017169	0.29671088	3.039042281	0.002373315	0.0655353
**Cacna1i**	3013.746066	0.8995246	0.16191112	5.55566945	2.77E-08	8.51E-06
**Hivep1**	974.5930834	0.8969293	0.23475678	3.820674876	0.000133087	0.0084493
**Sh3gl2**	3602.833056	0.896917	0.22629396	3.963504178	7.39E-05	0.0055697
**Stmn2**	3889.485747	0.8946676	0.26676073	3.353820301	0.000797041	0.0302835
**Tnks1bp1**	813.7947052	0.8839026	0.18753048	4.71338112	2.44E-06	0.0003731
**Dlgap1**	5161.362886	0.8814717	0.29475232	2.990550391	0.002784752	0.0730171
**Thoc3**	530.6168635	0.8805422	0.21926767	4.015832602	5.92E-05	0.004862
**Ildr2**	1402.514439	0.8592192	0.2725675	3.152317134	0.001619802	0.0500594
**Nt5c**	367.2103776	0.8582165	0.27864826	3.079927599	0.002070509	0.0597272
**Sncb**	3974.782978	0.8580933	0.28530803	3.007602965	0.002633169	0.0706898
**Tspan33**	266.0408108	0.8574229	0.29818325	2.875489933	0.00403401	0.0923409
**Pak1**	5903.557966	0.8554432	0.26506555	3.227289467	0.00124969	0.0422902
**Rims1**	3462.701146	0.8512317	0.24008856	3.545490457	0.000391883	0.0184235
**Tagln3**	3530.753161	0.8459993	0.14915728	5.671860292	1.41E-08	4.77E-06
**Arhgdig**	637.6564008	0.8447914	0.25522693	3.309961808	0.000933087	0.0338096
**Adcy1**	9051.897136	0.844466	0.26960001	3.132292204	0.001734472	0.0526939
**Igfbp6**	362.5749538	0.8331592	0.25140905	3.31395887	0.00091985	0.0334934
**Nr4a2**	708.1056767	0.8322955	0.21112295	3.942231134	8.07E-05	0.0058788
**Cabp7**	390.7313405	0.8320751	0.24611059	3.380899323	0.00072249	0.0282456
**Rasal1**	777.3544979	0.8192863	0.19717015	4.155224902	3.25E-05	0.0030173
**Sema4f**	653.7437018	0.8144097	0.25664239	3.173325053	0.001507037	0.0476352
**Igsf21**	948.156558	0.8106761	0.18631345	4.351140801	1.35E-05	0.0014169
**Sobp**	941.9924191	0.8077486	0.23942233	3.373739408	0.000741545	0.0287368
**Coprs**	258.795001	0.8073976	0.2721948	2.966249341	0.00301456	0.0769514
**Fam163b**	1939.087214	0.8048615	0.20758994	3.877170059	0.000105678	0.0071731
**Rapgefl1**	1574.444989	0.8044809	0.20980633	3.83439781	0.000125872	0.0081303
**Npy**	1306.512091	0.7962429	0.18128434	4.392232379	1.12E-05	0.0012504
**Etl4**	1434.670964	0.7940361	0.23111339	3.435699134	0.000591027	0.0247332
**Rims3**	3046.595693	0.7939202	0.2303386	3.446752725	0.000567368	0.0238777
**Uty**	538.6946994	0.793389	0.21084653	3.762874492	0.000167972	0.0100216
**Anxa11**	406.9602654	0.7898527	0.23886896	3.306635836	0.000944236	0.0341303
**Islr2**	1015.013404	0.7774529	0.2286563	3.400093806	0.000673627	0.0269679
**Phospho1**	482.3881736	0.7752523	0.27014894	2.869721963	0.004108329	0.0934661
**Brinp2**	1707.198381	0.7708741	0.16169334	4.767506965	1.87E-06	0.0003079
**Exosc4**	408.0294772	0.7667252	0.24132233	3.177182888	0.001487132	0.0474095
**Trib2**	1132.788654	0.7637704	0.26681646	2.862531231	0.004202718	0.0945994
**Il17ra**	337.0013338	0.7636058	0.26303799	2.903024567	0.003695776	0.0883458
**Rhof**	587.0962322	0.7612828	0.26299352	2.89468272	0.003795419	0.0894185
**Chga**	3480.67729	0.7587882	0.18047776	4.204331056	2.62E-05	0.0025058
**Cnrip1**	686.284965	0.7511568	0.26382736	2.847152626	0.004411221	0.0980195

**Cpne4**	907.0555494	0.7498758	0.22488786	3.334443328	0.000854703	0.0318451
**Faim2**	6617.065188	0.7416389	0.17195159	4.313067881	1.61E-05	0.0016383
**Ephb6**	1928.115809	0.7396353	0.21225749	3.484613182	0.000492849	0.0217263
**Akap2**	989.7410576	0.7391633	0.23262656	3.177467489	0.001485673	0.0474095
**Ppfia3**	3325.75262	0.7368452	0.18677675	3.945058316	7.98E-05	0.0058788
**Ddx3y**	1617.808649	0.7367423	0.18019408	4.088604688	4.34E-05	0.0037483
**Gcn1l1**	1326.016012	0.7340035	0.17348952	4.230823252	2.33E-05	0.0022461
**Gng3**	2022.539557	0.7338699	0.20689081	3.547136259	0.000389443	0.0183669
**Ankrd13d**	554.0025687	0.7289265	0.21440111	3.399826244	0.000674287	0.0269679
**Rims4**	1551.201945	0.7248694	0.1883106	3.849329018	0.000118442	0.0076933
**Pfkl**	2719.027613	0.7226004	0.1524646	4.73946344	2.14E-06	0.0003388
**Rph3a**	8002.362404	0.7206549	0.14966863	4.815003048	1.47E-06	0.0002543
**Arhgap44**	2204.520427	0.7163333	0.18044204	3.96988014	7.19E-05	0.0054783
**Depdc5**	910.7194789	0.7153484	0.22311859	3.206135368	0.001345307	0.0444131
**Ttc9b**	1429.920292	0.7084669	0.18119301	3.91001212	9.23E-05	0.006595
**Acsl4**	1250.393158	0.7047485	0.23689218	2.974975961	0.002930115	0.075704
**Ccl27a**	1152.99017	0.7042477	0.17745398	3.968621465	7.23E-05	0.0054793
**Kcnmb4**	838.4855774	0.7038586	0.22134158	3.179965478	0.001472926	0.0473632
**Snap91**	4803.149232	0.7031996	0.19240333	3.654820159	0.000257362	0.0138641
**Rpl21**	2233.183197	0.6945749	0.15540899	4.469335238	7.85E-06	0.0009477
**Rpl24**	971.9099754	0.6925002	0.22608778	3.062970577	0.002191516	0.0618407
**Sv2b**	5011.166858	0.6906585	0.17682754	3.905830944	9.39E-05	0.0066429
**Apba2**	2282.605224	0.6882647	0.1621924	4.243507626	2.20E-05	0.0021367
**Tubb2a**	6718.493403	0.6852323	0.17802355	3.849110461	0.000118548	0.0076933
**Kdm5d**	959.1311764	0.6846102	0.19823839	3.45346957	0.000553425	0.0235578
**Dgkz**	6036.181075	0.6842034	0.1756695	3.894833168	9.83E-05	0.00679
**Rap1gap2**	2966.935294	0.6839325	0.21908156	3.121816638	0.001797388	0.0540526
**Camk1**	831.3937552	0.6797998	0.23383764	2.907144498	0.003647447	0.0875387
**Nell2**	6860.150743	0.6789371	0.18595532	3.651076817	0.000261143	0.0139052
**Fam174b**	451.3167276	0.6787389	0.22854919	2.96977172	0.002980211	0.0763345
**Garnl3**	1448.540191	0.6772764	0.19274493	3.513848142	0.000441665	0.0201889
**Itpk1**	1163.09067	0.6757199	0.23669442	2.854819803	0.004306125	0.0963431
**Podxl2**	1701.054648	0.6703299	0.16713596	4.010686603	6.05E-05	0.0049195
**Btbd3**	1728.821824	0.6698782	0.21842681	3.066831523	0.002163408	0.0613351
**Enc1**	9110.539753	0.6655024	0.16124553	4.127260812	3.67E-05	0.0033255
**Syp**	15181.49607	0.6635847	0.22961058	2.890044055	0.003851878	0.0899741
**Syn1**	9528.203162	0.6633893	0.17203768	3.856069971	0.000115225	0.0076761
**Bcr**	7731.294284	-0.658839	0.1935325	-3.40427858	0.000663391	0.0269271
**Dkk3**	3795.501853	0.6526468	0.14420326	4.525881259	6.01E-06	0.0007785
**Prkar1b**	9664.441188	0.6471993	0.20744841	3.119808338	0.001809687	0.0542031
**Adcy2**	2084.633062	0.6467528	0.21556468	3.00027249	0.002697382	0.0719431
**Rpl27a**	947.2615204	0.6451617	0.18107875	3.562879002	0.00036681	0.0177502
**Plch2**	1060.900534	0.6446654	0.17871253	3.607275682	0.000309429	0.0157427
**Ywhah**	11632.05755	0.6419759	0.19473301	3.296697821	0.000978287	0.034769
**Ucp2**	699.2587817	0.6405998	0.1940052	3.301972194	0.000960076	0.0345735
**Nlk**	1661.095398	0.6390924	0.16205303	3.943723783	8.02E-05	0.0058788
**Gnas**	11894.97242	0.6380307	0.20049535	3.182271616	0.001461247	0.0471919
**Tspan5**	2312.77734	0.6351233	0.15443579	4.112539557	3.91E-05	0.0034611

**Slc36a1**	1150.370525	0.6322061	0.21700908	2.913270319	0.003576648	0.0862576
**Csmd2**	802.3050718	0.6276194	0.21361336	2.938109504	0.003302204	0.0810868
**Mturn**	2000.467348	0.6238733	0.18817373	3.315411414	0.000915083	0.0334017
**Rab26**	612.8435605	0.6153494	0.21033868	2.925517107	0.003438843	0.0837499
**Magi1**	1521.683955	0.6132036	0.20390754	3.007262782	0.002636118	0.0706898
**Htr2a**	672.4175037	0.6101961	0.20733585	2.943032296	0.003250145	0.0800732
**Snap25**	39699.12314	0.6016743	0.17270862	3.483753893	0.000494434	0.0217317
**Rab3b**	1142.536572	0.5988353	0.18975314	3.155864804	0.00160023	0.0499433
**Gabra5**	752.045997	0.5982113	0.19951524	2.998323663	0.002714692	0.0720169
**Map4k3**	1038.425712	0.5975491	0.18294342	3.266305707	0.001089606	0.0381773
**Rap2a**	1490.345576	0.595476	0.16996542	3.503512607	0.000459165	0.0206781
**Stmn3**	5629.624863	0.5949313	0.15166347	3.922706656	8.76E-05	0.0063453
**Cdh22**	710.2025617	0.5946648	0.2065874	2.878514648	0.003995527	0.0917427
**Slc45a4**	904.7313899	0.5936389	0.20851757	2.846949015	0.004414043	0.0980195
**Tubb4b**	4476.270029	0.5926025	0.15480734	3.828000114	0.000129189	0.0083083
**Srcin1**	5255.485505	0.5905576	0.16421197	3.596313011	0.000322759	0.016199
**Tango2**	1065.843907	0.5872566	0.20096474	2.922187467	0.003475823	0.0841193
**Nrgn**	24914.26307	0.5870354	0.16037197	3.66046136	0.000251762	0.0136503
