# Supplementary material for: α-Synuclein oligomers potentiate neuroinflammatory NF-κB activity and induce Cav3.2 calcium signaling in astrocytes
Source: Transl Neurodegener. 2024 Feb 21;13:11. doi: 10.1186/s40035-024-00401-4 (PMC10880263; doi:10.1186/s40035-024-00401-4)
Supplement: Supplementary file 3 — Additional file 3: Proteins detected in the CM of a1H- and mock-transfected astrocytes. Detection of all the proteins that are secreted from astrocytes 48 hours following transfection. Proteins identified in 3 independent experiments are listed. [file 40035_2024_401_MOESM3_ESM.pdf]

Accession	Description	Symbol	mock.1.xlsx	mock.1.2.xlsx	mock.3.4.xlsx	a1Ha.1.xlsx	a1Ha.1.2.xlsx	a1Ha.3.4.xlsx	Average_mock	Average_a1H	St_Dv_mock	St_Dv_a1H	MW_p_a1Hvsmock	BH_p_a1Hvsmock	Ratio_a1Hvsmock	Log2_Ratio.a1Hvsmock
Q80W15	Insulin-like growth factor-binding protein-like 1 OS=Mus musculus OX=10090 GN=Igfbpl1 PE=2 SV=1 - [IBPL1_MOUSE]	Igfbpl1	0	0	0	12.98234431	38.94703294	12.98234431	0	21.63724052	0	14.99071997	0.059346439	0.726530612	only in a1H	only in a1H
Q8R5G7	Arf-GAP with Rho-GAP domain, ANK repeat and PH domain-containing protein 3 OS=Mus musculus OX=10090 GN=Arap3 PE=1 SV=3 - [ARAP3_MOUSE]	Arap3	0	0	0	18.15570722	6.051902406	6.051902406	0	10.08650401	0	6.988134967	0.059346439	0.726530612	only in a1H	only in a1H
P17515	C-X-C motif chemokine 10 OS=Mus musculus OX=10090 GN=Cxcl10 PE=1 SV=1 - [CXL10_MOUSE]	Cxcl10	0	0	0	258.2531849	183.5637967	103.5682244	0	181.7950686	0	77.35764697	0.06360257	0.726530612	only in a1H	only in a1H
P62301	40S ribosomal protein S13 OS=Mus musculus OX=10090 GN=Rps13 PE=1 SV=2 - [RS13_MOUSE]	Rps13	0	0	0	56.67004152	69.7156691	36.20675152	0	54.19748738	0	16.89073806	0.06360257	0.726530612	only in a1H	only in a1H
P62748	Hippocalcin-like protein 1 OS=Mus musculus OX=10090 GN=Hpcal1 PE=1 SV=2 - [HPCL1_MOUSE]	Hpcal1	0	0	0	22.70314216	24.86579669	15.47055867	0	21.01316584	0	4.920329187	0.06360257	0.726530612	only in a1H	only in a1H
Q571F8	Glutaminase liver isoform, mitochondrial OS=Mus musculus OX=10090 GN=Gls2 PE=1 SV=2 - [GLSL_MOUSE]	Gls2	0	0	0	106.3299577	164.1155136	146.9389735	0	139.1281483	0	29.67405196	0.06360257	0.726530612	only in a1H	only in a1H
Q7TSK2	Seizure protein 6 OS=Mus musculus OX=10090 GN=Sez6 PE=1 SV=1 - [SEZ6_MOUSE]	Sez6	0	0	0	7.475158091	11.98504137	6.486733154	0	8.648977539	0	2.931081266	0.06360257	0.726530612	only in a1H	only in a1H
Q8CA71	Protein shisa-4 OS=Mus musculus OX=10090 GN=Shisa4 PE=1 SV=1 - [SHSA4_MOUSE]	Shisa4	0	0	0	21.14700895	31.32684657	29.69037139	0	27.38807564	0	5.466506908	0.06360257	0.726530612	only in a1H	only in a1H
Q8VCH8	UBX domain-containing protein 4 OS=Mus musculus OX=10090 GN=Ubxn4 PE=1 SV=1 - [UBXN4_MOUSE]	Ubxn4	0	0	0	67.89642693	82.80475368	5.443233569	0	52.04813806	0	41.04360356	0.06360257	0.726530612	only in a1H	only in a1H
Q920A5	Retinoid-inducible serine carboxypeptidase OS=Mus musculus OX=10090 GN=Scpep1 PE=1 SV=2 - [RISC_MOUSE]	Scpep1	0	0	0	6.420884577	29.35080368	32.08729782	0	22.61966202	0	14.09511948	0.06360257	0.726530612	only in a1H	only in a1H
Q9CQE5	Regulator of G-protein signaling 10 OS=Mus musculus OX=10090 GN=Rgs10 PE=1 SV=1 - [RGS10_MOUSE]	Rgs10	0	0	0	7.699459804	7.157981414	5.413764434	0	6.757068551	0	1.194424202	0.06360257	0.726530612	only in a1H	only in a1H
Q9DCN2	NADH-cytochrome b5 reductase 3 OS=Mus musculus OX=10090 GN=Cyb5r3 PE=1 SV=3 - [NB5R3_MOUSE]	Cyb5r3	0	0	0	26.91794647	26.74621469	17.88805372	0	23.85073829	0	5.164550168	0.06360257	0.726530612	only in a1H	only in a1H
Q9QY93	dCTP pyrophosphatase 1 OS=Mus musculus OX=10090 GN=Dctpp1 PE=1 SV=1 - [DCTP1_MOUSE]	Dctpp1	0	0	0	19.14231026	16.60989298	11.74816526	0	15.83345617	0	3.757723468	0.06360257	0.726530612	only in a1H	only in a1H
A2ANX9	Zinc finger protein 711 OS=Mus musculus OX=10090 GN=Znf711 PE=3 SV=1 - [ZN711_MOUSE]	Znf711	120.9397439	84.08222373	17.35343626	0	0	0	74.12513464	0	52.50608062	0	0.06360257	0.726530612	only in mock	only in mock
Q9EPX2	Papilin OS=Mus musculus OX=10090 GN=Papln PE=2 SV=2 - [PPN_MOUSE]	Papln	26.42014415	15.48638999	20.03902582	0	0	0	20.64851999	0	5.492299825	0	0.06360257	0.726530612	only in mock	#NUM!
Q8K209	Adhesion G-protein coupled receptor G1 OS=Mus musculus OX=10090 GN=Adgrg1 PE=1 SV=1 - [AGRG1_MOUSE]	Adgrg1	273.3998939	40.37594353	36.1331504	0	0	32.2954164	116.6363293	10.7651388	135.7778028	18.64576735	0.0765225	0.726530612	0.092296619	-3.437578396
Q9D9W6	Protein FAM217A OS=Mus musculus OX=10090 GN=Fam217a PE=2 SV=1 - [F217A_MOUSE]	Fam217a	58.69416194	144.9274287	95.65403107	41.32386666	0	0	99.75854057	13.77462222	43.26290932	23.85834554	0.0765225	0.726530612	0.138079628	-2.856427607
P61327	Protein mago nashi homolog OS=Mus musculus OX=10090 GN=Magoh PE=2 SV=1 - [MGN_MOUSE]	Magoh	92.82965264	91.24213316	70.54770288	0	48.89694181	0	84.87316289	16.2989806	12.43157906	28.23066252	0.0765225	0.726530612	0.192039274	-2.380526706
Q9WV85	Nucleoside diphosphate kinase 3 OS=Mus musculus OX=10090 GN=Nme3 PE=1 SV=3 - [NDK3_MOUSE]	Nme3	9.526347051	16.35535782	15.81873161	0	0	9.113112434	13.90014549	3.037704145	3.797311752	5.261457917	0.0765225	0.726530612	0.218537579	-2.194046713
P60867	40S ribosomal protein S20 OS=Mus musculus OX=10090 GN=Rps20 PE=1 SV=1 - [RS20_MOUSE]	Rps20	208.9874875	69.66249584	69.66249584	347.4036195	430.6817521	230.3181191	116.1041597	336.1344969	80.43932145	100.656054	0.0765225	0.726530612	2.895111577	1.53361895
Q62376	U1 small nuclear ribonucleoprotein 70 kDa OS=Mus musculus OX=10090 GN=Snrnp70 PE=1 SV=2 - [RU17_MOUSE]	Snrnp70	41.49998899	0	0	42.72174939	61.35239933	45.32994432	13.83332966	49.80136435	23.96002981	10.08813546	0.0765225	0.726530612	3.600099583	1.848036814
P56542	Deoxyribonuclease-2-alpha OS=Mus musculus OX=10090 GN=Dnase2 PE=1 SV=1 - [DNS2A_MOUSE]	Dnase2	35.28787831	0	0	41.35069509	52.35052023	39.19460356	11.7626261	44.29860629	20.37346604	7.056002495	0.0765225	0.726530612	3.766047301	1.91305112
Q6PDY2	2-aminoethanethiol dioxygenase OS=Mus musculus OX=10090 GN=Ado PE=1 SV=2 - [AEDO_MOUSE]	Ado	30.37228885	0	0	44.42869873	43.84780458	33.13684529	10.12409628	40.4711162	17.53544914	6.3583022	0.0765225	0.726530612	3.99750408	1.999099506
O89023	Tripeptidyl-peptidase 1 OS=Mus musculus OX=10090 GN=Tpp1 PE=1 SV=2 - [TPP1_MOUSE]	Tpp1	0	41.77432275	0	44.85070032	50.36329816	77.89285639	13.92477425	57.70228496	24.11841648	17.70145657	0.0765225	0.726530612	4.143857841	2.050974511
O88543	COP9 signalosome complex subunit 3 OS=Mus musculus OX=10090 GN=Cops3 PE=1 SV=3 - [CSN3_MOUSE]	Cops3	48.57490369	0	0	90.79534745	67.40137489	54.07063209	16.19163456	70.75578481	28.04473372	18.59072965	0.0765225	0.726530612	4.369897587	2.127599469
P48024	Eukaryotic translation initiation factor 1 OS=Mus musculus OX=10090 GN=Eif1 PE=1 SV=2 - [EIF1_MOUSE]	Eif1	63.0717003	0	0	113.6493452	100.9675408	67.53726294	21.0239001	94.05138297	36.41446315	23.82133415	0.0765225	0.726530612	4.473545941	2.161418832
Q8CJG1	Protein argonaute-1 OS=Mus musculus OX=10090 GN=Ago1 PE=1 SV=2 - [AGO1_MOUSE]	Ago1	0	14.92872915	0	31.07461664	22.92933692	20.59704024	4.976243049	24.86699793	8.619105791	5.500981678	0.0765225	0.726530612	4.997142963	2.321103493
Q9QUR7	Peptidyl-prolyl cis-trans isomerase NIMA-interacting 1 OS=Mus musculus OX=10090 GN=Pin1 PE=1 SV=1 - [PIN1_MOUSE]	Pin1	54.19958858	0	0	103.1118055	103.8418197	67.76280145	18.06652953	91.57214223	31.29214705	20.62272439	0.0765225	0.726530612	5.068607233	2.341589374
Q921F4	Heterogeneous nuclear ribonucleoprotein L-like OS=Mus musculus OX=10090 GN=Hnrnpll PE=1 SV=3 - [HNRLL_MOUSE]	Hnrnpll	43.98270173	0	0	91.19755892	87.73839224	45.36489928	14.66090058	74.76695015	25.39342468	25.52159684	0.0765225	0.726530612	5.09975153	2.350426958
O88508	DNA (cytosine-5)-methyltransferase 3A OS=Mus musculus OX=10090 GN=Dnmt3a PE=1 SV=2 - [DNM3A_MOUSE]	Dnmt3a	13.52387687	0	0	28.70974372	17.86163304	22.46798235	4.507958956	23.0131197	7.808013949	5.444562188	0.0765225	0.726530612	5.104997612	2.351910286
P97822	Acidic leucine-rich nuclear phosphoprotein 32 family member E OS=Mus musculus OX=10090 GN=Anp32e PE=1 SV=2 - [AN32E_MOUSE]	Anp32e	43.57657612	0	0	85.05833323	104.0791474	47.17665663	14.52552537	78.77137908	25.15894796	28.9675285	0.0765225	0.726530612	5.422962478	2.439081188
P47199	Quinone oxidoreductase OS=Mus musculus OX=10090 GN=Cryz PE=1 SV=1 - [QOR_MOUSE]	Cryz	34.30443628	0	0	56.18955335	83.83246583	57.49033978	11.43481209	65.83745299	19.80567552	15.59770421	0.0765225	0.726530612	5.757633133	2.525475866
Q80UW2	F-box only protein 2 OS=Mus musculus OX=10090 GN=Fbxo2 PE=1 SV=1 - [FBX2_MOUSE]	Fbxo2	21.47846786	0	0	40.84404556	59.01991142	28.66242688	7.159489288	42.84212795	12.4005992	15.27705688	0.0765225	0.726530612	5.983964251	2.581101556
O70589	Peripheral plasma membrane protein CASK OS=Mus musculus OX=10090 GN=Cask PE=1 SV=2 - [CSKP_MOUSE]	Cask	0	0	8.036304309	15.82172976	17.88830716	14.47787042	2.678768103	16.06263578	4.639762456	1.717933781	0.0765225	0.726530612	5.99627708	2.58406705
P15209	BDNF/NT-3 growth factors receptor OS=Mus musculus OX=10090 GN=Ntrk2 PE=1 SV=1 - [NTRK2_MOUSE]	Ntrk2	12.02166001	0	0	18.90113608	35.39005873	18.09706494	4.007220003	24.12941991	6.940708643	9.760282912	0.0765225	0.726530612	6.021486191	2.590119609
P70399	TP53-binding protein 1 OS=Mus musculus OX=10090 GN=Tp53bp1 PE=1 SV=3 - [TP53B_MOUSE]	Tp53bp1	4.825595164	0	0	13.39945165	13.15329373	8.850915129	1.608531721	11.80122017	2.786058667	2.558001821	0.0765225	0.726530612	7.336641246	2.875119741
P52760	2-iminobutanoate/2-iminopropanoate deaminase OS=Mus musculus OX=10090 GN=Rida PE=1 SV=3 - [RIDA_MOUSE]	Rida	30.93559756	0	0	51.43966586	151.805898	33.80383927	10.31186585	79.01646771	17.86067558	63.65124875	0.0765225	0.726530612	7.662674132	2.937847954
Q9JHW9	Aldehyde dehydrogenase family 1 member A3 OS=Mus musculus OX=10090 GN=Aldh1a3 PE=1 SV=1 - [AL1A3_MOUSE]	Aldh1a3	0	0	10.50918753	37.87444864	32.12679653	10.6095466	3.503062511	26.87026392	6.067482252	14.37244141	0.0765225	0.726530612	7.670506545	2.939321854
Q61730	Interleukin-1 receptor accessory protein OS=Mus musculus OX=10090 GN=Il1rap PE=1 SV=1 - [IL1AP_MOUSE]	Il1rap	72.66918257	96.93675017	121.0707975	0	18.02952674	54.08858022	96.89224343	24.03936899	24.20083818	27.54055701	0.1	0.726530612	0.248104163	-2.01098215
Q9QXT0	Protein canopy homolog 2 OS=Mus musculus OX=10090 GN=Cnpy2 PE=1 SV=1 - [CNPY2_MOUSE]	Cnpy2	130.4086202	260.8715644	140.4812593	52.6243403	44.29296214	60.9962953	177.2538146	52.63786591	72.59001707	8.351674793	0.1	0.726530612	0.296963233	-1.751643771
Q8BP92	Reticulocalbin-2 OS=Mus musculus OX=10090 GN=Rcn2 PE=1 SV=1 - [RCN2_MOUSE]	Rcn2	54.33581328	203.4263557	86.01385101	35.90144778	36.23872622	44.85388399	114.5920066	38.99801933	78.54636014	5.074130699	0.1	0.726530612	0.34032059	-1.555033655
Q8BH97	Reticulocalbin-3 OS=Mus musculus OX=10090 GN=Rcn3 PE=1 SV=1 - [RCN3_MOUSE]	Rcn3	17.84301122	36.06536254	21.41586024	9.510975934	6.732925841	14.32350525	25.108078	10.18913567	9.655976177	3.840462133	0.1	0.726530612	0.405811057	-1.301119921
P11087	Collagen alpha-1(I) chain OS=Mus musculus OX=10090 GN=Col1a1 PE=1 SV=4 - [CO1A1_MOUSE]	Col1a1	41.27204191	64.64545264	43.73762053	30.1549288	30.91587937	0	49.88503836	20.35693606	12.84220159	17.63372892	0.1	0.726530612	0.408076985	-1.293086748
P63323	40S ribosomal protein S12 OS=Mus musculus OX=10090 GN=Rps12 PE=1 SV=2 - [RS12_MOUSE]	Rps12	676.0867044	612.5339175	481.6610994	236.9666974	262.0853155	226.4384094	590.0939071	241.8301408	99.13624224	18.31434655	0.1	0.726530612	0.409816366	-1.286950494
P97370	Sodium/potassium-transporting ATPase subunit beta-3 OS=Mus musculus OX=10090 GN=Atp1b3 PE=1 SV=1 - [AT1B3_MOUSE]	Atp1b3	44.4512596	26.99684386	33.64915833	18.82430167	24.569191	0	35.03242059	14.46449756	8.809041838	12.8517394	0.1	0.726530612	0.412888899	-1.276174465
O35887	Calumenin OS=Mus musculus OX=10090 GN=Calu PE=1 SV=1 - [CALU_MOUSE]	Calu	229.6414322	462.7075803	386.1421176	172.9854447	124.9681366	160.9235844	359.4970434	152.9590552	118.7957379	24.97980712	0.1	0.726530612	0.425480704	-1.232834388
P62814	V-type proton ATPase subunit B, brain isoform OS=Mus musculus OX=10090 GN=Atp6v1b2 PE=1 SV=1 - [VATB2_MOUSE]	Atp6v1b2	95.79796919	227.7482235	215.9650821	66.91541823	90.67149539	73.75102857	179.8370916	77.1126474	73.01808802	12.22960185	0.1	0.726530612	0.428791673	-1.221651205
Q8BYB9	Protein O-glucosyltransferase 1 OS=Mus musculus OX=10090 GN=Poglut1 PE=1 SV=2 - [PGLT1_MOUSE]	Poglut1	29.65741124	61.07777501	61.61579888	22.05921688	23.9401776	20.21734471	50.78366171	22.0722464	18.29784719	1.861450647	0.1	0.726530612	0.434632826	-1.202130956
Q8R034	Anaphase-promoting complex subunit 13 OS=Mus musculus OX=10090 GN=Anapc13 PE=3 SV=1 - [APC13_MOUSE]	Anapc13	27.52025764	39.61877845	47.08450545	22.42071664	0	27.22791189	38.07451385	16.54954284	9.873120731	14.53247425	0.1	0.726530612	0.434661961	-1.202034251
Q9DC23	DnaJ homolog subfamily C member 10 OS=Mus musculus OX=10090 GN=Dnajc10 PE=1 SV=2 - [DJC10_MOUSE]	Dnajc10	22.37230909	45.28742312	19.89662857	9.128450296	19.5564343	12.38351402	29.18545359	13.68946621	13.99954698	5.335245631	0.1	0.726530612	0.469051	-1.092183298
Q61543	Golgi apparatus protein 1 OS=Mus musculus OX=10090 GN=Glg1 PE=1 SV=1 - [GSLG1_MOUSE]	Glg1	17.92937186	42.72502726	29.09849337	15.78664353	12.14544399	14.54697163	29.91763083	14.15968638	12.41810659	1.851236322	0.1	0.726530612	0.473289027	-1.079206621
O08795	Glucosidase 2 subunit beta OS=Mus musculus OX=10090 GN=Prkcsh PE=1 SV=1 - [GLU2B_MOUSE]	Prkcsh	109.3225064	205.4964377	133.3406086	72.0620817	70.6620858	76.17620996	149.3865176	72.96679249	50.05456683	2.866228795	0.1	0.726530612	0.488442958	-1.033738006
P09103	Protein disulfide-isomerase OS=Mus musculus OX=10090 GN=P4hb PE=1 SV=2 - [PDIA1_MOUSE]	P4hb	275.5485322	390.8340316	267.9831855	166.9613966	153.1437236	154.261066	311.4552498	158.1220621	68.84803463	7.675447212	0.1	0.726530612	0.507687901	-0.977986215
Q922R8	Protein disulfide-isomerase A6 OS=Mus musculus OX=10090 GN=Pdia6 PE=1 SV=3 - [PDIA6_MOUSE]	Pdia6	111.5228935	165.4409102	112.8543539	77.09821844	56.33686621	70.73113823	129.9393859	68.05540763	30.7524287	10.63616883	0.1	0.726530612	0.523747339	-0.933057085
Q05793	Basement membrane-specific heparan sulfate proteoglycan core protein OS=Mus musculus OX=10090 GN=Hspg2 PE=1 SV=1 - [PGBM_MOUSE]	Hspg2	42.36600388	49.97563239	50.75345889	16.68557303	23.38972606	36.72163624	47.69836505	25.59897845	4.634307961	10.19909576	0.1	0.726530612	0.53668461	-0.897853577
Q9DB15	39S ribosomal protein L12, mitochondrial OS=Mus musculus OX=10090 GN=Mrpl12 PE=1 SV=2 - [RM12_MOUSE]	Mrpl12	86.42380044	114.4815909	114.6135341	42.96207141	57.24771797	78.65390669	105.1729751	59.62123202	16.23739561	17.96390696	0.1	0.726530612	0.566887377	-0.81886595
P14733	Lamin-B1 OS=Mus musculus OX=10090 GN=Lmnb1 PE=1 SV=3 - [LMNB1_MOUSE]	Lmnb1	818.8138022	1042.510282	799.6773288	637.7408212	376.1300992	544.8083116	887.0004709	519.559744	135.0149144	132.6203607	0.1	0.726530612	0.585749119	-0.771645217
P21619	Lamin-B2 OS=Mus musculus OX=10090 GN=Lmnb2 PE=1 SV=2 - [LMNB2_MOUSE]	Lmnb2	373.7691976	519.6679474	294.6182137	250.1441178	219.7009725	243.8514761	396.0184529	237.8988555	114.1626808	16.07082896	0.1	0.726530612	0.600726693	-0.735219325
Q8BP67	60S ribosomal protein L24 OS=Mus musculus OX=10090 GN=Rpl24 PE=1 SV=2 - [RL24_MOUSE]	Rpl24	141.9528255	162.2061883	133.3590955	83.19224222	129.7283058	63.54692675	145.8393697	92.15582494	14.81106379	33.98901434	0.1	0.726530612	0.631899501	-0.662232969
Q62443	Neuronal pentraxin-1 OS=Mus musculus OX=10090 GN=Nptx1 PE=1 SV=1 - [NPTX1_MOUSE]	Nptx1	261.1822001	240.2411842	437.2047158	227.5304253	172.5213382	228.8569298	312.8760334	209.6362311	108.1797	32.14928244	0.1	0.726530612	0.670029688	-0.577703074
P43274	Histone H1.4 OS=Mus musculus OX=10090 GN=H1-4 PE=1 SV=2 - [H14_MOUSE]	H1-4	559.4101201	300.6794565	342.7626532	280.4063038	261.7873587	270.2219794	400.9507433	270.805214	138.8336394	9.323164771	0.1	0.726530612	0.675407687	-0.566169497
Q71M36	Chondroitin sulfate proteoglycan 5 OS=Mus musculus OX=10090 GN=Cspg5 PE=1 SV=2 - [CSPG5_MOUSE]	Cspg5	194.0452447	216.1266751	210.8514029	89.92709047	139.7833624	190.612377	207.0077742	140.10761	11.5315872	50.34342641	0.1	0.726530612	0.676822938	-0.563149632
Q80YX1	Tenascin OS=Mus musculus OX=10090 GN=Tnc PE=1 SV=1 - [TENA_MOUSE]	Tnc	1650.355871	1197.076749	1636.97721	765.5846802	1105.254639	1177.015731	1494.803277	1015.951683	257.9254955	219.77293	0.1	0.726530612	0.679655778	-0.557123839
P56480	ATP synthase subunit beta, mitochondrial OS=Mus musculus OX=10090 GN=Atp5f1b PE=1 SV=2 - [ATPB_MOUSE]	Atp5f1b	145.694229	227.2678622	193.3208144	136.4444872	126.6244132	124.4761869	188.7609685	129.1816958	40.97753715	6.380816848	0.1	0.726530612	0.684366566	-0.547158815
Q9CZX8	40S ribosomal protein S19 OS=Mus musculus OX=10090 GN=Rps19 PE=1 SV=3 - [RS19_MOUSE]	Rps19	179.5252889	289.2120542	238.241701	143.2443534	167.4266566	173.2743159	235.6596814	161.3151086	54.88894913	15.92051902	0.1	0.726530612	0.684525701	-0.546823385
Q91XA2	Golgi membrane protein 1 OS=Mus musculus OX=10090 GN=Golm1 PE=1 SV=2 - [GOLM1_MOUSE]	Golm1	84.3722698	100.2504361	85.67683014	49.69527292	82.50131341	54.29068546	90.09984536	62.16242393	8.814836316	17.76322811	0.1	0.726530612	0.689928198	-0.535481869
O35226	26S proteasome non-ATPase regulatory subunit 4 OS=Mus musculus OX=10090 GN=Psmd4 PE=1 SV=1 - [PSMD4_MOUSE]	Psmd4	151.2070776	135.4667369	130.6114988	113.7149411	103.216135	78.53163022	139.0951044	98.48756877	10.76653404	18.06200004	0.1	0.726530612	0.708059203	-0.498058102
P15864	Histone H1.2 OS=Mus musculus OX=10090 GN=H1-2 PE=1 SV=2 - [H12_MOUSE]	H1-2	397.9960697	431.1495781	359.45104	297.0829298	275.9790145	273.9121722	396.1988959	282.3247055	35.88303866	12.82270827	0.1	0.726530612	0.712583272	-0.488869479
P61290	Proteasome activator complex subunit 3 OS=Mus musculus OX=10090 GN=Psme3 PE=1 SV=1 - [PSME3_MOUSE]	Psme3	38.56303052	50.59476948	37.20657886	22.72147912	34.96954468	35.48072168	42.12145962	31.0572485	7.369377532	7.223511179	0.1	0.726530612	0.737326028	-0.43962541
Q6A068	Cell division cycle 5-like protein OS=Mus musculus OX=10090 GN=Cdc5l PE=1 SV=2 - [CDC5L_MOUSE]	Cdc5l	21.69568669	19.80278141	17.29064759	16.36018656	13.99576536	13.23086299	19.59637189	14.5289383	2.209761535	1.631371061	0.1	0.726530612	0.741409603	-0.431657294
P05202	Aspartate aminotransferase, mitochondrial OS=Mus musculus OX=10090 GN=Got2 PE=1 SV=1 - [AATM_MOUSE]	Got2	212.0258097	199.1647834	203.6675551	179.8378063	175.3448956	112.1157996	204.9527161	155.7661672	6.526118966	37.86901772	0.1	0.726530612	0.760010261	-0.395909199
P26645	Myristoylated alanine-rich C-kinase substrate OS=Mus musculus OX=10090 GN=Marcks PE=1 SV=2 - [MARCS_MOUSE]	Marcks	1956.770674	2377.886081	2149.468507	1631.59324	1644.629307	1789.907087	2161.375087	1688.709878	210.8100367	87.88140348	0.1	0.726530612	0.781312734	-0.356027968
P84099	60S ribosomal protein L19 OS=Mus musculus OX=10090 GN=Rpl19 PE=1 SV=1 - [RL19_MOUSE]	Rpl19	103.2474355	124.7776102	104.8849676	90.73925194	85.78300184	101.7065825	110.9700044	92.74294541	11.9857357	8.148692873	0.1	0.726530612	0.835747875	-0.258860313
P25785	Metalloproteinase inhibitor 2 OS=Mus musculus OX=10090 GN=Timp2 PE=1 SV=2 - [TIMP2_MOUSE]	Timp2	348.7493472	323.3422206	305.3431469	399.1178742	372.7512398	357.6533502	325.8115716	376.5074881	21.80820551	20.98591761	0.1	0.726530612	1.155598883	0.208640714
O88968	Transcobalamin-2 OS=Mus musculus OX=10090 GN=Tcn2 PE=1 SV=1 - [TCO2_MOUSE]	Tcn2	71.14072016	72.32934556	67.29902232	77.51854966	74.55570893	97.00715011	70.25636268	83.02713623	2.62918316	12.19734386	0.1	0.726530612	1.181773907	0.240954051
P35802	Neuronal membrane glycoprotein M6-a OS=Mus musculus OX=10090 GN=Gpm6a PE=1 SV=1 - [GPM6A_MOUSE]	Gpm6a	81.286855	85.02948764	84.74733749	105.6525664	88.8291053	111.2805766	83.68789338	101.9207494	2.084140382	11.68169441	0.1	0.726530612	1.217867308	0.284356954
Q62165	Dystroglycan OS=Mus musculus OX=10090 GN=Dag1 PE=1 SV=4 - [DAG1_MOUSE]	Dag1	872.4365973	730.1831443	899.1700697	900.9624297	1171.463717	1058.38138	833.9299371	1043.602509	90.83621195	135.8548781	0.1	0.726530612	1.251427083	0.323574232
Q8BKG3	Inactive tyrosine-protein kinase 7 OS=Mus musculus OX=10090 GN=Ptk7 PE=1 SV=1 - [PTK7_MOUSE]	Ptk7	190.7612379	209.3531126	248.474065	259.6009178	283.9259199	269.4269595	216.1961385	270.9845991	29.45866355	12.2370794	0.1	0.726530612	1.253420163	0.325870106
Q9R0B9	Procollagen-lysine,2-oxoglutarate 5-dioxygenase 2 OS=Mus musculus OX=10090 GN=Plod2 PE=1 SV=2 - [PLOD2_MOUSE]	Plod2	30.34522723	27.44361958	29.69648694	38.05597346	34.37636394	39.76665411	29.16177792	37.39966384	1.522913997	2.754426216	0.1	0.726530612	1.282489152	0.358946623
P35951	Low-density lipoprotein receptor OS=Mus musculus OX=10090 GN=Ldlr PE=1 SV=2 - [LDLR_MOUSE]	Ldlr	130.9751026	90.08035293	134.3127446	136.1888033	167.4896123	157.260523	118.4560667	153.6463129	24.63068842	15.96032726	0.1	0.726530612	1.297074242	0.375261058
Q8BWP8	Beta-1,4-glucuronyltransferase 1 OS=Mus musculus OX=10090 GN=B4gat1 PE=1 SV=1 - [B4GA1_MOUSE]	B4gat1	97.99291677	113.0996987	123.9483086	124.4541385	175.5018321	157.8951446	111.680308	152.6170384	13.0357813	25.92991629	0.1	0.726530612	1.366552807	0.45054121
P18242	Cathepsin D OS=Mus musculus OX=10090 GN=Ctsd PE=1 SV=1 - [CATD_MOUSE]	Ctsd	918.1393511	802.7526326	863.7898464	1075.174967	1429.186842	1106.469435	861.56061	1203.610415	57.72565132	195.9805589	0.1	0.726530612	1.397011888	0.482344298
Q8R2Y2	Cell surface glycoprotein MUC18 OS=Mus musculus OX=10090 GN=Mcam PE=1 SV=1 - [MUC18_MOUSE]	Mcam	45.58359226	36.37974278	40.30181928	55.45824153	68.92738307	48.56704802	40.75505144	57.65089087	4.618633549	10.35575175	0.1	0.726530612	1.414570436	0.500364015
P61961	Ubiquitin-fold modifier 1 OS=Mus musculus OX=10090 GN=Ufm1 PE=1 SV=1 - [UFM1_MOUSE]	Ufm1	74.20915547	53.85238458	66.33937761	96.53455616	89.59531721	92.67640094	64.80030589	92.93542477	10.26528551	3.476863428	0.1	0.726530612	1.434181884	0.520227999
Q8R5M8	Cell adhesion molecule 1 OS=Mus musculus OX=10090 GN=Cadm1 PE=1 SV=2 - [CADM1_MOUSE]	Cadm1	80.49727523	79.96853701	58.60051722	88.15306977	148.0056698	88.95502186	73.02210982	108.3712538	12.49226324	34.32675312	0.1	0.726530612	1.484088231	0.569576865
Q99LJ1	Tissue alpha-L-fucosidase OS=Mus musculus OX=10090 GN=Fuca1 PE=1 SV=1 - [FUCO_MOUSE]	Fuca1	59.27673666	54.98990695	63.27917162	79.37030929	95.00017183	91.56020576	59.18193841	88.64356229	4.145445358	8.212993217	0.1	0.726530612	1.497814446	0.582858909
Q9WVJ3	Carboxypeptidase Q OS=Mus musculus OX=10090 GN=Cpq PE=1 SV=1 - [CBPQ_MOUSE]	Cpq	62.05399456	52.585009	78.9229075	110.464524	101.6488281	80.0867365	64.52063702	97.40002954	13.34108197	15.62823441	0.1	0.726530612	1.509594977	0.594161527
Q62356	Follistatin-related protein 1 OS=Mus musculus OX=10090 GN=Fstl1 PE=1 SV=2 - [FSTL1_MOUSE]	Fstl1	530.989273	597.3925733	573.4908728	955.2794182	976.7602055	639.6974491	567.2909064	857.2456909	33.63300848	188.7081986	0.1	0.726530612	1.511121862	0.595620009
Q6S5C2	N-acetylglucosamine-1-phosphotransferase subunit gamma OS=Mus musculus OX=10090 GN=Gnptg PE=1 SV=1 - [GNPTG_MOUSE]	Gnptg	89.90881449	78.43090267	86.85358145	93.71247954	156.6262395	144.9621053	85.06443287	131.7669414	5.944443156	33.46818727	0.1	0.726530612	1.549025098	0.631360519
Q02596	Glycosylation-dependent cell adhesion molecule 1 OS=Mus musculus OX=10090 GN=Glycam1 PE=1 SV=1 - [GLCM1_MOUSE]	Glycam1	27.93073117	31.48564988	29.45491922	42.20656015	56.955911	39.47843542	29.62376676	46.21363552	1.783464009	9.402554328	0.1	0.726530612	1.560018883	0.641563492
Q8VCF1	Soluble calcium-activated nucleotidase 1 OS=Mus musculus OX=10090 GN=Cant1 PE=2 SV=1 - [CANT1_MOUSE]	Cant1	26.90994249	24.73423902	17.79151278	31.41563072	47.48302926	37.20347928	23.14523143	38.70071309	4.762367997	8.137665867	0.1	0.726530612	1.672081491	0.741645161
Q8BJU0	Small glutamine-rich tetratricopeptide repeat-containing protein alpha OS=Mus musculus OX=10090 GN=Sgta PE=1 SV=2 - [SGTA_MOUSE]	Sgta	108.2780364	75.98627731	78.87353529	160.2486122	169.0748469	115.6621361	87.71261634	148.3285317	17.86858814	28.63207392	0.1	0.726530612	1.691074077	0.757939858
Q60963	Platelet-activating factor acetylhydrolase OS=Mus musculus OX=10090 GN=Pla2g7 PE=1 SV=2 - [PAFA_MOUSE]	Pla2g7	1044.264674	712.3403569	1212.370414	1730.776963	2008.166292	1471.445706	989.6584817	1736.796321	254.4482103	268.410919	0.1	0.726530612	1.754945118	0.811425914
Q61508	Extracellular matrix protein 1 OS=Mus musculus OX=10090 GN=Ecm1 PE=1 SV=2 - [ECM1_MOUSE]	Ecm1	110.6793372	145.948576	134.395199	186.108654	240.8704443	263.4846587	130.3410374	230.1545857	17.98073891	39.78547155	0.1	0.726530612	1.765787585	0.820311805

Q06890	Clusterin OS=Mus musculus OX=10090 GN=Clu PE=1 SV=1 - [CLUS_MOUSE]	Clu	1233.187512	866.3147977	1028.302285	1729.528442	2197.290236	1598.350406	1042.601532	1841.723028	183.8538779	314.8379722	0.1	0.726530612	1.766468754	0.82086823
P25206	DNA replication licensing factor MCM3 OS=Mus musculus OX=10090 GN=Mcm3 PE=1 SV=2 - [MCM3_MOUSE]	Mcm3	54.72861166	35.27264231	48.59587544	92.30572125	97.69239127	55.04983732	46.19904314	81.68264995	9.94697389	23.22141436	0.1	0.726530612	1.768059345	0.8221667
P12025	Midkine OS=Mus musculus OX=10090 GN=Mdk PE=1 SV=2 - [MK_MOUSE]	Mdk	49.59616409	46.0032993	47.77463646	52.03747351	110.9205585	91.12065612	47.79136662	84.69289605	1.796490825	29.96316906	0.1	0.726530612	1.772137983	0.82549094
Q6PB93	Polypeptide N-acetylgalactosaminyltransferase 2 OS=Mus musculus OX=10090 GN=Galnt2 PE=1 SV=1 - [GALT2_MOUSE]	Galnt2	28.6043409	49.7970388	52.0222249	59.24941981	99.83639843	75.61558903	43.47453487	78.23380242	12.92593767	20.41976957	0.1	0.726530612	1.799531672	0.847621494
O88531	Palmitoyl-protein thioesterase 1 OS=Mus musculus OX=10090 GN=Ppt1 PE=1 SV=2 - [PPT1_MOUSE]	Ppt1	42.40594821	33.67399089	42.71856456	67.0026127	75.46630347	84.10818311	39.59950122	75.52569976	5.134022474	8.552939885	0.1	0.726530612	1.907238663	0.931485387
Q3TCN2	Putative phospholipase B-like 2 OS=Mus musculus OX=10090 GN=Plbd2 PE=1 SV=2 - [PLBL2_MOUSE]	Plbd2	30.13256347	33.32039187	20.57944881	48.15503225	63.19115926	52.13628819	28.01080139	54.4941599	6.630181995	7.790439534	0.1	0.726530612	1.945469505	0.960118366
Q9JKN6	RNA-binding protein Nova-1 OS=Mus musculus OX=10090 GN=Nova1 PE=1 SV=2 - [NOVA1_MOUSE]	Nova1	42.65010636	29.7354077	0	45.69140658	50.82284942	50.74226664	24.12850469	49.08550755	21.87089194	2.939653792	0.1	0.726530612	2.034336905	1.024558622
O09131	Glutathione S-transferase omega-1 OS=Mus musculus OX=10090 GN=Gsto1 PE=1 SV=2 - [GSTO1_MOUSE]	Gsto1	138.1525544	68.79729509	74.33778096	201.3065432	245.7492088	151.7276905	93.76254349	199.5944808	38.54256152	47.03413489	0.1	0.726530612	2.128722978	1.089988217
P29416	Beta-hexosaminidase subunit alpha OS=Mus musculus OX=10090 GN=Hexa PE=1 SV=2 - [HEXA_MOUSE]	Hexa	32.71058865	30.26463157	0	32.77882334	56.33737709	45.42872486	20.99174007	44.84830843	18.22047035	11.78999687	0.1	0.726530612	2.136474074	1.09523181
A2ASQ1	Agrin OS=Mus musculus OX=10090 GN=Agrn PE=1 SV=1 - [AGRIN_MOUSE]	Agrn	168.5643997	137.2713219	135.4766245	309.3324762	362.0946262	316.2781082	147.1041154	329.2350702	18.60680219	28.66833211	0.1	0.726530612	2.238109174	1.162280412
P01029	Complement C4-B OS=Mus musculus OX=10090 GN=C4b PE=1 SV=3 - [CO4B_MOUSE]	C4b	208.3802379	129.7443489	172.1110761	468.3563353	464.8039399	228.4829397	170.0785543	387.214405	39.3573261	137.476956	0.1	0.726530612	2.276679777	1.186931386
P54754	Ephrin type-B receptor 3 OS=Mus musculus OX=10090 GN=Ephb3 PE=1 SV=2 - [EPHB3_MOUSE]	Ephb3	0	11.81574391	11.26020436	12.33507361	25.0689011	15.71215964	7.691982757	17.70537812	6.66724119	6.596762888	0.1	0.726530612	2.301796387	1.202760221
Q99JX4	Eukaryotic translation initiation factor 3 subunit M OS=Mus musculus OX=10090 GN=Eif3m PE=1 SV=1 - [EIF3M_MOUSE]	Eif3m	45.85775791	31.00523267	0	46.9443521	87.55759451	54.0712977	25.62099686	62.8577481	23.39820477	21.68548242	0.1	0.726530612	2.453368557	1.294763979
Q9JJ61	Polypeptide N-acetylgalactosaminyltransferase 16 OS=Mus musculus OX=10090 GN=Galnt16 PE=2 SV=2 - [GLT16_MOUSE]	Galnt16	58.78815368	46.08318716	50.03873586	147.9032093	180.9403917	70.58279277	51.63669223	133.1421313	6.501472294	56.64024169	0.1	0.726530612	2.578440359	1.366498675
P48774	Glutathione S-transferase Mu 5 OS=Mus musculus OX=10090 GN=Gstm5 PE=1 SV=1 - [GSTM5_MOUSE]	Gstm5	354.96071	480.0245869	513.8024266	1534.954279	1450.021422	733.5496786	449.5959079	1239.50846	83.67855828	440.2262075	0.1	0.726530612	2.756938927	1.463067309
Q91ZJ9	Hyaluronidase-1 OS=Mus musculus OX=10090 GN=Hyal1 PE=1 SV=3 - [HYAL1_MOUSE]	Hyal1	14.27013117	20.17219246	0	25.77684497	42.11479079	29.48068205	11.48077455	32.45743927	10.37134128	8.566091934	0.1	0.726530612	2.827112329	1.499329207
Q4KML4	Costars family protein ABRACL OS=Mus musculus OX=10090 GN=Abracl PE=1 SV=1 - [ABRAL_MOUSE]	Abracl	0	23.34144585	28.47692057	56.43099154	56.92063721	36.71563361	17.27278881	50.02242079	15.17745697	11.52661602	0.1	0.726530612	2.896024571	1.534073843
Q924M7	Mannose-6-phosphate isomerase OS=Mus musculus OX=10090 GN=Mpi PE=1 SV=1 - [MPI_MOUSE]	Mpi	35.6627699	26.75045106	0	71.15229144	68.91489163	41.71898769	20.80440699	60.59539025	18.5600342	16.38567727	0.1	0.726530612	2.912622806	1.54231888
Q9R0P3	S-formylglutathione hydrolase OS=Mus musculus OX=10090 GN=Esd PE=1 SV=1 - [ESTD_MOUSE]	Esd	255.2638078	72.95152322	98.78779963	435.7435232	648.107213	270.4492861	142.3343769	451.4333408	98.64922997	189.3172079	0.1	0.726530612	3.171639562	1.665228827
P28798	Progranulin OS=Mus musculus OX=10090 GN=Grn PE=1 SV=2 - [GRN_MOUSE]	Grn	0	26.9673713	25.44869375	31.25418793	77.31362709	58.64129514	17.47202168	55.73637005	15.15025583	23.16672023	0.1	0.726530612	3.190035536	1.673572495
Q9R1E6	Ectonucleotide pyrophosphatase/phosphodiesterase family member 2 OS=Mus musculus OX=10090 GN=Enpp2 PE=1 SV=3 - [ENPP2_MOUSE]	Enpp2	23.30041514	0	21.46718344	31.82883387	82.20223476	31.82787893	14.92253286	48.61964919	12.95575837	29.08337223	0.1	0.726530612	3.258136514	1.704047053
P19157	Glutathione S-transferase P 1 OS=Mus musculus OX=10090 GN=Gstp1 PE=1 SV=2 - [GSTP1_MOUSE]	Gstp1	461.8797843	167.7693915	139.2713546	923.2177693	1175.921644	526.9904512	256.3068435	875.3766214	178.6007	327.1001447	0.1	0.726530612	3.415346268	1.772031855
O88998	Noelin OS=Mus musculus OX=10090 GN=Olfm1 PE=1 SV=1 - [NOE1_MOUSE]	Olfm1	38.09723742	22.84727349	0	82.68738161	81.62757314	62.73620965	20.31483697	75.68372147	19.17445699	11.22538842	0.1	0.726530612	3.725539199	1.897449243
P61922	4-aminobutyrate aminotransferase, mitochondrial OS=Mus musculus OX=10090 GN=Abat PE=1 SV=1 - [GABT_MOUSE]	Abat	5.214383309	8.18244564	0	18.49336204	32.20334394	11.48823846	4.46560965	20.72831481	4.14229413	10.53684769	0.1	0.726530612	4.641765949	2.21467378
P14901	Heme oxygenase 1 OS=Mus musculus OX=10090 GN=Hmox1 PE=1 SV=1 - [HMOX1_MOUSE]	Hmox1	203.4832244	136.849295	138.7883142	1027.048524	1096.695179	414.8227594	159.7069446	846.1888208	37.9237651	375.1935146	0.1	0.726530612	5.298384633	2.405552579
P50396	Rab GDP dissociation inhibitor alpha OS=Mus musculus OX=10090 GN=Gdi1 PE=1 SV=3 - [GDIA_MOUSE]	Gdi1	1389.913071	630.7962098	635.7133491	5299.438458	1440.994196	8287.690947	885.4742099	5009.374534	436.8637864	3432.552536	0.1	0.726530612	5.657278866	2.500108288
Q07797	Galectin-3-binding protein OS=Mus musculus OX=10090 GN=Lgals3bp PE=1 SV=1 - [LG3BP_MOUSE]	Lgals3bp	66.39048415	37.96507622	46.53989791	1223.071306	629.6656362	221.5932837	50.29848609	691.4434088	14.58067875	503.5890461	0.1	0.726530612	13.74680358	3.781024296
Q922Q9	Chitinase domain-containing protein 1 OS=Mus musculus OX=10090 GN=Chid1 PE=1 SV=1 - [CHID1_MOUSE]	Chid1	27.74684425	43.68243555	8.233212248	0	0	10.06174411	26.55416402	3.353914704	17.75468172	5.809150672	0.184038627	0.726530612	0.126304662	-2.985020207
Q01705	Neurogenic locus notch homolog protein 1 OS=Mus musculus OX=10090 GN=Notch1 PE=1 SV=3 - [NOTC1_MOUSE]	Notch1	26.69937661	24.57117969	30.76676815	0	0	26.47319284	27.34577482	8.824397612	3.147967925	15.28430501	0.184038627	0.726530612	0.322696931	-1.631748237
Q02788	Collagen alpha-2(VI) chain OS=Mus musculus OX=10090 GN=Col6a2 PE=1 SV=3 - [CO6A2_MOUSE]	Col6a2	20.79909717	33.66535546	28.73193604	0	28.08855456	0	27.73212956	9.36285152	6.491137063	16.21693454	0.184038627	0.726530612	0.337617474	-1.566538521
Q99PL5	Ribosome-binding protein 1 OS=Mus musculus OX=10090 GN=Rrbp1 PE=1 SV=2 - [RRBP1_MOUSE]	Rrbp1	157.6821246	0	0	176.9859444	160.9029865	8.59594208	52.5607082	115.4949577	91.03781709	92.92585772	0.184038627	0.726530612	2.197363042	1.135773247
Q9JMA1	Ubiquitin carboxyl-terminal hydrolase 14 OS=Mus musculus OX=10090 GN=Usp14 PE=1 SV=3 - [UBP14_MOUSE]	Usp14	128.3649657	0	0	143.5429643	141.6799706	27.86652656	42.78832191	104.3631538	74.11154752	66.25457096	0.184038627	0.726530612	2.439056947	1.286323443
Q9WVA3	Mitotic checkpoint protein BUB3 OS=Mus musculus OX=10090 GN=Bub3 PE=1 SV=2 - [BUB3_MOUSE]	Bub3	135.6663215	0	0	140.4332075	154.917076	44.80352147	45.22210717	113.3846017	78.32698725	59.83284241	0.184038627	0.726530612	2.507282583	1.326124604
P47754	F-actin-capping protein subunit alpha-2 OS=Mus musculus OX=10090 GN=Capza2 PE=1 SV=3 - [CAZA2_MOUSE]	Capza2	386.5210282	0	0	391.8845905	430.5496536	176.1432946	128.8403427	332.8591796	223.1580197	137.0899275	0.184038627	0.726530612	2.583501196	1.369327552
Q9D2R0	Acetoacetyl-CoA synthetase OS=Mus musculus OX=10090 GN=Aacs PE=1 SV=1 - [AACS_MOUSE]	Aacs	30.55869027	0	0	31.61347512	32.61443018	14.79852212	10.18623009	26.34214247	17.64306805	10.0095882	0.184038627	0.726530612	2.586054138	1.370752478
P55264	Adenosine kinase OS=Mus musculus OX=10090 GN=Adk PE=1 SV=2 - [ADK_MOUSE]	Adk	86.30342497	0	0	88.06026047	93.02485829	45.64994777	28.76780832	75.57835551	49.82730564	26.03735778	0.184038627	0.726530612	2.627185035	1.393517814
Q8C878	NEDD8-activating enzyme E1 catalytic subunit OS=Mus musculus OX=10090 GN=Uba3 PE=1 SV=2 - [UBA3_MOUSE]	Uba3	65.2750289	0	0	70.14999706	72.58450451	31.62413054	21.75834297	58.11954404	37.68655551	22.97796573	0.184038627	0.726530612	2.671138336	1.417454694
P61087	Ubiquitin-conjugating enzyme E2 K OS=Mus musculus OX=10090 GN=Ube2k PE=1 SV=3 - [UBE2K_MOUSE]	Ube2k	194.8255647	0	0	221.6176287	211.0762622	91.92523792	64.9418549	174.8730429	112.4825922	72.02800754	0.184038627	0.726530612	2.692763291	1.429087415
Q99LD4	COP9 signalosome complex subunit 1 OS=Mus musculus OX=10090 GN=Gps1 PE=1 SV=1 - [CSN1_MOUSE]	Gps1	49.17227144	0	0	56.12116006	54.65483892	23.61970733	16.39075715	44.79856877	28.38962415	18.35607947	0.184038627	0.726530612	2.733160425	1.450570142
P28658	Ataxin-10 OS=Mus musculus OX=10090 GN=Atxn10 PE=1 SV=2 - [ATX10_MOUSE]	Atxn10	74.93821142	0	0	92.01546543	84.24841037	28.79830581	24.97940381	68.35406054	43.26559653	34.47571733	0.184038627	0.726530612	2.736416812	1.452287999
Q9CZ30	Obg-like ATPase 1 OS=Mus musculus OX=10090 GN=Ola1 PE=1 SV=1 - [OLA1_MOUSE]	Ola1	135.6165339	0	0	162.3002023	170.2948346	43.17830993	45.20551131	125.2577823	78.29824237	71.19521314	0.184038627	0.726530612	2.770852019	1.470329664
Q9R190	Metastasis-associated protein MTA2 OS=Mus musculus OX=10090 GN=Mta2 PE=1 SV=1 - [MTA2_MOUSE]	Mta2	114.4575037	0	0	135.0017389	136.6818987	45.48138761	38.15250123	105.7216751	66.08207057	52.17638267	0.184038627	0.726530612	2.771028679	1.470421642
Q921F2	TAR DNA-binding protein 43 OS=Mus musculus OX=10090 GN=Tardbp PE=1 SV=1 - [TADBP_MOUSE]	Tardbp	193.5676673	0	0	219.9974445	40.32201099	276.2475789	64.52255575	178.8556781	111.7563448	123.2262233	0.184038627	0.726530612	2.771986882	1.47092043
Q8K2B3	Succinate dehydrogenase [ubiquinone] flavoprotein subunit, mitochondrial OS=Mus musculus OX=10090 GN=Sdha PE=1 SV=1 - [SDHA_MOUSE]	Sdha	35.35787681	0	0	44.52553759	38.06849977	15.68378227	11.78595894	32.75927321	20.4138797	15.13613648	0.184038627	0.726530612	2.779516998	1.474834205
O88544	COP9 signalosome complex subunit 4 OS=Mus musculus OX=10090 GN=Cops4 PE=1 SV=1 - [CSN4_MOUSE]	Cops4	74.80392542	0	0	81.80263126	94.04695689	33.12799533	24.93464181	69.65919449	43.18806648	32.22386168	0.184038627	0.726530612	2.793671352	1.482162312
Q501J6	Probable ATP-dependent RNA helicase DDX17 OS=Mus musculus OX=10090 GN=Ddx17 PE=1 SV=1 - [DDX17_MOUSE]	Ddx17	189.8937474	0	0	195.0198061	259.4830736	82.99480428	63.29791579	179.1658947	109.6352062	89.30586309	0.184038627	0.726530612	2.830518074	1.501066136
Q9WTU6	Mitogen-activated protein kinase 9 OS=Mus musculus OX=10090 GN=Mapk9 PE=1 SV=2 - [MK09_MOUSE]	Mapk9	31.48611089	0	0	32.30935219	39.23237203	17.68121676	10.4953703	29.74098033	18.1785146	11.00274847	0.184038627	0.726530612	2.833723774	1.502699134
Q9JII5	DAZ-associated protein 1 OS=Mus musculus OX=10090 GN=Dazap1 PE=1 SV=2 - [DAZP1_MOUSE]	Dazap1	114.430866	0	0	120.2403237	153.3365813	52.48337449	38.14362201	108.6867598	66.06669131	51.40968741	0.184038627	0.726530612	2.849408475	1.510662453
Q62422	Osteoclast-stimulating factor 1 OS=Mus musculus OX=10090 GN=Ostf1 PE=1 SV=2 - [OSTF1_MOUSE]	Ostf1	22.99064471	0	0	29.64273931	27.10608568	9.746817304	7.663548236	22.1652141	13.27365491	10.82917758	0.184038627	0.726530612	2.892291327	1.532212876
Q8K3X4	Probable E3 ubiquitin-protein ligase IRF2BPL OS=Mus musculus OX=10090 GN=Irf2bpl PE=1 SV=1 - [I2BPL_MOUSE]	Irf2bpl	28.61049028	0	0	29.52742024	42.65435751	11.73372636	9.536830095	27.9718347	16.51827427	15.51889953	0.184038627	0.726530612	2.933032719	1.552393165
Q8BM88	Cathepsin O OS=Mus musculus OX=10090 GN=Ctso PE=2 SV=1 - [CATO_MOUSE]	Ctso	0	44.15519258	0	32.35363291	47.47420514	51.76173396	14.71839753	43.86319067	25.49301232	10.19549732	0.184038627	0.726530612	2.980160754	1.575390154
Q9D8W5	26S proteasome non-ATPase regulatory subunit 12 OS=Mus musculus OX=10090 GN=Psmd12 PE=1 SV=4 - [PSD12_MOUSE]	Psmd12	62.71102037	0	0	83.94538998	71.31877366	31.85541817	20.90367346	62.37319394	36.20622449	27.17275912	0.184038627	0.726530612	2.983838897	1.577169644
Q3UMY5	Echinoderm microtubule-associated protein-like 4 OS=Mus musculus OX=10090 GN=Eml4 PE=1 SV=1 - [EMAL4_MOUSE]	Eml4	28.17176435	0	0	31.11452305	35.8514353	17.88742336	9.390588117	28.28446057	16.26497573	9.310390509	0.184038627	0.726530612	3.012000976	1.590722237
Q8BP47	Asparagine--tRNA ligase, cytoplasmic OS=Mus musculus OX=10090 GN=NARS1 PE=1 SV=2 - [SYNC_MOUSE]	NARS1	47.75772314	0	0	55.59658805	61.61633859	26.96462463	15.91924105	48.05918376	27.57293431	18.5147149	0.184038627	0.726530612	3.018936871	1.594040589
Q9EQS9	Immunoglobulin superfamily DCC subclass member 4 OS=Mus musculus OX=10090 GN=Igdcc4 PE=2 SV=1 - [IGDC4_MOUSE]	Igdcc4	16.13013514	0	0	17.40247314	12.30114608	19.65734174	5.376711714	16.45365366	9.312737866	3.768766038	0.184038627	0.726530612	3.060170329	1.613611956
Q6PEB6	MOB-like protein phocein OS=Mus musculus OX=10090 GN=Mob4 PE=1 SV=1 - [PHOCN_MOUSE]	Mob4	35.48039885	0	0	38.98293838	46.72550261	22.94272827	11.82679962	36.21705642	20.48461783	12.13023768	0.184038627	0.726530612	3.062287144	1.614609567
Q9WUA2	Phenylalanine--tRNA ligase beta subunit OS=Mus musculus OX=10090 GN=Farsb PE=1 SV=2 - [SYFB_MOUSE]	Farsb	40.33262616	0	0	55.80229138	44.49605766	24.86096381	13.44420872	41.71977095	23.28605257	15.65638093	0.184038627	0.726530612	3.103177867	1.633746391
Q9D1M0	Protein SEC13 homolog OS=Mus musculus OX=10090 GN=Sec13 PE=1 SV=3 - [SEC13_MOUSE]	Sec13	73.96311492	0	0	108.6167845	82.42429051	38.93849047	24.65437164	76.65985518	42.70262431	35.19499542	0.184038627	0.726530612	3.109381829	1.636627789
Q6P2B1	Transportin-3 OS=Mus musculus OX=10090 GN=Tnpo3 PE=1 SV=1 - [TNPO3_MOUSE]	Tnpo3	21.54522099	0	0	23.55624953	30.50676088	12.9491815	7.181740331	22.3373973	12.43913914	8.842021773	0.184038627	0.726530612	3.110304226	1.637055701
Q3UPH1	Protein PRRC1 OS=Mus musculus OX=10090 GN=Prrc1 PE=1 SV=1 - [PRRC1_MOUSE]	Prrc1	34.07924288	0	0	43.02698291	40.38321254	24.55386814	11.35974763	35.9880212	19.67566005	9.99010873	0.184038627	0.726530612	3.168029993	1.663585994
Q8VCA8	Secernin-2 OS=Mus musculus OX=10090 GN=Scrn2 PE=1 SV=1 - [SCRN2_MOUSE]	Scrn2	27.49617756	0	0	36.34729292	33.01520493	18.07807766	9.16539252	29.1468585	15.87492552	9.729549559	0.184038627	0.726530612	3.180099318	1.669071823
Q8BHJ5	F-box-like/WD repeat-containing protein TBL1XR1 OS=Mus musculus OX=10090 GN=Tbl1xr1 PE=1 SV=1 - [TBL1R_MOUSE]	Tbl1xr1	71.37909243	0	0	102.6925017	103.2096518	21.34374549	23.79303081	75.74863297	41.21073823	47.11672419	0.184038627	0.726530612	3.183647917	1.670680795
Q8CGF7	Transcription elongation regulator 1 OS=Mus musculus OX=10090 GN=Tcerg1 PE=1 SV=2 - [TCRG1_MOUSE]	Tcerg1	37.96285715	0	0	49.68576992	52.78979649	20.15509193	12.65428572	40.87688612	21.9178658	18.01258757	0.184038627	0.726530612	3.230280004	1.691659224
Q8BMJ2	Leucine--tRNA ligase, cytoplasmic OS=Mus musculus OX=10090 GN=Lars1 PE=1 SV=2 - [SYLC_MOUSE]	Lars1	25.63610476	0	0	38.11537475	36.25385956	8.482136766	8.545368254	27.61712369	14.80101199	16.59750292	0.184038627	0.726530612	3.231823705	1.692348502
Q9ERK4	Exportin-2 OS=Mus musculus OX=10090 GN=Cse1l PE=1 SV=1 - [XPO2_MOUSE]	Cse1l	114.4780556	0	0	145.0466682	180.530728	46.07754298	38.15935188	123.8849797	66.09393624	69.67982157	0.184038627	0.726530612	3.246516872	1.698892707
Q923D2	Flavin reductase (NADPH) OS=Mus musculus OX=10090 GN=Blvrb PE=1 SV=3 - [BLVRB_MOUSE]	Blvrb	143.0898596	0	0	190.6275024	189.9790867	89.06660355	47.69661987	156.5577309	82.61296896	58.44992997	0.184038627	0.726530612	3.282365319	1.714735816
P32921	Tryptophan--tRNA ligase, cytoplasmic OS=Mus musculus OX=10090 GN=Wars1 PE=1 SV=2 - [SYWC_MOUSE]	Wars1	71.23392593	0	0	94.18537132	102.0517537	38.23618258	23.74464198	78.15776919	41.12692631	34.79611762	0.184038627	0.726530612	3.291596027	1.718787287
Q6PHZ2	Calcium/calmodulin-dependent protein kinase type II subunit delta OS=Mus musculus OX=10090 GN=Camk2d PE=1 SV=1 - [KCC2D_MOUSE]	Camk2d	50.81965375	0	0	57.78398781	75.4053316	34.73251643	16.93988458	55.97394528	29.34074077	20.3967317	0.184038627	0.726530612	3.304269578	1.724331394
Q8VEH3	ADP-ribosylation factor-like protein 8A OS=Mus musculus OX=10090 GN=Arl8a PE=1 SV=1 - [ARL8A_MOUSE]	Arl8a	49.46264685	0	0	64.51510569	56.69609402	42.85355117	16.48754895	54.68825029	28.55727247	10.96947189	0.184038627	0.726530612	3.316942407	1.729853962
Q8K2K6	Arf-GAP domain and FG repeat-containing protein 1 OS=Mus musculus OX=10090 GN=Agfg1 PE=1 SV=2 - [AGFG1_MOUSE]	Agfg1	28.67658855	0	0	36.30702074	28.37199975	30.6429974	9.558862851	31.77400597	16.55643612	4.086627558	0.184038627	0.726530612	3.324036181	1.732936085
Q8R1B4	Eukaryotic translation initiation factor 3 subunit C OS=Mus musculus OX=10090 GN=Eif3c PE=1 SV=1 - [EIF3C_MOUSE]	Eif3c	35.83213602	0	0	36.82310315	55.01468669	27.3989794	11.94404534	39.74558975	20.68769338	14.03789579	0.184038627	0.726530612	3.327648934	1.734503238
Q9R1C7	Pre-mRNA-processing factor 40 homolog A OS=Mus musculus OX=10090 GN=Prpf40a PE=1 SV=1 - [PR40A_MOUSE]	Prpf40a	36.94876781	0	0	48.33098377	42.86187005	32.24283168	12.31625594	41.1452285	21.33238104	8.180299698	0.184038627	0.726530612	3.340725356	1.740161382
Q08093	Calponin-2 OS=Mus musculus OX=10090 GN=Cnn2 PE=1 SV=1 - [CNN2_MOUSE]	Cnn2	129.3665152	0	0	168.7577964	171.7033294	92.43419536	43.12217172	144.2984404	74.68979235	44.93989292	0.184038627	0.726530612	3.346270251	1.742553965
Q8BJ71	Nuclear pore complex protein Nup93 OS=Mus musculus OX=10090 GN=Nup93 PE=1 SV=1 - [NUP93_MOUSE]	Nup93	56.05802292	0	0	64.27611537	82.4427892	40.91611126	18.68600764	62.54500527	32.36511462	20.81739181	0.184038627	0.726530612	3.347157214	1.742936315
O55135	Eukaryotic translation initiation factor 6 OS=Mus musculus OX=10090 GN=Eif6 PE=1 SV=2 - [IF6_MOUSE]	Eif6	118.882864	0	0	181.6413124	177.0337239	44.79577818	39.62762135	134.4902715	68.63705356	77.71186567	0.184038627	0.726530612	3.393851736	1.762923541
Q8JZV7	N-acetylglucosamine-6-phosphate deacetylase OS=Mus musculus OX=10090 GN=Amdhd2 PE=1 SV=1 - [NAGA_MOUSE]	Amdhd2	28.8212495	0	0	29.15926186	53.27163765	15.52000394	9.607083166	32.65030115	16.63995616	19.116406	0.184038627	0.726530612	3.398565474	1.764925917
P62137	Serine/threonine-protein phosphatase PP1-alpha catalytic subunit OS=Mus musculus OX=10090 GN=Ppp1ca PE=1 SV=1 - [PP1A_MOUSE]	Ppp1ca	118.9916	0	0	141.3920014	157.6652506	106.0680947	39.66386666	135.0417822	68.69983227	26.37822183	0.184038627	0.726530612	3.404655008	1.767508618
Q921W4	Quinone oxidoreductase-like protein 1 OS=Mus musculus OX=10090 GN=Cryzl1 PE=1 SV=1 - [QORL1_MOUSE]	Cryzl1	32.53665732	0	0	35.47329207	48.44925148	26.8904647	10.84555244	36.93766941	18.78504786	10.85373773	0.184038627	0.726530612	3.405789573	1.767989301
Q3UGC7	Eukaryotic translation initiation factor 3 subunit J-A OS=Mus musculus OX=10090 GN=Eif3j1 PE=2 SV=1 - [EI3JA_MOUSE]	Eif3j1	62.81083064	0	0	76.62779244	108.2292232	30.15704597	20.93694355	71.67135387	36.26384998	39.27137542	0.184038627	0.726530612	3.423200417	1.77534576
O70310	Glycylpeptide N-tetradecanoyltransferase 1 OS=Mus musculus OX=10090 GN=Nmt1 PE=1 SV=1 - [NMT1_MOUSE]	Nmt1	44.96223092	0	0	71.50037644	65.15541364	17.79845169	14.98741031	51.48474725	25.95895613	29.34517882	0.184038627	0.726530612	3.435199691	1.780393966
Q8K2F8	Protein LSM14 homolog A OS=Mus musculus OX=10090 GN=Lsm14a PE=1 SV=1 - [LS14A_MOUSE]	Lsm14a	56.83900554	0	0	74.70615785	81.72087694	42.40587181	18.94633518	66.27763553	32.81601515	20.96896274	0.184038627	0.726530612	3.498177083	1.806603322
Q64152	Transcription factor BTF3 OS=Mus musculus OX=10090 GN=Btf3 PE=1 SV=3 - [BTF3_MOUSE]	Btf3	32.21571782	0	0	51.31248323	50.85159692	10.79826424	10.73857261	37.6541148	18.59975336	23.25899043	0.184038627	0.726530612	3.506435741	1.810005289
Q78PY7	Staphylococcal nuclease domain-containing protein 1 OS=Mus musculus OX=10090 GN=Snd1 PE=1 SV=1 - [SND1_MOUSE]	Snd1	63.73870974	0	0	101.2467157	103.9127216	23.50389866	21.24623658	76.221112	36.79956123	45.6739021	0.184038627	0.726530612	3.587511214	1.842983342
Q61191	Host cell factor 1 OS=Mus musculus OX=10090 GN=Hcfc1 PE=1 SV=2 - [HCFC1_MOUSE]	Hcfc1	147.1263374	0	0	281.883788	226.7831395	25.30059157	49.04211246	177.989173	84.9434305	135.0717311	0.184038627	0.726530612	3.629312933	1.859696457
O35286	Pre-mRNA-splicing factor ATP-dependent RNA helicase DHX15 OS=Mus musculus OX=10090 GN=Dhx15 PE=1 SV=2 - [DHX15_MOUSE]	Dhx15	77.06717835	0	0	107.0150178	130.6413587	49.36758799	25.68905945	95.67465485	44.49475616	41.80680957	0.184038627	0.726530612	3.724334674	1.896982721
P51174	Long-chain specific acyl-CoA dehydrogenase, mitochondrial OS=Mus musculus OX=10090 GN=Acadl PE=1 SV=2 - [ACADL_MOUSE]	Acadl	25.62001536	0	0	43.92387077	34.75669371	17.7774627	8.540005119	32.15267573	14.79172276	13.26628606	0.184038627	0.726530612	3.764948063	1.912629963
Q6P9R2	Serine/threonine-protein kinase OSR1 OS=Mus musculus OX=10090 GN=Oxsr1 PE=1 SV=1 - [OXSR1_MOUSE]	Oxsr1	35.09508804	0	0	51.03751004	48.20081818	33.37797625	11.69836268	44.20543482	20.26215853	9.483517257	0.184038627	0.726530612	3.778771101	1.91791713
Q64337	Sequestosome-1 OS=Mus musculus OX=10090 GN=Sqstm1 PE=1 SV=1 - [SQSTM_MOUSE]	Sqstm1	26.9809216	0	0	25.76611074	41.27450674	34.97918018	8.993640534	34.00659922	15.57744235	7.799809096	0.184038627	0.726530612	3.781182836	1.918837611
Q8BFR4	N-acetylglucosamine-6-sulfatase OS=Mus musculus OX=10090 GN=Gns PE=1 SV=1 - [GNS_MOUSE]	Gns	0	0	54.72263049	100.3397969	66.24120872	42.49401477	18.24087683	69.69167347	31.59412544	29.07684488	0.184038627	0.726530612	3.820631767	1.933811217
O35864	COP9 signalosome complex subunit 5 OS=Mus musculus OX=10090 GN=Cops5 PE=1 SV=3 - [CSN5_MOUSE]	Cops5	25.09148383	0	0	38.75307927	43.59378336	17.53809711	8.363827944	33.29498658	14.48657494	13.85885199	0.184038627	0.726530612	3.980831122	1.99306967
Q9DB29	Isoamyl acetate-hydrolyzing esterase 1 homolog OS=Mus musculus OX=10090 GN=Iah1 PE=1 SV=1 - [IAH1_MOUSE]	Iah1	26.60270844	0	0	52.83216158	37.88721426	15.93833921	8.86756948	35.55257168	15.35908088	18.5573826	0.184038627	0.726530612	4.009280307	2.003343286
Q9JHK4	Geranylgeranyl transferase type-2 subunit alpha OS=Mus musculus OX=10090 GN=Rabggta PE=1 SV=1 - [PGTA_MOUSE]	Rabggta	13.77866398	0	0	23.0491686	24.47281704	9.876599744	4.592887995	19.1328618	7.955115361	8.047700547	0.184038627	0.726530612	4.165758411	2.058579174
Q8K406	Leucine-rich repeat LGI family member 3 OS=Mus musculus OX=10090 GN=Lgi3 PE=1 SV=1 - [LGI3_MOUSE]	Lgi3	8.373706199	0	0	13.90847498	14.7994102	6.783414059	2.7912354	11.83043308	4.834561528	4.393488605	0.184038627	0.726530612	4.23842184	2.083527183
P27659	60S ribosomal protein L3 OS=Mus musculus OX=10090 GN=Rpl3 PE=1 SV=3 - [RL3_MOUSE]	Rpl3	53.04954024	0	0	77.51507999	124.7627327	29.41406345	17.68318008	77.23062538	30.62816634	47.67497108	0.184038627	0.726530612	4.367462472	2.126795307
Q9ESJ0	Exportin-4 OS=Mus musculus OX=10090 GN=Xpo4 PE=1 SV=2 - [XPO4_MOUSE]	Xpo4	4.525550835	0	0	7.730920065	9.945658801	2.110611543	1.508516945	6.595730136	2.612827993	4.03899536	0.184038627	0.726530612	4.372327509	2.12840147

P48758	Carbonyl reductase [NADPH] 1 OS=Mus musculus OX=10090 GN=Cbr1 PE=1 SV=3 - [CBR1_MOUSE]	Cbr1	104.063314	0	0	205.4645305	208.5023416	43.92574368	34.68777134	152.6308719	60.08098237	94.15365502	0.184038627	0.726530612	4.400134861	2.137547742
Q91YR9	Prostaglandin reductase 1 OS=Mus musculus OX=10090 GN=Ptgr1 PE=1 SV=2 - [PTGR1_MOUSE]	Ptgr1	122.5314153	0	0	238.3587148	243.2265855	74.97175497	40.84380511	185.5190184	70.74354562	95.76767269	0.184038627	0.726530612	4.542158056	2.183377909
Q99LI8	Hepatocyte growth factor-regulated tyrosine kinase substrate OS=Mus musculus OX=10090 GN=Hgs PE=1 SV=2 - [HGS_MOUSE]	Hgs	23.84504525	0	0	45.79918625	41.07620258	21.5036323	7.948348416	36.12634038	13.76694329	12.88193841	0.184038627	0.726530612	4.545137995	2.184324097
P24472	Glutathione S-transferase A4 OS=Mus musculus OX=10090 GN=Gsta4 PE=1 SV=3 - [GSTA4_MOUSE]	Gsta4	222.6043369	0	0	371.707991	469.9029225	186.1476562	74.20144563	342.5861899	128.5206738	144.1017775	0.184038627	0.726530612	4.616974601	2.206947796
Q9DC50	Peroxisomal carnitine O-octanoyltransferase OS=Mus musculus OX=10090 GN=Crot PE=1 SV=1 - [OCTC_MOUSE]	Crot	39.41284561	0	0	74.6577876	79.45589002	30.02145577	13.1376152	61.3783778	22.75501702	27.2616556	0.184038627	0.726530612	4.671957341	2.224027101
Q8BIJ6	Isoleucine--tRNA ligase, mitochondrial OS=Mus musculus OX=10090 GN=Iars2 PE=1 SV=1 - [SYIM_MOUSE]	Iars2	10.90989449	0	0	17.26044957	28.65104174	5.543030777	3.636631496	17.15150736	6.298830519	11.55439068	0.184038627	0.726530612	4.716317115	2.237660725
Q8BFQ4	WD repeat-containing protein 82 OS=Mus musculus OX=10090 GN=Wdr82 PE=1 SV=1 - [WDR82_MOUSE]	Wdr82	30.2457347	0	0	43.35406554	83.86243465	18.51537183	10.08191157	48.57729067	17.46238307	32.9851666	0.184038627	0.726530612	4.818261929	2.268512823
Q64442	Sorbitol dehydrogenase OS=Mus musculus OX=10090 GN=Sord PE=1 SV=3 - [DHSO_MOUSE]	Sord	33.01022829	0	0	66.25054197	68.86691614	31.3991	11.00340943	55.50551937	19.05846419	20.91771844	0.184038627	0.726530612	5.044392806	2.334680623
Q9D358	Low molecular weight phosphotyrosine protein phosphatase OS=Mus musculus OX=10090 GN=Acp1 PE=1 SV=3 - [PPAC_MOUSE]	Acp1	70.37435998	0	0	156.1436957	175.8184195	27.79037496	23.45811999	119.9174967	40.63065568	80.38860997	0.184038627	0.726530612	5.111982407	2.353882871
Q3UDE2	Tubulin--tyrosine ligase-like protein 12 OS=Mus musculus OX=10090 GN=Ttll12 PE=1 SV=1 - [TTL12_MOUSE]	Ttll12	47.42217209	0	0	96.09403888	125.1933814	26.63320022	15.8073907	82.64020682	27.37920382	50.63873542	0.184038627	0.726530612	5.227947383	2.386244621
Q8BU30	Isoleucine--tRNA ligase, cytoplasmic OS=Mus musculus OX=10090 GN=Iars1 PE=1 SV=2 - [SYIC_MOUSE]	Iars1	28.79338184	0	0	81.194342	75.64747978	8.482356626	9.597793945	55.10805947	16.62386675	40.4741775	0.184038627	0.726530612	5.741742299	2.521488581
Q8K4Z5	Splicing factor 3A subunit 1 OS=Mus musculus OX=10090 GN=Sf3a1 PE=1 SV=1 - [SF3A1_MOUSE]	Sf3a1	62.98835296	0	0	167.653631	176.4839419	19.64002361	20.99611765	121.2591988	36.36634254	88.11547071	0.184038627	0.726530612	5.775315268	2.529899704
Q80V70	Multiple epidermal growth factor-like domains protein 6 OS=Mus musculus OX=10090 GN=Megf6 PE=2 SV=3 - [MEGF6_MOUSE]	Megf6	6.912230475	0	0	3.595316007	35.00778701	7.039340224	2.304076825	15.21414775	3.990778125	17.22807134	0.184038627	0.726530612	6.603142561	2.723152794
Q6ZQ58	La-related protein 1 OS=Mus musculus OX=10090 GN=Larp1 PE=1 SV=3 - [LARP1_MOUSE]	Larp1	17.64524232	0	0	42.81999043	68.81337101	8.715729917	5.881747441	40.11636378	10.1874854	30.13990401	0.184038627	0.726530612	6.82048391	2.769874101
P97823	Acyl-protein thioesterase 1 OS=Mus musculus OX=10090 GN=Lypla1 PE=1 SV=1 - [LYPA1_MOUSE]	Lypla1	26.12685505	0	0	80.75556876	99.73138985	14.62397143	8.708951685	65.03697668	15.0843468	44.67800096	0.184038627	0.726530612	7.467830691	2.900689219
Q8K354	Carbonyl reductase [NADPH] 3 OS=Mus musculus OX=10090 GN=Cbr3 PE=1 SV=1 - [CBR3_MOUSE]	Cbr3	58.06163288	0	0	201.6346714	221.7628476	36.11640302	19.35387763	153.1713074	33.52189937	101.8708685	0.184038627	0.726530612	7.914243869	2.984451521
A2A432	Cullin-4B OS=Mus musculus OX=10090 GN=Cul4b PE=1 SV=1 - [CUL4B_MOUSE]	Cul4b	0	0	0	31.02930665	48.47986942	0	0	26.50305869	0	24.55482896	0.196705602	0.726530612	only in a1H	only in a1H
A2A690	Protein TANC2 OS=Mus musculus OX=10090 GN=Tanc2 PE=1 SV=1 - [TANC2_MOUSE]	Tanc2	0	0	0	839.0525113	0	1647.540647	0	828.8643861	0	823.8175735	0.196705602	0.726530612	only in a1H	only in a1H
A2AAY5	SH3 and PX domain-containing protein 2B OS=Mus musculus OX=10090 GN=Sh3pxd2b PE=1 SV=1 - [SPD2B_MOUSE]	Sh3pxd2b	0	0	0	0	33.7252391	19.73867574	0	17.82130495	0	16.94417809	0.196705602	0.726530612	only in a1H	only in a1H
E9Q555	E3 ubiquitin-protein ligase RNF213 OS=Mus musculus OX=10090 GN=Rnf213 PE=1 SV=2 - [RN213_MOUSE]	Rnf213	0	0	0	45.38328499	10.15195744	0	0	18.51174748	0	23.81858951	0.196705602	0.726530612	only in a1H	only in a1H
O08739	AMP deaminase 3 OS=Mus musculus OX=10090 GN=Ampd3 PE=1 SV=2 - [AMPD3_MOUSE]	Ampd3	0	0	0	13.44120638	12.41195596	0	0	8.61772078	0	7.480887159	0.196705602	0.726530612	only in a1H	only in a1H
O08756	3-hydroxyacyl-CoA dehydrogenase type-2 OS=Mus musculus OX=10090 GN=Hsd17b10 PE=1 SV=4 - [HCD2_MOUSE]	Hsd17b10	0	0	0	20.61213564	18.12673355	0	0	12.9129564	0	11.25178377	0.196705602	0.726530612	only in a1H	only in a1H
O08832	Polypeptide N-acetylgalactosaminyltransferase 4 OS=Mus musculus OX=10090 GN=Galnt4 PE=1 SV=1 - [GALT4_MOUSE]	Galnt4	0	0	0	22.09565393	30.91280891	0	0	17.66948761	0	15.92462401	0.196705602	0.726530612	only in a1H	only in a1H
O35295	Transcriptional activator protein Pur-beta OS=Mus musculus OX=10090 GN=Purb PE=1 SV=3 - [PURB_MOUSE]	Purb	0	0	0	39.11199559	46.48344507	0	0	28.53181356	0	24.98265109	0.196705602	0.726530612	only in a1H	only in a1H
O54950	5'-AMP-activated protein kinase subunit gamma-1 OS=Mus musculus OX=10090 GN=Prkag1 PE=1 SV=2 - [AAKG1_MOUSE]	Prkag1	0	0	0	17.30247148	3.770860189	0	0	7.024443889	0	9.098529276	0.196705602	0.726530612	only in a1H	only in a1H
O88587	Catechol O-methyltransferase OS=Mus musculus OX=10090 GN=Comt PE=1 SV=2 - [COMT_MOUSE]	Comt	0	0	0	27.4530615	31.38611422	0	0	19.61305857	0	17.09886768	0.196705602	0.726530612	only in a1H	only in a1H
O88746	Target of Myb protein 1 OS=Mus musculus OX=10090 GN=Tom1 PE=1 SV=1 - [TOM1_MOUSE]	Tom1	0	0	0	8.465655165	11.50732286	0	0	6.657659342	0	5.962906719	0.196705602	0.726530612	only in a1H	only in a1H
P00329	Alcohol dehydrogenase 1 OS=Mus musculus OX=10090 GN=Adh1 PE=1 SV=2 - [ADH1_MOUSE]	Adh1	0	0	0	70.86913578	0	25.51931874	0	32.12948484	0	35.89400122	0.196705602	0.726530612	only in a1H	only in a1H
P01899	H-2 class I histocompatibility antigen, D-B alpha chain OS=Mus musculus OX=10090 GN=H2-D1 PE=1 SV=2 - [HA11_MOUSE]	H2-D1	0	0	0	196.579258	41.37883964	0	0	79.31936587	0	103.636209	0.196705602	0.726530612	only in a1H	only in a1H
P05132	cAMP-dependent protein kinase catalytic subunit alpha OS=Mus musculus OX=10090 GN=Prkaca PE=1 SV=3 - [KAPCA_MOUSE]	Prkaca	0	0	0	19.51048119	0	19.67091507	0	13.06046542	0	11.31097929	0.196705602	0.726530612	only in a1H	only in a1H
P08030	Adenine phosphoribosyltransferase OS=Mus musculus OX=10090 GN=Aprt PE=1 SV=2 - [APT_MOUSE]	Aprt	0	0	0	18.82647839	20.96273234	0	0	13.26307024	0	11.53571278	0.196705602	0.726530612	only in a1H	only in a1H
P08207	Protein S100-A10 OS=Mus musculus OX=10090 GN=S100a10 PE=1 SV=2 - [S10AA_MOUSE]	S100a10	0	0	0	115.2697742	141.8510686	0	0	85.70694762	0	75.40491996	0.196705602	0.726530612	only in a1H	only in a1H
P09602	Non-histone chromosomal protein HMG-17 OS=Mus musculus OX=10090 GN=Hmgn2 PE=1 SV=2 - [HMGN2_MOUSE]	Hmgn2	0	0	0	30.89009897	42.78967446	0	0	24.55992448	0	22.08602255	0.196705602	0.726530612	only in a1H	only in a1H
P14426	H-2 class I histocompatibility antigen, D-K alpha chain OS=Mus musculus OX=10090 GN=H2-D1 PE=1 SV=1 - [HA13_MOUSE]	H2-D1	0	0	0	157.4296861	66.82091627	0	0	74.75020078	0	79.01380688	0.196705602	0.726530612	only in a1H	only in a1H
P14430	H-2 class I histocompatibility antigen, Q8 alpha chain OS=Mus musculus OX=10090 GN=H2-Q8 PE=3 SV=1 - [HA18_MOUSE]	H2-Q8	0	0	0	157.6167756	67.29089253	0	0	74.9692227	0	79.08842869	0.196705602	0.726530612	only in a1H	only in a1H
P19001	Keratin, type I cytoskeletal 19 OS=Mus musculus OX=10090 GN=Krt19 PE=1 SV=1 - [K1C19_MOUSE]	Krt19	0	0	0	94.41071504	114.1167582	0	0	69.50915775	0	60.99774006	0.196705602	0.726530612	only in a1H	only in a1H
P21126	Ubiquitin-like protein 4A OS=Mus musculus OX=10090 GN=Ubl4a PE=1 SV=1 - [UBL4A_MOUSE]	Ubl4a	0	0	0	0	33.95714882	11.71815165	0	15.22510016	0	17.2480725	0.196705602	0.726530612	only in a1H	only in a1H
P30285	Cyclin-dependent kinase 4 OS=Mus musculus OX=10090 GN=Cdk4 PE=1 SV=1 - [CDK4_MOUSE]	Cdk4	0	0	0	62.49007172	101.3032467	0	0	54.5977728	0	51.11069588	0.196705602	0.726530612	only in a1H	only in a1H
P32020	Sterol carrier protein 2 OS=Mus musculus OX=10090 GN=Scp2 PE=1 SV=3 - [SCP2_MOUSE]	Scp2	0	0	0	21.17287737	5.032497057	0	0	8.735124809	0	11.06140919	0.196705602	0.726530612	only in a1H	only in a1H
P45377	Aldose reductase-related protein 2 OS=Mus musculus OX=10090 GN=Akr1b8 PE=1 SV=2 - [ALD2_MOUSE]	Akr1b8	0	0	0	29.53455895	17.23730282	0	0	15.59062059	0	14.83597704	0.196705602	0.726530612	only in a1H	only in a1H
P49138	MAP kinase-activated protein kinase 2 OS=Mus musculus OX=10090 GN=Mapkapk2 PE=1 SV=2 - [MAPK2_MOUSE]	Mapkapk2	0	0	0	21.74994558	20.59249119	0	0	14.11414559	0	12.23690134	0.196705602	0.726530612	only in a1H	only in a1H
P49717	DNA replication licensing factor MCM4 OS=Mus musculus OX=10090 GN=Mcm4 PE=1 SV=1 - [MCM4_MOUSE]	Mcm4	0	0	0	5.918075827	10.77346928	0	0	5.563848368	0	5.395462713	0.196705602	0.726530612	only in a1H	only in a1H
P54818	Galactocerebrosidase OS=Mus musculus OX=10090 GN=Galc PE=1 SV=2 - [GALC_MOUSE]	Galc	0	0	0	19.66505453	12.55271995	0	0	10.73925816	0	9.957162002	0.196705602	0.726530612	only in a1H	only in a1H
P57784	U2 small nuclear ribonucleoprotein A' OS=Mus musculus OX=10090 GN=Snrpa1 PE=1 SV=2 - [RU2A_MOUSE]	Snrpa1	0	0	0	14.24080557	24.52631623	0	0	12.92237394	0	12.31619842	0.196705602	0.726530612	only in a1H	only in a1H
P60762	Mortality factor 4-like protein 1 OS=Mus musculus OX=10090 GN=Morf4l1 PE=1 SV=2 - [MO4L1_MOUSE]	Morf4l1	0	0	0	19.31312631	17.94222305	0	0	12.41844978	0	10.77651452	0.196705602	0.726530612	only in a1H	only in a1H
P61028	Ras-related protein Rab-8B OS=Mus musculus OX=10090 GN=Rab8b PE=1 SV=1 - [RAB8B_MOUSE]	Rab8b	0	0	0	301.0447886	339.2002185	0	0	213.4150024	0	185.8048214	0.196705602	0.726530612	only in a1H	only in a1H
P62077	Mitochondrial import inner membrane translocase subunit Tim8 B OS=Mus musculus OX=10090 GN=Timm8b PE=1 SV=1 - [TIM8B_MOUSE]	Timm8b	0	0	0	16.64812161	0	16.77834265	0	11.14215475	0	9.649608737	0.196705602	0.726530612	only in a1H	only in a1H
P67984	60S ribosomal protein L22 OS=Mus musculus OX=10090 GN=Rpl22 PE=1 SV=2 - [RL22_MOUSE]	Rpl22	0	0	0	30.96281323	26.74224192	0	0	19.23501838	0	16.79115108	0.196705602	0.726530612	only in a1H	only in a1H
P70398	Probable ubiquitin carboxyl-terminal hydrolase FAF-X OS=Mus musculus OX=10090 GN=Usp9x PE=1 SV=2 - [USP9X_MOUSE]	Usp9x	0	0	0	36.15153555	26.00566546	0	0	20.719067	0	18.64656733	0.196705602	0.726530612	only in a1H	only in a1H
P70697	Uroporphyrinogen decarboxylase OS=Mus musculus OX=10090 GN=Urod PE=1 SV=2 - [DCUP_MOUSE]	Urod	0	0	0	21.95134242	28.98320061	0	0	16.97818101	0	15.11805974	0.196705602	0.726530612	only in a1H	only in a1H
P80560	Receptor-type tyrosine-protein phosphatase N2 OS=Mus musculus OX=10090 GN=Ptprn2 PE=1 SV=2 - [PTPR2_MOUSE]	Ptprn2	0	0	0	13.40202265	47.91387148	0	0	20.43863138	0	24.71983347	0.196705602	0.726530612	only in a1H	only in a1H
P84104	Serine/arginine-rich splicing factor 3 OS=Mus musculus OX=10090 GN=Srsf3 PE=1 SV=1 - [SRSF3_MOUSE]	Srsf3	0	0	0	0	57.01364107	31.18279404	0	29.3988117	0	28.54865604	0.196705602	0.726530612	only in a1H	only in a1H
P97290	Plasma protease C1 inhibitor OS=Mus musculus OX=10090 GN=Serping1 PE=1 SV=3 - [IC1_MOUSE]	Serping1	0	0	0	23.89073618	44.97258128	0	0	22.95443915	0	22.50090567	0.196705602	0.726530612	only in a1H	only in a1H
Q04519	Sphingomyelin phosphodiesterase OS=Mus musculus OX=10090 GN=Smpd1 PE=1 SV=2 - [ASM_MOUSE]	Smpd1	0	0	0	0	7.722412577	23.92626684	0	10.54955981	0	12.21110693	0.196705602	0.726530612	only in a1H	only in a1H
Q06335	Amyloid-like protein 2 OS=Mus musculus OX=10090 GN=Aplp2 PE=1 SV=4 - [APLP2_MOUSE]	Aplp2	0	0	0	0	11.01093581	12.11530714	0	7.70874765	0	6.698768675	0.196705602	0.726530612	only in a1H	only in a1H
Q07113	Cation-independent mannose-6-phosphate receptor OS=Mus musculus OX=10090 GN=Igf2r PE=1 SV=1 - [MPRI_MOUSE]	Igf2r	0	0	0	32.0416028	35.46712991	0	0	22.5029109	0	19.56321314	0.196705602	0.726530612	only in a1H	only in a1H
Q0VGU4	Neurosecretory protein VGF OS=Mus musculus OX=10090 GN=Vgf PE=1 SV=1 - [VGF_MOUSE]	Vgf	0	0	0	0	3.60596338	18.57862962	0	7.394864333	0	9.851811887	0.196705602	0.726530612	only in a1H	only in a1H
Q148V7	RAB11-binding protein RELCH OS=Mus musculus OX=10090 GN=Relch PE=1 SV=1 - [RELCH_MOUSE]	Relch	0	0	0	8.681034558	6.402174066	0	0	5.027736208	0	4.500766581	0.196705602	0.726530612	only in a1H	only in a1H
Q3UFF7	Lysophospholipase-like protein 1 OS=Mus musculus OX=10090 GN=Lyplal1 PE=1 SV=3 - [LYPL1_MOUSE]	Lyplal1	0	0	0	10.04245278	6.622063597	0	0	5.554838791	0	5.105579403	0.196705602	0.726530612	only in a1H	only in a1H
Q5M8N0	CB1 cannabinoid receptor-interacting protein 1 OS=Mus musculus OX=10090 GN=Cnrip1 PE=1 SV=1 - [CNRP1_MOUSE]	Cnrip1	0	0	0	70.02664338	78.27911685	0	0	49.43525341	0	43.01056893	0.196705602	0.726530612	only in a1H	only in a1H
Q60739	BAG family molecular chaperone regulator 1 OS=Mus musculus OX=10090 GN=Bag1 PE=1 SV=3 - [BAG1_MOUSE]	Bag1	0	0	0	34.01743677	16.3153071	0	0	16.77758129	0	17.01342923	0.196705602	0.726530612	only in a1H	only in a1H
Q61107	Guanylate-binding protein 4 OS=Mus musculus OX=10090 GN=Gbp4 PE=1 SV=1 - [GBP4_MOUSE]	Gbp4	0	0	0	100.5890457	28.11336139	0	0	42.90080237	0	51.89932888	0.196705602	0.726530612	only in a1H	only in a1H
Q61187	Tumor susceptibility gene 101 protein OS=Mus musculus OX=10090 GN=Tsg101 PE=1 SV=2 - [TS101_MOUSE]	Tsg101	0	0	0	12.53319953	26.26753062	0	0	12.93357672	0	13.13834151	0.196705602	0.726530612	only in a1H	only in a1H
Q61211	Eukaryotic translation initiation factor 2D OS=Mus musculus OX=10090 GN=Eif2d PE=1 SV=3 - [EIF2D_MOUSE]	Eif2d	0	0	0	12.02993841	19.14837919	0	0	10.39277253	0	9.678602197	0.196705602	0.726530612	only in a1H	only in a1H
Q61699	Heat shock protein 105 kDa OS=Mus musculus OX=10090 GN=Hsph1 PE=1 SV=2 - [HS105_MOUSE]	Hsph1	0	0	0	260.0190927	238.7990602	0	0	166.2727176	0	144.3867546	0.196705602	0.726530612	only in a1H	only in a1H
Q62384	Zinc finger protein ZPR1 OS=Mus musculus OX=10090 GN=Zpr1 PE=1 SV=1 - [ZPR1_MOUSE]	Zpr1	0	0	0	60.58123753	30.86668198	0	0	30.48263984	0	30.29244463	0.196705602	0.726530612	only in a1H	only in a1H
Q64339	Ubiquitin-like protein ISG15 OS=Mus musculus OX=10090 GN=Isg15 PE=1 SV=4 - [ISG15_MOUSE]	Isg15	0	0	0	341.5189412	64.53309406	0	0	135.3506784	0	181.4390931	0.196705602	0.726530612	only in a1H	only in a1H
Q64669	NAD(P)H dehydrogenase [quinone] 1 OS=Mus musculus OX=10090 GN=Nqo1 PE=1 SV=3 - [NQO1_MOUSE]	Nqo1	0	0	0	52.03805069	26.8455822	0	0	26.2945443	0	26.02340124	0.196705602	0.726530612	only in a1H	only in a1H
Q6P1D5	Seizure 6-like protein OS=Mus musculus OX=10090 GN=Sez6l PE=1 SV=1 - [SE6L1_MOUSE]	Sez6l	0	0	0	0	19.89843411	9.339970101	0	9.746134738	0	9.955433055	0.196705602	0.726530612	only in a1H	only in a1H
Q6PB44	Tyrosine-protein phosphatase non-receptor type 23 OS=Mus musculus OX=10090 GN=Ptpn23 PE=1 SV=2 - [PTN23_MOUSE]	Ptpn23	0	0	0	22.13733277	25.50342229	0	0	15.88025169	0	13.85530349	0.196705602	0.726530612	only in a1H	only in a1H
Q6PDI5	Proteasome adapter and scaffold protein ECM29 OS=Mus musculus OX=10090 GN=Ecpas PE=1 SV=3 - [ECM29_MOUSE]	Ecpas	0	0	0	14.23534211	13.7380961	0	0	9.324479405	0	8.079062478	0.196705602	0.726530612	only in a1H	only in a1H
Q6PE55	Platelet-derived growth factor receptor-like protein OS=Mus musculus OX=10090 GN=Pdgfrl PE=2 SV=1 - [PGFRL_MOUSE]	Pdgfrl	0	0	0	51.90727585	0	15.40027957	0	22.43585181	0	26.65925316	0.196705602	0.726530612	only in a1H	only in a1H
Q78JE5	F-box only protein 22 OS=Mus musculus OX=10090 GN=Fbxo22 PE=1 SV=2 - [FBX22_MOUSE]	Fbxo22	0	0	0	11.02318341	10.24365711	0	0	7.088946838	0	6.151568155	0.196705602	0.726530612	only in a1H	only in a1H
Q7TMJ8	Phosphoinositide-3-kinase-interacting protein 1 OS=Mus musculus OX=10090 GN=Pik3ip1 PE=2 SV=1 - [P3IP1_MOUSE]	Pik3ip1	0	0	0	161.7236043	137.9743077	0	0	99.89930397	0	87.32645918	0.196705602	0.726530612	only in a1H	only in a1H
Q7TSC1	Protein PRRC2A OS=Mus musculus OX=10090 GN=Prrc2a PE=1 SV=1 - [PRC2A_MOUSE]	Prrc2a	0	0	0	10.50238875	11.07678756	0	0	7.193058769	0	6.235988641	0.196705602	0.726530612	only in a1H	only in a1H
Q80UY1	Carnosine N-methyltransferase OS=Mus musculus OX=10090 GN=Carnmt1 PE=1 SV=1 - [CARME_MOUSE]	Carnmt1	0	0	0	20.66741562	20.97505407	0	0	13.88082323	0	12.02212962	0.196705602	0.726530612	only in a1H	only in a1H
Q80W00	Serine/threonine-protein phosphatase 1 regulatory subunit 10 OS=Mus musculus OX=10090 GN=Ppp1r10 PE=1 SV=1 - [PP1RA_MOUSE]	Ppp1r10	0	0	0	5.343513053	15.18336003	0	0	6.842291027	0	7.701841144	0.196705602	0.726530612	only in a1H	only in a1H
Q80WW9	DDRGK domain-containing protein 1 OS=Mus musculus OX=10090 GN=Ddrgk1 PE=1 SV=2 - [DDRGK_MOUSE]	Ddrgk1	0	0	0	9.168124738	9.654474134	0	0	6.274199624	0	5.439055031	0.196705602	0.726530612	only in a1H	only in a1H
Q810B6	Rabankyrin-5 OS=Mus musculus OX=10090 GN=Ankfy1 PE=1 SV=2 - [ANFY1_MOUSE]	Ankfy1	0	0	0	36.31128698	36.64556471	0	0	24.31895056	0	21.06149218	0.196705602	0.726530612	only in a1H	only in a1H
Q811D0	Disks large homolog 1 OS=Mus musculus OX=10090 GN=Dlg1 PE=1 SV=1 - [DLG1_MOUSE]	Dlg1	0	0	0	0	24.98111936	20.80440586	0	15.26184174	0	13.38110959	0.196705602	0.726530612	only in a1H	only in a1H
Q8BG95	Protein phosphatase 1 regulatory subunit 12B OS=Mus musculus OX=10090 GN=Ppp1r12b PE=1 SV=2 - [MYPT2_MOUSE]	Ppp1r12b	0	0	0	35.78984896	23.01654491	0	0	19.60213129	0	18.13758494	0.196705602	0.726530612	only in a1H	only in a1H
Q8BHA3	D-aminoacyl-tRNA deacylase 2 OS=Mus musculus OX=10090 GN=Dtd2 PE=1 SV=1 - [DTD2_MOUSE]	Dtd2	0	0	0	42.70103435	35.48419374	0	0	26.0617427	0	22.85676096	0.196705602	0.726530612	only in a1H	only in a1H
Q8BJ37	Tyrosyl-DNA phosphodiesterase 1 OS=Mus musculus OX=10090 GN=Tdp1 PE=1 SV=2 - [TYDP1_MOUSE]	Tdp1	0	0	0	5.934530973	5.598894137	0	0	3.844475037	0	3.33363979	0.196705602	0.726530612	only in a1H	only in a1H
Q8BPU7	Engulfment and cell motility protein 1 OS=Mus musculus OX=10090 GN=Elmo1 PE=1 SV=2 - [ELMO1_MOUSE]	Elmo1	0	0	0	30.90558507	29.69961064	0	0	20.2017319	0	17.50560118	0.196705602	0.726530612	only in a1H	only in a1H
Q8BQB4	Four-jointed box protein 1 OS=Mus musculus OX=10090 GN=Fjx1 PE=1 SV=1 - [FJX1_MOUSE]	Fjx1	0	0	0	15.84356735	36.16109844	0	0	17.3348886	0	18.12661826	0.196705602	0.726530612	only in a1H	only in a1H
Q8BTI9	Phosphatidylinositol 4,5-bisphosphate 3-kinase catalytic subunit beta isoform OS=Mus musculus OX=10090 GN=Pik3cb PE=1 SV=2 - [PK3CB_MOUSE]	Pik3cb	0	0	0	26.59875086	79.79625258	0	0	35.46500114	0	40.63026307	0.196705602	0.726530612	only in a1H	only in a1H
Q8BTV2	Cleavage and polyadenylation specificity factor subunit 7 OS=Mus musculus OX=10090 GN=Cpsf7 PE=1 SV=2 - [CPSF7_MOUSE]	Cpsf7	0	0	0	19.44604132	16.77511122	0	0	12.07371751	0	10.54108428	0.196705602	0.726530612	only in a1H	only in a1H
Q8BTZ7	Mannose-1-phosphate guanyltransferase beta OS=Mus musculus OX=10090 GN=Gmppb PE=1 SV=1 - [GMPPB_MOUSE]	Gmppb	0	0	0	16.36102795	38.02078518	0	0	18.12727105	0	19.07183099	0.196705602	0.726530612	only in a1H	only in a1H
Q8BV13	COP9 signalosome complex subunit 7b OS=Mus musculus OX=10090 GN=Cops7b PE=1 SV=1 - [CSN7B_MOUSE]	Cops7b	0	0	0	5.850550599	5.879655269	0	0	3.910068622	0	3.386250027	0.196705602	0.726530612	only in a1H	only in a1H
Q8BV57	Soluble scavenger receptor cysteine-rich domain-containing protein SSC5D OS=Mus musculus OX=10090 GN=Ssc5d PE=1 SV=1 - [SRCRL_MOUSE]	Ssc5d	0	0	0	10.67849542	7.859701597	0	0	6.179399007	0	5.533997536	0.196705602	0.726530612	only in a1H	only in a1H
Q8BVP1	Probable E3 ubiquitin-protein ligase TRIML1 OS=Mus musculus OX=10090 GN=Triml1 PE=1 SV=1 - [TRIML_MOUSE]	Triml1	0	0	0	140.5692148	153.3493385	0	0	97.9728511	0	85.08726435	0.196705602	0.726530612	only in a1H	only in a1H
Q8C170	Unconventional myosin-IXa OS=Mus musculus OX=10090 GN=Myo9a PE=1 SV=2 - [MYO9A_MOUSE]	Myo9a	0	0	0	2243.827376	2019.824102	0	0	1421.217159	0	1235.895638	0.196705602	0.726530612	only in a1H	only in a1H
Q8C4B4	Protein unc-119 homolog B OS=Mus musculus OX=10090 GN=Unc119b PE=1 SV=1 - [U119B_MOUSE]	Unc119b	0	0	0	58.58284295	64.32885089	0	0	40.97056461	0	35.59767584	0.196705602	0.726530612	only in a1H	only in a1H
Q8C5W3	Tubulin-specific chaperone cofactor E-like protein OS=Mus musculus OX=10090 GN=Tbcel PE=1 SV=1 - [TBCEL_MOUSE]	Tbcel	0	0	0	16.22950259	19.98128742	0	0	12.07026334	0	10.62014195	0.196705602	0.726530612	only in a1H	only in a1H
Q8CHX7	Raftlin-2 OS=Mus musculus OX=10090 GN=Rftn2 PE=1 SV=3 - [RFTN2_MOUSE]	Rftn2	0	0	0	0	21.89855138	7.299517128	0	9.732689504	0	11.15019659	0.196705602	0.726530612	only in a1H	only in a1H

Q8CJH3	Plexin-B1 OS=Mus musculus OX=10090 GN=Plxnb1 PE=1 SV=2 - [PLXB1_MOUSE]	Plxnb1	0	0	0	0	22.90728937	40.57322101	0	21.16017013	0	20.34295664	0.196705602	0.726530612	only in a1H	only in a1H
Q8K114	Integrator complex subunit 9 OS=Mus musculus OX=10090 GN=Ints9 PE=1 SV=1 - [INT9_MOUSE]	Ints9	0	0	0	7.845687152	11.97794488	0	0	6.607877345	0	6.084153248	0.196705602	0.726530612	only in a1H	only in a1H
Q8R1F1	Protein Niban 2 OS=Mus musculus OX=10090 GN=Niban2 PE=1 SV=2 - [NIBA2_MOUSE]	Niban2	0	0	0	10.73559911	26.6033364	0	0	12.44631184	0	13.38391872	0.196705602	0.726530612	only in a1H	only in a1H
Q8R574	Phosphoribosyl pyrophosphate synthase-associated protein 2 OS=Mus musculus OX=10090 GN=Prpsap2 PE=1 SV=1 - [KPRB_MOUSE]	Prpsap2	0	0	0	28.11624968	24.30040309	0	0	17.47221759	0	15.25119543	0.196705602	0.726530612	only in a1H	only in a1H
Q8R5F7	Interferon-induced helicase C domain-containing protein 1 OS=Mus musculus OX=10090 GN=Ifih1 PE=1 SV=1 - [IFIH1_MOUSE]	Ifih1	0	0	0	23.15463496	11.29106259	0	0	11.48189918	0	11.57849705	0.196705602	0.726530612	only in a1H	only in a1H
Q8VE33	Ganglioside-induced differentiation-associated protein 1-like 1 OS=Mus musculus OX=10090 GN=Gdap1l1 PE=1 SV=1 - [GD1L1_MOUSE]	Gdap1l1	0	0	0	16.47210867	0	8.415656478	0	8.295921715	0	8.236707066	0.196705602	0.726530612	only in a1H	only in a1H
Q8VI47	ATP-binding cassette sub-family C member 2 OS=Mus musculus OX=10090 GN=Abcc2 PE=1 SV=2 - [MRP2_MOUSE]	Abcc2	0	0	0	33.16191858	25.87677272	0	0	19.67956377	0	17.42791612	0.196705602	0.726530612	only in a1H	only in a1H
Q8VI75	Importin-4 OS=Mus musculus OX=10090 GN=Ipo4 PE=1 SV=1 - [IPO4_MOUSE]	Ipo4	0	0	0	8.302848872	9.826594588	0	0	6.04314782	0	5.288683856	0.196705602	0.726530612	only in a1H	only in a1H
Q91VH6	Protein MEMO1 OS=Mus musculus OX=10090 GN=Memo1 PE=1 SV=1 - [MEMO1_MOUSE]	Memo1	0	0	0	38.49159147	48.94190363	0	0	29.14449836	0	25.77505761	0.196705602	0.726530612	only in a1H	only in a1H
Q91W82	Ubiquitin-conjugating enzyme E2 E2 OS=Mus musculus OX=10090 GN=Ube2e2 PE=2 SV=1 - [UB2E2_MOUSE]	Ube2e2	0	0	0	40.58388051	6.348035533	0	0	15.64397201	0	21.83056723	0.196705602	0.726530612	only in a1H	only in a1H
Q91XQ0	Dynein heavy chain 8, axonemal OS=Mus musculus OX=10090 GN=Dnah8 PE=1 SV=2 - [DYH8_MOUSE]	Dnah8	0	0	0	25.19533209	12.94515084	0	0	12.71349431	0	12.5992634	0.196705602	0.726530612	only in a1H	only in a1H
Q921I9	Exosome complex component RRP41 OS=Mus musculus OX=10090 GN=Exosc4 PE=1 SV=3 - [EXOS4_MOUSE]	Exosc4	0	0	0	25.15861026	41.85620793	0	0	22.33827273	0	21.07015101	0.196705602	0.726530612	only in a1H	only in a1H
Q925I1	ATPase family AAA domain-containing protein 3 OS=Mus musculus OX=10090 GN=Atad3 PE=1 SV=1 - [ATAD3_MOUSE]	Atad3	0	0	0	14.27696276	12.56360664	0	0	8.946856466	0	7.795420307	0.196705602	0.726530612	only in a1H	only in a1H
Q99J99	3-mercaptopyruvate sulfurtransferase OS=Mus musculus OX=10090 GN=Mpst PE=1 SV=4 - [THTM_MOUSE]	Mpst	0	0	0	10.50841878	10.27234022	0	0	6.926919666	0	6.000049609	0.196705602	0.726530612	only in a1H	only in a1H
Q99KR3	Endoribonuclease LACTB2 OS=Mus musculus OX=10090 GN=Lactb2 PE=1 SV=1 - [LACB2_MOUSE]	Lactb2	0	0	0	24.13749176	26.64546129	0	0	16.92765102	0	14.71331045	0.196705602	0.726530612	only in a1H	only in a1H
Q99LG2	Transportin-2 OS=Mus musculus OX=10090 GN=Tnpo2 PE=1 SV=1 - [TNPO2_MOUSE]	Tnpo2	0	0	0	11.54195617	0	6.038622342	0	5.860192838	0	5.773046499	0.196705602	0.726530612	only in a1H	only in a1H
Q99N28	Cell adhesion molecule 3 OS=Mus musculus OX=10090 GN=Cadm3 PE=1 SV=1 - [CADM3_MOUSE]	Cadm3	0	0	0	55.01004698	26.27683833	0	0	27.09562844	0	27.51416236	0.196705602	0.726530612	only in a1H	only in a1H
Q99PV0	Pre-mRNA-processing-splicing factor 8 OS=Mus musculus OX=10090 GN=Prpf8 PE=1 SV=2 - [PRP8_MOUSE]	Prpf8	0	0	0	79.24717191	47.38327629	0	0	42.2101494	0	39.87605165	0.196705602	0.726530612	only in a1H	only in a1H
Q9CPT3	N-acylneuraminate-9-phosphatase OS=Mus musculus OX=10090 GN=Nanp PE=1 SV=1 - [NANP_MOUSE]	Nanp	0	0	0	32.44144897	41.03392056	0	0	24.49178984	0	21.64124464	0.196705602	0.726530612	only in a1H	only in a1H
Q9CQC6	Basic leucine zipper and W2 domain-containing protein 1 OS=Mus musculus OX=10090 GN=Bzw1 PE=1 SV=1 - [BZW1_MOUSE]	Bzw1	0	0	0	19.96011618	29.47964785	0	0	16.47992134	0	15.04480729	0.196705602	0.726530612	only in a1H	only in a1H
Q9CQQ8	U6 snRNA-associated Sm-like protein LSm7 OS=Mus musculus OX=10090 GN=Lsm7 PE=1 SV=1 - [LSM7_MOUSE]	Lsm7	0	0	0	9.714769599	13.14809007	0	0	7.620953222	0	6.819539455	0.196705602	0.726530612	only in a1H	only in a1H
Q9CQR6	Serine/threonine-protein phosphatase 6 catalytic subunit OS=Mus musculus OX=10090 GN=Ppp6c PE=1 SV=1 - [PPP6_MOUSE]	Ppp6c	0	0	0	27.16174674	17.73829982	0	0	14.96668219	0	13.7913567	0.196705602	0.726530612	only in a1H	only in a1H
Q9CQV6	Microtubule-associated proteins 1A/1B light chain 3B OS=Mus musculus OX=10090 GN=Map1lc3b PE=1 SV=3 - [MLP3B_MOUSE]	Map1lc3b	0	0	0	101.5980583	78.08501033	0	0	59.89435621	0	53.18567745	0.196705602	0.726530612	only in a1H	only in a1H
Q9CRA5	Golgi phosphoprotein 3 OS=Mus musculus OX=10090 GN=Golph3 PE=1 SV=1 - [GOLP3_MOUSE]	Golph3	0	0	0	11.39219359	13.28167882	0	0	8.224624138	0	7.185114492	0.196705602	0.726530612	only in a1H	only in a1H
Q9CSN1	SNW domain-containing protein 1 OS=Mus musculus OX=10090 GN=Snw1 PE=1 SV=3 - [SNW1_MOUSE]	Snw1	0	0	0	23.17386896	18.46636581	0	0	13.88007825	0	12.24877854	0.196705602	0.726530612	only in a1H	only in a1H
Q9CYZ2	Tumor protein D54 OS=Mus musculus OX=10090 GN=Tpd52l2 PE=1 SV=1 - [TPD54_MOUSE]	Tpd52l2	0	0	0	64.04456979	67.01108526	0	0	43.68521835	0	37.86157393	0.196705602	0.726530612	only in a1H	only in a1H
Q9D071	MMS19 nucleotide excision repair protein homolog OS=Mus musculus OX=10090 GN=Mms19 PE=1 SV=1 - [MMS19_MOUSE]	Mms19	0	0	0	8.124949596	8.504224644	0	0	5.54305808	0	4.804173399	0.196705602	0.726530612	only in a1H	only in a1H
Q9D1C8	Vacuolar protein sorting-associated protein 28 homolog OS=Mus musculus OX=10090 GN=Vps28 PE=1 SV=1 - [VPS28_MOUSE]	Vps28	0	0	0	28.09951535	0	9.508030137	0	12.53584849	0	14.29235648	0.196705602	0.726530612	only in a1H	only in a1H
Q9D711	Pirin OS=Mus musculus OX=10090 GN=Pir PE=1 SV=1 - [PIR_MOUSE]	Pir	0	0	0	19.80682089	15.62555695	0	0	11.81079261	0	10.4399166	0.196705602	0.726530612	only in a1H	only in a1H
Q9D975	Sulfiredoxin-1 OS=Mus musculus OX=10090 GN=Srxn1 PE=2 SV=1 - [SRXN1_MOUSE]	Srxn1	0	0	0	30.29619584	46.91039445	0	0	25.7355301	0	23.78541635	0.196705602	0.726530612	only in a1H	only in a1H
Q9DB27	Malignant T-cell-amplified sequence 1 OS=Mus musculus OX=10090 GN=Mcts1 PE=1 SV=1 - [MCTS1_MOUSE]	Mcts1	0	0	0	18.53439016	20.56110273	0	0	13.03183096	0	11.33129978	0.196705602	0.726530612	only in a1H	only in a1H
Q9ER81	Torsin-1A-interacting protein 2, isoform IFRG15 OS=Mus musculus OX=10090 GN=Tor1aip2 PE=1 SV=1 - [IFG15_MOUSE]	Tor1aip2	0	0	0	14.29484975	11.45920537	0	0	8.584685039	0	7.568542309	0.196705602	0.726530612	only in a1H	only in a1H
Q9JHI0	Matrix metalloproteinase-19 OS=Mus musculus OX=10090 GN=Mmp19 PE=2 SV=1 - [MMP19_MOUSE]	Mmp19	0	0	0	0	17.79522684	6.441925051	0	8.079050629	0	9.009864574	0.196705602	0.726530612	only in a1H	only in a1H
Q9JHS9	Spliceosome-associated protein CWC15 homolog OS=Mus musculus OX=10090 GN=Cwc15 PE=1 SV=1 - [CWC15_MOUSE]	Cwc15	0	0	0	3.186259168	6.361381081	0	0	3.18254675	0	3.180692166	0.196705602	0.726530612	only in a1H	only in a1H
Q9JIH2	Nuclear pore complex protein Nup50 OS=Mus musculus OX=10090 GN=Nup50 PE=1 SV=3 - [NUP50_MOUSE]	Nup50	0	0	0	7.391087649	10.08925104	0	0	5.826779564	0	5.223364959	0.196705602	0.726530612	only in a1H	only in a1H
Q9JKK7	Tropomodulin-2 OS=Mus musculus OX=10090 GN=Tmod2 PE=1 SV=2 - [TMOD2_MOUSE]	Tmod2	0	0	0	61.40186253	66.57300714	0	0	42.65828989	0	37.03353151	0.196705602	0.726530612	only in a1H	only in a1H
Q9JLI8	Squamous cell carcinoma antigen recognized by T-cells 3 OS=Mus musculus OX=10090 GN=Sart3 PE=1 SV=1 - [SART3_MOUSE]	Sart3	0	0	0	65.16033215	18.56474353	0	0	27.90835856	0	33.5699952	0.196705602	0.726530612	only in a1H	only in a1H
Q9QUH0	Glutaredoxin-1 OS=Mus musculus OX=10090 GN=Glrx PE=1 SV=3 - [GLRX1_MOUSE]	Glrx	0	0	0	65.57792928	62.36063949	0	0	42.64618959	0	36.96770014	0.196705602	0.726530612	only in a1H	only in a1H
Q9QYB5	Gamma-adducin OS=Mus musculus OX=10090 GN=Add3 PE=1 SV=2 - [ADDG_MOUSE]	Add3	0	0	0	46.98298522	0	22.41516366	0	23.13271629	0	23.49971035	0.196705602	0.726530612	only in a1H	only in a1H
Q9QZQ8	Core histone macro-H2A.1 OS=Mus musculus OX=10090 GN=Macroh2a1 PE=1 SV=3 - [H2AY_MOUSE]	Macroh2a	0	0	0	0	3.903316792	1.301105597	0	1.734807463	0	1.987471629	0.196705602	0.726530612	only in a1H	only in a1H
Q9R069	Basal cell adhesion molecule OS=Mus musculus OX=10090 GN=Bcam PE=1 SV=1 - [BCAM_MOUSE]	Bcam	0	0	0	5.617650249	4.014534552	0	0	3.210728267	0	2.893799749	0.196705602	0.726530612	only in a1H	only in a1H
Q9WUB4	Dynactin subunit 6 OS=Mus musculus OX=10090 GN=Dctn6 PE=1 SV=1 - [DCTN6_MOUSE]	Dctn6	0	0	0	15.95588918	19.09460922	0	0	11.68349947	0	10.23918995	0.196705602	0.726530612	only in a1H	only in a1H
Q9Z0E6	Guanylate-binding protein 2 OS=Mus musculus OX=10090 GN=Gbp2 PE=1 SV=1 - [GBP2_MOUSE]	Gbp2	0	0	0	320.2940588	74.44193926	0	0	131.578666	0	167.6171984	0.196705602	0.726530612	only in a1H	only in a1H
Q9Z0Y1	Dynactin subunit 3 OS=Mus musculus OX=10090 GN=Dctn3 PE=1 SV=2 - [DCTN3_MOUSE]	Dctn3	0	0	0	0	18.45411592	5.089607016	0	7.847907645	0	9.531252036	0.196705602	0.726530612	only in a1H	only in a1H
Q9Z0Z4	Hephaestin OS=Mus musculus OX=10090 GN=Heph PE=1 SV=3 - [HEPH_MOUSE]	Heph	0	0	0	116.5035173	349.5105519	0	0	155.3380231	0	177.9620622	0.196705602	0.726530612	only in a1H	only in a1H
Q9Z1K5	E3 ubiquitin-protein ligase ARIH1 OS=Mus musculus OX=10090 GN=Arih1 PE=1 SV=3 - [ARI1_MOUSE]	Arih1	0	0	0	21.73888462	21.35357265	0	0	14.36415242	0	12.44121266	0.196705602	0.726530612	only in a1H	only in a1H
Q9Z1T1	AP-3 complex subunit beta-1 OS=Mus musculus OX=10090 GN=Ap3b1 PE=1 SV=2 - [AP3B1_MOUSE]	Ap3b1	0	0	0	64.31138177	70.60187345	0	0	44.97108507	0	39.07289906	0.196705602	0.726530612	only in a1H	only in a1H
Q9Z2D1	Myotubularin-related protein 2 OS=Mus musculus OX=10090 GN=Mtmr2 PE=1 SV=3 - [MTMR2_MOUSE]	Mtmr2	0	0	0	7.671166505	10.13289462	0	0	5.934687042	0	5.284922804	0.196705602	0.726530612	only in a1H	only in a1H
Q9Z2M7	Phosphomannomutase 2 OS=Mus musculus OX=10090 GN=Pmm2 PE=1 SV=1 - [PMM2_MOUSE]	Pmm2	0	0	0	70.89302376	61.47017941	0	0	44.12106772	0	38.49933716	0.196705602	0.726530612	only in a1H	only in a1H
P01831	Thy-1 membrane glycoprotein OS=Mus musculus OX=10090 GN=Thy1 PE=1 SV=1 - [THY1_MOUSE]	Thy1	88.31637982	0	51.59235143	0	0	0	46.63624375	0	44.36629338	0	0.196705602	0.726530612	only in mock	only in mock
P30115	Glutathione S-transferase A3 OS=Mus musculus OX=10090 GN=Gsta3 PE=1 SV=2 - [GSTA3_MOUSE]	Gsta3	0	82.16942681	78.81031765	0	0	0	53.65991482	0	46.50119082	0	0.196705602	0.726530612	only in mock	only in mock
P55302	Alpha-2-macroglobulin receptor-associated protein OS=Mus musculus OX=10090 GN=Lrpap1 PE=1 SV=1 - [AMRP_MOUSE]	Lrpap1	0	45.73708355	45.97305179	0	0	0	30.57004511	0	26.47469856	0	0.196705602	0.726530612	only in mock	only in mock
P58021	Transmembrane 9 superfamily member 2 OS=Mus musculus OX=10090 GN=Tm9sf2 PE=1 SV=1 - [TM9S2_MOUSE]	Tm9sf2	39.20890954	38.49597354	0	0	0	0	25.90162769	0	22.4342998	0	0.196705602	0.726530612	only in mock	only in mock
P60764	Ras-related C3 botulinum toxin substrate 3 OS=Mus musculus OX=10090 GN=Rac3 PE=1 SV=1 - [RAC3_MOUSE]	Rac3	0	210.319177	183.0221766	0	0	0	131.1137845	0	114.3652042	0	0.196705602	0.726530612	only in mock	only in mock
P62492	Ras-related protein Rab-11A OS=Mus musculus OX=10090 GN=Rab11a PE=1 SV=3 - [RB11A_MOUSE]	Rab11a	0	147.9514537	162.0109377	0	0	0	103.3207971	0	89.75415094	0	0.196705602	0.726530612	only in mock	only in mock
P63024	Vesicle-associated membrane protein 3 OS=Mus musculus OX=10090 GN=Vamp3 PE=1 SV=1 - [VAMP3_MOUSE]	Vamp3	41.36446219	39.89915386	0	0	0	0	27.08787201	0	23.47022347	0	0.196705602	0.726530612	only in mock	only in mock
Q80SZ7	Guanine nucleotide-binding protein G(I)/G(S)/G(O) subunit gamma-5 OS=Mus musculus OX=10090 GN=Gng5 PE=1 SV=2 - [GBG5_MOUSE]	Gng5	78.40634837	61.25478375	0	0	0	0	46.5537107	0	41.21868542	0	0.196705602	0.726530612	only in mock	only in mock
Q8R1A4	Dedicator of cytokinesis protein 7 OS=Mus musculus OX=10090 GN=Dock7 PE=1 SV=3 - [DOCK7_MOUSE]	Dock7	89.03262594	0	267.0978778	0	0	0	118.7101679	0	135.9995826	0	0.196705602	0.726530612	only in mock	only in mock
Q91ZW2	GDP-fucose protein O-fucosyltransferase 1 OS=Mus musculus OX=10090 GN=Pofut1 PE=1 SV=1 - [OFUT1_MOUSE]	Pofut1	0	59.99745987	82.76558443	0	0	0	47.58768143	0	42.75555452	0	0.196705602	0.726530612	only in mock	only in mock
Q99KV1	DnaJ homolog subfamily B member 11 OS=Mus musculus OX=10090 GN=Dnajb11 PE=1 SV=1 - [DJB11_MOUSE]	Dnajb11	0	58.8691921	41.05898641	0	0	0	33.30939284	0	30.19002476	0	0.196705602	0.726530612	only in mock	#NUM!
Q9DAU1	Protein canopy homolog 3 OS=Mus musculus OX=10090 GN=Cnpy3 PE=1 SV=1 - [CNPY3_MOUSE]	Cnpy3	0	58.62584903	42.6946808	0	0	0	33.77350994	0	30.31399035	0	0.196705602	0.726530612	only in mock	#NUM!
Q9DBV4	Matrix remodeling-associated protein 8 OS=Mus musculus OX=10090 GN=Mxra8 PE=1 SV=1 - [MXRA8_MOUSE]	Mxra8	65.33051396	0	38.08975719	0	0	0	34.47342372	0	32.8150486	0	0.196705602	0.726530612	only in mock	#NUM!
Q9QXX0	Protein jagged-1 OS=Mus musculus OX=10090 GN=Jag1 PE=1 SV=1 - [JAG1_MOUSE]	Jag1	17.62250018	37.65139531	16.60353872	0	8.67854303	17.16522225	23.95914474	8.614588428	11.86877689	8.582789838	0.2	0.726530612	0.359553253	-1.475722633
Q8CG85	MAM domain-containing protein 2 OS=Mus musculus OX=10090 GN=Mamdc2 PE=1 SV=1 - [MAMC2_MOUSE]	Mamdc2	25.88097972	84.13925152	84.3523974	0	14.71892023	56.54807181	64.79087621	23.75566401	33.69712735	29.33714642	0.2	0.726530612	0.366651377	-1.447519135
Q05186	Reticulocalbin-1 OS=Mus musculus OX=10090 GN=Rcn1 PE=1 SV=1 - [RCN1_MOUSE]	Rcn1	140.0131812	472.6300015	258.1834708	94.00954527	76.34365131	154.9003538	290.2755512	108.4178501	168.6146895	41.21272025	0.2	0.726530612	0.373499765	-1.42082076
Q9CPT4	Myeloid-derived growth factor OS=Mus musculus OX=10090 GN=Mydgf PE=1 SV=1 - [MYDGF_MOUSE]	Mydgf	64.56841885	291.7890539	263.6284906	48.04814161	24.10257709	160.6141001	206.6619878	77.58827294	123.8595619	72.89247903	0.2	0.726530612	0.375435627	-1.413362534
P45878	Peptidyl-prolyl cis-trans isomerase FKBP2 OS=Mus musculus OX=10090 GN=Fkbp2 PE=1 SV=1 - [FKBP2_MOUSE]	Fkbp2	104.4798345	195.3215301	193.881342	72.36093519	0	119.4626093	164.5609022	63.94118151	52.03671353	60.17472847	0.2	0.726530612	0.388556338	-1.363804301
P27773	Protein disulfide-isomerase A3 OS=Mus musculus OX=10090 GN=Pdia3 PE=1 SV=2 - [PDIA3_MOUSE]	Pdia3	870.4191631	2538.115966	2038.364529	607.6480915	561.8955331	948.6245366	1815.63322	706.0560537	855.8680296	211.3123891	0.2	0.726530612	0.388875928	-1.362618161
Q62059	Versican core protein OS=Mus musculus OX=10090 GN=Vcan PE=1 SV=2 - [CSPG2_MOUSE]	Vcan	64.3218046	49.33312982	73.48660044	0	18.53594814	55.60784441	62.38051162	24.71459752	12.1931948	28.31412847	0.2	0.726530612	0.396191004	-1.335731973
Q91W90	Thioredoxin domain-containing protein 5 OS=Mus musculus OX=10090 GN=Txndc5 PE=1 SV=2 - [TXND5_MOUSE]	Txndc5	295.2128705	845.2858322	805.9460307	228.4026953	203.8466415	370.5969852	648.8149111	267.6154407	306.8594261	90.02582312	0.2	0.726530612	0.41246808	-1.277645618
Q9Z247	Peptidyl-prolyl cis-trans isomerase FKBP9 OS=Mus musculus OX=10090 GN=Fkbp9 PE=1 SV=1 - [FKBP9_MOUSE]	Fkbp9	52.77844677	117.3565494	127.3497698	38.68997183	23.49131855	60.97091557	99.16158868	41.05073532	40.47854992	18.85099338	0.2	0.726530612	0.413978193	-1.272373321
Q9CXI5	Mesencephalic astrocyte-derived neurotrophic factor OS=Mus musculus OX=10090 GN=Manf PE=1 SV=1 - [MANF_MOUSE]	Manf	332.2709593	804.6422318	798.5993428	181.7082765	216.0358835	437.4987137	645.1708446	278.4142912	270.9960937	138.836185	0.2	0.726530612	0.431535761	-1.212447977
Q9CQU0	Thioredoxin domain-containing protein 12 OS=Mus musculus OX=10090 GN=Txndc12 PE=1 SV=1 - [TXD12_MOUSE]	Txndc12	423.2227344	1053.978564	992.0292703	303.8399373	273.4309267	493.1915173	823.0768564	356.8207938	347.6663888	119.0752185	0.2	0.726530612	0.433520626	-1.20582746
P02469	Laminin subunit beta-1 OS=Mus musculus OX=10090 GN=Lamb1 PE=1 SV=3 - [LAMB1_MOUSE]	Lamb1	22.57620382	24.30831255	33.64879762	0	11.88016665	23.94232521	26.844438	11.94083062	5.956050084	11.97127788	0.2	0.726530612	0.444815817	-1.168720006
Q8BPG6	Inactive C-alpha-formylglycine-generating enzyme 2 OS=Mus musculus OX=10090 GN=Sumf2 PE=1 SV=2 - [SUMF2_MOUSE]	Sumf2	30.88232573	89.26579761	106.9535025	23.26325121	27.98529526	51.8419061	75.70054196	34.36348419	39.80851801	15.31978621	0.2	0.726530612	0.453939738	-1.139427307
Q921M3	Splicing factor 3B subunit 3 OS=Mus musculus OX=10090 GN=Sf3b3 PE=1 SV=1 - [SF3B3_MOUSE]	Sf3b3	100.914591	249.2866155	202.4516872	80.82155977	101.5347101	72.65587689	184.2176312	85.00404894	75.84803898	14.88679496	0.2	0.726530612	0.461432754	-1.11580768
P43276	Histone H1.5 OS=Mus musculus OX=10090 GN=H1-5 PE=1 SV=2 - [H15_MOUSE]	H1-5	172.8395923	403.8256975	426.8119224	93.68824686	118.3820538	252.1454188	334.4924041	154.7385732	140.4664206	85.25559341	0.2	0.726530612	0.462607136	-1.112140573
P56391	Cytochrome c oxidase subunit 6B1 OS=Mus musculus OX=10090 GN=Cox6b1 PE=1 SV=2 - [CX6B1_MOUSE]	Cox6b1	177.3632687	379.8308457	325.4077041	126.3775427	109.8125931	193.1474475	294.2006061	143.1125278	104.7792544	44.11597534	0.2	0.726530612	0.486445387	-1.039650251
Q9CYA0	Protein disulfide isomerase Creld2 OS=Mus musculus OX=10090 GN=Creld2 PE=1 SV=1 - [CREL2_MOUSE]	Creld2	29.0240727	56.31803981	64.97345052	21.84437376	18.39830399	33.3505382	50.10518767	24.53107198	18.76270477	7.829820398	0.2	0.726530612	0.48959146	-1.030349702
P06837	Neuromodulin OS=Mus musculus OX=10090 GN=Gap43 PE=1 SV=1 - [NEUM_MOUSE]	Gap43	165.5754798	255.994542	288.5232005	61.48352146	106.7679188	179.6916249	236.6977408	115.9810217	63.70486961	59.64016999	0.2	0.726530612	0.489996319	-1.029157183
Q64433	10 kDa heat shock protein, mitochondrial OS=Mus musculus OX=10090 GN=Hspe1 PE=1 SV=2 - [CH10_MOUSE]	Hspe1	1123.569886	2024.420607	1946.344148	716.8139878	584.3679758	1211.482754	1698.111547	837.5549057	499.0967576	330.5329229	0.2	0.726530612	0.49322726	-1.019675556
P24369	Peptidyl-prolyl cis-trans isomerase B OS=Mus musculus OX=10090 GN=Ppib PE=1 SV=2 - [PPIB_MOUSE]	Ppib	988.3377369	2584.674294	2577.75517	827.0319397	915.3428489	1310.836573	2050.255734	1017.73712	919.6544691	257.6434956	0.2	0.726530612	0.496395207	-1.010438908
P57759	Endoplasmic reticulum resident protein 29 OS=Mus musculus OX=10090 GN=Erp29 PE=1 SV=2 - [ERP29_MOUSE]	Erp29	301.0511667	744.1520078	758.2327442	250.0028154	227.6296574	437.0563251	601.1453062	304.8962659	259.9844923	114.9993499	0.2	0.726530612	0.507192292	-0.979395273
Q6P5E4	UDP-glucose:glycoprotein glucosyltransferase 1 OS=Mus musculus OX=10090 GN=Uggt1 PE=1 SV=4 - [UGGG1_MOUSE]	Uggt1	51.21383505	96.14815644	133.9489701	33.00448066	37.37514533	77.74297297	93.77032052	49.37419965	41.41879066	24.66507961	0.2	0.726530612	0.526544	-0.925374001
Q60847	Collagen alpha-1(XII) chain OS=Mus musculus OX=10090 GN=Col12a1 PE=2 SV=3 - [COCA1_MOUSE]	Col12a1	48.06143662	82.54663027	60.55725211	14.82150913	30.80483954	56.8693379	63.721773	34.16522886	17.45903175	21.22437604	0.2	0.726530612	0.536162559	-0.899257618
P47915	60S ribosomal protein L29 OS=Mus musculus OX=10090 GN=Rpl29 PE=1 SV=2 - [RL29_MOUSE]	Rpl29	163.4497337	445.9016845	238.9058111	112.1831467	131.2249151	225.1027498	282.7524098	156.1702705	146.2418178	60.45173497	0.2	0.726530612	0.552321625	-0.85641948
Q91XY4	Protocadherin gamma-A4 OS=Mus musculus OX=10090 GN=Pcdhga4 PE=1 SV=1 - [PCDG4_MOUSE]	Pcdhga4	98.84215601	82.93172547	91.54080974	0	71.18037398	84.16489877	91.10489707	51.78175758	7.964167561	45.31183383	0.2	0.726530612	0.568375129	-0.81508467
Q9CQ45	Neudesin OS=Mus musculus OX=10090 GN=Nenf PE=1 SV=1 - [NENF_MOUSE]	Nenf	60.51976962	98.25306788	153.161913	48.26652262	40.29208402	90.98975447	103.9782502	59.8494537	46.5856734	27.26145019	0.2	0.726530612	0.575595892	-0.796871798
P37889	Fibulin-2 OS=Mus musculus OX=10090 GN=Fbln2 PE=1 SV=2 - [FBLN2_MOUSE]	Fbln2	256.9437226	415.6826985	536.2827821	139.3479995	191.4156786	378.8872155	402.9697344	236.5502979	140.1027914	125.9865445	0.2	0.726530612	0.587017529	-0.76852451
P10493	Nidogen-1 OS=Mus musculus OX=10090 GN=Nid1 PE=1 SV=2 - [NID1_MOUSE]	Nid1	90.73357052	241.3727796	195.0523333	48.49169025	86.94622171	182.0546553	175.7195611	105.8308558	77.15801894	68.75491592	0.2	0.726530612	0.602271341	-0.731514484
Q9D1Q6	Endoplasmic reticulum resident protein 44 OS=Mus musculus OX=10090 GN=Erp44 PE=1 SV=1 - [ERP44_MOUSE]	Erp44	124.6790962	243.3044608	224.7922509	111.0264618	102.2697733	146.8870746	197.591936	120.0611032	63.81917527	23.64094866	0.2	0.726530612	0.607621473	-0.71875524
Q9CX56	26S proteasome non-ATPase regulatory subunit 8 OS=Mus musculus OX=10090 GN=Psmd8 PE=1 SV=2 - [PSMD8_MOUSE]	Psmd8	155.4541275	137.7511034	226.5635051	110.9987961	147.9156374	58.10903614	173.2562453	105.6744899	47.00635304	45.13942385	0.2	0.726530612	0.609931779	-0.71328021
Q9QXV0	ProSAAS OS=Mus musculus OX=10090 GN=Pcsk1n PE=1 SV=2 - [PCS1N_MOUSE]	Pcsk1n	69.55806725	68.13595963	84.76302009	34.88414671	68.15248791	41.32571936	74.15234899	48.12078466	9.216580353	17.64441316	0.2	0.726530612	0.648944845	-0.623832228
P30999	Catenin delta-1 OS=Mus musculus OX=10090 GN=Ctnnd1 PE=1 SV=2 - [CTND1_MOUSE]	Ctnnd1	14.97051825	15.13873198	9.369372637	7.354144759	7.643666329	10.98835751	13.15954096	8.6620562	3.283459438	2.019830198	0.2	0.726530612	0.658233918	-0.603327727
Q8BHN3	Neutral alpha-glucosidase AB OS=Mus musculus OX=10090 GN=Ganab PE=1 SV=1 - [GANAB_MOUSE]	Ganab	199.2684781	325.3690019	311.2144142	163.3485954	202.9603421	185.0945809	278.6172981	183.8011728	69.0815787	19.83752246	0.2	0.726530612	0.659690458	-0.600138858

Q9D8E6	60S ribosomal protein L4 OS=Mus musculus OX=10090 GN=Rpl4 PE=1 SV=3 - [RL4_MOUSE]	Rpl4	161.6435029	171.3822595	169.9537462	108.8447276	165.058512	73.7313802	167.6598362	115.8782066	5.259026715	46.0680325	0.2	0.726530612	0.691150661	-0.532927864
P07724	Albumin OS=Mus musculus OX=10090 GN=Alb PE=1 SV=3 - [ALBU_MOUSE]	Alb	313814.3322	550157.6209	601174.4741	295593.8587	278891.9922	441185.7566	488382.1424	338557.2025	153317.0574	89270.3936	0.2	0.726530612	0.693221912	-0.528610838
Q8VDN2	Sodium/potassium-transporting ATPase subunit alpha-1 OS=Mus musculus OX=10090 GN=Atp1a1 PE=1 SV=1 - [AT1A1_MOUSE]	Atp1a1	239.7868023	199.8140987	207.518306	81.64259671	202.9005284	165.1939863	215.7064023	149.9123705	21.20702597	62.05656563	0.2	0.726530612	0.694983407	-0.524949561
Q61576	Peptidyl-prolyl cis-trans isomerase FKBP10 OS=Mus musculus OX=10090 GN=Fkbp10 PE=1 SV=2 - [FKB10_MOUSE]	Fkbp10	68.49042775	102.4826025	100.303708	45.5199542	68.10293626	78.46877791	90.42557941	64.03055613	19.02761294	16.84768399	0.2	0.726530612	0.708102249	-0.497970397
Q6PIE5	Sodium/potassium-transporting ATPase subunit alpha-2 OS=Mus musculus OX=10090 GN=Atp1a2 PE=1 SV=1 - [AT1A2_MOUSE]	Atp1a2	229.3299697	199.8140987	217.0009068	81.64259671	202.9005284	176.1434826	215.3816584	153.5622026	14.8244101	63.70484406	0.2	0.726530612	0.712977157	-0.488072239
P08905	Lysozyme C-2 OS=Mus musculus OX=10090 GN=Lyz2 PE=1 SV=2 - [LYZ2_MOUSE]	Lyz2	55.79255928	68.0361841	86.27704974	43.62628395	66.73340883	47.16059921	70.03526437	52.506764	15.34025036	12.446723	0.2	0.726530612	0.749718938	-0.41557825
Q02819	Nucleobindin-1 OS=Mus musculus OX=10090 GN=Nucb1 PE=1 SV=2 - [NUCB1_MOUSE]	Nucb1	468.7033883	612.9273625	642.3009936	392.8041582	423.1712817	478.4997712	574.6439148	431.491737	92.91527816	43.44947651	0.2	0.726530612	0.750885419	-0.413335317
P28653	Biglycan OS=Mus musculus OX=10090 GN=Bgn PE=1 SV=1 - [PGS1_MOUSE]	Bgn	62.90861951	69.69510131	33.16412209	60.75750504	32.11287872	32.68730275	55.25594764	41.85256217	19.43066265	16.37467983	0.2	0.726530612	0.7574309	-0.400813816
P35979	60S ribosomal protein L12 OS=Mus musculus OX=10090 GN=Rpl12 PE=1 SV=2 - [RL12_MOUSE]	Rpl12	265.865423	299.5807382	202.0282516	172.9860538	238.5564089	177.1805981	255.8248043	196.2410203	49.54525637	36.70616617	0.2	0.726530612	0.767091451	-0.382529512
P53026	60S ribosomal protein L10a OS=Mus musculus OX=10090 GN=Rpl10a PE=1 SV=3 - [RL10A_MOUSE]	Rpl10a	142.6916626	209.4924512	161.8107672	125.2409283	157.5044004	117.2159331	171.331627	133.3204206	34.40307276	21.32483985	0.2	0.726530612	0.7781425	-0.361893717
Q60605	Myosin light polypeptide 6 OS=Mus musculus OX=10090 GN=Myl6 PE=1 SV=3 - [MYL6_MOUSE]	Myl6	501.0216725	563.9552817	439.4787139	480.5859372	413.874858	311.8196428	501.4852227	402.0934793	62.23957857	84.99774225	0.2	0.726530612	0.80180524	-0.318676249
P29341	Polyadenylate-binding protein 1 OS=Mus musculus OX=10090 GN=Pabpc1 PE=1 SV=2 - [PABP1_MOUSE]	Pabpc1	681.8669611	830.14731	788.2296224	599.4388427	718.6665862	539.225589	766.7479645	619.1103393	76.43861387	91.32356595	0.2	0.726530612	0.807449603	-0.308555879
Q9CR21	Acyl carrier protein, mitochondrial OS=Mus musculus OX=10090 GN=Ndufab1 PE=1 SV=1 - [ACPM_MOUSE]	Ndufab1	105.8110375	138.3209051	118.1970738	109.0926264	103.1323905	94.64710209	120.7763388	102.2907063	16.40769117	7.259450219	0.2	0.726530612	0.846943262	-0.23966277
Q9ER00	Syntaxin-12 OS=Mus musculus OX=10090 GN=Stx12 PE=1 SV=1 - [STX12_MOUSE]	Stx12	88.44616867	81.21720934	106.3623167	106.7883343	110.8288179	91.70523822	92.00856491	103.1074635	12.94554389	10.07915834	0.2	0.726530612	1.120628972	0.164308696
Q00493	Carboxypeptidase E OS=Mus musculus OX=10090 GN=Cpe PE=1 SV=2 - [CBPE_MOUSE]	Cpe	3638.282086	3823.58617	4584.282974	4431.834313	4930.365552	4629.912716	4015.383743	4664.037527	501.3174162	251.011406	0.2	0.726530612	1.161542165	0.216041526
P97798	Neogenin OS=Mus musculus OX=10090 GN=Neo1 PE=1 SV=1 - [NEO1_MOUSE]	Neo1	78.07339861	59.43774151	76.71285357	79.74617395	95.19207906	77.212382	71.40799789	84.05021167	10.38884253	9.731954178	0.2	0.726530612	1.177041986	0.235165784
Q9JME2	Carbohydrate sulfotransferase 11 OS=Mus musculus OX=10090 GN=Chst11 PE=2 SV=1 - [CHSTB_MOUSE]	Chst11	65.53469666	90.90839819	77.84974107	84.51858383	115.1821491	101.9682157	78.09761197	100.5563162	12.68866669	15.38046338	0.2	0.726530612	1.287572228	0.364653364
Q810U4	Neuronal cell adhesion molecule OS=Mus musculus OX=10090 GN=Nrcam PE=1 SV=2 - [NRCAM_MOUSE]	Nrcam	279.6540754	282.9038602	322.59119	322.2255201	464.198987	354.7541119	295.0497085	380.392873	23.90690648	74.37826211	0.2	0.726530612	1.289250123	0.366532183
Q9R1Q9	V-type proton ATPase subunit S1 OS=Mus musculus OX=10090 GN=Atp6ap1 PE=1 SV=1 - [VAS1_MOUSE]	Atp6ap1	291.7635529	299.7524971	379.9831866	335.9203639	527.2435099	413.2865202	323.8330789	425.4834647	48.79120571	96.24297706	0.2	0.726530612	1.313897475	0.393852705
P15116	Cadherin-2 OS=Mus musculus OX=10090 GN=Cdh2 PE=1 SV=2 - [CADH2_MOUSE]	Cdh2	803.5164595	632.5559702	980.5683012	938.0373781	1219.242971	1046.028327	805.5469103	1067.769559	174.0150501	141.8578767	0.2	0.726530612	1.325521264	0.406559814
Q9Z0L8	Gamma-glutamyl hydrolase OS=Mus musculus OX=10090 GN=Ggh PE=1 SV=2 - [GGH_MOUSE]	Ggh	248.3736485	304.9989891	349.2738141	322.0200035	440.4080185	442.7274118	300.8821506	401.7184779	50.5759046	69.03064548	0.2	0.726530612	1.335135624	0.4169863
Q9QZE7	Translin-associated protein X OS=Mus musculus OX=10090 GN=Tsnax PE=1 SV=1 - [TSNAX_MOUSE]	Tsnax	91.87341615	82.09814274	90.08499714	130.0013143	136.5414103	90.36487184	88.01885201	118.9691988	5.204873617	24.98697369	0.2	0.726530612	1.351633157	0.434703647
P47867	Secretogranin-3 OS=Mus musculus OX=10090 GN=Scg3 PE=1 SV=1 - [SCG3_MOUSE]	Scg3	262.1446568	300.1662091	366.5504501	353.7577954	466.9634632	441.8867991	309.6204387	420.8693526	52.84107553	59.45738471	0.2	0.726530612	1.359307397	0.442871748
P31786	Acyl-CoA-binding protein OS=Mus musculus OX=10090 GN=Dbi PE=1 SV=2 - [ACBP_MOUSE]	Dbi	4721.539915	2586.871908	2902.587368	5290.623967	4956.467558	3686.152609	3403.666397	4644.414711	1152.177079	846.5310503	0.2	0.726530612	1.36453288	0.448407158
Q8C1Q4	Meteorin OS=Mus musculus OX=10090 GN=Metrn PE=1 SV=2 - [METRN_MOUSE]	Metrn	232.9680269	230.5446657	281.3879537	241.5489057	348.7884649	427.7230849	248.3002154	339.3534852	28.68042864	93.44501261	0.2	0.726530612	1.366706366	0.450703317
O88851	Putative hydrolase RBBP9 OS=Mus musculus OX=10090 GN=Rbbp9 PE=1 SV=2 - [RBBP9_MOUSE]	Rbbp9	108.9112909	105.3777744	130.7704072	183.5356356	129.9946865	158.5113651	115.0198242	157.3472291	13.75434781	26.78945159	0.2	0.726530612	1.368000953	0.452069235
O55029	Coatomer subunit beta' OS=Mus musculus OX=10090 GN=Copb2 PE=1 SV=2 - [COPB2_MOUSE]	Copb2	75.62351295	29.81198951	46.9823936	80.30627999	77.46553088	51.90109016	50.80596536	69.89096701	23.14386951	15.64430298	0.2	0.726530612	1.375644898	0.460108108
Q80X90	Filamin-B OS=Mus musculus OX=10090 GN=Flnb PE=1 SV=3 - [FLNB_MOUSE]	Flnb	506.5830985	226.7296502	219.8517727	513.0612232	516.9318061	281.3127781	317.7215071	437.1019358	163.595085	134.9312477	0.2	0.726530612	1.375739212	0.460207015
Q8VHX6	Filamin-C OS=Mus musculus OX=10090 GN=Flnc PE=1 SV=3 - [FLNC_MOUSE]	Flnc	506.5830985	226.7296502	219.8517727	513.0612232	516.9318061	281.3127781	317.7215071	437.1019358	163.595085	134.9312477	0.2	0.726530612	1.375739212	0.460207015
P40124	Adenylyl cyclase-associated protein 1 OS=Mus musculus OX=10090 GN=Cap1 PE=1 SV=4 - [CAP1_MOUSE]	Cap1	472.349188	194.0921436	192.4009167	492.6035346	497.9979459	198.3323192	286.2807494	396.3112665	161.1422134	171.4760118	0.2	0.726530612	1.384344799	0.46920332
P57780	Alpha-actinin-4 OS=Mus musculus OX=10090 GN=Actn4 PE=1 SV=1 - [ACTN4_MOUSE]	Actn4	584.3629037	382.7931893	325.7754586	677.151753	685.7707804	435.0411278	430.9771839	599.3212204	135.8607364	142.3359881	0.2	0.726530612	1.39061009	0.475717962
Q7TPR4	Alpha-actinin-1 OS=Mus musculus OX=10090 GN=Actn1 PE=1 SV=1 - [ACTN1_MOUSE]	Actn1	584.3629037	382.7931893	325.7754586	677.151753	685.7707804	435.0411278	430.9771839	599.3212204	135.8607364	142.3359881	0.2	0.726530612	1.39061009	0.475717962
Q9Z204	Heterogeneous nuclear ribonucleoproteins C1/C2 OS=Mus musculus OX=10090 GN=Hnrnpc PE=1 SV=1 - [HNRPC_MOUSE]	Hnrnpc	709.8323106	402.6588955	406.7863272	755.8995234	843.5440066	513.6865693	506.4258444	704.3766998	176.1672551	170.8579329	0.2	0.726530612	1.390878265	0.475996155
P49312	Heterogeneous nuclear ribonucleoprotein A1 OS=Mus musculus OX=10090 GN=Hnrnpa1 PE=1 SV=2 - [ROA1_MOUSE]	Hnrnpa1	2499.245962	1132.679104	1134.034219	2525.992896	2501.328195	1609.299	1588.653095	2212.206697	788.5968469	522.2790016	0.2	0.726530612	1.392504571	0.477682064
Q9D3A9	Protein tweety homolog 1 OS=Mus musculus OX=10090 GN=Ttyh1 PE=1 SV=1 - [TTYH1_MOUSE]	Ttyh1	67.00336013	30.43986377	48.48697451	48.83179172	81.51016217	72.95479605	48.64339947	67.76558331	18.28225009	16.94594112	0.2	0.726530612	1.393109529	0.47830869
P61089	Ubiquitin-conjugating enzyme E2 N OS=Mus musculus OX=10090 GN=Ube2n PE=1 SV=1 - [UBE2N_MOUSE]	Ube2n	1466.766195	633.5164563	700.6621986	1500.942625	1491.196408	919.0597701	933.6482834	1303.732934	462.9127008	333.1723723	0.2	0.726530612	1.39638551	0.481697292
Q01730	Ras suppressor protein 1 OS=Mus musculus OX=10090 GN=Rsu1 PE=1 SV=3 - [RSU1_MOUSE]	Rsu1	216.0821727	97.9873424	112.0334967	249.7993415	234.7926606	117.9284333	142.0343373	200.8401451	64.510736	72.19462645	0.2	0.726530612	1.414025291	0.499807924
Q06138	Calcium-binding protein 39 OS=Mus musculus OX=10090 GN=Cab39 PE=1 SV=2 - [CAB39_MOUSE]	Cab39	157.0798196	90.61348828	89.48837209	177.9429567	179.8545607	119.5009663	112.3938933	159.0994946	38.70323599	34.30664863	0.2	0.726530612	1.415552837	0.5013656
Q9CX34	Protein SGT1 homolog OS=Mus musculus OX=10090 GN=Sugt1 PE=1 SV=3 - [SGT1_MOUSE]	Sugt1	155.1009753	68.27085064	74.45727532	156.1948876	171.9276095	96.30328908	99.27636709	141.4752621	48.44438176	39.9031326	0.2	0.726530612	1.425064859	0.511027582
P23506	Protein-L-isoaspartate(D-aspartate) O-methyltransferase OS=Mus musculus OX=10090 GN=Pcmt1 PE=1 SV=3 - [PIMT_MOUSE]	Pcmt1	137.456616	62.98135102	57.18583886	144.0280915	137.9738867	86.11273911	85.87460197	122.7049058	44.76522223	31.83399667	0.2	0.726530612	1.42888471	0.514889517
P62889	60S ribosomal protein L30 OS=Mus musculus OX=10090 GN=Rpl30 PE=1 SV=2 - [RL30_MOUSE]	Rpl30	136.5640803	55.73522713	57.27227131	138.1514334	141.5138473	77.703911	83.19052623	119.1230639	46.22924213	35.90941555	0.2	0.726530612	1.431930645	0.517961618
P26369	Splicing factor U2AF 65 kDa subunit OS=Mus musculus OX=10090 GN=U2af2 PE=1 SV=3 - [U2AF2_MOUSE]	U2af2	372.2214613	186.304985	197.607629	411.6501469	419.9705703	252.6620915	252.0446918	361.4276029	104.2294555	94.2855223	0.2	0.726530612	1.433982205	0.520027121
P54728	UV excision repair protein RAD23 homolog B OS=Mus musculus OX=10090 GN=Rad23b PE=1 SV=2 - [RD23B_MOUSE]	Rad23b	319.1139373	145.3895933	157.6192834	336.6514397	361.3078479	194.8478574	207.3742713	297.6023817	96.96239502	89.83793078	0.2	0.726530612	1.435097902	0.52114916
Q61171	Peroxiredoxin-2 OS=Mus musculus OX=10090 GN=Prdx2 PE=1 SV=3 - [PRDX2_MOUSE]	Prdx2	2789.108989	1459.947711	1502.907631	3198.87535	3181.860314	1933.290164	1917.321444	2771.341943	755.2956583	725.8239908	0.2	0.726530612	1.445423746	0.531492501
P08228	Superoxide dismutase [Cu-Zn] OS=Mus musculus OX=10090 GN=Sod1 PE=1 SV=2 - [SODC_MOUSE]	Sod1	1511.673828	848.445476	909.210966	1866.34805	1821.196992	1050.281941	1089.776757	1579.275661	366.634648	458.6779057	0.2	0.726530612	1.449173559	0.535230389
P62962	Profilin-1 OS=Mus musculus OX=10090 GN=Pfn1 PE=1 SV=2 - [PROF1_MOUSE]	Pfn1	3860.922313	1527.047962	1526.79908	3904.930782	3875.888867	2281.807869	2304.923118	3354.209173	1347.534836	928.8402852	0.2	0.726530612	1.4552369	0.54125403
Q9JHW2	Omega-amidase NIT2 OS=Mus musculus OX=10090 GN=Nit2 PE=1 SV=1 - [NIT2_MOUSE]	Nit2	171.4098603	65.04327379	85.62362951	205.9936161	174.557613	89.48687395	107.3589212	156.6793677	56.4161312	60.2758574	0.2	0.726530612	1.459397747	0.545373132
Q91V76	Ester hydrolase C11orf54 homolog OS=Mus musculus OX=10090 PE=1 SV=1 - [CK054_MOUSE]	Not availa	161.1402982	62.43128384	77.29819496	176.7843155	168.9454088	93.38930452	100.2899257	146.3730096	53.21965933	46.0523275	0.2	0.726530612	1.459498635	0.545472861
Q60692	Proteasome subunit beta type-6 OS=Mus musculus OX=10090 GN=Psmb6 PE=1 SV=3 - [PSB6_MOUSE]	Psmb6	480.8050202	271.9185103	190.6186701	573.8289132	502.2540701	302.0677808	314.4474002	459.383588	149.6948874	140.8614104	0.2	0.726530612	1.460923473	0.546880608
O70591	Prefoldin subunit 2 OS=Mus musculus OX=10090 GN=Pfdn2 PE=1 SV=2 - [PFD2_MOUSE]	Pfdn2	145.7034149	87.25257459	55.40871774	148.7428887	157.7060545	114.8671456	96.12156908	140.4386963	45.79604111	22.59452891	0.2	0.726530612	1.46105289	0.547008404
P06745	Glucose-6-phosphate isomerase OS=Mus musculus OX=10090 GN=Gpi PE=1 SV=4 - [G6PI_MOUSE]	Gpi	2234.255481	880.5746383	899.4868578	2491.05355	2249.763547	1134.980332	1338.105659	1958.599143	776.1461177	723.405955	0.2	0.726530612	1.46371038	0.54963012
Q9Z2U0	Proteasome subunit alpha type-7 OS=Mus musculus OX=10090 GN=Psma7 PE=1 SV=1 - [PSA7_MOUSE]	Psma7	575.0880828	298.6497061	227.0192566	664.770259	593.2776401	362.355747	366.9190152	540.1345487	183.8028859	158.0562413	0.2	0.726530612	1.472081103	0.557857158
P17742	Peptidyl-prolyl cis-trans isomerase A OS=Mus musculus OX=10090 GN=Ppia PE=1 SV=2 - [PPIA_MOUSE]	Ppia	12867.96089	5671.971039	6645.55472	14559.65621	13695.14217	8870.799396	8395.162216	12375.19926	3904.025136	3065.527676	0.2	0.726530612	1.474086973	0.559821648
Q9Z2U1	Proteasome subunit alpha type-5 OS=Mus musculus OX=10090 GN=Psma5 PE=1 SV=1 - [PSA5_MOUSE]	Psma5	567.054228	277.3650888	232.1353788	622.7724017	616.0469796	348.9105148	358.8515652	529.2432987	181.7214716	156.2089707	0.2	0.726530612	1.474825109	0.560543884
Q76KF0	Semaphorin-6D OS=Mus musculus OX=10090 GN=Sema6d PE=1 SV=1 - [SEM6D_MOUSE]	Sema6d	11.75160528	9.971536943	18.16300555	13.42918603	22.21602099	23.21185784	13.29538259	19.61902162	4.308419176	5.383629929	0.2	0.726530612	1.475626706	0.561327805
Q60972	Histone-binding protein RBBP4 OS=Mus musculus OX=10090 GN=Rbbp4 PE=1 SV=5 - [RBBP4_MOUSE]	Rbbp4	488.813748	200.8639777	185.7084659	497.5146494	516.3052198	292.3092864	291.7953972	435.3763852	170.7910867	124.2554524	0.2	0.726530612	1.492060496	0.577306031
Q9Z2W0	Aspartyl aminopeptidase OS=Mus musculus OX=10090 GN=Dnpep PE=1 SV=2 - [DNPEP_MOUSE]	Dnpep	275.9190822	118.4428872	115.169765	290.3568701	311.7515166	159.5898424	169.8439115	253.8994097	91.87836914	82.37204374	0.2	0.726530612	1.494898507	0.580047539
O88958	Glucosamine-6-phosphate isomerase 1 OS=Mus musculus OX=10090 GN=Gnpda1 PE=1 SV=1 - [GNPI1_MOUSE]	Gnpda1	158.2720929	75.48369552	87.00206567	179.2108879	194.0354615	106.382154	106.9192847	159.8761678	44.84419021	46.91640578	0.2	0.726530612	1.495297769	0.580432806
Q61024	Asparagine synthetase [glutamine-hydrolyzing] OS=Mus musculus OX=10090 GN=Asns PE=1 SV=3 - [ASNS_MOUSE]	Asns	218.2342963	82.85309945	84.2176737	229.660468	220.3693492	126.450224	128.4350231	192.1600137	77.77144469	57.09565282	0.2	0.726530612	1.496165213	0.581269492
O08807	Peroxiredoxin-4 OS=Mus musculus OX=10090 GN=Prdx4 PE=1 SV=1 - [PRDX4_MOUSE]	Prdx4	2915.556567	1421.020596	1574.388646	3320.990718	3559.636782	1968.193994	1970.321936	2949.607165	822.1811354	858.263855	0.2	0.726530612	1.497017878	0.582091451
P51880	Fatty acid-binding protein, brain OS=Mus musculus OX=10090 GN=Fabp7 PE=1 SV=2 - [FABP7_MOUSE]	Fabp7	8863.371231	3996.191639	3985.447327	9308.349886	10232.43537	5711.386114	5615.003399	8417.390457	2813.174193	2388.58287	0.2	0.726530612	1.499089112	0.584086145
P23198	Chromobox protein homolog 3 OS=Mus musculus OX=10090 GN=Cbx3 PE=1 SV=2 - [CBX3_MOUSE]	Cbx3	84.28958051	56.13866181	49.91800099	130.9289933	94.77382338	59.94508506	63.44874777	95.21596725	18.31473143	35.49401959	0.2	0.726530612	1.500675279	0.585611836
P59017	Bcl-2-like protein 13 OS=Mus musculus OX=10090 GN=Bcl2l13 PE=1 SV=2 - [B2L13_MOUSE]	Bcl2l13	79.02174162	32.19311946	38.63669042	94.29435216	83.91395006	47.78729994	49.95051717	75.33186739	25.38172512	24.41240635	0.2	0.726530612	1.508129878	0.592760677
P37804	Transgelin OS=Mus musculus OX=10090 GN=Tagln PE=1 SV=3 - [TAGL_MOUSE]	Tagln	511.549969	381.3117222	409.0988811	597.2950366	868.8900736	500.6442315	433.9868575	655.6097806	68.59343087	190.9233016	0.2	0.726530612	1.510667361	0.595186023
O55023	Inositol monophosphatase 1 OS=Mus musculus OX=10090 GN=Impa1 PE=1 SV=1 - [IMPA1_MOUSE]	Impa1	277.1151143	113.4674096	116.856121	308.1448459	323.1091988	136.9809469	169.146215	256.0783306	93.51915989	103.4123931	0.2	0.726530612	1.513946562	0.598314283
Q9EQ80	NIF3-like protein 1 OS=Mus musculus OX=10090 GN=Nif3l1 PE=1 SV=4 - [NIF3L_MOUSE]	Nif3l1	103.7972353	44.45535082	58.46221288	115.8996723	110.5687732	87.61503054	68.904933	104.694492	31.018585	15.02949185	0.2	0.726530612	1.519404889	0.603506368
Q8C1A5	Thimet oligopeptidase OS=Mus musculus OX=10090 GN=Thop1 PE=1 SV=1 - [THOP1_MOUSE]	Thop1	266.8800884	115.5295269	119.8957284	296.4506992	292.7944246	175.7236915	167.4351146	254.9896051	86.14953885	68.67063332	0.2	0.726530612	1.522915941	0.606836313
Q8VDD5	Myosin-9 OS=Mus musculus OX=10090 GN=Myh9 PE=1 SV=4 - [MYH9_MOUSE]	Myh9	145.9362354	105.8378091	80.66824021	214.4520091	157.2055558	135.8062528	110.8140949	169.1546059	32.91732622	40.6616969	0.2	0.726530612	1.526471935	0.610201065
Q9CQ60	6-phosphogluconolactonase OS=Mus musculus OX=10090 GN=Pgls PE=1 SV=1 - [6PGL_MOUSE]	Pgls	798.4313952	256.0611778	289.2003492	816.3319664	849.1706353	389.4978028	447.8976407	685.0001348	304.0230023	256.4387173	0.2	0.726530612	1.529367589	0.612935205
Q8BTM8	Filamin-A OS=Mus musculus OX=10090 GN=Flna PE=1 SV=5 - [FLNA_MOUSE]	Flna	660.3414186	342.8475228	366.6216147	842.272692	787.759137	466.5223146	456.6035187	698.8513812	176.8421634	203.0407061	0.2	0.726530612	1.530543136	0.614043705
Q61598	Rab GDP dissociation inhibitor beta OS=Mus musculus OX=10090 GN=Gdi2 PE=1 SV=1 - [GDIB_MOUSE]	Gdi2	1296.356332	660.4188012	726.7796747	1404.21883	1841.666679	898.5506277	894.5182695	1381.478712	349.5801955	471.9690737	0.2	0.726530612	1.544382892	0.627030478
P05064	Fructose-bisphosphate aldolase A OS=Mus musculus OX=10090 GN=Aldoa PE=1 SV=2 - [ALDOA_MOUSE]	Aldoa	4652.210362	1788.881258	1925.89468	5105.942352	5074.73055	2758.083061	2788.995433	4312.918655	1615.045068	1346.617554	0.2	0.726530612	1.546405779	0.628918934
P62869	Elongin-B OS=Mus musculus OX=10090 GN=Elob PE=1 SV=1 - [ELOB_MOUSE]	Elob	211.3738175	87.05888643	127.172496	240.8782944	291.3364	127.4057064	141.8684	219.8734669	63.44704656	83.95963411	0.2	0.726530612	1.549841028	0.632120242
P35700	Peroxiredoxin-1 OS=Mus musculus OX=10090 GN=Prdx1 PE=1 SV=1 - [PRDX1_MOUSE]	Prdx1	6121.080464	2801.217482	3091.98042	7029.636294	7584.344764	4009.487913	4004.759455	6207.822991	1838.544732	1923.910914	0.2	0.726530612	1.550111326	0.632371831
P24527	Leukotriene A-4 hydrolase OS=Mus musculus OX=10090 GN=Lta4h PE=1 SV=4 - [LKHA4_MOUSE]	Lta4h	338.2242238	131.3122499	148.7341753	392.7760029	366.1666186	203.5701685	206.0902163	320.8375967	114.7624844	102.4243726	0.2	0.726530612	1.556782279	0.638567193
Q9WVA4	Transgelin-2 OS=Mus musculus OX=10090 GN=Tagln2 PE=1 SV=4 - [TAGL2_MOUSE]	Tagln2	1472.026046	545.9647547	679.4442042	1650.952752	1625.585111	927.5712008	899.1450015	1401.369688	500.5983509	410.5175197	0.2	0.726530612	1.558558058	0.640211898
Q9QYB1	Chloride intracellular channel protein 4 OS=Mus musculus OX=10090 GN=Clic4 PE=1 SV=3 - [CLIC4_MOUSE]	Clic4	587.0204402	187.5288287	213.4823326	604.7759478	587.8460297	354.8951159	329.3438672	515.8390311	223.5314481	139.6383307	0.2	0.726530612	1.56626275	0.647326254
Q99PT1	Rho GDP-dissociation inhibitor 1 OS=Mus musculus OX=10090 GN=Arhgdia PE=1 SV=3 - [GDIR1_MOUSE]	Arhgdia	1227.299767	766.4521131	934.7114319	1572.574238	1846.96132	1167.35441	976.1544374	1528.963322	233.2022343	341.8959225	0.2	0.726530612	1.566312936	0.647372479
Q99MN1	Lysine--tRNA ligase OS=Mus musculus OX=10090 GN=Kars1 PE=1 SV=1 - [SYK_MOUSE]	Kars1	286.736512	124.9910366	141.5619982	356.7729047	306.8154876	208.8124082	184.4298489	290.8002668	88.98673592	75.26913446	0.2	0.726530612	1.576752725	0.656956426
P17710	Hexokinase-1 OS=Mus musculus OX=10090 GN=Hk1 PE=1 SV=3 - [HXK1_MOUSE]	Hk1	53.8449255	24.05878279	35.50620243	59.49578736	76.63080527	43.11307608	37.80330357	59.74655624	15.0253479	16.76027166	0.2	0.726530612	1.5804586	0.660343244
P62245	40S ribosomal protein S15a OS=Mus musculus OX=10090 GN=Rps15a PE=1 SV=2 - [RS15A_MOUSE]	Rps15a	109.8628882	32.55111225	38.20885168	113.8851443	126.4386107	45.21097917	60.20761738	95.17824472	43.09567199	43.72577157	0.2	0.726530612	1.580833935	0.660685822
Q8R464	Cell adhesion molecule 4 OS=Mus musculus OX=10090 GN=Cadm4 PE=1 SV=1 - [CADM4_MOUSE]	Cadm4	307.5029139	164.8382454	162.2717175	329.5347987	412.1899186	263.200345	211.5376256	334.9750208	83.11828432	74.64362182	0.2	0.726530612	1.583524537	0.663139223
P13020	Gelsolin OS=Mus musculus OX=10090 GN=Gsn PE=1 SV=3 - [GELS_MOUSE]	Gsn	114.6219765	92.29417756	85.36451343	153.2429344	200.1770343	110.6132159	97.42688915	154.6777282	15.28915733	44.7991447	0.2	0.726530612	1.587628729	0.666873574
Q9QYC0	Alpha-adducin OS=Mus musculus OX=10090 GN=Add1 PE=1 SV=2 - [ADDA_MOUSE]	Add1	46.68057289	24.46453299	16.66937148	51.62993881	54.81468736	33.40401526	29.27149245	46.61621381	15.57235361	11.552372	0.2	0.726530612	1.592546532	0.671335527
P83940	Elongin-C OS=Mus musculus OX=10090 GN=Eloc PE=1 SV=1 - [ELOC_MOUSE]	Eloc	85.65126959	38.39231205	30.46192341	100.2656442	96.76437801	50.05172696	51.50183502	82.36058304	29.83891213	28.03500227	0.2	0.726530612	1.599177641	0.677330207
P68037	Ubiquitin-conjugating enzyme E2 L3 OS=Mus musculus OX=10090 GN=Ube2l3 PE=1 SV=1 - [UB2L3_MOUSE]	Ube2l3	766.2187788	304.276096	336.8944642	915.9035373	854.0852671	481.115172	469.1297796	750.3679921	257.803015	235.2194411	0.2	0.726530612	1.599489149	0.677611205
P18760	Cofilin-1 OS=Mus musculus OX=10090 GN=Cfl1 PE=1 SV=3 - [COF1_MOUSE]	Cfl1	6645.453516	1929.352027	2516.279782	7306.948027	6909.182925	3591.544621	3697.028442	5935.891858	2570.219614	2039.982167	0.2	0.726530612	1.60558458	0.683098666
Q03173	Protein enabled homolog OS=Mus musculus OX=10090 GN=Enah PE=1 SV=2 - [ENAH_MOUSE]	Enah	50.99978762	25.70690486	16.27084521	60.85814383	54.88495015	34.24451153	30.99251256	49.9958685	17.95767515	13.96419334	0.2	0.726530612	1.613159578	0.689889161
Q8K4Z3	NAD(P)H-hydrate epimerase OS=Mus musculus OX=10090 GN=Naxe PE=1 SV=1 - [NNRE_MOUSE]	Naxe	216.6139817	108.3150908	70.93062793	245.2207129	250.815426	142.6947931	131.9532335	212.9103107	75.66360572	60.87273102	0.2	0.726530612	1.613528559	0.690219114
Q9CQE8	RNA transcription, translation and transport factor protein OS=Mus musculus OX=10090 GN=RTRAF PE=1 SV=1 - [RTRAF_MOUSE]	RTRAF	126.0156906	45.09275227	46.55859808	150.5294955	136.0066423	65.25857702	72.55568032	117.264905	46.30352796	45.62041127	0.2	0.726530612	1.616205712	0.692610837
Q6P1F6	Serine/threonine-protein phosphatase 2A 55 kDa regulatory subunit B alpha isoform OS=Mus musculus OX=10090 GN=Ppp2r2a PE=1 SV=1 - [2ABA_MOUS	Ppp2r2a	86.12363704	35.61269547	30.11353215	97.77599082	94.03521636	54.53854413	50.61662156	82.11658377	30.87266284	23.9564094	0.2	0.726530612	1.622324471	0.698062393
P70296	Phosphatidylethanolamine-binding protein 1 OS=Mus musculus OX=10090 GN=Pebp1 PE=1 SV=3 - [PEBP1_MOUSE]	Pebp1	4495.041871	2042.896129	2294.375239	5500.711572	5533.957794	3326.611942	2944.104413	4787.093769	1349.023975	1264.923596	0.2	0.726530612	1.625993204	0.701321228
Q91ZJ5	UTP--glucose-1-phosphate uridylyltransferase OS=Mus musculus OX=10090 GN=Ugp2 PE=1 SV=3 - [UGPA_MOUSE]	Ugp2	172.7461914	92.19843722	70.05221468	205.7164584	243.4621676	96.08149294	111.6656144	181.753373	54.04388451	76.55676268	0.2	0.726530612	1.627657484	0.702797138
P22892	AP-1 complex subunit gamma-1 OS=Mus musculus OX=10090 GN=Ap1g1 PE=1 SV=3 - [AP1G1_MOUSE]	Ap1g1	59.3278388	35.70196741	19.08063783	84.55331869	64.84400845	36.63021966	38.03681468	62.00918227	20.22493338	24.08698891	0.2	0.726530612	1.630241196	0.705085429
P21460	Cystatin-C OS=Mus musculus OX=10090 GN=Cst3 PE=1 SV=2 - [CYTC_MOUSE]	Cst3	18514.82911	8689.552281	11893.64438	26350.13208	22958.92923	14719.10539	13032.67526	21342.72223	5010.694535	5981.579157	0.2	0.726530612	1.637631707	0.711610941

Q9D0I9	Arginine--tRNA ligase, cytoplasmic OS=Mus musculus OX=10090 GN=Rars1 PE=1 SV=2 - [SYRC_MOUSE]	Rars1	201.3319244	63.01045564	88.55137123	222.4962228	233.7675074	122.0096129	117.6312504	192.7577811	73.60323847	61.52835067	0.2	0.726530612	1.638661328	0.712517715
Q62446	Peptidyl-prolyl cis-trans isomerase FKBP3 OS=Mus musculus OX=10090 GN=Fkbp3 PE=1 SV=2 - [FKBP3_MOUSE]	Fkbp3	469.4161775	120.6553372	114.4676293	484.7572073	481.3385614	191.547086	234.8463813	385.8809515	203.1669606	168.3067446	0.2	0.726530612	1.643120704	0.716438465
Q9DBG3	AP-2 complex subunit beta OS=Mus musculus OX=10090 GN=Ap2b1 PE=1 SV=1 - [AP2B1_MOUSE]	Ap2b1	354.9036383	118.9751814	144.667224	382.1324251	414.3635755	223.9605856	206.1820146	340.1521954	129.4357419	101.9072098	0.2	0.726530612	1.649766572	0.72226191
Q7TQI3	Ubiquitin thioesterase OTUB1 OS=Mus musculus OX=10090 GN=Otub1 PE=1 SV=2 - [OTUB1_MOUSE]	Otub1	502.5279978	138.9433092	176.8262483	537.391387	567.9722139	245.7051115	272.7658518	450.3562375	199.8793688	177.8914255	0.2	0.726530612	1.651072649	0.723403602
Q61937	Nucleophosmin OS=Mus musculus OX=10090 GN=Npm1 PE=1 SV=1 - [NPM_MOUSE]	Npm1	364.0529032	185.7155562	165.934205	429.7891884	552.0974991	200.0572359	238.5675548	393.9813078	109.1226601	178.7309149	0.2	0.726530612	1.65144547	0.723729334
P07141	Macrophage colony-stimulating factor 1 OS=Mus musculus OX=10090 GN=Csf1 PE=1 SV=2 - [CSF1_MOUSE]	Csf1	43.0347525	56.63710548	76.3273814	83.78655483	131.0749328	76.17671997	58.66641312	97.01273586	16.73882767	29.74310576	0.2	0.726530612	1.653633326	0.725639369
Q9EQH3	Vacuolar protein sorting-associated protein 35 OS=Mus musculus OX=10090 GN=Vps35 PE=1 SV=1 - [VPS35_MOUSE]	Vps35	139.739704	36.31676312	44.22762299	147.7717996	170.0412744	51.16136957	73.42803004	122.9914812	57.56365177	63.19538019	0.2	0.726530612	1.674993611	0.744155593
P47962	60S ribosomal protein L5 OS=Mus musculus OX=10090 GN=Rpl5 PE=1 SV=3 - [RL5_MOUSE]	Rpl5	143.9988676	42.36659774	54.69036512	171.1737483	154.0104307	78.7271997	80.35194349	134.6371262	55.46320423	49.17402548	0.2	0.726530612	1.675592654	0.744671464
O08583	THO complex subunit 4 OS=Mus musculus OX=10090 GN=Alyref PE=1 SV=3 - [THOC4_MOUSE]	Alyref	382.7630182	309.3230133	0	433.3307139	394.9674188	332.3623261	230.6953438	386.8868196	203.1344747	50.9669098	0.2	0.726530612	1.677046503	0.745922694
P53994	Ras-related protein Rab-2A OS=Mus musculus OX=10090 GN=Rab2a PE=1 SV=1 - [RAB2A_MOUSE]	Rab2a	97.45290511	28.62313977	24.26238013	102.0678368	97.76416742	53.29568178	50.11280834	84.37589534	41.05566499	27.0021323	0.2	0.726530612	1.683719156	0.751651517
P54822	Adenylosuccinate lyase OS=Mus musculus OX=10090 GN=Adsl PE=1 SV=2 - [PUR8_MOUSE]	Adsl	272.2440048	78.27961518	65.97329493	282.5159915	290.7166927	128.0432897	138.832305	233.758658	115.7016535	91.64396973	0.2	0.726530612	1.683748304	0.751676492
Q99KP6	Pre-mRNA-processing factor 19 OS=Mus musculus OX=10090 GN=Prpf19 PE=1 SV=1 - [PRP19_MOUSE]	Prpf19	441.6731702	108.306413	108.8166108	455.4421285	452.0413606	202.5705958	219.5987314	370.0180283	192.3222748	145.0236991	0.2	0.726530612	1.684973433	0.752725845
Q9CPV4	Glyoxalase domain-containing protein 4 OS=Mus musculus OX=10090 GN=Glod4 PE=1 SV=1 - [GLOD4_MOUSE]	Glod4	344.448037	132.6209085	116.5355408	413.7476191	395.868547	191.5635525	197.8681621	333.7265729	127.1964212	123.4409112	0.2	0.726530612	1.686610768	0.75412707
P62774	Myotrophin OS=Mus musculus OX=10090 GN=Mtpn PE=1 SV=2 - [MTPN_MOUSE]	Mtpn	361.5476807	126.4515786	167.6059583	435.4235002	429.6219381	240.7692365	218.5350725	368.6048916	125.55029	110.7469213	0.2	0.726530612	1.686708167	0.754210381
P30416	Peptidyl-prolyl cis-trans isomerase FKBP4 OS=Mus musculus OX=10090 GN=Fkbp4 PE=1 SV=5 - [FKBP4_MOUSE]	Fkbp4	304.7000838	77.29473205	76.50158772	326.6452397	316.134718	130.772095	152.8321345	257.8506842	131.5221	110.1786896	0.2	0.726530612	1.687149663	0.754587957
P97855	Ras GTPase-activating protein-binding protein 1 OS=Mus musculus OX=10090 GN=G3bp1 PE=1 SV=1 - [G3BP1_MOUSE]	G3bp1	334.0907769	86.93833929	113.1029778	391.0612896	357.0717375	153.1673715	178.0440313	300.4334662	135.7721885	128.6635123	0.2	0.726530612	1.687411052	0.754811456
Q9Z2X1	Heterogeneous nuclear ribonucleoprotein F OS=Mus musculus OX=10090 GN=Hnrnpf PE=1 SV=3 - [HNRPF_MOUSE]	Hnrnpf	634.5632518	173.3139506	217.841273	646.4527709	758.8637516	338.4561773	341.9061585	581.2575666	254.4244511	217.6544122	0.2	0.726530612	1.700050005	0.765577183
Q8C854	Myelin expression factor 2 OS=Mus musculus OX=10090 GN=Myef2 PE=1 SV=1 - [MYEF2_MOUSE]	Myef2	20.99744589	10.61977336	13.08283956	30.36798138	27.77568855	17.99796046	14.9000196	25.38054346	5.422234712	6.523564548	0.2	0.726530612	1.703389938	0.768408733
Q99LS3	Phosphoserine phosphatase OS=Mus musculus OX=10090 GN=Psph PE=1 SV=1 - [SERB_MOUSE]	Psph	296.7792596	108.0280667	111.5524351	324.2716871	338.8138825	218.5410874	172.1199205	293.8755523	107.9725355	65.64548783	0.2	0.726530612	1.707388381	0.771791267
P27612	Phospholipase A-2-activating protein OS=Mus musculus OX=10090 GN=Plaa PE=1 SV=4 - [PLAP_MOUSE]	Plaa	95.64883771	37.00903232	23.04095954	115.9761359	103.4732733	46.5504843	51.89960985	88.66663117	38.52626335	37.00550991	0.2	0.726530612	1.708425775	0.772667569
Q91WJ8	Far upstream element-binding protein 1 OS=Mus musculus OX=10090 GN=Fubp1 PE=1 SV=1 - [FUBP1_MOUSE]	Fubp1	329.0723712	94.76950547	72.44960672	332.5810846	335.7631473	179.9772006	165.4304945	282.7738108	142.1567527	89.03869211	0.2	0.726530612	1.709320955	0.773423314
P06801	NADP-dependent malic enzyme OS=Mus musculus OX=10090 GN=Me1 PE=1 SV=2 - [MAOX_MOUSE]	Me1	484.163655	148.3882136	159.3580098	547.6994873	568.8204315	239.0599279	263.9699594	451.8599489	190.7721986	184.592552	0.2	0.726530612	1.7117855	0.775501932
P70168	Importin subunit beta-1 OS=Mus musculus OX=10090 GN=Kpnb1 PE=1 SV=2 - [IMB1_MOUSE]	Kpnb1	466.616783	151.7217574	181.0552034	517.4299167	564.480343	293.490381	266.4645813	458.4668802	173.9562895	144.7976852	0.2	0.726530612	1.720554672	0.782873736
P52480	Pyruvate kinase PKM OS=Mus musculus OX=10090 GN=Pkm PE=1 SV=4 - [KPYM_MOUSE]	Pkm	3413.421777	709.4749169	916.4994077	3552.683123	3539.769576	1582.130562	1679.798701	2891.527754	1504.92575	1133.989614	0.2	0.726530612	1.721353727	0.783543592
Q61205	Platelet-activating factor acetylhydrolase IB subunit alpha1 OS=Mus musculus OX=10090 GN=Pafah1b3 PE=1 SV=1 - [PA1B3_MOUSE]	Pafah1b3	150.7706106	57.25099618	55.18935483	152.3189964	212.8954421	90.92922361	87.7369872	152.0478874	54.59845098	60.98356123	0.2	0.726530612	1.73299645	0.793268699
Q9CZD3	Glycine--tRNA ligase OS=Mus musculus OX=10090 GN=Gars1 PE=1 SV=1 - [GARS_MOUSE]	Gars1	204.6085652	51.03686396	62.56565995	222.5524873	229.4254073	99.88008524	106.070363	183.95266	85.53105428	72.89003788	0.2	0.726530612	1.734251253	0.794312927
O88342	WD repeat-containing protein 1 OS=Mus musculus OX=10090 GN=Wdr1 PE=1 SV=3 - [WDR1_MOUSE]	Wdr1	746.039141	367.4839981	359.9351976	1006.736163	1015.315039	539.7813739	491.1527789	853.9441918	220.7703315	272.1067923	0.2	0.726530612	1.738652877	0.797969926
Q8C0M9	Isoaspartyl peptidase/L-asparaginase OS=Mus musculus OX=10090 GN=Asrgl1 PE=1 SV=1 - [ASGL1_MOUSE]	Asrgl1	221.6370553	143.5694113	129.5994061	288.4580038	358.2568812	214.3089416	164.9352909	287.0079422	49.59947403	71.98492438	0.2	0.726530612	1.740124509	0.799190538
Q9DAK9	14 kDa phosphohistidine phosphatase OS=Mus musculus OX=10090 GN=Phpt1 PE=1 SV=1 - [PHP14_MOUSE]	Phpt1	188.5643121	61.18038446	38.58732236	214.9900947	188.8024351	99.08938757	96.11067296	167.6273058	80.86017655	60.78267065	0.2	0.726530612	1.744107086	0.802488623
Q00612	Glucose-6-phosphate 1-dehydrogenase X OS=Mus musculus OX=10090 GN=G6pdx PE=1 SV=3 - [G6PD1_MOUSE]	G6pdx	118.9822259	36.49116223	41.09674252	133.2321075	142.5403647	67.06765408	65.52337688	114.2800421	46.35395609	41.15116158	0.2	0.726530612	1.744110996	0.802491857
Q99L47	Hsc70-interacting protein OS=Mus musculus OX=10090 GN=St13 PE=1 SV=1 - [F10A1_MOUSE]	St13	132.8395634	84.1692808	35.72744319	142.1063635	196.7337639	102.7327286	84.24542915	147.190952	48.55610491	47.20633898	0.2	0.726530612	1.747168404	0.805018672
P60843	Eukaryotic initiation factor 4A-I OS=Mus musculus OX=10090 GN=Eif4a1 PE=1 SV=1 - [IF4A1_MOUSE]	Eif4a1	752.9289593	258.9281144	255.0019055	809.1881333	948.2664099	459.7722416	422.2863264	739.0755949	286.3516488	251.6812749	0.2	0.726530612	1.750176477	0.807500402
Q8BJW6	Eukaryotic translation initiation factor 2A OS=Mus musculus OX=10090 GN=Eif2a PE=1 SV=2 - [EIF2A_MOUSE]	Eif2a	136.9747517	38.26039376	36.97270447	157.3722366	157.040443	57.27052242	70.73594996	123.8944007	57.36809805	57.69820955	0.2	0.726530612	1.751505433	0.808595463
Q62318	Transcription intermediary factor 1-beta OS=Mus musculus OX=10090 GN=Trim28 PE=1 SV=3 - [TIF1B_MOUSE]	Trim28	248.3105712	65.43253891	52.57763736	265.0804257	270.4358939	107.0259987	122.1069158	214.1807728	109.4844012	92.83738182	0.2	0.726530612	1.754042933	0.81068406
Q62465	Synaptic vesicle membrane protein VAT-1 homolog OS=Mus musculus OX=10090 GN=Vat1 PE=1 SV=3 - [VAT1_MOUSE]	Vat1	95.97805974	29.83121209	38.24945367	103.9340225	126.8589415	58.56219312	54.68624183	96.45171904	36.00662949	34.75773452	0.2	0.726530612	1.763729154	0.818629031
O35405	5'-3' exonuclease PLD3 OS=Mus musculus OX=10090 GN=Pld3 PE=1 SV=1 - [PLD3_MOUSE]	Pld3	149.6390122	114.5951994	101.010684	243.6270951	273.0619244	128.5107569	121.7482986	215.0665921	25.09090866	76.39068497	0.2	0.726530612	1.766485402	0.820881827
Q921M7	CYFIP-related Rac1 interactor B OS=Mus musculus OX=10090 GN=Cyrib PE=1 SV=1 - [CYRIB_MOUSE]	Cyrib	150.1449247	57.72688159	63.3698869	232.1288545	164.713807	82.58141733	90.41389775	159.8080263	51.80547816	74.89431891	0.2	0.726530612	1.76751617	0.821723414
Q91YR1	Twinfilin-1 OS=Mus musculus OX=10090 GN=Twf1 PE=1 SV=2 - [TWF1_MOUSE]	Twf1	177.6043421	60.66386386	61.9814048	202.4035387	214.8197229	113.8641134	100.0832036	177.029125	67.1385073	55.05365042	0.2	0.726530612	1.768819529	0.822786859
Q8VCT3	Aminopeptidase B OS=Mus musculus OX=10090 GN=Rnpep PE=1 SV=2 - [AMPB_MOUSE]	Rnpep	124.6708677	44.51742084	60.75247216	169.4172729	166.7575687	70.94527967	76.64692025	135.7067071	42.37474334	56.1008054	0.2	0.726530612	1.770543508	0.824192296
P22907	Porphobilinogen deaminase OS=Mus musculus OX=10090 GN=Hmbs PE=1 SV=2 - [HEM3_MOUSE]	Hmbs	53.64368396	14.53042708	16.55039582	64.87455579	69.25750137	16.92188963	28.24150228	50.3513156	22.02210693	29.03355719	0.2	0.726530612	1.782883753	0.83421264
Q9DAR7	m7GpppX diphosphatase OS=Mus musculus OX=10090 GN=Dcps PE=1 SV=1 - [DCPS_MOUSE]	Dcps	208.1978945	85.88176053	75.79247272	278.1850856	243.9753841	138.2760664	123.2907092	220.1455121	73.70462	72.9351117	0.2	0.726530612	1.785580709	0.836393346
Q8CI33	CWF19-like protein 1 OS=Mus musculus OX=10090 GN=Cwf19l1 PE=1 SV=2 - [C19L1_MOUSE]	Cwf19l1	27.33035708	5.454121285	7.673878173	30.66428874	32.77577507	9.227750328	13.48611885	24.22260471	12.04072379	13.02876959	0.2	0.726530612	1.79611384	0.844878793
Q9QXA5	U6 snRNA-associated Sm-like protein LSm4 OS=Mus musculus OX=10090 GN=Lsm4 PE=1 SV=1 - [LSM4_MOUSE]	Lsm4	122.1172647	19.11936593	23.09457181	135.1026503	127.2714948	32.83462432	54.77706749	98.40292316	58.35218238	56.91865343	0.2	0.726530612	1.796425542	0.84512914
Q99KK7	Dipeptidyl peptidase 3 OS=Mus musculus OX=10090 GN=Dpp3 PE=1 SV=2 - [DPP3_MOUSE]	Dpp3	223.2776999	75.3653039	96.48475067	286.4519722	280.1809617	145.318439	131.7092515	237.3171243	80.00058324	79.73487298	0.2	0.726530612	1.801825776	0.849459519
Q9R0P9	Ubiquitin carboxyl-terminal hydrolase isozyme L1 OS=Mus musculus OX=10090 GN=Uchl1 PE=1 SV=1 - [UCHL1_MOUSE]	Uchl1	1160.435501	608.9377437	616.5670695	1694.946459	1649.72409	960.9065712	795.3134379	1435.192374	316.2279908	411.3654487	0.2	0.726530612	1.804561956	0.851648676
P28271	Cytoplasmic aconitate hydratase OS=Mus musculus OX=10090 GN=Aco1 PE=1 SV=3 - [ACOC_MOUSE]	Aco1	188.6117503	23.08371151	70.56515394	197.5684343	224.1233123	88.03827139	94.08687192	169.910006	85.23400918	72.13547035	0.2	0.726530612	1.805884312	0.852705475
Q8BGQ7	Alanine--tRNA ligase, cytoplasmic OS=Mus musculus OX=10090 GN=Aars1 PE=1 SV=1 - [SYAC_MOUSE]	Aars1	143.5203886	30.70763141	36.75537959	157.7173239	159.4996147	64.30403861	70.32779988	127.1736591	63.45872755	54.45398079	0.2	0.726530612	1.808298558	0.854632892
Q9QZ73	DCN1-like protein 1 OS=Mus musculus OX=10090 GN=Dcun1d1 PE=2 SV=1 - [DCNL1_MOUSE]	Dcun1d1	58.12538289	12.40289576	12.55284062	59.02158325	60.96501152	30.69104252	27.69370642	50.2258791	26.35471154	16.94554834	0.2	0.726530612	1.813620695	0.858872759
Q9WV55	Vesicle-associated membrane protein-associated protein A OS=Mus musculus OX=10090 GN=Vapa PE=1 SV=2 - [VAPA_MOUSE]	Vapa	302.2087332	94.23165189	98.54267604	369.9673759	365.1887952	163.0731343	164.9943537	299.4097684	118.8506865	118.0951611	0.2	0.726530612	1.814666755	0.859704636
Q60864	Stress-induced-phosphoprotein 1 OS=Mus musculus OX=10090 GN=Stip1 PE=1 SV=1 - [STIP1_MOUSE]	Stip1	297.0002728	65.50783686	85.64857491	339.2251513	317.7292629	159.6232096	149.3855615	272.1925413	128.2341201	98.0785865	0.2	0.726530612	1.822080652	0.865586819
P28352	DNA-(apurinic or apyrimidinic site) endonuclease OS=Mus musculus OX=10090 GN=Apex1 PE=1 SV=2 - [APEX1_MOUSE]	Apex1	384.5143059	66.79865279	116.986614	472.0384329	405.7102378	163.6810178	189.4331909	347.1432295	170.7986796	162.307254	0.2	0.726530612	1.832536462	0.873841904
P54923	[Protein ADP-ribosylarginine] hydrolase OS=Mus musculus OX=10090 GN=Adprh PE=1 SV=1 - [ADPRH_MOUSE]	Adprh	189.4926932	96.86455429	93.1919281	248.0764062	293.5374826	155.8016772	126.5163918	232.4718553	54.56998205	70.18129757	0.2	0.726530612	1.837484075	0.877731747
P47753	F-actin-capping protein subunit alpha-1 OS=Mus musculus OX=10090 GN=Capza1 PE=1 SV=4 - [CAZA1_MOUSE]	Capza1	345.3228673	99.03222237	111.3592097	428.9222523	414.3564623	182.6349791	185.2380998	341.9712312	138.774415	138.1812993	0.2	0.726530612	1.846117141	0.884494099
P56959	RNA-binding protein FUS OS=Mus musculus OX=10090 GN=Fus PE=1 SV=1 - [FUS_MOUSE]	Fus	736.6040787	188.8568277	65.57281772	783.5713858	822.6395753	231.9287819	330.3445747	612.7132477	357.1901807	330.3470718	0.2	0.726530612	1.854770124	0.891240393
Q9QYG0	Protein NDRG2 OS=Mus musculus OX=10090 GN=Ndrg2 PE=1 SV=1 - [NDRG2_MOUSE]	Ndrg2	178.9045224	88.58869531	101.3833774	282.4335646	266.9072519	136.2426985	122.958865	228.5278384	48.87088504	80.29742755	0.2	0.726530612	1.858571469	0.894194166
P50247	Adenosylhomocysteinase OS=Mus musculus OX=10090 GN=Ahcy PE=1 SV=3 - [SAHH_MOUSE]	Ahcy	388.0462572	86.18641262	103.7167369	410.9921217	438.1827637	225.1742773	192.6498022	358.1163876	169.4451505	115.9311704	0.2	0.726530612	1.858898288	0.894447834
O88844	Isocitrate dehydrogenase [NADP] cytoplasmic OS=Mus musculus OX=10090 GN=Idh1 PE=1 SV=2 - [IDHC_MOUSE]	Idh1	749.4338531	150.9932046	145.5519537	792.8292869	778.0719654	385.1675973	348.6596705	652.0229498	347.0912861	231.2212772	0.2	0.726530612	1.870084226	0.903103248
Q91WP6	Serine protease inhibitor A3N OS=Mus musculus OX=10090 GN=Serpina3n PE=1 SV=1 - [SPA3N_MOUSE]	Serpina3n	1148.727693	428.1453307	635.4271392	1613.234955	1804.640319	726.0331787	737.4333875	1381.302818	370.9631937	575.4934648	0.2	0.726530612	1.873122157	0.905444989
Q9JKB1	Ubiquitin carboxyl-terminal hydrolase isozyme L3 OS=Mus musculus OX=10090 GN=Uchl3 PE=1 SV=2 - [UCHL3_MOUSE]	Uchl3	151.2843982	56.90531753	56.55399901	232.731387	190.5654074	73.25370366	88.2479049	165.5168327	54.59148713	82.63689365	0.2	0.726530612	1.875589374	0.907344011
P84091	AP-2 complex subunit mu OS=Mus musculus OX=10090 GN=Ap2m1 PE=1 SV=1 - [AP2M1_MOUSE]	Ap2m1	94.96899378	42.0818143	38.32524222	95.06341819	162.030063	72.22684064	58.45868343	109.7734406	31.67459596	46.67379625	0.2	0.726530612	1.877795294	0.909039797
Q62048	Astrocytic phosphoprotein PEA-15 OS=Mus musculus OX=10090 GN=Pea15 PE=1 SV=1 - [PEA15_MOUSE]	Pea15	448.4022116	123.8694372	190.7572163	494.933088	600.2380241	337.9107511	254.342955	477.6939544	171.3555953	132.010569	0.2	0.726530612	1.878148952	0.909311485
Q8CAY6	Acetyl-CoA acetyltransferase, cytosolic OS=Mus musculus OX=10090 GN=Acat2 PE=1 SV=2 - [THIC_MOUSE]	Acat2	283.7517908	69.28901526	71.71365704	356.7317656	304.1326949	138.4684645	141.584821	266.4443083	123.1261759	113.9079805	0.2	0.726530612	1.881870573	0.912167409
Q3UGR5	Haloacid dehalogenase-like hydrolase domain-containing protein 2 OS=Mus musculus OX=10090 GN=Hdhd2 PE=1 SV=2 - [HDHD2_MOUSE]	Hdhd2	275.6618702	45.56951976	135.0746226	367.6490397	344.3382277	152.1122518	152.1020042	288.0331731	115.9873753	118.2866058	0.2	0.726530612	1.893684272	0.921195815
Q61553	Fascin OS=Mus musculus OX=10090 GN=Fscn1 PE=1 SV=4 - [FSCN1_MOUSE]	Fscn1	1300.563527	465.8162633	489.8870844	1603.820364	1760.202379	911.8408333	752.0889582	1425.287859	475.1453624	451.480586	0.2	0.726530612	1.895105417	0.922278102
Q11011	Puromycin-sensitive aminopeptidase OS=Mus musculus OX=10090 GN=Npepps PE=1 SV=2 - [PSA_MOUSE]	Npepps	410.0077853	109.0755527	133.0054747	506.8631712	495.6471781	233.6401701	217.3629375	412.0501731	167.2638285	154.6093352	0.2	0.726530612	1.895678158	0.922714049
P63005	Platelet-activating factor acetylhydrolase IB subunit beta OS=Mus musculus OX=10090 GN=Pafah1b1 PE=1 SV=2 - [LIS1_MOUSE]	Pafah1b1	150.8104954	95.10612582	0	203.0104845	158.2876821	107.8431237	81.97220707	156.3804301	76.25828641	47.61233926	0.2	0.726530612	1.907725017	0.931853233
P46664	Adenylosuccinate synthetase isozyme 2 OS=Mus musculus OX=10090 GN=Adss2 PE=1 SV=2 - [PURA2_MOUSE]	Adss2	176.4159904	58.89460363	83.85472165	238.155637	252.3458681	121.1966893	106.3884386	203.8993981	61.91643873	71.97321861	0.2	0.726530612	1.91655598	0.938516138
P17426	AP-2 complex subunit alpha-1 OS=Mus musculus OX=10090 GN=Ap2a1 PE=1 SV=1 - [AP2A1_MOUSE]	Ap2a1	156.6684453	29.41046042	58.34641782	168.2072818	200.984545	99.83512113	81.47510784	156.342316	66.7072028	51.60798921	0.2	0.726530612	1.918896705	0.940277053
O08709	Peroxiredoxin-6 OS=Mus musculus OX=10090 GN=Prdx6 PE=1 SV=3 - [PRDX6_MOUSE]	Prdx6	1354.532044	553.2413884	892.0611414	1926.840285	2502.993303	944.1250284	933.2781914	1791.319539	402.2322871	788.2207677	0.2	0.726530612	1.919384333	0.940643622
P51859	Hepatoma-derived growth factor OS=Mus musculus OX=10090 GN=Hdgf PE=1 SV=2 - [HDGF_MOUSE]	Hdgf	298.4552141	103.43367	79.92566992	346.4595523	365.3582323	213.9263503	160.6048514	308.5813783	119.9591531	82.51648789	0.2	0.726530612	1.921370218	0.942135531
Q61220	Protein kinase C-binding protein NELL2 OS=Mus musculus OX=10090 GN=Nell2 PE=1 SV=3 - [NELL2_MOUSE]	Nell2	94.5353009	43.98279635	49.92419531	130.1856664	155.2075628	76.9209564	62.81409752	120.7713952	27.6315239	39.98336606	0.2	0.726530612	1.92267978	0.943118503
P56399	Ubiquitin carboxyl-terminal hydrolase 5 OS=Mus musculus OX=10090 GN=Usp5 PE=1 SV=1 - [UBP5_MOUSE]	Usp5	190.7305155	26.65729579	37.8933283	216.7205893	212.3136978	61.93616833	85.0937132	163.6568185	91.65649246	88.12022	0.2	0.726530612	1.923253932	0.943549258
P15105	Glutamine synthetase OS=Mus musculus OX=10090 GN=Glul PE=1 SV=6 - [GLNA_MOUSE]	Glul	308.679412	83.20063466	104.3801518	384.3060959	364.7302688	207.9154403	165.4200662	318.983935	124.5173607	96.68485475	0.2	0.726530612	1.928326728	0.947349517
P17182	Alpha-enolase OS=Mus musculus OX=10090 GN=Eno1 PE=1 SV=3 - [ENOA_MOUSE]	Eno1	6225.588145	1454.458894	1855.744444	7721.062597	7181.72503	3517.36482	3178.597161	6140.050815	2646.388684	2287.265316	0.2	0.726530612	1.931685742	0.949860407
P14206	40S ribosomal protein SA OS=Mus musculus OX=10090 GN=Rpsa PE=1 SV=4 - [RSSA_MOUSE]	Rpsa	875.3644457	178.8710357	182.3479523	970.9640671	1066.127255	361.5885329	412.1944779	799.5599518	401.1207257	382.2672244	0.2	0.726530612	1.93976386	0.955881035
Q61035	Histidine--tRNA ligase, cytoplasmic OS=Mus musculus OX=10090 GN=Hars1 PE=1 SV=2 - [HARS1_MOUSE]	Hars1	286.6709344	134.699353	155.6352947	467.5583411	464.7842235	190.3494851	192.335194	374.2306833	82.36505541	159.2518295	0.2	0.726530612	1.945721297	0.960305075
Q61166	Microtubule-associated protein RP/EB family member 1 OS=Mus musculus OX=10090 GN=Mapre1 PE=1 SV=3 - [MARE1_MOUSE]	Mapre1	418.1624293	88.67979219	149.8387624	477.6534422	489.9083363	312.6124707	218.8936613	426.7247497	175.2601961	99.01391209	0.2	0.726530612	1.949461429	0.963075611
Q9CR16	Peptidyl-prolyl cis-trans isomerase D OS=Mus musculus OX=10090 GN=Ppid PE=1 SV=3 - [PPID_MOUSE]	Ppid	354.1891802	66.10497437	83.21501022	428.0294275	370.0995747	186.9728036	167.8363883	328.3672686	161.6128418	125.8302945	0.2	0.726530612	1.956472443	0.96825479
Q68FD5	Clathrin heavy chain 1 OS=Mus musculus OX=10090 GN=Cltc PE=1 SV=3 - [CLH1_MOUSE]	Cltc	697.5735232	201.9063156	331.3307372	907.9048337	1013.901007	486.6045484	410.270192	802.803463	257.0896212	278.9177519	0.2	0.726530612	1.956767707	0.9684725
Q9CR09	Ubiquitin-fold modifier-conjugating enzyme 1 OS=Mus musculus OX=10090 GN=Ufc1 PE=1 SV=1 - [UFC1_MOUSE]	Ufc1	90.37218915	37.47517205	41.32161475	136.1452625	142.4583411	53.10334333	56.38965865	110.5689823	29.49250863	49.86670722	0.2	0.726530612	1.960802476	0.971444211
Q9R0Q7	Prostaglandin E synthase 3 OS=Mus musculus OX=10090 GN=Ptges3 PE=1 SV=1 - [TEBP_MOUSE]	Ptges3	96.93889238	35.52142583	40.39103611	94.0232389	129.6716799	115.3865973	57.6171181	113.027172	34.14058765	17.94095879	0.2	0.726530612	1.961694298	0.972100235
P07901	Heat shock protein HSP 90-alpha OS=Mus musculus OX=10090 GN=Hsp90aa1 PE=1 SV=4 - [HS90A_MOUSE]	Hsp90aa1	1242.244979	348.1450438	420.7744839	1343.478657	1884.173449	718.4687128	670.3881688	1315.373606	496.5721672	583.3603549	0.2	0.726530612	1.962107429	0.972404034
P11499	Heat shock protein HSP 90-beta OS=Mus musculus OX=10090 GN=Hsp90ab1 PE=1 SV=3 - [HS90B_MOUSE]	Hsp90ab1	1314.817299	353.3525366	439.990239	1376.08679	1996.610005	772.0110402	702.7200247	1381.569278	531.8588416	612.3178907	0.2	0.726530612	1.966030894	0.975285992
Q9D0L8	mRNA cap guanine-N7 methyltransferase OS=Mus musculus OX=10090 GN=Rnmt PE=1 SV=1 - [MCES_MOUSE]	Rnmt	46.84209799	10.90459325	7.288042888	54.12988738	56.59706043	17.62758312	21.67824471	42.78484364	21.86742996	21.82172206	0.2	0.726530612	1.97363044	0.980851872
P97379	Ras GTPase-activating protein-binding protein 2 OS=Mus musculus OX=10090 GN=G3bp2 PE=1 SV=2 - [G3BP2_MOUSE]	G3bp2	66.4871499	26.47898575	0	70.97072315	69.85899348	42.72747843	30.98871188	61.18573169	33.47220485	15.99497799	0.2	0.726530612	1.974452243	0.981452473
P10649	Glutathione S-transferase Mu 1 OS=Mus musculus OX=10090 GN=Gstm1 PE=1 SV=2 - [GSTM1_MOUSE]	Gstm1	2521.85313	953.1077836	1088.388205	3653.69264	3715.831707	1653.687349	1521.116373	3007.737232	869.2989937	1173.053123	0.2	0.726530612	1.977322239	0.983548002
Q9Z2N8	Actin-like protein 6A OS=Mus musculus OX=10090 GN=Actl6a PE=1 SV=2 - [ACL6A_MOUSE]	Actl6a	148.5346822	45.64795898	37.3389349	189.2795798	184.0783766	86.49672925	77.17385869	153.2848952	61.93977169	57.89868275	0.2	0.726530612	1.98622821	0.990031393
Q60668	Heterogeneous nuclear ribonucleoprotein D0 OS=Mus musculus OX=10090 GN=Hnrnpd PE=1 SV=2 - [HNRPD_MOUSE]	Hnrnpd	1087.473088	247.9610663	250.3744955	1285.960117	1250.960535	615.9707583	528.6028834	1050.963804	483.9972992	377.1212734	0.2	0.726530612	1.988191583	0.991456783
Q99KI0	Aconitate hydratase, mitochondrial OS=Mus musculus OX=10090 GN=Aco2 PE=1 SV=1 - [ACON_MOUSE]	Aco2	296.7908214	38.66530738	54.86569234	328.6529486	310.4981538	136.946172	130.1072737	258.6990915	144.5792755	105.8311353	0.2	0.726530612	1.988352258	0.991573369
Q8BJY1	26S proteasome non-ATPase regulatory subunit 5 OS=Mus musculus OX=10090 GN=Psmd5 PE=1 SV=4 - [PSMD5_MOUSE]	Psmd5	154.7997118	68.1371075	43.66719113	217.9571586	224.7243071	87.6688805	88.86800349	176.7834487	58.39466437	77.24961669	0.2	0.726530612	1.989281201	0.992247227
P62827	GTP-binding nuclear protein Ran OS=Mus musculus OX=10090 GN=Ran PE=1 SV=3 - [RAN_MOUSE]	Ran	1474.493408	179.7322098	341.6668822	1540.409788	1692.820842	749.6848748	665.2974999	1327.638502	705.4461159	506.2905078	0.2	0.726530612	1.995556126	0.996790856
P47757	F-actin-capping protein subunit beta OS=Mus musculus OX=10090 GN=Capzb PE=1 SV=3 - [CAPZB_MOUSE]	Capzb	212.0839218	82.16190385	84.02361619	306.8856982	302.2065471	147.9651834	126.089814	252.3524762	74.47889925	90.43231612	0.2	0.726530612	2.001370835	1.00098851
Q9R1P1	Proteasome subunit beta type-3 OS=Mus musculus OX=10090 GN=Psmb3 PE=1 SV=1 - [PSB3_MOUSE]	Psmb3	252.2726547	152.1382886	120.9468808	434.7939439	416.9606216	212.3539758	175.1192747	354.7028471	68.61274676	123.5997885	0.2	0.726530612	2.025492897	1.018273026
P08226	Apolipoprotein E OS=Mus musculus OX=10090 GN=Apoe PE=1 SV=2 - [APOE_MOUSE]	Apoe	8902.043885	4441.124712	4802.194078	14825.08179	14873.71606	7152.795107	6048.454225	12283.86432	2477.86667	4443.702822	0.2	0.726530612	2.030909694	1.022126091
Q9DBF1	Alpha-aminoadipic semialdehyde dehydrogenase OS=Mus musculus OX=10090 GN=Aldh7a1 PE=1 SV=4 - [AL7A1_MOUSE]	Aldh7a1	223.5236768	67.67138789	83.4459535	294.5553543	344.638159	121.7055358	124.8803394	253.6330164	85.79096829	116.9645969	0.2	0.726530612	2.031008384	1.022196195

Q64674	Spermidine synthase OS=Mus musculus OX=10090 GN=Srm PE=1 SV=1 - [SPEE_MOUSE]	Srm	268.7459904	54.260421	66.57627594	322.6363063	336.4764911	133.7706447	129.8608958	264.2944807	120.4355521	113.2485828	0.2	0.726530612	2.03521221	1.025179231
Q8R3Y8	Interferon regulatory factor 2-binding protein 1 OS=Mus musculus OX=10090 GN=Irf2bp1 PE=1 SV=2 - [I2BP1_MOUSE]	Irf2bp1	42.85654324	15.07465784	0	44.56835921	47.99542268	25.59000203	19.31040036	39.38459464	21.73998529	12.0687315	0.2	0.726530612	2.0395535	1.028253351
P62908	40S ribosomal protein S3 OS=Mus musculus OX=10090 GN=Rps3 PE=1 SV=1 - [RS3_MOUSE]	Rps3	250.3957456	40.86067168	32.66530711	267.5780384	272.6636347	122.3259648	107.9739081	220.8558793	123.408978	85.367288	0.2	0.726530612	2.04545601	1.03242251
Q9QXS1	Plectin OS=Mus musculus OX=10090 GN=Plec PE=1 SV=3 - [PLEC_MOUSE]	Plec	232.3467437	108.444802	126.4641969	331.3396899	440.4036441	186.986617	155.7519142	319.5766503	66.94214348	127.117364	0.2	0.726530612	2.051831285	1.036912108
Q99K48	Non-POU domain-containing octamer-binding protein OS=Mus musculus OX=10090 GN=Nono PE=1 SV=3 - [NONO_MOUSE]	Nono	162.694757	24.48818415	14.31654834	183.417858	189.7879074	41.71564765	67.16649651	138.3071377	82.88607822	83.71129761	0.2	0.726530612	2.059168557	1.042061929
P56375	Acylphosphatase-2 OS=Mus musculus OX=10090 GN=Acyp2 PE=1 SV=2 - [ACYP2_MOUSE]	Acyp2	31.92854492	12.44504226	0	34.39732116	34.82620717	22.19902751	14.79119573	30.47418528	16.09305199	7.169704521	0.2	0.726530612	2.060292207	1.042848966
P63280	SUMO-conjugating enzyme UBC9 OS=Mus musculus OX=10090 GN=Ube2i PE=1 SV=1 - [UBC9_MOUSE]	Ube2i	319.8214348	44.81549972	99.80418785	422.922602	333.3663675	206.3357375	154.8137075	320.8749023	145.5218249	108.832417	0.2	0.726530612	2.072651754	1.051477736
Q9D819	Inorganic pyrophosphatase OS=Mus musculus OX=10090 GN=Ppa1 PE=1 SV=1 - [IPYR_MOUSE]	Ppa1	271.1551806	0	100.0523384	326.8848841	292.0249557	152.6286135	123.7358397	257.1794845	137.1202544	92.20611214	0.2	0.726530612	2.078455887	1.055512129
Q76MZ3	Serine/threonine-protein phosphatase 2A 65 kDa regulatory subunit A alpha isoform OS=Mus musculus OX=10090 GN=Ppp2r1a PE=1 SV=3 - [2AAA_MOUS	Ppp2r1a	500.6020112	121.7524706	159.3754308	656.9643274	632.477433	339.2992488	260.5766375	542.9136697	208.7175287	176.7597983	0.2	0.726530612	2.083508617	1.059015067
Q80X50	Ubiquitin-associated protein 2-like OS=Mus musculus OX=10090 GN=Ubap2l PE=1 SV=1 - [UBP2L_MOUSE]	Ubap2l	64.33508887	23.21546567	0	73.76709512	76.01951585	32.69182641	29.18351818	60.82614579	32.58011899	24.39104949	0.2	0.726530612	2.084263639	1.059537776
P62267	40S ribosomal protein S23 OS=Mus musculus OX=10090 GN=Rps23 PE=1 SV=3 - [RS23_MOUSE]	Rps23	74.92601153	33.80633124	35.17516748	115.7120436	111.0686227	73.52417181	47.96917009	100.1016127	23.35533994	23.13353868	0.2	0.726530612	2.086790589	1.061285832
P45376	Aldo-keto reductase family 1 member B1 OS=Mus musculus OX=10090 GN=Akr1b1 PE=1 SV=3 - [ALDR_MOUSE]	Akr1b1	450.0913323	86.44855819	79.41328959	574.4820041	489.4540823	226.6352642	205.3177267	430.1904502	212.0093447	181.3380053	0.2	0.726530612	2.095242613	1.067117307
P17918	Proliferating cell nuclear antigen OS=Mus musculus OX=10090 GN=Pcna PE=1 SV=2 - [PCNA_MOUSE]	Pcna	333.5097014	128.1839379	142.0163433	427.7949889	557.7467496	287.3094441	201.2366609	424.2837275	114.7604104	135.2528402	0.2	0.726530612	2.108381871	1.076136192
P17427	AP-2 complex subunit alpha-2 OS=Mus musculus OX=10090 GN=Ap2a2 PE=1 SV=2 - [AP2A2_MOUSE]	Ap2a2	102.8155469	27.45468005	34.40601645	125.5658052	152.9926665	69.0801346	54.89208115	115.8795355	41.64821965	42.78663483	0.2	0.726530612	2.11104285	1.077955863
P10630	Eukaryotic initiation factor 4A-II OS=Mus musculus OX=10090 GN=Eif4a2 PE=1 SV=2 - [IF4A2_MOUSE]	Eif4a2	752.9289593	256.4145017	0	788.7960711	917.4897829	426.6882166	336.4478203	710.9913569	382.7917234	254.48325	0.2	0.726530612	2.113229196	1.079449247
Q8BGB7	Enolase-phosphatase E1 OS=Mus musculus OX=10090 GN=Enoph1 PE=1 SV=1 - [ENOPH_MOUSE]	Enoph1	96.4174728	38.31998221	0	112.6075176	101.2944352	70.92852363	44.912485	94.94349213	48.5456289	21.55308545	0.2	0.726530612	2.113966576	1.079952566
Q8BHG2	CXXC motif containing zinc binding protein OS=Mus musculus OX=10090 GN=Czib PE=1 SV=1 - [CZIB_MOUSE]	Czib	128.3496768	33.2423703	26.8367195	161.017547	138.1213638	99.67090216	62.80958885	132.9366043	56.84967399	31.00022593	0.2	0.726530612	2.116501744	1.081681677
Q9JI46	Diphosphoinositol polyphosphate phosphohydrolase 1 OS=Mus musculus OX=10090 GN=Nudt3 PE=1 SV=1 - [NUDT3_MOUSE]	Nudt3	69.36229322	0	45.88212281	68.95542005	88.8936413	87.74072347	38.41480534	81.8632616	35.27892505	11.19337237	0.2	0.726530612	2.131034138	1.091553705
Q9QUR6	Prolyl endopeptidase OS=Mus musculus OX=10090 GN=Prep PE=1 SV=1 - [PPCE_MOUSE]	Prep	288.2599121	56.51251877	72.48058722	386.2103734	348.492633	156.2966697	139.0843394	296.999892	129.4363103	123.3033031	0.2	0.726530612	2.135394203	1.094502422
Q7TMK9	Heterogeneous nuclear ribonucleoprotein Q OS=Mus musculus OX=10090 GN=Syncrip PE=1 SV=2 - [HNRPQ_MOUSE]	Syncrip	295.7200485	50.44766903	59.57170666	327.9371003	414.4050572	129.4934704	135.2464747	290.611876	139.0490486	146.0771452	0.2	0.726530612	2.148757493	1.103502671
Q9D0B6	Protein PBDC1 OS=Mus musculus OX=10090 GN=Pbdc1 PE=1 SV=1 - [PBDC1_MOUSE]	Pbdc1	44.16747762	11.07190775	14.02389996	62.73525958	63.11658205	24.1662424	23.08776178	50.00602801	18.31514072	22.37872297	0.2	0.726530612	2.165910602	1.114973697
Q8BRT1	CLIP-associating protein 2 OS=Mus musculus OX=10090 GN=Clasp2 PE=1 SV=1 - [CLAP2_MOUSE]	Clasp2	40.18746639	0	17.53029563	47.82055137	50.29876186	27.53571559	19.23925401	41.88500961	20.14816413	12.48847712	0.2	0.726530612	2.177059962	1.122381144
P63158	High mobility group protein B1 OS=Mus musculus OX=10090 GN=Hmgb1 PE=1 SV=2 - [HMGB1_MOUSE]	Hmgb1	224.9508769	101.4006708	103.933175	242.369597	491.7194626	207.0136756	143.4282409	313.7009118	70.61202822	155.1788118	0.2	0.726530612	2.187162792	1.129060605
Q9WTL7	Acyl-protein thioesterase 2 OS=Mus musculus OX=10090 GN=Lypla2 PE=1 SV=1 - [LYPA2_MOUSE]	Lypla2	422.4063333	84.07512846	116.4833286	513.2274737	599.8116458	251.139783	207.6549301	454.7263008	186.6847523	181.548361	0.2	0.726530612	2.189817023	1.130810326
Q02053	Ubiquitin-like modifier-activating enzyme 1 OS=Mus musculus OX=10090 GN=Uba1 PE=1 SV=1 - [UBA1_MOUSE]	Uba1	480.5865064	111.6091214	122.8983609	605.063869	710.6943212	251.303515	238.3646629	522.3539017	209.8462004	240.6048259	0.2	0.726530612	2.191406626	1.131857208
Q9CWS0	N(G),N(G)-dimethylarginine dimethylaminohydrolase 1 OS=Mus musculus OX=10090 GN=Ddah1 PE=1 SV=3 - [DDAH1_MOUSE]	Ddah1	1007.793397	99.22820084	366.1335174	1266.126975	1269.585117	696.7569201	491.051705	1077.489671	466.9861818	329.7287678	0.2	0.726530612	2.194248915	1.133727194
Q99K85	Phosphoserine aminotransferase OS=Mus musculus OX=10090 GN=Psat1 PE=1 SV=1 - [SERC_MOUSE]	Psat1	594.1755341	144.4491758	135.6841723	756.8121705	793.7923691	383.2315358	291.4362941	644.6120252	262.2164982	227.1160583	0.2	0.726530612	2.211845396	1.145250547
P24452	Macrophage-capping protein OS=Mus musculus OX=10090 GN=Capg PE=1 SV=2 - [CAPG_MOUSE]	Capg	146.5570495	0	21.56416061	152.0174302	185.1475	35.10269393	56.04040335	124.0892081	79.12774906	78.82480672	0.2	0.726530612	2.214281137	1.146838406
P01887	Beta-2-microglobulin OS=Mus musculus OX=10090 GN=B2m PE=1 SV=2 - [B2MG_MOUSE]	B2m	530.436701	540.9176341	1214.652577	2562.367131	1507.031544	997.542598	762.0023039	1688.980424	392.0416617	798.1215451	0.2	0.726530612	2.216503041	1.148285342
P28656	Nucleosome assembly protein 1-like 1 OS=Mus musculus OX=10090 GN=Nap1l1 PE=1 SV=2 - [NP1L1_MOUSE]	Nap1l1	67.45165978	25.89534451	29.23457125	94.68519863	127.3042462	51.03395689	40.86052518	91.00780058	23.08904368	38.26789387	0.2	0.726530612	2.227279267	1.155282462
P23116	Eukaryotic translation initiation factor 3 subunit A OS=Mus musculus OX=10090 GN=Eif3a PE=1 SV=5 - [EIF3A_MOUSE]	Eif3a	50.79436386	13.84461862	9.390714931	58.06345799	83.01300201	23.96901618	24.6765658	55.01515873	22.72804096	29.63979018	0.2	0.726530612	2.229449558	1.156687559
Q9CPY7	Cytosol aminopeptidase OS=Mus musculus OX=10090 GN=Lap3 PE=1 SV=3 - [AMPL_MOUSE]	Lap3	281.1920314	71.7892665	98.55699222	432.2831041	408.1874584	166.7896101	150.5127634	335.7533908	113.9602172	146.8220673	0.2	0.726530612	2.23073036	1.157516138
O70251	Elongation factor 1-beta OS=Mus musculus OX=10090 GN=Eef1b PE=1 SV=5 - [EF1B_MOUSE]	Eef1b	231.0707039	94.74396248	0	274.0380571	314.7334393	138.1699637	108.6048888	242.31382	116.1572706	92.45802096	0.2	0.726530612	2.231150206	1.157787643
Q61704	Inter-alpha-trypsin inhibitor heavy chain H3 OS=Mus musculus OX=10090 GN=Itih3 PE=1 SV=3 - [ITIH3_MOUSE]	Itih3	0	20.54825353	12.1259889	23.72867701	32.79574986	16.9657366	10.89141414	24.49672116	10.32960851	7.942905561	0.2	0.726530612	2.249177273	1.169397374
P61082	NEDD8-conjugating enzyme Ubc12 OS=Mus musculus OX=10090 GN=Ube2m PE=1 SV=1 - [UBC12_MOUSE]	Ube2m	116.6076545	18.2552655	18.86096403	123.1644887	160.1087225	67.37771621	51.24129468	116.8836425	56.60973827	46.68347301	0.2	0.726530612	2.281043896	1.18969421
Q60817	Nascent polypeptide-associated complex subunit alpha OS=Mus musculus OX=10090 GN=Naca PE=1 SV=1 - [NACA_MOUSE]	Naca	158.7413802	39.73831822	0	176.7442989	192.962867	86.57454275	66.15989949	152.0939029	82.60315622	57.31797625	0.2	0.726530612	2.298883524	1.200933372
Q93092	Transaldolase OS=Mus musculus OX=10090 GN=Taldo1 PE=1 SV=2 - [TALDO_MOUSE]	Taldo1	1045.22342	289.5099885	332.5935644	1429.209693	1681.240139	728.1368996	555.7756578	1279.52891	424.4212329	493.8670859	0.2	0.726530612	2.302239928	1.203038192
Q6ZQ38	Cullin-associated NEDD8-dissociated protein 1 OS=Mus musculus OX=10090 GN=Cand1 PE=1 SV=2 - [CAND1_MOUSE]	Cand1	112.3810137	23.29154519	25.73151104	124.4605538	180.6668267	66.67796315	53.80135665	123.9351146	50.74613803	56.9962483	0.2	0.726530612	2.303568577	1.203870547
Q61545	RNA-binding protein EWS OS=Mus musculus OX=10090 GN=Ewsr1 PE=1 SV=2 - [EWS_MOUSE]	Ewsr1	308.5684251	119.737273	104.1583082	512.8804214	480.6571936	239.9279481	177.4880021	411.1551877	113.785913	149.1598464	0.2	0.726530612	2.316523837	1.211961528
Q9Z1Q5	Chloride intracellular channel protein 1 OS=Mus musculus OX=10090 GN=Clic1 PE=1 SV=3 - [CLIC1_MOUSE]	Clic1	365.0922259	137.825862	157.5099756	554.0943811	656.5429004	320.8954389	220.1426878	510.5109068	125.9152196	172.0158249	0.2	0.726530612	2.319000062	1.213502859
Q9Z1Q9	Valine--tRNA ligase OS=Mus musculus OX=10090 GN=Vars1 PE=1 SV=1 - [SYVC_MOUSE]	Vars1	118.9376942	33.44813921	0	152.0027348	151.4154466	52.87931389	50.79527781	118.7658318	61.33707027	57.06015382	0.2	0.726530612	2.338127418	1.225353553
P63330	Serine/threonine-protein phosphatase 2A catalytic subunit alpha isoform OS=Mus musculus OX=10090 GN=Ppp2ca PE=1 SV=1 - [PP2AA_MOUSE]	Ppp2ca	226.4053823	75.07214055	0	283.3088885	283.7428323	144.3782159	100.4925076	237.1433122	115.323439	80.33722302	0.2	0.726530612	2.359810874	1.23867124
Q9JI75	Ribosyldihydronicotinamide dehydrogenase [quinone] OS=Mus musculus OX=10090 GN=Nqo2 PE=1 SV=3 - [NQO2_MOUSE]	Nqo2	42.86760304	9.974884042	0	57.63237628	43.76596671	23.65676557	17.61416236	41.68503619	22.43160221	17.0831272	0.2	0.726530612	2.36656364	1.242793718
Q922Q8	Leucine-rich repeat-containing protein 59 OS=Mus musculus OX=10090 GN=Lrrc59 PE=1 SV=1 - [LRC59_MOUSE]	Lrrc59	51.61193823	0	21.18854433	62.45257223	79.76833656	32.05045967	24.26682752	58.09045615	25.94330188	24.15615835	0.2	0.726530612	2.393821611	1.259315646
Q8R0Y6	Cytosolic 10-formyltetrahydrofolate dehydrogenase OS=Mus musculus OX=10090 GN=Aldh1l1 PE=1 SV=1 - [AL1L1_MOUSE]	Aldh1l1	820.8190868	150.7735172	205.9922164	1180.371949	1227.895365	426.321357	392.5282735	944.86289	371.9368788	449.6983543	0.2	0.726530612	2.407120592	1.26730842
Q91VI7	Ribonuclease inhibitor OS=Mus musculus OX=10090 GN=Rnh1 PE=1 SV=1 - [RINI_MOUSE]	Rnh1	108.5118881	14.88131825	11.24950058	160.1357867	140.0278203	30.51278366	44.88090232	110.2254636	55.13596187	69.76149425	0.2	0.726530612	2.455954712	1.296283958
P68040	Receptor of activated protein C kinase 1 OS=Mus musculus OX=10090 GN=Rack1 PE=1 SV=3 - [RACK1_MOUSE]	Rack1	657.3558904	151.3119029	190.5231007	977.9132861	1009.397169	487.1085514	333.0636313	824.8063355	281.5288289	292.8782234	0.2	0.726530612	2.476422695	1.308257586
Q9D1E6	Tubulin-folding cofactor B OS=Mus musculus OX=10090 GN=Tbcb PE=1 SV=2 - [TBCB_MOUSE]	Tbcb	73.15558127	11.48978658	0	74.53374522	86.97422208	49.27945357	28.21512261	70.26247362	39.34129404	19.20694443	0.2	0.726530612	2.490241655	1.316285749
P09411	Phosphoglycerate kinase 1 OS=Mus musculus OX=10090 GN=Pgk1 PE=1 SV=4 - [PGK1_MOUSE]	Pgk1	2630.581087	386.3818763	559.8659885	3810.7761	3739.637331	1523.475308	1192.276317	3024.62958	1248.625101	1300.524238	0.2	0.726530612	2.536852856	1.343039842
Q9DCD0	6-phosphogluconate dehydrogenase, decarboxylating OS=Mus musculus OX=10090 GN=Pgd PE=1 SV=3 - [6PGD_MOUSE]	Pgd	499.644395	112.2745712	150.564972	733.3700985	817.2677399	394.7868582	254.1613127	648.4748989	213.454905	223.6692201	0.2	0.726530612	2.551430396	1.351306285
O09172	Glutamate--cysteine ligase regulatory subunit OS=Mus musculus OX=10090 GN=Gclm PE=1 SV=1 - [GSH0_MOUSE]	Gclm	86.98593784	27.15098737	31.52508045	153.85584	151.1916733	67.2163767	48.55400189	124.0879633	33.35481147	49.27024935	0.2	0.726530612	2.555669121	1.353701065
O35098	Dihydropyrimidinase-related protein 4 OS=Mus musculus OX=10090 GN=Dpysl4 PE=1 SV=1 - [DPYL4_MOUSE]	Dpysl4	63.14595371	22.81915849	0	83.21317457	97.59250381	38.99407931	28.6550374	73.2665859	31.97492736	30.53923391	0.2	0.726530612	2.556848378	1.354366611
P70349	Histidine triad nucleotide-binding protein 1 OS=Mus musculus OX=10090 GN=Hint1 PE=1 SV=3 - [HINT1_MOUSE]	Hint1	435.8018411	0	114.4354427	499.2805213	698.2386282	211.4146372	183.4124279	469.6445956	225.9406549	244.7613442	0.2	0.726530612	2.56059309	1.356478009
P09405	Nucleolin OS=Mus musculus OX=10090 GN=Ncl PE=1 SV=2 - [NUCL_MOUSE]	Ncl	111.9715131	19.86836245	67.46110757	117.9938023	303.2501722	97.7092469	66.43366104	172.9844071	46.06017069	113.2684553	0.2	0.726530612	2.603866841	1.380655673
Q8R146	Acylamino-acid-releasing enzyme OS=Mus musculus OX=10090 GN=Apeh PE=1 SV=3 - [APEH_MOUSE]	Apeh	82.29812019	23.1828372	0	106.3004962	101.6530266	69.36526563	35.16031913	92.43959616	42.43630759	20.11761145	0.2	0.726530612	2.629088656	1.394562792
Q8BLK3	Limbic system-associated membrane protein OS=Mus musculus OX=10090 GN=Lsamp PE=1 SV=1 - [LSAMP_MOUSE]	Lsamp	21.07328176	42.9858711	0	79.14044754	63.95595874	25.98140572	21.35305095	56.35927067	21.49430114	27.38162129	0.2	0.726530612	2.63940131	1.400210723
O35381	Acidic leucine-rich nuclear phosphoprotein 32 family member A OS=Mus musculus OX=10090 GN=Anp32a PE=1 SV=1 - [AN32A_MOUSE]	Anp32a	104.9932215	33.27986956	48.83251531	141.6521294	256.5388584	95.96611668	62.36853544	164.7190348	37.7242507	82.73429121	0.2	0.726530612	2.64105985	1.401116995
P62309	Small nuclear ribonucleoprotein G OS=Mus musculus OX=10090 GN=Snrpg PE=1 SV=1 - [RUXG_MOUSE]	Snrpg	41.91194245	23.40499028	0	61.11229201	77.02125668	40.59611651	21.77231091	59.57655507	21.00361782	18.26106719	0.2	0.726530612	2.736345044	1.452250161
O35969	Guanidinoacetate N-methyltransferase OS=Mus musculus OX=10090 GN=Gamt PE=1 SV=1 - [GAMT_MOUSE]	Gamt	54.81229179	7.572304905	7.296207817	75.71800977	100.1751262	16.41749778	23.22693484	64.10354459	27.35406986	43.0697914	0.2	0.726530612	2.759879642	1.464605352
P61079	Ubiquitin-conjugating enzyme E2 D3 OS=Mus musculus OX=10090 GN=Ube2d3 PE=1 SV=1 - [UB2D3_MOUSE]	Ube2d3	197.1034509	0	16.4409045	247.4959471	304.1145895	48.66453777	71.18145179	200.0916915	109.3610453	134.1605561	0.2	0.726530612	2.811008857	1.491087999
Q9Z0H4	CUGBP Elav-like family member 2 OS=Mus musculus OX=10090 GN=Celf2 PE=1 SV=1 - [CELF2_MOUSE]	Celf2	60.91821967	11.78680177	0	82.08115661	99.54211151	31.73304513	24.23500715	71.11877108	32.31061334	35.20863237	0.2	0.726530612	2.934547147	1.553137887
Q9D1A2	Cytosolic non-specific dipeptidase OS=Mus musculus OX=10090 GN=Cndp2 PE=1 SV=1 - [CNDP2_MOUSE]	Cndp2	163.8946617	38.1773482	0	252.5877381	247.3898294	101.3551251	67.35733662	200.4442309	85.75528452	85.85302978	0.2	0.726530612	2.97583368	1.573293896
P61161	Actin-related protein 2 OS=Mus musculus OX=10090 GN=Actr2 PE=1 SV=1 - [ARP2_MOUSE]	Actr2	455.2640369	188.6934702	138.6640172	1061.268328	1118.14866	262.236338	260.8738415	813.8844422	170.195175	478.5870519	0.2	0.726530612	3.119839221	1.641471682
Q9EQU5	Protein SET OS=Mus musculus OX=10090 GN=Set PE=1 SV=1 - [SET_MOUSE]	Set	148.1247787	41.04950032	0	188.7959019	310.8334346	101.2510982	63.05809299	200.2934783	76.4756244	105.2631684	0.2	0.726530612	3.176332628	1.667362001
Q9EPL8	Importin-7 OS=Mus musculus OX=10090 GN=Ipo7 PE=1 SV=2 - [IPO7_MOUSE]	Ipo7	44.64632957	0	10.63116104	60.86887934	86.19351928	31.33812289	18.42583021	59.4668405	23.32147801	27.45456088	0.2	0.726530612	3.227362884	1.690355803
Q9CQM5	Thioredoxin domain-containing protein 17 OS=Mus musculus OX=10090 GN=Txndc17 PE=1 SV=1 - [TXD17_MOUSE]	Txndc17	94.88329586	46.04020512	0	212.3380095	239.4705698	73.10277902	46.97450032	174.9704528	47.44854728	89.2569951	0.2	0.726530612	3.724796465	1.897161594
Q8CI94	Glycogen phosphorylase, brain form OS=Mus musculus OX=10090 GN=Pygb PE=1 SV=3 - [PYGB_MOUSE]	Pygb	62.13499909	0	19.9264436	70.21008793	216.1156048	36.65450072	27.35381423	107.6600645	31.72639128	95.41198839	0.2	0.726530612	3.935833722	1.976669272
Q8CGC7	Bifunctional glutamate/proline--tRNA ligase OS=Mus musculus OX=10090 GN=Eprs1 PE=1 SV=4 - [SYEP_MOUSE]	Eprs1	114.8054333	25.09426958	23.88290676	141.8659792	642.0447236	42.04905703	54.5942032	275.31992	52.14797234	321.4905375	0.2	0.726530612	5.043024786	2.334289316
Q8BI66	Zinc finger protein 526 OS=Mus musculus OX=10090 GN=Znf526 PE=2 SV=1 - [ZN526_MOUSE]	Znf526	131.2059192	159.2617824	0	30.67506792	0	0	96.8225672	10.22502264	85.01611615	17.71025872	0.353678517	0.764125594	0.105605779	-3.243239309
Q3UV17	Keratin, type II cytoskeletal 2 oral OS=Mus musculus OX=10090 GN=Krt76 PE=1 SV=1 - [K22O_MOUSE]	Krt76	216.5298852	568.1465888	0	0	160.6994763	0	261.5588247	53.56649209	286.7374071	92.77988588	0.353678517	0.764125594	0.204797113	-2.287732719
Q5FWI3	Cell surface hyaluronidase OS=Mus musculus OX=10090 GN=Cemip2 PE=1 SV=1 - [CEIP2_MOUSE]	Cemip2	5.23673474	3.429346556	0	0	2.049138942	0	2.888693765	0.683046314	2.659901645	1.18307092	0.353678517	0.764125594	0.236455079	-2.080361961
P97450	ATP synthase-coupling factor 6, mitochondrial OS=Mus musculus OX=10090 GN=Atp5pf PE=1 SV=1 - [ATP5J_MOUSE]	Atp5pf	0	128.8108178	54.29934812	0	0	46.69794581	61.03672197	15.56598194	64.66916459	26.96107158	0.353678517	0.764125594	0.255026506	-1.971280895
Q6IFZ9	Keratin, type II cytoskeletal 74 OS=Mus musculus OX=10090 GN=Krt74 PE=3 SV=1 - [K2C74_MOUSE]	Krt74	150.4660093	0	140.8630888	77.04731206	0	0	97.10969935	25.68243735	84.23641909	44.48328636	0.353678517	0.764125594	0.264468303	-1.918833274
Q8R0F3	Formylglycine-generating enzyme OS=Mus musculus OX=10090 GN=Sumf1 PE=1 SV=2 - [SUMF1_MOUSE]	Sumf1	0	85.38215157	108.242788	0	0	54.27759518	64.54164651	18.09253173	57.05147039	31.33718419	0.353678517	0.764125594	0.280323368	-1.834836081
Q8BLX7	Collagen alpha-1(XVI) chain OS=Mus musculus OX=10090 GN=Col16a1 PE=1 SV=2 - [COGA1_MOUSE]	Col16a1	11.77096296	0	13.90192246	0	7.679791562	0	8.557628474	2.559930521	7.487322681	4.433929726	0.353678517	0.764125594	0.29914018	-1.741106392
Q9Z0S9	Prenylated Rab acceptor protein 1 OS=Mus musculus OX=10090 GN=Rabac1 PE=1 SV=1 - [PRAF1_MOUSE]	Rabac1	34.85072527	0	30.2297831	0	20.73903655	0	21.69350279	6.913012182	18.92866409	11.97368833	0.353678517	0.764125594	0.318667402	-1.649876647
Q9CQ40	39S ribosomal protein L49, mitochondrial OS=Mus musculus OX=10090 GN=Mrpl49 PE=1 SV=1 - [RM49_MOUSE]	Mrpl49	0	12.08774762	13.9867679	0	0	8.321976176	8.691505175	2.773992059	7.586716428	4.804695186	0.353678517	0.764125594	0.319161296	-1.647642384
Q3TMX7	Sulfhydryl oxidase 2 OS=Mus musculus OX=10090 GN=Qsox2 PE=1 SV=1 - [QSOX2_MOUSE]	Qsox2	25.08399654	23.41778632	0	0	15.84239061	0	16.16726096	5.280796871	14.02602257	9.146608486	0.353678517	0.764125594	0.326635222	-1.614247726
Q9QZS0	Collagen alpha-3(IV) chain OS=Mus musculus OX=10090 GN=Col4a3 PE=1 SV=2 - [CO4A3_MOUSE]	Col4a3	91.98443959	122.3143788	0	0	0	72.11992135	71.43293948	24.03997378	63.694387	41.638456	0.353678517	0.764125594	0.336539053	-1.571154167
Q8C9B9	Death-inducer obliterator 1 OS=Mus musculus OX=10090 GN=Dido1 PE=1 SV=4 - [DIDO1_MOUSE]	Dido1	8.395680293	0	0	8.886043477	8.716600325	0	2.798560098	5.867547934	4.847248277	5.08215179	0.353678517	0.764125594	2.096631028	1.068072993
Q9CZN7	Serine hydroxymethyltransferase, mitochondrial OS=Mus musculus OX=10090 GN=Shmt2 PE=1 SV=1 - [GLYM_MOUSE]	Shmt2	22.26929927	0	0	22.92313867	23.78628064	0	7.423099757	15.56980643	12.85718593	13.49075268	0.353678517	0.764125594	2.097480425	1.068657347
Q99JW4	LIM and senescent cell antigen-like-containing domain protein 1 OS=Mus musculus OX=10090 GN=Lims1 PE=1 SV=3 - [LIMS1_MOUSE]	Lims1	29.52512431	0	0	31.94414351	30.43880254	0	9.841708103	20.79431535	17.04633847	18.02412761	0.353678517	0.764125594	2.112876661	1.079208552
Q91W89	Alpha-mannosidase 2C1 OS=Mus musculus OX=10090 GN=Man2c1 PE=1 SV=1 - [MA2C1_MOUSE]	Man2c1	35.45408075	0	0	38.88469359	37.98988291	0	11.81802692	25.62485883	20.46942307	22.1962883	0.353678517	0.764125594	2.168285705	1.116554867
Q61160	FAS-associated death domain protein OS=Mus musculus OX=10090 GN=Fadd PE=1 SV=1 - [FADD_MOUSE]	Fadd	14.00350449	0	0	16.30476933	14.06061342	0	4.66783483	10.12179425	8.084927087	8.837256252	0.353678517	0.764125594	2.168413112	1.116639636
P13439	Uridine 5'-monophosphate synthase OS=Mus musculus OX=10090 GN=Umps PE=1 SV=3 - [UMPS_MOUSE]	Umps	33.43719563	0	0	33.61804439	39.19261729	0	11.14573188	24.27022056	19.3049739	21.20263357	0.353678517	0.764125594	2.177534937	1.122695866
Q922D4	Serine/threonine-protein phosphatase 6 regulatory subunit 3 OS=Mus musculus OX=10090 GN=Ppp6r3 PE=1 SV=1 - [PP6R3_MOUSE]	Ppp6r3	19.55477178	0	0	19.63241328	22.99240388	0	6.518257259	14.20827239	11.28995275	12.41888229	0.353678517	0.764125594	2.179765514	1.124172947
Q9D0R8	Protein LSM12 homolog OS=Mus musculus OX=10090 GN=Lsm12 PE=1 SV=1 - [LSM12_MOUSE]	Lsm12	33.96431844	0	0	34.07046763	40.04514669	0	11.32143948	24.70520477	19.6093084	21.60288302	0.353678517	0.764125594	2.182161095	1.12575761
Q6IQX8	Zinc finger protein 219 OS=Mus musculus OX=10090 GN=Znf219 PE=1 SV=1 - [ZN219_MOUSE]	Znf219	14.58767992	0	0	16.73123123	15.5174702	0	4.862559972	10.74956714	8.422200926	9.329158552	0.353678517	0.764125594	2.21068063	1.144490619
Q60676	Serine/threonine-protein phosphatase 5 OS=Mus musculus OX=10090 GN=Ppp5c PE=1 SV=3 - [PPP5_MOUSE]	Ppp5c	59.06944391	0	0	62.18385515	68.47898647	0	19.68981464	43.55428054	34.10375934	37.8502138	0.353678517	0.764125594	2.212020852	1.145364985
Q8VCN9	Tubulin-specific chaperone C OS=Mus musculus OX=10090 GN=Tbcc PE=1 SV=1 - [TBCC_MOUSE]	Tbcc	13.55785214	0	0	15.66372078	14.41904514	0	4.519284046	10.02758864	7.827629581	8.706417449	0.353678517	0.764125594	2.21884452	1.149808578
Q8R0H9	ADP-ribosylation factor-binding protein GGA1 OS=Mus musculus OX=10090 GN=Gga1 PE=1 SV=1 - [GGA1_MOUSE]	Gga1	21.61388408	0	0	23.77315285	24.25424803	0	7.204628028	16.00913363	12.47878179	13.86640302	0.353678517	0.764125594	2.22206248	1.151899383
Q8K2V6	Importin-11 OS=Mus musculus OX=10090 GN=Ipo11 PE=1 SV=1 - [IPO11_MOUSE]	Ipo11	26.2330994	0	0	30.63481061	27.97290911	0	8.744366466	19.53590657	15.145687	16.97086221	0.353678517	0.764125594	2.234113432	1.159702437
Q9WU84	Copper chaperone for superoxide dismutase OS=Mus musculus OX=10090 GN=Ccs PE=1 SV=1 - [CCS_MOUSE]	Ccs	26.65655494	0	0	30.28813205	29.30735657	0	8.885518314	19.86516287	15.39016917	17.21072346	0.353678517	0.764125594	2.23567857	1.160712782
P13864	DNA (cytosine-5)-methyltransferase 1 OS=Mus musculus OX=10090 GN=Dnmt1 PE=1 SV=5 - [DNMT1_MOUSE]	Dnmt1	45.98156558	0	0	52.51522052	50.80760392	0	15.32718853	34.44094148	26.54746926	29.83894814	0.353678517	0.764125594	2.247048859	1.168031494
Q8VDQ1	Prostaglandin reductase 2 OS=Mus musculus OX=10090 GN=Ptgr2 PE=1 SV=2 - [PTGR2_MOUSE]	Ptgr2	25.52176715	0	0	30.17387966	27.17899399	0	8.507255715	19.11762455	14.73499913	16.62392888	0.353678517	0.764125594	2.247214047	1.168137548

Q6NVF9	Cleavage and polyadenylation specificity factor subunit 6 OS=Mus musculus OX=10090 GN=Cpsf6 PE=1 SV=1 - [CPSF6_MOUSE]	Cpsf6	41.27974439	0	0	47.82320231	45.99274516	0	13.7599148	31.27198249	23.83287154	27.09779161	0.353678517	0.764125594	2.272687219	1.184399145
Q91VR5	ATP-dependent RNA helicase DDX1 OS=Mus musculus OX=10090 GN=Ddx1 PE=1 SV=1 - [DDX1_MOUSE]	Ddx1	34.88446156	0	0	39.10380549	40.84840256	0	11.62815385	26.65073602	20.14055327	23.09669246	0.353678517	0.764125594	2.291914637	1.196553311
Q8VBV3	Exosome complex component RRP4 OS=Mus musculus OX=10090 GN=Exosc2 PE=1 SV=1 - [EXOS2_MOUSE]	Exosc2	12.9337705	0	0	16.98995899	13.2184201	0	4.311256835	10.0694597	7.467315882	8.921975135	0.353678517	0.764125594	2.335620465	1.223805858
Q9WTX6	Cullin-1 OS=Mus musculus OX=10090 GN=Cul1 PE=1 SV=1 - [CUL1_MOUSE]	Cul1	22.4134224	0	0	24.18985407	28.36464662	0	7.471140799	17.5181669	12.94039545	15.3141063	0.353678517	0.764125594	2.344778042	1.229451363
Q6PAM1	Alpha-taxilin OS=Mus musculus OX=10090 GN=Txlna PE=1 SV=1 - [TXLNA_MOUSE]	Txlna	13.87933131	0	0	16.08187176	16.89206865	0	4.626443769	10.99131347	8.013235666	9.527372861	0.353678517	0.764125594	2.375758578	1.248388239
P0C7N9	Proteasome assembly chaperone 4 OS=Mus musculus OX=10090 GN=Psmg4 PE=1 SV=1 - [PSMG4_MOUSE]	Psmg4	36.77345218	0	0	46.76560645	40.65533716	0	12.25781739	29.14031454	21.23116251	25.42050936	0.353678517	0.764125594	2.377284112	1.249314332
Q9D662	Protein transport protein Sec23B OS=Mus musculus OX=10090 GN=Sec23b PE=1 SV=1 - [SC23B_MOUSE]	Sec23b	133.4503005	0	0	154.5749499	164.7300283	0	44.48343351	106.4349927	77.04756694	92.31515132	0.353678517	0.764125594	2.392688341	1.258632491
Q9Z0R4	Intersectin-1 OS=Mus musculus OX=10090 GN=Itsn1 PE=1 SV=2 - [ITSN1_MOUSE]	Itsn1	37.64909417	0	0	45.70554425	44.39471832	0	12.54969806	30.03342086	21.73671466	26.01796192	0.353678517	0.764125594	2.393158841	1.258916156
Q9CYT6	Adenylyl cyclase-associated protein 2 OS=Mus musculus OX=10090 GN=Cap2 PE=1 SV=1 - [CAP2_MOUSE]	Cap2	157.0318674	0	0	168.2568708	212.4008185	0	52.34395579	126.8858964	90.6623909	112.0811985	0.353678517	0.764125594	2.424079237	1.277436858
Q61881	DNA replication licensing factor MCM7 OS=Mus musculus OX=10090 GN=Mcm7 PE=1 SV=1 - [MCM7_MOUSE]	Mcm7	9.200655521	0	0	12.8020762	9.601131345	0	3.066885174	7.467735848	5.312000942	6.662343508	0.353678517	0.764125594	2.434957759	1.283896745
O08997	Copper transport protein ATOX1 OS=Mus musculus OX=10090 GN=Atox1 PE=1 SV=1 - [ATOX1_MOUSE]	Atox1	95.90385459	0	0	108.2967706	125.823234	0	31.96795153	78.04000151	55.37011626	68.15038963	0.353678517	0.764125594	2.44119494	1.287587505
Q8R010	Aminoacyl tRNA synthase complex-interacting multifunctional protein 2 OS=Mus musculus OX=10090 GN=Aimp2 PE=1 SV=2 - [AIMP2_MOUSE]	Aimp2	23.84957506	0	0	31.48159669	26.88180035	0	7.949858354	19.45446568	13.76955858	17.00431439	0.353678517	0.764125594	2.447146202	1.291100296
Q9WUM3	Coronin-1B OS=Mus musculus OX=10090 GN=Coro1b PE=1 SV=1 - [COR1B_MOUSE]	Coro1b	109.6645274	0	0	118.3985678	153.3428293	0	36.55484247	90.58046568	63.31484442	80.36722545	0.353678517	0.764125594	2.477933416	1.309137422
Q9DCS2	Methyltransferase-like 26 OS=Mus musculus OX=10090 GN=Mettl26 PE=1 SV=1 - [MTL26_MOUSE]	Mettl26	11.9015313	0	0	17.27080222	12.22681918	0	3.967177101	9.832540465	6.871352301	8.880854702	0.353678517	0.764125594	2.478472782	1.309451415
Q3UE37	Ubiquitin-conjugating enzyme E2 Z OS=Mus musculus OX=10090 GN=Ube2z PE=1 SV=2 - [UBE2Z_MOUSE]	Ube2z	16.10090558	0	0	21.54410348	18.67555987	0	5.366968525	13.40655445	9.295862168	11.69867139	0.353678517	0.764125594	2.497975233	1.320759173
Q9D554	Splicing factor 3A subunit 3 OS=Mus musculus OX=10090 GN=Sf3a3 PE=1 SV=2 - [SF3A3_MOUSE]	Sf3a3	29.44625604	0	0	31.67163881	42.75581602	0	9.81541868	24.80915161	17.00080385	22.18862882	0.353678517	0.764125594	2.52756937	1.337750688
O35604	NPC intracellular cholesterol transporter 1 OS=Mus musculus OX=10090 GN=Npc1 PE=1 SV=2 - [NPC1_MOUSE]	Npc1	57.19135707	0	0	76.74758004	68.0841169	0	19.06378569	48.27723231	33.0194454	42.03310919	0.353678517	0.764125594	2.532405321	1.340508332
P08556	GTPase NRas OS=Mus musculus OX=10090 GN=Nras PE=1 SV=1 - [RASN_MOUSE]	Nras	61.3205456	0	0	0	61.95149739	94.17814405	20.44018187	52.04321381	35.40343351	47.86450947	0.353678517	0.764125594	2.546122835	1.348302022
P49443	Protein phosphatase 1A OS=Mus musculus OX=10090 GN=Ppm1a PE=1 SV=1 - [PPM1A_MOUSE]	Ppm1a	24.66672013	0	0	27.91368315	35.90251123	0	8.222240042	21.27206479	14.24133751	18.85022278	0.353678517	0.764125594	2.587137408	1.371356681
O54774	AP-3 complex subunit delta-1 OS=Mus musculus OX=10090 GN=Ap3d1 PE=1 SV=1 - [AP3D1_MOUSE]	Ap3d1	30.63592598	0	0	38.7920485	40.65912886	0	10.21197533	26.48372579	17.68766011	22.95457026	0.353678517	0.764125594	2.59339892	1.37484414
Q8BZA9	Fructose-2,6-bisphosphatase TIGAR OS=Mus musculus OX=10090 GN=Tigar PE=1 SV=1 - [TIGAR_MOUSE]	Tigar	14.26446652	0	0	16.85623981	20.22837021	0	4.754822173	12.36153667	8.235593585	10.83736626	0.353678517	0.764125594	2.599789482	1.378394806
Q9D2G2	Dihydrolipoyllysine-residue succinyltransferase component of 2-oxoglutarate dehydrogenase complex, mitochondrial OS=Mus musculus OX=10090 GN=Dl:	Dlst	37.84252275	0	0	58.06295583	42.91327506	0	12.61417425	33.65874363	21.84839069	30.11746128	0.353678517	0.764125594	2.668327151	1.415935559
P61358	60S ribosomal protein L27 OS=Mus musculus OX=10090 GN=Rpl27 PE=1 SV=2 - [RL27_MOUSE]	Rpl27	72.14461963	0	0	87.99783387	105.4472449	0	24.04820654	64.48169292	41.65271556	56.52023556	0.353678517	0.764125594	2.681351427	1.422960316
Q8K3C3	Protein LZIC OS=Mus musculus OX=10090 GN=Lzic PE=1 SV=1 - [LZIC_MOUSE]	Lzic	19.70047917	0	0	33.30490498	19.95540461	0	6.566826391	17.75343653	11.37407695	16.7612849	0.353678517	0.764125594	2.703503256	1.434830094
Q9D967	Magnesium-dependent phosphatase 1 OS=Mus musculus OX=10090 GN=Mdp1 PE=1 SV=1 - [MGDP1_MOUSE]	Mdp1	40.88847606	0	0	63.75463562	48.4688867	0	13.62949202	37.40784078	23.60697266	33.28548398	0.353678517	0.764125594	2.744624724	1.456608901
Q60854	Serpin B6 OS=Mus musculus OX=10090 GN=Serpinb6 PE=1 SV=1 - [SPB6_MOUSE]	Serpinb6	55.63968132	0	0	70.61867724	82.74215903	0	18.54656044	51.12027876	32.12358499	44.68452616	0.353678517	0.764125594	2.756321255	1.462744047
P97390	Vacuolar protein sorting-associated protein 45 OS=Mus musculus OX=10090 GN=Vps45 PE=1 SV=1 - [VPS45_MOUSE]	Vps45	12.7679631	0	0	19.87113399	15.49902302	0	4.2559877	11.79005234	7.371586933	10.4418791	0.353678517	0.764125594	2.770227071	1.470004236
Q9Z1X4	Interleukin enhancer-binding factor 3 OS=Mus musculus OX=10090 GN=Ilf3 PE=1 SV=2 - [ILF3_MOUSE]	Ilf3	69.60402331	0	0	96.64469477	96.44119905	0	23.2013411	64.36196461	40.18590159	55.73918925	0.353678517	0.764125594	2.774062254	1.472000164
Q9CZ42	ATP-dependent (S)-NAD(P)H-hydrate dehydratase OS=Mus musculus OX=10090 GN=Naxd PE=1 SV=1 - [NNRD_MOUSE]	Naxd	12.93112421	0	0	19.08793771	16.96150012	0	4.310374736	12.01647928	7.465788043	10.46074877	0.353678517	0.764125594	2.787803848	1.479129056
P24288	Branched-chain-amino-acid aminotransferase, cytosolic OS=Mus musculus OX=10090 GN=Bcat1 PE=1 SV=2 - [BCAT1_MOUSE]	Bcat1	39.76726697	0	0	57.40842662	53.91917279	0	13.25575566	37.10919981	22.95964229	32.18482959	0.353678517	0.764125594	2.799478262	1.485157978
Q99K28	ADP-ribosylation factor GTPase-activating protein 2 OS=Mus musculus OX=10090 GN=Arfgap2 PE=1 SV=1 - [ARFG2_MOUSE]	Arfgap2	14.94648088	0	0	17.47909837	24.48252449	0	4.982160293	13.98720762	8.629354759	12.60926156	0.353678517	0.764125594	2.807458371	1.48926463
Q8VBT9	Tether containing UBX domain for GLUT4 OS=Mus musculus OX=10090 GN=Aspscr1 PE=1 SV=1 - [ASPC1_MOUSE]	Aspscr1	10.55142346	0	0	13.30692159	16.39135155	0	3.517141154	9.899424382	6.091867177	8.710762273	0.353678517	0.764125594	2.81462243	1.492941404
Q8VD62	UPF0696 protein C11orf68 homolog OS=Mus musculus OX=10090 GN=Bles03 PE=1 SV=2 - [CK068_MOUSE]	Bles03	15.83026311	0	0	23.49940774	21.46603633	0	5.27675437	14.98848135	9.139606667	13.02016051	0.353678517	0.764125594	2.840473576	1.506131482
Q4KMM3	Oxidation resistance protein 1 OS=Mus musculus OX=10090 GN=Oxr1 PE=1 SV=3 - [OXR1_MOUSE]	Oxr1	21.38311566	0	0	38.9158076	22.20047464	0	7.127705221	20.37209408	12.34554758	19.52222456	0.353678517	0.764125594	2.858156089	1.515084707
Q9CZ04	COP9 signalosome complex subunit 7a OS=Mus musculus OX=10090 GN=Cops7a PE=1 SV=2 - [CSN7A_MOUSE]	Cops7a	17.16290838	0	0	23.63722056	25.42846949	0	5.72096946	16.35523002	9.909009774	14.19233261	0.353678517	0.764125594	2.858821417	1.515420501
P60229	Eukaryotic translation initiation factor 3 subunit E OS=Mus musculus OX=10090 GN=Eif3e PE=1 SV=1 - [EIF3E_MOUSE]	Eif3e	23.48230692	0	0	31.64847133	35.72810616	0	7.82743564	22.45885916	13.55751622	19.55661324	0.353678517	0.764125594	2.869248653	1.520672999
Q9JLV6	Bifunctional polynucleotide phosphatase/kinase OS=Mus musculus OX=10090 GN=Pnkp PE=1 SV=2 - [PNKP_MOUSE]	Pnkp	18.37153126	0	0	26.40235398	26.55014939	0	6.123843753	17.65083446	10.60680852	15.28624966	0.353678517	0.764125594	2.882313	1.527227011
Q9QXE7	F-box-like/WD repeat-containing protein TBL1X OS=Mus musculus OX=10090 GN=Tbl1x PE=1 SV=2 - [TBL1X_MOUSE]	Tbl1x	71.37909243	0	0	102.6925017	103.2096518	0	23.79303081	68.63405115	41.21073823	59.43939429	0.353678517	0.764125594	2.884628347	1.528385455
Q9ESZ8	General transcription factor II-I OS=Mus musculus OX=10090 GN=Gtf2i PE=1 SV=3 - [GTF2I_MOUSE]	Gtf2i	34.23767314	0	0	44.95699844	53.83717013	0	11.41255771	32.93138952	19.7671298	28.86298104	0.353678517	0.764125594	2.885539802	1.528841231
Q8R0F6	Integrin-linked kinase-associated serine/threonine phosphatase 2C OS=Mus musculus OX=10090 GN=Ilkap PE=1 SV=1 - [ILKAP_MOUSE]	Ilkap	6.612902219	0	0	11.01470294	8.169264282	0	2.20430074	6.394655741	3.817960876	5.717765895	0.353678517	0.764125594	2.900990607	1.536545624
Q80U93	Nuclear pore complex protein Nup214 OS=Mus musculus OX=10090 GN=Nup214 PE=1 SV=2 - [NU214_MOUSE]	Nup214	4.826834314	0	0	7.707038201	6.401212169	0	1.608944771	4.702750123	2.78677409	4.124704771	0.353678517	0.764125594	2.922878527	1.547389873
Q9CRT8	Exportin-T OS=Mus musculus OX=10090 GN=Xpot PE=1 SV=3 - [XPOT_MOUSE]	Xpot	12.33866015	0	0	14.30653542	21.83846639	0	4.112886715	12.04833394	7.123728757	11.09298242	0.353678517	0.764125594	2.929410599	1.550610422
O55060	Thiopurine S-methyltransferase OS=Mus musculus OX=10090 GN=Tpmt PE=1 SV=1 - [TPMT_MOUSE]	Tpmt	34.17116068	0	0	58.90536423	42.07065541	0	11.39038689	33.65867321	19.72872882	30.34026313	0.353678517	0.764125594	2.955007018	1.563161557
Q91ZU6	Dystonin OS=Mus musculus OX=10090 GN=Dst PE=1 SV=2 - [DYST_MOUSE]	Dst	9.370209207	0	0	12.96172339	14.77124278	0	3.123403069	9.244322056	5.409892808	8.056780239	0.353678517	0.764125594	2.959695515	1.565448763
Q80VD1	Protein FAM98B OS=Mus musculus OX=10090 GN=Fam98b PE=1 SV=1 - [FA98B_MOUSE]	Fam98b	40.81385412	0	0	49.18572584	71.76242767	0	13.60461804	40.31605117	23.56388966	36.69420735	0.353678517	0.764125594	2.963409267	1.567257886
Q8BVG4	Dipeptidyl peptidase 9 OS=Mus musculus OX=10090 GN=Dpp9 PE=1 SV=2 - [DPP9_MOUSE]	Dpp9	18.08272664	0	0	27.04573909	27.29346079	0	6.027575545	18.11306663	10.44006709	15.68686484	0.353678517	0.764125594	3.005033531	1.587381089
Q80Y81	Zinc phosphodiesterase ELAC protein 2 OS=Mus musculus OX=10090 GN=Elac2 PE=1 SV=1 - [RNZ2_MOUSE]	Elac2	20.00030104	0	0	30.47307862	29.93535664	0	6.666767012	20.13614509	11.54717919	17.44048567	0.353678517	0.764125594	3.020376301	1.594728302
Q58A65	C-Jun-amino-terminal kinase-interacting protein 4 OS=Mus musculus OX=10090 GN=Spag9 PE=1 SV=2 - [JIP4_MOUSE]	Spag9	24.00549413	0	0	37.92055544	35.78604486	0	8.001831376	24.56886677	13.8595785	21.3040124	0.353678517	0.764125594	3.070405462	1.618429183
Q6PGH1	Protein BUD31 homolog OS=Mus musculus OX=10090 GN=Bud31 PE=1 SV=2 - [BUD31_MOUSE]	Bud31	18.51784911	0	0	18.8607052	38.04215928	0	6.172616369	18.96762149	10.69128517	19.021305	0.353678517	0.764125594	3.07286576	1.619584744
Q8VHK9	ATP-dependent DNA/RNA helicase DHX36 OS=Mus musculus OX=10090 GN=Dhx36 PE=1 SV=2 - [DHX36_MOUSE]	Dhx36	13.34254655	0	0	20.62116277	20.51120481	0	4.447515517	13.71078919	7.703322843	11.87401903	0.353678517	0.764125594	3.082797382	1.624240071
Q9JIX0	Transcription and mRNA export factor ENY2 OS=Mus musculus OX=10090 GN=Eny2 PE=1 SV=1 - [ENY2_MOUSE]	Eny2	39.54157794	0	0	77.02454296	45.2186299	0	13.18052598	40.74772429	22.82934067	38.7064181	0.353678517	0.764125594	3.091509728	1.628311545
Q60996	Serine/threonine-protein phosphatase 2A 56 kDa regulatory subunit gamma isoform OS=Mus musculus OX=10090 GN=Ppp2r5c PE=1 SV=2 - [2A5G_MOUS	Ppp2r5c	21.80189737	0	0	23.47368666	44.28346041	0	7.267299124	22.58571569	12.58733132	22.15508036	0.353678517	0.764125594	3.107855519	1.635919436
Q78ZA7	Nucleosome assembly protein 1-like 4 OS=Mus musculus OX=10090 GN=Nap1l4 PE=1 SV=1 - [NP1L4_MOUSE]	Nap1l4	43.70130419	0	0	59.17955428	76.97631856	0	14.5671014	45.38529095	25.23095974	40.29949976	0.353678517	0.764125594	3.115602049	1.639510972
Q99KG3	RNA-binding protein 10 OS=Mus musculus OX=10090 GN=Rbm10 PE=1 SV=1 - [RBM10_MOUSE]	Rbm10	12.71239645	0	0	15.30125499	24.45943968	0	4.237465485	13.25356489	7.339505515	12.35762208	0.353678517	0.764125594	3.127710405	1.64510694
E9Q5C9	Nucleolar and coiled-body phosphoprotein 1 OS=Mus musculus OX=10090 GN=Nolc1 PE=1 SV=1 - [NOLC1_MOUSE]	Nolc1	16.89214915	0	0	20.23244989	32.7025324	0	5.630716382	17.64499409	9.752686856	16.50409349	0.353678517	0.764125594	3.133703227	1.647868558
Q8CEC6	Peptidylprolyl isomerase domain and WD repeat-containing protein 1 OS=Mus musculus OX=10090 GN=Ppwd1 PE=1 SV=2 - [PPWD1_MOUSE]	Ppwd1	20.82971123	0	0	21.4971467	44.26152051	0	6.943237077	21.91955574	12.02603939	22.13378349	0.353678517	0.764125594	3.156964899	1.65853822
Q9CZU3	Exosome RNA helicase MTR4 OS=Mus musculus OX=10090 GN=Mtrex PE=1 SV=1 - [MTREX_MOUSE]	Mtrex	13.27616185	0	0	17.60794889	24.41619785	0	4.425387284	14.00804891	7.66499562	12.59988648	0.353678517	0.764125594	3.165383731	1.662380404
Q6ZQ08	CCR4-NOT transcription complex subunit 1 OS=Mus musculus OX=10090 GN=Cnot1 PE=1 SV=2 - [CNOT1_MOUSE]	Cnot1	16.36905176	0	0	27.80879143	24.04118501	0	5.456350587	17.28332548	9.45067644	15.08587814	0.353678517	0.764125594	3.167561396	1.663372583
Q3TW96	UDP-N-acetylhexosamine pyrophosphorylase-like protein 1 OS=Mus musculus OX=10090 GN=Uap1l1 PE=1 SV=1 - [UAP1L_MOUSE]	Uap1l1	10.2196678	0	0	15.24754258	17.23735324	0	3.406555933	10.8282986	5.900327955	9.43021074	0.353678517	0.764125594	3.178664557	1.668420777
Q03265	ATP synthase subunit alpha, mitochondrial OS=Mus musculus OX=10090 GN=Atp5f1a PE=1 SV=1 - [ATPA_MOUSE]	Atp5f1a	82.85104599	0	0	168.4246213	102.1603143	0	27.61701533	90.19497852	47.8340737	84.84745258	0.353678517	0.764125594	3.265920573	1.707489705
Q8VBV7	COP9 signalosome complex subunit 8 OS=Mus musculus OX=10090 GN=Cops8 PE=1 SV=1 - [CSN8_MOUSE]	Cops8	43.04157188	0	0	85.50922053	61.51277807	0	14.34719063	49.00733287	24.85006311	44.10494665	0.353678517	0.764125594	3.415813879	1.772229367
Q9ERG0	LIM domain and actin-binding protein 1 OS=Mus musculus OX=10090 GN=Lima1 PE=1 SV=3 - [LIMA1_MOUSE]	Lima1	30.75048555	0	0	62.96184392	42.30309389	0	10.25016185	35.08831261	17.75380111	32.09498792	0.353678517	0.764125594	3.423195957	1.77534388
Q08024	Core-binding factor subunit beta OS=Mus musculus OX=10090 GN=Cbfb PE=1 SV=1 - [PEBB_MOUSE]	Cbfb	22.96995619	0	0	43.33147515	36.85318361	0	7.656652063	26.72821958	13.26171039	23.37285513	0.353678517	0.764125594	3.490849443	1.803578136
Q9Z2Y8	Pyridoxal phosphate homeostasis protein OS=Mus musculus OX=10090 GN=Plpbp PE=1 SV=1 - [PLPHP_MOUSE]	Plpbp	32.31997944	0	0	53.60352902	62.11000997	0	10.77332648	38.57117966	18.65994883	33.67331258	0.353678517	0.764125594	3.580247915	1.840059491
Q8R164	Valacyclovir hydrolase OS=Mus musculus OX=10090 GN=Bphl PE=1 SV=1 - [BPHL_MOUSE]	Bphl	30.3965587	0	0	46.1106169	63.18148813	0	10.13218623	36.43070168	17.54946135	32.68410439	0.353678517	0.764125594	3.595542052	1.846209283
Q80X41	Serine/threonine-protein kinase VRK1 OS=Mus musculus OX=10090 GN=Vrk1 PE=1 SV=2 - [VRK1_MOUSE]	Vrk1	34.1499558	0	0	63.68504409	61.51357428	0	11.3833186	41.73287279	19.71648617	36.15803263	0.353678517	0.764125594	3.666142911	1.874263025
Q62448	Eukaryotic translation initiation factor 4 gamma 2 OS=Mus musculus OX=10090 GN=Eif4g2 PE=1 SV=2 - [IF4G2_MOUSE]	Eif4g2	14.31925052	0	0	22.82237776	30.34392114	0	4.773083507	17.72209963	8.267223143	15.80183582	0.353678517	0.764125594	3.712924697	1.892556056
B2RY56	RNA-binding protein 25 OS=Mus musculus OX=10090 GN=Rbm25 PE=1 SV=2 - [RBM25_MOUSE]	Rbm25	6.547287733	0	0	12.70873496	12.05780335	0	2.182429244	8.255512771	3.780078335	7.156888033	0.353678517	0.764125594	3.782717259	1.919422946
Q9QYR6	Microtubule-associated protein 1A OS=Mus musculus OX=10090 GN=Map1a PE=1 SV=2 - [MAP1A_MOUSE]	Map1a	19.89847638	0	0	39.2163992	37.67762532	0	6.632825459	25.63134151	11.48839069	22.21072279	0.353678517	0.764125594	3.8643172	1.950213522
P97467	Peptidyl-glycine alpha-amidating monooxygenase OS=Mus musculus OX=10090 GN=Pam PE=1 SV=2 - [AMD_MOUSE]	Pam	0	23.58367485	0	0	48.72626529	43.28720768	7.86122495	30.67115766	13.61604102	26.70085712	0.353678517	0.764125594	3.901574863	1.964056583
Q64105	Sepiapterin reductase OS=Mus musculus OX=10090 GN=Spr PE=1 SV=1 - [SPRE_MOUSE]	Spr	32.14809687	0	0	77.22769198	54.9437125	0	10.71603229	44.05713483	18.56071238	39.74817332	0.353678517	0.764125594	4.111329047	2.039604842
Q9D0M5	Dynein light chain 2, cytoplasmic OS=Mus musculus OX=10090 GN=Dynll2 PE=1 SV=1 - [DYL2_MOUSE]	Dynll2	0	0	126.6967488	232.7831304	291.3406697	0	42.23224959	174.7079334	73.148402	154.1083802	0.353678517	0.764125594	4.136837017	2.048528119
Q80W21	Glutathione S-transferase Mu 7 OS=Mus musculus OX=10090 GN=Gstm7 PE=1 SV=1 - [GSTM7_MOUSE]	Gstm7	1445.969256	0	0	3057.287907	3045.245691	0	481.989752	2034.177866	834.8307392	1761.659998	0.353678517	0.764125594	4.22037576	2.077371455
Q99J77	Sialic acid synthase OS=Mus musculus OX=10090 GN=Nans PE=1 SV=1 - [SIAS_MOUSE]	Nans	16.00208359	0	0	34.01569062	35.44901262	0	5.334027863	23.15490108	9.238807268	20.06553478	0.353678517	0.764125594	4.340978651	2.118020328
P29595	NEDD8 OS=Mus musculus OX=10090 GN=Nedd8 PE=1 SV=2 - [NEDD8_MOUSE]	Nedd8	60.53948741	0	0	94.63606282	173.8483716	0	20.17982914	89.49481147	34.95248935	87.03814351	0.353678517	0.764125594	4.434864679	2.148890084
O08810	116 kDa U5 small nuclear ribonucleoprotein component OS=Mus musculus OX=10090 GN=Eftud2 PE=1 SV=1 - [U5S1_MOUSE]	Eftud2	0	464.9347707	0	1042.876013	1122.280492	0	154.9782569	721.718835	268.430215	626.2865362	0.353678517	0.764125594	4.65690381	2.219371083
A2AGT5	Cytoskeleton-associated protein 5 OS=Mus musculus OX=10090 GN=Ckap5 PE=1 SV=1 - [CKAP5_MOUSE]	Ckap5	45.27019909	0	0	148.4697514	67.36733036	0	15.09006636	71.94569392	26.13676163	74.34068758	0.353678517	0.764125594	4.767751989	2.253309191
Q9DCB1	High mobility group nucleosome-binding domain-containing protein 3 OS=Mus musculus OX=10090 GN=Hmgn3 PE=1 SV=1 - [HMGN3_MOUSE]	Hmgn3	18.31159558	0	0	40.26656749	48.52324488	0	6.103865193	29.59660412	10.57220464	25.96174902	0.353678517	0.764125594	4.848829911	2.277636647
Q6P4T2	U5 small nuclear ribonucleoprotein 200 kDa helicase OS=Mus musculus OX=10090 GN=Snrnp200 PE=1 SV=1 - [U520_MOUSE]	Snrnp200	10.93402047	0	0	31.12384941	23.99552466	0	3.644673491	18.37312469	6.312759664	16.30588963	0.353678517	0.764125594	5.041089342	2.333735523
Q8K3H0	DCC-interacting protein 13-alpha OS=Mus musculus OX=10090 GN=Appl1 PE=1 SV=1 - [DP13A_MOUSE]	Appl1	6.345745079	0	0	18.52948616	14.22607946	0	2.11524836	10.91852188	3.66371763	9.697443852	0.353678517	0.764125594	5.16181555	2.36787859
P62843	40S ribosomal protein S15 OS=Mus musculus OX=10090 GN=Rps15 PE=1 SV=2 - [RS15_MOUSE]	Rps15	12.30979687	0	0	15.87021074	58.02235427	0	4.103265624	24.630855	7.107064538	29.98683152	0.353678517	0.764125594	6.002744463	2.585622254
Q69ZK9	Neuroligin-2 OS=Mus musculus OX=10090 GN=Nlgn2 PE=1 SV=2 - [NLGN2_MOUSE]	Nlgn2	0	20.25517653	0	65.48963572	0	57.58763612	6.751725508	41.02575728	11.69433162	35.74835618	0.353678517	0.764125594	6.076336668	2.603201807
P30681	High mobility group protein B2 OS=Mus musculus OX=10090 GN=Hmgb2 PE=1 SV=3 - [HMGB2_MOUSE]	Hmgb2	57.10541214	0	0	145.7255655	210.82747	0	19.03513738	118.8510119	32.96982507	107.9524745	0.353678517	0.764125594	6.243769586	2.642417299
Q9CQ48	NudC domain-containing protein 2 OS=Mus musculus OX=10090 GN=Nudcd2 PE=1 SV=1 - [NUDC2_MOUSE]	Nudcd2	13.1260857	0	0	30.27032847	59.34089308	0	4.375361901	29.87040718	7.578349113	29.67246789	0.353678517	0.764125594	6.826956915	2.771242647
P31938	Dual specificity mitogen-activated protein kinase kinase 1 OS=Mus musculus OX=10090 GN=Map2k1 PE=1 SV=2 - [MP2K1_MOUSE]	Map2k1	19.76189961	0	0	111.8457492	35.60287228	0	6.587299871	49.14954048	11.40953806	57.14019659	0.353678517	0.764125594	7.461257487	2.899418796
O89112	Glutathione S-transferase LANCL1 OS=Mus musculus OX=10090 GN=Lancl1 PE=1 SV=1 - [LANC1_MOUSE]	Lancl1	17.48284321	0	0	67.83499805	82.12967572	0	5.827614404	49.98822459	10.09372423	43.87711705	0.353678517	0.764125594	8.57781952	3.100610962
Q60967	Bifunctional 3'-phosphoadenosine 5'-phosphosulfate synthase 1 OS=Mus musculus OX=10090 GN=Papss1 PE=1 SV=1 - [PAPS1_MOUSE]	Papss1	20.12357888	0	0	102.7112997	100.6613168	0	6.707859625	67.79087215	11.61835368	58.71756439	0.353678517	0.764125594	10.10618527	3.337166628
O09159	Lysosomal alpha-mannosidase OS=Mus musculus OX=10090 GN=Man2b1 PE=1 SV=4 - [MA2B1_MOUSE]	Man2b1	0	9.104423353	0	0	78.09156025	14.51045024	3.034807784	30.86733683	5.256441274	41.53593323	0.353678517	0.764125594	10.17110111	3.346403967
Q8CG72	ADP-ribose glycohydrolase ARH3 OS=Mus musculus OX=10090 GN=Adprs PE=1 SV=1 - [ADPRS_MOUSE]	Adprs	65.98689547	28.31002437	35.43224983	54.28428585	0	0	43.24305656	18.09476195	20.01607305	31.34104705	0.375825087	0.764125594	0.418443177	-1.256896373
Q7TPD3	Roundabout homolog 2 OS=Mus musculus OX=10090 GN=Robo2 PE=1 SV=2 - [ROBO2_MOUSE]	Robo2	18.42642192	26.72803163	28.55123414	9.381879241	9.381879241	28.14563772	24.56856256	15.6364654	5.396798642	10.83326101	0.375825087	0.764125594	0.636442013	-0.651899022
P00493	Hypoxanthine-guanine phosphoribosyltransferase OS=Mus musculus OX=10090 GN=Hprt1 PE=1 SV=3 - [HPRT_MOUSE]	Hprt1	90.42261879	30.14087293	30.14087293	91.47531263	76.63705153	56.03745472	50.23478822	74.71660629	34.8036822	17.79681224	0.375825087	0.764125594	1.487347891	0.572742133
P26350	Prothymosin alpha OS=Mus musculus OX=10090 GN=Ptma PE=1 SV=2 - [PTMA_MOUSE]	Ptma	135.6000698	0	0	7.477757009	232.9111843	12.64257155	45.20002327	84.34383761	78.28873682	128.6890096	0.375825087	0.764125594	1.866013146	0.89995915
Q8VBW6	NEDD8-activating enzyme E1 regulatory subunit OS=Mus musculus OX=10090 GN=Nae1 PE=1 SV=1 - [ULA1_MOUSE]	Nae1	62.0762592	0	0	36.49200708	70.70799874	12.5690378	20.6920864	39.92301454	35.83974496	29.22094375	0.375825087	0.764125594	1.929385649	0.94814154
Q8WTY4	Anamorsin OS=Mus musculus OX=10090 GN=Ciapin1 PE=1 SV=1 - [CPIN1_MOUSE]	Ciapin1	73.86270477	0	0	79.24573224	39.24152673	35.50299287	24.62090159	51.33008395	42.64465248	24.24781895	0.375825087	0.764125594	2.084817396	1.059921027
Q80UG5	Septin-9 OS=Mus musculus OX=10090 GN=Septin9 PE=1 SV=1 - [SEPT9_MOUSE]	Septin9	145.8904286	0	0	121.5625517	162.0086417	21.25609988	48.63014285	101.6090978	84.2298782	72.46671478	0.375825087	0.764125594	2.089426265	1.063106848

Q64331	Unconventional myosin-VI OS=Mus musculus OX=10090 GN=Myo6 PE=1 SV=1 - [MYO6_MOUSE]	Myo6	45.27154519	0	0	35.80062182	46.5212831	13.50262555	15.09051506	31.94151015	26.1375388	16.84421247	0.375825087	0.764125594	2.116661361	1.081790475
O70493	Sorting nexin-12 OS=Mus musculus OX=10090 GN=Snx12 PE=1 SV=1 - [SNX12_MOUSE]	Snx12	66.00982966	0	0	80.16580249	46.08110281	14.09657029	22.00327655	46.78115853	38.11079292	33.04017886	0.375825087	0.764125594	2.126099648	1.088209216
Q8BP40	Lysophosphatidic acid phosphatase type 6 OS=Mus musculus OX=10090 GN=Acp6 PE=1 SV=1 - [PPA6_MOUSE]	Acp6	171.0581331	0	0	98.39690111	178.676479	92.35779338	57.01937772	123.1437245	98.76045922	48.18747579	0.375825087	0.764125594	2.159682014	1.110818909
P62717	60S ribosomal protein L18a OS=Mus musculus OX=10090 GN=Rpl18a PE=1 SV=1 - [RL18A_MOUSE]	Rpl18a	104.6248007	0	0	69.51520522	123.9557492	33.22896803	34.87493356	75.56664083	60.40515684	45.66510826	0.375825087	0.764125594	2.166789528	1.115559023
Q9CSU0	Regulation of nuclear pre-mRNA domain-containing protein 1B OS=Mus musculus OX=10090 GN=Rprd1b PE=1 SV=2 - [RPR1B_MOUSE]	Rprd1b	37.56604173	0	0	20.63395316	40.52359116	21.37826834	12.52201391	27.51193755	21.68876431	11.27456644	0.375825087	0.764125594	2.197085688	1.135591137
P62317	Small nuclear ribonucleoprotein Sm D2 OS=Mus musculus OX=10090 GN=Snrpd2 PE=1 SV=1 - [SMD2_MOUSE]	Snrpd2	137.0915082	0	0	76.04106461	161.8203748	64.97650449	45.69716939	100.9459813	79.14981915	53.00825371	0.375825087	0.764125594	2.209020442	1.143406769
P63073	Eukaryotic translation initiation factor 4E OS=Mus musculus OX=10090 GN=Eif4e PE=1 SV=1 - [IF4E_MOUSE]	Eif4e	178.95919	0	0	191.3252619	130.3226307	75.69375673	59.65306333	132.4472164	103.3221365	57.84502264	0.375825087	0.764125594	2.220291952	1.150749393
P97351	40S ribosomal protein S3a OS=Mus musculus OX=10090 GN=Rps3a PE=1 SV=3 - [RS3A_MOUSE]	Rps3a	168.8477925	0	0	110.2221856	229.7841139	36.75757462	56.2825975	125.587958	97.48431846	97.42633851	0.375825087	0.764125594	2.231381699	1.157937321
Q6A4J8	Ubiquitin carboxyl-terminal hydrolase 7 OS=Mus musculus OX=10090 GN=Usp7 PE=1 SV=1 - [UBP7_MOUSE]	Usp7	17.21747127	0	0	13.03825978	17.92575776	7.664559657	5.739157092	12.8761924	9.940511675	5.132518485	0.375825087	0.764125594	2.243568558	1.16579527
P11103	Poly [ADP-ribose] polymerase 1 OS=Mus musculus OX=10090 GN=Parp1 PE=1 SV=3 - [PARP1_MOUSE]	Parp1	64.55914463	0	0	52.07974565	80.67623809	12.74301368	21.51971488	48.49966581	37.27323953	34.10782129	0.375825087	0.764125594	2.253731803	1.172315843
P70318	Nucleolysin TIAR OS=Mus musculus OX=10090 GN=Tial1 PE=1 SV=1 - [TIAR_MOUSE]	Tial1	113.1164059	0	0	91.44615802	122.4501027	45.11624131	37.70546865	86.33750069	65.30778742	38.91921525	0.375825087	0.764125594	2.289787232	1.195213549
Q91VZ6	Stromal membrane-associated protein 1 OS=Mus musculus OX=10090 GN=Smap1 PE=1 SV=1 - [SMAP1_MOUSE]	Smap1	75.11331317	0	0	75.53105145	69.82079666	30.21217756	25.03777106	58.52134189	43.36669158	24.6821462	0.375825087	0.764125594	2.33732235	1.224856716
P55012	Solute carrier family 12 member 2 OS=Mus musculus OX=10090 GN=Slc12a2 PE=1 SV=2 - [S12A2_MOUSE]	Slc12a2	46.50823278	0	0	51.4461764	30.21452225	27.22023288	15.50274426	36.29364384	26.85154072	13.20760669	0.375825087	0.764125594	2.341110918	1.227193288
Q99KQ4	Nicotinamide phosphoribosyltransferase OS=Mus musculus OX=10090 GN=Nampt PE=1 SV=1 - [NAMPT_MOUSE]	Nampt	55.6101862	0	0	72.28929789	40.51710126	17.89646892	18.53672873	43.56762269	32.10655597	27.32442537	0.375825087	0.764125594	2.350340414	1.232869726
Q7TNC4	Putative RNA-binding protein Luc7-like 2 OS=Mus musculus OX=10090 GN=Luc7l2 PE=1 SV=1 - [LC7L2_MOUSE]	Luc7l2	20.92284262	0	0	16.75756402	23.11042956	9.778567505	6.974280872	16.5488537	12.07980882	6.668381098	0.375825087	0.764125594	2.372840154	1.246614917
P35980	60S ribosomal protein L18 OS=Mus musculus OX=10090 GN=Rpl18 PE=1 SV=3 - [RL18_MOUSE]	Rpl18	132.537567	0	0	94.32906296	167.415573	57.74743968	44.179189	106.4973586	76.52059998	55.83749127	0.375825087	0.764125594	2.410577491	1.269378807
Q9QZB7	Actin-related protein 10 OS=Mus musculus OX=10090 GN=Actr10 PE=1 SV=2 - [ARP10_MOUSE]	Actr10	19.8632526	0	0	15.56921148	24.92865697	7.902935766	6.621084198	16.13360141	11.46805423	8.526880885	0.375825087	0.764125594	2.436700837	1.284929137
Q9Z1Z2	Serine-threonine kinase receptor-associated protein OS=Mus musculus OX=10090 GN=Strap PE=1 SV=2 - [STRAP_MOUSE]	Strap	103.0569293	0	0	90.52895284	117.0414361	45.10157765	34.35230977	84.22398885	59.49994589	36.38200428	0.375825087	0.764125594	2.451770766	1.293824097
O70305	Ataxin-2 OS=Mus musculus OX=10090 GN=Atxn2 PE=1 SV=1 - [ATX2_MOUSE]	Atxn2	25.66762897	0	0	29.08644772	23.65240095	10.30721803	8.555876322	21.01535557	14.81921249	9.663352073	0.375825087	0.764125594	2.456248171	1.296456333
Q8C052	Microtubule-associated protein 1S OS=Mus musculus OX=10090 GN=Map1s PE=1 SV=2 - [MAP1S_MOUSE]	Map1s	27.20473608	0	0	23.01110913	34.22978262	9.588632387	9.068245358	22.27650804	15.7066617	12.33698911	0.375825087	0.764125594	2.456540065	1.296627769
Q9D172	Glutamine amidotransferase-like class 1 domain-containing protein 3A, mitochondrial OS=Mus musculus OX=10090 GN=Gatd3a PE=1 SV=1 - [GAL3A_MOU	Gatd3a	131.063905	0	0	121.8651856	131.6779631	70.00549351	43.68796834	107.8495474	75.66978084	33.13913037	0.375825087	0.764125594	2.468632704	1.303712201
Q91YT7	YTH domain-containing family protein 2 OS=Mus musculus OX=10090 GN=Ythdf2 PE=1 SV=1 - [YTHD2_MOUSE]	Ythdf2	78.31161335	0	0	58.57333271	86.91537378	49.04525375	26.10387112	64.84465342	45.21323105	19.69856836	0.375825087	0.764125594	2.484101041	1.312723856
Q9CR86	Calcium-regulated heat stable protein 1 OS=Mus musculus OX=10090 GN=Carhsp1 PE=1 SV=1 - [CHSP1_MOUSE]	Carhsp1	174.4198985	0	0	163.47422	197.0157627	76.04523506	58.13996616	145.5117393	100.7013753	62.45362419	0.375825087	0.764125594	2.502783349	1.323533411
Q91W50	Cold shock domain-containing protein E1 OS=Mus musculus OX=10090 GN=Csde1 PE=1 SV=1 - [CSDE1_MOUSE]	Csde1	30.40634455	0	0	28.63535654	34.8819757	12.86250905	10.13544818	25.4599471	17.55511121	11.34797985	0.375825087	0.764125594	2.511970526	1.328819536
Q8K2I4	Beta-mannosidase OS=Mus musculus OX=10090 GN=Manba PE=1 SV=1 - [MANBA_MOUSE]	Manba	12.27418982	0	0	8.606120632	13.69996514	8.805414174	4.091396608	10.37049998	7.086506799	2.885122726	0.375825087	0.764125594	2.534709043	1.341820151
P61750	ADP-ribosylation factor 4 OS=Mus musculus OX=10090 GN=Arf4 PE=1 SV=2 - [ARF4_MOUSE]	Arf4	421.6023606	0	0	341.2908615	477.4402128	255.0952096	140.5341202	357.9420946	243.4122364	112.1038482	0.375825087	0.764125594	2.547012029	1.348805773
Q6PE01	U5 small nuclear ribonucleoprotein 40 kDa protein OS=Mus musculus OX=10090 GN=Snrnp40 PE=1 SV=1 - [SNR40_MOUSE]	Snrnp40	33.40649597	0	0	48.37411335	31.5471209	5.360668102	11.13549866	28.42730079	19.28724944	21.67577164	0.375825087	0.764125594	2.552853865	1.352110955
Q9D6Y9	1,4-alpha-glucan-branching enzyme OS=Mus musculus OX=10090 GN=Gbe1 PE=1 SV=1 - [GLGB_MOUSE]	Gbe1	34.3291473	0	0	15.14391714	45.3561529	28.83869551	11.4430491	29.77958851	19.81994244	15.12807843	0.375825087	0.764125594	2.602417262	1.379852297
Q99JW2	Aminoacylase-1 OS=Mus musculus OX=10090 GN=Acy1 PE=1 SV=1 - [ACY1_MOUSE]	Acy1	69.50726525	0	0	71.71149473	67.04491202	42.21274809	23.16908842	60.32305162	40.1300383	15.85659386	0.375825087	0.764125594	2.603600562	1.380508131
Q8BMP6	Golgi resident protein GCP60 OS=Mus musculus OX=10090 GN=Acbd3 PE=1 SV=3 - [GCP60_MOUSE]	Acbd3	36.09737861	0	0	37.52822949	34.22425902	23.11885179	12.03245954	31.6237801	20.84083126	7.548470663	0.375825087	0.764125594	2.628205813	1.394078257
Q9WV02	RNA-binding motif protein, X chromosome OS=Mus musculus OX=10090 GN=Rbmx PE=1 SV=1 - [RBMX_MOUSE]	Rbmx	1002.137686	0	0	1029.597548	937.1712528	672.1219999	334.0458952	879.6302668	578.5844625	185.5543468	0.375825087	0.764125594	2.633261715	1.396850915
P27601	Guanine nucleotide-binding protein subunit alpha-13 OS=Mus musculus OX=10090 GN=Gna13 PE=1 SV=1 - [GNA13_MOUSE]	Gna13	188.14061	0	0	111.360407	177.5363293	207.780677	62.71353666	165.5591378	108.6230318	49.31335471	0.375825087	0.764125594	2.639926667	1.400497854
P54823	Probable ATP-dependent RNA helicase DDX6 OS=Mus musculus OX=10090 GN=Ddx6 PE=1 SV=1 - [DDX6_MOUSE]	Ddx6	112.2817739	0	0	114.8064484	112.1831519	69.86568644	37.42725797	98.95176224	64.82591238	25.22340729	0.375825087	0.764125594	2.643842152	1.402636045
P26638	Serine--tRNA ligase, cytoplasmic OS=Mus musculus OX=10090 GN=Sars1 PE=1 SV=3 - [SYSC_MOUSE]	Sars1	62.03626183	0	0	77.59721192	57.93587779	28.67139599	20.67875394	54.73482857	35.81665247	24.61948219	0.375825087	0.764125594	2.646911353	1.404309878
Q99K51	Plastin-3 OS=Mus musculus OX=10090 GN=Pls3 PE=1 SV=3 - [PLST_MOUSE]	Pls3	88.46099667	0	0	108.1145896	82.5915403	47.63546371	29.48699889	79.44719787	51.07298024	30.36192244	0.375825087	0.764125594	2.694312777	1.42991734
Q9DCG9	Multifunctional methyltransferase subunit TRM112-like protein OS=Mus musculus OX=10090 GN=Trmt112 PE=1 SV=1 - [TR112_MOUSE]	Trmt112	66.33511109	0	0	65.74253677	79.00830804	34.57281865	22.1117037	59.77455449	38.29859424	22.81097956	0.375825087	0.764125594	2.703299362	1.434721284
Q9CQ80	Vacuolar protein-sorting-associated protein 25 OS=Mus musculus OX=10090 GN=Vps25 PE=1 SV=1 - [VPS25_MOUSE]	Vps25	20.65187257	0	0	20.54589706	23.25869596	12.44986874	6.883957523	18.75148726	11.92336419	5.623399006	0.375825087	0.764125594	2.72394	1.445694926
Q9QZE5	Coatomer subunit gamma-1 OS=Mus musculus OX=10090 GN=Copg1 PE=1 SV=1 - [COPG1_MOUSE]	Copg1	47.60820005	0	0	45.51811715	51.82228166	32.44679961	15.86940002	43.26239947	27.48660712	9.882738635	0.375825087	0.764125594	2.726152181	1.4468661
Q8K183	Pyridoxal kinase OS=Mus musculus OX=10090 GN=Pdxk PE=1 SV=1 - [PDXK_MOUSE]	Pdxk	25.70717945	0	0	25.77501573	24.768629	19.87738248	8.569059818	23.47367574	14.84204698	3.154868847	0.375825087	0.764125594	2.739352535	1.453834943
P13707	Glycerol-3-phosphate dehydrogenase [NAD(+)], cytoplasmic OS=Mus musculus OX=10090 GN=Gpd1 PE=1 SV=3 - [GPDA_MOUSE]	Gpd1	154.3422805	0	0	139.5561357	176.5418359	108.9597018	51.44742682	141.6858911	89.10955717	33.84136677	0.375825087	0.764125594	2.753993734	1.461525277
Q9D868	Peptidyl-prolyl cis-trans isomerase H OS=Mus musculus OX=10090 GN=Ppih PE=1 SV=1 - [PPIH_MOUSE]	Ppih	73.17555428	0	0	72.24824258	80.25319277	49.97336437	24.39185143	67.49159991	42.24792596	15.6903231	0.375825087	0.764125594	2.766973229	1.468308686
P70699	Lysosomal alpha-glucosidase OS=Mus musculus OX=10090 GN=Gaa PE=1 SV=2 - [LYAG_MOUSE]	Gaa	26.07950389	0	0	16.21037045	46.43505494	9.752954058	8.693167962	24.13279315	15.05700859	19.58233176	0.375825087	0.764125594	2.77606429	1.473040979
Q9CPR4	60S ribosomal protein L17 OS=Mus musculus OX=10090 GN=Rpl17 PE=1 SV=3 - [RL17_MOUSE]	Rpl17	76.90360542	0	0	67.87096177	93.56665712	52.06766901	25.63453514	71.1684293	44.40031729	20.9450823	0.375825087	0.764125594	2.776271499	1.47314866
P61202	COP9 signalosome complex subunit 2 OS=Mus musculus OX=10090 GN=Cops2 PE=1 SV=1 - [CSN2_MOUSE]	Cops2	48.24492752	0	0	37.23863791	59.11936765	40.79963207	16.08164251	45.71921255	27.85422189	11.7406681	0.375825087	0.764125594	2.842944216	1.507385791
P46638	Ras-related protein Rab-11B OS=Mus musculus OX=10090 GN=Rab11b PE=1 SV=3 - [RB11B_MOUSE]	Rab11b	240.9319631	0	0	304.1471848	216.9662445	170.3680264	80.31065436	230.4938185	139.1021337	67.90774808	0.375825087	0.764125594	2.870027898	1.521064761
P62900	60S ribosomal protein L31 OS=Mus musculus OX=10090 GN=Rpl31 PE=1 SV=1 - [RL31_MOUSE]	Rpl31	131.5375901	0	0	119.5310587	178.8878798	79.50495894	43.84586338	125.9746325	75.94326308	50.00380957	0.375825087	0.764125594	2.873124686	1.522620603
Q9CR50	RING finger and CHY zinc finger domain-containing protein 1 OS=Mus musculus OX=10090 GN=Rchy1 PE=1 SV=1 - [ZN363_MOUSE]	Rchy1	8.311885002	0	0	11.83293443	7.133994214	5.239638731	2.770628334	8.068855792	4.798869043	3.394607735	0.375825087	0.764125594	2.91228372	1.542150913
P63168	Dynein light chain 1, cytoplasmic OS=Mus musculus OX=10090 GN=Dynll1 PE=1 SV=1 - [DYL1_MOUSE]	Dynll1	227.8681388	0	0	285.8192162	215.2628061	162.7931053	75.95604627	221.2917092	131.5597313	61.7342429	0.375825087	0.764125594	2.913417958	1.542712685
Q9CWW6	Peptidyl-prolyl cis-trans isomerase NIMA-interacting 4 OS=Mus musculus OX=10090 GN=Pin4 PE=1 SV=1 - [PIN4_MOUSE]	Pin4	46.45532642	0	0	39.16589133	63.87482389	36.17443205	15.48510881	46.40504909	26.82099521	15.20302538	0.375825087	0.764125594	2.996753182	1.583400266
P23591	GDP-L-fucose synthase OS=Mus musculus OX=10090 GN=Gfus PE=1 SV=3 - [FCL_MOUSE]	Gfus	35.61293231	0	0	60.29222465	31.98624174	15.29216696	11.87097744	35.85687778	20.56113605	22.74835522	0.375825087	0.764125594	3.020549738	1.594811143
P62242	40S ribosomal protein S8 OS=Mus musculus OX=10090 GN=Rps8 PE=1 SV=2 - [RS8_MOUSE]	Rps8	124.4505875	0	0	88.25348326	202.2631979	102.860623	41.48352917	131.1257681	71.8515802	62.03823482	0.375825087	0.764125594	3.160911588	1.660340683
P52293	Importin subunit alpha-1 OS=Mus musculus OX=10090 GN=Kpna2 PE=1 SV=2 - [IMA1_MOUSE]	Kpna2	28.48942864	0	0	26.89444293	41.74802041	22.06245691	9.496476214	30.23497341	16.4483793	10.25912816	0.375825087	0.764125594	3.183809735	1.670754123
Q6PAL1	Draxin OS=Mus musculus OX=10090 GN=Draxin PE=1 SV=2 - [DRAXI_MOUSE]	Draxin	58.17829341	0	0	45.9829939	109.6660152	36.17792844	19.39276447	63.94231252	33.58925336	39.90022016	0.375825087	0.764125594	3.297225242	1.721252444
Q3THK7	GMP synthase [glutamine-hydrolyzing] OS=Mus musculus OX=10090 GN=Gmps PE=1 SV=2 - [GUAA_MOUSE]	Gmps	30.92237094	0	0	27.21469818	65.69754604	12.50918782	10.30745698	35.14047735	17.85303918	27.46568485	0.375825087	0.764125594	3.409228621	1.769445349
Q8BYM5	Neuroligin-3 OS=Mus musculus OX=10090 GN=Nlgn3 PE=1 SV=2 - [NLGN3_MOUSE]	Nlgn3	0	0	14.55648207	12.77048477	11.89275726	25.38756564	4.852160688	16.68360256	8.404188839	7.550617945	0.375825087	0.764125594	3.438386242	1.781731615
Q60737	Casein kinase II subunit alpha OS=Mus musculus OX=10090 GN=Csnk2a1 PE=1 SV=2 - [CSK21_MOUSE]	Csnk2a1	16.82019078	0	0	14.5832133	30.81721438	12.52996342	5.606730261	19.31013037	9.711141676	10.01816828	0.375825087	0.764125594	3.444098337	1.784126336
Q64012	RNA-binding protein Raly OS=Mus musculus OX=10090 GN=Raly PE=1 SV=3 - [RALY_MOUSE]	Raly	12.67248833	0	0	12.19966287	32.55614502	7.514213947	4.224162777	17.42334061	7.316464549	13.31313956	0.375825087	0.764125594	4.124684945	2.044283927
Q9JKR6	Hypoxia up-regulated protein 1 OS=Mus musculus OX=10090 GN=Hyou1 PE=1 SV=1 - [HYOU1_MOUSE]	Hyou1	9.035391075	75.22379725	134.4769509	0	18.36350835	9.38062311	72.9120464	9.24804382	62.75272404	9.182472035	0.4	0.764125594	0.126838352	-2.978937055
P02088	Hemoglobin subunit beta-1 OS=Mus musculus OX=10090 GN=Hbb-b1 PE=1 SV=2 - [HBB1_MOUSE]	Hbb-b1	15.74369715	64.79650082	108.5084261	33.44220608	21.13143484	0	63.01620801	18.19121364	46.40798224	16.91386894	0.4	0.764125594	0.288675155	-1.792481147
Q9EQH2	Endoplasmic reticulum aminopeptidase 1 OS=Mus musculus OX=10090 GN=Erap1 PE=1 SV=2 - [ERAP1_MOUSE]	Erap1	28.91323127	90.51641439	88.96325618	38.56394532	0	35.22860419	69.46430061	24.5975165	35.1268415	21.36725272	0.4	0.764125594	0.354102989	-1.497759072
Q9ERE7	LRP chaperone MESD OS=Mus musculus OX=10090 GN=Mesd PE=1 SV=1 - [MESD_MOUSE]	Mesd	98.68196904	217.9864506	250.7969775	108.2923704	0	115.7623515	189.1551324	74.6849073	80.0510939	64.78677865	0.4	0.764125594	0.394834157	-1.340681291
P97797	Tyrosine-protein phosphatase non-receptor type substrate 1 OS=Mus musculus OX=10090 GN=Sirpa PE=1 SV=2 - [SHPS1_MOUSE]	Sirpa	80.03718469	48.41422082	48.31147488	29.83699165	62.92469363	0	58.92096013	30.92056176	18.28725906	31.4763381	0.4	0.764125594	0.524780345	-0.930214409
O35608	Angiopoietin-2 OS=Mus musculus OX=10090 GN=Angpt2 PE=2 SV=2 - [ANGP2_MOUSE]	Angpt2	81.40358285	51.90508855	89.74813546	53.65730357	0	73.46551971	74.35226895	42.37427443	19.88252551	38.0102053	0.4	0.764125594	0.569912324	-0.811188103
Q9JKZ2	Sodium/myo-inositol cotransporter OS=Mus musculus OX=10090 GN=Slc5a3 PE=1 SV=2 - [SC5A3_MOUSE]	Slc5a3	31.91723315	87.03525323	91.9007111	0	44.09366413	76.43287946	70.28439916	40.17551453	33.31587821	38.36678496	0.4	0.764125594	0.571613545	-0.806887993
E9PVD3	Protocadherin-16 OS=Mus musculus OX=10090 GN=Dchs1 PE=1 SV=1 - [PCD16_MOUSE]	Dchs1	25.20792429	58.56988293	57.89901091	0	33.53251214	54.50481067	47.22560604	29.34577427	19.07082194	27.49254766	0.4	0.764125594	0.621395398	-0.686416538
Q99LP6	GrpE protein homolog 1, mitochondrial OS=Mus musculus OX=10090 GN=Grpel1 PE=1 SV=1 - [GRPE1_MOUSE]	Grpel1	56.2356793	78.80392832	105.7418719	57.05610493	34.88177091	58.25630777	80.26049317	50.06472787	24.78521661	13.16251337	0.4	0.764125594	0.623777975	-0.680895482
Q6P9S0	Protein MTSS 2 OS=Mus musculus OX=10090 GN=Mtss2 PE=1 SV=1 - [MTSS2_MOUSE]	Mtss2	33.21160097	56.11661814	56.24431099	0	45.06313669	45.74864067	48.5241767	30.27059245	13.26123328	26.21734263	0.4	0.764125594	0.623824957	-0.680786823
P20060	Beta-hexosaminidase subunit beta OS=Mus musculus OX=10090 GN=Hexb PE=1 SV=2 - [HEXB_MOUSE]	Hexb	93.3214239	117.3663369	114.0914599	86.43992744	116.9372506	0	108.2597402	67.79239269	13.04017547	60.65787715	0.4	0.764125594	0.626201324	-0.675301536
Q8C176	Transcription initiation factor TFIID subunit 2 OS=Mus musculus OX=10090 GN=Taf2 PE=2 SV=2 - [TAF2_MOUSE]	Taf2	611.39184	1063.006489	654.8608022	345.8200319	621.6254107	617.3080442	776.4197103	528.2511623	249.1412728	158.0047401	0.4	0.764125594	0.680368047	-0.555612707
P12246	Serum amyloid P-component OS=Mus musculus OX=10090 GN=Apcs PE=1 SV=2 - [SAMP_MOUSE]	Apcs	50.82007709	115.6858113	103.2650417	49.95885299	55.43936074	80.14523823	89.92364335	61.84781732	34.42942855	16.08122198	0.4	0.764125594	0.687781489	-0.539977807
P62858	40S ribosomal protein S28 OS=Mus musculus OX=10090 GN=Rps28 PE=1 SV=1 - [RS28_MOUSE]	Rps28	509.7230368	1095.182624	1395.731084	480.9369605	590.7581771	1025.666188	1000.212248	699.1204417	450.5741828	288.0785795	0.4	0.764125594	0.698972086	-0.516693253
P32261	Antithrombin-III OS=Mus musculus OX=10090 GN=Serpinc1 PE=1 SV=1 - [ANT3_MOUSE]	Serpinc1	284.1231221	516.675487	574.9103973	247.1110856	381.933019	374.7862472	458.5696688	334.6101172	153.8555311	75.86059251	0.4	0.764125594	0.729682184	-0.454659867
Q9WTQ5	A-kinase anchor protein 12 OS=Mus musculus OX=10090 GN=Akap12 PE=1 SV=1 - [AKA12_MOUSE]	Akap12	96.18141894	101.7884738	165.9382577	88.68337371	62.00502957	115.2796822	121.3027168	88.6560285	38.75704318	26.63733685	0.4	0.764125594	0.730865976	-0.452321222
P08752	Guanine nucleotide-binding protein G(i) subunit alpha-2 OS=Mus musculus OX=10090 GN=Gnai2 PE=1 SV=5 - [GNAI2_MOUSE]	Gnai2	257.3607669	186.0598414	190.4069979	120.8136345	156.5035715	190.6073328	211.2758688	155.9748463	39.9698364	34.89985304	0.4	0.764125594	0.738252065	-0.437814609
P11276	Fibronectin OS=Mus musculus OX=10090 GN=Fn1 PE=1 SV=4 - [FINC_MOUSE]	Fn1	105.2760153	232.9584279	216.8078115	102.9327952	130.4622809	176.6440206	185.0140849	136.6796989	69.52575357	37.24685762	0.4	0.764125594	0.738752939	-0.436836131
Q6P5F6	Zinc transporter ZIP10 OS=Mus musculus OX=10090 GN=Slc39a10 PE=1 SV=1 - [S39AA_MOUSE]	Slc39a10	38.53569768	55.66314081	58.62875539	22.51534448	32.60837992	57.89846458	50.94253129	37.67406299	10.84646752	18.2273738	0.4	0.764125594	0.739540459	-0.435299017
P61924	Coatomer subunit zeta-1 OS=Mus musculus OX=10090 GN=Copz1 PE=1 SV=1 - [COPZ1_MOUSE]	Copz1	69.73790601	103.7976634	119.2510991	72.91738338	80.32663496	66.33397531	97.59555618	73.19266455	25.33256152	7.000390404	0.4	0.764125594	0.749958988	-0.415116392
Q62084	Protein phosphatase 1 regulatory subunit 14B OS=Mus musculus OX=10090 GN=Ppp1r14b PE=1 SV=2 - [PP14B_MOUSE]	Ppp1r14b	194.8870282	143.2375473	144.1708669	172.5511018	101.3709639	88.72137485	160.7651475	120.8811469	29.55410004	45.19226934	0.4	0.764125594	0.751911398	-0.411365423
F6ZDS4	Nucleoprotein TPR OS=Mus musculus OX=10090 GN=Tpr PE=1 SV=1 - [TPR_MOUSE]	Tpr	35.84159965	20.80052592	21.1494546	28.13613386	17.00948268	13.72815527	25.93052672	19.6245906	8.585013845	7.551592802	0.4	0.764125594	0.756814191	-0.401988954
Q8K0T0	Reticulon-1 OS=Mus musculus OX=10090 GN=Rtn1 PE=1 SV=1 - [RTN1_MOUSE]	Rtn1	123.7916994	92.23875419	72.0101512	71.0553966	93.14309454	54.69068554	96.01353493	72.96305889	26.09633903	19.29705472	0.4	0.764125594	0.759924723	-0.396071581
Q9WVJ9	EGF-containing fibulin-like extracellular matrix protein 2 OS=Mus musculus OX=10090 GN=Efemp2 PE=1 SV=1 - [FBLN4_MOUSE]	Efemp2	80.58285703	145.9721722	165.3214759	44.41158756	123.1009241	131.0928295	130.6255017	99.5351137	44.40493896	47.90532318	0.4	0.764125594	0.761988374	-0.392159108
Q8CIN4	Serine/threonine-protein kinase PAK 2 OS=Mus musculus OX=10090 GN=Pak2 PE=1 SV=1 - [PAK2_MOUSE]	Pak2	104.5765109	63.40844295	53.74190974	46.63295776	79.00335639	44.43622063	73.90895452	56.69084493	26.99508706	19.35439328	0.4	0.764125594	0.767036218	-0.382633395
Q99NB9	Splicing factor 3B subunit 1 OS=Mus musculus OX=10090 GN=Sf3b1 PE=1 SV=1 - [SF3B1_MOUSE]	Sf3b1	131.8231554	102.3139905	90.34257399	81.50032157	103.2056469	66.85848833	108.1599066	83.85481893	21.34925548	18.28761131	0.4	0.764125594	0.775285608	-0.367200212
P12815	Programmed cell death protein 6 OS=Mus musculus OX=10090 GN=Pdcd6 PE=1 SV=2 - [PDCD6_MOUSE]	Pdcd6	38.46153871	60.93513303	56.60823369	31.38365812	48.00556787	44.97918994	52.00163514	41.45613864	11.92397472	8.853298187	0.4	0.764125594	0.797208367	-0.326971243
P47963	60S ribosomal protein L13 OS=Mus musculus OX=10090 GN=Rpl13 PE=1 SV=3 - [RL13_MOUSE]	Rpl13	112.2016926	136.8427571	110.989934	82.6260235	129.7548157	78.67912839	120.0114613	97.0199892	14.58891634	28.41779628	0.4	0.764125594	0.808422697	-0.306818268
Q6DIB5	Multiple epidermal growth factor-like domains protein 10 OS=Mus musculus OX=10090 GN=Megf10 PE=1 SV=1 - [MEG10_MOUSE]	Megf10	64.63548256	58.92826936	66.78307072	44.24887095	46.16835294	64.90555799	63.44894088	51.77426063	4.059604287	11.41246383	0.4	0.764125594	0.815998816	-0.293361036
O08912	Polypeptide N-acetylgalactosaminyltransferase 1 OS=Mus musculus OX=10090 GN=Galnt1 PE=1 SV=1 - [GALT1_MOUSE]	Galnt1	36.667702	35.09721826	46.80030044	24.73337592	39.97632789	32.25044853	39.52174023	32.32005078	6.352140098	7.621714345	0.4	0.764125594	0.81777904	-0.290217008
P19324	Serpin H1 OS=Mus musculus OX=10090 GN=Serpinh1 PE=1 SV=3 - [SERPH_MOUSE]	Serpinh1	623.7470564	619.0381766	874.9373194	571.5040122	509.8019731	694.2359668	705.9075175	591.8473174	146.4030356	93.88483404	0.4	0.764125594	0.838420477	-0.254254143
P02468	Laminin subunit gamma-1 OS=Mus musculus OX=10090 GN=Lamc1 PE=1 SV=2 - [LAMC1_MOUSE]	Lamc1	93.62843342	137.4371618	102.64825	73.92462879	82.61312131	127.6585821	111.2379484	94.73211072	23.13305775	28.84418245	0.4	0.764125594	0.851616845	-0.231723608
P62751	60S ribosomal protein L23a OS=Mus musculus OX=10090 GN=Rpl23a PE=1 SV=1 - [RL23A_MOUSE]	Rpl23a	129.7398953	150.5635027	136.8533823	122.1032945	144.3574226	92.7209499	139.0522601	119.7272224	10.58451486	25.90010846	0.4	0.764125594	0.861023203	-0.215875979
Q08879	Fibulin-1 OS=Mus musculus OX=10090 GN=Fbln1 PE=1 SV=2 - [FBLN1_MOUSE]	Fbln1	102.9833143	107.3017748	85.33422501	66.27771081	97.06387865	92.01635339	98.53977138	85.11931429	11.63838977	16.51132623	0.4	0.764125594	0.863806695	-0.211219596
Q8K2Q7	BRO1 domain-containing protein BROX OS=Mus musculus OX=10090 GN=Brox PE=1 SV=1 - [BROX_MOUSE]	Brox	84.36307361	58.48629833	92.24840846	74.51176965	83.69465434	45.2684882	78.3659268	67.82497073	17.66194631	20.06682638	0.4	0.764125594	0.865490571	-0.208409993
P0DP27	Calmodulin-2 OS=Mus musculus OX=10090 GN=Calm2 PE=1 SV=1 - [CALM2_MOUSE]	Calm2	1854.350633	1375.682436	1156.695399	1596.292272	1140.840794	1065.382758	1462.242822	1267.505275	356.7915802	287.2266456	0.4	0.764125594	0.866822702	-0.206191156
B0V2N1	Receptor-type tyrosine-protein phosphatase S OS=Mus musculus OX=10090 GN=Ptprs PE=1 SV=1 - [PTPRS_MOUSE]	Ptprs	20.68653031	33.40367849	27.69576195	25.29333572	19.45310149	27.58866273	27.26199025	24.11169998	6.369661128	4.194524577	0.4	0.764125594	0.884443863	-0.177157518
Q91ZX7	Prolow-density lipoprotein receptor-related protein 1 OS=Mus musculus OX=10090 GN=Lrp1 PE=1 SV=1 - [LRP1_MOUSE]	Lrp1	116.4915894	94.18499682	124.970532	93.64265563	113.0859375	101.3300972	111.8823727	102.6862301	15.90191664	9.792324811	0.4	0.764125594	0.917805259	-0.123740021

P51655	Glypican-4 OS=Mus musculus OX=10090 GN=Gpc4 PE=1 SV=2 - [GPC4_MOUSE]	Gpc4	42.44576416	25.15559064	26.27555124	29.19603992	29.08110863	44.94055975	31.29230201	34.40590277	9.675400063	9.123461551	0.4	0.764125594	1.099500534	0.136848305
Q8BND5	Sulfhydryl oxidase 1 OS=Mus musculus OX=10090 GN=Qsox1 PE=1 SV=1 - [QSOX1_MOUSE]	Qsox1	100.0038292	132.5808977	102.1404454	107.1313278	135.0265726	128.6360323	111.5750575	123.5979775	18.22293267	14.61412561	0.4	0.764125594	1.107756342	0.147640587
Q8BFY6	Peflin OS=Mus musculus OX=10090 GN=Pef1 PE=1 SV=1 - [PEF1_MOUSE]	Pef1	44.46700162	38.4741307	47.26735316	52.49516629	55.80023469	39.10263956	43.40282849	49.13268018	4.492164139	8.842067372	0.4	0.764125594	1.132015629	0.178893876
P47911	60S ribosomal protein L6 OS=Mus musculus OX=10090 GN=Rpl6 PE=1 SV=3 - [RL6_MOUSE]	Rpl6	132.6098019	78.91226766	81.15987156	100.7928151	142.0544538	88.55010711	97.56064705	110.465792	30.37425512	28.03308117	0.4	0.764125594	1.132278181	0.179228447
P70193	Leucine-rich repeats and immunoglobulin-like domains protein 1 OS=Mus musculus OX=10090 GN=Lrig1 PE=1 SV=2 - [LRIG1_MOUSE]	Lrig1	38.52820451	25.91087848	32.95296669	43.01521959	34.31057572	34.86882909	32.46401656	37.39820813	6.322857991	4.872476265	0.4	0.764125594	1.151989559	0.204127641
Q04857	Collagen alpha-1(VI) chain OS=Mus musculus OX=10090 GN=Col6a1 PE=1 SV=1 - [CO6A1_MOUSE]	Col6a1	143.112779	212.1957546	212.4565886	176.6464908	223.1559235	255.0487291	189.2550407	218.2837145	39.96058365	39.42754821	0.4	0.764125594	1.153383887	0.205872773
Q9WTP6	Adenylate kinase 2, mitochondrial OS=Mus musculus OX=10090 GN=Ak2 PE=1 SV=5 - [KAD2_MOUSE]	Ak2	571.2138367	325.1883342	318.9964943	585.3090275	498.4875529	327.5293563	405.1328884	470.4419789	143.863636	131.1583218	0.4	0.764125594	1.161204119	0.215621595
P26040	Ezrin OS=Mus musculus OX=10090 GN=Ezr PE=1 SV=3 - [EZRI_MOUSE]	Ezr	1700.529504	1100.188707	1181.467964	1621.082991	1741.973659	1261.144367	1327.395392	1541.400339	325.6890828	250.1223616	0.4	0.764125594	1.161221704	0.215643443
P26043	Radixin OS=Mus musculus OX=10090 GN=Rdx PE=1 SV=3 - [RADI_MOUSE]	Rdx	1700.529504	1100.188707	1181.467964	1621.082991	1741.973659	1261.144367	1327.395392	1541.400339	325.6890828	250.1223616	0.4	0.764125594	1.161221704	0.215643443
P14869	60S acidic ribosomal protein P0 OS=Mus musculus OX=10090 GN=Rplp0 PE=1 SV=3 - [RLA0_MOUSE]	Rplp0	142.4053079	72.43796647	70.47717987	113.1047473	143.9242456	76.03741677	95.1068181	111.0221366	40.97342465	33.99129791	0.4	0.764125594	1.167341509	0.223226687
P50543	Protein S100-A11 OS=Mus musculus OX=10090 GN=S100a11 PE=1 SV=1 - [S10AB_MOUSE]	S100a11	559.6578203	262.4003285	278.7986384	374.9727626	604.5606457	311.3956287	366.9522624	430.309679	167.0891983	154.2175818	0.4	0.764125594	1.172658471	0.229782899
Q9R0E2	Procollagen-lysine,2-oxoglutarate 5-dioxygenase 1 OS=Mus musculus OX=10090 GN=Plod1 PE=1 SV=1 - [PLOD1_MOUSE]	Plod1	251.2266728	220.1063254	291.6268266	281.9747435	322.5535678	291.4836369	254.3199416	298.6706494	35.86044834	21.22263196	0.4	0.764125594	1.174389423	0.23191088
Q9R062	Glycogenin-1 OS=Mus musculus OX=10090 GN=Gyg1 PE=1 SV=3 - [GLYG_MOUSE]	Gyg1	104.9259696	57.70821371	50.01801152	64.70928057	124.5954659	63.10158217	70.88406494	84.1354429	29.73084688	35.04862725	0.4	0.764125594	1.186944385	0.247252339
P06797	Procathepsin L OS=Mus musculus OX=10090 GN=Ctsl PE=1 SV=2 - [CATL1_MOUSE]	Ctsl	441.8443162	725.7290157	689.4734549	566.5091139	838.8730454	830.3643267	619.0155956	745.2488287	154.5019842	154.8515864	0.4	0.764125594	1.203925772	0.267746445
Q9QZ06	Toll-interacting protein OS=Mus musculus OX=10090 GN=Tollip PE=1 SV=1 - [TOLIP_MOUSE]	Tollip	80.71858091	45.00319779	52.66705813	64.21478118	90.4192042	60.37228248	59.46294561	71.66875595	18.80253208	16.35162625	0.4	0.764125594	1.205267503	0.26935338
Q9CXW4	60S ribosomal protein L11 OS=Mus musculus OX=10090 GN=Rpl11 PE=1 SV=4 - [RL11_MOUSE]	Rpl11	214.9507302	90.4929121	96.05914545	140.8930884	241.0416533	102.0104545	133.8342626	161.3150654	70.30403069	71.7301291	0.4	0.764125594	1.205334585	0.269433675
P62320	Small nuclear ribonucleoprotein Sm D3 OS=Mus musculus OX=10090 GN=Snrpd3 PE=1 SV=1 - [SMD3_MOUSE]	Snrpd3	192.2529356	119.5041228	88.17957375	143.8158813	206.1876871	132.3048561	133.3122107	160.7694748	53.39301104	39.75218771	0.4	0.764125594	1.205962109	0.270184579
Q91VR7	Microtubule-associated proteins 1A/1B light chain 3A OS=Mus musculus OX=10090 GN=Map1lc3a PE=1 SV=1 - [MLP3A_MOUSE]	Map1lc3a	75.6232443	87.84862207	53.87161032	82.7207289	80.72651369	98.74885721	72.44782557	87.39869993	17.2096425	9.87996843	0.4	0.764125594	1.206367469	0.27066943
Q8BI72	CDKN2A-interacting protein OS=Mus musculus OX=10090 GN=Cdkn2aip PE=1 SV=1 - [CARF_MOUSE]	Cdkn2aip	9.494486063	7.384272237	5.214573956	7.456172963	8.991775992	10.2885432	7.364444085	8.912164053	2.140024948	1.417862419	0.4	0.764125594	1.21016114	0.275199164
Q9EQX4	Allograft inflammatory factor 1-like OS=Mus musculus OX=10090 GN=Aif1l PE=1 SV=1 - [AIF1L_MOUSE]	Aif1l	55.44457433	30.75203399	24.63644698	60.84973953	36.60644539	37.60350403	36.94435177	45.01989631	16.31084845	13.71810787	0.4	0.764125594	1.218586717	0.285208919
P16546	Spectrin alpha chain, non-erythrocytic 1 OS=Mus musculus OX=10090 GN=Sptan1 PE=1 SV=4 - [SPTN1_MOUSE]	Sptan1	197.1032216	142.2020273	138.8707143	193.6961769	244.7769875	145.7372776	159.3919877	194.736814	32.70133452	49.528055	0.4	0.764125594	1.221747823	0.288946534
P40240	CD9 antigen OS=Mus musculus OX=10090 GN=Cd9 PE=1 SV=2 - [CD9_MOUSE]	Cd9	428.2296692	545.2429155	940.0780682	598.142985	715.8350524	1028.097219	637.8502176	780.691752	268.1963463	222.1935066	0.4	0.764125594	1.223942127	0.291535343
P20029	Endoplasmic reticulum chaperone BiP OS=Mus musculus OX=10090 GN=Hspa5 PE=1 SV=3 - [BIP_MOUSE]	Hspa5	2882.652799	1667.476672	1546.346149	2904.427729	2686.779614	1872.53415	2032.15854	2487.913831	739.0355299	543.9317874	0.4	0.764125594	1.224271523	0.291923559
Q61001	Laminin subunit alpha-5 OS=Mus musculus OX=10090 GN=Lama5 PE=1 SV=4 - [LAMA5_MOUSE]	Lama5	50.55321867	50.97984932	38.95259618	43.15227439	58.13436905	70.75661788	46.82855472	57.34775377	6.824115012	13.8189731	0.4	0.764125594	1.224632153	0.292348466
P14211	Calreticulin OS=Mus musculus OX=10090 GN=Calr PE=1 SV=1 - [CALR_MOUSE]	Calr	123.8272521	77.88305027	102.3571771	147.1380623	137.8478484	88.05769319	101.3558265	124.347868	22.98846335	31.76963341	0.4	0.764125594	1.226844793	0.294952747
Q8R016	Bleomycin hydrolase OS=Mus musculus OX=10090 GN=Blmh PE=1 SV=1 - [BLMH_MOUSE]	Blmh	480.7735855	241.0355219	285.2198199	488.366368	508.2706229	241.7216902	335.6763091	412.7862271	127.5851785	148.4801389	0.4	0.764125594	1.229715103	0.298324115
Q8BIQ5	Cleavage stimulation factor subunit 2 OS=Mus musculus OX=10090 GN=Cstf2 PE=1 SV=2 - [CSTF2_MOUSE]	Cstf2	68.88845823	38.77826957	54.95937797	62.03786731	77.41735901	60.98768901	54.20870192	66.81430511	15.0691241	9.197515023	0.4	0.764125594	1.23253837	0.30163256
Q8VE43	Meteorin-like protein OS=Mus musculus OX=10090 GN=Metrnl PE=1 SV=1 - [METRL_MOUSE]	Metrnl	34.49601895	32.6519787	53.46534776	46.3392729	46.89767414	56.53433306	40.20444847	49.92376003	11.52122861	5.73172834	0.4	0.764125594	1.241747168	0.312371457
Q62179	Semaphorin-4B OS=Mus musculus OX=10090 GN=Sema4b PE=1 SV=2 - [SEM4B_MOUSE]	Sema4b	184.0190951	262.1161388	155.2024182	162.6611284	268.5117023	317.5141203	200.445884	249.562317	55.31740648	79.14652032	0.4	0.764125594	1.245035877	0.316187316
Q8BVE3	V-type proton ATPase subunit H OS=Mus musculus OX=10090 GN=Atp6v1h PE=1 SV=1 - [VATH_MOUSE]	Atp6v1h	36.77469263	19.46425223	22.51371267	26.39621038	38.04403387	33.65409452	26.25088584	32.69811292	9.240545668	5.882463225	0.4	0.764125594	1.245600363	0.316841269
P97820	Mitogen-activated protein kinase kinase kinase kinase 4 OS=Mus musculus OX=10090 GN=Map4k4 PE=1 SV=1 - [M4K4_MOUSE]	Map4k4	42.71452226	35.74479133	12.52373297	20.9253376	45.78962637	46.67190739	30.32768219	37.79562378	15.80758499	14.61675484	0.4	0.764125594	1.246241752	0.317583957
P10637	Microtubule-associated protein tau OS=Mus musculus OX=10090 GN=Mapt PE=1 SV=3 - [TAU_MOUSE]	Mapt	143.1809418	123.0831539	212.9878409	182.0288136	183.9191541	232.2328639	159.7506455	199.3936105	47.18717517	28.45532939	0.4	0.764125594	1.248155273	0.319797419
O70439	Syntaxin-7 OS=Mus musculus OX=10090 GN=Stx7 PE=1 SV=3 - [STX7_MOUSE]	Stx7	62.42976971	43.73714863	39.1826794	73.00848641	65.5483119	43.36343246	48.44986591	60.64007692	12.31925647	15.41996705	0.4	0.764125594	1.251604639	0.323778911
P61982	14-3-3 protein gamma OS=Mus musculus OX=10090 GN=Ywhag PE=1 SV=2 - [1433G_MOUSE]	Ywhag	6612.318447	3300.968091	3257.205211	6710.997423	5901.441817	3888.515177	4390.163917	5500.318139	1924.56667	1453.367352	0.4	0.764125594	1.252873069	0.32524026
Q9Z1D1	Eukaryotic translation initiation factor 3 subunit G OS=Mus musculus OX=10090 GN=Eif3g PE=1 SV=2 - [EIF3G_MOUSE]	Eif3g	157.746946	94.64347638	121.6305268	155.8478658	161.8096624	151.8981964	124.6736497	156.5185749	31.66160795	4.989657057	0.4	0.764125594	1.255426269	0.328177301
P29533	Vascular cell adhesion protein 1 OS=Mus musculus OX=10090 GN=Vcam1 PE=1 SV=1 - [VCAM1_MOUSE]	Vcam1	78.40813883	90.41722809	93.32407417	130.3614644	109.5765073	89.35370316	87.38314703	109.7638916	7.907307865	20.5045228	0.4	0.764125594	1.25612198	0.328976568
Q9CQV8	14-3-3 protein beta/alpha OS=Mus musculus OX=10090 GN=Ywhab PE=1 SV=3 - [1433B_MOUSE]	Ywhab	6612.318447	3282.998185	3247.324239	6710.997423	5925.420042	3888.515177	4380.88029	5508.31088	1932.564447	1456.738325	0.4	0.764125594	1.257352522	0.330389192
P47941	Crk-like protein OS=Mus musculus OX=10090 GN=Crkl PE=1 SV=2 - [CRKL_MOUSE]	Crkl	175.2722701	82.81597267	83.68584808	179.8715253	162.9590851	87.47214473	113.924697	143.4342517	53.13033712	49.19680601	0.4	0.764125594	1.259026844	0.332309044
Q99JF8	PC4 and SFRS1-interacting protein OS=Mus musculus OX=10090 GN=Psip1 PE=1 SV=1 - [PSIP1_MOUSE]	Psip1	107.2164075	73.81838065	77.10371728	106.1689112	129.6428188	89.24702831	86.04616846	108.3529194	18.40740675	20.28626112	0.4	0.764125594	1.259241653	0.332555168
P68510	14-3-3 protein eta OS=Mus musculus OX=10090 GN=Ywhah PE=1 SV=2 - [1433F_MOUSE]	Ywhah	6612.318447	3282.998185	3207.659813	6710.997423	5901.441817	3888.515177	4367.658815	5500.318139	1944.297204	1453.367352	0.4	0.764125594	1.259328709	0.332654904
P61226	Ras-related protein Rap-2b OS=Mus musculus OX=10090 GN=Rap2b PE=1 SV=1 - [RAP2B_MOUSE]	Rap2b	24.36991048	30.91316456	43.43305202	29.76471294	49.60475261	45.49156545	32.90537569	41.62034367	9.686460663	10.47122543	0.4	0.764125594	1.264849369	0.338965584
P68254	14-3-3 protein theta OS=Mus musculus OX=10090 GN=Ywhaq PE=1 SV=1 - [1433T_MOUSE]	Ywhaq	6612.318447	3282.998185	3265.183596	6710.997423	6034.571232	3916.540576	4386.833409	5554.03641	1927.347161	1457.886331	0.4	0.764125594	1.266069598	0.340356714
P12960	Contactin-1 OS=Mus musculus OX=10090 GN=Cntn1 PE=1 SV=1 - [CNTN1_MOUSE]	Cntn1	22.73032225	28.32775806	33.66917977	25.82982129	36.71801768	45.06059423	28.24242002	35.86947774	5.469928051	9.643426363	0.4	0.764125594	1.270056805	0.344893025
P63101	14-3-3 protein zeta/delta OS=Mus musculus OX=10090 GN=Ywhaz PE=1 SV=1 - [1433Z_MOUSE]	Ywhaz	7823.672654	4160.758355	4007.880048	8231.25834	7321.457106	4759.852648	5330.770352	6770.856031	2160.269511	1800.009869	0.4	0.764125594	1.270145886	0.344994211
Q99P72	Reticulon-4 OS=Mus musculus OX=10090 GN=Rtn4 PE=1 SV=2 - [RTN4_MOUSE]	Rtn4	207.0790759	170.9408499	154.3099968	239.5132041	285.8750373	154.6950573	177.4433075	226.6944329	26.97879526	66.52283409	0.4	0.764125594	1.27755978	0.3533908
Q9CPW4	Actin-related protein 2/3 complex subunit 5 OS=Mus musculus OX=10090 GN=Arpc5 PE=1 SV=3 - [ARPC5_MOUSE]	Arpc5	347.5581425	123.392003	150.1666067	357.0957745	260.0489002	176.6576296	207.0389174	264.6007681	122.4273634	90.30515301	0.4	0.764125594	1.278024303	0.353915271
O09010	Beta-1,3-N-acetylglucosaminyltransferase lunatic fringe OS=Mus musculus OX=10090 GN=Lfng PE=1 SV=1 - [LFNG_MOUSE]	Lfng	401.9919416	355.640171	432.4079338	381.8390651	642.7658386	502.2202108	396.6800154	508.9417048	38.65856656	130.5931818	0.4	0.764125594	1.283003139	0.3595247
P20152	Vimentin OS=Mus musculus OX=10090 GN=Vim PE=1 SV=3 - [VIME_MOUSE]	Vim	13095.32756	7416.613026	6956.987481	14402.15965	12827.11922	8051.940518	9156.309356	11760.40646	3419.022115	3306.770002	0.4	0.764125594	1.284404666	0.361099812
P63028	Translationally-controlled tumor protein OS=Mus musculus OX=10090 GN=Tpt1 PE=1 SV=1 - [TCTP_MOUSE]	Tpt1	1434.759347	794.93785	1160.35672	1734.096199	1571.051218	1079.510222	1130.017972	1461.552546	320.9878764	340.7538253	0.4	0.764125594	1.293388762	0.37115598
Q01279	Epidermal growth factor receptor OS=Mus musculus OX=10090 GN=Egfr PE=1 SV=1 - [EGFR_MOUSE]	Egfr	152.0306362	56.8762876	95.88650065	117.8294922	152.4692182	124.6340231	101.5978081	131.6442445	47.83358432	18.35306666	0.4	0.764125594	1.295739022	0.37377517
Q9JMG1	Endothelial differentiation-related factor 1 OS=Mus musculus OX=10090 GN=Edf1 PE=1 SV=1 - [EDF1_MOUSE]	Edf1	227.1374621	120.0708927	128.2176966	238.9487606	219.3832671	159.201233	158.4753505	205.8444203	59.60248978	41.56190951	0.4	0.764125594	1.29890497	0.377295884
Q9DAW9	Calponin-3 OS=Mus musculus OX=10090 GN=Cnn3 PE=1 SV=1 - [CNN3_MOUSE]	Cnn3	352.5264885	206.6746459	236.1612064	381.8989109	440.0235632	211.9447173	265.1207802	344.6223971	77.117979	118.5206473	0.4	0.764125594	1.299869429	0.378366712
P62259	14-3-3 protein epsilon OS=Mus musculus OX=10090 GN=Ywhae PE=1 SV=1 - [1433E_MOUSE]	Ywhae	7300.324299	3734.78327	3713.550229	7881.885719	6812.429897	4491.639636	4916.219266	6395.318417	2064.722819	1733.18453	0.4	0.764125594	1.300861103	0.379466929
O09061	Proteasome subunit beta type-1 OS=Mus musculus OX=10090 GN=Psmb1 PE=1 SV=1 - [PSB1_MOUSE]	Psmb1	494.6223167	287.7149221	216.1069333	517.4039701	488.9981199	292.6793131	332.814724	433.0271344	144.6312511	122.3717953	0.4	0.764125594	1.3011057	0.379738169
Q61206	Platelet-activating factor acetylhydrolase IB subunit alpha2 OS=Mus musculus OX=10090 GN=Pafah1b2 PE=1 SV=2 - [PA1B2_MOUSE]	Pafah1b2	305.7205409	157.9936188	192.1870434	286.1780409	317.7451503	250.0109436	218.6337344	284.6447116	77.33292623	33.89312636	0.4	0.764125594	1.301924941	0.380646276
Q8BGR9	Ubiquitin-like domain-containing CTD phosphatase 1 OS=Mus musculus OX=10090 GN=Ublcp1 PE=1 SV=1 - [UBCP1_MOUSE]	Ublcp1	44.73463963	19.0382623	23.80382676	46.30990493	48.65059641	19.0946711	29.1922429	38.01839081	13.66939001	16.43015779	0.4	0.764125594	1.302345659	0.381112409
P97371	Proteasome activator complex subunit 1 OS=Mus musculus OX=10090 GN=Psme1 PE=1 SV=2 - [PSME1_MOUSE]	Psme1	123.993676	73.4772118	72.76610981	158.7824245	109.2922855	84.1162465	90.07899919	117.3969855	29.37312363	37.98715779	0.4	0.764125594	1.303266983	0.38213266
Q8BM13	Noelin-2 OS=Mus musculus OX=10090 GN=Olfm2 PE=1 SV=2 - [NOE2_MOUSE]	Olfm2	154.1717006	115.4926744	149.6332855	129.0354579	194.7830741	223.0775943	139.7658868	182.2987087	21.14334254	48.24806096	0.4	0.764125594	1.304314757	0.383292063
P15532	Nucleoside diphosphate kinase A OS=Mus musculus OX=10090 GN=Nme1 PE=1 SV=1 - [NDKA_MOUSE]	Nme1	3781.233249	1721.451595	1878.416903	3142.964693	4174.551803	2315.710293	2460.367249	3211.075596	1146.592678	931.2906427	0.4	0.764125594	1.305120444	0.384182953
P53996	Cellular nucleic acid-binding protein OS=Mus musculus OX=10090 GN=Cnbp PE=1 SV=2 - [CNBP_MOUSE]	Cnbp	406.5370527	161.9358171	158.4383112	410.6483944	311.5217025	227.0404468	242.303727	316.4035146	142.2409824	91.90127134	0.4	0.764125594	1.305813652	0.384949029
P0C192	Leucine-rich repeat-containing protein 4B OS=Mus musculus OX=10090 GN=Lrrc4b PE=1 SV=1 - [LRC4B_MOUSE]	Lrrc4b	155.8592956	102.1349266	111.0016397	130.2996092	205.6088879	151.8227284	122.9986206	162.5770752	28.80143438	38.78935218	0.4	0.764125594	1.321779662	0.402481702
Q9CQW1	Synaptobrevin homolog YKT6 OS=Mus musculus OX=10090 GN=Ykt6 PE=1 SV=1 - [YKT6_MOUSE]	Ykt6	107.757952	40.55834965	46.39264539	86.58341184	110.4278933	60.79639221	64.90298233	85.93589911	37.22796084	24.82208552	0.4	0.764125594	1.324067031	0.40497616
Q8QZY1	Eukaryotic translation initiation factor 3 subunit L OS=Mus musculus OX=10090 GN=Eif3l PE=1 SV=1 - [EIF3L_MOUSE]	Eif3l	106.9688284	44.00630956	47.76147417	74.69261798	118.056945	71.20120065	66.24553737	87.98358787	35.31734897	26.10273175	0.4	0.764125594	1.328143621	0.409411163
P53810	Phosphatidylinositol transfer protein alpha isoform OS=Mus musculus OX=10090 GN=Pitpna PE=1 SV=2 - [PIPNA_MOUSE]	Pitpna	219.4074491	104.376227	74.48940236	195.3646559	224.8437477	112.431722	132.7576928	177.5467085	76.51431744	58.28572139	0.4	0.764125594	1.337374165	0.419403153
P63089	Pleiotrophin OS=Mus musculus OX=10090 GN=Ptn PE=1 SV=1 - [PTN_MOUSE]	Ptn	667.451985	308.9004821	369.5431251	495.4671245	671.9387891	638.2928578	448.6318641	601.8995905	191.9142166	93.69586184	0.4	0.764125594	1.341633617	0.423990744
Q9DBJ1	Phosphoglycerate mutase 1 OS=Mus musculus OX=10090 GN=Pgam1 PE=1 SV=3 - [PGAM1_MOUSE]	Pgam1	2897.087319	1169.268253	1299.898759	2486.943906	3056.723117	1671.84028	1788.751444	2405.169101	962.0667294	696.053487	0.4	0.764125594	1.344607776	0.427185398
Q9JM76	Actin-related protein 2/3 complex subunit 3 OS=Mus musculus OX=10090 GN=Arpc3 PE=1 SV=3 - [ARPC3_MOUSE]	Arpc3	539.9615109	235.8719985	248.0193685	505.8941136	546.0161098	324.8755353	341.2842926	458.9285862	172.166685	117.8138744	0.4	0.764125594	1.344710542	0.427295657
Q99JY9	Actin-related protein 3 OS=Mus musculus OX=10090 GN=Actr3 PE=1 SV=3 - [ARP3_MOUSE]	Actr3	486.070941	190.013732	231.2259715	496.6100477	436.5144599	290.9514353	302.4368815	408.0253143	160.3611955	105.7477649	0.4	0.764125594	1.349125518	0.432024578
P0CG49	Polyubiquitin-B OS=Mus musculus OX=10090 GN=Ubb PE=2 SV=1 - [UBB_MOUSE]	Ubb	2931.948393	2084.489764	1197.65967	3039.888266	2784.909639	2563.727605	2071.365942	2796.17517	867.2188418	238.2801459	0.4	0.764125594	1.349918483	0.43287229
Q9CY58	Plasminogen activator inhibitor 1 RNA-binding protein OS=Mus musculus OX=10090 GN=Serbp1 PE=1 SV=2 - [PAIRB_MOUSE]	Serbp1	235.1760115	83.9486434	126.4873094	238.7826866	212.4906356	151.4220421	148.5373214	200.8984548	77.98770088	44.81913144	0.4	0.764125594	1.35251163	0.435641
P60766	Cell division control protein 42 homolog OS=Mus musculus OX=10090 GN=Cdc42 PE=1 SV=2 - [CDC42_MOUSE]	Cdc42	265.8131804	129.3546225	101.2577429	235.2858167	267.9608564	171.4232068	165.4751819	224.88996	88.02354294	49.10127108	0.4	0.764125594	1.359055523	0.442604398
Q9WUM4	Coronin-1C OS=Mus musculus OX=10090 GN=Coro1c PE=1 SV=2 - [COR1C_MOUSE]	Coro1c	437.9022708	205.2013888	226.6478782	436.6369433	469.9592926	275.5237908	289.9171793	394.0400089	128.6066813	103.9815619	0.4	0.764125594	1.359146808	0.442701297
Q01768	Nucleoside diphosphate kinase B OS=Mus musculus OX=10090 GN=Nme2 PE=1 SV=1 - [NDKB_MOUSE]	Nme2	3781.233249	1721.451595	1878.416903	3581.374399	4174.551803	2315.710293	2460.367249	3357.212165	1146.592678	949.4785253	0.4	0.764125594	1.364516686	0.448390037
Q9R0Q6	Actin-related protein 2/3 complex subunit 1A OS=Mus musculus OX=10090 GN=Arpc1a PE=1 SV=1 - [ARC1A_MOUSE]	Arpc1a	256.5003031	94.47513471	100.1637307	202.6455667	281.3210354	132.8753928	150.3797228	205.6139983	91.94712177	74.26732724	0.4	0.764125594	1.367298692	0.45132844
P17047	Lysosome-associated membrane glycoprotein 2 OS=Mus musculus OX=10090 GN=Lamp2 PE=1 SV=2 - [LAMP2_MOUSE]	Lamp2	43.94186562	92.20237845	67.38641449	111.8441077	107.5723324	59.13426055	67.84355285	92.85023353	24.13350381	29.27690458	0.4	0.764125594	1.368593324	0.452693815
P21956	Lactadherin OS=Mus musculus OX=10090 GN=Mfge8 PE=1 SV=3 - [MFGM_MOUSE]	Mfge8	275.6543423	150.5315477	203.8782753	190.350995	369.7118819	308.0803454	210.0213885	289.3810741	62.78719464	91.13083447	0.4	0.764125594	1.37786478	0.462434312
P34884	Macrophage migration inhibitory factor OS=Mus musculus OX=10090 GN=Mif PE=1 SV=2 - [MIF_MOUSE]	Mif	1801.249802	824.1287668	833.2016274	1802.90115	1730.716304	1235.038041	1152.860066	1589.551832	561.5403079	309.1321461	0.4	0.764125594	1.378789915	0.463402652
P99024	Tubulin beta-5 chain OS=Mus musculus OX=10090 GN=Tubb5 PE=1 SV=1 - [TBB5_MOUSE]	Tubb5	1157.243141	327.2509037	468.045673	759.1018221	1286.724856	658.1621413	650.8465725	901.3296065	444.1665285	337.5564081	0.4	0.764125594	1.384857268	0.469737291
Q9CWF2	Tubulin beta-2B chain OS=Mus musculus OX=10090 GN=Tubb2b PE=1 SV=1 - [TBB2B_MOUSE]	Tubb2b	1157.243141	327.2509037	468.045673	759.1018221	1286.724856	658.1621413	650.8465725	901.3296065	444.1665285	337.5564081	0.4	0.764125594	1.384857268	0.469737291
P35585	AP-1 complex subunit mu-1 OS=Mus musculus OX=10090 GN=Ap1m1 PE=1 SV=3 - [AP1M1_MOUSE]	Ap1m1	62.27858262	30.81515511	23.96804487	65.51892668	67.25169796	29.38936535	39.0205942	54.05333	20.43088957	21.37718382	0.4	0.764125594	1.38525133	0.470147752
P63017	Heat shock cognate 71 kDa protein OS=Mus musculus OX=10090 GN=Hspa8 PE=1 SV=1 - [HSP7C_MOUSE]	Hspa8	4670.615728	2116.645499	2173.290968	4639.046421	4745.253119	3041.004394	2986.850731	4141.767978	1458.458295	954.7671527	0.4	0.764125594	1.386667212	0.471621596
P62305	Small nuclear ribonucleoprotein E OS=Mus musculus OX=10090 GN=Snrpe PE=1 SV=1 - [RUXE_MOUSE]	Snrpe	206.4321078	92.84752817	75.35146809	147.3065147	252.6605427	122.8214329	124.8770347	174.2628301	71.16846557	68.98935289	0.4	0.764125594	1.395475401	0.480756694
P25444	40S ribosomal protein S2 OS=Mus musculus OX=10090 GN=Rps2 PE=1 SV=3 - [RS2_MOUSE]	Rps2	119.6458347	30.41211477	35.56363536	81.13053051	127.7901197	52.32864382	61.8738616	87.08309802	50.09825541	38.08127337	0.4	0.764125594	1.407429499	0.493062656
P63242	Eukaryotic translation initiation factor 5A-1 OS=Mus musculus OX=10090 GN=Eif5a PE=1 SV=2 - [IF5A1_MOUSE]	Eif5a	1050.965778	403.1962484	481.5514107	1112.936067	1003.514502	613.5919593	645.2378123	910.014176	353.5481147	262.4745086	0.4	0.764125594	1.410354692	0.496058034
P10639	Thioredoxin OS=Mus musculus OX=10090 GN=Txn PE=1 SV=3 - [THIO_MOUSE]	Txn	2198.658979	1092.918158	1400.047545	2160.573599	2674.175572	1785.772995	1563.874894	2206.840722	570.7847589	446.0047865	0.4	0.764125594	1.411136358	0.496857402
P56376	Acylphosphatase-1 OS=Mus musculus OX=10090 GN=Acyp1 PE=1 SV=2 - [ACYP1_MOUSE]	Acyp1	104.4862086	35.26439586	52.9718345	89.57807392	110.7848419	72.44295387	64.24081298	90.93528988	35.96049563	19.20694197	0.4	0.764125594	1.415537657	0.501350129
Q9ER58	Testican-2 OS=Mus musculus OX=10090 GN=Spock2 PE=1 SV=1 - [TICN2_MOUSE]	Spock2	23.14500359	14.26006498	10.37951814	14.11121324	28.34886316	25.31930878	15.92819557	22.59312839	6.544188221	7.500114225	0.4	0.764125594	1.418436149	0.504301209
Q9R1P3	Proteasome subunit beta type-2 OS=Mus musculus OX=10090 GN=Psmb2 PE=1 SV=1 - [PSB2_MOUSE]	Psmb2	490.9789564	223.6355884	144.9528535	475.1915804	502.9466194	244.889068	286.5224661	407.675756	181.3824289	141.6587974	0.4	0.764125594	1.422840455	0.508773899
O35464	Semaphorin-6A OS=Mus musculus OX=10090 GN=Sema6a PE=1 SV=2 - [SEM6A_MOUSE]	Sema6a	13.4808549	19.30862977	28.67533454	14.05395685	42.00174823	31.66401657	20.48827307	29.23990722	7.665619457	14.1307109	0.4	0.764125594	1.427153334	0.513140347
Q9WU28	Prefoldin subunit 5 OS=Mus musculus OX=10090 GN=Pfdn5 PE=1 SV=1 - [PFD5_MOUSE]	Pfdn5	122.4165128	43.27464636	55.18597001	128.1416148	118.2009139	69.63763221	73.62570972	105.3267203	42.67173253	31.30475427	0.4	0.764125594	1.430569847	0.516589938
Q9QUM9	Proteasome subunit alpha type-6 OS=Mus musculus OX=10090 GN=Psma6 PE=1 SV=1 - [PSA6_MOUSE]	Psma6	528.7535689	231.3340076	188.0772461	577.1501276	523.5883353	256.1126741	316.0549409	452.2837123	185.4678318	171.9869867	0.4	0.764125594	1.431028767	0.517052674
Q9CVB6	Actin-related protein 2/3 complex subunit 2 OS=Mus musculus OX=10090 GN=Arpc2 PE=1 SV=3 - [ARPC2_MOUSE]	Arpc2	260.3006152	106.2212713	96.61997334	268.8328337	259.2144208	135.61117	154.38062	221.2194748	91.8549415	74.2947835	0.4	0.764125594	1.432948481	0.518986741
Q91YP2	Neurolysin, mitochondrial OS=Mus musculus OX=10090 GN=Nln PE=1 SV=1 - [NEUL_MOUSE]	Nln	93.58246191	32.5175767	20.24763608	74.84019862	94.47124557	40.95835477	48.78255823	70.08993299	39.27991074	27.07085403	0.4	0.764125594	1.436782644	0.522841828
P14152	Malate dehydrogenase, cytoplasmic OS=Mus musculus OX=10090 GN=Mdh1 PE=1 SV=3 - [MDHC_MOUSE]	Mdh1	2423.46958	791.2760669	820.719009	2515.182646	2081.271269	1202.184159	1345.154885	1932.879358	933.9639489	668.9591737	0.4	0.764125594	1.436919554	0.522979295
Q9Z0L0	Trophoblast glycoprotein OS=Mus musculus OX=10090 GN=Tpbg PE=1 SV=3 - [TPBG_MOUSE]	Tpbg	43.38856458	19.38593619	28.98529037	42.21117941	50.47642523	39.46044562	30.58659705	44.04935009	12.08117046	5.733420034	0.4	0.764125594	1.440152038	0.526221127

Q99KJ8	Dynactin subunit 2 OS=Mus musculus OX=10090 GN=Dctn2 PE=1 SV=3 - [DCTN2_MOUSE]	Dctn2	95.76527343	51.79905048	22.58407962	93.06062538	99.21307591	52.92438348	56.71613451	81.73269492	36.8375497	25.13766632	0.4	0.764125594	1.441083664	0.527154095
P23492	Purine nucleoside phosphorylase OS=Mus musculus OX=10090 GN=Pnp PE=1 SV=2 - [PNPH_MOUSE]	Pnp	227.1305996	87.35116353	123.3189901	258.7869465	255.4587669	118.1749799	145.9335844	210.8068978	72.5819371	80.2388519	0.4	0.764125594	1.444539985	0.530610138
Q9JMH6	Thioredoxin reductase 1, cytoplasmic OS=Mus musculus OX=10090 GN=Txnrd1 PE=1 SV=3 - [TRXR1_MOUSE]	Txnrd1	431.6084576	216.5552523	239.6680634	525.7936037	430.6619305	326.4236038	295.9439244	427.6263793	118.0559176	99.71965777	0.4	0.764125594	1.444957453	0.531027013
Q04447	Creatine kinase B-type OS=Mus musculus OX=10090 GN=Ckb PE=1 SV=1 - [KCRB_MOUSE]	Ckb	20243.68374	10947.56193	13641.74795	23384.86097	27949.47759	13483.20649	14944.3312	21605.84835	4782.992004	7395.398185	0.4	0.764125594	1.445755454	0.531823545
P80313	T-complex protein 1 subunit eta OS=Mus musculus OX=10090 GN=Cct7 PE=1 SV=1 - [TCPH_MOUSE]	Cct7	342.2856571	104.8501335	137.8985746	370.3130936	308.6985026	168.8207351	195.0114551	282.6107771	128.6091669	103.2483399	0.4	0.764125594	1.449200904	0.535257611
Q8BH69	Selenide, water dikinase 1 OS=Mus musculus OX=10090 GN=Sephs1 PE=1 SV=1 - [SPS1_MOUSE]	Sephs1	191.6754512	58.7440033	61.14485011	170.0637576	191.9409979	91.22293505	103.8547682	151.0758968	76.06441537	52.97580756	0.4	0.764125594	1.454684262	0.540706051
Q64727	Vinculin OS=Mus musculus OX=10090 GN=Vcl PE=1 SV=4 - [VINC_MOUSE]	Vcl	292.7302627	135.3072556	105.7425517	263.6433515	335.614724	180.7304468	177.92669	259.9961741	100.5157359	77.50652401	0.4	0.764125594	1.461254487	0.547207454
Q61316	Heat shock 70 kDa protein 4 OS=Mus musculus OX=10090 GN=Hspa4 PE=1 SV=1 - [HSP74_MOUSE]	Hspa4	552.7324569	226.3997086	167.2268778	575.6308666	520.6577439	286.9898228	315.4530144	461.0928111	207.6090228	153.2625165	0.4	0.764125594	1.461684593	0.547632036
P70202	Latexin OS=Mus musculus OX=10090 GN=Lxn PE=1 SV=2 - [LXN_MOUSE]	Lxn	547.3726417	319.5671198	192.9535753	358.4719328	700.8428655	492.2057133	353.2977789	517.1735039	179.6010487	172.5456659	0.4	0.764125594	1.463845896	0.549763685
P45591	Cofilin-2 OS=Mus musculus OX=10090 GN=Cfl2 PE=1 SV=1 - [COF2_MOUSE]	Cfl2	2785.803637	1028.443345	1168.287478	2993.308857	2746.006923	1593.350007	1660.84482	2444.221929	976.7488675	747.1792507	0.4	0.764125594	1.471673873	0.557458002
Q61879	Myosin-10 OS=Mus musculus OX=10090 GN=Myh10 PE=1 SV=2 - [MYH10_MOUSE]	Myh10	128.6579686	64.2242886	68.75551045	168.2998218	125.9230419	91.14062641	87.21258921	128.4544967	35.96418492	38.64183684	0.4	0.764125594	1.472889383	0.558649085
Q8BVI4	Dihydropteridine reductase OS=Mus musculus OX=10090 GN=Qdpr PE=1 SV=2 - [DHPR_MOUSE]	Qdpr	221.2642159	86.91028158	82.69137694	219.8767475	236.4752143	121.4098979	130.2886248	192.5872866	78.81540722	62.1976106	0.4	0.764125594	1.478158871	0.563801338
Q9JLV1	BAG family molecular chaperone regulator 3 OS=Mus musculus OX=10090 GN=Bag3 PE=1 SV=2 - [BAG3_MOUSE]	Bag3	68.46373929	30.68211772	61.65861721	88.75371344	91.6720058	57.41371327	53.60149141	79.27981084	20.13828913	18.99272952	0.4	0.764125594	1.479059794	0.564680378
P00920	Carbonic anhydrase 2 OS=Mus musculus OX=10090 GN=Ca2 PE=1 SV=4 - [CAH2_MOUSE]	Ca2	331.9354705	83.14257371	161.1438861	346.9984321	370.2697907	135.7164612	192.0739768	284.328228	127.2477083	129.2264754	0.4	0.764125594	1.480305832	0.565895268
Q7TSV4	Phosphoglucomutase-2 OS=Mus musculus OX=10090 GN=Pgm2 PE=1 SV=1 - [PGM2_MOUSE]	Pgm2	151.1016902	66.06193785	63.55906952	179.6866511	173.3190205	63.65664416	93.57423254	138.8874386	49.83595469	65.22952553	0.4	0.764125594	1.48424876	0.569732907
P16125	L-lactate dehydrogenase B chain OS=Mus musculus OX=10090 GN=Ldhb PE=1 SV=2 - [LDHB_MOUSE]	Ldhb	2405.8066	910.1673997	1045.394568	2351.981728	2565.160628	1561.088767	1453.789523	2159.410374	827.2387708	529.0112193	0.4	0.764125594	1.485366582	0.570819026
Q8BK64	Activator of 90 kDa heat shock protein ATPase homolog 1 OS=Mus musculus OX=10090 GN=Ahsa1 PE=1 SV=2 - [AHSA1_MOUSE]	Ahsa1	176.5186203	61.77110021	61.63023995	168.4226673	189.9473553	88.72305391	99.97332014	149.0310255	66.29021186	53.32557198	0.4	0.764125594	1.490707974	0.575997665
Q9R1T2	SUMO-activating enzyme subunit 1 OS=Mus musculus OX=10090 GN=Sae1 PE=1 SV=1 - [SAE1_MOUSE]	Sae1	186.3553126	53.17996103	58.49561117	167.289039	189.4056221	90.98156842	99.34362828	149.2254099	75.40118663	51.63859614	0.4	0.764125594	1.502113547	0.586993872
Q9CYN9	Renin receptor OS=Mus musculus OX=10090 GN=Atp6ap2 PE=1 SV=2 - [RENR_MOUSE]	Atp6ap2	61.96777987	51.36970162	66.3384255	55.02584495	124.9168848	90.90456397	59.89196899	90.28243122	7.697234355	34.94967308	0.4	0.764125594	1.507421324	0.592082707
Q60973	Histone-binding protein RBBP7 OS=Mus musculus OX=10090 GN=Rbbp7 PE=1 SV=1 - [RBBP7_MOUSE]	Rbbp7	477.4012004	174.1304696	162.7012406	459.8509072	496.1369705	277.5115513	271.4109702	411.1664763	178.4842795	117.1618489	0.4	0.764125594	1.514922098	0.599243608
P42932	T-complex protein 1 subunit theta OS=Mus musculus OX=10090 GN=Cct8 PE=1 SV=3 - [TCPQ_MOUSE]	Cct8	422.2924251	134.0853199	132.5276207	461.8422441	379.0725077	206.5237884	229.6351219	349.1461801	166.8479367	130.2634533	0.4	0.764125594	1.520438935	0.604487875
Q7TMB8	Cytoplasmic FMR1-interacting protein 1 OS=Mus musculus OX=10090 GN=Cyfip1 PE=1 SV=1 - [CYFP1_MOUSE]	Cyfip1	46.62679581	10.99869245	11.30808455	21.6584229	62.51768986	21.23500754	22.97785761	35.1370401	20.48116549	23.71328333	0.4	0.764125594	1.529169547	0.612748374
Q9JKF1	Ras GTPase-activating-like protein IQGAP1 OS=Mus musculus OX=10090 GN=Iqgap1 PE=1 SV=2 - [IQGA1_MOUSE]	Iqgap1	140.9539649	31.41980398	55.12118031	147.8587159	133.7798518	66.81390371	75.83164974	116.1508238	57.62920928	43.30302953	0.4	0.764125594	1.53169322	0.615127372
Q9CXW3	Calcyclin-binding protein OS=Mus musculus OX=10090 GN=Cacybp PE=1 SV=1 - [CYBP_MOUSE]	Cacybp	102.7884659	39.98337274	44.60479578	86.00247761	133.9242781	67.27230426	62.45887815	95.73302	35.00280168	34.37490358	0.4	0.764125594	1.532736784	0.616109965
P35278	Ras-related protein Rab-5C OS=Mus musculus OX=10090 GN=Rab5c PE=1 SV=2 - [RAB5C_MOUSE]	Rab5c	91.59301632	35.45898386	25.29358114	55.8894939	99.34883493	79.17706984	50.78186044	78.13846622	35.70709574	21.74827818	0.4	0.764125594	1.538708223	0.621719687
Q9CZU6	Citrate synthase, mitochondrial OS=Mus musculus OX=10090 GN=Cs PE=1 SV=1 - [CISY_MOUSE]	Cs	301.3360268	73.81858815	78.45457873	317.8324039	271.7457491	114.0620687	151.2030646	234.5467406	130.0396204	106.8569701	0.4	0.764125594	1.551203617	0.633388072
Q99020	Heterogeneous nuclear ribonucleoprotein A/B OS=Mus musculus OX=10090 GN=Hnrnpab PE=1 SV=1 - [ROAA_MOUSE]	Hnrnpab	1467.07385	501.692062	496.634773	1504.480045	1461.936112	866.3406883	821.8002282	1277.585615	558.8290695	356.783252	0.4	0.764125594	1.554618229	0.636560338
Q9Z0S1	3'(2'),5'-bisphosphate nucleotidase 1 OS=Mus musculus OX=10090 GN=Bpnt1 PE=1 SV=2 - [BPNT1_MOUSE]	Bpnt1	224.6426487	123.1555724	0	228.3096851	224.7865959	87.94183726	115.9327404	180.3460394	112.4953638	80.04377221	0.4	0.764125594	1.555609216	0.637479687
Q9QZ88	Vacuolar protein sorting-associated protein 29 OS=Mus musculus OX=10090 GN=Vps29 PE=1 SV=1 - [VPS29_MOUSE]	Vps29	147.207652	43.20533604	55.62358602	200.8983718	93.48466772	90.79712667	82.01219136	128.3933887	56.80131425	62.80553436	0.4	0.764125594	1.565540276	0.646660625
P05063	Fructose-bisphosphate aldolase C OS=Mus musculus OX=10090 GN=Aldoc PE=1 SV=4 - [ALDOC_MOUSE]	Aldoc	2296.064294	775.249432	777.9021869	2581.194864	2153.051381	1301.08382	1283.071971	2011.776689	877.278088	651.6440558	0.4	0.764125594	1.567937524	0.648868075
P02798	Metallothionein-2 OS=Mus musculus OX=10090 GN=Mt2 PE=1 SV=2 - [MT2_MOUSE]	Mt2	12.09627208	4.466197511	0	13.25532441	8.075592794	4.647274602	5.520823196	8.65939727	6.116708759	4.333618712	0.4	0.764125594	1.56849748	0.649383211
Q8VEE0	Ribulose-phosphate 3-epimerase OS=Mus musculus OX=10090 GN=Rpe PE=1 SV=1 - [RPE_MOUSE]	Rpe	56.52256148	16.98527069	16.16577519	56.63053433	51.18679901	32.93388461	29.89120245	46.91707265	23.06707298	12.41191864	0.4	0.764125594	1.56959469	0.650392066
P49722	Proteasome subunit alpha type-2 OS=Mus musculus OX=10090 GN=Psma2 PE=1 SV=3 - [PSA2_MOUSE]	Psma2	318.8089297	164.2276145	87.42098322	369.6617255	366.051497	160.0322176	190.1525091	298.5818134	117.8523252	120.0010471	0.4	0.764125594	1.570222843	0.650969318
P54726	UV excision repair protein RAD23 homolog A OS=Mus musculus OX=10090 GN=Rad23a PE=1 SV=2 - [RD23A_MOUSE]	Rad23a	173.699622	22.60264171	78.54314902	181.6202173	159.7946886	90.36692268	91.61513759	143.9272762	76.39196313	47.65104435	0.4	0.764125594	1.570998854	0.651682129
P62082	40S ribosomal protein S7 OS=Mus musculus OX=10090 GN=Rps7 PE=2 SV=1 - [RS7_MOUSE]	Rps7	166.9667066	19.56950339	159.2523621	212.7110633	259.0863346	72.07445359	115.2628574	181.2906172	82.96258961	97.38477415	0.4	0.764125594	1.572845072	0.65337657
Q9DBZ5	Eukaryotic translation initiation factor 3 subunit K OS=Mus musculus OX=10090 GN=Eif3k PE=1 SV=1 - [EIF3K_MOUSE]	Eif3k	0	58.01147621	67.62780101	59.65926387	62.50072951	75.45932349	41.87975907	65.87310562	36.58625549	8.422598261	0.4	0.764125594	1.572910329	0.653436426
O70435	Proteasome subunit alpha type-3 OS=Mus musculus OX=10090 GN=Psma3 PE=1 SV=3 - [PSA3_MOUSE]	Psma3	457.4582022	287.9465372	72.56834494	538.7866662	475.0199979	273.5521321	272.6576948	429.1195987	192.8998758	138.4466486	0.4	0.764125594	1.573840045	0.654288922
O08576	RUN domain-containing protein 3A OS=Mus musculus OX=10090 GN=Rundc3a PE=1 SV=1 - [RUN3A_MOUSE]	Rundc3a	687.2785595	184.9832329	224.3014851	648.220268	692.8608302	392.4208398	365.5210925	577.8339793	279.3427678	162.1163754	0.4	0.764125594	1.580849891	0.660700384
Q9R0E1	Multifunctional procollagen lysine hydroxylase and glycosyltransferase LH3 OS=Mus musculus OX=10090 GN=Plod3 PE=1 SV=1 - [PLOD3_MOUSE]	Plod3	80.53625101	155.2917057	150.8134579	137.4063152	206.5132705	267.8484683	128.8804716	203.9226847	41.92715614	65.25965201	0.4	0.764125594	1.582262093	0.661988594
Q9QZF2	Glypican-1 OS=Mus musculus OX=10090 GN=Gpc1 PE=1 SV=1 - [GPC1_MOUSE]	Gpc1	99.13568472	48.3900872	46.11892805	97.96576487	95.93528825	113.6935069	64.54823332	102.53152	29.97512946	9.719731078	0.4	0.764125594	1.588448122	0.667617973
Q8BPB0	MOB kinase activator 1B OS=Mus musculus OX=10090 GN=Mob1b PE=1 SV=3 - [MOB1B_MOUSE]	Mob1b	69.93415482	33.41533623	34.32606761	68.33515341	91.41964932	60.33639082	45.89185289	73.36373118	20.82622312	16.14023499	0.4	0.764125594	1.598622121	0.676828958
Q9DCL9	Multifunctional protein ADE2 OS=Mus musculus OX=10090 GN=Paics PE=1 SV=4 - [PUR6_MOUSE]	Paics	327.3917695	78.37922688	104.1971591	311.6727082	371.0478484	135.7991303	169.9893851	272.8398956	136.9243381	122.3375671	0.4	0.764125594	1.605040782	0.682609955
Q9D0W5	Peptidyl-prolyl cis-trans isomerase-like 1 OS=Mus musculus OX=10090 GN=Ppil1 PE=1 SV=1 - [PPIL1_MOUSE]	Ppil1	161.8608072	44.20975735	46.38013109	124.454067	167.7604017	114.999689	84.15023188	135.7380526	67.30808099	28.13217817	0.4	0.764125594	1.613044308	0.689786068
O89017	Legumain OS=Mus musculus OX=10090 GN=Lgmn PE=1 SV=1 - [LGMN_MOUSE]	Lgmn	116.7730552	57.85949344	131.3125738	110.0960508	214.2479173	169.9192691	101.9817075	164.7544124	38.89635882	52.26767296	0.4	0.764125594	1.615529064	0.692006705
P47791	Glutathione reductase, mitochondrial OS=Mus musculus OX=10090 GN=Gsr PE=1 SV=3 - [GSHR_MOUSE]	Gsr	279.9699819	78.64759817	57.4578252	277.2691465	288.8129576	106.5805691	138.6918018	224.2208911	122.808366	102.0428778	0.4	0.764125594	1.616684535	0.693038192
P17751	Triosephosphate isomerase OS=Mus musculus OX=10090 GN=Tpi1 PE=1 SV=5 - [TPIS_MOUSE]	Tpi1	1524.294378	361.9862154	444.4771994	1680.500548	1416.54697	698.4146714	776.9192644	1265.154063	648.55868	508.2450455	0.4	0.764125594	1.628424112	0.703476488
Q9CQM9	Glutaredoxin-3 OS=Mus musculus OX=10090 GN=Glrx3 PE=1 SV=1 - [GLRX3_MOUSE]	Glrx3	348.7926817	105.5048479	121.5706709	393.3300099	308.0077823	236.7249398	191.9560668	312.6875773	136.061826	78.40734886	0.4	0.764125594	1.628953867	0.703945746
Q64213	Splicing factor 1 OS=Mus musculus OX=10090 GN=Sf1 PE=1 SV=6 - [SF01_MOUSE]	Sf1	233.615543	63.64524504	62.78774561	218.1351798	282.0539178	86.4621718	120.0161779	195.5504231	98.38087028	99.73257343	0.4	0.764125594	1.629367195	0.704311767
O35643	AP-1 complex subunit beta-1 OS=Mus musculus OX=10090 GN=Ap1b1 PE=1 SV=2 - [AP1B1_MOUSE]	Ap1b1	247.1006325	102.1207022	83.28518193	241.4061869	314.7433567	151.5508835	144.1688389	235.9001424	89.63765844	81.73544656	0.4	0.764125594	1.636276911	0.71041692
Q9R0P5	Destrin OS=Mus musculus OX=10090 GN=Dstn PE=1 SV=3 - [DEST_MOUSE]	Dstn	298.6385515	70.24507996	95.43306969	352.8451431	269.3733594	137.8797505	154.7722337	253.3660844	125.2267813	108.3729885	0.4	0.764125594	1.637025442	0.711076744
Q64010	Adapter molecule crk OS=Mus musculus OX=10090 GN=Crk PE=1 SV=1 - [CRK_MOUSE]	Crk	123.4092488	50.30770102	48.69690086	122.7559092	184.9467154	58.35661278	74.13795024	122.0197458	42.67779655	63.29826198	0.4	0.764125594	1.645847307	0.718830497
P26443	Glutamate dehydrogenase 1, mitochondrial OS=Mus musculus OX=10090 GN=Glud1 PE=1 SV=1 - [DHE3_MOUSE]	Glud1	57.67150024	102.0478256	65.29479736	84.10083231	213.6334838	73.19409405	75.00470773	123.6428034	23.72817591	78.1247794	0.4	0.764125594	1.648467238	0.721125215
Q9Z1N5	Spliceosome RNA helicase Ddx39b OS=Mus musculus OX=10090 GN=Ddx39b PE=1 SV=1 - [DX39B_MOUSE]	Ddx39b	541.2636727	128.4763649	136.9064419	508.7659521	561.3007989	260.2942979	268.8821598	443.453683	235.9269653	160.7808999	0.4	0.764125594	1.649249185	0.721809392
Q9D8N0	Elongation factor 1-gamma OS=Mus musculus OX=10090 GN=Eef1g PE=1 SV=3 - [EF1G_MOUSE]	Eef1g	441.6908677	105.1526857	118.8641669	389.2100126	505.9788215	213.0079022	221.9025734	369.3989121	190.4656712	147.486778	0.4	0.764125594	1.664689627	0.735253219
P47964	60S ribosomal protein L36 OS=Mus musculus OX=10090 GN=Rpl36 PE=1 SV=2 - [RL36_MOUSE]	Rpl36	101.0478205	0	33.68260685	77.69808009	110.1179714	37.16280181	44.91014246	74.9929511	51.45103183	36.55273566	0.4	0.764125594	1.669844427	0.739713698
P80318	T-complex protein 1 subunit gamma OS=Mus musculus OX=10090 GN=Cct3 PE=1 SV=1 - [TCPG_MOUSE]	Cct3	376.3741301	90.36539546	101.6385085	407.7591855	356.4615711	186.0039869	189.4593447	316.7415812	161.9710577	116.0909136	0.4	0.764125594	1.671818203	0.741417974
Q922B2	Aspartate--tRNA ligase, cytoplasmic OS=Mus musculus OX=10090 GN=Dars1 PE=1 SV=2 - [SYDC_MOUSE]	Dars1	102.1109791	19.52124145	25.34547444	110.163209	87.98864772	48.34904297	48.99256499	82.16696657	46.09397855	31.31559931	0.4	0.764125594	1.677131348	0.745995681
Q9EST5	Acidic leucine-rich nuclear phosphoprotein 32 family member B OS=Mus musculus OX=10090 GN=Anp32b PE=1 SV=1 - [AN32B_MOUSE]	Anp32b	263.5930213	0	77.40470843	204.6245522	283.1303404	84.22093123	113.6659099	190.6586079	135.4860741	100.1874441	0.4	0.764125594	1.677359624	0.746192034
Q8CG19	Latent-transforming growth factor beta-binding protein 1 OS=Mus musculus OX=10090 GN=Ltbp1 PE=1 SV=2 - [LTBP1_MOUSE]	Ltbp1	29.07890171	62.02155604	21.92450366	44.57878138	89.36965451	57.28204824	37.67498713	63.74349471	21.38604488	23.08394024	0.4	0.764125594	1.691931426	0.758671097
Q4VAA2	Protein CDV3 OS=Mus musculus OX=10090 GN=Cdv3 PE=1 SV=2 - [CDV3_MOUSE]	Cdv3	124.550241	69.46742928	0	112.3217695	140.6927171	76.19662182	64.67255677	109.7370361	62.41340991	32.32564322	0.4	0.764125594	1.696809924	0.762824964
Q06186	Proheparin-binding EGF-like growth factor OS=Mus musculus OX=10090 GN=Hbegf PE=2 SV=1 - [HBEGF_MOUSE]	Hbegf	254.401386	211.1738284	0	250.8565051	233.9983956	306.5232553	155.1917381	263.7927187	136.1268237	37.95356419	0.4	0.764125594	1.699785838	0.765352988
P70195	Proteasome subunit beta type-7 OS=Mus musculus OX=10090 GN=Psmb7 PE=1 SV=1 - [PSB7_MOUSE]	Psmb7	116.5222624	34.29552556	42.83306487	130.7787819	94.24440388	105.5367364	64.55028427	110.1866407	45.21103046	18.70578523	0.4	0.764125594	1.706989241	0.771453965
P61971	Nuclear transport factor 2 OS=Mus musculus OX=10090 GN=Nutf2 PE=1 SV=1 - [NTF2_MOUSE]	Nutf2	404.1390361	99.7216606	121.3395447	498.5684959	280.1586732	289.6246305	208.4000805	356.1172665	169.859169	123.4571409	0.4	0.764125594	1.708815398	0.772996553
P80317	T-complex protein 1 subunit zeta OS=Mus musculus OX=10090 GN=Cct6a PE=1 SV=3 - [TCPZ_MOUSE]	Cct6a	304.8352501	67.2980434	79.3505497	364.6211869	278.112139	132.4396407	150.4946144	258.3909889	133.7986906	117.3403614	0.4	0.764125594	1.716945088	0.779843899
Q6P1B1	Xaa-Pro aminopeptidase 1 OS=Mus musculus OX=10090 GN=Xpnpep1 PE=1 SV=1 - [XPP1_MOUSE]	Xpnpep1	146.0233602	22.32331887	31.07731111	124.1181723	156.1055573	62.82129277	66.47466338	114.3483408	69.03009825	47.40333013	0.4	0.764125594	1.720179313	0.782558961
Q91YE6	Importin-9 OS=Mus musculus OX=10090 GN=Ipo9 PE=1 SV=3 - [IPO9_MOUSE]	Ipo9	30.8807574	2.064039853	6.656675875	24.06880213	33.02603412	11.43944697	13.20049104	22.84476107	15.48279498	10.84522446	0.4	0.764125594	1.730599339	0.791271757
Q8CIE6	Coatomer subunit alpha OS=Mus musculus OX=10090 GN=Copa PE=1 SV=2 - [COPA_MOUSE]	Copa	112.6098716	37.74083	0	114.0426559	108.2713577	39.48856765	50.11690054	87.26752709	57.3159763	41.47829185	0.4	0.764125594	1.741279412	0.800147721
Q9JMK0	Beta-1,4-galactosyltransferase 5 OS=Mus musculus OX=10090 GN=B4galt5 PE=1 SV=2 - [B4GT5_MOUSE]	B4galt5	25.66988849	29.07726366	0	20.45513323	42.28846813	32.8427662	18.24905071	31.86212252	15.89570509	10.94965181	0.4	0.764125594	1.745960544	0.804020957
Q9CZM2	60S ribosomal protein L15 OS=Mus musculus OX=10090 GN=Rpl15 PE=1 SV=4 - [RL15_MOUSE]	Rpl15	70.1081508	19.39093731	12.45035947	58.42629564	91.08980972	29.15883018	33.9831492	59.55831185	31.47705054	30.9810047	0.4	0.764125594	1.752583656	0.80948331
Q3U2P1	Protein transport protein Sec24A OS=Mus musculus OX=10090 GN=Sec24a PE=1 SV=1 - [SC24A_MOUSE]	Sec24a	42.7573988	9.988637499	12.74295959	42.29586428	50.16292027	22.42330122	21.8296653	38.29402859	18.17619583	14.29624541	0.4	0.764125594	1.754219685	0.810829431
P68369	Tubulin alpha-1A chain OS=Mus musculus OX=10090 GN=Tuba1a PE=1 SV=1 - [TBA1A_MOUSE]	Tuba1a	1669.43201	507.9326442	0	1179.455999	1718.642947	934.1691534	725.7882182	1277.4227	855.7725151	401.3077236	0.4	0.764125594	1.760048824	0.81561545
Q99KC8	von Willebrand factor A domain-containing protein 5A OS=Mus musculus OX=10090 GN=Vwa5a PE=1 SV=2 - [VMA5A_MOUSE]	Vwa5a	211.1098104	23.80246929	69.00590792	234.8801575	205.348909	95.24820587	101.3060625	178.4924241	97.7419306	73.58820262	0.4	0.764125594	1.761912561	0.817142329
Q8CI51	PDZ and LIM domain protein 5 OS=Mus musculus OX=10090 GN=Pdlim5 PE=1 SV=4 - [PDLI5_MOUSE]	Pdlim5	136.0436801	0	95.89773768	173.6265902	164.0163979	71.86830435	77.31380591	136.5037642	69.89987561	56.18181153	0.4	0.764125594	1.765580708	0.820142771
P08113	Endoplasmin OS=Mus musculus OX=10090 GN=Hsp90b1 PE=1 SV=2 - [ENPL_MOUSE]	Hsp90b1	203.4053019	53.11135698	77.02876856	201.8699005	245.9198736	141.8302325	111.1818091	196.5400022	80.75822005	52.24910723	0.4	0.764125594	1.767735241	0.821902214
Q99M71	Mammalian ependymin-related protein 1 OS=Mus musculus OX=10090 GN=Epdr1 PE=1 SV=1 - [EPDR1_MOUSE]	Epdr1	49.95741594	75.30666627	0	72.76257719	71.10558866	78.43463302	41.75469407	74.10093296	38.31758057	3.84345156	0.4	0.764125594	1.774673114	0.827553312
Q9D892	Inosine triphosphate pyrophosphatase OS=Mus musculus OX=10090 GN=Itpa PE=1 SV=2 - [ITPA_MOUSE]	Itpa	206.6526554	26.33504187	45.9256959	243.721216	193.483343	59.41298416	92.97113106	165.5391811	98.93717788	95.27873961	0.4	0.764125594	1.780543908	0.832318013
P63328	Serine/threonine-protein phosphatase 2B catalytic subunit alpha isoform OS=Mus musculus OX=10090 GN=Ppp3ca PE=1 SV=1 - [PP2BA_MOUSE]	Ppp3ca	82.02658362	46.37167175	0	105.701991	65.4637448	58.63389979	42.79941846	76.59987854	41.12980489	25.43347025	0.4	0.764125594	1.789741106	0.839750911
Q9DBP5	UMP-CMP kinase OS=Mus musculus OX=10090 GN=Cmpk1 PE=1 SV=1 - [KCY_MOUSE]	Cmpk1	219.0464425	46.12515446	25.22849197	221.9790562	179.1762729	118.794701	96.80002965	173.3166767	106.3828309	51.84114141	0.4	0.764125594	1.790460988	0.840331084
Q3TTY5	Keratin, type II cytoskeletal 2 epidermal OS=Mus musculus OX=10090 GN=Krt2 PE=1 SV=1 - [K22E_MOUSE]	Krt2	219.1904161	40.87094652	62.28045947	321.7243242	85.0760545	173.531997	107.447274	193.4441252	97.3626682	119.5741215	0.4	0.764125594	1.800363266	0.848288034
Q9D2M8	Ubiquitin-conjugating enzyme E2 variant 2 OS=Mus musculus OX=10090 GN=Ube2v2 PE=1 SV=4 - [UB2V2_MOUSE]	Ube2v2	682.2760866	222.5469313	69.60232948	676.71364	704.9473667	374.7530961	324.8084491	585.4713676	318.8813991	183.032588	0.4	0.764125594	1.80251274	0.850009456
Q9Z0P5	Twinfilin-2 OS=Mus musculus OX=10090 GN=Twf2 PE=1 SV=1 - [TWF2_MOUSE]	Twf2	110.067453	0	37.3942037	104.7828148	114.9019075	49.76208233	49.15388558	89.81560154	55.96810379	35.05441927	0.4	0.764125594	1.827232995	0.869660607
Q8BML9	Glutamine--tRNA ligase OS=Mus musculus OX=10090 GN=Qars1 PE=1 SV=1 - [SYQ_MOUSE]	Qars1	69.56148553	0	39.18280879	75.28746183	98.25679339	25.31665698	36.24809811	66.28697073	34.87347792	37.29373278	0.4	0.764125594	1.828702034	0.870820023
P62331	ADP-ribosylation factor 6 OS=Mus musculus OX=10090 GN=Arf6 PE=1 SV=2 - [ARF6_MOUSE]	Arf6	131.041266	27.89129148	0	90.05948151	131.5828437	69.2797768	52.97751915	96.97403401	69.02856265	31.72185861	0.4	0.764125594	1.830475182	0.872218213
P47955	60S acidic ribosomal protein P1 OS=Mus musculus OX=10090 GN=Rplp1 PE=1 SV=1 - [RLA1_MOUSE]	Rplp1	180.1503383	20.85676756	73.57978595	310.5859899	115.5853495	77.03199949	91.52896394	167.7344463	81.14949268	125.2058818	0.4	0.764125594	1.832583251	0.873878739
P80315	T-complex protein 1 subunit delta OS=Mus musculus OX=10090 GN=Cct4 PE=1 SV=3 - [TCPD_MOUSE]	Cct4	327.8812225	43.93926085	62.79909101	367.8117306	312.5362033	117.6400976	144.8731915	265.9960105	158.7698897	131.4190003	0.4	0.764125594	1.836060957	0.876613956
Q8QZS1	3-hydroxyisobutyryl-CoA hydrolase, mitochondrial OS=Mus musculus OX=10090 GN=Hibch PE=1 SV=1 - [HIBCH_MOUSE]	Hibch	34.69458828	18.60246273	0	43.94157533	43.97576067	9.95388535	17.76568367	32.62374045	17.36242389	19.63267786	0.4	0.764125594	1.836334647	0.876828994
P51855	Glutathione synthetase OS=Mus musculus OX=10090 GN=Gss PE=1 SV=1 - [GSHB_MOUSE]	Gss	74.50603181	0	51.32902786	89.94215383	97.76097889	47.66407555	41.94501989	78.45573609	38.12914762	26.95140582	0.4	0.764125594	1.870442219	0.9033794
Q80Y14	Glutaredoxin-related protein 5, mitochondrial OS=Mus musculus OX=10090 GN=Glrx5 PE=1 SV=2 - [GLRX5_MOUSE]	Glrx5	64.67343362	21.55781121	0	40.1702829	98.02645362	23.73166447	28.74374827	53.97613366	32.93010056	39.02409589	0.4	0.764125594	1.877839075	0.909073434
P80316	T-complex protein 1 subunit epsilon OS=Mus musculus OX=10090 GN=Cct5 PE=1 SV=1 - [TCPE_MOUSE]	Cct5	328.27063	39.30301218	66.51141252	397.6104028	297.2688196	121.2148746	144.6950182	272.0313657	159.5621452	139.9153987	0.4	0.764125594	1.880032699	0.910757755
Q3THE2	Myosin regulatory light chain 12B OS=Mus musculus OX=10090 GN=Myl12b PE=1 SV=2 - [ML12B_MOUSE]	Myl12b	83.31473335	0	30.68808574	101.6536561	67.61509623	45.87287189	38.0009397	71.71387473	42.13602465	28.11536874	0.4	0.764125594	1.887160562	0.916217175
O35900	U6 snRNA-associated Sm-like protein LSm2 OS=Mus musculus OX=10090 GN=Lsm2 PE=1 SV=1 - [LSM2_MOUSE]	Lsm2	75.43697714	0	26.06975266	82.02014695	69.5731543	40.25003198	33.8355766	63.94777774	38.31338404	21.44572853	0.4	0.764125594	1.88995679	0.91835325
P00375	Dihydrofolate reductase OS=Mus musculus OX=10090 GN=Dhfr PE=1 SV=3 - [DYR_MOUSE]	Dhfr	124.2454428	0	29.48756821	124.9540194	119.4472831	47.23052788	51.24433701	97.21061013	64.917257	43.37150565	0.4	0.764125594	1.897002007	0.923721205
P26039	Talin-1 OS=Mus musculus OX=10090 GN=Tln1 PE=1 SV=2 - [TLN1_MOUSE]	Tln1	202.1001456	36.07001053	41.77163729	179.8422597	276.4066043	77.00870612	93.31393115	177.7525234	94.2547478	99.71537343	0.4	0.764125594	1.904887311	0.929705653
P97372	Proteasome activator complex subunit 2 OS=Mus musculus OX=10090 GN=Psme2 PE=1 SV=4 - [PSME2_MOUSE]	Psme2	70.51262144	38.13037714	12.46029231	118.973547	78.66088491	33.14789176	40.36776363	76.92744122	29.09076577	42.93907769	0.4	0.764125594	1.905665172	0.930294658
P51150	Ras-related protein Rab-7a OS=Mus musculus OX=10090 GN=Rab7a PE=1 SV=2 - [RAB7A_MOUSE]	Rab7a	143.7170838	57.04839307	0	123.5214847	178.4210235	81.64763363	66.92182563	127.8633806	72.36548588	48.53257972	0.4	0.764125594	1.910637963	0.934054436

Q8R081	Heterogeneous nuclear ribonucleoprotein L OS=Mus musculus OX=10090 GN=Hnrnpl PE=1 SV=2 - [HNRPL_MOUSE]	Hnrnpl	355.5935113	64.33027084	58.52968115	350.7785699	402.4153202	162.0088815	159.4844878	305.0675905	169.8601588	126.5540761	0.4	0.764125594	1.912835504	0.935712813
Q8R0J7	Vacuolar protein sorting-associated protein 37B OS=Mus musculus OX=10090 GN=Vps37b PE=1 SV=1 - [VP37B_MOUSE]	Vps37b	15.32284436	0	22.76575923	11.97507045	30.56774467	30.34928539	12.6962012	24.29736684	11.60794508	10.67198071	0.4	0.764125594	1.913750929	0.936403078
Q9CYR6	Phosphoacetylglucosamine mutase OS=Mus musculus OX=10090 GN=Pgm3 PE=1 SV=1 - [AGM1_MOUSE]	Pgm3	121.930793	20.76789823	22.14489512	116.7886264	136.1106743	63.06391873	54.9478621	105.3210731	58.01300544	37.84951396	0.4	0.764125594	1.916745604	0.938658871
Q8BKC5	Importin-5 OS=Mus musculus OX=10090 GN=Ipo5 PE=1 SV=3 - [IPO5_MOUSE]	Ipo5	208.7276264	48.64782112	32.68148646	197.8276224	255.588493	103.4747746	96.68564465	185.6302967	97.35905305	76.78689272	0.4	0.764125594	1.919936484	0.941058584
P11352	Glutathione peroxidase 1 OS=Mus musculus OX=10090 GN=Gpx1 PE=1 SV=2 - [GPX1_MOUSE]	Gpx1	110.3000208	18.97996916	22.38672471	110.0649257	116.0445056	65.12443593	50.55557155	97.07795575	51.76824225	27.83360228	0.4	0.764125594	1.920222693	0.941273634
Q9WTP7	GTP:AMP phosphotransferase AK3, mitochondrial OS=Mus musculus OX=10090 GN=Ak3 PE=1 SV=3 - [KAD3_MOUSE]	Ak3	71.71931407	23.60815323	0	77.74933568	55.87275096	50.43148715	31.77582243	61.35119126	36.55062404	14.45946725	0.4	0.764125594	1.930750696	0.949161891
Q60519	Semaphorin-5B OS=Mus musculus OX=10090 GN=Sema5b PE=2 SV=2 - [SEM5B_MOUSE]	Sema5b	0	28.52517283	10.1957528	23.8660708	18.05027517	32.94007221	12.90697521	24.95213939	14.45456386	7.504077069	0.4	0.764125594	1.933229048	0.951012577
Q6P5F9	Exportin-1 OS=Mus musculus OX=10090 GN=Xpo1 PE=1 SV=1 - [XPO1_MOUSE]	Xpo1	269.4742002	36.46271161	48.47462558	261.2489045	317.017253	109.7662375	118.1371791	229.3441317	131.1992453	107.2458939	0.4	0.764125594	1.941337464	0.957050924
Q8R242	Di-N-acetylchitobiase OS=Mus musculus OX=10090 GN=Ctbs PE=1 SV=2 - [DIAC_MOUSE]	Ctbs	0	16.49622491	67.57131654	35.56167507	55.58279754	74.19090902	28.02251382	55.11179388	35.22942246	19.31892368	0.4	0.764125594	1.966697001	0.975774706
Q9D0R2	Threonine--tRNA ligase 1, cytoplasmic OS=Mus musculus OX=10090 GN=Tars1 PE=1 SV=2 - [SYTC_MOUSE]	Tars1	133.8822861	10.25945371	26.95241294	152.3286613	128.9647632	56.03378781	57.03138426	112.4424041	67.07614747	50.22864647	0.4	0.764125594	1.971588197	0.97935825
P50580	Proliferation-associated protein 2G4 OS=Mus musculus OX=10090 GN=Pa2g4 PE=1 SV=3 - [PA2G4_MOUSE]	Pa2g4	360.5608131	68.9116889	41.73897045	312.8728578	470.5469652	153.9588018	157.0704908	312.4595416	176.7507358	158.2944864	0.4	0.764125594	1.989294997	0.992257233
Q91WQ3	Tyrosine--tRNA ligase, cytoplasmic OS=Mus musculus OX=10090 GN=Yars1 PE=1 SV=3 - [SYYC_MOUSE]	Yars1	84.48460864	0	13.11942902	86.6505523	79.77441937	28.10048696	32.53467922	64.84181954	45.46565989	32.00413162	0.4	0.764125594	1.993006266	0.994946246
P28474	Alcohol dehydrogenase class-3 OS=Mus musculus OX=10090 GN=Adh5 PE=1 SV=3 - [ADHX_MOUSE]	Adh5	257.6071398	48.49312842	46.33392211	401.449817	177.0690078	126.9897791	117.4780635	235.1695347	121.360142	146.1637148	0.4	0.764125594	2.001816575	1.001309787
Q9CQR2	40S ribosomal protein S21 OS=Mus musculus OX=10090 GN=Rps21 PE=1 SV=1 - [RS21_MOUSE]	Rps21	193.5481657	0	64.51605523	146.6350201	193.5649951	177.9041023	86.02140697	172.7013725	98.54990221	23.89365893	0.4	0.764125594	2.007655752	1.005511915
P42227	Signal transducer and activator of transcription 3 OS=Mus musculus OX=10090 GN=Stat3 PE=1 SV=2 - [STAT3_MOUSE]	Stat3	114.6889369	0	45.01324245	113.7326374	139.7151031	70.12353515	53.23405979	107.8570919	57.78472497	35.16586566	0.4	0.764125594	2.026091797	1.018699541
Q6A026	Sister chromatid cohesion protein PDS5 homolog A OS=Mus musculus OX=10090 GN=Pds5a PE=1 SV=3 - [PDS5A_MOUSE]	Pds5a	28.59151018	0	8.046611697	30.34091831	27.50845633	16.70281123	12.21270729	24.85072862	14.7440116	7.197022201	0.4	0.764125594	2.034825533	1.024905102
P32067	Lupus La protein homolog OS=Mus musculus OX=10090 GN=Ssb PE=1 SV=1 - [LA_MOUSE]	Ssb	61.63340984	16.10013955	0	53.32475567	69.56195668	36.04612744	25.91118313	52.97761326	31.96657214	16.76061106	0.4	0.764125594	2.044584881	1.031807957
P62830	60S ribosomal protein L23 OS=Mus musculus OX=10090 GN=Rpl23 PE=1 SV=1 - [RL23_MOUSE]	Rpl23	429.5561438	23.29766902	30.20691513	362.5601271	582.8954548	47.0209524	161.0202427	330.8255114	232.5845698	269.3430618	0.4	0.764125594	2.05455852	1.038828423
P61205	ADP-ribosylation factor 3 OS=Mus musculus OX=10090 GN=Arf3 PE=2 SV=2 - [ARF3_MOUSE]	Arf3	715.9005827	0	213.841022	578.6360491	896.2435755	447.6104983	309.9138682	640.830041	367.4927277	230.6923903	0.4	0.764125594	2.067768198	1.048074465
Q8JZQ9	Eukaryotic translation initiation factor 3 subunit B OS=Mus musculus OX=10090 GN=Eif3b PE=1 SV=1 - [EIF3B_MOUSE]	Eif3b	39.01095664	30.71133546	13.65467192	67.84074129	87.35986159	20.94048543	27.79232134	58.7136961	12.9277135	34.13737831	0.4	0.764125594	2.112586976	1.079010739
Q9JIF7	Coatomer subunit beta OS=Mus musculus OX=10090 GN=Copb1 PE=1 SV=1 - [COPB_MOUSE]	Copb1	71.15790933	0	11.66613145	55.2473916	86.16605544	36.00275039	27.60801359	59.13873248	38.16372256	25.30703842	0.4	0.764125594	2.142085749	1.099016233
P80314	T-complex protein 1 subunit beta OS=Mus musculus OX=10090 GN=Cct2 PE=1 SV=4 - [TCPB_MOUSE]	Cct2	276.7910064	33.34863024	37.62658545	324.3930504	269.7755554	152.781644	115.922074	248.9834166	139.3330014	87.67470265	0.4	0.764125594	2.147851638	1.102894343
Q8BK67	Protein RCC2 OS=Mus musculus OX=10090 GN=Rcc2 PE=1 SV=1 - [RCC2_MOUSE]	Rcc2	310.6379375	0	55.08279046	341.7154889	304.1990864	143.871487	121.9069093	263.2620208	165.7500504	105.0830375	0.4	0.764125594	2.159533223	1.110719512
B2RQC6	CAD protein OS=Mus musculus OX=10090 GN=Cad PE=1 SV=1 - [PYR1_MOUSE]	Cad	41.84283744	0	10.62346304	37.99192579	52.33990823	24.15002699	17.48876683	38.16062034	21.74982893	14.09569773	0.4	0.764125594	2.182007497	1.125656059
Q9D0E1	Heterogeneous nuclear ribonucleoprotein M OS=Mus musculus OX=10090 GN=Hnrnpm PE=1 SV=3 - [HNRPM_MOUSE]	Hnrnpm	74.23223407	0	14.47935308	78.55989535	68.98869264	49.18286266	29.57052905	65.57715022	39.34988993	14.98270599	0.4	0.764125594	2.217652248	1.149033153
Q8CHU3	Epsin-2 OS=Mus musculus OX=10090 GN=Epn2 PE=1 SV=1 - [EPN2_MOUSE]	Epn2	192.5638301	0	59.16617071	179.6689315	178.4400893	212.0789918	83.91000026	190.0626709	98.63772092	19.07659044	0.4	0.764125594	2.265077706	1.179560544
Q9R087	Glypican-6 OS=Mus musculus OX=10090 GN=Gpc6 PE=1 SV=1 - [GPC6_MOUSE]	Gpc6	34.12579428	7.341077878	0	25.08147123	29.256111	40.56620203	13.82229072	31.63459475	17.9623787	8.011685883	0.4	0.764125594	2.288665128	1.194506386
Q8BJD1	Inter-alpha-trypsin inhibitor heavy chain H5 OS=Mus musculus OX=10090 GN=Itih5 PE=1 SV=1 - [ITIH5_MOUSE]	Itih5	71.82233151	77.46349333	83.16934379	441.8752109	86.66904388	73.83502273	77.48505621	200.7930925	5.673536872	208.8818299	0.4	0.764125594	2.591378291	1.373719636
Q8BH95	Enoyl-CoA hydratase, mitochondrial OS=Mus musculus OX=10090 GN=Echs1 PE=1 SV=1 - [ECHM_MOUSE]	Echs1	68.40089682	0	16.70483049	37.16747107	64.89530756	158.0261019	28.36857577	86.69629353	35.66094344	63.31006559	0.4	0.764125594	3.056067891	1.611676593
Q3UPL0	Protein transport protein Sec31A OS=Mus musculus OX=10090 GN=Sec31a PE=1 SV=2 - [SC31A_MOUSE]	Sec31a	64.67270621	10.14341555	12.07946444	254.9326782	58.50547123	17.93288395	28.9651954	110.4570111	30.93875915	126.7534911	0.4	0.764125594	3.813439185	1.931092692
Q62523	Zyxin OS=Mus musculus OX=10090 GN=Zyx PE=1 SV=2 - [ZYX_MOUSE]	Zyx	85.32315143	31.85269263	27.20148738	636.5026899	60.39492233	44.00463084	48.12577714	246.9674143	32.29770762	337.4469715	0.4	0.764125594	5.131707559	2.359438958
P61164	Alpha-centractin OS=Mus musculus OX=10090 GN=Actr1a PE=1 SV=1 - [ACTZ_MOUSE]	Actr1a	79.69496791	0	57.35250868	788.5960695	699.2550817	21.58641308	45.68249219	503.1458548	41.10917101	419.4282715	0.4	0.764125594	11.01397561	3.461263414
A2ALU4	Protein Shroom2 OS=Mus musculus OX=10090 GN=Shroom2 PE=1 SV=1 - [SHRM2_MOUSE]	Shroom2	0	0	0	0	0	47.24199815	0	15.74733272	0	27.27518035	0.504985075	0.764125594	only in a1H	only in a1H
A9Q751	Cilia- and flagella-associated protein 221 OS=Mus musculus OX=10090 GN=Cfap221 PE=1 SV=1 - [PCDP1_MOUSE]	Cfap221	0	0	0	0	17.27167741	0	0	5.757225803	0	9.971807601	0.504985075	0.764125594	only in a1H	only in a1H
B0F2B4	Neuroligin 4-like OS=Mus musculus OX=10090 GN=Nlgn4l PE=1 SV=1 - [NLGN4_MOUSE]	Nlgn4l	0	0	0	0	0	43.48849802	0	14.49616601	0	25.10809604	0.504985075	0.764125594	only in a1H	only in a1H
B8ZXI1	Queuine tRNA-ribosyltransferase accessory subunit 2 OS=Mus musculus OX=10090 GN=Qtrt2 PE=1 SV=2 - [QTRT2_MOUSE]	Qtrt2	0	0	0	0	5.358638144	0	0	1.786212715	0	3.093811175	0.504985075	0.764125594	only in a1H	only in a1H
E9Q4F7	Ankyrin repeat domain-containing protein 11 OS=Mus musculus OX=10090 GN=Ankrd11 PE=1 SV=1 - [ANR11_MOUSE]	Ankrd11	0	0	0	0	0	11.26026761	0	3.753422538	0	6.501118538	0.504985075	0.764125594	only in a1H	only in a1H
E9Q612	Receptor-type tyrosine-protein phosphatase O OS=Mus musculus OX=10090 GN=Ptpro PE=1 SV=1 - [PTPRO_MOUSE]	Ptpro	0	0	0	212.1819899	0	0	0	70.72732998	0	122.503329	0.504985075	0.764125594	only in a1H	only in a1H
E9Q8T7	Dynein heavy chain 1, axonemal OS=Mus musculus OX=10090 GN=Dnah1 PE=1 SV=1 - [DYH1_MOUSE]	Dnah1	0	0	0	0	288.8343239	0	0	96.27810796	0	166.7585746	0.504985075	0.764125594	only in a1H	only in a1H
O08784	Treacle protein OS=Mus musculus OX=10090 GN=Tcof1 PE=1 SV=1 - [TCOF_MOUSE]	Tcof1	0	0	0	5.335966869	0	0	0	1.778655623	0	3.080721908	0.504985075	0.764125594	only in a1H	only in a1H
O09118	Netrin-1 OS=Mus musculus OX=10090 GN=Ntn1 PE=1 SV=3 - [NET1_MOUSE]	Ntn1	0	0	0	0	22.32799207	0	0	7.442664023	0	12.89107223	0.504985075	0.764125594	only in a1H	only in a1H
O35207	Cyclin-dependent kinase 2-associated protein 1 OS=Mus musculus OX=10090 GN=Cdk2ap1 PE=1 SV=2 - [CDKA1_MOUSE]	Cdk2ap1	0	0	0	7.626888105	0	0	0	2.542296035	0	4.403385901	0.504985075	0.764125594	only in a1H	only in a1H
O35423	Serine--pyruvate aminotransferase, mitochondrial OS=Mus musculus OX=10090 GN=Agxt PE=1 SV=3 - [SPYA_MOUSE]	Agxt	0	0	0	15.58845909	0	0	0	5.196153029	0	9.00000105	0.504985075	0.764125594	only in a1H	only in a1H
O54751	Cone-rod homeobox protein OS=Mus musculus OX=10090 GN=Crx PE=1 SV=1 - [CRX_MOUSE]	Crx	0	0	0	0	48.68670942	0	0	16.22890314	0	28.10928479	0.504985075	0.764125594	only in a1H	only in a1H
O54834	Rho GTPase-activating protein 6 OS=Mus musculus OX=10090 GN=Arhgap6 PE=1 SV=3 - [RHG06_MOUSE]	Arhgap6	0	0	0	0	0	16.38331507	0	5.461105024	0	9.458911366	0.504985075	0.764125594	only in a1H	only in a1H
O70274	Protein tyrosine phosphatase type IVA 2 OS=Mus musculus OX=10090 GN=Ptp4a2 PE=1 SV=1 - [TP4A2_MOUSE]	Ptp4a2	0	0	0	0	12.92263692	0	0	4.30754564	0	7.460887905	0.504985075	0.764125594	only in a1H	only in a1H
O70475	UDP-glucose 6-dehydrogenase OS=Mus musculus OX=10090 GN=Ugdh PE=1 SV=1 - [UGDH_MOUSE]	Ugdh	0	0	0	0	16.48742608	0	0	5.495808695	0	9.519019888	0.504985075	0.764125594	only in a1H	only in a1H
O88199	Carbohydrate sulfotransferase 3 OS=Mus musculus OX=10090 GN=Chst3 PE=2 SV=1 - [CHST3_MOUSE]	Chst3	0	0	0	0	25.3194324	0	0	8.439810799	0	14.61818111	0.504985075	0.764125594	only in a1H	only in a1H
O88466	Zinc finger protein 106 OS=Mus musculus OX=10090 GN=Znf106 PE=1 SV=3 - [ZN106_MOUSE]	Znf106	0	0	0	0	150.90642	0	0	50.30214002	0	87.12586224	0.504985075	0.764125594	only in a1H	only in a1H
O88545	COP9 signalosome complex subunit 6 OS=Mus musculus OX=10090 GN=Cops6 PE=1 SV=1 - [CSN6_MOUSE]	Cops6	0	0	0	0	38.71770916	0	0	12.90590305	0	22.35367981	0.504985075	0.764125594	only in a1H	only in a1H
O88566	Axin-2 OS=Mus musculus OX=10090 GN=Axin2 PE=1 SV=2 - [AXIN2_MOUSE]	Axin2	0	0	0	35.17455695	0	0	0	11.72485232	0	20.30803992	0.504985075	0.764125594	only in a1H	only in a1H
O88809	Neuronal migration protein doublecortin OS=Mus musculus OX=10090 GN=Dcx PE=1 SV=1 - [DCX_MOUSE]	Dcx	0	0	0	0	22.51944493	0	0	7.506481645	0	13.0016076	0.504985075	0.764125594	only in a1H	only in a1H
P01898	H-2 class I histocompatibility antigen, Q10 alpha chain OS=Mus musculus OX=10090 GN=H2-Q10 PE=1 SV=3 - [HA10_MOUSE]	H2-Q10	0	0	0	0	38.76219806	0	0	12.92073269	0	22.37936549	0.504985075	0.764125594	only in a1H	only in a1H
P02716	Acetylcholine receptor subunit delta OS=Mus musculus OX=10090 GN=Chrnd PE=2 SV=1 - [ACHD_MOUSE]	Chrnd	0	0	0	0	8788.901127	0	0	2929.633709	0	5074.274431	0.504985075	0.764125594	only in a1H	only in a1H
P04223	H-2 class I histocompatibility antigen, K-K alpha chain OS=Mus musculus OX=10090 GN=H2-K1 PE=1 SV=1 - [HA1K_MOUSE]	H2-K1	0	0	0	359.5166515	0	0	0	119.8388838	0	207.5670355	0.504985075	0.764125594	only in a1H	only in a1H
P06800	Receptor-type tyrosine-protein phosphatase C OS=Mus musculus OX=10090 GN=Ptprc PE=1 SV=4 - [PTPRC_MOUSE]	Ptprc	0	0	0	169.63415	0	0	0	56.54471667	0	97.93832217	0.504985075	0.764125594	only in a1H	only in a1H
P07742	Ribonucleoside-diphosphate reductase large subunit OS=Mus musculus OX=10090 GN=Rrm1 PE=1 SV=2 - [RIR1_MOUSE]	Rrm1	0	0	0	0	16.98947403	0	0	5.663158009	0	9.808877403	0.504985075	0.764125594	only in a1H	only in a1H
P09793	Cytotoxic T-lymphocyte protein 4 OS=Mus musculus OX=10090 GN=Ctla4 PE=1 SV=1 - [CTLA4_MOUSE]	Ctla4	0	0	0	100.6005325	0	0	0	33.53351083	0	58.08174452	0.504985075	0.764125594	only in a1H	only in a1H
P10148	C-C motif chemokine 2 OS=Mus musculus OX=10090 GN=Ccl2 PE=1 SV=1 - [CCL2_MOUSE]	Ccl2	0	0	0	79.56196949	0	0	0	26.5206565	0	45.9351245	0.504985075	0.764125594	only in a1H	only in a1H
P14431	H-2 class I histocompatibility antigen, Q9 alpha chain (Fragment) OS=Mus musculus OX=10090 GN=H2-Q9 PE=3 SV=1 - [HA19_MOUSE]	H2-Q9	0	0	0	0	0	18.90842936	0	6.302809787	0	10.91678678	0.504985075	0.764125594	only in a1H	only in a1H
P15388	Potassium voltage-gated channel subfamily C member 1 OS=Mus musculus OX=10090 GN=Kcnc1 PE=1 SV=1 - [KCNC1_MOUSE]	Kcnc1	0	0	0	0	166.4085835	0	0	55.46952783	0	96.07604048	0.504985075	0.764125594	only in a1H	only in a1H
P16056	Hepatocyte growth factor receptor OS=Mus musculus OX=10090 GN=Met PE=1 SV=1 - [MET_MOUSE]	Met	0	0	0	0	12.40688549	0	0	4.135628497	0	7.163118679	0.504985075	0.764125594	only in a1H	only in a1H
P16301	Phosphatidylcholine-sterol acyltransferase OS=Mus musculus OX=10090 GN=Lcat PE=1 SV=2 - [LCAT_MOUSE]	Lcat	0	0	0	0	12.69055557	0	0	4.230185189	0	7.326895672	0.504985075	0.764125594	only in a1H	only in a1H
P17095	High mobility group protein HMG-I/HMG-Y OS=Mus musculus OX=10090 GN=Hmga1 PE=1 SV=4 - [HMGA1_MOUSE]	Hmga1	0	0	0	0	59.6659311	0	0	19.8886437	0	34.44814138	0.504985075	0.764125594	only in a1H	only in a1H
P17563	Methanethiol oxidase OS=Mus musculus OX=10090 GN=Selenbp1 PE=1 SV=2 - [SBP1_MOUSE]	Selenbp1	0	0	0	22.84796079	0	0	0	7.615986929	0	13.19127631	0.504985075	0.764125594	only in a1H	only in a1H
P18052	Receptor-type tyrosine-protein phosphatase alpha OS=Mus musculus OX=10090 GN=Ptpra PE=1 SV=3 - [PTPRA_MOUSE]	Ptpra	0	0	0	0	4.030892202	0	0	1.343630734	0	2.327236698	0.504985075	0.764125594	only in a1H	only in a1H
P18654	Ribosomal protein S6 kinase alpha-3 OS=Mus musculus OX=10090 GN=Rps6ka3 PE=1 SV=2 - [KS6A3_MOUSE]	Rps6ka3	0	0	0	0	6.596358456	0	0	2.198786152	0	3.80840933	0.504985075	0.764125594	only in a1H	only in a1H
P23359	Bone morphogenetic protein 7 OS=Mus musculus OX=10090 GN=Bmp7 PE=1 SV=2 - [BMP7_MOUSE]	Bmp7	0	0	0	133.9412708	0	0	0	44.64709027	0	77.33102875	0.504985075	0.764125594	only in a1H	only in a1H
P26450	Phosphatidylinositol 3-kinase regulatory subunit alpha OS=Mus musculus OX=10090 GN=Pik3r1 PE=1 SV=2 - [P85A_MOUSE]	Pik3r1	0	0	0	7.488721306	0	0	0	2.496240435	0	4.323615262	0.504985075	0.764125594	only in a1H	only in a1H
P30677	Guanine nucleotide-binding protein subunit alpha-14 OS=Mus musculus OX=10090 GN=Gna14 PE=1 SV=2 - [GNA14_MOUSE]	Gna14	0	0	0	5.623410611	0	0	0	1.874470204	0	3.24667763	0.504985075	0.764125594	only in a1H	only in a1H
P33435	Collagenase 3 OS=Mus musculus OX=10090 GN=Mmp13 PE=1 SV=1 - [MMP13_MOUSE]	Mmp13	0	0	0	0	239.6183576	0	0	79.87278587	0	138.3437233	0.504985075	0.764125594	only in a1H	only in a1H
P36993	Protein phosphatase 1B OS=Mus musculus OX=10090 GN=Ppm1b PE=1 SV=1 - [PPM1B_MOUSE]	Ppm1b	0	0	0	29.98172358	0	0	0	9.993907859	0	17.30995618	0.504985075	0.764125594	only in a1H	only in a1H
P38060	Hydroxymethylglutaryl-CoA lyase, mitochondrial OS=Mus musculus OX=10090 GN=Hmgcl PE=1 SV=2 - [HMGCL_MOUSE]	Hmgcl	0	0	0	12.0202746	0	0	0	4.006758201	0	6.939908777	0.504985075	0.764125594	only in a1H	only in a1H
P42225	Signal transducer and activator of transcription 1 OS=Mus musculus OX=10090 GN=Stat1 PE=1 SV=1 - [STAT1_MOUSE]	Stat1	0	0	0	67.14606736	0	0	0	22.38202245	0	38.76680007	0.504985075	0.764125594	only in a1H	only in a1H
P42859	Huntingtin OS=Mus musculus OX=10090 GN=Htt PE=1 SV=2 - [HD_MOUSE]	Htt	0	0	0	2089.964948	0	0	0	696.6549826	0	1206.641825	0.504985075	0.764125594	only in a1H	only in a1H
P43275	Histone H1.1 OS=Mus musculus OX=10090 GN=H1-1 PE=1 SV=2 - [H11_MOUSE]	H1-1	0	0	0	0	0	38.55986251	0	12.8532875	0	22.262547	0.504985075	0.764125594	only in a1H	only in a1H
P45700	Mannosyl-oligosaccharide 1,2-alpha-mannosidase IA OS=Mus musculus OX=10090 GN=Man1a1 PE=1 SV=1 - [MA1A1_MOUSE]	Man1a1	0	0	0	4.932394133	0	0	0	1.644131378	0	2.847719081	0.504985075	0.764125594	only in a1H	only in a1H
P46737	Lys-63-specific deubiquitinase BRCC36 OS=Mus musculus OX=10090 GN=Brcc3 PE=1 SV=1 - [BRCC3_MOUSE]	Brcc3	0	0	0	0	18.18845479	0	0	6.062818263	0	10.50110927	0.504985075	0.764125594	only in a1H	only in a1H
P47738	Aldehyde dehydrogenase, mitochondrial OS=Mus musculus OX=10090 GN=Aldh2 PE=1 SV=1 - [ALDH2_MOUSE]	Aldh2	0	0	0	0	36.95071148	0	0	12.31690383	0	21.33350322	0.504985075	0.764125594	only in a1H	only in a1H
P48302	Endothelin receptor type B OS=Mus musculus OX=10090 GN=Ednrb PE=2 SV=1 - [EDNRB_MOUSE]	Ednrb	0	0	0	0	46.86226612	0	0	15.62075537	0	27.05594196	0.504985075	0.764125594	only in a1H	only in a1H
P49813	Tropomodulin-1 OS=Mus musculus OX=10090 GN=Tmod1 PE=1 SV=2 - [TMOD1_MOUSE]	Tmod1	0	0	0	0	0	52.21507736	0	17.40502579	0	30.14638897	0.504985075	0.764125594	only in a1H	only in a1H
P49935	Pro-cathepsin H OS=Mus musculus OX=10090 GN=Ctsh PE=1 SV=2 - [CATH_MOUSE]	Ctsh	0	0	0	0	0	14.00171545	0	4.667238483	0	8.083894184	0.504985075	0.764125594	only in a1H	only in a1H
P50652	Fanconi anemia group C protein homolog OS=Mus musculus OX=10090 GN=Fancc PE=2 SV=2 - [FANCC_MOUSE]	Fancc	0	0	0	0	4.682213031	0	0	1.560737677	0	2.703276954	0.504985075	0.764125594	only in a1H	only in a1H
P52800	Ephrin-B2 OS=Mus musculus OX=10090 GN=Efnb2 PE=1 SV=1 - [EFNB2_MOUSE]	Efnb2	0	0	0	0	30.83211338	0	0	10.27737113	0	17.80092896	0.504985075	0.764125594	only in a1H	only in a1H
P53690	Matrix metalloproteinase-14 OS=Mus musculus OX=10090 GN=Mmp14 PE=2 SV=3 - [MMP14_MOUSE]	Mmp14	0	0	0	0	25.89003781	0	0	8.630012603	0	14.9476203	0.504985075	0.764125594	only in a1H	only in a1H
P55271	Cyclin-dependent kinase 4 inhibitor B OS=Mus musculus OX=10090 GN=Cdkn2b PE=1 SV=1 - [CDN2B_MOUSE]	Cdkn2b	0	0	0	5.647978667	0	0	0	1.882659556	0	3.260862004	0.504985075	0.764125594	only in a1H	only in a1H
P55288	Cadherin-11 OS=Mus musculus OX=10090 GN=Cdh11 PE=1 SV=1 - [CAD11_MOUSE]	Cdh11	0	0	0	0	0	43.08522642	0	14.36174214	0	24.87526707	0.504985075	0.764125594	only in a1H	only in a1H
P57716	Nicastrin OS=Mus musculus OX=10090 GN=Ncstn PE=1 SV=3 - [NICA_MOUSE]	Ncstn	0	0	0	0	10.59465645	0	0	3.531552152	0	6.116827756	0.504985075	0.764125594	only in a1H	only in a1H
P58242	Acid sphingomyelinase-like phosphodiesterase 3b OS=Mus musculus OX=10090 GN=Smpdl3b PE=1 SV=1 - [ASM3B_MOUSE]	Smpdl3b	0	0	0	0	5.456068087	0	0	1.818689362	0	3.150062379	0.504985075	0.764125594	only in a1H	only in a1H
P58281	Dynamin-like 120 kDa protein, mitochondrial OS=Mus musculus OX=10090 GN=Opa1 PE=1 SV=1 - [OPA1_MOUSE]	Opa1	0	0	0	15.26543311	0	0	0	5.088477703	0	8.813501915	0.504985075	0.764125594	only in a1H	only in a1H
P59325	Eukaryotic translation initiation factor 5 OS=Mus musculus OX=10090 GN=Eif5 PE=1 SV=1 - [IF5_MOUSE]	Eif5	0	0	0	0	22.40114442	0	0	7.467048139	0	12.93330676	0.504985075	0.764125594	only in a1H	only in a1H
P59384	A disintegrin and metalloproteinase with thrombospondin motifs 15 OS=Mus musculus OX=10090 GN=Adamts15 PE=1 SV=2 - [ATS15_MOUSE]	Adamts15	0	0	0	27.59290388	0	0	0	9.197634628	0	15.93077049	0.504985075	0.764125594	only in a1H	only in a1H
P62141	Serine/threonine-protein phosphatase PP1-beta catalytic subunit OS=Mus musculus OX=10090 GN=Ppp1cb PE=1 SV=3 - [PP1B_MOUSE]	Ppp1cb	0	0	0	112.4096945	0	0	0	37.46989815	0	64.89976736	0.504985075	0.764125594	only in a1H	only in a1H
P62835	Ras-related protein Rap-1A OS=Mus musculus OX=10090 GN=Rap1a PE=1 SV=1 - [RAP1A_MOUSE]	Rap1a	0	0	0	67.78689517	0	0	0	22.59563172	0	39.13678217	0.504985075	0.764125594	only in a1H	only in a1H
P63037	DnaJ homolog subfamily A member 1 OS=Mus musculus OX=10090 GN=Dnaja1 PE=1 SV=1 - [DNJA1_MOUSE]	Dnaja1	0	0	0	0	24.74085385	0	0	8.246951283	0	14.28413863	0.504985075	0.764125594	only in a1H	only in a1H

P70279	Surfeit locus protein 6 OS=Mus musculus OX=10090 GN=Surf6 PE=1 SV=1 - [SURF6_MOUSE]	Surf6	0	0	0	0	47.59831275	0	0	15.86610425	0	27.48089868	0.504985075	0.764125594	only in a1H	only in a1H
P70336	Rho-associated protein kinase 2 OS=Mus musculus OX=10090 GN=Rock2 PE=1 SV=1 - [ROCK2_MOUSE]	Rock2	0	0	0	18.9259444	0	0	0	6.308648134	0	10.9268991	0.504985075	0.764125594	only in a1H	only in a1H
P70460	Vasodilator-stimulated phosphoprotein OS=Mus musculus OX=10090 GN=Vasp PE=1 SV=4 - [VASP_MOUSE]	Vasp	0	0	0	28.49538296	0	0	0	9.498460987	0	16.45181702	0.504985075	0.764125594	only in a1H	only in a1H
P70698	CTP synthase 1 OS=Mus musculus OX=10090 GN=Ctps1 PE=1 SV=2 - [PYRG1_MOUSE]	Ctps1	0	0	0	0	11.49772623	0	0	3.832575409	0	6.638215332	0.504985075	0.764125594	only in a1H	only in a1H
P82198	Transforming growth factor-beta-induced protein ig-h3 OS=Mus musculus OX=10090 GN=Tgfbi PE=1 SV=1 - [BGH3_MOUSE]	Tgfbi	0	0	0	3.386717841	0	0	0	1.128905947	0	1.955322457	0.504985075	0.764125594	only in a1H	only in a1H
P83877	Thioredoxin-like protein 4A OS=Mus musculus OX=10090 GN=Txnl4a PE=1 SV=1 - [TXN4A_MOUSE]	Txnl4a	0	0	0	14.37807821	0	0	0	4.792692737	0	8.301187326	0.504985075	0.764125594	only in a1H	only in a1H
P84102	Small EDRK-rich factor 2 OS=Mus musculus OX=10090 GN=Serf2 PE=1 SV=1 - [SERF2_MOUSE]	Serf2	0	0	0	39.33604821	0	0	0	13.11201607	0	22.71067802	0.504985075	0.764125594	only in a1H	only in a1H
P85094	Isochorismatase domain-containing protein 2A OS=Mus musculus OX=10090 GN=Isoc2a PE=1 SV=1 - [ISC2A_MOUSE]	Isoc2a	0	0	0	0	6.031065911	0	0	2.010355304	0	3.482037527	0.504985075	0.764125594	only in a1H	only in a1H
P97377	Cyclin-dependent kinase 2 OS=Mus musculus OX=10090 GN=Cdk2 PE=1 SV=2 - [CDK2_MOUSE]	Cdk2	0	0	0	76.88528573	0	0	0	25.62842858	0	44.38974041	0.504985075	0.764125594	only in a1H	only in a1H
P97785	GDNF family receptor alpha-1 OS=Mus musculus OX=10090 GN=Gfra1 PE=1 SV=2 - [GFRA1_MOUSE]	Gfra1	0	0	0	0	30.3577447	0	0	10.11924823	0	17.52705208	0.504985075	0.764125594	only in a1H	only in a1H
P98064	Mannan-binding lectin serine protease 1 OS=Mus musculus OX=10090 GN=Masp1 PE=1 SV=2 - [MASP1_MOUSE]	Masp1	0	0	0	0	0	75.96653211	0	25.32217737	0	43.85929776	0.504985075	0.764125594	only in a1H	only in a1H
P98156	Very low-density lipoprotein receptor OS=Mus musculus OX=10090 GN=Vldlr PE=1 SV=1 - [VLDLR_MOUSE]	Vldlr	0	0	0	0	0	10.81007949	0	3.603359828	0	6.241202301	0.504985075	0.764125594	only in a1H	only in a1H
Q00731	Vascular endothelial growth factor A OS=Mus musculus OX=10090 GN=Vegfa PE=1 SV=2 - [VEGFA_MOUSE]	Vegfa	0	0	0	0	0	58.22379392	0	19.40793131	0	33.61552309	0.504985075	0.764125594	only in a1H	only in a1H
Q00PI9	Heterogeneous nuclear ribonucleoprotein U-like protein 2 OS=Mus musculus OX=10090 GN=Hnrnpul2 PE=1 SV=2 - [HNRL2_MOUSE]	Hnrnpul2	0	0	0	10.37308552	0	0	0	3.457695173	0	5.988903717	0.504985075	0.764125594	only in a1H	only in a1H
Q03157	Amyloid-like protein 1 OS=Mus musculus OX=10090 GN=Aplp1 PE=1 SV=1 - [APLP1_MOUSE]	Aplp1	0	0	0	0	0	10.68363214	0	3.561210715	0	6.168197894	0.504985075	0.764125594	only in a1H	only in a1H
Q04859	Serine/threonine-protein kinase MAK OS=Mus musculus OX=10090 GN=Mak PE=1 SV=2 - [MAK_MOUSE]	Mak	0	0	0	0	86.86104629	0	0	28.9536821	0	50.14924846	0.504985075	0.764125594	only in a1H	only in a1H
Q05860	Formin-1 OS=Mus musculus OX=10090 GN=Fmn1 PE=1 SV=2 - [FMN1_MOUSE]	Fmn1	0	0	0	51.42132733	0	0	0	17.14044244	0	29.68811718	0.504985075	0.764125594	only in a1H	only in a1H
Q05CL8	La-related protein 7 OS=Mus musculus OX=10090 GN=Larp7 PE=1 SV=2 - [LARP7_MOUSE]	Larp7	0	0	0	75.00169252	0	0	0	25.00056417	0	43.30224736	0.504985075	0.764125594	only in a1H	only in a1H
Q08890	Iduronate 2-sulfatase OS=Mus musculus OX=10090 GN=Ids PE=2 SV=3 - [IDS_MOUSE]	Ids	0	0	0	0	9.298440679	0	0	3.099480226	0	5.368457229	0.504985075	0.764125594	only in a1H	only in a1H
Q0VGB7	Serine/threonine-protein phosphatase 4 regulatory subunit 2 OS=Mus musculus OX=10090 GN=Ppp4r2 PE=1 SV=1 - [PP4R2_MOUSE]	Ppp4r2	0	0	0	0	0	63.48722756	0	21.16240919	0	36.65436792	0.504985075	0.764125594	only in a1H	only in a1H
Q149F3	Eukaryotic peptide chain release factor GTP-binding subunit ERF3B OS=Mus musculus OX=10090 GN=Gspt2 PE=1 SV=1 - [ERF3B_MOUSE]	Gspt2	0	0	0	16.16400975	0	0	0	5.388003251	0	9.332295383	0.504985075	0.764125594	only in a1H	only in a1H
Q14C51	Pentatricopeptide repeat domain-containing protein 3, mitochondrial OS=Mus musculus OX=10090 GN=Ptcd3 PE=1 SV=2 - [PTCD3_MOUSE]	Ptcd3	0	0	0	47.88287702	0	0	0	15.96095901	0	27.64519194	0.504985075	0.764125594	only in a1H	only in a1H
Q2PZL6	Protocadherin Fat 4 OS=Mus musculus OX=10090 GN=Fat4 PE=1 SV=2 - [FAT4_MOUSE]	Fat4	0	0	0	0	4.225019762	0	0	1.408339921	0	2.439316297	0.504985075	0.764125594	only in a1H	only in a1H
Q3TCJ1	BRISC complex subunit Abraxas 2 OS=Mus musculus OX=10090 GN=Abraxas2 PE=1 SV=1 - [ABRX2_MOUSE]	Abraxas2	0	0	0	0	8.305036093	0	0	2.768345364	0	4.794914824	0.504985075	0.764125594	only in a1H	only in a1H
Q3TDN0	Protein dispatched homolog 1 OS=Mus musculus OX=10090 GN=Disp1 PE=1 SV=2 - [DISP1_MOUSE]	Disp1	0	0	0	0	2.36857546	0	0	0.789525153	0	1.36749768	0.504985075	0.764125594	only in a1H	only in a1H
Q3TKY6	Spliceosome-associated protein CWC27 homolog OS=Mus musculus OX=10090 GN=Cwc27 PE=1 SV=1 - [CWC27_MOUSE]	Cwc27	0	0	0	0	0	4.36358946	0	1.45452982	0	2.519319549	0.504985075	0.764125594	only in a1H	only in a1H
Q3U4G3	Xyloside xylosyltransferase 1 OS=Mus musculus OX=10090 GN=Xxylt1 PE=1 SV=2 - [XXLT1_MOUSE]	Xxylt1	0	0	0	0	34.67546058	0	0	11.55848686	0	20.0198865	0.504985075	0.764125594	only in a1H	only in a1H
Q3U5Q7	UMP-CMP kinase 2, mitochondrial OS=Mus musculus OX=10090 GN=Cmpk2 PE=1 SV=2 - [CMPK2_MOUSE]	Cmpk2	0	0	0	95.70256477	0	0	0	31.90085492	0	55.25390153	0.504985075	0.764125594	only in a1H	only in a1H
Q3UHX2	28 kDa heat- and acid-stable phosphoprotein OS=Mus musculus OX=10090 GN=Pdap1 PE=1 SV=1 - [HAP28_MOUSE]	Pdap1	0	0	0	2.36333096	0	0	0	0.787776987	0	1.364469766	0.504985075	0.764125594	only in a1H	only in a1H
Q3UIK4	N6-adenosine-methyltransferase non-catalytic subunit OS=Mus musculus OX=10090 GN=Mettl14 PE=1 SV=1 - [MET14_MOUSE]	Mettl14	0	0	0	0	2.58041264	0	0	0.860137547	0	1.489801933	0.504985075	0.764125594	only in a1H	only in a1H
Q3UMW8	Ceroid-lipofuscinosis neuronal protein 5 homolog OS=Mus musculus OX=10090 GN=Cln5 PE=1 SV=1 - [CLN5_MOUSE]	Cln5	0	0	0	0	13.12159858	0	0	4.373866192	0	7.575758471	0.504985075	0.764125594	only in a1H	only in a1H
Q3UYG8	ADP-ribose glycohydrolase MACROD2 OS=Mus musculus OX=10090 GN=Macrod2 PE=1 SV=1 - [MACD2_MOUSE]	Macrod2	0	0	0	31.78070585	0	0	0	10.59356862	0	18.34859908	0.504985075	0.764125594	only in a1H	only in a1H
Q3V0K9	Plastin-1 OS=Mus musculus OX=10090 GN=Pls1 PE=1 SV=1 - [PLSI_MOUSE]	Pls1	0	0	0	0	55.26847492	0	0	18.42282497	0	31.90926888	0.504985075	0.764125594	only in a1H	only in a1H
Q497V5	S1 RNA-binding domain-containing protein 1 OS=Mus musculus OX=10090 GN=Srbd1 PE=2 SV=3 - [SRBD1_MOUSE]	Srbd1	0	0	0	0	58.19586807	0	0	19.39862269	0	33.5994001	0.504985075	0.764125594	only in a1H	only in a1H
Q4FZG7	Putative mitochondrial import inner membrane translocase subunit Tim8 A-B OS=Mus musculus OX=10090 GN=Timm8a2 PE=3 SV=1 - [TI8AB_MOUSE]	Timm8a2	0	0	0	45.08769338	0	0	0	15.02923113	0	26.03139191	0.504985075	0.764125594	only in a1H	only in a1H
Q5SSW2	Proteasome activator complex subunit 4 OS=Mus musculus OX=10090 GN=Psme4 PE=1 SV=1 - [PSME4_MOUSE]	Psme4	0	0	0	0	5.327861217	0	0	1.775953739	0	3.076042108	0.504985075	0.764125594	only in a1H	only in a1H
Q5STE3	Follistatin-related protein 4 OS=Mus musculus OX=10090 GN=Fstl4 PE=2 SV=1 - [FSTL4_MOUSE]	Fstl4	0	0	0	0	4.162925154	0	0	1.387641718	0	2.403465958	0.504985075	0.764125594	only in a1H	only in a1H
Q5SUF2	Luc7-like protein 3 OS=Mus musculus OX=10090 GN=Luc7l3 PE=1 SV=1 - [LC7L3_MOUSE]	Luc7l3	0	0	0	23.7788386	0	0	0	7.926279534	0	13.72871887	0.504985075	0.764125594	only in a1H	only in a1H
Q5SW19	Clustered mitochondria protein homolog OS=Mus musculus OX=10090 GN=Cluh PE=1 SV=2 - [CLU_MOUSE]	Cluh	0	0	0	13.38471378	0	0	0	4.46157126	0	7.727668103	0.504985075	0.764125594	only in a1H	only in a1H
Q60520	Paired amphipathic helix protein Sin3a OS=Mus musculus OX=10090 GN=Sin3a PE=1 SV=3 - [SIN3A_MOUSE]	Sin3a	0	0	0	17.54794112	0	0	0	5.849313706	0	10.13130853	0.504985075	0.764125594	only in a1H	only in a1H
Q60648	Ganglioside GM2 activator OS=Mus musculus OX=10090 GN=Gm2a PE=1 SV=2 - [SAP3_MOUSE]	Gm2a	0	0	0	23.08265567	0	0	0	7.694218557	0	13.32677747	0.504985075	0.764125594	only in a1H	only in a1H
Q60710	Deoxynucleoside triphosphate triphosphohydrolase SAMHD1 OS=Mus musculus OX=10090 GN=Samhd1 PE=1 SV=3 - [SAMH1_MOUSE]	Samhd1	0	0	0	10.56609826	0	0	0	3.522032755	0	6.100339677	0.504985075	0.764125594	only in a1H	only in a1H
Q60714	Long-chain fatty acid transport protein 1 OS=Mus musculus OX=10090 GN=Slc27a1 PE=1 SV=1 - [S27A1_MOUSE]	Slc27a1	0	0	0	0	15.00875091	0	0	5.002916969	0	8.665306376	0.504985075	0.764125594	only in a1H	only in a1H
Q60899	ELAV-like protein 2 OS=Mus musculus OX=10090 GN=Elavl2 PE=1 SV=1 - [ELAV2_MOUSE]	Elavl2	0	0	0	0	19.77261575	0	0	6.590871916	0	11.41572502	0.504985075	0.764125594	only in a1H	only in a1H
Q61074	Protein phosphatase 1G OS=Mus musculus OX=10090 GN=Ppm1g PE=1 SV=3 - [PPM1G_MOUSE]	Ppm1g	0	0	0	0	22.35958349	0	0	7.453194495	0	12.90931154	0.504985075	0.764125594	only in a1H	only in a1H
Q61136	Serine/threonine-protein kinase PRP4 homolog OS=Mus musculus OX=10090 GN=Prpf4b PE=1 SV=3 - [PRP4B_MOUSE]	Prpf4b	0	0	0	8.083213006	0	0	0	2.694404335	0	4.666845205	0.504985075	0.764125594	only in a1H	only in a1H
Q61183	Poly(A) polymerase alpha OS=Mus musculus OX=10090 GN=Papola PE=1 SV=4 - [PAPOA_MOUSE]	Papola	0	0	0	0	21.43847446	0	0	7.146158155	0	12.377509	0.504985075	0.764125594	only in a1H	only in a1H
Q61285	ATP-binding cassette sub-family D member 2 OS=Mus musculus OX=10090 GN=Abcd2 PE=1 SV=1 - [ABCD2_MOUSE]	Abcd2	0	0	0	0	29.17806896	0	0	9.726022986	0	16.84596597	0.504985075	0.764125594	only in a1H	only in a1H
Q61477	Neuroblastoma suppressor of tumorigenicity 1 OS=Mus musculus OX=10090 GN=Nbl1 PE=1 SV=2 - [NBL1_MOUSE]	Nbl1	0	0	0	0	106.2759375	0	0	35.42531251	0	61.35844114	0.504985075	0.764125594	only in a1H	only in a1H
Q61584	Fragile X mental retardation syndrome-related protein 1 OS=Mus musculus OX=10090 GN=Fxr1 PE=1 SV=2 - [FXR1_MOUSE]	Fxr1	0	0	0	0	18.45799105	0	0	6.152663684	0	10.6567261	0.504985075	0.764125594	only in a1H	only in a1H
Q61790	Lymphocyte activation gene 3 protein OS=Mus musculus OX=10090 GN=Lag3 PE=1 SV=1 - [LAG3_MOUSE]	Lag3	0	0	0	3.406513161	0	0	0	1.135504387	0	1.96675129	0.504985075	0.764125594	only in a1H	only in a1H
Q61805	Lipopolysaccharide-binding protein OS=Mus musculus OX=10090 GN=Lbp PE=1 SV=2 - [LBP_MOUSE]	Lbp	0	0	0	8.843748639	0	0	0	2.947916213	0	5.105940657	0.504985075	0.764125594	only in a1H	only in a1H
Q61830	Macrophage mannose receptor 1 OS=Mus musculus OX=10090 GN=Mrc1 PE=1 SV=2 - [MRC1_MOUSE]	Mrc1	0	0	0	4.917771206	0	0	0	1.639257069	0	2.83927653	0.504985075	0.764125594	only in a1H	only in a1H
Q61982	Neurogenic locus notch homolog protein 3 OS=Mus musculus OX=10090 GN=Notch3 PE=1 SV=1 - [NOTC3_MOUSE]	Notch3	0	0	0	0	26.74769001	0	0	8.915896669	0	15.44278603	0.504985075	0.764125594	only in a1H	only in a1H
Q62036	Centrosomal protein of 131 kDa OS=Mus musculus OX=10090 GN=Cep131 PE=1 SV=2 - [CP131_MOUSE]	Cep131	0	0	0	19.52115949	0	0	0	6.507053163	0	11.27054669	0.504985075	0.764125594	only in a1H	only in a1H
Q62093	Serine/arginine-rich splicing factor 2 OS=Mus musculus OX=10090 GN=Srsf2 PE=1 SV=4 - [SRSF2_MOUSE]	Srsf2	0	0	0	0	72.59889832	0	0	24.19963277	0	41.91499349	0.504985075	0.764125594	only in a1H	only in a1H
Q62095	ATP-dependent RNA helicase DDX3Y OS=Mus musculus OX=10090 GN=Ddx3y PE=1 SV=2 - [DDX3Y_MOUSE]	Ddx3y	0	0	0	55.31862278	0	0	0	18.43954093	0	31.93822175	0.504985075	0.764125594	only in a1H	only in a1H
Q62189	U1 small nuclear ribonucleoprotein A OS=Mus musculus OX=10090 GN=Snrpa PE=1 SV=3 - [SNRPA_MOUSE]	Snrpa	0	0	0	0	90.16434819	0	0	30.05478273	0	52.0564107	0.504985075	0.764125594	only in a1H	only in a1H
Q62351	Transferrin receptor protein 1 OS=Mus musculus OX=10090 GN=Tfrc PE=1 SV=1 - [TFR1_MOUSE]	Tfrc	0	0	0	0	11.95442239	0	0	3.984807465	0	6.901888988	0.504985075	0.764125594	only in a1H	only in a1H
Q62419	Endophilin-A2 OS=Mus musculus OX=10090 GN=Sh3gl1 PE=1 SV=1 - [SH3G1_MOUSE]	Sh3gl1	0	0	0	0	9.848880339	0	0	3.282960113	0	5.686253715	0.504985075	0.764125594	only in a1H	only in a1H
Q63870	Collagen alpha-1(VII) chain OS=Mus musculus OX=10090 GN=Col7a1 PE=1 SV=3 - [CO7A1_MOUSE]	Col7a1	0	0	0	0	70.14808478	0	0	23.38269493	0	40.50001563	0.504985075	0.764125594	only in a1H	only in a1H
Q63912	Oligodendrocyte-myelin glycoprotein OS=Mus musculus OX=10090 GN=Omg PE=1 SV=1 - [OMGP_MOUSE]	Omg	0	0	0	14.96197487	0	0	0	4.987324956	0	8.638300218	0.504985075	0.764125594	only in a1H	only in a1H
Q64096	Guanine nucleotide exchange factor DBS OS=Mus musculus OX=10090 GN=Mcf2l PE=1 SV=2 - [MCF2L_MOUSE]	Mcf2l	0	0	0	0	16.70336142	0	0	5.567787141	0	9.643690214	0.504985075	0.764125594	only in a1H	only in a1H
Q64282	Interferon-induced protein with tetratricopeptide repeats 1 OS=Mus musculus OX=10090 GN=Ifit1 PE=1 SV=2 - [IFIT1_MOUSE]	Ifit1	0	0	0	23.76883385	0	0	0	7.922944617	0	13.72294262	0.504985075	0.764125594	only in a1H	only in a1H
Q64345	Interferon-induced protein with tetratricopeptide repeats 3 OS=Mus musculus OX=10090 GN=Ifit3 PE=1 SV=1 - [IFIT3_MOUSE]	Ifit3	0	0	0	32.15695153	0	0	0	10.71898384	0	18.56582462	0.504985075	0.764125594	only in a1H	only in a1H
Q64511	DNA topoisomerase 2-beta OS=Mus musculus OX=10090 GN=Top2b PE=1 SV=2 - [TOP2B_MOUSE]	Top2b	0	0	0	0	7.276118033	0	0	2.425372678	0	4.200868705	0.504985075	0.764125594	only in a1H	only in a1H
Q68FF6	ARF GTPase-activating protein GIT1 OS=Mus musculus OX=10090 GN=Git1 PE=1 SV=1 - [GIT1_MOUSE]	Git1	0	0	0	8.936428574	0	0	0	2.978809525	0	5.159449443	0.504985075	0.764125594	only in a1H	only in a1H
Q68SA9	A disintegrin and metalloproteinase with thrombospondin motifs 7 OS=Mus musculus OX=10090 GN=Adamts7 PE=1 SV=3 - [ATS7_MOUSE]	Adamts7	0	0	0	0	6.01243589	0	0	2.004145297	0	3.47128148	0.504985075	0.764125594	only in a1H	only in a1H
Q6IQX7	Chondroitin sulfate synthase 2 OS=Mus musculus OX=10090 GN=Chpf PE=1 SV=1 - [CHSS2_MOUSE]	Chpf	0	0	0	0	86.6060315	0	0	28.86867717	0	50.0020156	0.504985075	0.764125594	only in a1H	only in a1H
Q6KCD5	Nipped-B-like protein OS=Mus musculus OX=10090 GN=Nipbl PE=1 SV=1 - [NIPBL_MOUSE]	Nipbl	0	0	0	16.6700347	0	0	0	5.556678233	0	9.624449021	0.504985075	0.764125594	only in a1H	only in a1H
Q6P2K6	Serine/threonine-protein phosphatase 4 regulatory subunit 3A OS=Mus musculus OX=10090 GN=Ppp4r3a PE=1 SV=1 - [P4R3A_MOUSE]	Ppp4r3a	0	0	0	13.65775175	0	0	0	4.552583915	0	7.885306647	0.504985075	0.764125594	only in a1H	only in a1H
Q6P5D8	Structural maintenance of chromosomes flexible hinge domain-containing protein 1 OS=Mus musculus OX=10090 GN=Smchd1 PE=1 SV=2 - [SMHD1_MOUS	Smchd1	0	0	0	0	7.431330709	0	0	2.477110236	0	4.290480785	0.504985075	0.764125594	only in a1H	only in a1H
Q6P5G6	UBX domain-containing protein 7 OS=Mus musculus OX=10090 GN=Ubxn7 PE=1 SV=2 - [UBXN7_MOUSE]	Ubxn7	0	0	0	0	13.57467921	0	0	4.524893072	0	7.837344699	0.504985075	0.764125594	only in a1H	only in a1H
Q6P8M1	Putative deoxyribonuclease TATDN1 OS=Mus musculus OX=10090 GN=Tatdn1 PE=1 SV=1 - [TATD1_MOUSE]	Tatdn1	0	0	0	0	13.73260639	0	0	4.577535464	0	7.928523997	0.504985075	0.764125594	only in a1H	only in a1H
Q6PGH2	Jupiter microtubule associated homolog 2 OS=Mus musculus OX=10090 GN=Jpt2 PE=1 SV=1 - [JUPI2_MOUSE]	Jpt2	0	0	0	70.75683217	0	0	0	23.58561072	0	40.8514761	0.504985075	0.764125594	only in a1H	only in a1H
Q6Q899	Antiviral innate immune response receptor RIG-I OS=Mus musculus OX=10090 GN=Ddx58 PE=1 SV=2 - [DDX58_MOUSE]	Ddx58	0	0	0	35.3271857	0	0	0	11.77572857	0	20.39616017	0.504985075	0.764125594	only in a1H	only in a1H
Q6V4S5	Protein sidekick-2 OS=Mus musculus OX=10090 GN=Sdk2 PE=1 SV=1 - [SDK2_MOUSE]	Sdk2	0	0	0	0	0	5.17982227	0	1.726607423	0	2.990571782	0.504985075	0.764125594	only in a1H	only in a1H
Q6V595	Kelch-like protein 6 OS=Mus musculus OX=10090 GN=Klhl6 PE=2 SV=2 - [KLHL6_MOUSE]	Klhl6	0	0	0	0	115.921404	0	0	38.64046801	0	66.92725382	0.504985075	0.764125594	only in a1H	only in a1H
Q6ZQ06	Centrosomal protein of 162 kDa OS=Mus musculus OX=10090 GN=Cep162 PE=1 SV=2 - [CE162_MOUSE]	Cep162	0	0	0	0	0	93666.28651	0	31222.0955	0	54078.25573	0.504985075	0.764125594	only in a1H	only in a1H
Q6ZWV3	60S ribosomal protein L10 OS=Mus musculus OX=10090 GN=Rpl10 PE=1 SV=3 - [RL10_MOUSE]	Rpl10	0	0	0	0	73.73989037	0	0	24.57996346	0	42.57374555	0.504985075	0.764125594	only in a1H	only in a1H
Q6ZWZ2	Ubiquitin-conjugating enzyme E2 R2 OS=Mus musculus OX=10090 GN=Ube2r2 PE=1 SV=1 - [UB2R2_MOUSE]	Ube2r2	0	0	0	0	17.50775325	0	0	5.83591775	0	10.10810605	0.504985075	0.764125594	only in a1H	only in a1H
Q71RI9	Kynurenine--oxoglutarate transaminase 3 OS=Mus musculus OX=10090 GN=Kyat3 PE=1 SV=1 - [KAT3_MOUSE]	Kyat3	0	0	0	8.360663904	0	0	0	2.786887968	0	4.827031556	0.504985075	0.764125594	only in a1H	only in a1H
Q78YZ6	Short coiled-coil protein OS=Mus musculus OX=10090 GN=Scoc PE=1 SV=1 - [SCOC_MOUSE]	Scoc	0	0	0	0	23.14417808	0	0	7.714726028	0	13.36229745	0.504985075	0.764125594	only in a1H	only in a1H
Q7TMS5	Broad substrate specificity ATP-binding cassette transporter ABCG2 OS=Mus musculus OX=10090 GN=Abcg2 PE=1 SV=1 - [ABCG2_MOUSE]	Abcg2	0	0	0	0	0	8.756603583	0	2.918867861	0	5.055627436	0.504985075	0.764125594	only in a1H	only in a1H
Q7TPD0	Integrator complex subunit 3 OS=Mus musculus OX=10090 GN=Ints3 PE=1 SV=2 - [INT3_MOUSE]	Ints3	0	0	0	0	29.81592225	0	0	9.938640749	0	17.21423074	0.504985075	0.764125594	only in a1H	only in a1H
Q7TPM1	Protein PRRC2B OS=Mus musculus OX=10090 GN=Prrc2b PE=1 SV=1 - [PRC2B_MOUSE]	Prrc2b	0	0	0	0	684.760049	0	0	228.2533497	0	395.3463986	0.504985075	0.764125594	only in a1H	only in a1H
Q7TPV4	Myb-binding protein 1A OS=Mus musculus OX=10090 GN=Mybbp1a PE=1 SV=2 - [MBB1A_MOUSE]	Mybbp1a	0	0	0	36.56002133	0	0	0	12.18667378	0	21.10793816	0.504985075	0.764125594	only in a1H	only in a1H
Q7TQK1	Integrator complex subunit 7 OS=Mus musculus OX=10090 GN=Ints7 PE=1 SV=1 - [INT7_MOUSE]	Ints7	0	0	0	0	34.49882603	0	0	11.49960868	0	19.9179065	0.504985075	0.764125594	only in a1H	only in a1H
Q7TSH2	Phosphorylase b kinase regulatory subunit beta OS=Mus musculus OX=10090 GN=Phkb PE=1 SV=1 - [KPBB_MOUSE]	Phkb	0	0	0	0	10.12689719	0	0	3.375632398	0	5.846766821	0.504985075	0.764125594	only in a1H	only in a1H
Q7TSH6	SR-related and CTD-associated factor 4 OS=Mus musculus OX=10090 GN=Scaf4 PE=1 SV=1 - [SCAF4_MOUSE]	Scaf4	0	0	0	0	26.79520628	0	0	8.931735428	0	15.47021956	0.504985075	0.764125594	only in a1H	only in a1H
Q80TM9	Nischarin OS=Mus musculus OX=10090 GN=Nisch PE=1 SV=2 - [NISCH_MOUSE]	Nisch	0	0	0	0	9.966396889	0	0	3.322132296	0	5.754101926	0.504985075	0.764125594	only in a1H	only in a1H
Q80TN7	Neuron navigator 3 OS=Mus musculus OX=10090 GN=Nav3 PE=1 SV=2 - [NAV3_MOUSE]	Nav3	0	0	0	673.2772892	0	0	0	224.4257631	0	388.7168242	0.504985075	0.764125594	only in a1H	only in a1H
Q80U78	Pumilio homolog 1 OS=Mus musculus OX=10090 GN=Pum1 PE=1 SV=2 - [PUM1_MOUSE]	Pum1	0	0	0	0	2.906894493	0	0	0.968964831	0	1.678296318	0.504985075	0.764125594	only in a1H	only in a1H
Q80VJ3	2'-deoxynucleoside 5'-phosphate N-hydrolase 1 OS=Mus musculus OX=10090 GN=Dnph1 PE=1 SV=2 - [DNPH1_MOUSE]	Dnph1	0	0	0	0	52.56544211	0	0	17.52181404	0	30.34867215	0.504985075	0.764125594	only in a1H	only in a1H
Q80WQ2	Protein VAC14 homolog OS=Mus musculus OX=10090 GN=Vac14 PE=1 SV=1 - [VAC14_MOUSE]	Vac14	0	0	0	0	7.152674743	0	0	2.384224914	0	4.129598688	0.504985075	0.764125594	only in a1H	only in a1H

Q80X82	Symplekin OS=Mus musculus OX=10090 GN=Sympk PE=1 SV=2 - [SYMPK_MOUSE]	Sympk	0	0	0	4.832409246	0	0	0	1.610803082	0	2.789992779	0.504985075	0.764125594	only in a1H	only in a1H
Q80Y17	Lethal(2) giant larvae protein homolog 1 OS=Mus musculus OX=10090 GN=Llgl1 PE=1 SV=1 - [L2GL1_MOUSE]	Llgl1	0	0	0	0	12.80589873	0	0	4.26863291	0	7.393489079	0.504985075	0.764125594	only in a1H	only in a1H
Q80YN3	Breast carcinoma-amplified sequence 1 homolog OS=Mus musculus OX=10090 GN=Bcas1 PE=1 SV=3 - [BCAS1_MOUSE]	Bcas1	0	0	0	0	19.4063593	0	0	6.468786432	0	11.20426676	0.504985075	0.764125594	only in a1H	only in a1H
Q810A7	ATP-dependent RNA helicase DDX42 OS=Mus musculus OX=10090 GN=Ddx42 PE=1 SV=2 - [DDX42_MOUSE]	Ddx42	0	0	0	0	18.87223499	0	0	6.290744998	0	10.89588995	0.504985075	0.764125594	only in a1H	only in a1H
Q810U3	Neurofascin OS=Mus musculus OX=10090 GN=Nfasc PE=1 SV=1 - [NFASC_MOUSE]	Nfasc	0	0	0	0	12.54385974	0	0	4.18128658	0	7.242200797	0.504985075	0.764125594	only in a1H	only in a1H
Q8BFU3	RING finger protein 214 OS=Mus musculus OX=10090 GN=Rnf214 PE=1 SV=1 - [RN214_MOUSE]	Rnf214	0	0	0	4.970925641	0	0	0	1.656975214	0	2.869965257	0.504985075	0.764125594	only in a1H	only in a1H
Q8BGS2	BolA-like protein 2 OS=Mus musculus OX=10090 GN=Bola2 PE=1 SV=1 - [BOLA2_MOUSE]	Bola2	0	0	0	0	71.62939916	0	0	23.87646639	0	41.35525289	0.504985075	0.764125594	only in a1H	only in a1H
Q8BGT8	Phytanoyl-CoA hydroxylase-interacting protein-like OS=Mus musculus OX=10090 GN=Phyhipl PE=1 SV=1 - [PHIPL_MOUSE]	Phyhipl	0	0	0	42.93628055	0	0	0	14.31209352	0	24.78927313	0.504985075	0.764125594	only in a1H	only in a1H
Q8BGW1	Alpha-ketoglutarate-dependent dioxygenase FTO OS=Mus musculus OX=10090 GN=Fto PE=1 SV=1 - [FTO_MOUSE]	Fto	0	0	0	3.447669723	0	0	0	1.149223241	0	1.990513043	0.504985075	0.764125594	only in a1H	only in a1H
Q8BH43	Wiskott-Aldrich syndrome protein family member 2 OS=Mus musculus OX=10090 GN=Wasf2 PE=1 SV=1 - [WASF2_MOUSE]	Wasf2	0	0	0	0	15.8872529	0	0	5.295750965	0	9.172509736	0.504985075	0.764125594	only in a1H	only in a1H
Q8BH53	Cilia- and flagella-associated protein 69 OS=Mus musculus OX=10090 GN=Cfap69 PE=1 SV=1 - [CFA69_MOUSE]	Cfap69	0	0	0	3.226956318	0	0	0	1.075652106	0	1.863084099	0.504985075	0.764125594	only in a1H	only in a1H
Q8BK63	Casein kinase I isoform alpha OS=Mus musculus OX=10090 GN=Csnk1a1 PE=1 SV=2 - [KC1A_MOUSE]	Csnk1a1	0	0	0	0	10.58711893	0	0	3.529039643	0	6.112475964	0.504985075	0.764125594	only in a1H	only in a1H
Q8BKV1	Glypican-2 OS=Mus musculus OX=10090 GN=Gpc2 PE=2 SV=1 - [GPC2_MOUSE]	Gpc2	0	0	0	0	7.170575382	0	0	2.390191794	0	4.139933627	0.504985075	0.764125594	only in a1H	only in a1H
Q8BL66	Early endosome antigen 1 OS=Mus musculus OX=10090 GN=Eea1 PE=1 SV=2 - [EEA1_MOUSE]	Eea1	0	0	0	0	24.92170129	0	0	8.307233764	0	14.38855095	0.504985075	0.764125594	only in a1H	only in a1H
Q8BP56	Protein-glucosylgalactosylhydroxylysine glucosidase OS=Mus musculus OX=10090 GN=Pgghg PE=1 SV=1 - [PGGHG_MOUSE]	Pgghg	0	0	0	21.74618772	0	0	0	7.24872924	0	12.55516733	0.504985075	0.764125594	only in a1H	only in a1H
Q8BQ89	LIX1-like protein OS=Mus musculus OX=10090 GN=Lix1l PE=2 SV=2 - [LIX1L_MOUSE]	Lix1l	0	0	0	0	0	24.0238193	0	8.007939766	0	13.87015854	0.504985075	0.764125594	only in a1H	only in a1H
Q8BSF5	Kelch-like protein 38 OS=Mus musculus OX=10090 GN=Klhl38 PE=2 SV=1 - [KLH38_MOUSE]	Klhl38	0	0	0	38.31003556	0	0	0	12.77001185	0	22.11830934	0.504985075	0.764125594	only in a1H	only in a1H
Q8BTS4	Nuclear pore complex protein Nup54 OS=Mus musculus OX=10090 GN=Nup54 PE=1 SV=1 - [NUP54_MOUSE]	Nup54	0	0	0	0	16.84921152	0	0	5.61640384	0	9.727896807	0.504985075	0.764125594	only in a1H	only in a1H
Q8BWS5	G protein-regulated inducer of neurite outgrowth 3 OS=Mus musculus OX=10090 GN=Gprin3 PE=1 SV=1 - [GRIN3_MOUSE]	Gprin3	0	0	0	65.05703803	0	0	0	21.68567934	0	37.56069842	0.504985075	0.764125594	only in a1H	only in a1H
Q8BWT1	3-ketoacyl-CoA thiolase, mitochondrial OS=Mus musculus OX=10090 GN=Acaa2 PE=1 SV=3 - [THIM_MOUSE]	Acaa2	0	0	0	0	19.95541481	0	0	6.651804937	0	11.52126411	0.504985075	0.764125594	only in a1H	only in a1H
Q8C2S5	F-box/LRR-repeat protein 5 OS=Mus musculus OX=10090 GN=Fbxl5 PE=2 SV=2 - [FBXL5_MOUSE]	Fbxl5	0	0	0	0	0	25.68366237	0	8.561220789	0	14.82846938	0.504985075	0.764125594	only in a1H	only in a1H
Q8C5L3	CCR4-NOT transcription complex subunit 2 OS=Mus musculus OX=10090 GN=Cnot2 PE=1 SV=2 - [CNOT2_MOUSE]	Cnot2	0	0	0	0	7.721010706	0	0	2.573670235	0	4.45772761	0.504985075	0.764125594	only in a1H	only in a1H
Q8CCH2	NHL repeat-containing protein 3 OS=Mus musculus OX=10090 GN=Nhlrc3 PE=1 SV=1 - [NHLC3_MOUSE]	Nhlrc3	0	0	0	0	12.25595196	0	0	4.085317321	0	7.075977165	0.504985075	0.764125594	only in a1H	only in a1H
Q8CDM1	ATPase family AAA domain-containing protein 2 OS=Mus musculus OX=10090 GN=Atad2 PE=1 SV=1 - [ATAD2_MOUSE]	Atad2	0	0	0	6.420013111	0	0	0	2.14000437	0	3.706596298	0.504985075	0.764125594	only in a1H	only in a1H
Q8CGA0	Protein phosphatase 1F OS=Mus musculus OX=10090 GN=Ppm1f PE=1 SV=1 - [PPM1F_MOUSE]	Ppm1f	0	0	0	29.72470538	0	0	0	9.908235127	0	17.16156665	0.504985075	0.764125594	only in a1H	only in a1H
Q8JZP2	Synapsin-3 OS=Mus musculus OX=10090 GN=Syn3 PE=1 SV=2 - [SYN3_MOUSE]	Syn3	0	0	0	0	81.66880757	0	0	27.22293586	0	47.15150804	0.504985075	0.764125594	only in a1H	only in a1H
Q8K1R7	Serine/threonine-protein kinase Nek9 OS=Mus musculus OX=10090 GN=Nek9 PE=1 SV=2 - [NEK9_MOUSE]	Nek9	0	0	0	0	22.63983009	0	0	7.54661003	0	13.071112	0.504985075	0.764125594	only in a1H	only in a1H
Q8K221	Arfaptin-2 OS=Mus musculus OX=10090 GN=Arfip2 PE=1 SV=2 - [ARFP2_MOUSE]	Arfip2	0	0	0	3.631706132	0	0	0	1.210568711	0	2.096766513	0.504985075	0.764125594	only in a1H	only in a1H
Q8K274	Ketosamine-3-kinase OS=Mus musculus OX=10090 GN=Fn3krp PE=1 SV=2 - [KT3K_MOUSE]	Fn3krp	0	0	0	0	6.454945201	0	0	2.1516484	0	3.726764349	0.504985075	0.764125594	only in a1H	only in a1H
Q8K411	Presequence protease, mitochondrial OS=Mus musculus OX=10090 GN=Pitrm1 PE=1 SV=1 - [PREP_MOUSE]	Pitrm1	0	0	0	6.656918515	0	0	0	2.218972838	0	3.843373696	0.504985075	0.764125594	only in a1H	only in a1H
Q8R0F8	Acylpyruvase FAHD1, mitochondrial OS=Mus musculus OX=10090 GN=Fahd1 PE=1 SV=2 - [FAHD1_MOUSE]	Fahd1	0	0	0	14.62971848	0	0	0	4.876572827	0	8.446471903	0.504985075	0.764125594	only in a1H	only in a1H
Q8R1N0	Zinc finger protein 830 OS=Mus musculus OX=10090 GN=Znf830 PE=1 SV=1 - [ZN830_MOUSE]	Znf830	0	0	0	10.0739671	0	0	0	3.357989032	0	5.816207615	0.504985075	0.764125594	only in a1H	only in a1H
Q8R2R9	AP-3 complex subunit mu-2 OS=Mus musculus OX=10090 GN=Ap3m2 PE=1 SV=1 - [AP3M2_MOUSE]	Ap3m2	0	0	0	0	53.67234802	0	0	17.89078267	0	30.98774458	0.504985075	0.764125594	only in a1H	only in a1H
Q8R2Z5	von Willebrand factor A domain-containing protein 1 OS=Mus musculus OX=10090 GN=Vwa1 PE=1 SV=1 - [VWA1_MOUSE]	Vwa1	0	0	0	0	0	6.426626046	0	2.142208682	0	3.710414278	0.504985075	0.764125594	only in a1H	only in a1H
Q8R3B1	1-phosphatidylinositol 4,5-bisphosphate phosphodiesterase delta-1 OS=Mus musculus OX=10090 GN=Plcd1 PE=1 SV=2 - [PLCD1_MOUSE]	Plcd1	0	0	0	3.994449943	0	0	0	1.331483314	0	2.30619675	0.504985075	0.764125594	only in a1H	only in a1H
Q8R3R8	Gamma-aminobutyric acid receptor-associated protein-like 1 OS=Mus musculus OX=10090 GN=Gabarapl1 PE=1 SV=2 - [GBRL1_MOUSE]	Gabarapl1	0	0	0	0	9.001176528	0	0	3.000392176	0	5.196831692	0.504985075	0.764125594	only in a1H	only in a1H
Q8R3V5	Endophilin-B2 OS=Mus musculus OX=10090 GN=Sh3glb2 PE=1 SV=2 - [SHLB2_MOUSE]	Sh3glb2	0	0	0	0	15.5017347	0	0	5.1672449	0	8.949930701	0.504985075	0.764125594	only in a1H	only in a1H
Q8R409	Protein HEXIM1 OS=Mus musculus OX=10090 GN=Hexim1 PE=1 SV=1 - [HEXI1_MOUSE]	Hexim1	0	0	0	21.45623292	0	0	0	7.152077638	0	12.38776185	0.504985075	0.764125594	only in a1H	only in a1H
Q8R480	Nuclear pore complex protein Nup85 OS=Mus musculus OX=10090 GN=Nup85 PE=1 SV=1 - [NUP85_MOUSE]	Nup85	0	0	0	0	4.293540826	0	0	1.431180275	0	2.478876952	0.504985075	0.764125594	only in a1H	only in a1H
Q8VE62	Polyadenylate-binding protein-interacting protein 1 OS=Mus musculus OX=10090 GN=Paip1 PE=1 SV=1 - [PAIP1_MOUSE]	Paip1	0	0	0	33.44857923	0	0	0	11.14952641	0	19.31154622	0.504985075	0.764125594	only in a1H	only in a1H
Q8VEJ4	Notchless protein homolog 1 OS=Mus musculus OX=10090 GN=Nle1 PE=1 SV=4 - [NLE1_MOUSE]	Nle1	0	0	0	10.36654903	0	0	0	3.455516344	0	5.985129874	0.504985075	0.764125594	only in a1H	only in a1H
Q8VH51	RNA-binding protein 39 OS=Mus musculus OX=10090 GN=Rbm39 PE=1 SV=2 - [RBM39_MOUSE]	Rbm39	0	0	0	0	40.30126586	0	0	13.43375529	0	23.2679467	0.504985075	0.764125594	only in a1H	only in a1H
Q91V87	Fibroblast growth factor receptor-like 1 OS=Mus musculus OX=10090 GN=Fgfrl1 PE=1 SV=1 - [FGRL1_MOUSE]	Fgfrl1	0	0	0	0	15.040961	0	0	5.013653666	0	8.683902881	0.504985075	0.764125594	only in a1H	only in a1H
Q91VX2	Ubiquitin-associated protein 2 OS=Mus musculus OX=10090 GN=Ubap2 PE=1 SV=1 - [UBAP2_MOUSE]	Ubap2	0	0	0	17.29734883	0	0	0	5.765782945	0	9.986629006	0.504985075	0.764125594	only in a1H	only in a1H
Q91W39	Nuclear receptor coactivator 5 OS=Mus musculus OX=10090 GN=Ncoa5 PE=1 SV=1 - [NCOA5_MOUSE]	Ncoa5	0	0	0	46.58266726	0	0	0	15.52755575	0	26.89451548	0.504985075	0.764125594	only in a1H	only in a1H
Q91WC0	Actin-histidine N-methyltransferase OS=Mus musculus OX=10090 GN=Setd3 PE=1 SV=1 - [SETD3_MOUSE]	Setd3	0	0	0	4.473085848	0	0	0	1.491028616	0	2.582537319	0.504985075	0.764125594	only in a1H	only in a1H
Q91X52	L-xylulose reductase OS=Mus musculus OX=10090 GN=Dcxr PE=1 SV=2 - [DCXR_MOUSE]	Dcxr	0	0	0	23.12077233	0	0	0	7.706924111	0	13.34878413	0.504985075	0.764125594	only in a1H	only in a1H
Q91XZ4	Protocadherin beta-6 OS=Mus musculus OX=10090 GN=Pcdhb6 PE=1 SV=1 - [PCDB6_MOUSE]	Pcdhb6	0	0	0	0	0	30.27960725	0	10.09320242	0	17.4819394	0.504985075	0.764125594	only in a1H	only in a1H
Q920B9	FACT complex subunit SPT16 OS=Mus musculus OX=10090 GN=Supt16h PE=1 SV=2 - [SP16H_MOUSE]	Supt16h	0	0	0	0	31.31010913	0	0	10.43670304	0	18.07689994	0.504985075	0.764125594	only in a1H	only in a1H
Q920R0	Alsin OS=Mus musculus OX=10090 GN=Als2 PE=1 SV=3 - [ALS2_MOUSE]	Als2	0	0	0	169.2020694	0	0	0	56.4006898	0	97.68886032	0.504985075	0.764125594	only in a1H	only in a1H
Q921V5	Alpha-1,6-mannosyl-glycoprotein 2-beta-N-acetylglucosaminyltransferase OS=Mus musculus OX=10090 GN=Mgat2 PE=1 SV=1 - [MGAT2_MOUSE]	Mgat2	0	0	0	21.41691981	0	0	0	7.138973269	0	12.36506442	0.504985075	0.764125594	only in a1H	only in a1H
Q922F4	Tubulin beta-6 chain OS=Mus musculus OX=10090 GN=Tubb6 PE=1 SV=1 - [TBB6_MOUSE]	Tubb6	0	0	0	0	966.8537363	0	0	322.2845788	0	558.2132649	0.504985075	0.764125594	only in a1H	only in a1H
Q922K7	Probable 28S rRNA (cytosine-C(5))-methyltransferase OS=Mus musculus OX=10090 GN=Nop2 PE=1 SV=1 - [NOP2_MOUSE]	Nop2	0	0	0	292.0543556	0	0	0	97.35145188	0	168.6176608	0.504985075	0.764125594	only in a1H	only in a1H
Q922S4	cGMP-dependent 3',5'-cyclic phosphodiesterase OS=Mus musculus OX=10090 GN=Pde2a PE=1 SV=4 - [PDE2A_MOUSE]	Pde2a	0	0	0	59.96483982	0	0	0	19.98827994	0	34.62071641	0.504985075	0.764125594	only in a1H	only in a1H
Q922U1	U4/U6 small nuclear ribonucleoprotein Prp3 OS=Mus musculus OX=10090 GN=Prpf3 PE=1 SV=1 - [PRPF3_MOUSE]	Prpf3	0	0	0	0	11.37995205	0	0	3.793317351	0	6.570218381	0.504985075	0.764125594	only in a1H	only in a1H
Q924X6	Low-density lipoprotein receptor-related protein 8 OS=Mus musculus OX=10090 GN=Lrp8 PE=1 SV=2 - [LRP8_MOUSE]	Lrp8	0	0	0	0	15.79276713	0	0	5.264255711	0	9.117958355	0.504985075	0.764125594	only in a1H	only in a1H
Q99104	Unconventional myosin-Va OS=Mus musculus OX=10090 GN=Myo5a PE=1 SV=2 - [MYO5A_MOUSE]	Myo5a	0	0	0	0	11.0859751	0	0	3.695325033	0	6.400490708	0.504985075	0.764125594	only in a1H	only in a1H
Q99JF5	Diphosphomevalonate decarboxylase OS=Mus musculus OX=10090 GN=Mvd PE=1 SV=2 - [MVD1_MOUSE]	Mvd	0	0	0	0	7.715251905	0	0	2.571750635	0	4.454402764	0.504985075	0.764125594	only in a1H	only in a1H
Q99JP4	Anaphase-promoting complex subunit CDC26 OS=Mus musculus OX=10090 GN=Cdc26 PE=1 SV=1 - [CDC26_MOUSE]	Cdc26	0	0	0	10.50582333	0	0	0	3.501941108	0	6.065539925	0.504985075	0.764125594	only in a1H	only in a1H
Q99JT9	1,2-dihydroxy-3-keto-5-methylthiopentene dioxygenase OS=Mus musculus OX=10090 GN=Adi1 PE=1 SV=1 - [MTND_MOUSE]	Adi1	0	0	0	0	49.42239969	0	0	16.47413323	0	28.53403576	0.504985075	0.764125594	only in a1H	only in a1H
Q99JX7	Nuclear RNA export factor 1 OS=Mus musculus OX=10090 GN=Nxf1 PE=1 SV=3 - [NXF1_MOUSE]	Nxf1	0	0	0	0	13.5297963	0	0	4.509932099	0	7.811431534	0.504985075	0.764125594	only in a1H	only in a1H
Q99K01	Pyridoxal-dependent decarboxylase domain-containing protein 1 OS=Mus musculus OX=10090 GN=Pdxdc1 PE=1 SV=2 - [PDXD1_MOUSE]	Pdxdc1	0	0	0	10.14536639	0	0	0	3.381788796	0	5.857430015	0.504985075	0.764125594	only in a1H	only in a1H
Q99K10	Neuroligin-1 OS=Mus musculus OX=10090 GN=Nlgn1 PE=1 SV=2 - [NLGN1_MOUSE]	Nlgn1	0	0	0	0	47.60378631	0	0	15.86792877	0	27.48405884	0.504985075	0.764125594	only in a1H	only in a1H
Q99KK9	Histidine--tRNA ligase, mitochondrial OS=Mus musculus OX=10090 GN=Hars2 PE=1 SV=1 - [SYHM_MOUSE]	Hars2	0	0	0	0	0	157.0061564	0	52.33538547	0	90.64754667	0.504985075	0.764125594	only in a1H	only in a1H
Q99L13	3-hydroxyisobutyrate dehydrogenase, mitochondrial OS=Mus musculus OX=10090 GN=Hibadh PE=1 SV=1 - [3HIDH_MOUSE]	Hibadh	0	0	0	0	54.18163136	0	0	18.06054379	0	31.28177945	0.504985075	0.764125594	only in a1H	only in a1H
Q99LC2	Cleavage stimulation factor subunit 1 OS=Mus musculus OX=10090 GN=Cstf1 PE=1 SV=1 - [CSTF1_MOUSE]	Cstf1	0	0	0	13.98816781	0	0	0	4.662722602	0	8.076072449	0.504985075	0.764125594	only in a1H	only in a1H
Q99LW6	YY1-associated factor 2 OS=Mus musculus OX=10090 GN=Yaf2 PE=1 SV=1 - [YAF2_MOUSE]	Yaf2	0	0	0	0	4.873171218	0	0	1.624390406	0	2.813526715	0.504985075	0.764125594	only in a1H	only in a1H
Q99MS8	Tubulin polyglutamylase complex subunit 1 OS=Mus musculus OX=10090 GN=Tpgs1 PE=1 SV=1 - [TPGS1_MOUSE]	Tpgs1	0	0	0	0	44.83310703	0	0	14.94436901	0	25.88440641	0.504985075	0.764125594	only in a1H	only in a1H
Q99NF2	NMDA receptor synaptonuclear signaling and neuronal migration factor OS=Mus musculus OX=10090 GN=Nsmf PE=1 SV=1 - [NSMF_MOUSE]	Nsmf	0	0	0	60.32169295	0	0	0	20.10723098	0	34.82674566	0.504985075	0.764125594	only in a1H	only in a1H
Q99PG2	Opioid growth factor receptor OS=Mus musculus OX=10090 GN=Ogfr PE=1 SV=1 - [OGFR_MOUSE]	Ogfr	0	0	0	53.82772834	0	0	0	17.94257611	0	31.07745345	0.504985075	0.764125594	only in a1H	only in a1H
Q99PL6	UBX domain-containing protein 6 OS=Mus musculus OX=10090 GN=Ubxn6 PE=1 SV=1 - [UBXN6_MOUSE]	Ubxn6	0	0	0	0	133.0211201	0	0	44.34037335	0	76.79977948	0.504985075	0.764125594	only in a1H	only in a1H
Q9CQ22	Ragulator complex protein LAMTOR1 OS=Mus musculus OX=10090 GN=Lamtor1 PE=1 SV=1 - [LTOR1_MOUSE]	Lamtor1	0	0	0	0	10.08907053	0	0	3.363023511	0	5.824927588	0.504985075	0.764125594	only in a1H	only in a1H
Q9CQ26	STAM-binding protein OS=Mus musculus OX=10090 GN=Stambp PE=1 SV=1 - [STABP_MOUSE]	Stambp	0	0	0	0	38.7518291	0	0	12.91727637	0	22.37337896	0.504985075	0.764125594	only in a1H	only in a1H
Q9CQ46	Dynein regulatory complex protein 8 OS=Mus musculus OX=10090 GN=Efcab2 PE=1 SV=1 - [DRC8_MOUSE]	Efcab2	0	0	0	3.644550854	0	0	0	1.214850285	0	2.104182417	0.504985075	0.764125594	only in a1H	only in a1H
Q9CQ56	Vesicle transport protein USE1 OS=Mus musculus OX=10090 GN=Use1 PE=1 SV=1 - [USE1_MOUSE]	Use1	0	0	0	0	11.86368799	0	0	3.954562663	0	6.849503453	0.504985075	0.764125594	only in a1H	only in a1H
Q9CQL0	Protein N-lysine methyltransferase METTL21A OS=Mus musculus OX=10090 GN=Mettl21A PE=2 SV=1 - [MT21A_MOUSE]	Mettl21A	0	0	0	0	9.32741746	0	0	3.109139153	0	5.385186981	0.504985075	0.764125594	only in a1H	only in a1H
Q9CQP2	Trafficking protein particle complex subunit 2 OS=Mus musculus OX=10090 GN=Trappc2 PE=1 SV=1 - [TPPC2_MOUSE]	Trappc2	0	0	0	7.240966	0	0	0	2.413655333	0	4.18057367	0.504985075	0.764125594	only in a1H	only in a1H
Q9CQW9	Interferon-induced transmembrane protein 3 OS=Mus musculus OX=10090 GN=Ifitm3 PE=1 SV=1 - [IFM3_MOUSE]	Ifitm3	0	0	0	41.77445078	0	0	0	13.92481693	0	24.11849041	0.504985075	0.764125594	only in a1H	only in a1H
Q9CQZ1	Heat shock factor-binding protein 1 OS=Mus musculus OX=10090 GN=Hsbp1 PE=1 SV=1 - [HSBP1_MOUSE]	Hsbp1	0	0	0	4.983890946	0	0	0	1.661296982	0	2.877450779	0.504985075	0.764125594	only in a1H	only in a1H
Q9CR26	Vacuolar protein sorting-associated protein VTA1 homolog OS=Mus musculus OX=10090 GN=Vta1 PE=1 SV=1 - [VTA1_MOUSE]	Vta1	0	0	0	0	44.84874791	0	0	14.94958264	0	25.89343668	0.504985075	0.764125594	only in a1H	only in a1H
Q9CSB4	Partitioning defective 3 homolog B OS=Mus musculus OX=10090 GN=Pard3b PE=1 SV=2 - [PAR3L_MOUSE]	Pard3b	0	0	0	67.90443543	0	0	0	22.63481181	0	39.20464408	0.504985075	0.764125594	only in a1H	only in a1H
Q9CSH3	Exosome complex exonuclease RRP44 OS=Mus musculus OX=10090 GN=Dis3 PE=1 SV=4 - [RRP44_MOUSE]	Dis3	0	0	0	14.03664985	0	0	0	4.678883283	0	8.104063569	0.504985075	0.764125594	only in a1H	only in a1H
Q9CT10	Ran-binding protein 3 OS=Mus musculus OX=10090 GN=Ranbp3 PE=1 SV=2 - [RANB3_MOUSE]	Ranbp3	0	0	0	0	22.70349366	0	0	7.567831219	0	13.10786817	0.504985075	0.764125594	only in a1H	only in a1H
Q9D0J4	ADP-ribosylation factor-like protein 2 OS=Mus musculus OX=10090 GN=Arl2 PE=1 SV=1 - [ARL2_MOUSE]	Arl2	0	0	0	0	7.819970758	0	0	2.606656919	0	4.514862222	0.504985075	0.764125594	only in a1H	only in a1H
Q9D0J8	Parathymosin OS=Mus musculus OX=10090 GN=Ptms PE=1 SV=3 - [PTMS_MOUSE]	Ptms	0	0	0	0	144.4191437	0	0	48.13971457	0	83.3804315	0.504985075	0.764125594	only in a1H	only in a1H
Q9D125	28S ribosomal protein S25, mitochondrial OS=Mus musculus OX=10090 GN=Mrps25 PE=1 SV=1 - [RT25_MOUSE]	Mrps25	0	0	0	0	48.29669143	0	0	16.09889714	0	27.8841078	0.504985075	0.764125594	only in a1H	only in a1H
Q9D1K2	V-type proton ATPase subunit F OS=Mus musculus OX=10090 GN=Atp6v1f PE=1 SV=2 - [VATF_MOUSE]	Atp6v1f	0	0	0	0	5.799879859	0	0	1.933293286	0	3.348562198	0.504985075	0.764125594	only in a1H	only in a1H
Q9D2V7	Coronin-7 OS=Mus musculus OX=10090 GN=Coro7 PE=1 SV=2 - [CORO7_MOUSE]	Coro7	0	0	0	7.501320886	0	0	0	2.500440295	0	4.330889633	0.504985075	0.764125594	only in a1H	only in a1H
Q9D3D9	ATP synthase subunit delta, mitochondrial OS=Mus musculus OX=10090 GN=Atp5f1d PE=1 SV=1 - [ATPD_MOUSE]	Atp5f1d	0	0	0	0	60.66884109	0	0	20.22294703	0	35.02717173	0.504985075	0.764125594	only in a1H	only in a1H
Q9D4H8	Cullin-2 OS=Mus musculus OX=10090 GN=Cul2 PE=1 SV=2 - [CUL2_MOUSE]	Cul2	0	0	0	14.44479483	0	0	0	4.814931611	0	8.339706186	0.504985075	0.764125594	only in a1H	only in a1H
Q9D4V0	Ethanolamine kinase 1 OS=Mus musculus OX=10090 GN=Etnk1 PE=1 SV=2 - [EKI1_MOUSE]	Etnk1	0	0	0	24.89010965	0	0	0	8.296703217	0	14.37031151	0.504985075	0.764125594	only in a1H	only in a1H
Q9D4V7	Rab-like protein 3 OS=Mus musculus OX=10090 GN=Rabl3 PE=1 SV=1 - [RABL3_MOUSE]	Rabl3	0	0	0	0	0	219.6916309	0	73.23054363	0	126.8390222	0.504985075	0.764125594	only in a1H	only in a1H
Q9D5V5	Cullin-5 OS=Mus musculus OX=10090 GN=Cul5 PE=1 SV=3 - [CUL5_MOUSE]	Cul5	0	0	0	0	18.17184835	0	0	6.057282785	0	10.49152154	0.504985075	0.764125594	only in a1H	only in a1H
Q9D6K7	Tetratricopeptide repeat protein 33 OS=Mus musculus OX=10090 GN=Ttc33 PE=1 SV=1 - [TTC33_MOUSE]	Ttc33	0	0	0	6.311344455	0	0	0	2.103781485	0	3.64385642	0.504985075	0.764125594	only in a1H	only in a1H
Q9D6V8	Polyadenylate-binding protein-interacting protein 2 OS=Mus musculus OX=10090 GN=Paip2 PE=1 SV=1 - [PAIP2_MOUSE]	Paip2	0	0	0	12.2105181	0	0	0	4.070172699	0	7.04974591	0.504985075	0.764125594	only in a1H	only in a1H

Q9D6Y7	Mitochondrial peptide methionine sulfoxide reductase OS=Mus musculus OX=10090 GN=Msra PE=1 SV=1 - [MSRA_MOUSE]	Msra	0	0	0	46.90096321	0	0	0	15.6336544	0	27.07828374	0.504985075	0.764125594	only in a1H	only in a1H
Q9D753	Exosome complex component RRP43 OS=Mus musculus OX=10090 GN=Exosc8 PE=1 SV=1 - [EXOS8_MOUSE]	Exosc8	0	0	0	0	21.78426814	0	0	7.261422712	0	12.57715307	0.504985075	0.764125594	only in a1H	only in a1H
Q9D8T3	GTPase RhebL1 OS=Mus musculus OX=10090 GN=Rhebl1 PE=2 SV=1 - [REBL1_MOUSE]	Rhebl1	0	0	0	0	23.02674008	0	0	7.675580028	0	13.29449459	0.504985075	0.764125594	only in a1H	only in a1H
Q9DAW6	U4/U6 small nuclear ribonucleoprotein Prp4 OS=Mus musculus OX=10090 GN=Prpf4 PE=1 SV=1 - [PRP4_MOUSE]	Prpf4	0	0	0	0	14.78232689	0	0	4.927442297	0	8.534580411	0.504985075	0.764125594	only in a1H	only in a1H
Q9DB20	ATP synthase subunit O, mitochondrial OS=Mus musculus OX=10090 GN=Atp5po PE=1 SV=1 - [ATPO_MOUSE]	Atp5po	0	0	0	0	4.506938188	0	0	1.502312729	0	2.602081976	0.504985075	0.764125594	only in a1H	only in a1H
Q9DBT5	AMP deaminase 2 OS=Mus musculus OX=10090 GN=Ampd2 PE=1 SV=1 - [AMPD2_MOUSE]	Ampd2	0	0	0	2.285963683	0	0	0	0.761987894	0	1.319801748	0.504985075	0.764125594	only in a1H	only in a1H
Q9DCE5	p21-activated protein kinase-interacting protein 1 OS=Mus musculus OX=10090 GN=Pak1ip1 PE=1 SV=2 - [PK1IP_MOUSE]	Pak1ip1	0	0	0	0	20.38882894	0	0	6.796276314	0	11.77149588	0.504985075	0.764125594	only in a1H	only in a1H
Q9DCK3	Tetraspanin-4 OS=Mus musculus OX=10090 GN=Tspan4 PE=1 SV=1 - [TSN4_MOUSE]	Tspan4	0	0	0	0	54.05017274	0	0	18.01672425	0	31.20588178	0.504985075	0.764125594	only in a1H	only in a1H
Q9EPJ9	ADP-ribosylation factor GTPase-activating protein 1 OS=Mus musculus OX=10090 GN=Arfgap1 PE=1 SV=2 - [ARFG1_MOUSE]	Arfgap1	0	0	0	0	29.94754685	0	0	9.982515616	0	17.29022423	0.504985075	0.764125594	only in a1H	only in a1H
Q9EQF6	Dihydropyrimidinase-related protein 5 OS=Mus musculus OX=10090 GN=Dpysl5 PE=1 SV=1 - [DPYL5_MOUSE]	Dpysl5	0	0	0	0	41.26620254	0	0	13.75540085	0	23.82505315	0.504985075	0.764125594	only in a1H	only in a1H
Q9ERV7	p53-induced death domain-containing protein 1 OS=Mus musculus OX=10090 GN=Pidd1 PE=1 SV=1 - [PIDD1_MOUSE]	Pidd1	0	0	0	0	82.70093866	0	0	27.56697955	0	47.7474092	0.504985075	0.764125594	only in a1H	only in a1H
Q9ES97	Reticulon-3 OS=Mus musculus OX=10090 GN=Rtn3 PE=1 SV=2 - [RTN3_MOUSE]	Rtn3	0	0	0	175.3290489	0	0	0	58.44301632	0	101.2262736	0.504985075	0.764125594	only in a1H	only in a1H
Q9ESD7	Dysferlin OS=Mus musculus OX=10090 GN=Dysf PE=1 SV=3 - [DYSF_MOUSE]	Dysf	0	0	0	0	4.147964103	0	0	1.382654701	0	2.394828191	0.504985075	0.764125594	only in a1H	only in a1H
Q9ESN6	Tripartite motif-containing protein 2 OS=Mus musculus OX=10090 GN=Trim2 PE=1 SV=1 - [TRIM2_MOUSE]	Trim2	0	0	0	0	7.97784253	0	0	2.659280843	0	4.606009532	0.504985075	0.764125594	only in a1H	only in a1H
Q9EST4	Proteasome assembly chaperone 2 OS=Mus musculus OX=10090 GN=Psmg2 PE=1 SV=1 - [PSMG2_MOUSE]	Psmg2	0	0	0	0	8.650368155	0	0	2.883456052	0	4.994292383	0.504985075	0.764125594	only in a1H	only in a1H
Q9JKW0	ADP-ribosylation factor-like protein 6-interacting protein 1 OS=Mus musculus OX=10090 GN=Arl6ip1 PE=1 SV=1 - [AR6P1_MOUSE]	Arl6ip1	0	0	0	0	68.23816515	0	0	22.74605505	0	39.39732302	0.504985075	0.764125594	only in a1H	only in a1H
Q9JKY0	CCR4-NOT transcription complex subunit 9 OS=Mus musculus OX=10090 GN=Cnot9 PE=1 SV=1 - [CNOT9_MOUSE]	Cnot9	0	0	0	0	24.23298655	0	0	8.077662183	0	13.99092131	0.504985075	0.764125594	only in a1H	only in a1H
Q9JLC8	Sacsin OS=Mus musculus OX=10090 GN=Sacs PE=1 SV=2 - [SACS_MOUSE]	Sacs	0	0	0	1164.653669	0	0	0	388.2178897	0	672.4131094	0.504985075	0.764125594	only in a1H	only in a1H
Q9JLQ2	ARF GTPase-activating protein GIT2 OS=Mus musculus OX=10090 GN=Git2 PE=1 SV=2 - [GIT2_MOUSE]	Git2	0	0	0	5.68444032	0	0	0	1.89481344	0	3.281913149	0.504985075	0.764125594	only in a1H	only in a1H
Q9QVN7	Transcription elongation factor A protein 2 OS=Mus musculus OX=10090 GN=Tcea2 PE=2 SV=2 - [TCEA2_MOUSE]	Tcea2	0	0	0	0	0	25.31561318	0	8.438537726	0	14.61597608	0.504985075	0.764125594	only in a1H	only in a1H
Q9QWR8	Alpha-N-acetylgalactosaminidase OS=Mus musculus OX=10090 GN=Naga PE=1 SV=2 - [NAGAB_MOUSE]	Naga	0	0	0	0	6.657900346	0	0	2.219300115	0	3.843940557	0.504985075	0.764125594	only in a1H	only in a1H
Q9QXA1	Cysteine and histidine-rich protein 1 OS=Mus musculus OX=10090 GN=Cyhr1 PE=1 SV=1 - [CYHR1_MOUSE]	Cyhr1	0	0	0	19.42474019	0	0	0	6.474913397	0	11.21487898	0.504985075	0.764125594	only in a1H	only in a1H
Q9QXN0	Protein Shroom3 OS=Mus musculus OX=10090 GN=Shroom3 PE=1 SV=2 - [SHRM3_MOUSE]	Shroom3	0	0	0	0	741.7152159	0	0	247.2384053	0	428.2294796	0.504985075	0.764125594	only in a1H	only in a1H
Q9QY76	Vesicle-associated membrane protein-associated protein B OS=Mus musculus OX=10090 GN=Vapb PE=1 SV=3 - [VAPB_MOUSE]	Vapb	0	0	0	0	215.9574133	0	0	71.98580444	0	124.6830707	0.504985075	0.764125594	only in a1H	only in a1H
Q9QZH3	Peptidyl-prolyl cis-trans isomerase E OS=Mus musculus OX=10090 GN=Ppie PE=1 SV=2 - [PPIE_MOUSE]	Ppie	0	0	0	27.34416386	0	0	0	9.114721286	0	15.78716036	0.504985075	0.764125594	only in a1H	only in a1H
Q9R0D8	WD repeat-containing protein 54 OS=Mus musculus OX=10090 GN=Wdr54 PE=1 SV=1 - [WDR54_MOUSE]	Wdr54	0	0	0	6.156483355	0	0	0	2.052161118	0	3.554447322	0.504985075	0.764125594	only in a1H	only in a1H
Q9WTK5	Nuclear factor NF-kappa-B p100 subunit OS=Mus musculus OX=10090 GN=Nfkb2 PE=1 SV=1 - [NFKB2_MOUSE]	Nfkb2	0	0	0	0	4.244831614	0	0	1.414943871	0	2.450754675	0.504985075	0.764125594	only in a1H	only in a1H
Q9WTX8	Mitotic spindle assembly checkpoint protein MAD1 OS=Mus musculus OX=10090 GN=Mad1l1 PE=1 SV=1 - [MD1L1_MOUSE]	Mad1l1	0	0	0	0	552.4269363	0	0	184.1423121	0	318.9438404	0.504985075	0.764125594	only in a1H	only in a1H
Q9WV60	Glycogen synthase kinase-3 beta OS=Mus musculus OX=10090 GN=Gsk3b PE=1 SV=2 - [GSK3B_MOUSE]	Gsk3b	0	0	0	0	15.42277191	0	0	5.140923969	0	8.904341512	0.504985075	0.764125594	only in a1H	only in a1H
Q9Z0E0	Neurochondrin OS=Mus musculus OX=10090 GN=Ncdn PE=1 SV=1 - [NCDN_MOUSE]	Ncdn	0	0	0	0	8.553381352	0	0	2.851127117	0	4.938297026	0.504985075	0.764125594	only in a1H	only in a1H
Q9Z0J0	NPC intracellular cholesterol transporter 2 OS=Mus musculus OX=10090 GN=Npc2 PE=1 SV=1 - [NPC2_MOUSE]	Npc2	0	0	0	28.60642329	0	0	0	9.535474428	0	16.51592618	0.504985075	0.764125594	only in a1H	only in a1H
Q9Z0U1	Tight junction protein ZO-2 OS=Mus musculus OX=10090 GN=Tjp2 PE=1 SV=2 - [ZO2_MOUSE]	Tjp2	0	0	0	53.07282987	0	0	0	17.69094329	0	30.64161261	0.504985075	0.764125594	only in a1H	only in a1H
Q9Z175	Lysyl oxidase homolog 3 OS=Mus musculus OX=10090 GN=Loxl3 PE=1 SV=2 - [LOXL3_MOUSE]	Loxl3	0	0	0	0	2.496391408	0	0	0.832130469	0	1.441292252	0.504985075	0.764125594	only in a1H	only in a1H
Q9Z2Y2	Beta-1,4-galactosyltransferase 2 OS=Mus musculus OX=10090 GN=B4galt2 PE=2 SV=1 - [B4GT2_MOUSE]	B4galt2	0	0	0	0	194.639389	0	0	64.87979632	0	112.3751036	0.504985075	0.764125594	only in a1H	only in a1H
A2A9C3	KICSTOR complex protein SZT2 OS=Mus musculus OX=10090 GN=Szt2 PE=1 SV=1 - [SZT2_MOUSE]	Szt2	74.7014895	0	0	0	0	0	24.9004965	0	43.12892507	0	0.504985075	0.764125594	only in mock	only in mock
A2A9R3	Melanoma-associated antigen B4 OS=Mus musculus OX=10090 GN=Mageb4 PE=1 SV=1 - [MAGB4_MOUSE]	Mageb4	194.5978598	0	0	0	0	0	64.86595327	0	112.3511267	0	0.504985075	0.764125594	only in mock	only in mock
A2AKX3	Probable helicase senataxin OS=Mus musculus OX=10090 GN=Setx PE=1 SV=1 - [SETX_MOUSE]	Setx	36.54763117	0	0	0	0	0	12.18254372	0	21.1007847	0	0.504985075	0.764125594	only in mock	only in mock
A6H584	Collagen alpha-5(VI) chain OS=Mus musculus OX=10090 GN=Col6a5 PE=1 SV=4 - [CO6A5_MOUSE]	Col6a5	0	146.4486291	0	0	0	0	48.8162097	0	84.55215544	0	0.504985075	0.764125594	only in mock	only in mock
A6H6E9	Tetratricopeptide repeat protein 23-like OS=Mus musculus OX=10090 GN=Ttc23l PE=2 SV=1 - [TT23L_MOUSE]	Ttc23l	0	24.74239824	0	0	0	0	8.247466081	0	14.28503029	0	0.504985075	0.764125594	only in mock	only in mock
A8C756	Thyroid adenoma-associated protein homolog OS=Mus musculus OX=10090 GN=Thada PE=1 SV=1 - [THADA_MOUSE]	Thada	0	0	98.66285605	0	0	0	32.88761868	0	56.9630265	0	0.504985075	0.764125594	only in mock	only in mock
B2RRE7	OTU domain-containing protein 4 OS=Mus musculus OX=10090 GN=Otud4 PE=1 SV=1 - [OTUD4_MOUSE]	Otud4	0	0	441.2341814	0	0	0	147.0780605	0	254.7466734	0	0.504985075	0.764125594	only in mock	only in mock
B2RY04	Dedicator of cytokinesis protein 5 OS=Mus musculus OX=10090 GN=Dock5 PE=1 SV=2 - [DOCK5_MOUSE]	Dock5	0	619.9997505	0	0	0	0	206.6665835	0	357.9570229	0	0.504985075	0.764125594	only in mock	only in mock
O08585	Clathrin light chain A OS=Mus musculus OX=10090 GN=Clta PE=1 SV=2 - [CLCA_MOUSE]	Clta	0	129.6170397	0	0	0	0	43.20567991	0	74.83443278	0	0.504985075	0.764125594	only in mock	only in mock
O08663	Methionine aminopeptidase 2 OS=Mus musculus OX=10090 GN=Metap2 PE=1 SV=1 - [MAP2_MOUSE]	Metap2	20.40708035	0	0	0	0	0	6.802360115	0	11.78203333	0	0.504985075	0.764125594	only in mock	only in mock
O08689	Growth/differentiation factor 8 OS=Mus musculus OX=10090 GN=Mstn PE=1 SV=1 - [GDF8_MOUSE]	Mstn	0	26.62145414	0	0	0	0	8.873818047	0	15.36990371	0	0.504985075	0.764125594	only in mock	only in mock
O08842	GDNF family receptor alpha-2 OS=Mus musculus OX=10090 GN=Gfra2 PE=1 SV=2 - [GFRA2_MOUSE]	Gfra2	28.99315363	0	0	0	0	0	9.664384545	0	16.73920506	0	0.504985075	0.764125594	only in mock	only in mock
O08915	AH receptor-interacting protein OS=Mus musculus OX=10090 GN=Aip PE=1 SV=1 - [AIP_MOUSE]	Aip	17.55460475	0	0	0	0	0	5.851534916	0	10.13515578	0	0.504985075	0.764125594	only in mock	only in mock
O09167	60S ribosomal protein L21 OS=Mus musculus OX=10090 GN=Rpl21 PE=1 SV=3 - [RL21_MOUSE]	Rpl21	44.93131134	0	0	0	0	0	14.97710378	0	25.9411047	0	0.504985075	0.764125594	only in mock	only in mock
O35136	Neural cell adhesion molecule 2 OS=Mus musculus OX=10090 GN=Ncam2 PE=1 SV=1 - [NCAM2_MOUSE]	Ncam2	74.78286532	0	0	0	0	0	24.92762177	0	43.17590742	0	0.504985075	0.764125594	only in mock	only in mock
O35639	Annexin A3 OS=Mus musculus OX=10090 GN=Anxa3 PE=1 SV=4 - [ANXA3_MOUSE]	Anxa3	67.16185046	0	0	0	0	0	22.38728349	0	38.77591244	0	0.504985075	0.764125594	only in mock	only in mock
O35658	Complement component 1 Q subcomponent-binding protein, mitochondrial OS=Mus musculus OX=10090 GN=C1qbp PE=1 SV=1 - [C1QBP_MOUSE]	C1qbp	48.64735245	0	0	0	0	0	16.21578415	0	28.08656203	0	0.504985075	0.764125594	only in mock	only in mock
O54967	Activated CDC42 kinase 1 OS=Mus musculus OX=10090 GN=Tnk2 PE=1 SV=2 - [ACK1_MOUSE]	Tnk2	0	0	901.5981742	0	0	0	300.5327247	0	520.5379486	0	0.504985075	0.764125594	only in mock	only in mock
O70451	Monocarboxylate transporter 2 OS=Mus musculus OX=10090 GN=Slc16a7 PE=1 SV=1 - [MOT2_MOUSE]	Slc16a7	0	85403.62887	0	0	0	0	28467.87629	0	49307.80812	0	0.504985075	0.764125594	only in mock	only in mock
O88322	Nidogen-2 OS=Mus musculus OX=10090 GN=Nid2 PE=1 SV=2 - [NID2_MOUSE]	Nid2	0	76.3810826	0	0	0	0	25.46036087	0	44.0986386	0	0.504985075	0.764125594	only in mock	only in mock
P02535	Keratin, type I cytoskeletal 10 OS=Mus musculus OX=10090 GN=Krt10 PE=1 SV=3 - [K1C10_MOUSE]	Krt10	0	411.1163057	0	0	0	0	137.0387686	0	237.3581098	0	0.504985075	0.764125594	only in mock	only in mock
P02802	Metallothionein-1 OS=Mus musculus OX=10090 GN=Mt1 PE=1 SV=1 - [MT1_MOUSE]	Mt1	5.607042164	0	0	0	0	0	1.869014055	0	3.237227303	0	0.504985075	0.764125594	only in mock	only in mock
P08775	DNA-directed RNA polymerase II subunit RPB1 OS=Mus musculus OX=10090 GN=Polr2a PE=1 SV=3 - [RPB1_MOUSE]	Polr2a	8.849919535	0	0	0	0	0	2.949973178	0	5.109503426	0	0.504985075	0.764125594	only in mock	only in mock
P0C027	Diphosphoinositol polyphosphate phosphohydrolase 3-alpha OS=Mus musculus OX=10090 GN=Nudt10 PE=1 SV=1 - [NUD10_MOUSE]	Nudt10	0	59.36537278	0	0	0	0	19.78845759	0	34.27461396	0	0.504985075	0.764125594	only in mock	only in mock
P12265	Beta-glucuronidase OS=Mus musculus OX=10090 GN=Gusb PE=1 SV=2 - [BGLR_MOUSE]	Gusb	5.042150695	0	0	0	0	0	1.680716898	0	2.911087061	0	0.504985075	0.764125594	only in mock	only in mock
P16330	2',3'-cyclic-nucleotide 3'-phosphodiesterase OS=Mus musculus OX=10090 GN=Cnp PE=1 SV=3 - [CN37_MOUSE]	Cnp	26.27861082	0	0	0	0	0	8.759536941	0	15.17196303	0	0.504985075	0.764125594	only in mock	only in mock
P19536	Cytochrome c oxidase subunit 5B, mitochondrial OS=Mus musculus OX=10090 GN=Cox5b PE=1 SV=1 - [COX5B_MOUSE]	Cox5b	0	0	24.30155496	0	0	0	8.100518321	0	14.0305093	0	0.504985075	0.764125594	only in mock	only in mock
P21279	Guanine nucleotide-binding protein G(q) subunit alpha OS=Mus musculus OX=10090 GN=Gnaq PE=1 SV=4 - [GNAQ_MOUSE]	Gnaq	29.84133953	0	0	0	0	0	9.947113176	0	17.22890541	0	0.504985075	0.764125594	only in mock	only in mock
P28740	Kinesin-like protein KIF2A OS=Mus musculus OX=10090 GN=Kif2a PE=1 SV=2 - [KIF2A_MOUSE]	Kif2a	9.697668541	0	0	0	0	0	3.23255618	0	5.598951543	0	0.504985075	0.764125594	only in mock	only in mock
P35396	Peroxisome proliferator-activated receptor delta OS=Mus musculus OX=10090 GN=Ppard PE=1 SV=1 - [PPARD_MOUSE]	Ppard	0	0	1307.400308	0	0	0	435.8001028	0	754.82792	0	0.504985075	0.764125594	only in mock	only in mock
P35922	Synaptic functional regulator FMR1 OS=Mus musculus OX=10090 GN=Fmr1 PE=1 SV=1 - [FMR1_MOUSE]	Fmr1	20.38163025	0	0	0	0	0	6.79387675	0	11.76733971	0	0.504985075	0.764125594	only in mock	only in mock
P36552	Oxygen-dependent coproporphyrinogen-III oxidase, mitochondrial OS=Mus musculus OX=10090 GN=Cpox PE=1 SV=2 - [HEM6_MOUSE]	Cpox	12.59418706	0	0	0	0	0	4.198062354	0	7.271257291	0	0.504985075	0.764125594	only in mock	only in mock
P41241	Tyrosine-protein kinase CSK OS=Mus musculus OX=10090 GN=Csk PE=1 SV=2 - [CSK_MOUSE]	Csk	14.49277215	0	0	0	0	0	4.83092405	0	8.367405902	0	0.504985075	0.764125594	only in mock	only in mock
P47739	Aldehyde dehydrogenase, dimeric NADP-preferring OS=Mus musculus OX=10090 GN=Aldh3a1 PE=1 SV=2 - [AL3A1_MOUSE]	Aldh3a1	0	0	84.76267873	0	0	0	28.25422624	0	48.93775538	0	0.504985075	0.764125594	only in mock	only in mock
P47857	ATP-dependent 6-phosphofructokinase, muscle type OS=Mus musculus OX=10090 GN=Pfkm PE=1 SV=3 - [PFKAM_MOUSE]	Pfkm	21.52990728	0	0	0	0	0	7.176635761	0	12.43029777	0	0.504985075	0.764125594	only in mock	only in mock
P48379	DNA-binding protein RFX2 OS=Mus musculus OX=10090 GN=Rfx2 PE=1 SV=2 - [RFX2_MOUSE]	Rfx2	0	522.1652167	0	0	0	0	174.0550722	0	301.4722284	0	0.504985075	0.764125594	only in mock	only in mock
P48759	Pentraxin-related protein PTX3 OS=Mus musculus OX=10090 GN=Ptx3 PE=1 SV=2 - [PTX3_MOUSE]	Ptx3	0	14.73203256	0	0	0	0	4.910677519	0	8.505542962	0	0.504985075	0.764125594	only in mock	only in mock
P51807	Dynein light chain Tctex-type 1 OS=Mus musculus OX=10090 GN=Dynlt1 PE=1 SV=1 - [DYLT1_MOUSE]	Dynlt1	31.13395691	0	0	0	0	0	10.37798564	0	17.9751984	0	0.504985075	0.764125594	only in mock	only in mock
P51881	ADP/ATP translocase 2 OS=Mus musculus OX=10090 GN=Slc25a5 PE=1 SV=3 - [ADT2_MOUSE]	Slc25a5	41.72691188	0	0	0	0	0	13.90897063	0	24.09104381	0	0.504985075	0.764125594	only in mock	only in mock
P54320	Elastin OS=Mus musculus OX=10090 GN=Eln PE=1 SV=2 - [ELN_MOUSE]	Eln	66.46140777	0	0	0	0	0	22.15380259	0	38.37151167	0	0.504985075	0.764125594	only in mock	only in mock
P59326	YTH domain-containing family protein 1 OS=Mus musculus OX=10090 GN=Ythdf1 PE=1 SV=1 - [YTHD1_MOUSE]	Ythdf1	0	0	29.62066695	0	0	0	9.873555651	0	17.10150004	0	0.504985075	0.764125594	only in mock	only in mock
P59708	Splicing factor 3B subunit 6 OS=Mus musculus OX=10090 GN=Sf3b6 PE=1 SV=1 - [SF3B6_MOUSE]	Sf3b6	0	17.90214641	0	0	0	0	5.967382136	0	10.33580905	0	0.504985075	0.764125594	only in mock	only in mock
P62715	Serine/threonine-protein phosphatase 2A catalytic subunit beta isoform OS=Mus musculus OX=10090 GN=Ppp2cb PE=1 SV=1 - [PP2AB_MOUSE]	Ppp2cb	0	0	88.14867495	0	0	0	29.38289165	0	50.89266121	0	0.504985075	0.764125594	only in mock	only in mock
P63094	Guanine nucleotide-binding protein G(s) subunit alpha isoforms short OS=Mus musculus OX=10090 GN=Gnas PE=1 SV=1 - [GNAS2_MOUSE]	Gnas	178.0098709	0	0	0	0	0	59.33662362	0	102.7740469	0	0.504985075	0.764125594	only in mock	only in mock
P68373	Tubulin alpha-1C chain OS=Mus musculus OX=10090 GN=Tuba1c PE=1 SV=1 - [TBA1C_MOUSE]	Tuba1c	0	0	648.7243439	0	0	0	216.241448	0	374.5411746	0	0.504985075	0.764125594	only in mock	only in mock
P70379	Fibroblast growth factor 14 OS=Mus musculus OX=10090 GN=Fgf14 PE=2 SV=1 - [FGF14_MOUSE]	Fgf14	0	0	91.62481634	0	0	0	30.54160545	0	52.89961238	0	0.504985075	0.764125594	only in mock	only in mock
P97300	Neuroplastin OS=Mus musculus OX=10090 GN=Nptn PE=1 SV=3 - [NPTN_MOUSE]	Nptn	0	0	44.26179402	0	0	0	14.75393134	0	25.55455869	0	0.504985075	0.764125594	only in mock	only in mock
P97333	Neuropilin-1 OS=Mus musculus OX=10090 GN=Nrp1 PE=1 SV=2 - [NRP1_MOUSE]	Nrp1	0	23.8102432	0	0	0	0	7.936747734	0	13.74685032	0	0.504985075	0.764125594	only in mock	only in mock
P97434	Myosin phosphatase Rho-interacting protein OS=Mus musculus OX=10090 GN=Mprip PE=1 SV=2 - [MPRIP_MOUSE]	Mprip	9.617996674	0	0	0	0	0	3.205998891	0	5.552952969	0	0.504985075	0.764125594	only in mock	only in mock
P97471	Mothers against decapentaplegic homolog 4 OS=Mus musculus OX=10090 GN=Smad4 PE=1 SV=2 - [SMAD4_MOUSE]	Smad4	18.86110211	0	0	0	0	0	6.287034036	0	10.88946238	0	0.504985075	0.764125594	only in mock	only in mock
P97865	Peroxisomal targeting signal 2 receptor OS=Mus musculus OX=10090 GN=Pex7 PE=1 SV=1 - [PEX7_MOUSE]	Pex7	24.66281456	0	0	0	0	0	8.220938188	0	14.23908263	0	0.504985075	0.764125594	only in mock	only in mock
P97927	Laminin subunit alpha-4 OS=Mus musculus OX=10090 GN=Lama4 PE=1 SV=2 - [LAMA4_MOUSE]	Lama4	0	8.092600931	0	0	0	0	2.697533644	0	4.672265326	0	0.504985075	0.764125594	only in mock	only in mock
P97930	Thymidylate kinase OS=Mus musculus OX=10090 GN=Dtymk PE=1 SV=2 - [KTHY_MOUSE]	Dtymk	15.78288398	0	0	0	0	0	5.260961328	0	9.112252316	0	0.504985075	0.764125594	only in mock	only in mock
P98078	Disabled homolog 2 OS=Mus musculus OX=10090 GN=Dab2 PE=1 SV=2 - [DAB2_MOUSE]	Dab2	9.777820611	0	0	0	0	0	3.259273537	0	5.645227362	0	0.504985075	0.764125594	only in mock	only in mock
Q02357	Ankyrin-1 OS=Mus musculus OX=10090 GN=Ank1 PE=1 SV=2 - [ANK1_MOUSE]	Ank1	0	35.14058328	0	0	0	0	11.71352776	0	20.28842522	0	0.504985075	0.764125594	only in mock	only in mock
Q03141	MAP/microtubule affinity-regulating kinase 3 OS=Mus musculus OX=10090 GN=Mark3 PE=1 SV=2 - [MARK3_MOUSE]	Mark3	3.22925524	0	0	0	0	0	1.076418413	0	1.864411382	0	0.504985075	0.764125594	only in mock	only in mock
Q03350	Thrombospondin-2 OS=Mus musculus OX=10090 GN=Thbs2 PE=1 SV=2 - [TSP2_MOUSE]	Thbs2	43.60901075	0	0	0	0	0	14.53633692	0	25.17767409	0	0.504985075	0.764125594	only in mock	only in mock
Q04692	SWI/SNF-related matrix-associated actin-dependent regulator of chromatin subfamily A containing DEAD/H box 1 OS=Mus musculus OX=10090 GN=Smarc	Smarcad1	3.993228379	0	0	0	0	0	1.331076126	0	2.30549148	0	0.504985075	0.764125594	only in mock	only in mock
Q07076	Annexin A7 OS=Mus musculus OX=10090 GN=Anxa7 PE=1 SV=2 - [ANXA7_MOUSE]	Anxa7	6.018524112	0	0	0	0	0	2.006174704	0	3.474796516	0	0.504985075	0.764125594	only in mock	only in mock
Q149F1	RNA pseudouridylate synthase domain-containing protein 2 OS=Mus musculus OX=10090 GN=Rpusd2 PE=1 SV=2 - [RUSD2_MOUSE]	Rpusd2	7.855121562	0	0	0	0	0	2.618373854	0	4.535156548	0	0.504985075	0.764125594	only in mock	only in mock
Q3TCH7	Cullin-4A OS=Mus musculus OX=10090 GN=Cul4a PE=1 SV=1 - [CUL4A_MOUSE]	Cul4a	32.85937974	0	0	0	0	0	10.95312658	0	18.97137174	0	0.504985075	0.764125594	only in mock	only in mock

Q3TUH1	Phosphatidate cytidylyltransferase, mitochondrial OS=Mus musculus OX=10090 GN=Tamm41 PE=1 SV=2 - [TAM41_MOUSE]	Tamm41	0	0	342.5991111	0	0	0	114.1997037	0	197.799689	0	0.504985075	0.764125594	only in mock	only in mock
Q3UHA3	Spatacsin OS=Mus musculus OX=10090 GN=Spg11 PE=1 SV=3 - [SPTCS_MOUSE]	Spg11	27.27326855	0	0	0	0	0	9.091089517	0	15.74622894	0	0.504985075	0.764125594	only in mock	only in mock
Q3UJD6	Ubiquitin carboxyl-terminal hydrolase 19 OS=Mus musculus OX=10090 GN=Usp19 PE=1 SV=1 - [UBP19_MOUSE]	Usp19	16.29331468	0	0	0	0	0	5.431104892	0	9.406949615	0	0.504985075	0.764125594	only in mock	only in mock
Q3UM45	Protein phosphatase 1 regulatory subunit 7 OS=Mus musculus OX=10090 GN=Ppp1r7 PE=1 SV=2 - [PP1R7_MOUSE]	Ppp1r7	23.95591148	0	0	0	0	0	7.985303826	0	13.83095194	0	0.504985075	0.764125594	only in mock	only in mock
Q497I4	Keratin, type I cuticular Ha5 OS=Mus musculus OX=10090 GN=Krt35 PE=1 SV=1 - [KRT35_MOUSE]	Krt35	0	296.8600833	0	0	0	0	98.9533611	0	171.392249	0	0.504985075	0.764125594	only in mock	only in mock
Q61112	45 kDa calcium-binding protein OS=Mus musculus OX=10090 GN=Sdf4 PE=1 SV=1 - [CAB45_MOUSE]	Sdf4	0	5.107678799	0	0	0	0	1.7025596	0	2.94891973	0	0.504985075	0.764125594	only in mock	only in mock
Q61765	Keratin, type I cuticular Ha1 OS=Mus musculus OX=10090 GN=Krt31 PE=1 SV=2 - [K1H1_MOUSE]	Krt31	0	323.7103969	0	0	0	0	107.9034656	0	186.8942848	0	0.504985075	0.764125594	only in mock	only in mock
Q62168	Keratin, type I cuticular Ha2 OS=Mus musculus OX=10090 GN=Krt32 PE=1 SV=2 - [K1H2_MOUSE]	Krt32	0	428.3113924	0	0	0	0	142.7704641	0	247.2856977	0	0.504985075	0.764125594	only in mock	only in mock
Q64520	Guanylate kinase OS=Mus musculus OX=10090 GN=Guk1 PE=1 SV=2 - [KGUA_MOUSE]	Guk1	37.09525984	0	0	0	0	0	12.36508661	0	21.41695826	0	0.504985075	0.764125594	only in mock	only in mock
Q64707	U2 small nuclear ribonucleoprotein auxiliary factor 35 kDa subunit-related protein 1 OS=Mus musculus OX=10090 GN=Zrsr1 PE=1 SV=1 - [U2AFL_MOUSE]	Zrsr1	10.81148408	0	0	0	0	0	3.603828027	0	6.242013244	0	0.504985075	0.764125594	only in mock	only in mock
Q69ZK7	Protein FAM214A OS=Mus musculus OX=10090 GN=Fam214a PE=2 SV=3 - [F214A_MOUSE]	Fam214a	0	655.1270643	0	0	0	0	218.3756881	0	378.2377869	0	0.504985075	0.764125594	only in mock	only in mock
Q6A065	Centrosomal protein of 170 kDa OS=Mus musculus OX=10090 GN=Cep170 PE=1 SV=2 - [CE170_MOUSE]	Cep170	28.16238549	0	0	0	0	0	9.387461831	0	16.25956085	0	0.504985075	0.764125594	only in mock	only in mock
Q6IME9	Keratin, type II cytoskeletal 72 OS=Mus musculus OX=10090 GN=Krt72 PE=3 SV=1 - [K2C72_MOUSE]	Krt72	0	62.98926417	0	0	0	0	20.99642139	0	36.36686862	0	0.504985075	0.764125594	only in mock	only in mock
Q6KAR6	Exocyst complex component 3 OS=Mus musculus OX=10090 GN=Exoc3 PE=1 SV=2 - [EXOC3_MOUSE]	Exoc3	7.393805109	0	0	0	0	0	2.464601703	0	4.26881537	0	0.504985075	0.764125594	only in mock	only in mock
Q6NS59	Protein FAM135A OS=Mus musculus OX=10090 GN=Fam135a PE=1 SV=2 - [F135A_MOUSE]	Fam135a	875.3086656	0	0	0	0	0	291.7695552	0	505.3596937	0	0.504985075	0.764125594	only in mock	only in mock
Q6NV83	U2 snRNP-associated SURP motif-containing protein OS=Mus musculus OX=10090 GN=U2surp PE=1 SV=3 - [SR140_MOUSE]	U2surp	0	0	490.9995564	0	0	0	163.6665188	0	283.4787261	0	0.504985075	0.764125594	only in mock	only in mock
Q6PAJ1	Breakpoint cluster region protein OS=Mus musculus OX=10090 GN=Bcr PE=1 SV=3 - [BCR_MOUSE]	Bcr	3.740106726	0	0	0	0	0	1.246702242	0	2.159351625	0	0.504985075	0.764125594	only in mock	only in mock
Q6VN19	Ran-binding protein 10 OS=Mus musculus OX=10090 GN=Ranbp10 PE=1 SV=2 - [RBP10_MOUSE]	Ranbp10	58.50230427	0	0	0	0	0	19.50076809	0	33.77632112	0	0.504985075	0.764125594	only in mock	only in mock
Q6ZQ73	Cullin-associated NEDD8-dissociated protein 2 OS=Mus musculus OX=10090 GN=Cand2 PE=1 SV=2 - [CAND2_MOUSE]	Cand2	6.349353724	0	0	0	0	0	2.116451241	0	3.665801081	0	0.504985075	0.764125594	only in mock	only in mock
Q80XI3	Eukaryotic translation initiation factor 4 gamma 3 OS=Mus musculus OX=10090 GN=Eif4g3 PE=1 SV=2 - [IF4G3_MOUSE]	Eif4g3	19.8381126	0	0	0	0	0	6.612704199	0	11.45353965	0	0.504985075	0.764125594	only in mock	only in mock
Q80Z10	Astrotactin-2 OS=Mus musculus OX=10090 GN=Astn2 PE=1 SV=2 - [ASTN2_MOUSE]	Astn2	0	4.903041383	0	0	0	0	1.634347128	0	2.830772263	0	0.504985075	0.764125594	only in mock	only in mock
Q80ZA4	Fibrocystin-L OS=Mus musculus OX=10090 GN=Pkhd1l1 PE=1 SV=1 - [PKHL1_MOUSE]	Pkhd1l1	0	18.94931808	0	0	0	0	6.316439359	0	10.94039389	0	0.504985075	0.764125594	only in mock	only in mock
Q8BGZ7	Keratin, type II cytoskeletal 75 OS=Mus musculus OX=10090 GN=Krt75 PE=1 SV=1 - [K2C75_MOUSE]	Krt75	0	0	231.3223861	0	0	0	77.10746202	0	133.5540419	0	0.504985075	0.764125594	only in mock	only in mock
Q8BHC9	Alpha-(1,3)-fucosyltransferase 11 OS=Mus musculus OX=10090 GN=Fut11 PE=1 SV=1 - [FUT11_MOUSE]	Fut11	0	27.34498738	0	0	0	0	9.114995793	0	15.78763582	0	0.504985075	0.764125594	only in mock	only in mock
Q8BI84	Transport and Golgi organization protein 1 homolog OS=Mus musculus OX=10090 GN=Mia3 PE=1 SV=2 - [TGO1_MOUSE]	Mia3	0	16.77583319	0	0	0	0	5.591944396	0	9.685531807	0	0.504985075	0.764125594	only in mock	only in mock
Q8BID6	Zinc finger and BTB domain-containing protein 46 OS=Mus musculus OX=10090 GN=Zbtb46 PE=1 SV=2 - [ZBT46_MOUSE]	Zbtb46	9.658454458	0	0	0	0	0	3.219484819	0	5.576311281	0	0.504985075	0.764125594	only in mock	only in mock
Q8BJA3	Homeobox-containing protein 1 OS=Mus musculus OX=10090 GN=Hmbox1 PE=1 SV=1 - [HMBX1_MOUSE]	Hmbox1	0	0	47.52803319	0	0	0	15.84267773	0	27.44032276	0	0.504985075	0.764125594	only in mock	only in mock
Q8BKX7	Zinc finger protein 410 OS=Mus musculus OX=10090 GN=Znf410 PE=2 SV=2 - [ZN410_MOUSE]	Znf410	0	0	1134.379853	0	0	0	378.1266177	0	654.9345136	0	0.504985075	0.764125594	only in mock	only in mock
Q8BMJ3	Eukaryotic translation initiation factor 1A, X-chromosomal OS=Mus musculus OX=10090 GN=Eif1ax PE=2 SV=3 - [IF1AX_MOUSE]	Eif1ax	16.59843213	0	0	0	0	0	5.532810709	0	9.583109256	0	0.504985075	0.764125594	only in mock	only in mock
Q8BSL7	ADP-ribosylation factor 2 OS=Mus musculus OX=10090 GN=Arf2 PE=1 SV=2 - [ARF2_MOUSE]	Arf2	0	182.4596551	0	0	0	0	60.81988504	0	105.343131	0	0.504985075	0.764125594	only in mock	only in mock
Q8BTU1	Cilia- and flagella-associated protein 20 OS=Mus musculus OX=10090 GN=Cfap20 PE=1 SV=1 - [CFA20_MOUSE]	Cfap20	12.94991955	0	0	0	0	0	4.316639851	0	7.47663954	0	0.504985075	0.764125594	only in mock	only in mock
Q8BUR4	Dedicator of cytokinesis protein 1 OS=Mus musculus OX=10090 GN=Dock1 PE=1 SV=3 - [DOCK1_MOUSE]	Dock1	19.93714315	0	0	0	0	0	6.645714383	0	11.51071496	0	0.504985075	0.764125594	only in mock	only in mock
Q8BZW8	NHL repeat-containing protein 2 OS=Mus musculus OX=10090 GN=Nhlrc2 PE=1 SV=1 - [NHLC2_MOUSE]	Nhlrc2	11.72886258	0	0	0	0	0	3.909620859	0	6.771661967	0	0.504985075	0.764125594	only in mock	only in mock
Q8C2Q3	RNA-binding protein 14 OS=Mus musculus OX=10090 GN=Rbm14 PE=1 SV=1 - [RBM14_MOUSE]	Rbm14	11.13188213	0	0	0	0	0	3.710627377	0	6.426995145	0	0.504985075	0.764125594	only in mock	only in mock
Q8C570	mRNA export factor OS=Mus musculus OX=10090 GN=Rae1 PE=1 SV=1 - [RAE1L_MOUSE]	Rae1	85.32376065	0	0	0	0	0	28.44125355	0	49.26169618	0	0.504985075	0.764125594	only in mock	only in mock
Q8CBE3	WD repeat-containing protein 37 OS=Mus musculus OX=10090 GN=Wdr37 PE=1 SV=1 - [WDR37_MOUSE]	Wdr37	10.08300738	0	0	0	0	0	3.36100246	0	5.821427024	0	0.504985075	0.764125594	only in mock	only in mock
Q8CCS6	Polyadenylate-binding protein 2 OS=Mus musculus OX=10090 GN=Pabpn1 PE=1 SV=3 - [PABP2_MOUSE]	Pabpn1	55.57007264	0	0	0	0	0	18.52335755	0	32.0833964	0	0.504985075	0.764125594	only in mock	only in mock
Q8CG79	Apoptosis-stimulating of p53 protein 2 OS=Mus musculus OX=10090 GN=Tp53bp2 PE=1 SV=3 - [ASPP2_MOUSE]	Tp53bp2	20946.33193	0	0	0	0	0	6982.110645	0	12093.37038	0	0.504985075	0.764125594	only in mock	only in mock
Q8CGY8	UDP-N-acetylglucosamine--peptide N-acetylglucosaminyltransferase 110 kDa subunit OS=Mus musculus OX=10090 GN=Ogt PE=1 SV=2 - [OGT1_MOUSE]	Ogt	20.48225423	0	0	0	0	0	6.827418076	0	11.82543499	0	0.504985075	0.764125594	only in mock	only in mock
Q8CI70	Leucine-rich repeat-containing protein 20 OS=Mus musculus OX=10090 GN=Lrrc20 PE=1 SV=1 - [LRC20_MOUSE]	Lrrc20	0	36.60330058	0	0	0	0	12.20110019	0	21.13292544	0	0.504985075	0.764125594	only in mock	only in mock
Q8CJG0	Protein argonaute-2 OS=Mus musculus OX=10090 GN=Ago2 PE=1 SV=3 - [AGO2_MOUSE]	Ago2	23.1334216	0	0	0	0	0	7.711140532	0	13.35608719	0	0.504985075	0.764125594	only in mock	only in mock
Q8K003	Translation machinery-associated protein 7 OS=Mus musculus OX=10090 GN=Tma7 PE=3 SV=1 - [TMA7_MOUSE]	Tma7	0	79.73824084	0	0	0	0	26.57941361	0	46.03689481	0	0.504985075	0.764125594	only in mock	only in mock
Q8K1E0	Syntaxin-5 OS=Mus musculus OX=10090 GN=Stx5 PE=1 SV=3 - [STX5_MOUSE]	Stx5	26.55484202	0	0	0	0	0	8.851614007	0	15.33144519	0	0.504985075	0.764125594	only in mock	only in mock
Q8K4G1	Latent-transforming growth factor beta-binding protein 4 OS=Mus musculus OX=10090 GN=Ltbp4 PE=1 SV=2 - [LTBP4_MOUSE]	Ltbp4	0	9.416688899	0	0	0	0	3.1388963	0	5.436727871	0	0.504985075	0.764125594	only in mock	only in mock
Q8K4P0	pre-mRNA 3' end processing protein WDR33 OS=Mus musculus OX=10090 GN=Wdr33 PE=1 SV=1 - [WDR33_MOUSE]	Wdr33	3.800037202	0	0	0	0	0	1.266679067	0	2.193952502	0	0.504985075	0.764125594	only in mock	only in mock
Q8N7N5	DDB1- and CUL4-associated factor 8 OS=Mus musculus OX=10090 GN=Dcaf8 PE=1 SV=1 - [DCAF8_MOUSE]	Dcaf8	13.63557314	0	0	0	0	0	4.545191046	0	7.872501822	0	0.504985075	0.764125594	only in mock	only in mock
Q8R059	UDP-glucose 4-epimerase OS=Mus musculus OX=10090 GN=Gale PE=1 SV=1 - [GALE_MOUSE]	Gale	11.28491093	0	0	0	0	0	3.761636977	0	6.515346364	0	0.504985075	0.764125594	only in mock	only in mock
Q8R0A0	General transcription factor IIF subunit 2 OS=Mus musculus OX=10090 GN=Gtf2f2 PE=1 SV=1 - [T2FB_MOUSE]	Gtf2f2	5.919997602	0	0	0	0	0	1.973332534	0	3.417912209	0	0.504985075	0.764125594	only in mock	only in mock
Q8R0P4	Mth938 domain-containing protein OS=Mus musculus OX=10090 GN=Aamdc PE=1 SV=1 - [AAMDC_MOUSE]	Aamdc	35.09586891	0	0	0	0	0	11.69862297	0	20.26260936	0	0.504985075	0.764125594	only in mock	only in mock
Q8R0Z6	Angiopoietin-related protein 6 OS=Mus musculus OX=10090 GN=Angptl6 PE=2 SV=1 - [ANGL6_MOUSE]	Angptl6	0	0	24.33901407	0	0	0	8.11300469	0	14.05213633	0	0.504985075	0.764125594	only in mock	only in mock
Q8R5K2	Ubiquitin carboxyl-terminal hydrolase 33 OS=Mus musculus OX=10090 GN=Usp33 PE=1 SV=2 - [UBP33_MOUSE]	Usp33	0	0	36.10477877	0	0	0	12.03492626	0	20.84510374	0	0.504985075	0.764125594	only in mock	only in mock
Q8VC97	Beta-ureidopropionase OS=Mus musculus OX=10090 GN=Upb1 PE=1 SV=1 - [BUP1_MOUSE]	Upb1	0	42.89232343	0	0	0	0	14.29744114	0	24.76389448	0	0.504985075	0.764125594	only in mock	only in mock
Q8VDM6	Heterogeneous nuclear ribonucleoprotein U-like protein 1 OS=Mus musculus OX=10090 GN=Hnrnpul1 PE=1 SV=1 - [HNRL1_MOUSE]	Hnrnpul1	10.32018466	0	0	0	0	0	3.440061555	0	5.958361394	0	0.504985075	0.764125594	only in mock	only in mock
Q91VA6	Polymerase delta-interacting protein 2 OS=Mus musculus OX=10090 GN=Poldip2 PE=1 SV=1 - [PDIP2_MOUSE]	Poldip2	0	16.68478446	0	0	0	0	5.561594821	0	9.632964801	0	0.504985075	0.764125594	only in mock	only in mock
Q91VC4	Plasmalemma vesicle-associated protein OS=Mus musculus OX=10090 GN=Plvap PE=1 SV=1 - [PLVAP_MOUSE]	Plvap	0	0	32.88603891	0	0	0	10.96201297	0	18.98676341	0	0.504985075	0.764125594	only in mock	only in mock
Q91WM2	Haloacid dehalogenase-like hydrolase domain-containing 5 OS=Mus musculus OX=10090 GN=Hdhd5 PE=1 SV=1 - [HDHD5_MOUSE]	Hdhd5	10.01707172	0	0	0	0	0	3.339023907	0	5.783359055	0	0.504985075	0.764125594	only in mock	only in mock
Q91XM9	Disks large homolog 2 OS=Mus musculus OX=10090 GN=Dlg2 PE=1 SV=2 - [DLG2_MOUSE]	Dlg2	0	23.19638483	0	0	0	0	7.732128277	0	13.39243903	0	0.504985075	0.764125594	only in mock	only in mock
Q921T2	Torsin-1A-interacting protein 1 OS=Mus musculus OX=10090 GN=Tor1aip1 PE=1 SV=3 - [TOIP1_MOUSE]	Tor1aip1	0	24.83759862	0	0	0	0	8.279199539	0	14.33999425	0	0.504985075	0.764125594	only in mock	#NUM!
Q922V4	Pleiotropic regulator 1 OS=Mus musculus OX=10090 GN=Plrg1 PE=1 SV=1 - [PLRG1_MOUSE]	Plrg1	7.69360498	0	0	0	0	0	2.564534993	0	4.441904906	0	0.504985075	0.764125594	only in mock	#NUM!
Q923B0	Gamma-glutamylaminecyclotransferase OS=Mus musculus OX=10090 GN=Ggact PE=1 SV=1 - [GGACT_MOUSE]	Ggact	9.067087274	0	0	0	0	0	3.022362425	0	5.234885278	0	0.504985075	0.764125594	only in mock	#NUM!
Q923G2	DNA-directed RNA polymerases I, II, and III subunit RPABC3 OS=Mus musculus OX=10090 GN=Polr2h PE=1 SV=3 - [RPAB3_MOUSE]	Polr2h	0	39.6135565	0	0	0	0	13.20451883	0	22.87089751	0	0.504985075	0.764125594	only in mock	#NUM!
Q99M74	Keratin, type II cuticular Hb2 OS=Mus musculus OX=10090 GN=Krt82 PE=1 SV=2 - [KRT82_MOUSE]	Krt82	0	206.8316743	0	0	0	0	68.94389144	0	119.4143228	0	0.504985075	0.764125594	only in mock	#NUM!
Q9CYL5	Golgi-associated plant pathogenesis-related protein 1 OS=Mus musculus OX=10090 GN=Glipr2 PE=1 SV=3 - [GAPR1_MOUSE]	Glipr2	0	34.56698535	0	0	0	0	11.52232845	0	19.9572583	0	0.504985075	0.764125594	only in mock	#NUM!
Q9CZT8	Ras-related protein Rab-3B OS=Mus musculus OX=10090 GN=Rab3b PE=1 SV=1 - [RAB3B_MOUSE]	Rab3b	190.0537548	0	0	0	0	0	63.35125162	0	109.7275865	0	0.504985075	0.764125594	only in mock	#NUM!
Q9D187	Cytosolic iron-sulfur assembly component 2B OS=Mus musculus OX=10090 GN=Ciao2b PE=1 SV=1 - [CIA2B_MOUSE]	Ciao2b	9.006076415	0	0	0	0	0	3.002025472	0	5.199660643	0	0.504985075	0.764125594	only in mock	#NUM!
Q9D1K7	UPF0687 protein C20orf27 homolog OS=Mus musculus OX=10090 PE=1 SV=3 - [CT027_MOUSE]	Not availa	48.86887501	0	0	0	0	0	16.289625	0	28.21445814	0	0.504985075	0.764125594	only in mock	#NUM!
Q9D2F7	Nuclear pore membrane glycoprotein 210-like OS=Mus musculus OX=10090 GN=Nup210l PE=1 SV=2 - [P210L_MOUSE]	Nup210l	0	41.10850801	0	0	0	0	13.702836	0	23.73400817	0	0.504985075	0.764125594	only in mock	#NUM!
Q9D6F9	Tubulin beta-4A chain OS=Mus musculus OX=10090 GN=Tubb4a PE=1 SV=3 - [TBB4A_MOUSE]	Tubb4a	1128.202431	0	0	0	0	0	376.0674771	0	651.3679774	0	0.504985075	0.764125594	only in mock	#NUM!
Q9D6J6	NADH dehydrogenase [ubiquinone] flavoprotein 2, mitochondrial OS=Mus musculus OX=10090 GN=Ndufv2 PE=1 SV=2 - [NDUV2_MOUSE]	Ndufv2	0	72.38150744	0	0	0	0	24.12716915	0	41.7894828	0	0.504985075	0.764125594	only in mock	#NUM!
Q9D7M8	DNA-directed RNA polymerase II subunit RPB4 OS=Mus musculus OX=10090 GN=Polr2d PE=1 SV=2 - [RPB4_MOUSE]	Polr2d	6.883978354	0	0	0	0	0	2.294659451	0	3.974466756	0	0.504985075	0.764125594	only in mock	#NUM!
Q9D832	DnaJ homolog subfamily B member 4 OS=Mus musculus OX=10090 GN=Dnajb4 PE=1 SV=1 - [DNJB4_MOUSE]	Dnajb4	8.090872666	0	0	0	0	0	2.696957555	0	4.671267512	0	0.504985075	0.764125594	only in mock	#NUM!
Q9D968	Host cell factor 2 OS=Mus musculus OX=10090 GN=Hcfc2 PE=1 SV=2 - [HCFC2_MOUSE]	Hcfc2	0	0	26.53011222	0	0	0	8.84337074	0	15.31716743	0	0.504985075	0.764125594	only in mock	#NUM!
Q9DBE0	Cysteine sulfinic acid decarboxylase OS=Mus musculus OX=10090 GN=Csad PE=1 SV=1 - [CSAD_MOUSE]	Csad	38.42826014	0	0	0	0	0	12.80942005	0	22.18656634	0	0.504985075	0.764125594	only in mock	#NUM!
Q9DC11	Plexin domain-containing protein 2 OS=Mus musculus OX=10090 GN=Plxdc2 PE=1 SV=1 - [PXDC2_MOUSE]	Plxdc2	0	0	16.13146803	0	0	0	5.37715601	0	9.313507409	0	0.504985075	0.764125594	only in mock	#NUM!
Q9DCW4	Electron transfer flavoprotein subunit beta OS=Mus musculus OX=10090 GN=Etfb PE=1 SV=3 - [ETFB_MOUSE]	Etfb	9.434834815	0	0	0	0	0	3.144944938	0	5.44720442	0	0.504985075	0.764125594	only in mock	#NUM!
Q9EPR5	VPS10 domain-containing receptor SorCS2 OS=Mus musculus OX=10090 GN=Sorcs2 PE=1 SV=2 - [SORC2_MOUSE]	Sorcs2	17.74676521	0	0	0	0	0	5.915588405	0	10.24609967	0	0.504985075	0.764125594	only in mock	#NUM!
Q9EPU5	Tumor necrosis factor receptor superfamily member 21 OS=Mus musculus OX=10090 GN=Tnfrsf21 PE=1 SV=2 - [TNR21_MOUSE]	Tnfrsf21	35.98956828	0	0	0	0	0	11.99652276	0	20.77858693	0	0.504985075	0.764125594	only in mock	#NUM!
Q9ESW8	Pyroglutamyl-peptidase 1 OS=Mus musculus OX=10090 GN=Pgpep1 PE=1 SV=1 - [PGPI_MOUSE]	Pgpep1	11.54554862	0	0	0	0	0	3.848516208	0	6.665825606	0	0.504985075	0.764125594	only in mock	#NUM!
Q9JHI7	Exosome complex component RRP45 OS=Mus musculus OX=10090 GN=Exosc9 PE=1 SV=1 - [EXOS9_MOUSE]	Exosc9	122.3152992	0	0	0	0	0	40.7717664	0	70.61877091	0	0.504985075	0.764125594	only in mock	#NUM!
Q9JHS3	Ragulator complex protein LAMTOR2 OS=Mus musculus OX=10090 GN=Lamtor2 PE=1 SV=1 - [LTOR2_MOUSE]	Lamtor2	12.25469969	0	0	0	0	0	4.084899897	0	7.075254165	0	0.504985075	0.764125594	only in mock	#NUM!
Q9JJW6	Aly/REF export factor 2 OS=Mus musculus OX=10090 GN=Alyref2 PE=1 SV=1 - [ALRF2_MOUSE]	Alyref2	0	0	482.3071208	0	0	0	160.7690403	0	278.4601461	0	0.504985075	0.764125594	only in mock	#NUM!
Q9JKC7	AP-4 complex subunit mu-1 OS=Mus musculus OX=10090 GN=Ap4m1 PE=1 SV=1 - [AP4M1_MOUSE]	Ap4m1	16.16856641	0	0	0	0	0	5.389522138	0	9.334926171	0	0.504985075	0.764125594	only in mock	#NUM!
Q9JMD0	BUB3-interacting and GLEBS motif-containing protein ZNF207 OS=Mus musculus OX=10090 GN=Znf207 PE=1 SV=1 - [ZN207_MOUSE]	Znf207	94.73260648	0	0	0	0	0	31.57753549	0	54.69389585	0	0.504985075	0.764125594	only in mock	#NUM!
Q9QX66	Zinc finger protein neuro-d4 OS=Mus musculus OX=10090 GN=Dpf1 PE=1 SV=2 - [DPF1_MOUSE]	Dpf1	0	0	111.8162612	0	0	0	37.27208707	0	64.5571485	0	0.504985075	0.764125594	only in mock	#NUM!
Q9QZV9	NTF2-related export protein 1 OS=Mus musculus OX=10090 GN=Nxt1 PE=1 SV=2 - [NXT1_MOUSE]	Nxt1	0	0	22.65352849	0	0	0	7.551176163	0	13.07902077	0	0.504985075	0.764125594	only in mock	#NUM!
Q9R045	Angiopoietin-related protein 2 OS=Mus musculus OX=10090 GN=Angptl2 PE=2 SV=2 - [ANGL2_MOUSE]	Angptl2	24.5374082	0	0	0	0	0	8.179136065	0	14.16667923	0	0.504985075	0.764125594	only in mock	#NUM!
Q9R0K7	Plasma membrane calcium-transporting ATPase 2 OS=Mus musculus OX=10090 GN=Atp2b2 PE=1 SV=2 - [AT2B2_MOUSE]	Atp2b2	15.48084516	0	0	0	0	0	5.160281722	0	8.937870123	0	0.504985075	0.764125594	only in mock	#NUM!
Q9R226	KH domain-containing, RNA-binding, signal transduction-associated protein 3 OS=Mus musculus OX=10090 GN=Khdrbs3 PE=1 SV=1 - [KHDR3_MOUSE]	Khdrbs3	0	93.37743737	0	0	0	0	31.12581246	0	53.9114886	0	0.504985075	0.764125594	only in mock	#NUM!
Q9WV80	Sorting nexin-1 OS=Mus musculus OX=10090 GN=Snx1 PE=1 SV=1 - [SNX1_MOUSE]	Snx1	15.01861986	0	0	0	0	0	5.006206621	0	8.671004221	0	0.504985075	0.764125594	only in mock	#NUM!
Q9Z110	Delta-1-pyrroline-5-carboxylate synthase OS=Mus musculus OX=10090 GN=Aldh18a1 PE=1 SV=2 - [P5CS_MOUSE]	Aldh18a1	9.113720995	0	0	0	0	0	3.037906998	0	5.26180927	0	0.504985075	0.764125594	only in mock	#NUM!
Q9Z1L3	Death effector domain-containing protein OS=Mus musculus OX=10090 GN=Dedd PE=2 SV=1 - [DEDD_MOUSE]	Dedd	87.29361406	0	0	0	0	0	29.09787135	0	50.39899158	0	0.504985075	0.764125594	only in mock	#NUM!
Q9Z2T6	Keratin, type II cuticular Hb5 OS=Mus musculus OX=10090 GN=Krt85 PE=1 SV=2 - [KRT85_MOUSE]	Krt85	0	194.295742	0	0	0	0	64.76524733	0	112.1766989	0	0.504985075	0.764125594	only in mock	#NUM!
Q9Z2V5	Histone deacetylase 6 OS=Mus musculus OX=10090 GN=Hdac6 PE=1 SV=3 - [HDAC6_MOUSE]	Hdac6	7.842236177	0	0	0	0	0	2.614078726	0	4.527717168	0	0.504985075	0.764125594	only in mock	#NUM!
P29699	Alpha-2-HS-glycoprotein OS=Mus musculus OX=10090 GN=Ahsg PE=1 SV=1 - [FETUA_MOUSE]	Ahsg	2550.96091	0	1613.683335	1282.772793	325.9459872	0	1388.214748	536.2395936	1290.340054	666.7413746	0.506555169	0.764125594	0.38628	-1.372281111
O55013	Trafficking protein particle complex subunit 3 OS=Mus musculus OX=10090 GN=Trappc3 PE=1 SV=1 - [TPPC3_MOUSE]	Trappc3	0	36.70952803	36.34010036	0	21.17087143	20.09992507	24.34987613	13.75693217	21.08842028	11.9258802	0.506555169	0.764125594	0.564969287	-0.823755652

P02301	Histone H3.3C OS=Mus musculus OX=10090 GN=H3-5 PE=3 SV=3 - [H3C_MOUSE]	H3-5	1658.708343	1306.231678	0	929.7555083	958.7253914	0	988.3133403	629.4936332	873.8606261	545.3498778	0.506555169	0.764125594	0.636937303	-0.650776728
Q9JM58	Cytokine receptor-like factor 1 OS=Mus musculus OX=10090 GN=Crlf1 PE=1 SV=1 - [CRLF1_MOUSE]	Crlf1	0	15.725634	16.10855405	0	12.15819651	12.6942715	10.61139602	8.284156006	9.191732753	7.179294857	0.506555169	0.764125594	0.78068484	-0.35718784
P50428	Arylsulfatase A OS=Mus musculus OX=10090 GN=Arsa PE=1 SV=2 - [ARSA_MOUSE]	Arsa	26.50417859	0	32.07156098	24.25540013	22.60749462	0	19.52524652	15.62096491	17.13695934	13.55322127	0.506555169	0.764125594	0.800039318	-0.321857192
Q80WV3	Carbohydrate sulfotransferase 2 OS=Mus musculus OX=10090 GN=Chst2 PE=2 SV=3 - [CHST2_MOUSE]	Chst2	30.3469262	0	26.42594274	0	31.92184112	47.68024433	18.92428965	26.53402848	16.50575905	24.29244365	0.506555169	0.764125594	1.4021149	0.48760458
Q32MD9	Cell adhesion molecule-related/down-regulated by oncogenes OS=Mus musculus OX=10090 GN=Cdon PE=1 SV=2 - [CDON_MOUSE]	Cdon	27.4818509	0	37.68647118	0	40.59462487	51.48145375	21.72277403	30.69202621	19.49212026	27.13173631	0.506555169	0.764125594	1.412896261	0.498655543
Q9D1J1	Adaptin ear-binding coat-associated protein 2 OS=Mus musculus OX=10090 GN=Necap2 PE=1 SV=1 - [NECP2_MOUSE]	Necap2	50.20237042	0	25.20961819	52.1463247	56.34067438	0	25.13732954	36.16233303	25.10126328	31.38763912	0.506555169	0.764125594	1.43859088	0.524656363
Q8BU31	Ras-related protein Rap-2c OS=Mus musculus OX=10090 GN=Rap2c PE=1 SV=1 - [RAP2C_MOUSE]	Rap2c	11.3297895	9.766230901	0	0	17.10233504	16.68034624	7.032006801	11.26089376	6.139871221	9.754502286	0.506555169	0.764125594	1.601376972	0.679312966
P97470	Serine/threonine-protein phosphatase 4 catalytic subunit OS=Mus musculus OX=10090 GN=Ppp4c PE=1 SV=2 - [PP4C_MOUSE]	Ppp4c	117.8461758	46.85603307	0	134.9078546	136.4475249	0	54.90073628	90.45179317	59.33353371	78.33733345	0.506555169	0.764125594	1.64755155	0.720323607
P35235	Tyrosine-protein phosphatase non-receptor type 11 OS=Mus musculus OX=10090 GN=Ptpn11 PE=1 SV=3 - [PTN11_MOUSE]	Ptpn11	70.43510776	23.47836925	0	107.5801828	70.46987323	0	31.30449234	59.35001868	35.86380143	54.64533198	0.506555169	0.764125594	1.895894623	0.922878779
Q6ZWN5	40S ribosomal protein S9 OS=Mus musculus OX=10090 GN=Rps9 PE=1 SV=3 - [RS9_MOUSE]	Rps9	73.2635026	0	19.85305272	82.34624639	110.6949159	0	31.03885177	64.34705409	37.89098545	57.50060254	0.506555169	0.764125594	2.073113225	1.051798913
P42567	Epidermal growth factor receptor substrate 15 OS=Mus musculus OX=10090 GN=Eps15 PE=1 SV=1 - [EPS15_MOUSE]	Eps15	15.14296437	5.639883646	0	20.85519151	23.81230893	0	6.927616006	14.88916681	7.653171465	12.97889064	0.506555169	0.764125594	2.149248284	1.103832155
Q9D6X6	Serine protease 23 OS=Mus musculus OX=10090 GN=Prss23 PE=2 SV=2 - [PRS23_MOUSE]	Prss23	41.51868431	28.85428548	0	86.42141807	69.84673135	0	23.45765659	52.08938314	21.27893299	45.86565095	0.506555169	0.764125594	2.220570624	1.150930456
Q09163	Protein delta homolog 1 OS=Mus musculus OX=10090 GN=Dlk1 PE=1 SV=1 - [DLK1_MOUSE]	Dlk1	1520.271951	2890.751841	0	5482.357956	5335.173297	0	1470.341264	3605.843751	1446.022599	3123.619327	0.506555169	0.764125594	2.452385605	1.294185842
P49962	Signal recognition particle 9 kDa protein OS=Mus musculus OX=10090 GN=Srp9 PE=1 SV=2 - [SRP09_MOUSE]	Srp9	0	65.9284559	107.5312327	0	265.2621198	180.6167733	57.81989621	148.6262977	54.22225612	135.4936934	0.506555169	0.764125594	2.570504401	1.362051482
P04925	Major prion protein OS=Mus musculus OX=10090 GN=Prnp PE=1 SV=2 - [PRIO_MOUSE]	Prnp	134.340494	58.34212236	0	0	331.4908061	266.1137333	64.22753878	199.2015131	67.36334816	175.5832353	0.506555169	0.764125594	3.101496911	1.632964688
Q810Y8	Preferentially expressed antigen in melanoma-like protein 7 OS=Mus musculus OX=10090 GN=Pramel7 PE=1 SV=1 - [PRAL7_MOUSE]	Pramel7	19.51729109	0	37.76275645	20.2196661	0	0	19.09334918	6.739888701	18.88494741	11.67382967	0.642834826	0.838405797	0.352996671	-1.502273516
P41105	60S ribosomal protein L28 OS=Mus musculus OX=10090 GN=Rpl28 PE=1 SV=2 - [RL28_MOUSE]	Rpl28	91.76661425	30.41910349	0	0	0	46.66745426	40.72857258	15.55581809	46.74389519	26.94346728	0.642834826	0.838405797	0.381938701	-1.388586984
P57016	Ladinin-1 OS=Mus musculus OX=10090 GN=Lad1 PE=1 SV=1 - [LAD1_MOUSE]	Lad1	88.34367188	0	40.28057614	51.59264724	0	0	42.87474934	17.19754908	44.22893161	29.78702877	0.642834826	0.838405797	0.401111361	-1.317925264
Q99LJ6	Glutathione peroxidase 7 OS=Mus musculus OX=10090 GN=Gpx7 PE=1 SV=1 - [GPX7_MOUSE]	Gpx7	0	35.74929918	107.2478975	0	0	70.59479751	47.66573224	23.53159917	54.60795651	40.75792535	0.642834826	0.838405797	0.49367959	-1.018353092
Q6P9J9	Anoctamin-6 OS=Mus musculus OX=10090 GN=Ano6 PE=1 SV=1 - [ANO6_MOUSE]	Ano6	0	23.46328464	19.72302733	0	0	21.55949695	14.39543732	7.186498983	12.60630172	12.44738137	0.642834826	0.838405797	0.499220609	-1.002250602
Q80SW1	S-adenosylhomocysteine hydrolase-like protein 1 OS=Mus musculus OX=10090 GN=Ahcyl1 PE=1 SV=1 - [SAHH2_MOUSE]	Ahcyl1	179.6579506	33.50287046	0	0	117.4837998	0	71.05360702	39.16126661	95.53422511	67.82930347	0.642834826	0.838405797	0.551151001	-0.859480462
Q62108	Disks large homolog 4 OS=Mus musculus OX=10090 GN=Dlg4 PE=1 SV=1 - [DLG4_MOUSE]	Dlg4	19.43396354	0	14.4980485	19.40015739	0	0	11.31067068	6.466719131	10.1014488	11.20068609	0.642834826	0.838405797	0.571736134	-0.806578622
Q3ULJ0	Glycerol-3-phosphate dehydrogenase 1-like protein OS=Mus musculus OX=10090 GN=Gpd1l PE=1 SV=2 - [GPD1L_MOUSE]	Gpd1l	58.81478475	0	0	14.79264236	65.50790661	0	19.60492825	26.76684966	33.95673181	34.35633402	0.642834826	0.838405797	1.365312299	0.449230988
Q6ZQL4	WD repeat-containing protein 43 OS=Mus musculus OX=10090 GN=Wdr43 PE=1 SV=2 - [WDR43_MOUSE]	Wdr43	34.53177174	0	0	11.03233957	37.86648518	0	11.51059058	16.29960825	19.93692771	19.47500358	0.642834826	0.838405797	1.416053167	0.501875434
Q99KK2	N-acylneuraminate cytidylyltransferase OS=Mus musculus OX=10090 GN=Cmas PE=1 SV=2 - [NEUA_MOUSE]	Cmas	28.20962077	0	0	29.41215009	13.5570766	0	9.403206922	14.32307556	16.28683214	14.72102948	0.642834826	0.838405797	1.523211781	0.607116542
Q9ERU9	E3 SUMO-protein ligase RanBP2 OS=Mus musculus OX=10090 GN=Ranbp2 PE=1 SV=2 - [RBP2_MOUSE]	Ranbp2	8.503406677	0	0	10.03528932	3.345096439	0	2.834468892	4.460128585	4.909444134	5.109719213	0.642834826	0.838405797	1.573532381	0.654006868
Q920C1	Chordin-like protein 1 OS=Mus musculus OX=10090 GN=Chrdl1 PE=1 SV=1 - [CRDL1_MOUSE]	Chrdl1	0	58.48901891	0	0	27.18444829	65.30243341	19.49633964	30.82896057	33.76865081	32.80341148	0.642834826	0.838405797	1.581269159	0.661082959
Q91XL9	Oxysterol-binding protein-related protein 1 OS=Mus musculus OX=10090 GN=Osbpl1a PE=1 SV=2 - [OSBL1_MOUSE]	Osbpl1a	28.71523461	0	0	11.28682039	34.9153631	0	9.571744868	15.40072783	16.57874843	17.81751444	0.642834826	0.838405797	1.608978095	0.686144685
P39054	Dynamin-2 OS=Mus musculus OX=10090 GN=Dnm2 PE=1 SV=2 - [DYN2_MOUSE]	Dnm2	19.75360104	0	0	10.27947166	21.78698189	0	6.58453368	10.68881785	11.40474688	10.89925769	0.642834826	0.838405797	1.623321919	0.698949128
Q571I9	Aldehyde dehydrogenase family 16 member A1 OS=Mus musculus OX=10090 GN=Aldh16a1 PE=1 SV=2 - [A16A1_MOUSE]	Aldh16a1	22.83199055	0	0	24.29373916	12.88564113	0	7.610663515	12.39312677	13.18205589	12.15435595	0.642834826	0.838405797	1.62838979	0.703446081
P84084	ADP-ribosylation factor 5 OS=Mus musculus OX=10090 GN=Arf5 PE=1 SV=2 - [ARF5_MOUSE]	Arf5	686.0548534	0	0	341.2908615	781.6876372	0	228.6849511	374.3261662	396.0939543	391.8895113	0.642834826	0.838405797	1.63686401	0.710934469
Q9CR41	Huntingtin-interacting protein K OS=Mus musculus OX=10090 GN=Hypk PE=1 SV=2 - [HYPK_MOUSE]	Hypk	19.46641809	0	0	19.77985106	12.98581219	0	6.48880603	10.92188775	11.23894173	10.05014751	0.642834826	0.838405797	1.683189125	0.751197289
P52624	Uridine phosphorylase 1 OS=Mus musculus OX=10090 GN=Upp1 PE=1 SV=2 - [UPP1_MOUSE]	Upp1	55.35765377	0	0	62.30884508	31.40061141	0	18.45255126	31.2364855	31.96075631	31.15474678	0.642834826	0.838405797	1.692800365	0.759411843
Q922D8	C-1-tetrahydrofolate synthase, cytoplasmic OS=Mus musculus OX=10090 GN=Mthfd1 PE=1 SV=4 - [C1TC_MOUSE]	Mthfd1	34.73524764	0	0	18.96719747	40.15289822	0	11.57841588	19.70669856	20.05440458	20.08666113	0.642834826	0.838405797	1.702020273	0.767248222
P05213	Tubulin alpha-1B chain OS=Mus musculus OX=10090 GN=Tuba1b PE=1 SV=2 - [TBA1B_MOUSE]	Tuba1b	1669.43201	0	0	1179.455999	1718.642947	0	556.4773368	966.0329818	963.8470205	878.9740797	0.642834826	0.838405797	1.73597902	0.795749513
P70335	Rho-associated protein kinase 1 OS=Mus musculus OX=10090 GN=Rock1 PE=1 SV=1 - [ROCK1_MOUSE]	Rock1	16.06329102	0	0	0	19.70184352	8.261178931	5.354430341	9.321007485	9.274145397	9.893588175	0.642834826	0.838405797	1.740802829	0.799752806
Q9D115	Zinc finger protein 706 OS=Mus musculus OX=10090 GN=Znf706 PE=1 SV=1 - [ZN706_MOUSE]	Znf706	104.3461859	0	0	123.9085322	59.38279413	0	34.78206197	61.09710877	60.24429853	61.97205212	0.642834826	0.838405797	1.756569488	0.812760649
Q8VEH5	EPM2A-interacting protein 1 OS=Mus musculus OX=10090 GN=Epm2aip1 PE=1 SV=1 - [EPMIP_MOUSE]	Epm2aip1	44.8987241	0	0	6.586608446	72.4838473	0	14.96624137	26.35681858	25.92229044	40.08270121	0.642834826	0.838405797	1.761084693	0.816464292
Q7TMM9	Tubulin beta-2A chain OS=Mus musculus OX=10090 GN=Tubb2a PE=1 SV=1 - [TBB2A_MOUSE]	Tubb2a	1157.243141	0	0	759.1018221	1286.724856	0	385.7477136	681.9422261	668.1346388	646.823327	0.642834826	0.838405797	1.767845154	0.821991914
Q9CQ92	Mitochondrial fission 1 protein OS=Mus musculus OX=10090 GN=Fis1 PE=1 SV=1 - [FIS1_MOUSE]	Fis1	50.0268119	0	0	39.90448296	51.09554917	0	16.67560397	30.33334404	28.88299332	26.85877532	0.642834826	0.838405797	1.819025212	0.863165539
P14131	40S ribosomal protein S16 OS=Mus musculus OX=10090 GN=Rps16 PE=1 SV=4 - [RS16_MOUSE]	Rps16	144.9114943	0	0	116.2913706	151.881888	0	48.30383144	89.39108622	83.66469025	79.43390935	0.642834826	0.838405797	1.850600326	0.88799335
P70297	Signal transducing adapter molecule 1 OS=Mus musculus OX=10090 GN=Stam PE=1 SV=3 - [STAM1_MOUSE]	Stam	21.18031507	0	0	16.02531483	23.20654575	0	7.060105025	13.07728686	12.22846061	11.8808288	0.642834826	0.838405797	1.852279366	0.889301706
Q9D1R9	60S ribosomal protein L34 OS=Mus musculus OX=10090 GN=Rpl34 PE=1 SV=2 - [RL34_MOUSE]	Rpl34	77.19727736	0	0	54.55932717	90.46252196	0	25.73242579	48.34061638	44.56986887	45.55075458	0.642834826	0.838405797	1.878587615	0.909648403
Q3TIX9	U4/U6.U5 tri-snRNP-associated protein 2 OS=Mus musculus OX=10090 GN=Usp39 PE=1 SV=2 - [SNUT2_MOUSE]	Usp39	37.69458301	0	0	29.30182861	41.65963138	0	12.564861	23.65382	21.76297765	21.39640677	0.642834826	0.838405797	1.882537339	0.91267848
Q91YI0	Argininosuccinate lyase OS=Mus musculus OX=10090 GN=Asl PE=1 SV=1 - [ARLY_MOUSE]	Asl	30.96550438	0	0	33.90628712	24.45005276	0	10.32183479	19.45211329	17.87794229	17.49696047	0.642834826	0.838405797	1.884559643	0.914227455
Q9Z1Z0	General vesicular transport factor p115 OS=Mus musculus OX=10090 GN=Uso1 PE=1 SV=2 - [USO1_MOUSE]	Uso1	26.60785727	0	0	19.68303101	31.93383454	0	8.869285758	17.20562185	15.36205356	16.11041943	0.642834826	0.838405797	1.939910644	0.955990201
Q91Z53	Glyoxylate reductase/hydroxypyruvate reductase OS=Mus musculus OX=10090 GN=Grhpr PE=1 SV=1 - [GRHPR_MOUSE]	Grhpr	34.71322648	0	0	29.2038913	38.24015521	0	11.57107549	22.48134884	20.04169065	19.98679065	0.642834826	0.838405797	1.942891899	0.958205633
Q9Z0P4	Paralemmin-1 OS=Mus musculus OX=10090 GN=Palm PE=1 SV=1 - [PALM_MOUSE]	Palm	31.1828567	0	0	31.29130318	29.46242677	0	10.39428557	20.25124332	18.00343071	17.56191445	0.642834826	0.838405797	1.948305459	0.962219884
P10711	Transcription elongation factor A protein 1 OS=Mus musculus OX=10090 GN=Tcea1 PE=1 SV=2 - [TCEA1_MOUSE]	Tcea1	100.1375954	0	0	100.9668456	94.36533719	0	33.37919847	65.1107276	57.81446766	56.48406957	0.642834826	0.838405797	1.95063784	0.963945949
P14231	Sodium/potassium-transporting ATPase subunit beta-2 OS=Mus musculus OX=10090 GN=Atp1b2 PE=1 SV=2 - [AT1B2_MOUSE]	Atp1b2	50.97219826	0	0	0	54.52809529	45.47214961	16.99073275	33.33341497	29.42881239	29.22053992	0.642834826	0.838405797	1.961858588	0.972221055
B1AZP2	Disks large-associated protein 4 OS=Mus musculus OX=10090 GN=Dlgap4 PE=1 SV=1 - [DLGP4_MOUSE]	Dlgap4	39.15092836	0	0	40.30672593	36.93910232	0	13.05030945	25.74860942	22.60379903	22.3624325	0.642834826	0.838405797	1.973026732	0.980410503
Q3UDR8	Protein YIPF3 OS=Mus musculus OX=10090 GN=Yipf3 PE=1 SV=1 - [YIPF3_MOUSE]	Yipf3	62.3591712	0	0	0	63.65636016	59.93863233	20.7863904	41.19833083	36.00308428	35.72719164	0.642834826	0.838405797	1.981985811	0.986946634
P61255	60S ribosomal protein L26 OS=Mus musculus OX=10090 GN=Rpl26 PE=1 SV=1 - [RL26_MOUSE]	Rpl26	75.41493179	0	0	59.42440163	90.74621875	0	25.1383106	50.05687346	43.54083117	46.09264575	0.642834826	0.838405797	1.991258453	0.993680486
Q8CC86	Nicotinate phosphoribosyltransferase OS=Mus musculus OX=10090 GN=Naprt PE=1 SV=1 - [PNCB_MOUSE]	Naprt	8.474937667	0	0	7.338026588	9.567106134	0	2.824979222	5.635044241	4.893007543	5.005745859	0.642834826	0.838405797	1.994720597	0.996186681
Q80UJ1	Solute carrier family 22 member 20 OS=Mus musculus OX=10090 GN=Slc22a20 PE=1 SV=1 - [S22AK_MOUSE]	Slc22a20	1655.409322	0	0	1702.895384	1613.873943	0	551.8031072	1105.589776	955.7510175	958.5028785	0.642834826	0.838405797	2.00359469	1.002590693
P62855	40S ribosomal protein S26 OS=Mus musculus OX=10090 GN=Rps26 PE=1 SV=3 - [RS26_MOUSE]	Rps26	160.7329957	0	0	128.9798919	193.7527268	0	53.57766524	107.5775396	92.79923834	98.63354045	0.642834826	0.838405797	2.007880319	1.005673279
Q8BVU5	ADP-ribose pyrophosphatase, mitochondrial OS=Mus musculus OX=10090 GN=Nudt9 PE=1 SV=1 - [NUDT9_MOUSE]	Nudt9	42.08044756	0	0	44.24016273	40.39302129	0	14.02681585	28.21106134	24.29515772	24.50710327	0.642834826	0.838405797	2.011223476	1.008073395
Q6PDI6	Ubiquitin carboxyl-terminal hydrolase MINDY-2 OS=Mus musculus OX=10090 GN=Mindy2 PE=1 SV=1 - [MINY2_MOUSE]	Mindy2	0	6.09047749	0	0	8.043129124	4.297395343	2.030159163	4.113508156	3.516338819	4.024716437	0.642834826	0.838405797	2.026199832	1.018776465
Q6ZWX6	Eukaryotic translation initiation factor 2 subunit 1 OS=Mus musculus OX=10090 GN=Eif2s1 PE=1 SV=3 - [IF2A_MOUSE]	Eif2s1	45.71287752	0	0	37.22217785	55.41578249	0	15.23762584	30.87932011	26.39234214	28.24714358	0.642834826	0.838405797	2.026517808	1.019002853
P70333	Heterogeneous nuclear ribonucleoprotein H2 OS=Mus musculus OX=10090 GN=Hnrnph2 PE=1 SV=1 - [HNRH2_MOUSE]	Hnrnph2	307.7224116	0	0	297.2708053	327.907742	0	102.5741372	208.3928491	177.6636172	181.1224452	0.642834826	0.838405797	2.031631509	1.022638754
Q3UA37	Glutamine-rich protein 1 OS=Mus musculus OX=10090 GN=Qrich1 PE=1 SV=1 - [QRIC1_MOUSE]	Qrich1	18.4543844	0	0	20.38230653	17.78656392	0	6.151461468	12.72295682	10.6546438	11.09457942	0.642834826	0.838405797	2.068281966	1.048432879
Q91VC3	Eukaryotic initiation factor 4A-III OS=Mus musculus OX=10090 GN=Eif4a3 PE=1 SV=3 - [IF4A3_MOUSE]	Eif4a3	440.5908811	0	0	433.9277408	477.9938259	0	146.863627	303.9738556	254.3752638	264.1695168	0.642834826	0.838405797	2.069769498	1.049470109
Q9D7X3	Dual specificity protein phosphatase 3 OS=Mus musculus OX=10090 GN=Dusp3 PE=1 SV=1 - [DUS3_MOUSE]	Dusp3	35.00776723	0	0	38.44597225	34.26417756	0	11.66925574	24.2367166	20.21174383	21.09349842	0.642834826	0.838405797	2.076971928	1.054481717
Q99LF4	RNA-splicing ligase RtcB homolog OS=Mus musculus OX=10090 GN=Rtcb PE=1 SV=1 - [RTCB_MOUSE]	Rtcb	57.1230167	0	0	47.33389222	71.34849179	0	19.04100557	39.56079467	32.97998907	36.30382355	0.642834826	0.838405797	2.077663101	1.054961736
Q8VIM9	Immunity-related GTPase family Q protein OS=Mus musculus OX=10090 GN=Irgq PE=1 SV=1 - [IRGQ_MOUSE]	Irgq	33.24749095	0	0	27.63949699	41.64407436	0	11.08249698	23.09452378	19.19544785	21.19079561	0.642834826	0.838405797	2.083873681	1.059267828
Q8QZZ7	EKC/KEOPS complex subunit Tprkb OS=Mus musculus OX=10090 GN=Tprkb PE=1 SV=1 - [TPRKB_MOUSE]	Tprkb	38.67721814	0	0	44.0275723	37.74656045	0	12.89240605	27.25804425	22.3303023	23.81414516	0.642834826	0.838405797	2.114271312	1.080160521
Q91W67	Ubiquitin-like protein 7 OS=Mus musculus OX=10090 GN=Ubl7 PE=1 SV=2 - [UBL7_MOUSE]	Ubl7	19.85361117	0	0	18.3351651	23.68234648	0	6.617870391	14.00583719	11.46248775	12.42057542	0.642834826	0.838405797	2.116366197	1.08158928
P53995	Anaphase-promoting complex subunit 1 OS=Mus musculus OX=10090 GN=Anapc1 PE=1 SV=2 - [APC1_MOUSE]	Anapc1	8.561870667	0	0	8.275300514	10.04736526	0	2.853956889	6.107555256	4.943198334	5.362996063	0.642834826	0.838405797	2.140030664	1.097631469
Q62203	Splicing factor 3A subunit 2 OS=Mus musculus OX=10090 GN=Sf3a2 PE=1 SV=2 - [SF3A2_MOUSE]	Sf3a2	12.55939407	0	0	6.722320716	20.16696215	0	4.186464691	8.963094287	7.251169549	10.26851451	0.642834826	0.838405797	2.140969756	1.098264416
Q9WVL0	Maleylacetoacetate isomerase OS=Mus musculus OX=10090 GN=Gstz1 PE=1 SV=1 - [MAAI_MOUSE]	Gstz1	11.99367944	0	0	11.18460427	14.55940129	0	3.997893148	8.581335186	6.924554056	7.620813533	0.642834826	0.838405797	2.146464367	1.101962223
P60670	Nuclear protein localization protein 4 homolog OS=Mus musculus OX=10090 GN=Nploc4 PE=1 SV=3 - [NPL4_MOUSE]	Nploc4	22.17091284	0	0	18.36324922	29.26767769	0	7.39030428	15.87697564	12.8003825	14.79139637	0.642834826	0.838405797	2.148352089	1.103230453
Q9DCC4	Pyrroline-5-carboxylate reductase 3 OS=Mus musculus OX=10090 GN=Pycr3 PE=1 SV=2 - [P5CR3_MOUSE]	Pycr3	87.21025654	0	0	102.5085805	86.15436427	0	29.07008551	62.88764826	50.35086509	55.07274576	0.642834826	0.838405797	2.16331143	1.11324137
P24638	Lysosomal acid phosphatase OS=Mus musculus OX=10090 GN=Acp2 PE=1 SV=2 - [PPAL_MOUSE]	Acp2	26.78083946	0	0	34.60539008	23.9785655	0	8.926946485	19.5279852	15.46192487	17.72678777	0.642834826	0.838405797	2.187532459	1.129304424
Q01149	Collagen alpha-2(I) chain OS=Mus musculus OX=10090 GN=Col1a2 PE=1 SV=2 - [CO1A2_MOUSE]	Col1a2	9.670153749	0	0	15.99155023	5.330516743	0	3.223384583	7.107355657	5.58306587	8.142498822	0.642834826	0.838405797	2.204935674	1.140736567
P62281	40S ribosomal protein S11 OS=Mus musculus OX=10090 GN=Rps11 PE=1 SV=3 - [RS11_MOUSE]	Rps11	69.5069344	0	0	59.79973392	93.56646786	0	23.16897813	51.12206726	40.12984729	47.38298641	0.642834826	0.838405797	2.206487786	1.141751761
Q9D0L7	Armadillo repeat-containing protein 10 OS=Mus musculus OX=10090 GN=Armc10 PE=1 SV=1 - [ARM10_MOUSE]	Armc10	32.12399208	0	0	41.69815477	29.68609329	0	10.70799736	23.79474935	18.54679548	21.46427212	0.642834826	0.838405797	2.22214748	1.151954569
Q9JJA4	Ribosome biogenesis protein WDR12 OS=Mus musculus OX=10090 GN=Wdr12 PE=1 SV=1 - [WDR12_MOUSE]	Wdr12	15.140505	0	0	23.19377927	0	11.27244694	5.046834999	11.48874207	8.741374636	11.59840234	0.642834826	0.838405797	2.276425141	1.186770018
Q9WUP7	Ubiquitin carboxyl-terminal hydrolase isozyme L5 OS=Mus musculus OX=10090 GN=Uchl5 PE=1 SV=2 - [UCHL5_MOUSE]	Uchl5	47.47828633	0	0	62.01232185	47.38701721	0	15.82609544	36.46644635	27.41160139	32.41644901	0.642834826	0.838405797	2.304197298	1.204264253
Q9QZD9	Eukaryotic translation initiation factor 3 subunit I OS=Mus musculus OX=10090 GN=Eif3i PE=1 SV=1 - [EIF3I_MOUSE]	Eif3i	45.76424083	0	0	68.69936742	38.44141709	0	15.25474694	35.71359484	26.42199676	34.43082241	0.642834826	0.838405797	2.341146331	1.227215111
P09528	Ferritin heavy chain OS=Mus musculus OX=10090 GN=Fth1 PE=1 SV=2 - [FRIH_MOUSE]	Fth1	20.37313959	0	0	0	30.13602072	18.57763541	6.791046532	16.23788538	11.76243763	15.20364295	0.642834826	0.838405797	2.391072614	1.257657943
Q9EPK7	Exportin-7 OS=Mus musculus OX=10090 GN=Xpo7 PE=1 SV=3 - [XPO7_MOUSE]	Xpo7	9.868127801	0	0	9.300061588	14.59246904	0	3.289375934	7.964176877	5.697366243	7.38738649	0.642834826	0.838405797	2.421181719	1.275711363
P62702	40S ribosomal protein S4, X isoform OS=Mus musculus OX=10090 GN=Rps4x PE=1 SV=2 - [RS4X_MOUSE]	Rps4x	51.78411458	0	0	32.09657956	93.78757178	0	17.26137153	41.96138378	29.89757249	47.66563675	0.642834826	0.838405797	2.430941465	1.281515155
Q8K3A9	7SK snRNA methylphosphate capping enzyme OS=Mus musculus OX=10090 GN=Mepce PE=1 SV=2 - [MEPCE_MOUSE]	Mepce	12.72789535	0	0	19.26012395	12.24809654	0	4.242631783	10.50274016	7.348453805	9.747963649	0.642834826	0.838405797	2.475524792	1.307734398
Q6PDQ2	Chromodomain-helicase-DNA-binding protein 4 OS=Mus musculus OX=10090 GN=Chd4 PE=1 SV=1 - [CHD4_MOUSE]	Chd4	5.690486869	0	0	5.476530088	9.427942051	0	1.896828956	4.96815738	3.285404126	4.73448571	0.642834826	0.838405797	2.619191026	1.389121284
Q8VD66	(Lyso)-N-acylphosphatidylethanolamine lipase OS=Mus musculus OX=10090 GN=Abhd4 PE=1 SV=1 - [ABHD4_MOUSE]	Abhd4	8.48155518	0	0	15.83959055	6.387325442	0	2.82718506	7.408971998	4.896828166	7.96906384	0.642834826	0.838405797	2.620617979	1.38990706
Q8R326	Paraspeckle component 1 OS=Mus musculus OX=10090 GN=Pspc1 PE=1 SV=1 - [PSPC1_MOUSE]	Pspc1	35.87464087	0	0	60.66838755	34.94346053	0	11.95821362	31.87061603	20.71223356	30.45069937	0.642834826	0.838405797	2.665165302	1.414225016
Q9D8B3	Charged multivesicular body protein 4b OS=Mus musculus OX=10090 GN=Chmp4b PE=1 SV=2 - [CHM4B_MOUSE]	Chmp4b	19.82516514	0	0	18.72951138	35.37136783	0	6.608388379	18.0336264	11.44606443	17.6959489	0.642834826	0.838405797	2.728899297	1.448319157
Q6ZPZ3	Zinc finger CCCH domain-containing protein 4 OS=Mus musculus OX=10090 GN=Zc3h4 PE=1 SV=2 - [ZC3H4_MOUSE]	Zc3h4	8.226259748	0	0	15.35922577	7.529148131	0	2.742086583	7.629457966	4.74943328	7.680104204	0.642834826	0.838405797	2.782354873	1.476306439
P41233	Phospholipid-transporting ATPase ABCA1 OS=Mus musculus OX=10090 GN=Abca1 PE=1 SV=4 - [ABCA1_MOUSE]	Abca1	14.81956111	0	0	11.60160885	30.5808694	0	4.939853705	14.06082608	8.556077599	15.43804409	0.642834826	0.838405797	2.846405364	1.509141135
Q9CRC8	Leucine-rich repeat-containing protein 40 OS=Mus musculus OX=10090 GN=Lrrc40 PE=1 SV=2 - [LRC40_MOUSE]	Lrrc40	6.541854953	0	0	5.637340132	13.32158861	0	2.180618318	6.319642914	3.776941718	6.686952489	0.642834826	0.838405797	2.898096775	1.535105771
Q9QZQ1	Afadin OS=Mus musculus OX=10090 GN=Afdn PE=1 SV=3 - [AFAD_MOUSE]	Afdn	11.43220806	0	0	37.63130742	0	9.226912516	3.810736021	15.61940665	6.600388402	19.61317951	0.642834826	0.838405797	4.098789987	2.035198071
P63321	Ras-related protein Ral-A OS=Mus musculus OX=10090 GN=Rala PE=1 SV=1 - [RALA_MOUSE]	Rala	16.94586477	0	0	4.847388697	4.847388697	14.54216609	5.648621589	8.078981161	9.783699584	5.597282338	0.653095115	0.838405797	1.430257105	0.516274511
P14115	60S ribosomal protein L27a OS=Mus musculus OX=10090 GN=Rpl27a PE=1 SV=5 - [RL27A_MOUSE]	Rpl27a	35.92785153	0	0	11.64073606	34.92220817	11.64073606	11.97595051	19.40122676	20.74295475	13.44156419	0.653095115	0.838405797	1.620015609	0.696007714
Q99LD8	N(G),N(G)-dimethylarginine dimethylaminohydrolase 2 OS=Mus musculus OX=10090 GN=Ddah2 PE=1 SV=1 - [DDAH2_MOUSE]	Ddah2	433.567041	0	0	177.0744792	198.6970986	53.78795199	144.522347	143.18651	250.3200478	78.17263614	0.657905019	0.838405797	0.990756882	-0.013397011
Q91WN1	DnaJ homolog subfamily C member 9 OS=Mus musculus OX=10090 GN=Dnajc9 PE=1 SV=2 - [DNJC9_MOUSE]	Dnajc9	88.18460636	0	0	22.33786544	40.90160414	26.11199219	29.39486879	29.78382059	50.91340622	9.811464798	0.657905019	0.838405797	1.013231963	0.018964493

Q9Z1R2	Large proline-rich protein BAG6 OS=Mus musculus OX=10090 GN=Bag6 PE=1 SV=1 - [BAG6_MOUSE]	Bag6	60.49951849	0	0	31.65073556	23.46633989	8.087717416	20.16650616	21.06826429	34.92941328	11.96315305	0.657905019	0.838405797	1.044715635	0.063110303
Q9CQT1	Methylthioribose-1-phosphate isomerase OS=Mus musculus OX=10090 GN=Mri1 PE=1 SV=1 - [MTNA_MOUSE]	Mri1	62.20881763	0	0	20.22094681	39.26409493	9.408058395	20.73627254	22.96436671	35.91627761	15.11590203	0.657905019	0.838405797	1.107449117	0.147240414
E9Q912	Rap1 GTPase-GDP dissociation stimulator 1 OS=Mus musculus OX=10090 GN=Rap1gds1 PE=2 SV=1 - [GDS1_MOUSE]	Rap1gds1	62.82734176	0	0	23.11769717	34.68680122	14.81864162	20.94244725	24.20771334	36.27338268	9.978829736	0.657905019	0.838405797	1.155916166	0.209036769
Q9WVJ2	26S proteasome non-ATPase regulatory subunit 13 OS=Mus musculus OX=10090 GN=Psmd13 PE=1 SV=1 - [PSD13_MOUSE]	Psmd13	173.1827502	0	0	78.21750136	93.98906764	48.19707175	57.72758338	73.46788025	99.98710742	23.2625428	0.657905019	0.838405797	1.272665093	0.347852818
P62192	26S proteasome regulatory subunit 4 OS=Mus musculus OX=10090 GN=Psmc1 PE=1 SV=1 - [PRS4_MOUSE]	Psmc1	123.8349215	0	0	59.21887398	69.39938355	42.28476231	41.27830716	56.96767328	71.49612526	13.69677333	0.657905019	0.838405797	1.38008744	0.464759677
P39749	Flap endonuclease 1 OS=Mus musculus OX=10090 GN=Fen1 PE=1 SV=1 - [FEN1_MOUSE]	Fen1	120.3601536	0	0	67.57717219	62.87549017	54.4235961	40.12005119	61.62541949	69.48996707	6.665294329	0.657905019	0.838405797	1.536025445	0.619202115
O08529	Calpain-2 catalytic subunit OS=Mus musculus OX=10090 GN=Capn2 PE=1 SV=4 - [CAN2_MOUSE]	Capn2	52.98129957	0	0	21.27861393	39.80169423	20.36010272	17.66043319	27.14680363	30.58876757	10.96907507	0.657905019	0.838405797	1.537153893	0.620261608
Q8VCF0	Mitochondrial antiviral-signaling protein OS=Mus musculus OX=10090 GN=Mavs PE=1 SV=1 - [MAVS_MOUSE]	Mavs	17.51109178	0	0	13.58766354	8.554604934	5.295259021	5.837030594	9.145842499	10.11003355	4.177698479	0.657905019	0.838405797	1.56686561	0.647881445
P54775	26S proteasome regulatory subunit 6B OS=Mus musculus OX=10090 GN=Psmc4 PE=1 SV=2 - [PRS6B_MOUSE]	Psmc4	72.2084045	0	0	46.14573539	39.56296871	27.90068572	24.06946817	37.86979661	41.68954178	9.239620293	0.657905019	0.838405797	1.573354108	0.653843408
P68433	Histone H3.1 OS=Mus musculus OX=10090 GN=H3c1 PE=1 SV=2 - [H31_MOUSE]	H3c1	1665.250743	0	0	929.7555083	958.7253914	770.416948	555.0835809	886.2992826	961.4329645	101.3969927	0.657905019	0.838405797	1.596695188	0.675088926
Q6A028	Switch-associated protein 70 OS=Mus musculus OX=10090 GN=Swap70 PE=1 SV=2 - [SWP70_MOUSE]	Swap70	37.7660978	0	0	26.22021714	21.18185474	13.58684044	12.58869927	20.32963744	21.80426673	6.359658596	0.657905019	0.838405797	1.614911677	0.691455263
Q9D2N4	Dystrobrevin alpha OS=Mus musculus OX=10090 GN=Dtna PE=1 SV=2 - [DTNA_MOUSE]	Dtna	6.063526501	0	0	3.780624321	3.29391345	2.837190821	2.0211755	3.30390953	3.500778657	0.471796178	0.657905019	0.838405797	1.634647526	0.708979585
Q9Z0X1	Apoptosis-inducing factor 1, mitochondrial OS=Mus musculus OX=10090 GN=Aifm1 PE=1 SV=1 - [AIFM1_MOUSE]	Aifm1	55.70123857	0	0	41.76511109	44.32555473	6.314803797	18.56707952	30.80182321	32.15912508	21.244989	0.657905019	0.838405797	1.658948203	0.730268842
P48193	Protein 4.1 OS=Mus musculus OX=10090 GN=Epb41 PE=1 SV=2 - [EPB41_MOUSE]	Epb41	25.96140406	0	0	10.46004684	23.67940498	9.419924314	8.653801355	14.51979204	14.98882362	7.949487122	0.657905019	0.838405797	1.677851322	0.746614881
P51410	60S ribosomal protein L9 OS=Mus musculus OX=10090 GN=Rpl9 PE=1 SV=2 - [RL9_MOUSE]	Rpl9	44.65699696	0	0	24.80926487	27.72771287	25.10746218	14.88566565	25.88147998	25.78272922	1.605821391	0.657905019	0.838405797	1.738684757	0.79799638
Q60631	Growth factor receptor-bound protein 2 OS=Mus musculus OX=10090 GN=Grb2 PE=1 SV=1 - [GRB2_MOUSE]	Grb2	111.8512129	0	0	51.74151624	104.8446356	44.77073867	37.28373763	67.1189635	64.57732787	32.85677535	0.657905019	0.838405797	1.800220894	0.848173942
Q9CWZ3	RNA-binding protein 8A OS=Mus musculus OX=10090 GN=Rbm8a PE=1 SV=4 - [RBM8A_MOUSE]	Rbm8a	89.61011679	0	0	63.06025849	61.77332146	38.06352771	29.87003893	54.29903589	51.73642505	14.0750789	0.657905019	0.838405797	1.817842823	0.862227464
Q9QYF9	Protein NDRG3 OS=Mus musculus OX=10090 GN=Ndrg3 PE=1 SV=1 - [NDRG3_MOUSE]	Ndrg3	55.28760005	0	0	39.75148481	36.33246984	26.02751258	18.42920002	34.03715575	31.92031077	7.14410271	0.657905019	0.838405797	1.846914446	0.885117039
Q9JJI8	60S ribosomal protein L38 OS=Mus musculus OX=10090 GN=Rpl38 PE=1 SV=3 - [RL38_MOUSE]	Rpl38	117.5454758	0	0	98.83465895	96.02326032	22.51230178	39.18182526	72.45674035	67.86491209	43.27598871	0.657905019	0.838405797	1.849243619	0.886935298
P0C0S6	Histone H2A.Z OS=Mus musculus OX=10090 GN=H2az1 PE=1 SV=2 - [H2AZ_MOUSE]	H2az1	1951.408766	0	0	1067.200002	1251.669524	1298.141071	650.4695887	1205.670199	1126.646376	122.1490799	0.657905019	0.838405797	1.853538151	0.890281811
P62754	40S ribosomal protein S6 OS=Mus musculus OX=10090 GN=Rps6 PE=1 SV=1 - [RS6_MOUSE]	Rps6	127.4372294	0	0	70.23513477	83.89183583	82.40531167	42.47907648	78.84409409	73.57591872	7.492534545	0.657905019	0.838405797	1.856068931	0.892250291
Q9R1Q8	Transgelin-3 OS=Mus musculus OX=10090 GN=Tagln3 PE=1 SV=1 - [TAGL3_MOUSE]	Tagln3	497.4581336	0	0	462.5685938	285.2933814	184.1599478	165.8193779	310.6739743	287.2075873	140.9289694	0.657905019	0.838405797	1.873568568	0.905788778
Q91Z67	SLIT-ROBO Rho GTPase-activating protein 2 OS=Mus musculus OX=10090 GN=Srgap2 PE=1 SV=2 - [SRGP2_MOUSE]	Srgap2	79.75839273	0	0	59.7056581	37.53973128	55.43780702	26.58613091	50.8943988	46.04852951	11.76069712	0.657905019	0.838405797	1.91432138	0.936833052
P34022	Ran-specific GTPase-activating protein OS=Mus musculus OX=10090 GN=Ranbp1 PE=1 SV=2 - [RANG_MOUSE]	Ranbp1	285.4092647	0	0	236.8518825	248.3955156	61.71801073	95.13642156	182.3218029	164.7811158	104.6053053	0.657905019	0.838405797	1.916424856	0.93841743
O54984	ATPase GET3 OS=Mus musculus OX=10090 GN=Get3 PE=1 SV=2 - [GET3_MOUSE]	Get3	47.1061869	0	0	22.91225092	46.00131108	21.68188775	15.7020623	30.19848325	27.19676969	13.69946984	0.657905019	0.838405797	1.923217643	0.943522036
Q9Z1F9	SUMO-activating enzyme subunit 2 OS=Mus musculus OX=10090 GN=Uba2 PE=1 SV=1 - [SAE2_MOUSE]	Uba2	60.65061006	0	0	39.52300471	57.70226119	19.52464493	20.21687002	38.91663694	35.01664605	19.09602988	0.657905019	0.838405797	1.924958557	0.944827386
Q8BG32	26S proteasome non-ATPase regulatory subunit 11 OS=Mus musculus OX=10090 GN=Psmd11 PE=1 SV=3 - [PSD11_MOUSE]	Psmd11	90.96035651	0	0	75.12435126	69.54208869	32.01537084	30.32011884	58.89393693	52.51598632	23.44426156	0.657905019	0.838405797	1.942404555	0.95784371
Q3UKJ7	WD40 repeat-containing protein SMU1 OS=Mus musculus OX=10090 GN=Smu1 PE=2 SV=2 - [SMU1_MOUSE]	Smu1	50.19726692	0	0	47.8640059	36.26788174	13.88106535	16.73242231	32.67098433	28.98140557	17.27464371	0.657905019	0.838405797	1.952555567	0.965363606
A2AJI0	MAP7 domain-containing protein 1 OS=Mus musculus OX=10090 GN=Map7d1 PE=1 SV=1 - [MA7D1_MOUSE]	Map7d1	27.91017371	0	0	19.18674798	17.36148503	18.02617993	9.303391238	18.19147098	16.11394631	0.92378951	0.657905019	0.838405797	1.955359128	0.967433602
Q8JZK9	Hydroxymethylglutaryl-CoA synthase, cytoplasmic OS=Mus musculus OX=10090 GN=Hmgcs1 PE=1 SV=1 - [HMCS1_MOUSE]	Hmgcs1	151.2959589	0	0	139.4415478	119.3894966	38.48209824	50.43198631	99.10438089	87.35076261	53.44920069	0.657905019	0.838405797	1.965109609	0.974609785
O35841	Apoptosis inhibitor 5 OS=Mus musculus OX=10090 GN=Api5 PE=1 SV=2 - [API5_MOUSE]	Api5	103.4423687	0	0	78.73824279	98.627636	26.36898636	34.48078958	67.91162172	59.72247944	37.32612898	0.657905019	0.838405797	1.969549495	0.977865673
Q8QZT1	Acetyl-CoA acetyltransferase, mitochondrial OS=Mus musculus OX=10090 GN=Acat1 PE=1 SV=1 - [THIL_MOUSE]	Acat1	59.65087134	0	0	44.86352381	53.98888865	18.95915457	19.88362378	39.27052234	34.43944663	18.17228154	0.657905019	0.838405797	1.975018376	0.981866076
O70133	ATP-dependent RNA helicase A OS=Mus musculus OX=10090 GN=Dhx9 PE=1 SV=2 - [DHX9_MOUSE]	Dhx9	45.22089841	0	0	41.10113515	37.9930839	10.39459437	15.0736328	29.82960448	26.10829787	16.90280181	0.657905019	0.838405797	1.97892604	0.984717695
Q99J09	Methylosome protein 50 OS=Mus musculus OX=10090 GN=Wdr77 PE=1 SV=1 - [MEP50_MOUSE]	Wdr77	116.1917864	0	0	102.2226088	84.7851447	43.66076646	38.73059546	76.88950665	67.08335915	30.06872411	0.657905019	0.838405797	1.985239466	0.98931304
P62311	U6 snRNA-associated Sm-like protein LSm3 OS=Mus musculus OX=10090 GN=Lsm3 PE=1 SV=2 - [LSM3_MOUSE]	Lsm3	124.9336654	0	0	100.2819115	85.38810795	66.52058067	41.64455514	84.06353336	72.13048536	16.91959626	0.657905019	0.838405797	2.018596023	1.013352216
Q9R059	Four and a half LIM domains protein 3 OS=Mus musculus OX=10090 GN=Fhl3 PE=1 SV=2 - [FHL3_MOUSE]	Fhl3	89.65716108	0	0	76.35329372	67.77278804	41.17825259	29.88572036	61.76811145	51.76358608	18.34020135	0.657905019	0.838405797	2.066810193	1.047405904
P54071	Isocitrate dehydrogenase [NADP], mitochondrial OS=Mus musculus OX=10090 GN=Idh2 PE=1 SV=3 - [IDHP_MOUSE]	Idh2	331.2065535	0	0	296.7031949	288.8082406	100.9727152	110.4021845	228.8280502	191.2221928	110.7963113	0.657905019	0.838405797	2.072676834	1.051495193
Q9DCT8	Cysteine-rich protein 2 OS=Mus musculus OX=10090 GN=Crip2 PE=1 SV=1 - [CRIP2_MOUSE]	Crip2	196.1858615	0	0	180.1583837	152.7849644	74.16146187	65.39528716	135.7016033	113.2679599	55.02470155	0.657905019	0.838405797	2.075097598	1.053179192
Q922P9	Putative oxidoreductase GLYR1 OS=Mus musculus OX=10090 GN=Glyr1 PE=1 SV=1 - [GLYR1_MOUSE]	Glyr1	61.78644585	0	0	59.25826542	47.25510676	22.12276565	20.59548195	42.87871261	35.67242115	18.95061886	0.657905019	0.838405797	2.081947522	1.057933704
P14685	26S proteasome non-ATPase regulatory subunit 3 OS=Mus musculus OX=10090 GN=Psmd3 PE=1 SV=3 - [PSMD3_MOUSE]	Psmd3	77.34075844	0	0	77.22255971	51.52010879	37.45568083	25.78025281	55.39944978	44.6527077	20.16527037	0.657905019	0.838405797	2.14891026	1.103605236
O08547	Vesicle-trafficking protein SEC22b OS=Mus musculus OX=10090 GN=Sec22b PE=1 SV=3 - [SC22B_MOUSE]	Sec22b	83.12561932	0	0	70.18488013	62.94421483	48.26988545	27.70853977	60.4663268	47.99259869	11.16564798	0.657905019	0.838405797	2.182227115	1.125801258
P58404	Striatin-4 OS=Mus musculus OX=10090 GN=Strn4 PE=1 SV=2 - [STRN4_MOUSE]	Strn4	21.09330202	0	0	18.95182632	16.39590193	10.85337344	7.031100672	15.40036723	12.1782236	4.139994175	0.657905019	0.838405797	2.190320967	1.131142296
Q91WT9	Cystathionine beta-synthase OS=Mus musculus OX=10090 GN=Cbs PE=1 SV=3 - [CBS_MOUSE]	Cbs	91.84688642	0	0	77.04152206	81.50666455	43.28564233	30.61562881	67.27794298	53.0278246	20.89754153	0.657905019	0.838405797	2.197503223	1.135865281
Q8CIG8	Protein arginine N-methyltransferase 5 OS=Mus musculus OX=10090 GN=Prmt5 PE=1 SV=3 - [ANM5_MOUSE]	Prmt5	56.19705735	0	0	48.78744075	50.73665413	24.26044432	18.73235245	41.26151306	32.44538619	14.75557902	0.657905019	0.838405797	2.202687205	1.139264638
Q9QX47	Protein SON OS=Mus musculus OX=10090 GN=Son PE=1 SV=2 - [SON_MOUSE]	Son	53.13205739	0	0	42.45335674	49.18931402	25.54046901	17.7106858	39.06104659	30.67580764	12.18391536	0.657905019	0.838405797	2.205507287	1.141110527
P63001	Ras-related C3 botulinum toxin substrate 1 OS=Mus musculus OX=10090 GN=Rac1 PE=1 SV=1 - [RAC1_MOUSE]	Rac1	891.5125036	0	0	768.436112	819.8161397	391.2100288	297.1708345	659.8207602	514.714984	234.0379682	0.657905019	0.838405797	2.22034158	1.150781639
Q91V41	Ras-related protein Rab-14 OS=Mus musculus OX=10090 GN=Rab14 PE=1 SV=3 - [RAB14_MOUSE]	Rab14	237.9669763	0	0	206.4213802	229.0307666	96.65245121	79.32232544	177.3681993	137.3902978	70.81009876	0.657905019	0.838405797	2.236043867	1.160948492
Q8VDJ3	Vigilin OS=Mus musculus OX=10090 GN=Hdlbp PE=1 SV=1 - [VIGLN_MOUSE]	Hdlbp	62.5925496	0	0	58.77735046	59.52570409	21.72028062	20.8641832	46.67444506	36.13782536	21.61417938	0.657905019	0.838405797	2.237060737	1.161604427
Q9D1J3	SAP domain-containing ribonucleoprotein OS=Mus musculus OX=10090 GN=Sarnp PE=1 SV=3 - [SARNP_MOUSE]	Sarnp	45.98842634	0	0	40.21565168	41.71994038	21.20826237	15.32947545	34.38128481	26.55143033	11.43293975	0.657905019	0.838405797	2.242822002	1.165315128
Q9CX86	Heterogeneous nuclear ribonucleoprotein A0 OS=Mus musculus OX=10090 GN=Hnrnpa0 PE=1 SV=1 - [ROA0_MOUSE]	Hnrnpa0	1135.587852	0	0	1019.311916	926.0376965	615.2339468	378.5292842	853.5278532	655.6319523	211.5727046	0.657905019	0.838405797	2.254852897	1.173033318
P20065	Thymosin beta-4 OS=Mus musculus OX=10090 GN=Tmsb4x PE=1 SV=1 - [TYB4_MOUSE]	Tmsb4x	376.778058	0	0	290.0420217	328.560754	235.4785174	125.592686	284.6937644	217.5329132	46.77102292	0.657905019	0.838405797	2.2668021	1.180658444
Q9QYR9	Acyl-coenzyme A thioesterase 2, mitochondrial OS=Mus musculus OX=10090 GN=Acot2 PE=1 SV=2 - [ACOT2_MOUSE]	Acot2	759.3812577	0	0	622.8214117	665.9476193	440.5184837	253.1270859	576.4291716	438.4289736	119.6609951	0.657905019	0.838405797	2.277232282	1.187281457
Q8CB77	Elongin-A OS=Mus musculus OX=10090 GN=Eloa PE=1 SV=3 - [ELOA1_MOUSE]	Eloa	28.20585351	0	0	24.69461154	25.42246901	15.22889382	9.401951168	21.78199146	16.28465711	5.686805836	0.657905019	0.838405797	2.316752243	1.212103768
Q8BUK6	Protein Hook homolog 3 OS=Mus musculus OX=10090 GN=Hook3 PE=1 SV=2 - [HOOK3_MOUSE]	Hook3	13.04422782	0	0	12.4697436	12.86818223	5.299051755	4.34807594	10.21232586	7.531088443	4.259681347	0.657905019	0.838405797	2.348699977	1.231862435
P97427	Dihydropyrimidinase-related protein 1 OS=Mus musculus OX=10090 GN=Crmp1 PE=1 SV=1 - [DPYL1_MOUSE]	Crmp1	496.7478906	0	0	470.2426003	473.7055222	238.094844	165.5826302	394.0143222	286.7975284	135.0413296	0.657905019	0.838405797	2.379563132	1.250696731
P51660	Peroxisomal multifunctional enzyme type 2 OS=Mus musculus OX=10090 GN=Hsd17b4 PE=1 SV=3 - [DHB4_MOUSE]	Hsd17b4	7.109507489	0	0	6.676507182	6.687139981	4.018805092	2.36983583	5.794150751	4.104676063	1.537503633	0.657905019	0.838405797	2.444958709	1.289810101
Q9Z0G0	PDZ domain-containing protein GIPC1 OS=Mus musculus OX=10090 GN=Gipc1 PE=1 SV=1 - [GIPC1_MOUSE]	Gipc1	48.11394763	0	0	41.67454049	44.74791193	34.94444621	16.03798254	40.45563287	27.77860061	5.01410878	0.657905019	0.838405797	2.522488896	1.334847919
O08539	Myc box-dependent-interacting protein 1 OS=Mus musculus OX=10090 GN=Bin1 PE=1 SV=1 - [BIN1_MOUSE]	Bin1	84.72482787	0	0	67.63804115	81.87514528	68.04301785	28.24160929	72.51873476	48.91590218	8.10541886	0.657905019	0.838405797	2.567797536	1.360531454
P51432	1-phosphatidylinositol 4,5-bisphosphate phosphodiesterase beta-3 OS=Mus musculus OX=10090 GN=Plcb3 PE=1 SV=2 - [PLCB3_MOUSE]	Plcb3	22.11930127	0	0	19.37427625	19.90417474	17.52028199	7.373100422	18.932911	12.77058454	1.251734245	0.657905019	0.838405797	2.567835769	1.360552935
P32037	Solute carrier family 2, facilitated glucose transporter member 3 OS=Mus musculus OX=10090 GN=Slc2a3 PE=1 SV=1 - [GTR3_MOUSE]	Slc2a3	1228.906371	11.76321884	0	5.499391048	4.882545572	11.61562757	413.5565301	7.332521397	706.1381707	3.722079185	0.7	0.838405797	0.017730397	-5.817631362
Q9DBR1	5'-3' exoribonuclease 2 OS=Mus musculus OX=10090 GN=Xrn2 PE=1 SV=1 - [XRN2_MOUSE]	Xrn2	473.6747472	6.108096462	0	17.09739103	28.54673425	9.623261288	159.9276146	18.42246219	271.7301504	9.531071159	0.7	0.838405797	0.115192503	-3.117881271
Q9CPT0	Apoptosis facilitator Bcl-2-like protein 14 OS=Mus musculus OX=10090 GN=Bcl2l14 PE=1 SV=1 - [B2L14_MOUSE]	Bcl2l14	0	656.4777001	875.3883885	79.20928703	306.941676	70.56504869	510.6220296	152.2386706	455.5563798	134.0464309	0.7	0.838405797	0.298143562	-1.745920912
Q8QZY6	Tetraspanin-14 OS=Mus musculus OX=10090 GN=Tspan14 PE=1 SV=1 - [TSN14_MOUSE]	Tspan14	25967.97586	37.53430243	52.17014291	11073.87001	0	44.01043905	8685.893435	3705.960151	14966.7242	6380.835058	0.7	0.838405797	0.426664243	-1.228826884
P12787	Cytochrome c oxidase subunit 5A, mitochondrial OS=Mus musculus OX=10090 GN=Cox5a PE=1 SV=2 - [COX5A_MOUSE]	Cox5a	0	105.8623296	120.0071652	17.66178594	26.62626092	85.86214039	75.2898316	43.38339575	65.58535012	37.05972561	0.7	0.838405797	0.576218525	-0.795312051
Q99J85	Neuronal pentraxin receptor OS=Mus musculus OX=10090 GN=Nptxr PE=1 SV=1 - [NPTXR_MOUSE]	Nptxr	52.62491345	107.7491037	105.1239473	0	52.13640652	108.7649553	88.49932149	53.63378728	31.09586348	54.39793644	0.7	0.838405797	0.606036141	-0.722524263
Q9Z0M3	Cadherin-20 OS=Mus musculus OX=10090 GN=Cdh20 PE=2 SV=1 - [CAD20_MOUSE]	Cdh20	50.43084451	36.8292147	62.12153938	0	32.79022418	64.19807918	49.7938662	32.32943446	12.65818818	32.10152003	0.7	0.838405797	0.6492654	-0.623119766
Q8R180	ERO1-like protein alpha OS=Mus musculus OX=10090 GN=Ero1a PE=1 SV=2 - [ERO1A_MOUSE]	Ero1a	0	27.05150977	32.14129082	7.689591855	11.67486609	19.85070065	19.73093353	13.07171953	17.27595934	6.199721075	0.7	0.838405797	0.662498787	-0.594010282
P55002	Microfibrillar-associated protein 2 OS=Mus musculus OX=10090 GN=Mfap2 PE=2 SV=1 - [MFAP2_MOUSE]	Mfap2	260.8043739	213.3712655	112.8173572	127.015039	116.7817257	150.6987568	195.6643322	131.4985072	75.56580504	17.39733787	0.7	0.838405797	0.672061718	-0.573334368
Q61301	Catenin alpha-2 OS=Mus musculus OX=10090 GN=Ctnna2 PE=1 SV=3 - [CTNA2_MOUSE]	Ctnna2	78.26866963	42.08280329	46.7216143	44.68800581	71.50245015	0	55.69102907	38.73015199	19.68989669	36.12162941	0.7	0.838405797	0.695446872	-0.523987788
Q9DAS9	Guanine nucleotide-binding protein G(I)/G(S)/G(O) subunit gamma-12 OS=Mus musculus OX=10090 GN=Gng12 PE=1 SV=3 - [GBG12_MOUSE]	Gng12	99.8789318	83.6191052	16.59018338	36.38848876	49.93297455	57.96624088	66.69607346	48.0959014	44.14799719	10.9055479	0.7	0.838405797	0.721120433	-0.471687873
O09114	Prostaglandin-H2 D-isomerase OS=Mus musculus OX=10090 GN=Ptgds PE=1 SV=1 - [PTGDS_MOUSE]	Ptgds	66.18635625	52.60109827	47.46134574	88.3375781	0	32.86067384	55.41626675	40.39941731	9.674729189	44.64870005	0.7	0.838405797	0.729017303	-0.455975039
P62960	Y-box-binding protein 1 OS=Mus musculus OX=10090 GN=Ybx1 PE=1 SV=3 - [YBOX1_MOUSE]	Ybx1	174.1688826	142.880095	42.22262272	88.4831691	134.9032635	47.39861252	119.7572001	90.2616817	68.94530495	43.77942799	0.7	0.838405797	0.753705678	-0.407926834
P54227	Stathmin OS=Mus musculus OX=10090 GN=Stmn1 PE=1 SV=2 - [STMN1_MOUSE]	Stmn1	497.2452605	355.1790338	285.6091266	208.9186236	288.6553527	362.7605663	379.3444736	286.7781808	107.8676951	76.9381483	0.7	0.838405797	0.755983547	-0.403573258
P97326	Cadherin-6 OS=Mus musculus OX=10090 GN=Cdh6 PE=1 SV=2 - [CADH6_MOUSE]	Cdh6	13.17389616	25.55772994	40.84945286	0	25.29671341	35.54113354	26.52702632	20.27928232	13.86321612	18.29409753	0.7	0.838405797	0.764476277	-0.387456362
P62627	Dynein light chain roadblock-type 1 OS=Mus musculus OX=10090 GN=Dynlrb1 PE=1 SV=3 - [DLRB1_MOUSE]	Dynlrb1	123.6523568	102.3070793	100.7836229	0	128.2675169	127.5884463	108.914353	85.28532104	12.78619558	73.86003502	0.7	0.838405797	0.783049421	-0.352824731
Q6GQT1	Alpha-2-macroglobulin-P OS=Mus musculus OX=10090 GN=A2m PE=2 SV=2 - [A2MG_MOUSE]	A2m	4200.667266	5771.110786	2920.534257	3130.512133	3977.547556	3003.231322	4297.437436	3370.430337	1427.749973	529.6164553	0.7	0.838405797	0.784288401	-0.35054383
Q9Z127	Large neutral amino acids transporter small subunit 1 OS=Mus musculus OX=10090 GN=Slc7a5 PE=1 SV=2 - [LAT1_MOUSE]	Slc7a5	35.19271925	68.77501609	35.39453535	0	34.63242552	77.06580293	46.45409023	37.23274282	19.3307522	38.59864937	0.7	0.838405797	0.801495469	-0.319233731
O35598	Disintegrin and metalloproteinase domain-containing protein 10 OS=Mus musculus OX=10090 GN=Adam10 PE=1 SV=2 - [ADA10_MOUSE]	Adam10	82.69817935	99.14751203	115.110285	39.02069057	84.63202234	114.6171594	98.98532545	79.42329076	16.20666147	38.06645094	0.7	0.838405797	0.802374396	-0.317652524
O88207	Collagen alpha-1(V) chain OS=Mus musculus OX=10090 GN=Col5a1 PE=1 SV=2 - [CO5A1_MOUSE]	Col5a1	18.75700906	38.10654541	49.26418071	13.7985312	23.80595273	47.76308613	35.37591172	28.45585669	15.43580742	17.4531932	0.7	0.838405797	0.804385111	-0.314041717
P07356	Annexin A2 OS=Mus musculus OX=10090 GN=Anxa2 PE=1 SV=2 - [ANXA2_MOUSE]	Anxa2	111.1691556	101.4116565	102.5774081	50.97254187	119.0628014	85.27473313	105.0527401	85.10335879	5.328944378	34.04545325	0.7	0.838405797	0.810101276	-0.303825815
P62878	E3 ubiquitin-protein ligase RBX1 OS=Mus musculus OX=10090 GN=Rbx1 PE=1 SV=1 - [RBX1_MOUSE]	Rbx1	147.1345384	115.2259787	90.51321541	97.26826713	101.1476665	93.2165041	117.6245775	97.21081256	28.38676649	3.965893324	0.7	0.838405797	0.826449834	-0.275000845
P10852	4F2 cell-surface antigen heavy chain OS=Mus musculus OX=10090 GN=Slc3a2 PE=1 SV=1 - [4F2_MOUSE]	Slc3a2	247.0128531	244.3914467	233.192091	120.695949	205.6698734	274.4943651	241.5321303	200.2867291	7.340649447	77.04039125	0.7	0.838405797	0.829234309	-0.270148287
P48036	Annexin A5 OS=Mus musculus OX=10090 GN=Anxa5 PE=1 SV=1 - [ANXA5_MOUSE]	Anxa5	104.8673393	82.2859606	69.35912627	40.94021071	102.1041749	69.78031595	85.50414207	70.9415672	17.97152796	30.59851318	0.7	0.838405797	0.829685738	-0.269363108
P28667	MARCKS-related protein OS=Mus musculus OX=10090 GN=Marcksl1 PE=1 SV=2 - [MRP_MOUSE]	Marcksl1	659.1813211	574.5678505	387.2324537	450.5451878	426.279222	476.5177636	540.3272084	451.1140578	139.1702655	25.12410146	0.7	0.838405797	0.834890508	-0.260341087
P07214	SPARC OS=Mus musculus OX=10090 GN=Sparc PE=1 SV=1 - [SPRC_MOUSE]	Sparc	2922.049907	6111.780021	5862.602675	3582.767083	3471.952622	5398.343022	4965.477534	4151.020909	1774.040498	1081.632702	0.7	0.838405797	0.835976174	-0.258466271
P50518	V-type proton ATPase subunit E 1 OS=Mus musculus OX=10090 GN=Atp6v1e1 PE=1 SV=2 - [VATE1_MOUSE]	Atp6v1e1	36.62437051	33.98155295	32.95972978	17.68746552	32.7948712	36.7066538	34.52188441	29.06299684	1.891128364	10.04378143	0.7	0.838405797	0.84187168	-0.248327743
P50516	V-type proton ATPase catalytic subunit A OS=Mus musculus OX=10090 GN=Atp6v1a PE=1 SV=2 - [VATA_MOUSE]	Atp6v1a	143.7633134	217.9488215	197.7436543	148.1240909	178.0834757	146.0164589	186.4852631	157.4080085	38.3527826	17.93646387	0.7	0.838405797	0.844077467	-0.244552684
P70232	Neural cell adhesion molecule L1-like protein OS=Mus musculus OX=10090 GN=Chl1 PE=1 SV=2 - [NCHL1_MOUSE]	Chl1	86.12490937	52.21031493	57.7182488	60.77767471	50.56190996	54.39149861	65.3511577	55.24369443	18.20016216	5.160924399	0.7	0.838405797	0.845336125	-0.242402991
O54962	Barrier-to-autointegration factor OS=Mus musculus OX=10090 GN=Banf1 PE=1 SV=1 - [BAF_MOUSE]	Banf1	86.85531045	50.48432457	64.34879631	58.7147478	56.25890424	55.77629244	67.22947711	56.91664816	18.35581366	1.575785606	0.7	0.838405797	0.846602571	-0.240243225
P41731	CD63 antigen OS=Mus musculus OX=10090 GN=Cd63 PE=1 SV=2 - [CD63_MOUSE]	Cd63	102.0273633	81.28985669	114.0987271	53.78352366	93.46778925	108.6059395	99.13864903	85.28575081	16.59409526	28.31225239	0.7	0.838405797	0.86026743	-0.217142877
Q640R3	Hepatocyte cell adhesion molecule OS=Mus musculus OX=10090 GN=Hepacam PE=1 SV=2 - [HECAM_MOUSE]	Hepacam	84.26531006	113.4156557	115.0177039	55.30488814	75.71981925	138.7410536	104.2328899	89.92192033	17.31097403	43.49336942	0.7	0.838405797	0.862701979	-0.21306583
P10853	Histone H2B type 1-F/J/L OS=Mus musculus OX=10090 GN=H2bc7 PE=1 SV=2 - [H2B1F_MOUSE]	H2bc7	2670.407546	1395.488981	1181.933007	1396.671815	1593.246521	1620.68876	1749.276511	1536.869032	804.8374527	122.1872083	0.7	0.838405797	0.878574097	-0.186764129
P14069	Protein S100-A6 OS=Mus musculus OX=10090 GN=S100a6 PE=1 SV=3 - [S10A6_MOUSE]	S100a6	104.6004163	154.5235046	96.41817895	93.09502829	115.0842932	104.4609788	118.5140333	104.2134335	31.45232535	10.99672234	0.7	0.838405797	0.879334123	-0.185516641
P39061	Collagen alpha-1(XVIII) chain OS=Mus musculus OX=10090 GN=Col18a1 PE=1 SV=4 - [COIA1_MOUSE]	Col18a1	78.62666938	93.2869157	108.6935279	56.45053867	92.64824461	100.0576052	93.53570431	83.0521295	15.03497311	23.33362663	0.7	0.838405797	0.887919005	-0.171500013
Q3UZ39	Leucine-rich repeat flightless-interacting protein 1 OS=Mus musculus OX=10090 GN=Lrrfip1 PE=1 SV=2 - [LRRF1_MOUSE]	Lrrfip1	27.38634319	17.42904813	21.98715258	20.91799343	18.91713668	19.48379092	22.26751463	19.77297368	4.984564516	1.031298664	0.7	0.838405797	0.887973984	-0.171410686

P24399	Zinc finger protein 239 OS=Mus musculus OX=10090 GN=Znf239 PE=2 SV=2 - [ZN239_MOUSE]	Znf239	0	1352.452323	1069.23503	636.5346281	557.400661	977.3001209	807.2291179	723.7451367	713.2791496	223.1213655	0.7	0.838405797	0.896579572	-0.157496467
P30412	Peptidyl-prolyl cis-trans isomerase C OS=Mus musculus OX=10090 GN=Ppic PE=1 SV=1 - [PPIC_MOUSE]	Ppic	121.0566171	86.49166187	109.3586867	116.4154979	81.07655172	88.44471791	105.6356552	95.31225585	17.58066423	18.64356693	0.7	0.838405797	0.902273533	-0.148363228
Q923X4	Glutaredoxin-2, mitochondrial OS=Mus musculus OX=10090 GN=Glrx2 PE=1 SV=1 - [GLRX2_MOUSE]	Glrx2	22.24314268	31.26257651	11.91388675	19.84231234	18.81339536	20.52021002	21.80653531	19.72530591	9.681731167	0.859402089	0.7	0.838405797	0.904559373	-0.144712894
Q3UQ28	Peroxidasin homolog OS=Mus musculus OX=10090 GN=Pxdn PE=1 SV=2 - [PXDN_MOUSE]	Pxdn	65.88890004	51.5194541	56.01533666	68.87450365	47.52370847	41.90191164	57.80789693	52.76670792	7.35052368	14.23014274	0.7	0.838405797	0.912794112	-0.13163861
Q9CR00	26S proteasome non-ATPase regulatory subunit 9 OS=Mus musculus OX=10090 GN=Psmd9 PE=1 SV=1 - [PSMD9_MOUSE]	Psmd9	94.69018273	76.71558881	65.73941859	88.87684105	70.29775176	57.39143007	79.04839671	72.1886743	14.6156827	15.82764882	0.7	0.838405797	0.913221233	-0.130963692
Q61739	Integrin alpha-6 OS=Mus musculus OX=10090 GN=Itga6 PE=1 SV=3 - [ITA6_MOUSE]	Itga6	29.62793393	29.87406582	22.5008527	10.12691906	19.91924221	44.89542889	27.33428415	24.98053005	4.187683119	17.92832382	0.7	0.838405797	0.913890041	-0.129907505
Q60865	Caprin-1 OS=Mus musculus OX=10090 GN=Caprin1 PE=1 SV=2 - [CAPR1_MOUSE]	Caprin1	171.8498374	125.5835222	95.40942848	118.6972808	157.7281701	87.39379498	130.947596	121.273082	38.50148041	35.23786521	0.7	0.838405797	0.926119193	-0.110730212
Q8QZY9	Splicing factor 3B subunit 4 OS=Mus musculus OX=10090 GN=Sf3b4 PE=1 SV=1 - [SF3B4_MOUSE]	Sf3b4	112.737473	146.2918879	158.426072	128.4484713	147.7023778	110.7113978	139.151811	128.9540823	23.66638006	18.50067246	0.7	0.838405797	0.926715085	-0.10980224
Q8CGP6	Histone H2A type 1-H OS=Mus musculus OX=10090 GN=H2ac12 PE=1 SV=3 - [H2A1H_MOUSE]	H2ac12	2651.975613	1386.818591	1270.827997	1451.128973	1736.795512	1764.568113	1769.874067	1650.830866	766.1206224	173.5034982	0.7	0.838405797	0.932739169	-0.100454393
Q3U1J4	DNA damage-binding protein 1 OS=Mus musculus OX=10090 GN=Ddb1 PE=1 SV=2 - [DDB1_MOUSE]	Ddb1	371.1510926	342.7876805	416.0149471	365.6069557	378.6831436	312.6226595	376.6512401	352.3042529	36.92217304	34.98168543	0.7	0.838405797	0.935359334	-0.096407388
Q60902	Epidermal growth factor receptor substrate 15-like 1 OS=Mus musculus OX=10090 GN=Eps15l1 PE=1 SV=3 - [EP15R_MOUSE]	Eps15l1	34.5491899	34.65514554	0	25.20859265	30.99274421	8.905619084	23.06811181	21.70231865	19.97764109	11.45341618	0.7	0.838405797	0.940793023	-0.088050734
P62270	40S ribosomal protein S18 OS=Mus musculus OX=10090 GN=Rps18 PE=1 SV=3 - [RS18_MOUSE]	Rps18	128.7064001	104.0494303	102.0497292	75.00237107	166.6652761	74.8046385	111.6018532	105.4907619	14.84667789	52.97877565	0.7	0.838405797	0.945242027	-0.08124432
P97493	Thioredoxin, mitochondrial OS=Mus musculus OX=10090 GN=Txn2 PE=1 SV=1 - [THIOM_MOUSE]	Txn2	121.2708894	109.8247293	166.8192021	131.0607727	123.4475948	122.7153381	132.6382736	125.7412352	30.14971891	4.62138067	0.7	0.838405797	0.948001144	-0.077039294
P62852	40S ribosomal protein S25 OS=Mus musculus OX=10090 GN=Rps25 PE=1 SV=1 - [RS25_MOUSE]	Rps25	178.1550745	140.4067487	66.86349529	184.9968539	181.8017828	0	128.4751062	122.2662122	56.59705798	105.8976964	0.7	0.838405797	0.951672397	-0.071463069
P97873	Lysyl oxidase homolog 1 OS=Mus musculus OX=10090 GN=Loxl1 PE=2 SV=3 - [LOXL1_MOUSE]	Loxl1	0	35.57287064	49.19646012	26.06893206	33.48935623	21.17713706	28.25644359	26.91180845	25.40119093	6.199235089	0.7	0.838405797	0.95241315	-0.070340554
Q61245	Collagen alpha-1(XI) chain OS=Mus musculus OX=10090 GN=Col11a1 PE=1 SV=2 - [COBA1_MOUSE]	Col11a1	188.3584062	185.7325177	180.0409522	137.4227979	197.4163642	203.3782471	184.7106254	179.4058031	4.251847609	36.48034473	0.7	0.838405797	0.971280362	-0.042040302
P43406	Integrin alpha-V OS=Mus musculus OX=10090 GN=Itgav PE=1 SV=2 - [ITAV_MOUSE]	Itgav	43.02964148	16.48781252	42.64203999	24.93454275	34.38983471	40.41379353	34.05316466	33.246057	15.21327564	7.802754117	0.7	0.838405797	0.9762986	-0.034605632
Q8VED5	Keratin, type II cytoskeletal 79 OS=Mus musculus OX=10090 GN=Krt79 PE=1 SV=2 - [K2C79_MOUSE]	Krt79	30.0479526	52.35949381	27.4691488	0	80.05525686	27.42324138	36.62553173	35.82616608	13.68688164	40.68375469	0.7	0.838405797	0.978174634	-0.031836042
Q62159	Rho-related GTP-binding protein RhoC OS=Mus musculus OX=10090 GN=Rhoc PE=1 SV=2 - [RHOC_MOUSE]	Rhoc	447.9321077	142.5024316	150.5646504	293.1785686	287.8276564	153.8475409	246.9997299	244.9512553	174.059229	78.94348071	0.7	0.838405797	0.991706571	-0.012014779
Q9CZT5	Vasorin OS=Mus musculus OX=10090 GN=Vasn PE=2 SV=2 - [VASN_MOUSE]	Vasn	77.92159653	58.58338839	77.44582436	87.71849594	36.42878844	88.64941438	71.31693643	70.93223292	11.03014162	29.88448449	0.7	0.838405797	0.99460572	-0.007803366
Q8CDN6	Thioredoxin-like protein 1 OS=Mus musculus OX=10090 GN=Txnl1 PE=1 SV=3 - [TXNL1_MOUSE]	Txnl1	134.4782879	41.81385983	32.20120705	68.65313508	75.18308607	64.60931939	69.49778492	69.48184684	56.4796432	5.335373262	0.7	0.838405797	0.999770668	-0.000330894
Q64449	C-type mannose receptor 2 OS=Mus musculus OX=10090 GN=Mrc2 PE=1 SV=3 - [MRC2_MOUSE]	Mrc2	34.29057351	25.70621764	36.63866384	23.00354273	38.58298609	36.71977717	32.21181833	32.76876867	5.755042334	8.508091148	0.7	0.838405797	1.017290248	0.024731361
P62806	Histone H4 OS=Mus musculus OX=10090 GN=H4c1 PE=1 SV=2 - [H4_MOUSE]	H4c1	3173.655894	1197.34088	1411.517396	1674.465815	2076.195821	2151.480721	1927.504723	1967.380786	1084.498709	256.4494861	0.7	0.838405797	1.020687919	0.029541822
Q7TMR0	Lysosomal Pro-X carboxypeptidase OS=Mus musculus OX=10090 GN=Prcp PE=1 SV=2 - [PCP_MOUSE]	Prcp	88.18756985	105.2726053	99.01199096	65.58080501	110.5452651	124.4813562	97.49072203	100.2024754	8.643512201	30.78227909	0.7	0.838405797	1.027815502	0.039581317
Q9JIX8	Apoptotic chromatin condensation inducer in the nucleus OS=Mus musculus OX=10090 GN=Acin1 PE=1 SV=3 - [ACINU_MOUSE]	Acin1	43.13948091	23.44592295	16.22049663	33.98635555	27.61905407	23.55510185	27.60196683	28.38683715	13.93242456	5.257840011	0.7	0.838405797	1.028435304	0.040451041
P20108	Thioredoxin-dependent peroxide reductase, mitochondrial OS=Mus musculus OX=10090 GN=Prdx3 PE=1 SV=1 - [PRDX3_MOUSE]	Prdx3	153.6890538	132.6706145	163.50762	166.8938423	164.2584818	131.6984639	149.9557627	154.283596	15.7538352	19.60363298	0.7	0.838405797	1.028860734	0.041047712
Q8BM72	Heat shock 70 kDa protein 13 OS=Mus musculus OX=10090 GN=Hspa13 PE=1 SV=1 - [HSP13_MOUSE]	Hspa13	22.96876374	30.5999359	56.88976043	32.85624777	37.02211372	44.07814346	36.81948669	37.98550165	17.79524131	5.672638095	0.7	0.838405797	1.03166842	0.04497936
Q9CR51	V-type proton ATPase subunit G 1 OS=Mus musculus OX=10090 GN=Atp6v1g1 PE=1 SV=3 - [VATG1_MOUSE]	Atp6v1g1	87.50044836	103.9288003	93.13707824	108.0707068	108.42933	77.22216613	94.8554423	97.90740099	8.34789003	17.91483627	0.7	0.838405797	1.03217484	0.04568737
P63038	60 kDa heat shock protein, mitochondrial OS=Mus musculus OX=10090 GN=Hspd1 PE=1 SV=1 - [CH60_MOUSE]	Hspd1	179.9986232	204.19887	206.8101768	176.3266571	263.3529932	170.4417376	197.0025567	203.373796	14.78360736	52.02678293	0.7	0.838405797	1.032340897	0.045919452
P42125	Enoyl-CoA delta isomerase 1, mitochondrial OS=Mus musculus OX=10090 GN=Eci1 PE=1 SV=2 - [ECI1_MOUSE]	Eci1	126.7393973	71.14635856	78.25720984	83.29908689	120.0400806	82.71287276	92.04765525	95.35068008	30.25357465	21.38365697	0.7	0.838405797	1.035883856	0.050862257
P62075	Mitochondrial import inner membrane translocase subunit Tim13 OS=Mus musculus OX=10090 GN=Timm13 PE=1 SV=1 - [TIM13_MOUSE]	Timm13	67.64519828	41.9624895	42.50850662	63.04239486	52.3263226	43.96004269	50.70539813	53.10958672	14.67283733	9.565258391	0.7	0.838405797	1.047414845	0.066832957
P84089	Enhancer of rudimentary homolog OS=Mus musculus OX=10090 GN=Erh PE=1 SV=1 - [ERH_MOUSE]	Erh	757.6079225	543.8948424	604.0539203	625.4161063	670.4674169	700.3295095	635.1855617	665.4043442	110.2052834	37.71247176	0.7	0.838405797	1.047574731	0.067053166
Q921E2	Ras-related protein Rab-31 OS=Mus musculus OX=10090 GN=Rab31 PE=1 SV=1 - [RAB31_MOUSE]	Rab31	74.41677371	27.17258018	34.40213587	60.78611921	41.99576962	40.34362538	45.33049659	47.70850474	25.44750001	11.35563276	0.7	0.838405797	1.052459345	0.073764505
Q9EPL2	Calsyntenin-1 OS=Mus musculus OX=10090 GN=Clstn1 PE=1 SV=1 - [CSTN1_MOUSE]	Clstn1	616.4456786	704.7238421	764.2115392	567.7150652	789.6916593	865.8052526	695.12702	741.070659	74.3489182	154.8787952	0.7	0.838405797	1.066093876	0.092334482
P10810	Monocyte differentiation antigen CD14 OS=Mus musculus OX=10090 GN=Cd14 PE=1 SV=1 - [CD14_MOUSE]	Cd14	86.83877397	17.12755213	23.87010789	77.28874087	33.03763675	25.99689696	42.61214466	45.44109153	38.44946774	27.80463236	0.7	0.838405797	1.066388277	0.092732826
Q8CIF4	Biotinidase OS=Mus musculus OX=10090 GN=Btd PE=1 SV=2 - [BTD_MOUSE]	Btd	66.54137048	62.60323085	98.26752073	79.07357169	80.04977748	85.54313678	75.80404069	81.55549532	19.55334211	3.487722432	0.7	0.838405797	1.075872666	0.105507339
P68033	Actin, alpha cardiac muscle 1 OS=Mus musculus OX=10090 GN=Actc1 PE=1 SV=1 - [ACTC_MOUSE]	Actc1	8650.260215	4031.143286	3875.952802	7053.731341	6537.655598	4282.674167	5519.118768	5958.020369	2712.758023	1473.659463	0.7	0.838405797	1.079523855	0.110395124
P11438	Lysosome-associated membrane glycoprotein 1 OS=Mus musculus OX=10090 GN=Lamp1 PE=1 SV=2 - [LAMP1_MOUSE]	Lamp1	187.8484691	0	190.9045788	136.9517249	157.9040866	115.6687167	126.251016	136.8415094	109.3472644	21.11790068	0.7	0.838405797	1.083884421	0.116210925
Q78JW9	Ubiquitin domain-containing protein UBFD1 OS=Mus musculus OX=10090 GN=Ubfd1 PE=1 SV=2 - [UBFD1_MOUSE]	Ubfd1	104.8966042	55.69425631	55.35204226	109.2012824	125.3209642	0	71.98096758	78.17408221	28.50629101	68.17881916	0.7	0.838405797	1.086038224	0.119074881
P16110	Galectin-3 OS=Mus musculus OX=10090 GN=Lgals3 PE=1 SV=3 - [LEG3_MOUSE]	Lgals3	217.4505159	167.1767685	179.9255149	250.450309	220.3417445	142.9528425	188.1842664	204.581632	26.13460644	55.45459569	0.7	0.838405797	1.087134625	0.120530607
Q62283	Tetraspanin-7 OS=Mus musculus OX=10090 GN=Tspan7 PE=1 SV=2 - [TSN7_MOUSE]	Tspan7	51.77624683	72.41960868	61.96082456	29.50807745	82.31868454	93.16570908	62.05222669	68.33082369	10.32198444	34.05611112	0.7	0.838405797	1.101182461	0.139053537
Q07235	Glia-derived nexin OS=Mus musculus OX=10090 GN=Serpine2 PE=1 SV=2 - [GDN_MOUSE]	Serpine2	2619.350495	1693.018983	2416.034547	1866.415277	2988.938746	2562.211189	2242.801342	2472.521737	486.8570889	566.610874	0.7	0.838405797	1.102425654	0.140681366
P08249	Malate dehydrogenase, mitochondrial OS=Mus musculus OX=10090 GN=Mdh2 PE=1 SV=3 - [MDHM_MOUSE]	Mdh2	1500.730553	1088.139958	1197.091098	1654.944453	1397.802752	1132.29105	1261.987203	1395.012752	213.813887	261.337871	0.7	0.838405797	1.105409586	0.144581029
Q60598	Src substrate cortactin OS=Mus musculus OX=10090 GN=Cttn PE=1 SV=2 - [SRC8_MOUSE]	Cttn	71.0630786	122.2038232	107.5185763	109.6652316	118.3275944	107.9426935	100.261826	111.9785065	26.33133604	5.565516369	0.7	0.838405797	1.116860833	0.159449429
O08749	Dihydrolipoyl dehydrogenase, mitochondrial OS=Mus musculus OX=10090 GN=Dld PE=1 SV=2 - [DLDH_MOUSE]	Dld	170.5072584	169.6688485	170.7319191	202.3592086	209.5263665	160.0977232	170.3026753	190.6610994	0.560286028	26.71014814	0.7	0.838405797	1.119542597	0.162909422
Q9CQ89	Protein CutA OS=Mus musculus OX=10090 GN=Cuta PE=1 SV=3 - [CUTA_MOUSE]	Cuta	100.6349034	83.80620353	85.27060652	123.5718679	116.4449232	63.03899759	89.90390448	101.0185962	9.322117375	33.08376897	0.7	0.838405797	1.123628576	0.168165221
Q9Z0H8	CAP-Gly domain-containing linker protein 2 OS=Mus musculus OX=10090 GN=Clip2 PE=1 SV=2 - [CLIP2_MOUSE]	Clip2	25.71083197	12.89275363	10.9120288	23.48768123	17.72508951	14.53445085	16.5052048	18.5824072	8.033585525	4.537766939	0.7	0.838405797	1.125851355	0.171016363
P16675	Lysosomal protective protein OS=Mus musculus OX=10090 GN=Ctsa PE=1 SV=1 - [PPGB_MOUSE]	Ctsa	210.0182859	204.2925223	201.1788437	162.8399566	273.5903471	259.3848726	205.1632173	231.9383921	4.483583039	60.2610506	0.7	0.838405797	1.130506702	0.176969544
P70372	ELAV-like protein 1 OS=Mus musculus OX=10090 GN=Elavl1 PE=1 SV=2 - [ELAV1_MOUSE]	Elavl1	430.6159372	139.7819858	135.7284489	295.4679043	321.8519531	184.5825682	235.3754573	267.3008085	169.0953622	72.8406478	0.7	0.838405797	1.135635854	0.183500304
O88952	Protein lin-7 homolog C OS=Mus musculus OX=10090 GN=Lin7c PE=1 SV=2 - [LIN7C_MOUSE]	Lin7c	87.41596982	50.29521803	45.90372928	65.07890899	81.81820368	61.92082582	61.20497238	69.6059795	22.80534112	10.69332441	0.7	0.838405797	1.137260206	0.185562382
Q6P069	Sorcin OS=Mus musculus OX=10090 GN=Sri PE=1 SV=1 - [SORCN_MOUSE]	Sri	301.4227627	98.68838965	108.4879629	234.689996	223.1172124	121.6063901	169.5330384	193.1378662	114.3248986	62.21773367	0.7	0.838405797	1.139234381	0.188064592
P12032	Metalloproteinase inhibitor 1 OS=Mus musculus OX=10090 GN=Timp1 PE=1 SV=2 - [TIMP1_MOUSE]	Timp1	518.0336324	608.2547396	545.4376263	690.1850216	795.1477153	421.9008792	557.2419994	635.7445387	46.25439939	192.4866873	0.7	0.838405797	1.140876925	0.190143165
P48678	Prelamin-A/C OS=Mus musculus OX=10090 GN=Lmna PE=1 SV=2 - [LMNA_MOUSE]	Lmna	227.3262728	195.9952764	57.75474271	230.7961011	196.6695347	121.6025648	160.3587639	183.0227335	90.22802621	55.86128783	0.7	0.838405797	1.141332903	0.190719657
P26041	Moesin OS=Mus musculus OX=10090 GN=Msn PE=1 SV=3 - [MOES_MOUSE]	Msn	1758.862349	1142.724698	1209.626137	1698.308069	1741.973659	1261.144367	1370.404395	1567.142032	338.0734203	265.8996033	0.7	0.838405797	1.143561738	0.193534256
P97464	Exostosin-1 OS=Mus musculus OX=10090 GN=Ext1 PE=1 SV=1 - [EXT1_MOUSE]	Ext1	60.36000715	29.40895476	46.31127534	46.70661597	55.60859174	53.51144911	45.36007908	51.94221894	15.497435	4.653832362	0.7	0.838405797	1.14510865	0.195484491
P02463	Collagen alpha-1(IV) chain OS=Mus musculus OX=10090 GN=Col4a1 PE=1 SV=4 - [CO4A1_MOUSE]	Col4a1	169.7598034	192.7845783	199.606309	260.2679871	133.9690094	250.9768334	187.3835636	215.0712766	15.63910786	70.39008947	0.7	0.838405797	1.147759561	0.19882045
P40336	Vacuolar protein sorting-associated protein 26A OS=Mus musculus OX=10090 GN=Vps26a PE=1 SV=1 - [VP26A_MOUSE]	Vps26a	46.40148488	59.81472375	79.38973089	84.04993449	84.37151973	45.18595081	61.86864651	71.20246834	16.58975717	22.53153885	0.7	0.838405797	1.150865137	0.202718783
Q9WV54	Acid ceramidase OS=Mus musculus OX=10090 GN=Asah1 PE=1 SV=1 - [ASAH1_MOUSE]	Asah1	77.15902036	78.49696583	91.37837636	55.76432187	126.6432475	102.0535298	82.34478751	94.82036639	7.851867297	35.98881095	0.7	0.838405797	1.151504172	0.203519638
A2A935	Histone-lysine N-methyltransferase PRDM16 OS=Mus musculus OX=10090 GN=Prdm16 PE=1 SV=1 - [PRD16_MOUSE]	Prdm16	377.6510155	0	87.62732177	235.1886964	205.3461162	99.32675804	155.0927791	179.9538569	197.6581982	71.40162253	0.7	0.838405797	1.1602981	0.214495505
P97461	40S ribosomal protein S5 OS=Mus musculus OX=10090 GN=Rps5 PE=1 SV=3 - [RS5_MOUSE]	Rps5	90.12824773	62.92510879	55.06697979	79.89392359	102.3677996	60.75390771	69.37344544	81.00521029	18.39861167	20.82919146	0.7	0.838405797	1.167668836	0.223631168
O88569	Heterogeneous nuclear ribonucleoproteins A2/B1 OS=Mus musculus OX=10090 GN=Hnrnpa2b1 PE=1 SV=2 - [ROA2_MOUSE]	Hnrnpa2b	3480.133051	1558.934107	1555.625826	3010.066387	2716.420096	1981.049585	2198.230995	2569.178689	1110.160978	530.0744463	0.7	0.838405797	1.168748278	0.224964239
Q8VHY0	Chondroitin sulfate proteoglycan 4 OS=Mus musculus OX=10090 GN=Cspg4 PE=1 SV=3 - [CSPG4_MOUSE]	Cspg4	292.8921121	123.079444	166.799087	265.7235371	228.2206215	188.0383281	194.256881	227.3274956	88.17331457	38.85030475	0.7	0.838405797	1.170241663	0.226806487
Q9CZC8	Secernin-1 OS=Mus musculus OX=10090 GN=Scrn1 PE=1 SV=1 - [SCRN1_MOUSE]	Scrn1	389.2530036	28.05027198	49.03231629	171.2201672	285.0259804	90.31424891	155.4451973	182.1867988	202.7550968	97.81801903	0.7	0.838405797	1.172032343	0.229012383
P23780	Beta-galactosidase OS=Mus musculus OX=10090 GN=Glb1 PE=1 SV=1 - [BGAL_MOUSE]	Glb1	78.97139173	52.37485582	44.77672579	75.60531965	79.96436135	51.53574428	58.70765778	69.03514176	17.95541924	15.31084567	0.7	0.838405797	1.175913746	0.233782241
P26231	Catenin alpha-1 OS=Mus musculus OX=10090 GN=Ctnna1 PE=1 SV=1 - [CTNA1_MOUSE]	Ctnna1	66.34026769	36.05673982	38.52500107	52.63645825	62.74295675	50.43391248	46.97400286	55.27110916	16.81702228	6.563854186	0.7	0.838405797	1.176631877	0.234663027
P61957	Small ubiquitin-related modifier 2 OS=Mus musculus OX=10090 GN=Sumo2 PE=1 SV=1 - [SUMO2_MOUSE]	Sumo2	826.8450746	917.8365822	999.0606327	757.3897321	1439.745476	1037.801578	914.5807631	1078.312262	86.15393132	342.9769333	0.7	0.838405797	1.179023554	0.237592541
P60710	Actin, cytoplasmic 1 OS=Mus musculus OX=10090 GN=Actb PE=1 SV=1 - [ACTB_MOUSE]	Actb	11260.65174	5101.6598	5091.425172	9979.517405	9211.460378	6116.456822	7151.245571	8435.811535	3558.853816	2045.001862	0.7	0.838405797	1.179628283	0.238332318
P70441	Na(+)/H(+) exchange regulatory cofactor NHE-RF1 OS=Mus musculus OX=10090 GN=Slc9a3r1 PE=1 SV=3 - [NHRF1_MOUSE]	Slc9a3r1	215.0365573	118.59781	114.8087274	132.2523665	204.0349821	197.452457	149.4810315	177.9132685	56.80435282	39.68023299	0.7	0.838405797	1.19020632	0.251211684
B9EKR1	Receptor-type tyrosine-protein phosphatase zeta OS=Mus musculus OX=10090 GN=Ptprz1 PE=1 SV=1 - [PTPRZ_MOUSE]	Ptprz1	4135.331175	4751.639312	5465.337254	3780.584494	6735.810708	6570.142748	4784.10258	5695.51265	665.597056	1660.443872	0.7	0.838405797	1.190508053	0.25157738
Q8K1J6	CCA tRNA nucleotidyltransferase 1, mitochondrial OS=Mus musculus OX=10090 GN=Trnt1 PE=1 SV=1 - [TRNT1_MOUSE]	Trnt1	96.13848279	54.31430309	35.28030243	99.22462099	76.08448218	46.25219702	61.91102944	73.85376673	31.13217335	26.55657158	0.7	0.838405797	1.192901611	0.254475056
P60122	RuvB-like 1 OS=Mus musculus OX=10090 GN=Ruvbl1 PE=1 SV=1 - [RUVB1_MOUSE]	Ruvbl1	84.24332028	22.91915385	24.5642953	68.76626956	60.30329272	28.96419053	43.90892314	52.6779176	34.94029646	20.96809798	0.7	0.838405797	1.199708711	0.262684163
Q05816	Fatty acid-binding protein 5 OS=Mus musculus OX=10090 GN=Fabp5 PE=1 SV=3 - [FABP5_MOUSE]	Fabp5	1260.390234	359.8451487	373.2326877	1037.506989	870.2588589	488.343351	664.4893567	798.7030665	516.1087075	281.4877422	0.7	0.838405797	1.201980225	0.265413161
Q9DB05	Alpha-soluble NSF attachment protein OS=Mus musculus OX=10090 GN=Napa PE=1 SV=1 - [SNAA_MOUSE]	Napa	35.92606638	22.52787367	19.48464668	30.81070647	35.42462812	27.57864295	25.97952891	31.27132585	8.747314633	3.943221837	0.7	0.838405797	1.203691028	0.267465118
Q7M6Y3	Phosphatidylinositol-binding clathrin assembly protein OS=Mus musculus OX=10090 GN=Picalm PE=1 SV=1 - [PICAL_MOUSE]	Picalm	165.6527031	74.49792252	103.5365793	144.659581	174.3187667	94.75287752	114.5624016	137.9104084	46.56688817	40.21002585	0.7	0.838405797	1.203801653	0.267597703
Q9CZ44	NSFL1 cofactor p47 OS=Mus musculus OX=10090 GN=Nsfl1c PE=1 SV=1 - [NSF1C_MOUSE]	Nsfl1c	233.777243	127.8987419	152.6173556	223.7535462	224.2368581	172.7225295	171.4311135	206.9043113	55.38982185	29.60327772	0.7	0.838405797	1.206923919	0.271334735
P10126	Elongation factor 1-alpha 1 OS=Mus musculus OX=10090 GN=Eef1a1 PE=1 SV=3 - [EF1A1_MOUSE]	Eef1a1	6564.742331	1513.060482	1867.587279	5089.347898	4719.635781	2217.406079	3315.130031	4008.796586	2819.824	1562.364145	0.7	0.838405797	1.209242639	0.274103755
P10605	Cathepsin B OS=Mus musculus OX=10090 GN=Ctsb PE=1 SV=2 - [CATB_MOUSE]	Ctsb	457.4293168	752.1673924	705.1310392	608.3546714	963.2828385	745.1226268	638.2425828	772.2533789	158.3451373	179.0127348	0.7	0.838405797	1.209968435	0.274969411
Q9JKV1	Proteasomal ubiquitin receptor ADRM1 OS=Mus musculus OX=10090 GN=Adrm1 PE=1 SV=2 - [ADRM1_MOUSE]	Adrm1	147.9720365	55.05607478	48.02279306	115.6092508	116.6865593	72.05619375	83.68363476	101.4506679	55.78633979	25.46205968	0.7	0.838405797	1.21231192	0.277760943
Q9WVK4	EH domain-containing protein 1 OS=Mus musculus OX=10090 GN=Ehd1 PE=1 SV=1 - [EHD1_MOUSE]	Ehd1	57.58925809	42.00886708	0	50.01371381	62.5963461	9.366304833	33.19937506	40.65878825	29.78818598	27.82077097	0.7	0.838405797	1.22468535	0.292411135
P27048	Small nuclear ribonucleoprotein-associated protein B OS=Mus musculus OX=10090 GN=Snrpb PE=1 SV=1 - [RSMB_MOUSE]	Snrpb	159.2348415	72.09467803	58.9393459	110.9759059	153.5610035	91.24928041	96.75628849	118.5953966	54.50635637	31.84698084	0.7	0.838405797	1.225712545	0.293620677
Q99JX3	Golgi reassembly-stacking protein 2 OS=Mus musculus OX=10090 GN=Gorasp2 PE=1 SV=3 - [GORS2_MOUSE]	Gorasp2	34.99342035	19.64518501	25.70734998	35.06203486	46.12474268	17.31299764	26.78198511	32.8332584	7.730343623	14.53460514	0.7	0.838405797	1.225945659	0.293895032
P31230	Aminoacyl tRNA synthase complex-interacting multifunctional protein 1 OS=Mus musculus OX=10090 GN=Aimp1 PE=1 SV=2 - [AIMP1_MOUSE]	Aimp1	166.6157648	53.97432709	72.45470127	138.8168081	130.1220739	90.4144476	97.68159774	119.7844432	60.40960689	25.8040129	0.7	0.838405797	1.226274406	0.29428185
Q9D8S9	BolA-like protein 1 OS=Mus musculus OX=10090 GN=Bola1 PE=1 SV=1 - [BOLA1_MOUSE]	Bola1	43.34482679	19.36141995	22.39397163	43.0532393	33.39562129	28.06840866	28.36673946	34.83908975	13.05972502	7.595985007	0.7	0.838405797	1.228166875	0.296506597
P83741	Serine/threonine-protein kinase WNK1 OS=Mus musculus OX=10090 GN=Wnk1 PE=1 SV=2 - [WNK1_MOUSE]	Wnk1	51.95536618	6.908051368	5.021815573	47.51573321	24.26840562	7.172215211	21.29507771	26.31878468	26.56933267	20.24976301	0.7	0.838405797	1.235909305	0.305572877
Q9D0K2	Succinyl-CoA:3-ketoacid coenzyme A transferase 1, mitochondrial OS=Mus musculus OX=10090 GN=Oxct1 PE=1 SV=1 - [SCOT1_MOUSE]	Oxct1	105.547379	43.03953278	62.17247842	122.325319	83.64677766	54.86592605	70.25313008	86.94600756	32.02780681	33.85049647	0.7	0.838405797	1.237610445	0.307557277
Q61792	LIM and SH3 domain protein 1 OS=Mus musculus OX=10090 GN=Lasp1 PE=1 SV=1 - [LASP1_MOUSE]	Lasp1	202.775716	49.71157495	49.78303259	176.0947672	139.0485298	59.02716763	100.7567745	124.7234882	88.35100224	59.83402736	0.7	0.838405797	1.23786702	0.307856339
Q9CQI6	Coactosin-like protein OS=Mus musculus OX=10090 GN=Cotl1 PE=1 SV=3 - [COTL1_MOUSE]	Cotl1	1026.810792	252.2964684	371.3407663	803.9708135	763.564035	475.9988444	550.1493421	681.1778976	417.0701251	178.8351471	0.7	0.838405797	1.238169067	0.308208322
P70158	Acid sphingomyelinase-like phosphodiesterase 3a OS=Mus musculus OX=10090 GN=Smpdl3a PE=1 SV=2 - [ASM3A_MOUSE]	Smpdl3a	47.44287841	50.79070613	47.22341684	45.02943581	69.126378	66.00872591	48.48566713	60.05484657	1.999235968	13.10542502	0.7	0.838405797	1.238610297	0.308722345
Q11136	Xaa-Pro dipeptidase OS=Mus musculus OX=10090 GN=Pepd PE=1 SV=3 - [PEPD_MOUSE]	Pepd	91.89995658	52.93265769	35.70406367	82.32613068	88.27445373	53.83825126	60.17889264	74.81294522	28.79019861	18.4064928	0.7	0.838405797	1.243175837	0.314030368
P97807	Fumarate hydratase, mitochondrial OS=Mus musculus OX=10090 GN=Fh PE=1 SV=3 - [FUMH_MOUSE]	Fh	222.4249641	81.50469883	96.21230551	200.4304958	183.1608794	115.7204055	133.3806561	166.4372602	77.46447578	44.762813	0.7	0.838405797	1.247836568	0.319428994
Q61072	Disintegrin and metalloproteinase domain-containing protein 9 OS=Mus musculus OX=10090 GN=Adam9 PE=1 SV=2 - [ADAM9_MOUSE]	Adam9	45.47755926	20.51951708	16.66739097	35.31528234	39.28177338	28.55751062	27.55482243	34.38485544	15.64059111	5.422335622	0.7	0.838405797	1.247870696	0.31946845
Q6IRU2	Tropomyosin alpha-4 chain OS=Mus musculus OX=10090 GN=Tpm4 PE=1 SV=3 - [TPM4_MOUSE]	Tpm4	152.8752536	98.33407741	59.01510566	159.1362287	136.4005686	91.86820378	103.4081456	129.1350004	47.13535266	34.21751242	0.7	0.838405797	1.248789442	0.320530245
Q61824	Disintegrin and metalloproteinase domain-containing protein 12 OS=Mus musculus OX=10090 GN=Adam12 PE=1 SV=2 - [ADA12_MOUSE]	Adam12	78.15321977	0	91.34915159	64.51438559	70.07941845	77.94175539	56.50079045	70.84518648	49.37395716	6.746359421	0.7	0.838405797	1.253879564	0.326398783
P06151	L-lactate dehydrogenase A chain OS=Mus musculus OX=10090 GN=Ldha PE=1 SV=3 - [LDHA_MOUSE]	Ldha	3203.016159	1233.599604	1577.199083	2903.200555	2907.85427	1748.870387	2004.604949	2519.975071	1051.977767	667.8002989	0.7	0.838405797	1.257093111	0.330091512
Q62261	Spectrin beta chain, non-erythrocytic 1 OS=Mus musculus OX=10090 GN=Sptbn1 PE=1 SV=2 - [SPTB2_MOUSE]	Sptbn1	122.4000182	71.61609575	59.20159221	132.4355607	120.9303698	65.95265039	84.40590206	106.439527	33.48424438	35.53143307	0.7	0.838405797	1.261043652	0.334618216

O55234	Proteasome subunit beta type-5 OS=Mus musculus OX=10090 GN=Psmb5 PE=1 SV=3 - [PSB5_MOUSE]	Psmb5	390.2222362	225.6113464	148.1280118	332.4083254	434.6584804	196.4673281	254.6538648	321.1780446	123.6325373	119.4920318	0.7	0.838405797	1.261233733	0.334835663
P24547	Inosine-5'-monophosphate dehydrogenase 2 OS=Mus musculus OX=10090 GN=Impdh2 PE=1 SV=2 - [IMDH2_MOUSE]	Impdh2	106.7380716	40.39014471	37.72885574	88.5296761	91.68276616	54.05552917	61.61902401	78.08932381	39.09689188	20.87349894	0.7	0.838405797	1.267292448	0.341749488
Q61990	Poly(rC)-binding protein 2 OS=Mus musculus OX=10090 GN=Pcbp2 PE=1 SV=1 - [PCBP2_MOUSE]	Pcbp2	939.7495433	266.4095808	290.3568108	722.8912993	783.0835965	395.7502032	498.838645	633.9083663	382.027725	208.4352685	0.7	0.838405797	1.270768359	0.345701074
Q3UH60	Disco-interacting protein 2 homolog B OS=Mus musculus OX=10090 GN=Dip2b PE=1 SV=1 - [DIP2B_MOUSE]	Dip2b	37.51822814	34.8968722	43.2603034	17.09001172	54.13032105	76.18025002	38.55846791	49.13352759	4.277653498	29.860342	0.7	0.838405797	1.27426037	0.349660094
Q9Z1G3	V-type proton ATPase subunit C 1 OS=Mus musculus OX=10090 GN=Atp6v1c1 PE=1 SV=4 - [VATC1_MOUSE]	Atp6v1c1	36.40244218	14.33769266	15.40447293	31.76740707	34.09995068	18.47784685	22.04820259	28.1150682	12.44257412	8.42717132	0.7	0.838405797	1.275163728	0.350682497
P62196	26S proteasome regulatory subunit 8 OS=Mus musculus OX=10090 GN=Psmc5 PE=1 SV=1 - [PRS8_MOUSE]	Psmc5	70.21669128	6.475544108	14.61797163	52.71621597	44.1120773	19.72484535	30.43673567	38.85104621	34.69017775	17.11334187	0.7	0.838405797	1.276452463	0.35213981
Q61425	Hydroxyacyl-coenzyme A dehydrogenase, mitochondrial OS=Mus musculus OX=10090 GN=Hadh PE=1 SV=2 - [HCDH_MOUSE]	Hadh	442.0343112	108.5028364	110.6192493	196.9703235	338.9427441	309.0142144	220.3854656	281.6424273	191.9564478	74.83951558	0.7	0.838405797	1.277953728	0.3538356
P16045	Galectin-1 OS=Mus musculus OX=10090 GN=Lgals1 PE=1 SV=3 - [LEG1_MOUSE]	Lgals1	6481.08018	1180.264506	2445.119088	5137.159646	4934.503645	2857.142217	3368.821258	4309.601836	2768.497964	1261.941588	0.7	0.838405797	1.279261055	0.355310701
Q91X96	Guanine nucleotide exchange factor MSS4 OS=Mus musculus OX=10090 GN=Rabif PE=1 SV=1 - [MSS4_MOUSE]	Rabif	75.83765158	40.92762141	32.34856133	73.09299352	74.85587883	43.41922899	49.70461144	63.78936711	23.03479777	17.66306419	0.7	0.838405797	1.283369194	0.359936258
P07310	Creatine kinase M-type OS=Mus musculus OX=10090 GN=Ckm PE=1 SV=1 - [KCRM_MOUSE]	Ckm	651.1772385	272.6527804	287.5265783	549.9118008	566.2634563	446.7539084	403.7855324	520.9763885	214.3765371	64.79642408	0.7	0.838405797	1.290230448	0.367628769
Q9D898	Actin-related protein 2/3 complex subunit 5-like protein OS=Mus musculus OX=10090 GN=Arpc5l PE=1 SV=1 - [ARP5L_MOUSE]	Arpc5l	134.8675637	74.08662127	0	98.79743321	76.56093849	94.3761308	69.65139498	89.91150084	67.54308518	11.77136844	0.7	0.838405797	1.29087868	0.368353419
Q3U0V1	Far upstream element-binding protein 2 OS=Mus musculus OX=10090 GN=Khsrp PE=1 SV=2 - [FUBP2_MOUSE]	Khsrp	384.2876911	59.02783792	74.29634621	298.4678939	259.4637231	110.6084651	172.5372917	222.846694	183.5400653	99.13826893	0.7	0.838405797	1.291585673	0.369143343
P16858	Glyceraldehyde-3-phosphate dehydrogenase OS=Mus musculus OX=10090 GN=Gapdh PE=1 SV=2 - [G3P_MOUSE]	Gapdh	1601.802906	382.6451718	492.5580433	1316.615015	1234.954741	652.4476034	825.6687071	1068.005786	674.3948628	362.1927011	0.7	0.838405797	1.293504014	0.371284531
P49442	Inositol polyphosphate 1-phosphatase OS=Mus musculus OX=10090 GN=Inpp1 PE=1 SV=2 - [INPP_MOUSE]	Inpp1	59.67384275	24.85791046	33.08609704	58.95039132	58.58193646	34.89892874	39.20595008	50.81041884	18.19688809	13.78098609	0.7	0.838405797	1.295987439	0.374051735
Q8C1B7	Septin-11 OS=Mus musculus OX=10090 GN=Septin11 PE=1 SV=4 - [SEP11_MOUSE]	Septin11	391.7389738	154.0226311	158.8949356	359.7309357	367.8525241	187.9373943	234.8855135	305.173618	135.8609247	101.6107237	0.7	0.838405797	1.299244102	0.37767251
Q9WV32	Actin-related protein 2/3 complex subunit 1B OS=Mus musculus OX=10090 GN=Arpc1b PE=1 SV=4 - [ARC1B_MOUSE]	Arpc1b	324.418647	78.16804544	98.15887078	208.1036109	314.2697036	128.2770539	166.9151877	216.8834562	136.7677339	93.30664876	0.7	0.838405797	1.299363222	0.377804776
Q9WUL7	ADP-ribosylation factor-like protein 3 OS=Mus musculus OX=10090 GN=Arl3 PE=1 SV=1 - [ARL3_MOUSE]	Arl3	146.7576582	41.28299785	59.9676214	127.7153634	133.990685	61.1469589	82.66942581	107.6176691	56.28281208	40.36694311	0.7	0.838405797	1.301783193	0.380489193
P99027	60S acidic ribosomal protein P2 OS=Mus musculus OX=10090 GN=Rplp2 PE=1 SV=3 - [RLA2_MOUSE]	Rplp2	299.0618092	68.447846	111.0733905	241.484242	245.4755845	137.7960161	159.5276819	208.2519475	122.7051905	61.04925397	0.7	0.838405797	1.305428281	0.384523199
Q920E5	Farnesyl pyrophosphate synthase OS=Mus musculus OX=10090 GN=Fdps PE=1 SV=1 - [FPPS_MOUSE]	Fdps	768.8106938	149.9076436	211.5505532	633.4203124	553.4502169	289.2087143	376.7562969	492.0264145	340.925138	180.139026	0.7	0.838405797	1.305954057	0.385104145
Q8BG05	Heterogeneous nuclear ribonucleoprotein A3 OS=Mus musculus OX=10090 GN=Hnrnpa3 PE=1 SV=1 - [ROA3_MOUSE]	Hnrnpa3	1636.108693	636.4416214	605.3765795	1451.357138	1434.810432	872.8502957	959.3089646	1253.005955	586.3315303	329.3283961	0.7	0.838405797	1.306154744	0.385325827
P63325	40S ribosomal protein S10 OS=Mus musculus OX=10090 GN=Rps10 PE=1 SV=1 - [RS10_MOUSE]	Rps10	178.8153517	33.83661009	34.51698633	147.2075974	132.2965925	43.37622178	82.38964936	107.6268039	83.5078007	56.13989218	0.7	0.838405797	1.306314625	0.385502411
P60335	Poly(rC)-binding protein 1 OS=Mus musculus OX=10090 GN=Pcbp1 PE=1 SV=1 - [PCBP1_MOUSE]	Pcbp1	1492.265891	572.7243422	645.6724573	1340.504186	1395.024145	805.9150559	903.5542302	1180.481129	511.1422714	325.5271321	0.7	0.838405797	1.306486196	0.385691881
Q91V64	Isochorismatase domain-containing protein 1 OS=Mus musculus OX=10090 GN=Isoc1 PE=1 SV=1 - [ISOC1_MOUSE]	Isoc1	97.1087658	53.15070652	57.79562972	117.4923215	108.9922797	46.4311484	69.35170068	90.97191652	24.15025519	38.80686404	0.7	0.838405797	1.31174745	0.391489985
P97494	Glutamate--cysteine ligase catalytic subunit OS=Mus musculus OX=10090 GN=Gclc PE=1 SV=4 - [GSH1_MOUSE]	Gclc	89.25245335	52.85457281	54.37258524	109.946843	100.1201384	47.79931249	65.4932038	85.95543128	20.59010794	33.40745548	0.7	0.838405797	1.312432837	0.392243596
P09671	Superoxide dismutase [Mn], mitochondrial OS=Mus musculus OX=10090 GN=Sod2 PE=1 SV=3 - [SODM_MOUSE]	Sod2	114.5837181	177.9655896	15.79199948	131.9860935	142.6009779	130.773707	102.7804357	135.1202595	81.72855338	6.506791219	0.7	0.838405797	1.314649607	0.39467833
O54782	Epididymis-specific alpha-mannosidase OS=Mus musculus OX=10090 GN=Man2b2 PE=1 SV=2 - [MA2B2_MOUSE]	Man2b2	41.59160933	0	19.18429844	23.18180709	30.67098707	26.07923391	20.25863593	26.64400936	20.81660737	3.776398109	0.7	0.838405797	1.315192664	0.395274157
Q9JII6	Aldo-keto reductase family 1 member A1 OS=Mus musculus OX=10090 GN=Akr1a1 PE=1 SV=3 - [AK1A1_MOUSE]	Akr1a1	285.1488778	140.2418437	104.0072929	242.0967996	284.3385053	169.9379566	176.4660048	232.1244205	95.84993915	57.8485751	0.7	0.838405797	1.315405881	0.395508026
P55065	Phospholipid transfer protein OS=Mus musculus OX=10090 GN=Pltp PE=1 SV=1 - [PLTP_MOUSE]	Pltp	75.9339966	52.25395522	87.31576553	132.3245854	48.24752062	104.0788093	71.83457245	94.88363846	17.88677149	42.78611437	0.7	0.838405797	1.320863133	0.401480984
Q9JJU8	SH3 domain-binding glutamic acid-rich-like protein OS=Mus musculus OX=10090 GN=Sh3bgrl PE=1 SV=1 - [SH3L1_MOUSE]	Sh3bgrl	522.6159834	200.4153372	203.6961691	498.4883377	448.3555399	279.6234289	308.9091632	408.8224355	185.082805	114.663036	0.7	0.838405797	1.323439005	0.404291705
O55137	Acyl-coenzyme A thioesterase 1 OS=Mus musculus OX=10090 GN=Acot1 PE=1 SV=1 - [ACOT1_MOUSE]	Acot1	759.3812577	248.8642866	298.0908556	622.8214117	665.9476193	440.5184837	435.4454666	576.4291716	281.6142953	119.6609951	0.7	0.838405797	1.323768912	0.404651295
P27546	Microtubule-associated protein 4 OS=Mus musculus OX=10090 GN=Map4 PE=1 SV=3 - [MAP4_MOUSE]	Map4	329.5258317	104.5392856	93.07354053	289.7319797	254.3203779	157.4085627	175.7128859	233.8203068	133.3292261	68.50227268	0.7	0.838405797	1.330695274	0.412180235
Q6NZB0	DnaJ homolog subfamily C member 8 OS=Mus musculus OX=10090 GN=Dnajc8 PE=1 SV=2 - [DNJC8_MOUSE]	Dnajc8	212.3957563	80.45941881	0	143.7480188	135.8649894	111.592008	97.61839171	130.4016721	107.2325141	16.75971762	0.7	0.838405797	1.335830982	0.41773748
P05201	Aspartate aminotransferase, cytoplasmic OS=Mus musculus OX=10090 GN=Got1 PE=1 SV=3 - [AATC_MOUSE]	Got1	425.7321315	130.540075	132.7725883	422.7093357	325.0276742	174.2872932	229.6815983	307.3414344	169.7884116	125.1518282	0.7	0.838405797	1.338119539	0.420207003
P99026	Proteasome subunit beta type-4 OS=Mus musculus OX=10090 GN=Psmb4 PE=1 SV=1 - [PSB4_MOUSE]	Psmb4	351.1793628	199.1085255	113.4730193	353.4788161	341.8830781	195.4190511	221.2536358	296.9269818	120.3905348	88.09943404	0.7	0.838405797	1.342020802	0.424407034
P70271	PDZ and LIM domain protein 4 OS=Mus musculus OX=10090 GN=Pdlim4 PE=1 SV=3 - [PDLI4_MOUSE]	Pdlim4	143.2665985	46.21926682	63.1619551	137.185019	130.518184	71.58039155	84.21594014	113.0945315	51.83626974	36.10650269	0.7	0.838405797	1.342911227	0.425363939
Q9WU78	Programmed cell death 6-interacting protein OS=Mus musculus OX=10090 GN=Pdcd6ip PE=1 SV=3 - [PDC6I_MOUSE]	Pdcd6ip	151.5824888	173.52065	168.3049836	109.3544851	288.3465109	267.8448744	164.4693741	221.8486235	11.46100581	97.96059237	0.7	0.838405797	1.348874978	0.431756637
P60824	Cold-inducible RNA-binding protein OS=Mus musculus OX=10090 GN=Cirbp PE=1 SV=1 - [CIRBP_MOUSE]	Cirbp	56.73710678	34.9339335	54.42588582	77.2055572	91.66207521	28.23448195	48.69897537	65.70070479	11.97675744	33.24208355	0.7	0.838405797	1.349118832	0.432017429
P17225	Polypyrimidine tract-binding protein 1 OS=Mus musculus OX=10090 GN=Ptbp1 PE=1 SV=2 - [PTBP1_MOUSE]	Ptbp1	466.0199431	130.9754302	160.0621553	409.4595162	409.8916383	203.0817294	252.3525095	340.8109613	185.6120646	119.2772094	0.7	0.838405797	1.350535257	0.433531303
P30275	Creatine kinase U-type, mitochondrial OS=Mus musculus OX=10090 GN=Ckmt1 PE=1 SV=1 - [KCRU_MOUSE]	Ckmt1	390.8131401	107.4711106	86.02044472	388.19388	269.1640577	133.3136007	194.7682318	263.5571795	170.1183039	127.5326115	0.7	0.838405797	1.353183612	0.436357611
P61027	Ras-related protein Rab-10 OS=Mus musculus OX=10090 GN=Rab10 PE=1 SV=1 - [RAB10_MOUSE]	Rab10	414.8707595	164.8850195	74.53168362	318.3385209	364.6802896	203.7759958	218.0958209	295.5982688	176.2986491	82.82745462	0.7	0.838405797	1.35535962	0.438675695
Q9R1P0	Proteasome subunit alpha type-4 OS=Mus musculus OX=10090 GN=Psma4 PE=1 SV=1 - [PSA4_MOUSE]	Psma4	452.5677118	248.7222013	144.4469652	457.6973511	444.9066629	244.9647754	281.9122928	382.5229298	156.7188095	119.3003973	0.7	0.838405797	1.356886307	0.440299843
Q91V92	ATP-citrate synthase OS=Mus musculus OX=10090 GN=Acly PE=1 SV=1 - [ACLY_MOUSE]	Acly	231.8198866	54.74625553	42.119658	186.6098006	176.6204764	85.32802638	109.5619334	149.5194345	106.0665503	55.81531423	0.7	0.838405797	1.36470241	0.448586388
Q8BLY1	SPARC-related modular calcium-binding protein 1 OS=Mus musculus OX=10090 GN=Smoc1 PE=2 SV=2 - [SMOC1_MOUSE]	Smoc1	378.9876615	97.57055914	101.9904614	310.5255809	333.6260875	150.650282	192.8495607	264.9339835	161.2154717	99.64427562	0.7	0.838405797	1.373785776	0.458157053
Q91VR8	Protein BRICK1 OS=Mus musculus OX=10090 GN=Brk1 PE=1 SV=1 - [BRK1_MOUSE]	Brk1	49.94683481	26.87914175	0	30.14932539	44.92437862	30.55532351	25.60865885	35.20967584	24.99764333	8.415628093	0.7	0.838405797	1.374912917	0.459340245
P62821	Ras-related protein Rab-1A OS=Mus musculus OX=10090 GN=Rab1A PE=1 SV=3 - [RAB1A_MOUSE]	Rab1A	484.340885	191.4501551	88.64687239	384.3387527	441.9298585	225.3492165	254.8126375	350.5392759	205.3157106	112.1766335	0.7	0.838405797	1.375674611	0.460139269
P11157	Ribonucleoside-diphosphate reductase subunit M2 OS=Mus musculus OX=10090 GN=Rrm2 PE=1 SV=1 - [RIR2_MOUSE]	Rrm2	35.15170056	23.43712076	44.99139306	28.63250061	77.71348503	36.35667794	34.52673813	47.56755453	10.7907181	26.39125941	0.7	0.838405797	1.377701952	0.462263814
Q99LT0	Protein dpy-30 homolog OS=Mus musculus OX=10090 GN=Dpy30 PE=1 SV=1 - [DPY30_MOUSE]	Dpy30	111.0714761	44.43004422	52.13780167	135.6653511	129.9809287	20.9750296	69.21310733	95.54043646	36.45469323	64.6380545	0.7	0.838405797	1.380380684	0.465066191
Q9D0F9	Phosphoglucomutase-1 OS=Mus musculus OX=10090 GN=Pgm1 PE=1 SV=4 - [PGM1_MOUSE]	Pgm1	257.4494177	119.7359744	81.34256326	218.0349383	301.5313589	114.3449201	152.8426518	211.3037391	92.60369923	93.77458343	0.7	0.838405797	1.382491972	0.467271103
O35737	Heterogeneous nuclear ribonucleoprotein H OS=Mus musculus OX=10090 GN=Hnrnph1 PE=1 SV=3 - [HNRH1_MOUSE]	Hnrnph1	384.2420803	126.7311188	150.1840813	343.9580456	380.8468811	189.9621715	220.3857601	304.9223661	142.3874314	101.2525619	0.7	0.838405797	1.383584701	0.468410966
P58252	Elongation factor 2 OS=Mus musculus OX=10090 GN=Eef2 PE=1 SV=2 - [EF2_MOUSE]	Eef2	1966.23202	778.9163447	908.4702507	1962.419176	1959.735582	1142.734315	1217.872872	1688.296358	651.3271945	472.4724934	0.7	0.838405797	1.386266495	0.471204627
P26516	26S proteasome non-ATPase regulatory subunit 7 OS=Mus musculus OX=10090 GN=Psmd7 PE=1 SV=2 - [PSMD7_MOUSE]	Psmd7	92.02386439	35.7051968	53.15914572	79.84131951	80.77897052	90.40283129	60.29606897	83.67437377	28.82966935	5.845844913	0.7	0.838405797	1.38772519	0.472721901
Q9JHU4	Cytoplasmic dynein 1 heavy chain 1 OS=Mus musculus OX=10090 GN=Dync1h1 PE=1 SV=2 - [DYHC1_MOUSE]	Dync1h1	104.6008057	9.540442913	18.63229094	65.80778723	85.70497017	33.14442207	44.2578465	61.55239316	52.45588589	26.53740922	0.7	0.838405797	1.390767921	0.475881696
O08553	Dihydropyrimidinase-related protein 2 OS=Mus musculus OX=10090 GN=Dpysl2 PE=1 SV=2 - [DPYL2_MOUSE]	Dpysl2	1083.226175	379.4458535	331.811736	1024.232574	980.8567767	491.582071	598.1612549	832.2238072	420.7531759	295.8005397	0.7	0.838405797	1.391303433	0.476437095
P35279	Ras-related protein Rab-6A OS=Mus musculus OX=10090 GN=Rab6a PE=1 SV=4 - [RAB6A_MOUSE]	Rab6a	258.9145273	110.9507259	0	193.4057461	210.2133888	116.1449443	123.2884177	173.254693	129.8974484	50.1673875	0.7	0.838405797	1.405279557	0.490857159
Q8BGD9	Eukaryotic translation initiation factor 4B OS=Mus musculus OX=10090 GN=Eif4b PE=1 SV=1 - [IF4B_MOUSE]	Eif4b	28.17950066	8.591322163	0	15.94678545	24.9821761	10.77556306	12.25694094	17.23484154	14.44294413	7.190360208	0.7	0.838405797	1.406129117	0.491729075
Q64737	Trifunctional purine biosynthetic protein adenosine-3 OS=Mus musculus OX=10090 GN=Gart PE=1 SV=3 - [PUR2_MOUSE]	Gart	271.8066542	34.97649756	72.99256073	222.0589091	214.7662914	98.49790275	126.5919042	178.4410344	127.1880418	69.32873715	0.7	0.838405797	1.409576984	0.495262272
P59999	Actin-related protein 2/3 complex subunit 4 OS=Mus musculus OX=10090 GN=Arpc4 PE=1 SV=3 - [ARPC4_MOUSE]	Arpc4	588.0388299	190.5259436	148.5266737	545.0438098	514.1675451	250.0302504	309.0304824	436.4138684	242.5391271	162.1495474	0.7	0.838405797	1.412203304	0.497947798
Q9WV91	Prostaglandin F2 receptor negative regulator OS=Mus musculus OX=10090 GN=Ptgfrn PE=1 SV=2 - [FPRP_MOUSE]	Ptgfrn	49.73623719	30.36436547	41.35685922	27.07523167	64.60667001	79.84624961	40.48582063	57.17605043	9.715265548	27.1588966	0.7	0.838405797	1.412248771	0.497994245
P14148	60S ribosomal protein L7 OS=Mus musculus OX=10090 GN=Rpl7 PE=1 SV=2 - [RL7_MOUSE]	Rpl7	97.84835275	40.95842824	55.95957078	91.75370726	139.6639131	43.67745789	64.92211726	91.69835942	29.48493356	47.99325155	0.7	0.838405797	1.412436367	0.498185873
P58389	Serine/threonine-protein phosphatase 2A activator OS=Mus musculus OX=10090 GN=Ptpa PE=1 SV=1 - [PTPA_MOUSE]	Ptpa	225.0881802	57.10610567	82.58011588	216.3822661	200.0332574	100.5385336	121.5914672	172.318019	90.53125698	62.69803361	0.7	0.838405797	1.417188417	0.50303158
Q62188	Dihydropyrimidinase-related protein 3 OS=Mus musculus OX=10090 GN=Dpysl3 PE=1 SV=1 - [DPYL3_MOUSE]	Dpysl3	776.4583461	298.9706194	264.4870862	742.5217837	736.6392789	425.7121364	446.6386839	634.957733	286.1521199	181.2358705	0.7	0.838405797	1.421636226	0.50755235
Q01405	Protein transport protein Sec23A OS=Mus musculus OX=10090 GN=Sec23a PE=1 SV=2 - [SC23A_MOUSE]	Sec23a	216.0197258	90.07636819	66.31696174	191.5502145	208.8764631	129.0408428	124.1376852	176.4891734	80.45408242	41.99473521	0.7	0.838405797	1.421721157	0.507638536
O89086	RNA-binding protein 3 OS=Mus musculus OX=10090 GN=Rbm3 PE=1 SV=1 - [RBM3_MOUSE]	Rbm3	544.0460654	85.66609638	117.4566896	404.7765552	464.1236416	195.9882643	249.0562838	354.9628204	255.9626709	140.8374995	0.7	0.838405797	1.425231337	0.51119611
Q3UEB3	Poly(U)-binding-splicing factor PUF60 OS=Mus musculus OX=10090 GN=Puf60 PE=1 SV=2 - [PUF60_MOUSE]	Puf60	255.5571226	73.28807917	31.88140997	202.5631529	259.5217828	53.56073699	120.2422039	171.8818909	119.000939	106.3531522	0.7	0.838405797	1.42946391	0.515474196
Q9CRC9	Glucosamine-6-phosphate isomerase 2 OS=Mus musculus OX=10090 GN=Gnpda2 PE=1 SV=1 - [GNPI2_MOUSE]	Gnpda2	62.75560862	32.14484724	29.34839024	64.43060892	85.48503713	28.41275059	41.41628203	59.44279888	18.53321849	28.86122242	0.7	0.838405797	1.435251934	0.521304
Q91Z31	Polypyrimidine tract-binding protein 2 OS=Mus musculus OX=10090 GN=Ptbp2 PE=1 SV=2 - [PTBP2_MOUSE]	Ptbp2	234.6167825	65.54286177	67.23085676	171.6781129	206.7752463	150.6214572	122.4635003	176.3582722	97.13125838	28.36793834	0.7	0.838405797	1.440088448	0.526157423
Q9JJV2	Profilin-2 OS=Mus musculus OX=10090 GN=Pfn2 PE=1 SV=3 - [PROF2_MOUSE]	Pfn2	424.555782	268.2780662	178.3972841	401.9641491	589.8769326	262.8888716	290.4103774	418.2433177	124.562759	164.1007511	0.7	0.838405797	1.440180346	0.526249484
Q60749	KH domain-containing, RNA-binding, signal transduction-associated protein 1 OS=Mus musculus OX=10090 GN=Khdrbs1 PE=1 SV=2 - [KHDR1_MOUSE]	Khdrbs1	305.5607496	0	56.16769396	225.6403671	211.1180023	84.97419187	120.5761479	173.9108538	162.6443347	77.36292484	0.7	0.838405797	1.442332143	0.52840343
P97298	Pigment epithelium-derived factor OS=Mus musculus OX=10090 GN=Serpinf1 PE=1 SV=2 - [PEDF_MOUSE]	Serpinf1	0	45.49805881	72.17060598	33.77598968	62.0658171	73.91939917	39.22288826	56.58706865	36.49222405	20.62488412	0.7	0.838405797	1.442705297	0.528776629
P16015	Carbonic anhydrase 3 OS=Mus musculus OX=10090 GN=Ca3 PE=1 SV=3 - [CAH3_MOUSE]	Ca3	261.1844425	0	17.37569337	215.5119567	80.74070283	105.9457619	92.85337863	134.0661405	146.0376287	71.65116528	0.7	0.838405797	1.443847736	0.529918607
Q5SUR0	Phosphoribosylformylglycinamidine synthase OS=Mus musculus OX=10090 GN=Pfas PE=1 SV=1 - [PUR4_MOUSE]	Pfas	357.7907174	39.84026461	33.37338255	198.4065072	346.9851109	83.68498809	143.6681215	209.6922021	185.4637962	132.0123625	0.7	0.838405797	1.459559712	0.545533234
P63085	Mitogen-activated protein kinase 1 OS=Mus musculus OX=10090 GN=Mapk1 PE=1 SV=3 - [MK01_MOUSE]	Mapk1	345.2951928	91.09546139	120.1985944	336.3257579	293.7896802	182.6437838	185.5297496	270.9197407	139.1240301	79.3524599	0.7	0.838405797	1.46024959	0.546214979
Q6NZJ6	Eukaryotic translation initiation factor 4 gamma 1 OS=Mus musculus OX=10090 GN=Eif4g1 PE=1 SV=1 - [IF4G1_MOUSE]	Eif4g1	24.5345748	26.71100873	29.99024869	52.47305489	46.400083	19.84170301	27.07861074	39.57161363	2.746350821	17.3543158	0.7	0.838405797	1.461360556	0.547312172
Q8VIJ6	Splicing factor, proline- and glutamine-rich OS=Mus musculus OX=10090 GN=Sfpq PE=1 SV=1 - [SFPQ_MOUSE]	Sfpq	516.5058121	131.631714	119.4252403	446.9871721	459.2799384	216.0986713	255.8542555	374.1219273	225.8133631	136.9901095	0.7	0.838405797	1.462246257	0.548186296
Q99LX0	Parkinson disease protein 7 homolog OS=Mus musculus OX=10090 GN=Park7 PE=1 SV=1 - [PARK7_MOUSE]	Park7	760.5478728	222.3398601	305.7381883	737.66702	730.7298758	416.1302512	429.5419737	628.1757157	289.6765424	183.6695136	0.7	0.838405797	1.462431506	0.548369057
Q8BWY3	Eukaryotic peptide chain release factor subunit 1 OS=Mus musculus OX=10090 GN=Etf1 PE=1 SV=4 - [ERF1_MOUSE]	Etf1	103.8753438	23.11666851	0	80.38722871	70.22323557	35.53806844	42.33067077	62.04951091	54.53810754	23.51529556	0.7	0.838405797	1.465828672	0.551716489
P40142	Transketolase OS=Mus musculus OX=10090 GN=Tkt PE=1 SV=1 - [TKT_MOUSE]	Tkt	2788.407895	757.605599	780.3287363	2531.330699	2574.526666	1248.452129	1442.114077	2118.103165	1165.980004	753.4495114	0.7	0.838405797	1.468748693	0.554587567
Q6ZWM4	U6 snRNA-associated Sm-like protein LSm8 OS=Mus musculus OX=10090 GN=Lsm8 PE=1 SV=3 - [LSM8_MOUSE]	Lsm8	146.7483988	26.6435854	36.21846097	84.29341947	134.260691	91.9478497	69.8701484	103.5006534	66.75042025	26.91249732	0.7	0.838405797	1.481328661	0.566891766
O54983	Ketimine reductase mu-crystallin OS=Mus musculus OX=10090 GN=Crym PE=1 SV=1 - [CRYM_MOUSE]	Crym	71.0024004	46.0865382	47.28462178	83.78679585	116.3009429	45.51761582	54.79118679	81.86845152	14.05209721	35.43063479	0.7	0.838405797	1.494190148	0.579363755
Q61029	Lamina-associated polypeptide 2, isoforms beta/delta/epsilon/gamma OS=Mus musculus OX=10090 GN=Tmpo PE=1 SV=4 - [LAP2B_MOUSE]	Tmpo	428.9600506	120.8827797	50.46564704	406.5796591	340.1712266	154.9646236	200.1028258	300.5718364	201.2991932	130.3979114	0.7	0.838405797	1.502086916	0.586968294
Q9CPU0	Lactoylglutathione lyase OS=Mus musculus OX=10090 GN=Glo1 PE=1 SV=3 - [LGUL_MOUSE]	Glo1	913.6619033	200.9456843	203.3804132	839.358289	857.0891888	285.2235518	439.3293336	660.5570098	410.785859	325.1691864	0.7	0.838405797	1.503557717	0.58838025
Q91WK2	Eukaryotic translation initiation factor 3 subunit H OS=Mus musculus OX=10090 GN=Eif3h PE=1 SV=1 - [EIF3H_MOUSE]	Eif3h	45.32574016	38.17067551	38.49962538	61.04963491	85.87426585	36.87152074	40.66534702	61.2651405	4.039368784	24.50208336	0.7	0.838405797	1.506568737	0.591266497
Q9QYS9	Protein quaking OS=Mus musculus OX=10090 GN=Qki PE=1 SV=1 - [QKI_MOUSE]	Qki	266.4955451	50.26238543	89.58645504	262.8964827	252.5381762	103.7292329	135.4481285	206.3879639	115.1810082	89.05579667	0.7	0.838405797	1.523741717	0.607618379
Q9JIF0	Protein arginine N-methyltransferase 1 OS=Mus musculus OX=10090 GN=Prmt1 PE=1 SV=1 - [ANM1_MOUSE]	Prmt1	346.4753105	99.07930633	99.69285928	320.5808168	322.1690666	189.7170807	181.7491587	277.488988	142.657362	76.01684962	0.7	0.838405797	1.526769037	0.610481834
P19096	Fatty acid synthase OS=Mus musculus OX=10090 GN=Fasn PE=1 SV=2 - [FAS_MOUSE]	Fasn	1014.238879	118.2398739	164.6750878	851.3456924	773.9638591	357.2552065	432.3846136	660.8549193	504.4351763	265.756611	0.7	0.838405797	1.528396012	0.612018399
Q7TQF7	Amphiphysin OS=Mus musculus OX=10090 GN=Amph PE=1 SV=1 - [AMPH_MOUSE]	Amph	74.19696022	58.50415549	0	69.20938804	71.13743129	62.5036349	44.23370524	67.61681807	39.10283288	4.531867092	0.7	0.838405797	1.528626592	0.612236033
Q9QZM0	Ubiquilin-2 OS=Mus musculus OX=10090 GN=Ubqln2 PE=1 SV=2 - [UBQL2_MOUSE]	Ubqln2	96.643087	23.70281978	28.1345502	96.52941843	93.89698611	36.58860387	49.49348566	75.67166947	40.89283236	33.87251006	0.7	0.838405797	1.528921806	0.612514624
P11983	T-complex protein 1 subunit alpha OS=Mus musculus OX=10090 GN=Tcp1 PE=1 SV=3 - [TCPA_MOUSE]	Tcp1	363.1890317	86.58399107	92.17370503	361.317584	330.2255074	141.0818242	180.6489093	277.5416385	158.1090872	119.1958053	0.7	0.838405797	1.536359337	0.619515686
O55131	Septin-7 OS=Mus musculus OX=10090 GN=Septin7 PE=1 SV=1 - [SEPT7_MOUSE]	Septin7	454.3296254	113.6099164	122.2720668	422.5452365	445.9751616	194.9523304	230.0705362	354.4909095	194.2623549	138.6602287	0.7	0.838405797	1.540792295	0.623672394
Q9CS84	Neurexin-1 OS=Mus musculus OX=10090 GN=Nrxn1 PE=1 SV=3 - [NRX1A_MOUSE]	Nrxn1	126.1050972	126.1199964	95.58022983	27.72696217	216.6493793	297.4099463	115.9351078	180.5954293	17.62784298	138.409333	0.7	0.838405797	1.557728566	0.639443866
Q9CWJ9	Bifunctional purine biosynthesis protein ATIC OS=Mus musculus OX=10090 GN=Atic PE=1 SV=2 - [PUR9_MOUSE]	Atic	175.3239908	41.46852949	51.05656249	160.8456668	174.7494833	82.0755799	89.2830276	139.2235767	74.66771799	49.97748672	0.7	0.838405797	1.559350981	0.640945688
Q9WTM5	RuvB-like 2 OS=Mus musculus OX=10090 GN=Ruvbl2 PE=1 SV=3 - [RUVB2_MOUSE]	Ruvbl2	78.770634	23.87116197	0	60.51668014	66.15439946	33.43483466	34.21393199	53.36863809	40.39099979	17.49180834	0.7	0.838405797	1.55985106	0.641408282
Q8VEK3	Heterogeneous nuclear ribonucleoprotein U OS=Mus musculus OX=10090 GN=Hnrnpu PE=1 SV=1 - [HNRPU_MOUSE]	Hnrnpu	269.457864	60.76962697	80.81577649	234.4640267	253.2449027	156.2120675	137.0144225	214.6403323	115.1364882	51.46432673	0.7	0.838405797	1.566552838	0.647593431
P11859	Angiotensinogen OS=Mus musculus OX=10090 GN=Agt PE=1 SV=1 - [ANGT_MOUSE]	Agt	121.684047	55.08177967	82.58159957	220.8914841	121.5870446	64.95915368	86.44914207	135.8125608	33.46914871	78.93349671	0.7	0.838405797	1.571011089	0.651693364
Q8CHH9	Septin-8 OS=Mus musculus OX=10090 GN=Septin8 PE=1 SV=4 - [SEPT8_MOUSE]	Septin8	361.2438377	141.3096584	0	301.9797026	318.0852074	170.3988613	167.517832	263.4879238	182.042382	81.01868297	0.7	0.838405797	1.572894781	0.653422165

P48428	Tubulin-specific chaperone A OS=Mus musculus OX=10090 GN=Tbca PE=1 SV=3 - [TBCA_MOUSE]	Tbca	157.5120572	21.10595633	23.03340523	127.7022346	123.737156	65.81862006	67.21713959	105.7526702	78.20363082	34.64068033	0.7	0.838405797	1.573299174	0.653793036
Q8VD75	Huntingtin-interacting protein 1 OS=Mus musculus OX=10090 GN=Hip1 PE=1 SV=2 - [HIP1_MOUSE]	Hip1	88.9807425	29.6602475	0	59.83128136	71.50199104	55.37548974	39.54699667	62.23625405	45.30677643	8.327900767	0.7	0.838405797	1.573728963	0.654187093
Q9R1P4	Proteasome subunit alpha type-1 OS=Mus musculus OX=10090 GN=Psma1 PE=1 SV=1 - [PSA1_MOUSE]	Psma1	538.8295288	175.90067	114.2240716	517.1888004	492.8737112	295.3504716	276.3180901	435.1376611	229.4236088	121.6681949	0.7	0.838405797	1.574770804	0.655141871
Q62417	Sorbin and SH3 domain-containing protein 1 OS=Mus musculus OX=10090 GN=Sorbs1 PE=1 SV=2 - [SRBS1_MOUSE]	Sorbs1	94.43049387	19.16731093	0	75.15571754	77.12337215	27.39063775	37.86593493	59.88990914	49.91501225	28.16238444	0.7	0.838405797	1.581630277	0.661412393
Q9CQI3	Glia maturation factor beta OS=Mus musculus OX=10090 GN=Gmfb PE=1 SV=3 - [GMFB_MOUSE]	Gmfb	97.19341904	0	55.13511167	86.96215173	79.44241208	74.97649491	50.7761769	80.46035291	48.74310628	6.0573216	0.7	0.838405797	1.58460833	0.664126292
P99029	Peroxiredoxin-5, mitochondrial OS=Mus musculus OX=10090 GN=Prdx5 PE=1 SV=2 - [PRDX5_MOUSE]	Prdx5	361.7767776	54.57685531	101.3894535	278.1343431	347.6546611	199.8839837	172.5810288	275.2243293	165.5117167	73.92830583	0.7	0.838405797	1.594754251	0.673334124
Q61656	Probable ATP-dependent RNA helicase DDX5 OS=Mus musculus OX=10090 GN=Ddx5 PE=1 SV=2 - [DDX5_MOUSE]	Ddx5	189.8937474	44.61658275	48.36760287	195.0198061	259.4830736	0	94.29264433	151.5009599	82.81422411	135.1047144	0.7	0.838405797	1.606710269	0.684109797
Q61753	D-3-phosphoglycerate dehydrogenase OS=Mus musculus OX=10090 GN=Phgdh PE=1 SV=3 - [SERA_MOUSE]	Phgdh	298.7686795	44.81069502	66.30456767	258.6246472	273.6616707	129.1902021	136.6279807	220.4921733	140.8286238	79.4264785	0.7	0.838405797	1.613814185	0.690474476
P97447	Four and a half LIM domains protein 1 OS=Mus musculus OX=10090 GN=Fhl1 PE=1 SV=3 - [FHL1_MOUSE]	Fhl1	427.2148313	87.72016528	47.94192554	430.4787658	421.0532964	66.59584897	187.6256407	306.0426371	208.4413881	207.4205465	0.7	0.838405797	1.631134401	0.705875661
Q6PCX7	Repulsive guidance molecule A OS=Mus musculus OX=10090 GN=Rgma PE=1 SV=1 - [RGMA_MOUSE]	Rgma	55.46347072	0	28.56874444	52.3692017	54.96553049	30.19284943	28.01073839	45.84252721	27.73594553	13.61504836	0.7	0.838405797	1.636605454	0.710706565
P08003	Protein disulfide-isomerase A4 OS=Mus musculus OX=10090 GN=Pdia4 PE=1 SV=3 - [PDIA4_MOUSE]	Pdia4	64.69091974	0	33.74439637	58.56780518	69.6621092	33.19739655	32.81177204	53.80910364	32.35554227	18.69231787	0.7	0.838405797	1.639932875	0.713636764
Q99KW3	TRIO and F-actin-binding protein OS=Mus musculus OX=10090 GN=Triobp PE=1 SV=3 - [TARA_MOUSE]	Triobp	21.98935054	0	8.316238377	21.98691661	17.16020411	10.69775337	10.10186297	16.61495803	11.10289267	5.664297984	0.7	0.838405797	1.644741972	0.717861271
Q9Z0N1	Eukaryotic translation initiation factor 2 subunit 3, X-linked OS=Mus musculus OX=10090 GN=Eif2s3x PE=1 SV=2 - [IF2G_MOUSE]	Eif2s3x	77.40636127	0	37.07372477	67.97537729	73.9656896	47.38935111	38.16002868	63.11013933	38.71461266	13.94017027	0.7	0.838405797	1.653828404	0.725809553
Q9JHR7	Insulin-degrading enzyme OS=Mus musculus OX=10090 GN=Ide PE=1 SV=1 - [IDE_MOUSE]	Ide	376.3649354	84.06518409	111.559607	360.8307622	337.6047651	250.4491963	190.6632422	316.2949079	161.4088751	58.19454469	0.7	0.838405797	1.658919172	0.730243595
P29758	Ornithine aminotransferase, mitochondrial OS=Mus musculus OX=10090 GN=Oat PE=1 SV=1 - [OAT_MOUSE]	Oat	75.50834723	63.20821123	60.02848401	126.3348479	146.7802958	59.54393314	66.24834749	110.886359	8.17547324	45.62387095	0.7	0.838405797	1.673798112	0.743125525
Q9CQ65	S-methyl-5'-thioadenosine phosphorylase OS=Mus musculus OX=10090 GN=Mtap PE=1 SV=1 - [MTAP_MOUSE]	Mtap	99.38407667	0	17.00316344	83.37611275	95.94980373	19.01060938	38.7957467	66.11217528	53.15530254	41.27278231	0.7	0.838405797	1.70410885	0.76901749
Q9JK81	MYG1 exonuclease OS=Mus musculus OX=10090 GN=Myg1 PE=1 SV=1 - [MYG1_MOUSE]	Myg1	135.2047995	68.71261572	0	120.7435415	172.7071575	55.27210283	67.97247173	116.2409339	67.60543846	58.84686162	0.7	0.838405797	1.710117802	0.774095709
P58044	Isopentenyl-diphosphate Delta-isomerase 1 OS=Mus musculus OX=10090 GN=Idi1 PE=1 SV=1 - [IDI1_MOUSE]	Idi1	269.6881113	26.61358173	47.9659344	261.5024695	238.8349171	89.25123927	114.7558758	196.529542	134.5993274	93.59449853	0.7	0.838405797	1.712588053	0.776178167
Q8VDM4	26S proteasome non-ATPase regulatory subunit 2 OS=Mus musculus OX=10090 GN=Psmd2 PE=1 SV=1 - [PSMD2_MOUSE]	Psmd2	132.7367833	23.88774036	14.42315312	119.23198	128.3158429	45.56092739	57.01589224	97.70291677	65.74674596	45.38413195	0.7	0.838405797	1.713608486	0.777037529
Q9QYJ0	DnaJ homolog subfamily A member 2 OS=Mus musculus OX=10090 GN=Dnaja2 PE=1 SV=1 - [DNJA2_MOUSE]	Dnaja2	25.81007997	13.84474796	0	22.49256538	23.65077335	22.36344392	13.21827597	22.83559422	12.91643942	0.708911735	0.7	0.838405797	1.727577353	0.788750309
Q80VP1	Epsin-1 OS=Mus musculus OX=10090 GN=Epn1 PE=1 SV=3 - [EPN1_MOUSE]	Epn1	26.85022631	8.950075436	0	24.41923768	24.01719683	13.69476697	11.93343391	20.7104005	13.67146605	6.079041419	0.7	0.838405797	1.735493794	0.795346206
P54763	Ephrin type-B receptor 2 OS=Mus musculus OX=10090 GN=Ephb2 PE=1 SV=3 - [EPHB2_MOUSE]	Ephb2	15.87869249	0	6.309178286	13.36580722	10.1849617	15.33750335	7.395956927	12.96275742	7.994938067	2.599809269	0.7	0.838405797	1.752681573	0.809563911
P26883	Peptidyl-prolyl cis-trans isomerase FKBP1A OS=Mus musculus OX=10090 GN=Fkbp1a PE=1 SV=2 - [FKB1A_MOUSE]	Fkbp1a	302.7565738	85.38933395	0	297.3260944	286.6620361	100.4184774	129.3819693	228.135536	156.0990062	110.734664	0.7	0.838405797	1.763271476	0.818254612
P56380	Bis(5'-nucleosyl)-tetraphosphatase [asymmetrical] OS=Mus musculus OX=10090 GN=Nudt2 PE=1 SV=3 - [AP4A_MOUSE]	Nudt2	82.31259615	0	27.02078962	71.26214632	73.60362662	54.23510901	36.44446193	66.36696065	41.95765808	10.57151837	0.7	0.838405797	1.821043779	0.864765606
P62315	Small nuclear ribonucleoprotein Sm D1 OS=Mus musculus OX=10090 GN=Snrpd1 PE=1 SV=1 - [SMD1_MOUSE]	Snrpd1	149.5533987	75.9786118	0	125.0989322	149.4224569	137.3907516	75.17733684	137.3040469	74.77991909	12.16199414	0.7	0.838405797	1.826402114	0.869004434
P61759	Prefoldin subunit 3 OS=Mus musculus OX=10090 GN=Vbp1 PE=1 SV=2 - [PFD3_MOUSE]	Vbp1	91.54118949	0	30.28728058	90.41246096	79.98840934	53.44068719	40.60949002	74.6138525	46.63537658	19.06285419	0.7	0.838405797	1.837350148	0.87762659
P46471	26S proteasome regulatory subunit 7 OS=Mus musculus OX=10090 GN=Psmc2 PE=1 SV=5 - [PRS7_MOUSE]	Psmc2	75.27840412	26.94055019	0	60.85306767	67.34449104	59.81059831	34.07298477	62.66938568	38.14266966	4.082173838	0.7	0.838405797	1.839269031	0.879132519
P42208	Septin-2 OS=Mus musculus OX=10090 GN=Septin2 PE=1 SV=2 - [SEPT2_MOUSE]	Septin2	651.8669358	77.8413676	82.2081357	432.6923851	567.8075393	496.5778973	270.638813	499.0259406	330.1604585	67.59083452	0.7	0.838405797	1.843881648	0.882746057
O88685	26S proteasome regulatory subunit 6A OS=Mus musculus OX=10090 GN=Psmc3 PE=1 SV=2 - [PRS6A_MOUSE]	Psmc3	82.96089659	24.81035571	0	75.2204585	64.34204059	66.25521457	35.92375077	68.60590455	42.58237021	5.807693315	0.7	0.838405797	1.909764518	0.933394759
Q99JI4	26S proteasome non-ATPase regulatory subunit 6 OS=Mus musculus OX=10090 GN=Psmd6 PE=1 SV=1 - [PSMD6_MOUSE]	Psmd6	108.6428935	0	15.71039018	91.9829494	101.7030627	49.71105462	41.45109456	81.13235556	58.71760787	27.64224603	0.7	0.838405797	1.957303092	0.968867177
P28660	Nck-associated protein 1 OS=Mus musculus OX=10090 GN=Nckap1 PE=1 SV=2 - [NCKP1_MOUSE]	Nckap1	51.30716209	9.804177415	0	41.63272073	49.54064104	30.39112059	20.3704465	40.52149412	27.2367537	9.623001185	0.7	0.838405797	1.989229549	0.992209767
P62264	40S ribosomal protein S14 OS=Mus musculus OX=10090 GN=Rps14 PE=1 SV=3 - [RS14_MOUSE]	Rps14	150.1162234	0	14.02513534	131.1984699	131.4495031	65.60710447	54.71378625	109.4183592	82.91800087	37.94186715	0.7	0.838405797	1.999831608	0.999878526
Q8R4G6	Alpha-1,6-mannosylglycoprotein 6-beta-N-acetylglucosaminyltransferase A OS=Mus musculus OX=10090 GN=Mgat5 PE=1 SV=1 - [MGT5A_MOUSE]	Mgat5	489.5460868	12.7332607	22.98060701	345.3411741	343.9731842	393.8257162	175.0866515	361.0466915	272.3780541	28.39570735	0.7	0.838405797	2.062102898	1.044116324
Q99P91	Transmembrane glycoprotein NMB OS=Mus musculus OX=10090 GN=Gpnmb PE=1 SV=2 - [GPNMB_MOUSE]	Gpnmb	84.82513034	24.61113556	0	14.58840028	47.3940318	0	36.4787553	20.66081069	43.64007314	24.27352856	0.824778095	0.977733869	0.566379267	-0.82015964
P35803	Neuronal membrane glycoprotein M6-b OS=Mus musculus OX=10090 GN=Gpm6b PE=1 SV=2 - [GPM6B_MOUSE]	Gpm6b	0	662.3189276	770.9203677	323.2332172	0	665.3513836	477.7464318	329.5282003	417.2886387	332.7203571	0.824778095	0.977733869	0.68975544	-0.535843165
Q9QYH6	Melanoma-associated antigen D1 OS=Mus musculus OX=10090 GN=Maged1 PE=1 SV=1 - [MAGD1_MOUSE]	Maged1	55.00382456	0	26.10820666	33.57379926	0	24.30601444	27.03734374	19.29327123	27.51368116	17.33913767	0.824778095	0.977733869	0.71357865	-0.486855645
Q9EPK2	Protein XRP2 OS=Mus musculus OX=10090 GN=Rp2 PE=1 SV=3 - [XRP2_MOUSE]	Rp2	22.22600464	17.49535109	0	12.95786064	18.2095883	0	13.24045191	10.38914965	11.70798641	9.372618827	0.824778095	0.977733869	0.784652195	-0.349874789
Q3TH73	Protein tweety homolog 2 OS=Mus musculus OX=10090 GN=Ttyh2 PE=1 SV=1 - [TTYH2_MOUSE]	Ttyh2	38.07534425	32.51956282	0	19.16583181	0	37.88062005	23.53163569	19.01548395	20.56745209	18.94075756	0.824778095	0.977733869	0.808081691	-0.30742695
Q9DC51	Guanine nucleotide-binding protein G(i) subunit alpha-3 OS=Mus musculus OX=10090 GN=Gnai3 PE=1 SV=3 - [GNAI3_MOUSE]	Gnai3	224.1379814	172.328268	0	0	199.4556388	172.3016898	132.1554164	123.9191095	117.3450134	108.1725174	0.824778095	0.977733869	0.937677114	-0.092836874
P14873	Microtubule-associated protein 1B OS=Mus musculus OX=10090 GN=Map1b PE=1 SV=2 - [MAP1B_MOUSE]	Map1b	66.79704602	44.04741065	0	67.96058098	46.94952429	0	36.94815222	38.30336842	33.95969585	34.79550474	0.824778095	0.977733869	1.036678863	0.051969052
Q9CQE6	Histone chaperone ASF1A OS=Mus musculus OX=10090 GN=Asf1a PE=1 SV=1 - [ASF1A_MOUSE]	Asf1a	57.32415757	0	51.5103829	58.05203478	55.48097736	0	36.27818015	37.84433738	31.55201674	32.79935957	0.824778095	0.977733869	1.043170777	0.06097536
P17809	Solute carrier family 2, facilitated glucose transporter member 1 OS=Mus musculus OX=10090 GN=Slc2a1 PE=1 SV=4 - [GTR1_MOUSE]	Slc2a1	0	16.92796865	21.58235818	0	17.58277052	24.18303823	12.83677561	13.92193625	11.35794618	12.5002446	0.824778095	0.977733869	1.084535297	0.117077008
Q9Z108	Double-stranded RNA-binding protein Staufen homolog 1 OS=Mus musculus OX=10090 GN=Stau1 PE=1 SV=1 - [STAU1_MOUSE]	Stau1	28.17669043	0	14.76360832	0	32.48065052	20.454587	14.31343291	17.64507917	14.09373848	16.42157621	0.824778095	0.977733869	1.232763606	0.301896176
Q8BH58	TIP41-like protein OS=Mus musculus OX=10090 GN=Tiprl PE=1 SV=1 - [TIPRL_MOUSE]	Tiprl	85.08138196	0	58.25087169	101.4338633	76.28902672	0	47.77741788	59.24096335	43.49690006	52.8221971	0.824778095	0.977733869	1.23993648	0.310266215
P60487	Pyridoxal phosphate phosphatase OS=Mus musculus OX=10090 GN=Pdxp PE=1 SV=1 - [PLPP_MOUSE]	Pdxp	33.85832586	10.21604	0	21.71129941	34.05905155	0	14.69145529	18.59011698	17.36716914	17.24271101	0.824778095	0.977733869	1.265369334	0.339558537
Q91VM9	Inorganic pyrophosphatase 2, mitochondrial OS=Mus musculus OX=10090 GN=Ppa2 PE=1 SV=1 - [IPYR2_MOUSE]	Ppa2	45.19091976	15.06363992	0	46.03197067	0	34.30414248	20.08485322	26.77870438	23.01009006	23.92090602	0.824778095	0.977733869	1.33327857	0.414978243
Q9D0A3	Arpin OS=Mus musculus OX=10090 GN=Arpin PE=1 SV=1 - [ARPIN_MOUSE]	Arpin	57.61403868	0	22.40747367	61.34677668	48.10546899	0	26.67383745	36.48408189	29.04299844	32.2823362	0.824778095	0.977733869	1.367785267	0.451841755
P52912	Nucleolysin TIA-1 OS=Mus musculus OX=10090 GN=Tia1 PE=1 SV=1 - [TIA1_MOUSE]	Tia1	94.0590307	0	29.84165626	62.87895437	109.2836007	0	41.30022899	57.38751836	48.06505467	54.84836596	0.824778095	0.977733869	1.389520585	0.474587208
Q8BUV3	Gephyrin OS=Mus musculus OX=10090 GN=Gphn PE=1 SV=2 - [GEPH_MOUSE]	Gphn	22.25817331	0	11.73754832	34.00332872	0	19.5910275	11.33190721	17.86478541	11.13462969	17.06726472	0.824778095	0.977733869	1.576502973	0.656727891
P12970	60S ribosomal protein L7a OS=Mus musculus OX=10090 GN=Rpl7a PE=1 SV=2 - [RL7A_MOUSE]	Rpl7a	89.96447976	0	29.98815992	72.70894162	119.2538095	0	39.98421323	63.9875837	45.80767093	60.10336373	0.824778095	0.977733869	1.60032119	0.678361488
Q99LI7	Cleavage stimulation factor subunit 3 OS=Mus musculus OX=10090 GN=Cstf3 PE=1 SV=1 - [CSTF3_MOUSE]	Cstf3	40.95155947	0	8.164685857	39.55009677	54.04978027	0	16.37208177	31.19995901	21.6743758	27.97567313	0.824778095	0.977733869	1.905680624	0.930306356
Q9QXZ0	Microtubule-actin cross-linking factor 1 OS=Mus musculus OX=10090 GN=Macf1 PE=1 SV=2 - [MACF1_MOUSE]	Macf1	24.19233559	8.064111863	0	16.91984515	60.63457703	0	10.75214915	25.85147406	12.31813434	31.28847198	0.824778095	0.977733869	2.404307613	1.26562149
O35242	Protein FAN OS=Mus musculus OX=10090 GN=Nsmaf PE=1 SV=2 - [FAN_MOUSE]	Nsmaf	0	93.1020771	279.3062313	234.986517	2524.642656	0	124.1361028	919.8763911	142.2157719	1394.726045	0.824778095	0.977733869	7.410224507	2.889517252
Q9QY15	ATP-dependent RNA helicase DDX25 OS=Mus musculus OX=10090 GN=Ddx25 PE=1 SV=2 - [DDX25_MOUSE]	Ddx25	228.1242824	0	0	0	0	24.09091344	76.04142747	8.030304481	131.7076159	13.90889536	1	1	0.105604336	-3.243259019
P04104	Keratin, type II cytoskeletal 1 OS=Mus musculus OX=10090 GN=Krt1 PE=1 SV=4 - [K2C1_MOUSE]	Krt1	0	562.3313831	0	0	133.6507013	0	187.4437944	44.55023375	324.6621754	77.16326835	1	1	0.237672492	-2.072953157
Q8BHT6	Beta-1,3-glucosyltransferase OS=Mus musculus OX=10090 GN=B3glct PE=1 SV=3 - [B3GLT_MOUSE]	B3glct	0	0	32.80779723	0	0	11.37369838	10.93593241	3.791232794	18.94159056	6.566607822	1	1	0.346676685	-1.528337281
Q99MR6	Serrate RNA effector molecule homolog OS=Mus musculus OX=10090 GN=Srrt PE=1 SV=1 - [SRRT_MOUSE]	Srrt	248.2688206	0	0	6.333762082	84.81096971	0	82.75627354	30.38157726	143.3380704	47.2434991	1	1	0.367121137	-1.445671913
Q62193	Replication protein A 32 kDa subunit OS=Mus musculus OX=10090 GN=Rpa2 PE=1 SV=1 - [RFA2_MOUSE]	Rpa2	68.62381563	0	0	0	26.64163784	0	22.87460521	8.880545948	39.61997842	15.38155678	1	1	0.388227288	-1.365026567
P21278	Guanine nucleotide-binding protein subunit alpha-11 OS=Mus musculus OX=10090 GN=Gna11 PE=1 SV=1 - [GNA11_MOUSE]	Gna11	0	23.79954609	0	0	0	9.924511423	7.933182031	3.308170474	13.74067434	5.729919341	1	1	0.417004231	-1.261866072
P18572	Basigin OS=Mus musculus OX=10090 GN=Bsg PE=1 SV=2 - [BASI_MOUSE]	Bsg	0	47.74063019	0	0	20.7557977	0	15.9135434	6.918599233	27.56306569	11.98336539	1	1	0.434761703	-1.201703231
Q922U2	Keratin, type II cytoskeletal 5 OS=Mus musculus OX=10090 GN=Krt5 PE=1 SV=1 - [K2C5_MOUSE]	Krt5	0	284.4994154	0	0	133.1830765	0	94.83313848	44.39435883	164.2558141	76.89328506	1	1	0.468131283	-1.095014917
Q9CY64	Biliverdin reductase A OS=Mus musculus OX=10090 GN=Blvra PE=1 SV=1 - [BIEA_MOUSE]	Blvra	146.8579006	0	0	35.78370385	37.62274395	0	48.95263354	24.46881593	84.78844846	21.21055709	1	1	0.499846774	-1.000442186
P29391	Ferritin light chain 1 OS=Mus musculus OX=10090 GN=Ftl1 PE=1 SV=2 - [FRIL1_MOUSE]	Ftl1	0	122.2691618	0	23.766277	37.53584873	0	40.75638727	20.43404191	70.59213349	18.98849199	1	1	0.501370295	-0.996051573
Q8K0Q5	Rho GTPase-activating protein 18 OS=Mus musculus OX=10090 GN=Arhgap18 PE=1 SV=1 - [RHG18_MOUSE]	Arhgap18	21.67976904	0	0	5.810325526	5.17300685	0	7.226589681	3.661110792	12.51682049	3.186587979	1	1	0.506616669	-0.981033549
P97825	Jupiter microtubule associated homolog 1 OS=Mus musculus OX=10090 GN=Jpt1 PE=1 SV=3 - [JUPI1_MOUSE]	Jpt1	126.6393703	0	0	0	68.14121068	0	42.21312344	22.71373689	73.11527455	39.34134633	1	1	0.53807288	-0.894126502
Q80YR4	E3 ubiquitin-protein ligase ZNF598 OS=Mus musculus OX=10090 GN=Znf598 PE=1 SV=1 - [ZN598_MOUSE]	Znf598	13.80397478	0	0	7.717252128	0	0	4.601324926	2.572417376	7.969728555	4.455557593	1	1	0.559060144	-0.838924598
Q8BY71	Histone acetyltransferase type B catalytic subunit OS=Mus musculus OX=10090 GN=Hat1 PE=1 SV=1 - [HAT1_MOUSE]	Hat1	33.84586441	0	0	19.59290073	0	0	11.2819548	6.530966911	19.54091893	11.31196651	1	1	0.578886108	-0.788648559
Q9Z2R6	Protein unc-119 homolog A OS=Mus musculus OX=10090 GN=Unc119 PE=1 SV=1 - [U119A_MOUSE]	Unc119	61.98508803	0	0	0	0	37.45886169	20.66169601	12.48628723	35.78710726	21.62688388	1	1	0.604320537	-0.726614124
Q9CQV3	Serpin B11 OS=Mus musculus OX=10090 GN=Serpinb11 PE=2 SV=1 - [SPB11_MOUSE]	Serpinb11	0	21.32781477	19.27930469	0	0	24.79479642	13.53570649	8.26493214	11.7669287	14.31528239	1	1	0.610602199	-0.711695311
Q8VCI5	Peroxisomal biogenesis factor 19 OS=Mus musculus OX=10090 GN=Pex19 PE=1 SV=1 - [PEX19_MOUSE]	Pex19	0	22.83884611	0	14.23177438	0	0	7.612948702	4.743924792	13.18601395	8.216718768	1	1	0.623138941	-0.682374219
P46414	Cyclin-dependent kinase inhibitor 1B OS=Mus musculus OX=10090 GN=Cdkn1b PE=1 SV=2 - [CDN1B_MOUSE]	Cdkn1b	30.36677311	18.31738901	0	30.49223506	0	0	16.22805404	10.16407835	15.29082135	17.60470012	1	1	0.626327613	-0.67501061
P08121	Collagen alpha-1(III) chain OS=Mus musculus OX=10090 GN=Col3a1 PE=1 SV=4 - [CO3A1_MOUSE]	Col3a1	0	37.19168522	0	0	0	23.36046979	12.39722841	7.786823264	21.47262947	13.48717352	1	1	0.62811001	-0.670910832
O09106	Histone deacetylase 1 OS=Mus musculus OX=10090 GN=Hdac1 PE=1 SV=1 - [HDAC1_MOUSE]	Hdac1	19.052905	0	0	0	12.1175775	0	6.350968332	4.039192501	11.00019983	6.996086633	1	1	0.635996322	-0.652909672
Q9QUI0	Transforming protein RhoA OS=Mus musculus OX=10090 GN=Rhoa PE=1 SV=1 - [RHOA_MOUSE]	Rhoa	447.9321077	0	0	0	287.8276564	0	149.3107026	95.94255213	258.613723	166.1773749	1	1	0.642569826	-0.63807486
O35988	Syndecan-4 OS=Mus musculus OX=10090 GN=Sdc4 PE=1 SV=1 - [SDC4_MOUSE]	Sdc4	0	80.47707149	78.96839451	0	0	102.9626793	53.14848867	34.3208931	46.03412226	59.44553062	1	1	0.645754827	-0.630941572
Q09143	High affinity cationic amino acid transporter 1 OS=Mus musculus OX=10090 GN=Slc7a1 PE=1 SV=1 - [SL7A1_MOUSE]	Slc7a1	0	22.32001852	13.59115778	0	0	23.47521039	11.9703921	7.825070131	11.24793171	13.55341904	1	1	0.653702073	-0.613294823
Q8VCN5	Cystathionine gamma-lyase OS=Mus musculus OX=10090 GN=Cth PE=1 SV=1 - [CGL_MOUSE]	Cth	24.40045286	0	0	16.05290224	0	0	8.133484285	5.350967412	14.08760802	9.268147427	1	1	0.657893619	-0.604073775
Q8VDP4	Cell cycle and apoptosis regulator protein 2 OS=Mus musculus OX=10090 GN=Ccar2 PE=1 SV=2 - [CCAR2_MOUSE]	Ccar2	76.65260711	0	0	34.94753667	16.486307	0	25.55086904	17.14461456	44.25540335	17.48306628	1	1	0.670999273	-0.575616891
Q571E4	N-acetylgalactosamine-6-sulfatase OS=Mus musculus OX=10090 GN=Galns PE=1 SV=2 - [GALNS_MOUSE]	Galns	0	16.73749332	26.96602868	0	0	29.34859318	14.56784067	9.782864393	13.61331041	16.94441817	1	1	0.671538399	-0.5744582
Q91VK1	Basic leucine zipper and W2 domain-containing protein 2 OS=Mus musculus OX=10090 GN=Bzw2 PE=1 SV=1 - [BZW2_MOUSE]	Bzw2	13.16444471	0	0	8.931355788	0	0	4.388148238	2.977118596	7.6004957	5.156520669	1	1	0.678445311	-0.559695569
O35874	Neutral amino acid transporter A OS=Mus musculus OX=10090 GN=Slc1a4 PE=1 SV=1 - [SATT_MOUSE]	Slc1a4	29.40919459	42.48909799	23.96609753	0	30.79975185	35.43022114	31.9547967	22.07665766	9.520265446	19.25861941	1	1	0.690871479	-0.53351074
Q9WUU7	Cathepsin Z OS=Mus musculus OX=10090 GN=Ctsz PE=1 SV=1 - [CATZ_MOUSE]	Ctsz	96.52254462	94.12759241	0	0	107.6316139	25.48535311	63.55004568	44.37232235	55.04897981	56.24659355	1	1	0.698226443	-0.5182331
Q9CXP8	Guanine nucleotide-binding protein G(I)/G(S)/G(O) subunit gamma-10 OS=Mus musculus OX=10090 GN=Gng10 PE=3 SV=1 - [GBG10_MOUSE]	Gng10	0	23.38579246	0	0	0	16.37430893	7.795264153	5.458102976	13.50179357	9.453711668	1	1	0.700181914	-0.514198298
Q6PDL0	Cytoplasmic dynein 1 light intermediate chain 2 OS=Mus musculus OX=10090 GN=Dync1li2 PE=1 SV=2 - [DC1L2_MOUSE]	Dync1li2	18.95467962	0	0	13.53037985	0	0	6.318226541	4.510126617	10.94348938	7.81176845	1	1	0.713827937	-0.48635173
P62631	Elongation factor 1-alpha 2 OS=Mus musculus OX=10090 GN=Eef1a2 PE=1 SV=1 - [EF1A2_MOUSE]	Eef1a2	6025.180958	0	0	4342.425862	0	0	2008.393653	1447.475287	3478.639848	2507.10074	1	1	0.720712937	-0.472503353
Q505B7	Protein archease OS=Mus musculus OX=10090 GN=Zbtb8os PE=1 SV=2 - [ARCH_MOUSE]	Zbtb8os	74.07201194	0	0	0	53.71136559	0	24.69067065	17.90378853	42.76549603	31.01027138	1	1	0.725123622	-0.463701122
P62307	Small nuclear ribonucleoprotein F OS=Mus musculus OX=10090 GN=Snrpf PE=1 SV=1 - [RUXF_MOUSE]	Snrpf	137.6774799	98.79863103	61.1862562	107.2414948	0	109.1064532	99.22078903	72.11598265	38.24735923	62.46123383	1	1	0.726823313	-0.460323399
Q8R1Q8	Cytoplasmic dynein 1 light intermediate chain 1 OS=Mus musculus OX=10090 GN=Dync1li1 PE=1 SV=1 - [DC1L1_MOUSE]	Dync1li1	39.95681572	0	0	0	29.04548229	0	13.31893857	9.681827429	23.06907831	16.76941702	1	1	0.726921847	-0.460127829
P14094	Sodium/potassium-transporting ATPase subunit beta-1 OS=Mus musculus OX=10090 GN=Atp1b1 PE=1 SV=1 - [AT1B1_MOUSE]	Atp1b1	42.55149194	116.2743432	124.0919878	0	69.03726259	138.9476418	94.30594098	69.32830147	44.99078948	69.47427811	1	1	0.73514246	-0.443904244
Q8K310	Matrin-3 OS=Mus musculus OX=10090 GN=Matr3 PE=1 SV=1 - [MATR3_MOUSE]	Matr3	118.2013907	0	0	39.59878921	47.36546185	0	39.40046358	28.98808369	68.24360476	25.40299289	1	1	0.735729508	-0.44275264
Q99JI6	Ras-related protein Rap-1b OS=Mus musculus OX=10090 GN=Rap1b PE=1 SV=2 - [RAP1B_MOUSE]	Rap1b	133.5568708	82.37830875	113.5687844	0	125.2690936	119.9068146	109.8346547	81.72530271	25.79281096	70.82695346	1	1	0.744075747	-0.4264786
P86048	60S ribosomal protein L10-like OS=Mus musculus OX=10090 GN=Rpl10l PE=2 SV=1 - [RL10L_MOUSE]	Rpl10l	70.56276962	21.75661915	20.54017997	45.66331604	0	38.8662875	37.61985624	28.17653451	28.53588245	24.63712082	1	1	0.748980388	-0.417000153
P62334	26S proteasome regulatory subunit 10B OS=Mus musculus OX=10090 GN=Psmc6 PE=1 SV=1 - [PRS10_MOUSE]	Psmc6	42.91659341	0	0	19.91962113	12.52098733	0	14.30553114	10.81353615	24.77790676	10.06898051	1	1	0.755898963	-0.403734685
Q0VBD0	Integrin beta-8 OS=Mus musculus OX=10090 GN=Itgb8 PE=1 SV=1 - [ITB8_MOUSE]	Itgb8	14.06885458	16.76781709	19.05942527	0	21.8921366	16.02929871	16.63203231	12.64047844	2.498054659	11.33267349	1	1	0.760008049	-0.395913397
Q91YS8	Calcium/calmodulin-dependent protein kinase type 1 OS=Mus musculus OX=10090 GN=Camk1 PE=1 SV=1 - [KCC1A_MOUSE]	Camk1	18.74609163	0	0	14.3069544	0	0	6.24869721	4.7689848	10.82306105	8.260123974	1	1	0.763196654	-0.389873249

P39688	Tyrosine-protein kinase Fyn OS=Mus musculus OX=10090 GN=Fyn PE=1 SV=4 - [FYN_MOUSE]	Fyn	111.8652404	61.03914095	59.09175932	0	108.3512757	71.242463	77.33204688	59.86457957	29.9224692	55.06443417	1	1	0.77412382	-0.369363752
Q91VM5	RNA binding motif protein, X-linked-like-1 OS=Mus musculus OX=10090 GN=Rbmxl1 PE=2 SV=1 - [RMXL1_MOUSE]	Rbmxl1	0	659.5704245	657.2955651	1029.597548	0	0	438.9553299	343.1991826	380.1481684	594.4384213	1	1	0.78185446	-0.355028015
Q9DBG9	Tax1-binding protein 3 OS=Mus musculus OX=10090 GN=Tax1bp3 PE=1 SV=1 - [TX1B3_MOUSE]	Tax1bp3	83.77635343	0	39.07881072	96.22483248	0	0	40.95172139	32.07494416	41.91956816	55.55543294	1	1	0.783237995	-0.352477342
Q6ZWV7	60S ribosomal protein L35 OS=Mus musculus OX=10090 GN=Rpl35 PE=1 SV=1 - [RL35_MOUSE]	Rpl35	106.4332219	64.2968546	55.42564657	78.73616134	100.8535581	0	75.38524103	59.86323982	27.25174162	53.00943709	1	1	0.794097611	-0.332611739
Q9JLQ0	CD2-associated protein OS=Mus musculus OX=10090 GN=Cd2ap PE=1 SV=3 - [CD2AP_MOUSE]	Cd2ap	23.86237981	0	0	19.26103368	0	0	7.954126602	6.420344561	13.7769514	11.12036298	1	1	0.807171533	-0.3090528
P47879	Insulin-like growth factor-binding protein 4 OS=Mus musculus OX=10090 GN=Igfbp4 PE=1 SV=2 - [IBP4_MOUSE]	Igfbp4	110.7309921	300.2529521	257.4972567	45.70535763	165.9820685	333.661024	222.8270669	181.7828167	99.4040234	144.6266372	1	1	0.815802224	-0.293708655
O88983	Syntaxin-8 OS=Mus musculus OX=10090 GN=Stx8 PE=1 SV=1 - [STX8_MOUSE]	Stx8	11.58952589	0	0	9.465615953	0	0	3.863175298	3.155205318	6.691215895	5.464975918	1	1	0.816738842	-0.292053255
P62500	TSC22 domain family protein 1 OS=Mus musculus OX=10090 GN=Tsc22d1 PE=1 SV=2 - [T22D1_MOUSE]	Tsc22d1	67.9169535	0	0	56.21887578	0	0	22.6389845	18.73962526	39.21187138	32.45798307	1	1	0.827759092	-0.272717142
O70340	Neuronal pentraxin-2 OS=Mus musculus OX=10090 GN=Nptx2 PE=2 SV=1 - [NPTX2_MOUSE]	Nptx2	122.6241021	214.9194291	174.4332457	37.79496189	157.733466	228.3290262	170.6589256	141.2858181	46.26327877	96.3260155	1	1	0.827884141	-0.272499212
P62488	DNA-directed RNA polymerase II subunit RPB7 OS=Mus musculus OX=10090 GN=Polr2g PE=1 SV=1 - [RPB7_MOUSE]	Polr2g	0	0	47.41304474	0	0	39.44062809	15.80434825	13.14687603	27.37393414	22.77105724	1	1	0.831851831	-0.265601515
Q6P8I4	PEST proteolytic signal-containing nuclear protein OS=Mus musculus OX=10090 GN=Pcnp PE=1 SV=1 - [PCNP_MOUSE]	Pcnp	52.72104333	0	0	44.0173527	0	0	17.57368111	14.6724509	30.43850856	25.41343043	1	1	0.834910501	-0.26030654
Q9R1T4	Septin-6 OS=Mus musculus OX=10090 GN=Septin6 PE=1 SV=4 - [SEPT6_MOUSE]	Septin6	211.6149106	0	0	177.2704756	0	0	70.53830354	59.09015855	122.1759256	102.3471568	1	1	0.837703143	-0.255489009
Q8K135	Dyslexia-associated protein KIAA0319-like protein OS=Mus musculus OX=10090 GN=Kiaa0319l PE=1 SV=1 - [K319L_MOUSE]	Kiaa0319l	30.99894159	0	37.95876463	19.96658273	0	38.04293226	22.98590207	19.336505	20.20825462	19.02929117	1	1	0.841233245	-0.249422229
P56546	C-terminal-binding protein 2 OS=Mus musculus OX=10090 GN=Ctbp2 PE=1 SV=2 - [CTBP2_MOUSE]	Ctbp2	99.63987964	0	0	0	70.22403943	13.66850497	33.21329321	27.96418147	57.52711133	37.23074943	1	1	0.841957504	-0.248180676
E9PVX6	Proliferation marker protein Ki-67 OS=Mus musculus OX=10090 GN=Mki67 PE=1 SV=1 - [KI67_MOUSE]	Mki67	21.77395827	0	0	8.138795905	10.20483986	0	7.257986088	6.114545254	12.57120067	5.395172115	1	1	0.84245756	-0.247324084
P62918	60S ribosomal protein L8 OS=Mus musculus OX=10090 GN=Rpl8 PE=1 SV=2 - [RL8_MOUSE]	Rpl8	29.65871054	18.52088749	16.9592852	22.52058668	33.09798946	0	21.71296108	18.53952538	6.925377342	16.90431494	1	1	0.853846019	-0.227952174
Q9WUK2	Eukaryotic translation initiation factor 4H OS=Mus musculus OX=10090 GN=Eif4h PE=1 SV=3 - [IF4H_MOUSE]	Eif4h	78.76598986	52.64442428	0	56.54192816	55.86503626	0	43.80347138	37.46898814	40.12034553	32.45086054	1	1	0.855388556	-0.225348189
Q8BFW7	Lipoma-preferred partner homolog OS=Mus musculus OX=10090 GN=Lpp PE=1 SV=1 - [LPP_MOUSE]	Lpp	10.70619962	0	0	0	9.214135808	0	3.568733205	3.071378603	6.18122723	5.319783789	1	1	0.860635533	-0.21652569
Q9Z1B5	Mitotic spindle assembly checkpoint protein MAD2A OS=Mus musculus OX=10090 GN=Mad2l1 PE=1 SV=2 - [MD2L1_MOUSE]	Mad2l1	34.29699321	0	0	29.60719889	0	0	11.43233107	9.869066296	19.80137826	17.09372425	1	1	0.863259316	-0.212134097
Q9Z0M5	Lysosomal acid lipase/cholesteryl ester hydrolase OS=Mus musculus OX=10090 GN=Lipa PE=1 SV=2 - [LICH_MOUSE]	Lipa	46.90978577	0	35.00411719	0	70.71781305	0	27.30463432	23.57260435	24.38428854	40.8289484	1	1	0.863318808	-0.212034676
P33434	72 kDa type IV collagenase OS=Mus musculus OX=10090 GN=Mmp2 PE=1 SV=1 - [MMP2_MOUSE]	Mmp2	77.56624702	35.92503954	30.20887159	57.31438788	33.85880782	33.48342084	47.90005272	41.55220551	25.85016359	13.65174068	1	1	0.86747724	-0.205102189
Q9DD18	D-aminoacyl-tRNA deacylase 1 OS=Mus musculus OX=10090 GN=Dtd1 PE=1 SV=2 - [DTD1_MOUSE]	Dtd1	37.08805077	0	0	5.221567204	26.95927896	0	12.36268359	10.72694872	21.4127961	14.29799285	1	1	0.867687719	-0.204752186
Q8JZM7	Parafibromin OS=Mus musculus OX=10090 GN=Cdc73 PE=1 SV=1 - [CDC73_MOUSE]	Cdc73	102.6910088	12.46973231	0	44.48275278	55.46284817	0	38.38691371	33.31520031	56.03691677	29.36950038	1	1	0.867879105	-0.204434005
Q922Y1	UBX domain-containing protein 1 OS=Mus musculus OX=10090 GN=Ubxn1 PE=1 SV=1 - [UBXN1_MOUSE]	Ubxn1	32.5224807	0	0	0	28.27220569	0	10.8408269	9.424068562	18.77686299	16.32296556	1	1	0.869312705	-0.202052864
Q60805	Tyrosine-protein kinase Mer OS=Mus musculus OX=10090 GN=Mertk PE=1 SV=1 - [MERTK_MOUSE]	Mertk	0	0	19.29623346	0	16.78481631	0	6.432077821	5.594938769	11.14068558	9.690718213	1	1	0.869849359	-0.201162519
Q9DB72	BTB/POZ domain-containing protein 17 OS=Mus musculus OX=10090 GN=Btbd17 PE=1 SV=1 - [BTBDH_MOUSE]	Btbd17	129.6867345	110.6979214	187.8157793	82.44025907	134.947556	157.397253	142.7334784	124.928356	40.1802706	38.46980649	1	1	0.875256159	-0.192222787
P47878	Insulin-like growth factor-binding protein 3 OS=Mus musculus OX=10090 GN=Igfbp3 PE=2 SV=2 - [IBP3_MOUSE]	Igfbp3	5148.392031	3557.142347	3277.090682	3709.137533	3667.108272	3130.201332	3994.208353	3502.149046	1009.312739	322.800931	1	1	0.8768068	-0.189669107
Q8BQ48	Centrosomal protein of 295 kDa OS=Mus musculus OX=10090 GN=Cep295 PE=1 SV=3 - [CE295_MOUSE]	Cep295	70.15829356	0	0	61.61663294	0	0	23.38609785	20.53887765	40.50590967	35.57437961	1	1	0.878251591	-0.18729381
Q08943	FACT complex subunit SSRP1 OS=Mus musculus OX=10090 GN=Ssrp1 PE=1 SV=2 - [SSRP1_MOUSE]	Ssrp1	0	37.3671877	0	24.49630557	8.324391182	0	12.45572923	10.94023225	21.57395587	12.45589078	1	1	0.878329325	-0.187166122
P61965	WD repeat-containing protein 5 OS=Mus musculus OX=10090 GN=Wdr5 PE=1 SV=1 - [WDR5_MOUSE]	Wdr5	48.42014758	0	0	42.58869286	0	0	16.14004919	14.19623095	27.95538524	24.58859329	1	1	0.879565532	-0.185137026
P35762	CD81 antigen OS=Mus musculus OX=10090 GN=Cd81 PE=1 SV=2 - [CD81_MOUSE]	Cd81	1924.729189	1704.52404	2408.09106	1156.698423	1724.816347	2433.042502	2012.448096	1771.519091	359.8924745	639.452431	1	1	0.880280636	-0.183964562
P55066	Neurocan core protein OS=Mus musculus OX=10090 GN=Ncan PE=1 SV=1 - [NCAN_MOUSE]	Ncan	1869.097642	2845.962636	2698.256284	1226.87218	2289.653108	3022.984669	2471.105521	2179.836652	526.5590242	903.0779362	1	1	0.882130137	-0.180936588
P51125	Calpastatin OS=Mus musculus OX=10090 GN=Cast PE=1 SV=2 - [ICAL_MOUSE]	Cast	17.86697476	0	0	15.76602529	0	0	5.955658252	5.255341762	10.31550269	9.102518943	1	1	0.882411572	-0.180476384
O35566	CD151 antigen OS=Mus musculus OX=10090 GN=Cd151 PE=1 SV=2 - [CD151_MOUSE]	Cd151	19.39556024	19.50777147	0	10.87818405	0	23.50954617	12.96777724	11.46257674	11.23056467	11.76566302	1	1	0.883927641	-0.177999821
Q8K0S0	Phytanoyl-CoA hydroxylase-interacting protein OS=Mus musculus OX=10090 GN=Phyhip PE=1 SV=1 - [PHYIP_MOUSE]	Phyhip	48.5519069	0	0	0	43.41338408	0	16.18396897	14.47112803	28.03145652	25.06472898	1	1	0.894164346	-0.161388074
Q80T85	DDB1- and CUL4-associated factor 5 OS=Mus musculus OX=10090 GN=Dcaf5 PE=1 SV=2 - [DCAF5_MOUSE]	Dcaf5	7.391848886	0	0	6.61265287	0	0	2.463949629	2.204217623	4.267685944	3.817816914	1	1	0.894587129	-0.160706094
Q3UHD6	Sorting nexin-27 OS=Mus musculus OX=10090 GN=Snx27 PE=1 SV=2 - [SNX27_MOUSE]	Snx27	12.60778627	0	0	0	11.33059104	0	4.202595425	3.77686368	7.279108799	6.541719788	1	1	0.898697899	-0.154091865
P98063	Bone morphogenetic protein 1 OS=Mus musculus OX=10090 GN=Bmp1 PE=1 SV=2 - [BMP1_MOUSE]	Bmp1	177.9535638	203.5226143	208.9735408	111.0086409	223.0786973	197.7536134	196.816573	177.2803172	16.56164203	58.77321906	1	1	0.900738767	-0.15081934
Q8VCC9	Spondin-1 OS=Mus musculus OX=10090 GN=Spon1 PE=1 SV=1 - [SPON1_MOUSE]	Spon1	55.4549538	29.26097424	38.08309391	42.43804049	68.60866976	0	40.93300732	37.01557009	13.32751462	34.62426578	1	1	0.904296374	-0.145132416
P14824	Annexin A6 OS=Mus musculus OX=10090 GN=Anxa6 PE=1 SV=3 - [ANXA6_MOUSE]	Anxa6	144.8384059	91.5582092	106.2435543	84.54155174	108.7417655	117.075376	114.2133898	103.4528977	27.51969384	16.8994533	1	1	0.905786072	-0.142757739
Q8R366	Immunoglobulin superfamily member 8 OS=Mus musculus OX=10090 GN=Igsf8 PE=1 SV=2 - [IGSF8_MOUSE]	Igsf8	14.9310113	136.2838136	175.1797761	30.9769638	90.56917821	174.4239578	108.7982003	98.65669995	83.58535073	72.06466562	1	1	0.906786139	-0.141165757
Q9Z315	U4/U6.U5 tri-snRNP-associated protein 1 OS=Mus musculus OX=10090 GN=Sart1 PE=1 SV=1 - [SNUT1_MOUSE]	Sart1	70.61404168	0	0	64.10673286	0	0	23.53801389	21.36891095	40.76903597	37.01203947	1	1	0.907846815	-0.139479209
Q9WVQ5	Methylthioribulose-1-phosphate dehydratase OS=Mus musculus OX=10090 GN=Apip PE=1 SV=1 - [MTNB_MOUSE]	Apip	85.86787581	0	0	55.93142668	22.77858327	0	28.62262527	26.23666998	49.57584121	28.12560925	1	1	0.916640935	-0.125571381
O88668	Protein CREG1 OS=Mus musculus OX=10090 GN=Creg1 PE=1 SV=1 - [CREG1_MOUSE]	Creg1	0	36.99700246	0	0	0	34.11829189	12.33233415	11.37276396	21.36022933	19.69820501	1	1	0.922190708	-0.116862966
Q07813	Apoptosis regulator BAX OS=Mus musculus OX=10090 GN=Bax PE=1 SV=1 - [BAX_MOUSE]	Bax	218.7915452	111.8082713	156.4302009	142.6836315	176.2423034	131.3853031	162.3433391	150.103746	53.73619939	23.33090638	1	1	0.924606743	-0.113088211
Q9DBC7	cAMP-dependent protein kinase type I-alpha regulatory subunit OS=Mus musculus OX=10090 GN=Prkar1a PE=1 SV=3 - [KAP0_MOUSE]	Prkar1a	50.05134976	0	0	22.16721871	24.139561	0	16.68378325	15.43559324	28.89716026	13.40394298	1	1	0.925185433	-0.112185544
Q9Z2Q6	Septin-5 OS=Mus musculus OX=10090 GN=Septin5 PE=1 SV=2 - [SEPT5_MOUSE]	Septin5	10.8675874	0	0	10.07268112	0	0	3.622529133	3.357560373	6.27440451	5.815465156	1	1	0.926855313	-0.109583951
P97857	A disintegrin and metalloproteinase with thrombospondin motifs 1 OS=Mus musculus OX=10090 GN=Adamts1 PE=1 SV=4 - [ATS1_MOUSE]	Adamts1	64.98299713	112.3437743	113.0236424	41.31802525	99.74620413	128.1820108	96.78347127	89.74874673	27.54211633	44.28656527	1	1	0.927314815	-0.108868891
P70677	Caspase-3 OS=Mus musculus OX=10090 GN=Casp3 PE=1 SV=1 - [CASP3_MOUSE]	Casp3	47.90822155	22.2309077	37.24108255	19.71280816	42.836429	37.4259502	35.79340393	33.32506245	12.89972642	12.09497501	1	1	0.931039208	-0.103086171
O54879	High mobility group protein B3 OS=Mus musculus OX=10090 GN=Hmgb3 PE=1 SV=3 - [HMGB3_MOUSE]	Hmgb3	69.14468454	0	112.9172336	0	97.64079583	72.11281166	60.68730604	56.58453583	56.93172093	50.63868842	1	1	0.93239492	-0.100986951
Q8BT60	Copine-3 OS=Mus musculus OX=10090 GN=Cpne3 PE=1 SV=2 - [CPNE3_MOUSE]	Cpne3	32.97706491	21.07774105	22.98389631	18.76658457	28.94052795	24.25754206	25.67956742	23.98821819	6.39127993	5.092316025	1	1	0.934136382	-0.0982949
E9Q7G0	Nuclear mitotic apparatus protein 1 OS=Mus musculus OX=10090 GN=Numa1 PE=1 SV=1 - [NUMA1_MOUSE]	Numa1	87.76841113	24.67528373	63.1818947	88.85303492	66.02479929	9.277169089	58.54186319	54.71833443	31.80146381	40.97507433	1	1	0.934687273	-0.097444346
P20352	Tissue factor OS=Mus musculus OX=10090 GN=F3 PE=1 SV=2 - [TF_MOUSE]	F3	20.73631131	0	28.05945611	0	0	45.65555	16.26525581	15.21851667	14.55424413	26.35924408	1	1	0.935645701	-0.095965764
O70318	Band 4.1-like protein 2 OS=Mus musculus OX=10090 GN=Epb41l2 PE=1 SV=2 - [E41L2_MOUSE]	Epb41l2	45.5089227	18.50415877	28.64750129	10.83553546	41.41379472	34.63516504	30.88686092	28.96149841	13.64094452	16.05927628	1	1	0.937664028	-0.092857009
O35664	Interferon alpha/beta receptor 2 OS=Mus musculus OX=10090 GN=Ifnar2 PE=1 SV=2 - [INAR2_MOUSE]	Ifnar2	66.52816664	30.74683685	101.1687839	50.6276738	77.60838991	58.60029272	66.14792912	62.27878547	35.21251327	13.86139395	1	1	0.941507713	-0.086955181
Q61129	Complement factor I OS=Mus musculus OX=10090 GN=Cfi PE=1 SV=3 - [CFAI_MOUSE]	Cfi	26.42215787	33.37531671	46.46167699	32.01122456	34.79980146	33.26940732	35.41971719	33.36014445	10.17498214	1.396501053	1	1	0.941852366	-0.086427157
Q9D1M4	Eukaryotic translation elongation factor 1 epsilon-1 OS=Mus musculus OX=10090 GN=Eef1e1 PE=1 SV=1 - [MCA3_MOUSE]	Eef1e1	38.77033862	42.95470515	59.63659996	41.18369673	46.72380146	45.27363334	47.12054791	44.39371051	11.03928893	2.872958104	1	1	0.942130609	-0.086001018
P62874	Guanine nucleotide-binding protein G(I)/G(S)/G(T) subunit beta-1 OS=Mus musculus OX=10090 GN=Gnb1 PE=1 SV=3 - [GBB1_MOUSE]	Gnb1	297.2944497	319.8251282	244.1239157	159.7027403	264.1904989	391.2312995	287.0811646	271.7081796	38.87032013	115.9472081	1	1	0.946450736	-0.079400681
Q8R422	CD109 antigen OS=Mus musculus OX=10090 GN=Cd109 PE=1 SV=1 - [CD109_MOUSE]	Cd109	603.3154913	504.1260836	962.5557851	692.3952024	367.6375018	900.1249203	689.99912	653.3858748	241.1949138	268.3784798	1	1	0.946937258	-0.078659256
Q61361	Brevican core protein OS=Mus musculus OX=10090 GN=Bcan PE=1 SV=2 - [PGCB_MOUSE]	Bcan	272.2110567	465.5497761	331.1846308	159.5871291	341.4015647	512.3144122	356.3151545	337.767702	99.08896271	176.3917169	1	1	0.947946495	-0.077122463
P10518	Delta-aminolevulinic acid dehydratase OS=Mus musculus OX=10090 GN=Alad PE=1 SV=1 - [HEM2_MOUSE]	Alad	55.16319432	78.61744038	34.49648281	62.50418126	39.90407977	57.12240502	56.0923725	53.17688868	22.07515013	11.80535773	1	1	0.948023525	-0.077005236
P31650	Sodium- and chloride-dependent GABA transporter 3 OS=Mus musculus OX=10090 GN=Slc6a11 PE=1 SV=2 - [S6A11_MOUSE]	Slc6a11	29.26388486	50.84941993	48.68995983	25.4095775	46.33221638	50.87724856	42.93442154	40.87301415	11.88816625	13.58317835	1	1	0.95198707	-0.070986117
Q9D964	Glycine amidinotransferase, mitochondrial OS=Mus musculus OX=10090 GN=Gatm PE=1 SV=1 - [GATM_MOUSE]	Gatm	21.74451	30.08236834	60.42747087	28.48573737	43.65979382	34.76656828	37.4181164	35.63736649	20.35811301	7.624415679	1	1	0.952409419	-0.070346207
P62897	Cytochrome c, somatic OS=Mus musculus OX=10090 GN=Cycs PE=1 SV=2 - [CYC_MOUSE]	Cycs	1170.27159	763.5524391	858.5329738	1039.175454	918.6639339	702.9108247	930.7856677	886.9167374	212.7685499	170.3654558	1	1	0.952868924	-0.069650324
P39038	Cadherin-4 OS=Mus musculus OX=10090 GN=Cdh4 PE=2 SV=1 - [CADH4_MOUSE]	Cdh4	76.83064686	138.9905975	164.4667838	26.63499069	153.4681447	182.3934392	126.762676	120.8321915	45.07953717	82.8492767	1	1	0.953215846	-0.06912516
Q8R3Q6	Protein MIX23 OS=Mus musculus OX=10090 GN=Mix23 PE=1 SV=1 - [MIX23_MOUSE]	Mix23	95.36445129	44.96056295	0	59.06961716	74.92494875	0	46.77500475	44.6648553	47.70811033	39.48493205	1	1	0.954887243	-0.066597712
Q99MD9	Nuclear autoantigenic sperm protein OS=Mus musculus OX=10090 GN=Nasp PE=1 SV=2 - [NASP_MOUSE]	Nasp	120.1541563	44.293598	60.69531628	84.7320403	87.11239175	44.29801025	75.04769018	72.04748077	39.91489762	24.06120017	1	1	0.960022628	-0.058859684
Q3TXS7	26S proteasome non-ATPase regulatory subunit 1 OS=Mus musculus OX=10090 GN=Psmd1 PE=1 SV=1 - [PSMD1_MOUSE]	Psmd1	88.36627814	64.58976125	20.22527526	79.57689327	57.26598934	29.4762395	57.72710489	55.43970737	34.58498385	25.10020631	1	1	0.960375676	-0.058329231
P68181	cAMP-dependent protein kinase catalytic subunit beta OS=Mus musculus OX=10090 GN=Prkacb PE=1 SV=2 - [KAPCB_MOUSE]	Prkacb	8.901951405	0	0	0	8.558232345	0	2.967317135	2.852744115	5.13954404	4.941097748	1	1	0.961388347	-0.056808778
O70401	Tetraspanin-6 OS=Mus musculus OX=10090 GN=Tspan6 PE=1 SV=1 - [TSN6_MOUSE]	Tspan6	0	0	46.35343341	0	0	44.67585158	15.45114447	14.89195053	26.76216726	25.79361494	1	1	0.963808898	-0.053180974
Q811B1	Xylosyltransferase 1 OS=Mus musculus OX=10090 GN=Xylt1 PE=1 SV=1 - [XYLT1_MOUSE]	Xylt1	214.581763	289.3690148	300.0983775	176.8060324	261.901935	336.2596136	268.0163851	258.322527	46.58566233	79.78703068	1	1	0.963831099	-0.053147743
P56564	Excitatory amino acid transporter 1 OS=Mus musculus OX=10090 GN=Slc1a3 PE=1 SV=2 - [EAA1_MOUSE]	Slc1a3	200.5408389	286.2741013	262.7410509	108.2264099	281.4288952	333.393608	249.851997	241.0163044	44.29609299	117.8980487	1	1	0.964636294	-0.051943003
Q5XJY5	Coatomer subunit delta OS=Mus musculus OX=10090 GN=Arcn1 PE=1 SV=2 - [COPD_MOUSE]	Arcn1	118.4393047	91.70567062	94.43453074	107.1701198	111.2541011	75.59998293	101.526502	98.00806794	14.71033107	19.51310921	1	1	0.965344673	-0.050883951
P63087	Serine/threonine-protein phosphatase PP1-gamma catalytic subunit OS=Mus musculus OX=10090 GN=Ppp1cc PE=1 SV=1 - [PP1G_MOUSE]	Ppp1cc	171.1793474	47.81956586	70.55453196	0	174.2441074	106.0680947	96.51781506	93.43740071	65.6504225	87.8060537	1	1	0.9680845	-0.046795115
Q9CYK2	Glutaminyl-peptide cyclotransferase OS=Mus musculus OX=10090 GN=Qpct PE=1 SV=2 - [QPCT_MOUSE]	Qpct	66.1499851	66.50873439	87.77727882	72.60075245	78.43501272	63.4414211	73.4786661	71.49239542	12.38426096	7.557995031	1	1	0.972968063	-0.039535645
Q9DCJ9	N-acetylneuraminate lyase OS=Mus musculus OX=10090 GN=Npl PE=1 SV=1 - [NPL_MOUSE]	Npl	65.7039854	20.96587486	33.19837545	48.92768815	37.02491687	30.74664689	39.95607857	38.89975064	23.12194947	9.234382306	1	1	0.973562773	-0.038654092
A2A8L5	Receptor-type tyrosine-protein phosphatase F OS=Mus musculus OX=10090 GN=Ptprf PE=1 SV=1 - [PTPRF_MOUSE]	Ptprf	46.41846454	28.65713976	35.9974003	28.32936458	39.91470464	40.10277945	37.02433487	36.11561623	8.92508322	6.743747402	1	1	0.975456179	-0.035851031
Q80ZV0	Ribonuclease H2 subunit B OS=Mus musculus OX=10090 GN=Rnaseh2b PE=1 SV=1 - [RNH2B_MOUSE]	Rnaseh2b	28.56805583	0	0	27.90415752	0	0	9.522685275	9.301385839	16.49377472	16.11047285	1	1	0.976760816	-0.033922769
Q61581	Insulin-like growth factor-binding protein 7 OS=Mus musculus OX=10090 GN=Igfbp7 PE=1 SV=1 - [IBP7_MOUSE]	Igfbp7	30.5903266	73.79156638	31.19608968	61.65245977	41.15702136	30.10934033	45.19266089	44.30627382	24.76923059	16.00563773	1	1	0.980386482	-0.028577503
Q80UY2	E3 ubiquitin-protein ligase KCMF1 OS=Mus musculus OX=10090 GN=Kcmf1 PE=1 SV=1 - [KCMF1_MOUSE]	Kcmf1	8.690889981	0	0	0	8.539174709	0	2.896963327	2.84639157	5.01768767	4.930094817	1	1	0.982543183	-0.025407279
Q05909	Receptor-type tyrosine-protein phosphatase gamma OS=Mus musculus OX=10090 GN=Ptprg PE=1 SV=1 - [PTPRG_MOUSE]	Ptprg	60.65471986	52.9504394	72.02000772	53.85405925	61.75485389	66.84231621	61.87505566	60.81707645	9.593175883	6.544713486	1	1	0.982901362	-0.024881452
P54103	DnaJ homolog subfamily C member 2 OS=Mus musculus OX=10090 GN=Dnajc2 PE=1 SV=2 - [DNJC2_MOUSE]	Dnajc2	36.45157436	25.50062814	24.85012809	31.75301796	28.39158397	25.22343926	28.9341102	28.45601373	6.518434487	3.265266133	1	1	0.983476372	-0.024037702
Q9CYS6	Uncharacterized protein C2orf72 homolog OS=Mus musculus OX=10090 PE=1 SV=2 - [CB072_MOUSE]	Not availa	81.78521032	68.93388346	86.62194017	57.78376541	98.11016786	77.97568918	79.11367798	77.95654082	9.141643799	20.16320804	1	1	0.985373741	-0.021257069
Q9D379	Epoxide hydrolase 1 OS=Mus musculus OX=10090 GN=Ephx1 PE=1 SV=2 - [HYEP_MOUSE]	Ephx1	85.3018966	9.993742175	21.15466874	57.9327927	56.9602994	0	38.81676917	38.29769737	40.64224249	33.17034298	1	1	0.98662764	-0.01942239
Q62418	Drebrin-like protein OS=Mus musculus OX=10090 GN=Dbnl PE=1 SV=2 - [DBNL_MOUSE]	Dbnl	49.77059063	17.93955048	12.12312031	25.31147007	37.40137314	16.09097507	26.61108714	26.26793943	20.26646655	10.68734727	1	1	0.987105085	-0.018724416
Q03146	Epithelial discoidin domain-containing receptor 1 OS=Mus musculus OX=10090 GN=Ddr1 PE=2 SV=2 - [DDR1_MOUSE]	Ddr1	149.7179934	121.2279284	143.1045715	150.1363472	146.5905825	112.1171401	138.0168311	136.2813566	14.91089459	21.00178878	1	1	0.987425631	-0.018256
P36536	GTP-binding protein SAR1a OS=Mus musculus OX=10090 GN=Sar1a PE=1 SV=1 - [SAR1A_MOUSE]	Sar1a	53.75581619	0	0	0	53.09997744	0	17.9186054	17.69999248	31.03593495	30.65728627	1	1	0.987799669	-0.01770961
O35188	Fractalkine OS=Mus musculus OX=10090 GN=Cx3cl1 PE=1 SV=3 - [X3CL1_MOUSE]	Cx3cl1	550.7198809	773.4900259	840.8172315	482.3210303	716.0665033	945.7761033	721.6757127	714.7212123	151.8310261	231.7304653	1	1	0.9903634	-0.013970096
Q8BTI8	Serine/arginine repetitive matrix protein 2 OS=Mus musculus OX=10090 GN=Srrm2 PE=1 SV=3 - [SRRM2_MOUSE]	Srrm2	37.24693669	0	0	0	27.78264226	9.260880754	12.41564556	12.34784101	21.50452893	14.14622902	1	1	0.994538781	-0.007900466
Q62348	Translin OS=Mus musculus OX=10090 GN=Tsn PE=1 SV=1 - [TSN_MOUSE]	Tsn	358.762147	108.2637168	183.4573776	356.4695872	185.3132086	105.2889237	216.8277471	215.6905732	128.5400796	128.3160927	1	1	0.994755404	-0.007586264
P03995	Glial fibrillary acidic protein OS=Mus musculus OX=10090 GN=Gfap PE=1 SV=4 - [GFAP_MOUSE]	Gfap	281.1274691	556.6448879	472.5167795	377.7850009	604.4684183	323.0688846	436.7630455	435.1074346	141.1956414	149.2006047	1	1	0.996209361	-0.005479127
Q61554	Fibrillin-1 OS=Mus musculus OX=10090 GN=Fbn1 PE=1 SV=2 - [FBN1_MOUSE]	Fbn1	66.64094347	42.83254814	44.09282883	42.2065744	65.47301612	45.40538365	51.18877348	51.02832472	13.39679981	12.61130157	1	1	0.996865548	-0.00452916
Q8C0E2	Vacuolar protein sorting-associated protein 26B OS=Mus musculus OX=10090 GN=Vps26b PE=1 SV=1 - [VP26B_MOUSE]	Vps26b	50.73456872	33.18158142	21.73831595	50.15651174	55.29226765	0	35.21815536	35.14959313	14.60501262	30.5485584	1	1	0.998053213	-0.002811357
P70122	Ribosome maturation protein SBDS OS=Mus musculus OX=10090 GN=Sbds PE=1 SV=4 - [SBDS_MOUSE]	Sbds	46.63937311	0	0	29.31150906	17.28931545	0	15.5464577	15.53360817	26.92725462	14.73441624	1	1	0.999173475	-0.001192916
Q8BWW4	La-related protein 4 OS=Mus musculus OX=10090 GN=Larp4 PE=1 SV=2 - [LARP4_MOUSE]	Larp4	4.418790962	0	0	4.421104063	0	0	1.472930321	1.473701354	2.551190151	2.552525621	1	1	1.000523469	0.000755009

P10404	MLV-related proviral Env polyprotein OS=Mus musculus OX=10090 PE=1 SV=3 - [ENV1_MOUSE]	Not availa	144.1890847	143.4514467	158.6812898	129.1593422	161.0888231	157.8297619	148.7739404	149.3593091	8.587939555	17.56941567	1	1	1.003934618	0.005665316
Q8BNJ6	Neuropilin and tolloid-like protein 2 OS=Mus musculus OX=10090 GN=Neto2 PE=1 SV=1 - [NETO2_MOUSE]	Neto2	0	21.87460742	0	0	0	22.06011944	7.291535807	7.353373147	12.62931048	12.7364159	1	1	1.008480702	0.012183477
Q91XV3	Brain acid soluble protein 1 OS=Mus musculus OX=10090 GN=Basp1 PE=1 SV=3 - [BASP1_MOUSE]	Basp1	693.5929892	694.1227265	608.7632185	698.0761762	648.1652916	669.5585461	665.4929781	671.933338	49.13012694	25.0400445	1	1	1.009677578	0.013894668
Q5FWK3	Rho GTPase-activating protein 1 OS=Mus musculus OX=10090 GN=Arhgap1 PE=1 SV=1 - [RHG01_MOUSE]	Arhgap1	105.4291785	32.40808066	82.18816722	78.73406694	96.26147625	48.10406261	73.34180881	74.36653527	37.30567955	24.37397427	1	1	1.013971928	0.020017711
P39447	Tight junction protein ZO-1 OS=Mus musculus OX=10090 GN=Tjp1 PE=1 SV=2 - [ZO1_MOUSE]	Tjp1	83.46105378	17.33123928	25.01381402	63.04274964	46.00120079	18.56887017	41.93536903	42.53760687	36.16686826	22.4383344	1	1	1.014361096	0.020571319
P62313	U6 snRNA-associated Sm-like protein LSm6 OS=Mus musculus OX=10090 GN=Lsm6 PE=1 SV=1 - [LSM6_MOUSE]	Lsm6	62.78698795	0	0	63.76888928	0	0	20.92899598	21.25629643	36.2500844	36.81698539	1	1	1.015638612	0.022387148
Q9JHB3	Metalloproteinase inhibitor 4 OS=Mus musculus OX=10090 GN=Timp4 PE=2 SV=1 - [TIMP4_MOUSE]	Timp4	31.8877171	27.9993617	32.97745164	29.755393	26.48764741	38.15461677	30.95484348	31.46588573	2.616875001	6.018627963	1	1	1.016509282	0.023623389
Q02248	Catenin beta-1 OS=Mus musculus OX=10090 GN=Ctnnb1 PE=1 SV=1 - [CTNB1_MOUSE]	Ctnnb1	75.88904028	76.86543496	99.18519872	68.87943155	110.1459049	77.19163727	83.97989132	85.40565792	13.17722909	21.82505573	1	1	1.016977476	0.024287727
Q61207	Prosaposin OS=Mus musculus OX=10090 GN=Psap PE=1 SV=2 - [SAP_MOUSE]	Psap	1073.508683	923.9039067	1071.165874	962.7007656	1068.007964	1091.118707	1022.859488	1040.609145	85.70605264	68.45300099	1	1	1.017352977	0.024820319
Q91VU0	Protein FAM3C OS=Mus musculus OX=10090 GN=Fam3c PE=1 SV=1 - [FAM3C_MOUSE]	Fam3c	63.28365577	66.05848329	92.47547442	57.70966732	84.75210201	84.81209205	73.93920449	75.75795379	16.1127246	15.63030336	1	1	1.024597902	0.035057843
Q9D8Y0	EF-hand domain-containing protein D2 OS=Mus musculus OX=10090 GN=Efhd2 PE=1 SV=1 - [EFHD2_MOUSE]	Efhd2	56.31713704	70.23566653	64.81714825	68.44098168	59.87324231	67.89127314	63.78998394	65.40183238	7.015886663	4.795782102	1	1	1.025268049	0.036001141
P47877	Insulin-like growth factor-binding protein 2 OS=Mus musculus OX=10090 GN=Igfbp2 PE=2 SV=2 - [IBP2_MOUSE]	Igfbp2	7001.734731	9400.708791	9814.343436	6244.610976	9124.397384	11528.05516	8738.928986	8965.687839	1518.60341	2645.295285	1	1	1.025948129	0.036957791
Q921I1	Serotransferrin OS=Mus musculus OX=10090 GN=Tf PE=1 SV=1 - [TRFE_MOUSE]	Tf	27362.02404	48645.59814	71433.15682	48498.43962	47479.93136	55398.47234	49146.92633	50458.94777	22039.84309	4307.959723	1	1	1.0266959	0.038008929
Q8JZM4	Delta and Notch-like epidermal growth factor-related receptor OS=Mus musculus OX=10090 GN=Dner PE=1 SV=1 - [DNER_MOUSE]	Dner	7.576797198	18.21346793	29.23586345	15.87472479	21.41796374	19.30972421	18.34204286	18.86747091	10.83010556	2.797957379	1	1	1.028646103	0.040746721
P97821	Dipeptidyl peptidase 1 OS=Mus musculus OX=10090 GN=Ctsc PE=1 SV=1 - [CATC_MOUSE]	Ctsc	130.2131867	134.1724433	121.7294809	76.05175685	125.9298634	195.7735873	128.705037	132.5850692	6.357099561	60.13774188	1	1	1.0301467	0.042849802
Q9Z2L6	Multiple inositol polyphosphate phosphatase 1 OS=Mus musculus OX=10090 GN=Minpp1 PE=1 SV=3 - [MINP1_MOUSE]	Minpp1	42.09091312	79.71800835	95.51318229	51.91014294	86.14405389	86.56009225	72.44070126	74.87142969	27.4445643	19.88614566	1	1	1.033554734	0.047614791
Q99NB1	Acetyl-coenzyme A synthetase 2-like, mitochondrial OS=Mus musculus OX=10090 GN=Acss1 PE=1 SV=1 - [ACS2L_MOUSE]	Acss1	45.65975763	0	0	40.03844283	7.206057806	0	15.21991921	15.74816688	26.36167336	21.34232758	1	1	1.034707653	0.049223205
Q00993	Tyrosine-protein kinase receptor UFO OS=Mus musculus OX=10090 GN=Axl PE=1 SV=2 - [UFO_MOUSE]	Axl	54.96399805	65.10236944	38.21180515	61.51092986	51.82065524	50.85246871	52.75939088	54.72801794	13.58016316	5.894087452	1	1	1.037313301	0.0528517
Q80UW8	DNA-directed RNA polymerases I, II, and III subunit RPABC1 OS=Mus musculus OX=10090 GN=Polr2e PE=1 SV=1 - [RPAB1_MOUSE]	Polr2e	19.42785423	13.44481672	9.749362289	16.0800292	15.6602455	12.48493422	14.20734441	14.74173631	4.884095406	1.965685969	1	1	1.037613778	0.053269541
Q9D0T1	NHP2-like protein 1 OS=Mus musculus OX=10090 GN=Snu13 PE=1 SV=4 - [NH2L1_MOUSE]	Snu13	78.56245258	0	0	0	81.52739143	0	26.18748419	27.17579714	45.35805314	47.06986139	1	1	1.037739897	0.053444886
P50429	Arylsulfatase B OS=Mus musculus OX=10090 GN=Arsb PE=1 SV=3 - [ARSB_MOUSE]	Arsb	34.16986945	40.00463119	47.25497771	33.27300613	53.82600153	39.05436896	40.47649278	42.05112554	6.555303545	10.5991423	1	1	1.038902401	0.055060128
O88456	Calpain small subunit 1 OS=Mus musculus OX=10090 GN=Capns1 PE=1 SV=1 - [CPNS1_MOUSE]	Capns1	0	0	26.65183353	0	27.71568997	0	8.883944511	9.238563322	15.38744326	16.00166106	1	1	1.03991682	0.056468135
P97765	WW domain-binding protein 2 OS=Mus musculus OX=10090 GN=Wbp2 PE=1 SV=1 - [WBP2_MOUSE]	Wbp2	101.0998087	75.34937087	110.3080158	83.85408481	125.8835139	88.94896848	95.58573176	99.56218907	18.11989228	22.93683862	1	1	1.041600951	0.05880267
Q9EP78	Carbohydrate sulfotransferase 7 OS=Mus musculus OX=10090 GN=Chst7 PE=2 SV=1 - [CHST7_MOUSE]	Chst7	6.050532561	0	0	0	6.303831022	0	2.016844187	2.101277007	3.493276603	3.639518538	1	1	1.041863829	0.059166731
P56812	Programmed cell death protein 5 OS=Mus musculus OX=10090 GN=Pdcd5 PE=1 SV=3 - [PDCD5_MOUSE]	Pdcd5	85.20902982	52.67339548	52.47234425	132.4642513	0	66.77071237	63.45158985	66.41165455	18.84276389	66.23285559	1	1	1.046650757	0.065780129
Q61147	Ceruloplasmin OS=Mus musculus OX=10090 GN=Cp PE=1 SV=2 - [CERU_MOUSE]	Cp	260.8260633	103.2153689	123.7041629	250.169442	148.44546	112.6978488	162.5818651	170.4375836	85.69649663	71.32566062	1	1	1.048318541	0.06807716
P08122	Collagen alpha-2(IV) chain OS=Mus musculus OX=10090 GN=Col4a2 PE=1 SV=4 - [CO4A2_MOUSE]	Col4a2	9.931118858	0	44.22353703	0	49.09702633	7.883154021	18.05155196	18.99339345	23.20315458	26.36678903	1	1	1.052175098	0.073374811
Q6P5F7	Protein tweety homolog 3 OS=Mus musculus OX=10090 GN=Ttyh3 PE=1 SV=1 - [TTYH3_MOUSE]	Ttyh3	49.12348575	31.03777509	54.97107599	30.00483955	59.93826547	52.2925018	45.04411228	47.41186894	12.47724685	15.55210371	1	1	1.052565286	0.073909721
Q6W8Q3	Purkinje cell protein 4-like protein 1 OS=Mus musculus OX=10090 GN=Pcp4l1 PE=1 SV=1 - [PC4L1_MOUSE]	Pcp4l1	38.29341467	0	0	0	40.45905764	0	12.76447156	13.48635255	22.10871327	23.35904782	1	1	1.056553927	0.079366405
P09055	Integrin beta-1 OS=Mus musculus OX=10090 GN=Itgb1 PE=1 SV=1 - [ITB1_MOUSE]	Itgb1	94.52213217	144.2086056	131.9717306	106.4584724	128.8457803	156.4644821	123.5674895	130.5895783	25.88744603	25.04857032	1	1	1.056827964	0.079740546
Q920Q6	RNA-binding protein Musashi homolog 2 OS=Mus musculus OX=10090 GN=Msi2 PE=1 SV=1 - [MSI2H_MOUSE]	Msi2	42.34749996	0	30.56421762	39.76096649	37.48421079	0	24.30390586	25.74839242	21.85683682	22.3278008	1	1	1.059434338	0.083294175
P38647	Stress-70 protein, mitochondrial OS=Mus musculus OX=10090 GN=Hspa9 PE=1 SV=3 - [GRP75_MOUSE]	Hspa9	206.0176007	11.03313238	85.41972219	129.7974763	120.9835463	70.67207996	100.8234851	107.1510342	98.40067408	31.89760048	1	1	1.062758682	0.087814045
Q61292	Laminin subunit beta-2 OS=Mus musculus OX=10090 GN=Lamb2 PE=1 SV=2 - [LAMB2_MOUSE]	Lamb2	206.2058856	114.4191989	105.5159057	25.62626619	246.7056386	180.771321	142.0469967	151.0344086	55.74127217	113.4999348	1	1	1.063270693	0.088508932
Q8C129	Leucyl-cystinyl aminopeptidase OS=Mus musculus OX=10090 GN=Lnpep PE=1 SV=1 - [LCAP_MOUSE]	Lnpep	17.39291128	13.84908238	12.72697929	14.22249759	22.87916281	9.667500891	14.65632432	15.58972043	2.435458885	6.711108431	1	1	1.063685553	0.089071723
Q3U962	Collagen alpha-2(V) chain OS=Mus musculus OX=10090 GN=Col5a2 PE=1 SV=1 - [CO5A2_MOUSE]	Col5a2	148.6104594	107.6789073	114.3017595	145.5889496	140.9969162	108.8187652	123.5303754	131.8015437	21.97096953	20.03566252	1	1	1.066956554	0.093501432
Q61768	Kinesin-1 heavy chain OS=Mus musculus OX=10090 GN=Kif5b PE=1 SV=3 - [KINH_MOUSE]	Kif5b	47.89348193	0	0	22.54130016	28.56007708	0	15.96449398	17.03379241	27.65131468	15.05552994	1	1	1.066979789	0.093532848
Q91X97	Neurocalcin-delta OS=Mus musculus OX=10090 GN=Ncald PE=1 SV=4 - [NCALD_MOUSE]	Ncald	85.85218673	94.64927446	29.67122562	75.38300022	87.50640482	61.5528296	70.05756227	74.81407821	35.25108976	12.98613764	1	1	1.067894397	0.094768987
P13595	Neural cell adhesion molecule 1 OS=Mus musculus OX=10090 GN=Ncam1 PE=1 SV=3 - [NCAM1_MOUSE]	Ncam1	575.2575684	623.7529122	412.0376235	495.6165917	616.0473584	608.8338594	537.0160347	573.4992699	110.9173207	67.54474298	1	1	1.067936957	0.094826483
Q9WTX5	S-phase kinase-associated protein 1 OS=Mus musculus OX=10090 GN=Skp1 PE=1 SV=3 - [SKP1_MOUSE]	Skp1	116.6408474	70.64187745	42.64811942	98.57851424	93.79862629	53.78304974	76.64361477	82.05339676	37.35969203	24.59921183	1	1	1.070583597	0.098397453
Q5I012	Putative sodium-coupled neutral amino acid transporter 10 OS=Mus musculus OX=10090 GN=Slc38a10 PE=1 SV=2 - [S38AA_MOUSE]	Slc38a10	0	0	26.19820927	0	0	28.06619728	8.732736423	9.355399092	15.12554317	16.20402655	1	1	1.071302126	0.099365402
P84228	Histone H3.2 OS=Mus musculus OX=10090 GN=H3c2 PE=1 SV=2 - [H32_MOUSE]	H3c2	1683.039312	0	813.675422	929.7555083	958.7253914	788.954537	832.2382448	892.4784789	841.673194	90.81695021	1	1	1.0723834	0.100820793
Q9WTR5	Cadherin-13 OS=Mus musculus OX=10090 GN=Cdh13 PE=1 SV=2 - [CAD13_MOUSE]	Cdh13	264.2308393	375.2782005	424.0485823	254.4129798	405.591339	480.5952481	354.5192074	380.1998556	81.90622436	115.2091585	1	1	1.07243796	0.100894191
Q9ERR7	Selenoprotein F OS=Mus musculus OX=10090 GN=Selenof PE=1 SV=3 - [SEP15_MOUSE]	Selenof	0	31.42242112	61.28566985	0	42.19144666	57.30544189	30.90269699	33.16562952	30.64614033	29.6997887	1	1	1.073227671	0.101956157
P57776	Elongation factor 1-delta OS=Mus musculus OX=10090 GN=Eef1d PE=1 SV=3 - [EF1D_MOUSE]	Eef1d	162.4435688	79.63755737	111.0794495	126.5007294	173.7506886	79.19803206	117.7201919	126.48315	41.80052007	47.27633071	1	1	1.074438871	0.103583404
Q9CQI7	U2 small nuclear ribonucleoprotein B'' OS=Mus musculus OX=10090 GN=Snrpb2 PE=1 SV=1 - [RU2B_MOUSE]	Snrpb2	98.83427829	77.78042894	0	91.30611971	83.44666144	15.0799022	58.87156907	63.27756112	52.05970013	41.92497483	1	1	1.074840744	0.104122916
Q9D8S4	Oligoribonuclease, mitochondrial OS=Mus musculus OX=10090 GN=Rexo2 PE=1 SV=2 - [ORN_MOUSE]	Rexo2	92.74494264	34.5096714	60.90807727	92.62780628	55.39570192	54.31189119	62.72089711	67.44513313	29.15992868	21.81556627	1	1	1.075321563	0.104768147
O88653	Ragulator complex protein LAMTOR3 OS=Mus musculus OX=10090 GN=Lamtor3 PE=1 SV=1 - [LTOR3_MOUSE]	Lamtor3	37.60163424	27.0775079	27.48396245	32.65062052	40.00448764	26.8065671	30.72103486	33.15389175	5.962238437	6.613337892	1	1	1.079191892	0.109951415
P61979	Heterogeneous nuclear ribonucleoprotein K OS=Mus musculus OX=10090 GN=Hnrnpk PE=1 SV=1 - [HNRPK_MOUSE]	Hnrnpk	1554.017785	852.0389798	883.2872358	1230.424314	1457.5106	868.767388	1096.448	1185.567434	396.5749544	296.9238069	1	1	1.081280128	0.112740331
Q64514	Tripeptidyl-peptidase 2 OS=Mus musculus OX=10090 GN=Tpp2 PE=1 SV=3 - [TPP2_MOUSE]	Tpp2	87.07047927	43.83798716	46.11066407	74.13896349	81.22260929	36.86219618	59.00637684	64.07458965	24.33077576	23.8312878	1	1	1.085892629	0.118881459
Q8BFY9	Transportin-1 OS=Mus musculus OX=10090 GN=Tnpo1 PE=1 SV=2 - [TNPO1_MOUSE]	Tnpo1	47.87015725	0	0	0	51.99717776	0	15.95671908	17.33239259	27.63784817	30.02058457	1	1	1.086212804	0.119306775
Q9WVB2	Transducin-like enhancer protein 2 OS=Mus musculus OX=10090 GN=Tle2 PE=2 SV=1 - [TLE2_MOUSE]	Tle2	71.30643973	0	0	0	77.67369735	0	23.76881324	25.89123245	41.16879217	44.84493007	1	1	1.089294286	0.123393768
Q8BFQ8	Glutamine amidotransferase-like class 1 domain-containing protein 1 OS=Mus musculus OX=10090 GN=Gatd1 PE=1 SV=1 - [GALD1_MOUSE]	Gatd1	46.6819117	0	0	0	50.93922597	0	15.56063723	16.97974199	26.95181429	29.40977583	1	1	1.09119837	0.125913395
O35685	Nuclear migration protein nudC OS=Mus musculus OX=10090 GN=Nudc PE=1 SV=1 - [NUDC_MOUSE]	Nudc	67.84442741	38.4820641	0	40.55941128	41.98609148	33.53966653	35.44216384	38.69505643	34.02421679	4.521325895	1	1	1.091780305	0.126682577
P70428	Exostosin-2 OS=Mus musculus OX=10090 GN=Ext2 PE=1 SV=2 - [EXT2_MOUSE]	Ext2	39.7012454	32.1379058	45.92606821	31.72556793	51.44189993	45.5075786	39.25507313	42.89168215	6.904901008	10.11511852	1	1	1.092640485	0.127818785
P58771	Tropomyosin alpha-1 chain OS=Mus musculus OX=10090 GN=Tpm1 PE=1 SV=1 - [TPM1_MOUSE]	Tpm1	373.7682219	239.706721	0	349.4161512	321.5919857	0	204.4916476	223.669379	189.3561379	194.2023155	1	1	1.093782468	0.129325842
Q6IRU5	Clathrin light chain B OS=Mus musculus OX=10090 GN=Cltb PE=1 SV=1 - [CLCB_MOUSE]	Cltb	207.5848202	126.7485825	92.11265979	167.661504	186.5198786	112.9615865	142.1486875	155.7143231	59.25645434	38.20676555	1	1	1.095432718	0.131500876
P12023	Amyloid-beta A4 protein OS=Mus musculus OX=10090 GN=App PE=1 SV=3 - [A4_MOUSE]	App	42.5253887	22.61657474	39.36477583	28.71986785	47.14643574	39.01602175	34.83557976	38.29410845	10.69931919	9.234471842	1	1	1.099281502	0.136560877
P21107	Tropomyosin alpha-3 chain OS=Mus musculus OX=10090 GN=Tpm3 PE=1 SV=3 - [TPM3_MOUSE]	Tpm3	519.7527805	341.4341618	207.0964163	479.2063473	461.8607424	233.8870984	356.0944529	391.651396	156.8428947	136.9028769	1	1	1.099852561	0.137310139
O70492	Sorting nexin-3 OS=Mus musculus OX=10090 GN=Snx3 PE=1 SV=3 - [SNX3_MOUSE]	Snx3	82.26565162	0	0	0	90.53455569	0	27.42188387	30.17818523	47.49609611	52.2701501	1	1	1.100514661	0.138178365
Q8C8R3	Ankyrin-2 OS=Mus musculus OX=10090 GN=Ank2 PE=1 SV=2 - [ANK2_MOUSE]	Ank2	60.3898319	0	0	36.60262891	29.89433923	0	20.12994397	22.16565604	34.86608571	19.48685502	1	1	1.101128552	0.138982907
Q9QXS6	Drebrin OS=Mus musculus OX=10090 GN=Dbn1 PE=1 SV=4 - [DREB_MOUSE]	Dbn1	33.73296426	24.55008909	30.49931839	30.43842665	67.54782067	0	29.59412391	32.66208244	4.657878475	33.82876739	1	1	1.103667827	0.142306027
Q8VEE4	Replication protein A 70 kDa DNA-binding subunit OS=Mus musculus OX=10090 GN=Rpa1 PE=1 SV=1 - [RFA1_MOUSE]	Rpa1	35.19129596	0	0	5.641641885	33.25232525	0	11.73043199	12.96465571	20.3177042	17.79463915	1	1	1.105215539	0.144327751
Q6PIP5	NudC domain-containing protein 1 OS=Mus musculus OX=10090 GN=Nudcd1 PE=1 SV=2 - [NUDC1_MOUSE]	Nudcd1	21.34564985	0	0	0	23.60200273	0	7.115216617	7.867334242	12.32391669	13.62662263	1	1	1.105705513	0.144967199
Q08761	Vitamin K-dependent protein S OS=Mus musculus OX=10090 GN=Pros1 PE=2 SV=1 - [PROS_MOUSE]	Pros1	103.6542367	113.8726773	112.9124477	73.96074977	183.5841509	108.3412563	110.1464539	121.9620523	5.642886952	56.06663076	1	1	1.10727171	0.147009283
P50431	Serine hydroxymethyltransferase, cytosolic OS=Mus musculus OX=10090 GN=Shmt1 PE=1 SV=3 - [GLYC_MOUSE]	Shmt1	60.41832176	0	0	34.23621151	32.74901027	0	20.13944059	22.32840726	34.88253433	19.35126017	1	1	1.10869054	0.148856734
O08665	Semaphorin-3A OS=Mus musculus OX=10090 GN=Sema3a PE=1 SV=2 - [SEM3A_MOUSE]	Sema3a	0	0	23.47662063	0	0	26.28442845	7.825540211	8.761476149	13.55423324	15.17532184	1	1	1.11960017	0.162983611
P63166	Small ubiquitin-related modifier 1 OS=Mus musculus OX=10090 GN=Sumo1 PE=1 SV=1 - [SUMO1_MOUSE]	Sumo1	172.8160396	0	0	135.5792934	0	58.07089486	57.60534653	64.55006277	99.77538698	68.02147393	1	1	1.1205568	0.16421578
Q6P5H2	Nestin OS=Mus musculus OX=10090 GN=Nes PE=1 SV=1 - [NEST_MOUSE]	Nes	320.7856652	188.5397512	217.018863	332.0232167	304.3558884	180.6906673	242.1147598	272.3565908	69.60314987	80.58133414	1	1	1.124907011	0.169805748
Q91VW3	SH3 domain-binding glutamic acid-rich-like protein 3 OS=Mus musculus OX=10090 GN=Sh3bgrl3 PE=1 SV=1 - [SH3L3_MOUSE]	Sh3bgrl3	138.4654939	89.82392436	90.94913431	124.3610141	149.0297736	88.01311482	106.4128509	120.4679675	27.76410398	30.69405514	1	1	1.132081008	0.178977197
Q99L04	Dehydrogenase/reductase SDR family member 1 OS=Mus musculus OX=10090 GN=Dhrs1 PE=1 SV=1 - [DHRS1_MOUSE]	Dhrs1	34.43389667	12.94936281	0	0	53.6568582	0	15.79441983	17.8856194	17.39235672	30.97880153	1	1	1.132401164	0.179385137
P70663	SPARC-like protein 1 OS=Mus musculus OX=10090 GN=Sparcl1 PE=1 SV=3 - [SPRL1_MOUSE]	Sparcl1	1128.181562	1018.256466	1000.544546	959.8880526	1492.523069	1111.732372	1048.994191	1188.047831	69.1477244	274.3957948	1	1	1.132559018	0.179586232
B2RXS4	Plexin-B2 OS=Mus musculus OX=10090 GN=Plxnb2 PE=1 SV=1 - [PLXB2_MOUSE]	Plxnb2	68.38560046	73.98515974	59.25597029	39.78944499	65.89510927	122.7183698	67.20891016	76.13430803	7.434763313	42.40203459	1	1	1.132800812	0.179894205
Q91X72	Hemopexin OS=Mus musculus OX=10090 GN=Hpx PE=1 SV=2 - [HEMO_MOUSE]	Hpx	68.07945856	123.0187048	0	59.42471198	63.61226676	94.727173	63.69938779	72.58805058	61.62620545	19.28702828	1	1	1.139540788	0.188452565
Q01065	Calcium/calmodulin-dependent 3',5'-cyclic nucleotide phosphodiesterase 1B OS=Mus musculus OX=10090 GN=Pde1b PE=1 SV=2 - [PDE1B_MOUSE]	Pde1b	11.12269597	0	0	12.70166244	0	0	3.707565322	4.233887481	6.42169151	7.33330823	1	1	1.141958971	0.191510818
Q9CQF3	Cleavage and polyadenylation specificity factor subunit 5 OS=Mus musculus OX=10090 GN=Nudt21 PE=1 SV=1 - [CPSF5_MOUSE]	Nudt21	186.2832843	74.02443289	82.86886988	181.8611972	196.2547356	14.10785124	114.3921957	130.7412613	62.41636388	101.2635559	1	1	1.142921163	0.192725892
Q9CR57	60S ribosomal protein L14 OS=Mus musculus OX=10090 GN=Rpl14 PE=1 SV=3 - [RL14_MOUSE]	Rpl14	249.8371253	155.328262	161.4719684	247.4415873	270.3907806	133.5975188	188.8791186	217.1432956	52.88048052	73.25700633	1	1	1.149641619	0.201184195
Q9D1L9	Ragulator complex protein LAMTOR5 OS=Mus musculus OX=10090 GN=Lamtor5 PE=1 SV=1 - [LTOR5_MOUSE]	Lamtor5	73.536055	91.89060457	62.71850942	61.41428873	114.9491773	87.42311181	76.04838966	87.92885929	14.74742869	26.77102744	1	1	1.156222501	0.209419054
Q99PU5	Long-chain-fatty-acid--CoA ligase ACSBG1 OS=Mus musculus OX=10090 GN=Acsbg1 PE=1 SV=1 - [ACBG1_MOUSE]	Acsbg1	28.26469725	0	0	0	32.74663728	0	9.421565749	10.91554576	16.31863056	18.90627985	1	1	1.158570247	0.212345521
Q91X20	Set1/Ash2 histone methyltransferase complex subunit ASH2 OS=Mus musculus OX=10090 GN=Ash2l PE=1 SV=1 - [ASH2L_MOUSE]	Ash2l	18.10513234	0	0	21.06795715	0	0	6.035044115	7.022652382	10.45300303	12.16359073	1	1	1.163645575	0.218651707
Q8CGM1	Adhesion G protein-coupled receptor B2 OS=Mus musculus OX=10090 GN=Adgrb2 PE=1 SV=2 - [AGRB2_MOUSE]	Adgrb2	0	36.59157829	0	0	42.86025383	0	12.19719276	14.28675128	21.12615757	24.74537909	1	1	1.171314708	0.22812875
Q99LN9	Deoxyhypusine hydroxylase OS=Mus musculus OX=10090 GN=Dohh PE=1 SV=2 - [DOHH_MOUSE]	Dohh	5.186769313	0	0	0	6.076883258	0	1.728923104	2.025627753	2.994582659	3.508490185	1	1	1.171612403	0.228495371
Q9WVG6	Histone-arginine methyltransferase CARM1 OS=Mus musculus OX=10090 GN=Carm1 PE=1 SV=2 - [CARM1_MOUSE]	Carm1	58.23944126	31.56720444	36.46217205	56.7030848	56.92578654	34.78648731	42.08960592	49.47178622	14.19869935	12.71832937	1	1	1.175392003	0.233141988
Q9JL35	High mobility group nucleosome-binding domain-containing protein 5 OS=Mus musculus OX=10090 GN=Hmgn5 PE=1 SV=2 - [HMGN5_MOUSE]	Hmgn5	50.27332575	0	0	31.29271698	27.81377194	0	16.75777525	19.70216297	29.02531815	17.15101117	1	1	1.175702782	0.233523392
Q9CX00	IST1 homolog OS=Mus musculus OX=10090 GN=Ist1 PE=1 SV=1 - [IST1_MOUSE]	Ist1	35.31808577	16.67238875	25.58106375	32.01337955	34.47876092	24.83812222	25.85717942	30.4434209	9.325914659	5.008398575	1	1	1.177368204	0.235565572
P28862	Stromelysin-1 OS=Mus musculus OX=10090 GN=Mmp3 PE=2 SV=2 - [MMP3_MOUSE]	Mmp3	0	21.09987701	0	0	24.91539613	0	7.033292336	8.305132042	12.18201967	14.38491066	1	1	1.180831344	0.239802922
P15379	CD44 antigen OS=Mus musculus OX=10090 GN=Cd44 PE=1 SV=3 - [CD44_MOUSE]	Cd44	936.4641689	1000.446945	847.6749425	1487.118676	879.9360496	921.5597736	928.195352	1096.204833	76.72093045	339.1804212	1	1	1.181006596	0.240017022
O08992	Syntenin-1 OS=Mus musculus OX=10090 GN=Sdcbp PE=1 SV=1 - [SDCB1_MOUSE]	Sdcbp	170.0425467	147.0426905	311.1938522	138.4416449	320.5792528	296.7622802	209.4263631	251.927726	88.88033933	99.00065652	1	1	1.202941799	0.266566843
Q68FL6	Methionine--tRNA ligase, cytoplasmic OS=Mus musculus OX=10090 GN=Mars1 PE=1 SV=1 - [SYMC_MOUSE]	Mars1	18.00382734	0	0	0	21.67018925	0	6.00127578	7.223396417	10.39451456	12.5112896	1	1	1.203643472	0.267408119
O88487	Cytoplasmic dynein 1 intermediate chain 2 OS=Mus musculus OX=10090 GN=Dync1i2 PE=1 SV=1 - [DC1I2_MOUSE]	Dync1i2	68.78256158	50.81935621	0	63.88226577	45.93018763	34.37646072	39.86730593	48.06297137	35.67521196	14.8680768	1	1	1.205573596	0.269719725
Q810C0	SLIT and NTRK-like protein 2 OS=Mus musculus OX=10090 GN=Slitrk2 PE=1 SV=1 - [SLIK2_MOUSE]	Slitrk2	10.84443742	17.91284187	17.77300807	13.84609804	26.58982865	15.70615741	15.51009579	18.71402803	4.041183534	6.883758546	1	1	1.206570758	0.270912523
Q99JT2	Serine/threonine-protein kinase 26 OS=Mus musculus OX=10090 GN=Stk26 PE=1 SV=1 - [STK26_MOUSE]	Stk26	9.045882893	0	0	3.826763243	7.102242171	0	3.015294298	3.643001804	5.222642923	3.554685241	1	1	1.208174541	0.272828891
G3XA57	Rab11 family-interacting protein 2 OS=Mus musculus OX=10090 GN=Rab11fip2 PE=1 SV=1 - [RFIP2_MOUSE]	Rab11fip2	0	109.1597942	0	0	132.0092006	0	36.38659807	44.00306688	63.02343658	76.21554752	1	1	1.209320717	0.274196904
Q99KB8	Hydroxyacylglutathione hydrolase, mitochondrial OS=Mus musculus OX=10090 GN=Hagh PE=1 SV=2 - [GLO2_MOUSE]	Hagh	58.48479905	13.92902811	26.26546516	44.70593203	53.41050835	21.62976049	32.89309744	39.91540029	23.00539748	16.42302894	1	1	1.213488646	0.27916061
Q9CXU9	Eukaryotic translation initiation factor 1b OS=Mus musculus OX=10090 GN=Eif1b PE=1 SV=2 - [EIF1B_MOUSE]	Eif1b	67.75346087	0	0	0	82.59590883	0	22.58448696	27.53196961	39.11747887	47.6867702	1	1	1.219065532	0.285775682
P62880	Guanine nucleotide-binding protein G(I)/G(S)/G(T) subunit beta-2 OS=Mus musculus OX=10090 GN=Gnb2 PE=1 SV=3 - [GBB2_MOUSE]	Gnb2	251.622459	179.1509099	189.8311418	138.8530489	236.0728456	382.3211607	206.8681702	252.4156851	39.12450139	122.5540561	1	1	1.220176525	0.28708988

Q9Z130	Heterogeneous nuclear ribonucleoprotein D-like OS=Mus musculus OX=10090 GN=Hnrnpdl PE=1 SV=1 - [HNRDL_MOUSE]	Hnrnpdl	597.3457284	0	0	733.3528118	0	0	199.1152428	244.4509373	344.8777171	423.4014433	1	1	1.227685705	0.295941269
Q61738	Integrin alpha-7 OS=Mus musculus OX=10090 GN=Itga7 PE=1 SV=3 - [ITA7_MOUSE]	Itga7	30.6883312	45.42961404	91.63451575	26.95928793	75.82002542	103.3833037	55.91748699	68.72087235	31.79789242	38.70343689	1	1	1.228969255	0.297448825
Q6KAU4	Multivesicular body subunit 12B OS=Mus musculus OX=10090 GN=Mvb12b PE=1 SV=2 - [MB12B_MOUSE]	Mvb12b	18.3982333	0	0	0	22.63047104	0	6.132744433	7.543490346	10.62222495	13.06570855	1	1	1.230035008	0.298699376
Q9R118	Serine protease HTRA1 OS=Mus musculus OX=10090 GN=Htra1 PE=1 SV=2 - [HTRA1_MOUSE]	Htra1	112.8713735	56.2465547	99.88601551	90.21703972	148.6872377	94.59169402	89.66798123	111.1653238	29.66308639	32.56846503	1	1	1.2397438	0.310042011
Q9WUA3	ATP-dependent 6-phosphofructokinase, platelet type OS=Mus musculus OX=10090 GN=Pfkp PE=1 SV=1 - [PFKAP_MOUSE]	Pfkp	16.50053442	0	0	14.86921648	0	5.593173211	5.500178139	6.820796564	9.526587987	7.510239278	1	1	1.240104664	0.310461888
P62911	60S ribosomal protein L32 OS=Mus musculus OX=10090 GN=Rpl32 PE=1 SV=2 - [RL32_MOUSE]	Rpl32	60.40403992	0	0	27.36917823	48.42952659	0	20.13467997	25.26623494	34.87428871	24.2831534	1	1	1.254861511	0.327528155
P01027	Complement C3 OS=Mus musculus OX=10090 GN=C3 PE=1 SV=3 - [CO3_MOUSE]	C3	0	42.35354343	0	53.36806239	0	0	14.11784781	17.78935413	24.4528297	30.81206519	1	1	1.260061333	0.333493958
Q3THS6	S-adenosylmethionine synthase isoform type-2 OS=Mus musculus OX=10090 GN=Mat2a PE=1 SV=2 - [METK2_MOUSE]	Mat2a	114.8595525	0	0	79.8374063	66.38642057	0	38.28651749	48.74127562	66.31419354	42.74361024	1	1	1.273066312	0.348307569
Q00915	Retinol-binding protein 1 OS=Mus musculus OX=10090 GN=Rbp1 PE=1 SV=2 - [RET1_MOUSE]	Rbp1	110.3393181	0	0	105.4352992	35.14509975	0	36.7797727	46.860133	63.70443501	53.68502664	1	1	1.27407348	0.349448485
Q9D6R2	Isocitrate dehydrogenase [NAD] subunit alpha, mitochondrial OS=Mus musculus OX=10090 GN=Idh3a PE=1 SV=1 - [IDH3A_MOUSE]	Idh3a	50.86331712	0	0	38.17618986	27.04344938	0	16.95443904	21.73987975	29.36594983	19.63291258	1	1	1.282252966	0.358680909
Q9JHJ0	Tropomodulin-3 OS=Mus musculus OX=10090 GN=Tmod3 PE=1 SV=1 - [TMOD3_MOUSE]	Tmod3	103.5066491	0	0	60.92718442	72.1921476	0	34.50221636	44.37311067	59.75959171	38.83882802	1	1	1.286094499	0.362996653
P97311	DNA replication licensing factor MCM6 OS=Mus musculus OX=10090 GN=Mcm6 PE=1 SV=1 - [MCM6_MOUSE]	Mcm6	26.89512597	0	0	0	34.60388472	0	8.965041989	11.53462824	15.52790822	19.97856216	1	1	1.286622891	0.363589261
Q60876	Eukaryotic translation initiation factor 4E-binding protein 1 OS=Mus musculus OX=10090 GN=Eif4ebp1 PE=1 SV=3 - [4EBP1_MOUSE]	Eif4ebp1	78.09511139	0	0	0	63.03769033	37.49947228	26.0317038	33.51238753	45.08823359	31.70741607	1	1	1.287368195	0.364424732
Q9D0D5	General transcription factor IIE subunit 1 OS=Mus musculus OX=10090 GN=Gtf2e1 PE=1 SV=1 - [T2EA_MOUSE]	Gtf2e1	3.547009661	0	0	2.382274404	2.249253557	0	1.182336554	1.543842654	2.047866982	1.338660241	1	1	1.305755666	0.384884964
Q91WK5	Glycine cleavage system H protein, mitochondrial OS=Mus musculus OX=10090 GN=Gcsh PE=1 SV=2 - [GCSH_MOUSE]	Gcsh	0	157.976123	112.9792422	131.0407943	125.1722331	97.86758412	90.31845506	118.0268705	81.38947914	17.70332237	1	1	1.306785755	0.386022633
Q01853	Transitional endoplasmic reticulum ATPase OS=Mus musculus OX=10090 GN=Vcp PE=1 SV=4 - [TERA_MOUSE]	Vcp	1125.675607	521.0932457	359.9680993	999.3779837	1102.676616	520.4210968	668.9123175	874.1585656	403.6890579	310.6691158	1	1	1.306835803	0.386077886
Q8CHP8	Glycerol-3-phosphate phosphatase OS=Mus musculus OX=10090 GN=Pgp PE=1 SV=1 - [PGP_MOUSE]	Pgp	122.0805799	29.97302526	51.17072895	117.6029327	106.8711166	42.04558697	67.74144471	88.83987877	48.23786762	40.87875094	1	1	1.311455331	0.391168669
Q8R3G1	Nuclear inhibitor of protein phosphatase 1 OS=Mus musculus OX=10090 GN=Ppp1r8 PE=1 SV=1 - [PP1R8_MOUSE]	Ppp1r8	11.36295616	0	0	14.9519669	0	0	3.787652052	4.983988967	6.560405795	8.632522116	1	1	1.315851852	0.395997069
Q9D8U8	Sorting nexin-5 OS=Mus musculus OX=10090 GN=Snx5 PE=1 SV=1 - [SNX5_MOUSE]	Snx5	36.25627692	0	0	47.78058098	0	0	12.08542564	15.92686033	20.93257124	27.58613129	1	1	1.317856797	0.398193611
Q9JLV5	Cullin-3 OS=Mus musculus OX=10090 GN=Cul3 PE=1 SV=1 - [CUL3_MOUSE]	Cul3	28.93613828	0	0	18.44789975	19.80361802	0	9.645379428	12.75050593	16.70628723	11.06304859	1	1	1.321928911	0.402644596
O89051	Integral membrane protein 2B OS=Mus musculus OX=10090 GN=Itm2b PE=1 SV=1 - [ITM2B_MOUSE]	Itm2b	72.05865128	0	0	0	35.23675416	60.15854318	24.01955043	31.79843245	41.60308172	30.22629851	1	1	1.323856271	0.404746499
Q61686	Chromobox protein homolog 5 OS=Mus musculus OX=10090 GN=Cbx5 PE=1 SV=1 - [CBX5_MOUSE]	Cbx5	74.36140465	0	0	32.42361416	67.03690219	0	24.78713488	33.15350545	42.93257699	33.5244108	1	1	1.337528746	0.419569899
Q99NH0	Ankyrin repeat domain-containing protein 17 OS=Mus musculus OX=10090 GN=Ankrd17 PE=1 SV=2 - [ANR17_MOUSE]	Ankrd17	18.52949313	0	0	11.15042132	13.65962028	0	6.176497709	8.270013867	10.69800785	7.271098052	1	1	1.338948747	0.421100737
Q61490	CD166 antigen OS=Mus musculus OX=10090 GN=Alcam PE=1 SV=3 - [CD166_MOUSE]	Alcam	25.971028	0	31.91308278	25.28204392	32.06512439	20.24324535	19.2947036	25.86347122	16.97177639	5.932347706	1	1	1.340444081	0.422711036
Q9EPC1	Alpha-parvin OS=Mus musculus OX=10090 GN=Parva PE=1 SV=1 - [PARVA_MOUSE]	Parva	48.85229268	0	0	48.15219056	17.6086473	0	16.28409756	21.92027929	28.20488433	24.36392809	1	1	1.346115694	0.42880241
P46935	E3 ubiquitin-protein ligase NEDD4 OS=Mus musculus OX=10090 GN=Nedd4 PE=1 SV=3 - [NEDD4_MOUSE]	Nedd4	0	20.01941828	13.59832034	9.241840443	16.15388494	19.93743632	11.20591288	15.1110539	10.22188765	5.423519634	1	1	1.348489326	0.431344102
O08788	Dynactin subunit 1 OS=Mus musculus OX=10090 GN=Dctn1 PE=1 SV=3 - [DCTN1_MOUSE]	Dctn1	51.87149902	0	0	36.38156407	34.1020051	0	17.29049967	23.49452306	29.94802392	20.3787526	1	1	1.35881111	0.442344919
Q9ER72	Cysteine--tRNA ligase, cytoplasmic OS=Mus musculus OX=10090 GN=Cars1 PE=1 SV=2 - [SYCC_MOUSE]	Cars1	98.55276852	0	24.01978789	63.9436627	81.90632452	21.05680828	40.8575188	55.6355985	51.38865568	31.26393857	1	1	1.361697923	0.445406694
Q9R1V6	Disintegrin and metalloproteinase domain-containing protein 22 OS=Mus musculus OX=10090 GN=Adam22 PE=1 SV=2 - [ADA22_MOUSE]	Adam22	0	0	24.9598091	0	34.04123887	0	8.319936368	11.34707962	14.4105525	19.65371842	1	1	1.363842116	0.447676642
Q9CYG7	Mitochondrial import receptor subunit TOM34 OS=Mus musculus OX=10090 GN=Tomm34 PE=1 SV=1 - [TOM34_MOUSE]	Tomm34	9.993712432	0	0	13.6780277	0	0	3.331237477	4.559342566	5.769872563	7.897012974	1	1	1.368663326	0.452767606
Q505F5	Leucine-rich repeat-containing protein 47 OS=Mus musculus OX=10090 GN=Lrrc47 PE=1 SV=1 - [LRC47_MOUSE]	Lrrc47	23.04861789	0	0	17.0678296	14.59761883	0	7.682872631	10.55514948	13.30712574	9.224091862	1	1	1.373854544	0.458229268
Q99P31	Hsp70-binding protein 1 OS=Mus musculus OX=10090 GN=Hspbp1 PE=1 SV=1 - [HPBP1_MOUSE]	Hspbp1	29.87677968	0	0	0	41.27402949	0	9.958926558	13.75800983	17.24936679	23.82957204	1	1	1.381475177	0.466209639
Q80TV8	CLIP-associating protein 1 OS=Mus musculus OX=10090 GN=Clasp1 PE=1 SV=2 - [CLAP1_MOUSE]	Clasp1	79.57287414	0	0	41.25193202	68.70334799	0	26.52429138	36.65176	45.9414203	34.5819128	1	1	1.381818631	0.466568269
Q8K4F5	Protein ABHD11 OS=Mus musculus OX=10090 GN=Abhd11 PE=1 SV=1 - [ABHDB_MOUSE]	Abhd11	19.91349133	0	5.990007535	19.70170561	16.16625936	0	8.63449962	11.95598832	10.21674058	10.50400341	1	1	1.384676455	0.469548914
Q9ES28	Rho guanine nucleotide exchange factor 7 OS=Mus musculus OX=10090 GN=Arhgef7 PE=1 SV=2 - [ARHG7_MOUSE]	Arhgef7	23.82269379	0	0	9.705329296	23.54699349	0	7.94089793	11.08410759	13.75403867	11.83389191	1	1	1.395825471	0.481118564
Q9D883	Splicing factor U2AF 35 kDa subunit OS=Mus musculus OX=10090 GN=U2af1 PE=1 SV=4 - [U2AF1_MOUSE]	U2af1	14.87554689	0	0	0	20.76395661	0	4.958515629	6.92131887	8.588400999	11.98807594	1	1	1.395844924	0.481138669
Q6NSR8	Probable aminopeptidase NPEPL1 OS=Mus musculus OX=10090 GN=Npepl1 PE=1 SV=1 - [PEPL1_MOUSE]	Npepl1	69.82082688	0	0	67.9426203	29.66581142	0	23.27360896	32.53614391	40.3110732	34.06213469	1	1	1.397984471	0.483348335
P97315	Cysteine and glycine-rich protein 1 OS=Mus musculus OX=10090 GN=Csrp1 PE=1 SV=3 - [CSRP1_MOUSE]	Csrp1	58.49662371	0	0	50.26923435	31.63506802	0	19.49887457	27.30143412	33.77304144	25.41326952	1	1	1.400154354	0.485585879
O89079	Coatomer subunit epsilon OS=Mus musculus OX=10090 GN=Cope PE=1 SV=3 - [COPE_MOUSE]	Cope	57.72206182	47.10027739	0	36.8885089	68.81181219	41.61304492	34.94077974	49.10445534	30.7221279	17.22977788	1	1	1.405362322	0.490942125
P11214	Tissue-type plasminogen activator OS=Mus musculus OX=10090 GN=Plat PE=1 SV=3 - [TPA_MOUSE]	Plat	0	39.1881524	0	22.91535775	0	32.33964888	13.06271747	18.41833554	22.62529033	16.6322166	1	1	1.409992645	0.495687637
O08599	Syntaxin-binding protein 1 OS=Mus musculus OX=10090 GN=Stxbp1 PE=1 SV=2 - [STXB1_MOUSE]	Stxbp1	31.61981672	0	0	0	44.80892295	0	10.53993891	14.93630765	18.25570969	25.87044373	1	1	1.417115202	0.502957044
Q9D2L1	Arylsulfatase K OS=Mus musculus OX=10090 GN=Arsk PE=2 SV=2 - [ARSK_MOUSE]	Arsk	9.118435786	0	0	0	13.09608038	0	3.039478595	4.365360125	5.264531356	7.56102553	1	1	1.436220058	0.522276816
Q9ET22	Dipeptidyl peptidase 2 OS=Mus musculus OX=10090 GN=Dpp7 PE=1 SV=2 - [DPP2_MOUSE]	Dpp7	18.28964974	13.88916214	0	18.01516964	16.51166368	11.72983666	10.72627062	15.41889	9.546241873	3.282067704	1	1	1.437488437	0.523550351
Q8CH18	Cell division cycle and apoptosis regulator protein 1 OS=Mus musculus OX=10090 GN=Ccar1 PE=1 SV=1 - [CCAR1_MOUSE]	Ccar1	66.76641089	0	0	53.25374261	42.96798833	0	22.2554703	32.07391031	38.5476053	28.24891108	1	1	1.441169739	0.527240264
Q99J36	THUMP domain-containing protein 1 OS=Mus musculus OX=10090 GN=Thumpd1 PE=1 SV=1 - [THUM1_MOUSE]	Thumpd1	38.73690934	0	0	36.88086422	0	19.15800811	12.91230311	18.67962411	22.36476504	18.44508538	1	1	1.44665316	0.532719073
Q8BFR5	Elongation factor Tu, mitochondrial OS=Mus musculus OX=10090 GN=Tufm PE=1 SV=1 - [EFTU_MOUSE]	Tufm	32.24314449	0	0	21.67647359	25.20485008	0	10.74771483	15.62710789	18.61558815	13.64797553	1	1	1.453993536	0.540020855
Q60823	RAC-beta serine/threonine-protein kinase OS=Mus musculus OX=10090 GN=Akt2 PE=1 SV=1 - [AKT2_MOUSE]	Akt2	14.83614994	0	0	0	11.91976111	9.693037847	4.945383312	7.204266319	8.565655159	6.337638679	1	1	1.456766011	0.542769167
Q9D7G0	Ribose-phosphate pyrophosphokinase 1 OS=Mus musculus OX=10090 GN=Prps1 PE=1 SV=4 - [PRPS1_MOUSE]	Prps1	128.7026742	0	0	114.4052222	75.56241697	0	42.90089139	63.3225464	74.30652357	58.17645178	1	1	1.476019363	0.561711648
P19253	60S ribosomal protein L13a OS=Mus musculus OX=10090 GN=Rpl13a PE=1 SV=4 - [RL13A_MOUSE]	Rpl13a	59.30682831	0	0	31.52339663	56.41020573	0	19.76894277	29.31120079	34.24081329	28.27009348	1	1	1.482689344	0.568216353
Q61595	Kinectin OS=Mus musculus OX=10090 GN=Ktn1 PE=1 SV=1 - [KTN1_MOUSE]	Ktn1	42.93150395	0	0	33.6140172	30.34883872	0	14.31050132	21.32095197	24.78651536	18.5365205	1	1	1.489881556	0.575197643
Q6PGB6	N-alpha-acetyltransferase 50 OS=Mus musculus OX=10090 GN=Naa50 PE=1 SV=1 - [NAA50_MOUSE]	Naa50	62.10540082	0	0	45.78618092	47.40765138	0	20.70180027	31.06461077	35.85656988	26.91495538	1	1	1.500575329	0.585515745
Q8CIB5	Fermitin family homolog 2 OS=Mus musculus OX=10090 GN=Fermt2 PE=1 SV=1 - [FERM2_MOUSE]	Fermt2	28.76926364	0	0	22.88679159	20.49407274	0	9.589754546	14.46028811	16.60994211	12.57999305	1	1	1.507889283	0.592530503
P07607	Thymidylate synthase OS=Mus musculus OX=10090 GN=Tyms PE=1 SV=1 - [TYSY_MOUSE]	Tyms	27.22520091	0	0	19.73162827	21.34067754	0	9.075066969	13.6907686	15.71847707	11.88381751	1	1	1.508613507	0.593223248
P70206	Plexin-A1 OS=Mus musculus OX=10090 GN=Plxna1 PE=1 SV=1 - [PLXA1_MOUSE]	Plxna1	0	0	9.576894313	0	6.372493225	8.232166862	3.192298104	4.868220029	5.52922251	4.317322225	1	1	1.524989168	0.608798995
P52479	Ubiquitin carboxyl-terminal hydrolase 10 OS=Mus musculus OX=10090 GN=Usp10 PE=1 SV=3 - [UBP10_MOUSE]	Usp10	24.68190649	0	0	0	37.77477651	0	8.227302164	12.59159217	14.25010536	21.80927739	1	1	1.530464291	0.613969384
Q9EPU0	Regulator of nonsense transcripts 1 OS=Mus musculus OX=10090 GN=Upf1 PE=1 SV=2 - [RENT1_MOUSE]	Upf1	30.97666375	0	0	24.88119787	22.67961593	0	10.32555458	15.8536046	17.88438515	13.77368233	1	1	1.535375604	0.61859163
Q9QZB9	Dynactin subunit 5 OS=Mus musculus OX=10090 GN=Dctn5 PE=1 SV=1 - [DCTN5_MOUSE]	Dctn5	0	0	13.78207812	21.16686181	0	0	4.594026041	7.055620605	7.957086515	12.22069337	1	1	1.535825122	0.619013952
Q68FH0	Plakophilin-4 OS=Mus musculus OX=10090 GN=Pkp4 PE=1 SV=1 - [PKP4_MOUSE]	Pkp4	8.975206026	0	0	0	6.631685842	7.201572597	2.991735342	4.611086146	5.181837615	4.003470908	1	1	1.541274752	0.624124063
P11440	Cyclin-dependent kinase 1 OS=Mus musculus OX=10090 GN=Cdk1 PE=1 SV=3 - [CDK1_MOUSE]	Cdk1	103.7533251	0	0	80.3820795	80.71119598	0	34.5844417	53.69775849	59.90201017	46.50391413	1	1	1.552656508	0.6347387
C0HKG5	Ribonuclease T2-A OS=Mus musculus OX=10090 GN=Rnaset2a PE=1 SV=1 - [RNT2A_MOUSE]	Rnaset2a	26.15394864	52.26505409	51.90671693	38.58092243	50.82823511	113.2417496	43.44190656	67.55030236	14.97288276	40.04098434	1	1	1.554957131	0.636874806
P16381	Putative ATP-dependent RNA helicase Pl10 OS=Mus musculus OX=10090 GN=D1Pas1 PE=1 SV=1 - [DDX3L_MOUSE]	D1Pas1	51.32436836	0	0	0	79.9087691	0	17.10812279	26.63625637	29.63213789	46.13534935	1	1	1.55693624	0.638709864
Q8CGZ0	Calcium homeostasis endoplasmic reticulum protein OS=Mus musculus OX=10090 GN=Cherp PE=1 SV=1 - [CHERP_MOUSE]	Cherp	46.5401708	0	0	41.47315428	31.13993179	0	15.51339027	24.20436202	26.86998014	21.58893762	1	1	1.560223884	0.641753063
Q8K1M6	Dynamin-1-like protein OS=Mus musculus OX=10090 GN=Dnm1l PE=1 SV=2 - [DNM1L_MOUSE]	Dnm1l	30.15103097	0	0	21.41918314	25.64811747	0	10.05034366	15.6891002	17.40770585	13.75070436	1	1	1.561051118	0.642517781
Q8CFG0	Extracellular sulfatase Sulf-2 OS=Mus musculus OX=10090 GN=Sulf2 PE=2 SV=2 - [SULF2_MOUSE]	Sulf2	28.42417789	0	0	0	25.8047327	18.5687193	9.474725965	14.79115067	16.41070676	13.31065648	1	1	1.561116461	0.642578168
Q9CWM4	Prefoldin subunit 1 OS=Mus musculus OX=10090 GN=Pfdn1 PE=1 SV=1 - [PFD1_MOUSE]	Pfdn1	49.66546331	0	0	41.72531351	35.91247127	0	16.55515444	25.87926159	28.67436861	22.59976591	1	1	1.563214749	0.644515985
Q61142	Spindlin-1 OS=Mus musculus OX=10090 GN=Spin1 PE=1 SV=2 - [SPIN1_MOUSE]	Spin1	9.29684431	0	0	8.181790081	6.394166559	0	3.098948103	4.858652213	5.367535565	4.3016015	1	1	1.567839167	0.648777572
O55229	Choline/ethanolamine kinase OS=Mus musculus OX=10090 GN=Chkb PE=1 SV=3 - [CHKB_MOUSE]	Chkb	42.82628927	0	0	37.34924143	29.90340537	0	14.27542976	22.41754893	24.72576964	19.76790312	1	1	1.570358953	0.651094369
Q8BGY3	Leucine zipper protein 2 OS=Mus musculus OX=10090 GN=Luzp2 PE=2 SV=1 - [LUZP2_MOUSE]	Luzp2	49.88394487	0	0	38.80534528	39.53154759	0	16.62798162	26.11229762	28.800509	22.61682798	1	1	1.570382877	0.651116347
Q6PDJ1	VWFA and cache domain-containing protein 1 OS=Mus musculus OX=10090 GN=Cachd1 PE=2 SV=1 - [CAHD1_MOUSE]	Cachd1	0	15.5979748	0	0	0	24.59489525	5.199324934	8.198298416	9.005494951	14.19986939	1	1	1.57680055	0.657000185
Q9CXY6	Interleukin enhancer-binding factor 2 OS=Mus musculus OX=10090 GN=Ilf2 PE=1 SV=1 - [ILF2_MOUSE]	Ilf2	165.369013	0	0	101.8358435	159.0824241	0	55.12300432	86.97275588	95.47584415	80.57597623	1	1	1.577794189	0.657909029
Q3UH68	LIM and calponin homology domains-containing protein 1 OS=Mus musculus OX=10090 GN=Limch1 PE=1 SV=2 - [LIMC1_MOUSE]	Limch1	16.59426083	0	0	10.6737763	15.59661746	0	5.531420278	8.756797921	9.58070096	7.973062376	1	1	1.583101171	0.662753457
Q9DCH4	Eukaryotic translation initiation factor 3 subunit F OS=Mus musculus OX=10090 GN=Eif3f PE=1 SV=2 - [EIF3F_MOUSE]	Eif3f	93.55734913	0	0	67.57257857	80.72615738	0	31.18578304	49.43291199	54.01536071	43.31239718	1	1	1.585110495	0.664583411
Q8VDK1	Deaminated glutathione amidase OS=Mus musculus OX=10090 GN=Nit1 PE=1 SV=2 - [NIT1_MOUSE]	Nit1	25.76823674	0	0	20.54690461	20.42796967	0	8.589412246	13.65829143	14.87729842	11.82857684	1	1	1.590131087	0.669145703
D3YXK2	Scaffold attachment factor B1 OS=Mus musculus OX=10090 GN=Safb PE=1 SV=2 - [SAFB1_MOUSE]	Safb	99.42476855	0	0	87.20793683	71.53734883	0	33.14158952	52.91509522	57.40291689	46.49083037	1	1	1.596637216	0.675036544
Q6PDG5	SWI/SNF complex subunit SMARCC2 OS=Mus musculus OX=10090 GN=Smarcc2 PE=1 SV=2 - [SMRC2_MOUSE]	Smarcc2	38.53178529	0	0	24.87825599	36.77479596	0	12.84392843	20.55101732	22.24633661	18.76539769	1	1	1.600056978	0.678123281
Q8R317	Ubiquilin-1 OS=Mus musculus OX=10090 GN=Ubqln1 PE=1 SV=1 - [UBQL1_MOUSE]	Ubqln1	105.8916144	0	0	93.21343371	76.24556126	0	35.2972048	56.48633166	61.13655208	49.64883102	1	1	1.600306086	0.678347872
P61021	Ras-related protein Rab-5B OS=Mus musculus OX=10090 GN=Rab5b PE=1 SV=1 - [RAB5B_MOUSE]	Rab5b	37.79620942	0	0	0	60.52208873	0	12.59873647	20.17402958	21.82165169	34.94244422	1	1	1.601274034	0.679220224
Q9DBG5	Perilipin-3 OS=Mus musculus OX=10090 GN=Plin3 PE=1 SV=1 - [PLIN3_MOUSE]	Plin3	50.69270285	0	23.486352	48.22309328	49.65605825	20.91676801	24.72635162	39.59863985	25.36909005	16.19483247	1	1	1.601475238	0.679401492
Q8CGK3	Lon protease homolog, mitochondrial OS=Mus musculus OX=10090 GN=Lonp1 PE=1 SV=2 - [LONM_MOUSE]	Lonp1	13.6894817	0	0	10.81075653	11.14740637	0	4.563160567	7.319387632	7.903625945	6.341010153	1	1	1.604017112	0.681689532
Q8BVQ5	Protein phosphatase methylesterase 1 OS=Mus musculus OX=10090 GN=Ppme1 PE=1 SV=5 - [PPME1_MOUSE]	Ppme1	53.71671185	0	0	42.11553737	44.45459421	0	17.90557062	28.85671052	31.01335805	25.01799559	1	1	1.611605189	0.688498356
P34152	Focal adhesion kinase 1 OS=Mus musculus OX=10090 GN=Ptk2 PE=1 SV=4 - [FAK1_MOUSE]	Ptk2	34.74165515	0	0	25.13291636	31.24305074	0	11.58055172	18.79198903	20.05810395	16.55861028	1	1	1.622719668	0.698413789
Q8K3G9	DCC-interacting protein 13-beta OS=Mus musculus OX=10090 GN=Appl2 PE=1 SV=1 - [DP13B_MOUSE]	Appl2	0	0	35.94226436	0	58.6670576	0	11.98075479	19.55568587	20.751276	33.8714415	1	1	1.632258252	0.706869335
P47968	Ribose-5-phosphate isomerase OS=Mus musculus OX=10090 GN=Rpia PE=1 SV=2 - [RPIA_MOUSE]	Rpia	18.28975785	0	0	30.13233908	0	0	6.096585949	10.04411303	10.55959662	17.39691408	1	1	1.647497979	0.720276695
O88712	C-terminal-binding protein 1 OS=Mus musculus OX=10090 GN=Ctbp1 PE=1 SV=2 - [CTBP1_MOUSE]	Ctbp1	107.4014901	0	0	95.25819206	81.88613007	0	35.8004967	59.04810738	62.00827922	51.57239813	1	1	1.64936559	0.721911215
O35127	Protein C10 OS=Mus musculus OX=10090 GN=Grcc10 PE=1 SV=1 - [C10_MOUSE]	Grcc10	25.84684178	0	0	20.19611797	23.49488487	0	8.615613926	14.56366761	14.92268106	12.71989688	1	1	1.690380713	0.757348212
Q9EQG7	Ectonucleotide pyrophosphatase/phosphodiesterase family member 5 OS=Mus musculus OX=10090 GN=Enpp5 PE=1 SV=3 - [ENPP5_MOUSE]	Enpp5	27.07049393	0	0	24.0786261	21.93842162	0	9.023497978	15.33901591	15.62915696	13.32700925	1	1	1.699896863	0.765447217
Q9D1G1	Ras-related protein Rab-1B OS=Mus musculus OX=10090 GN=Rab1b PE=1 SV=1 - [RAB1B_MOUSE]	Rab1b	484.340885	0	0	384.3387527	441.9298585	0	161.4469617	275.4228704	279.6343403	240.255077	1	1	1.705965027	0.770588072
P97355	Spermine synthase OS=Mus musculus OX=10090 GN=Sms PE=1 SV=1 - [SPSY_MOUSE]	Sms	29.18601955	0	0	27.14732314	23.20912401	0	9.728673182	16.78548238	16.85055624	14.66941264	1	1	1.725361935	0.786899032
A2BH40	AT-rich interactive domain-containing protein 1A OS=Mus musculus OX=10090 GN=Arid1a PE=1 SV=1 - [ARI1A_MOUSE]	Arid1a	21.39577428	0	0	18.87858107	18.44338858	0	7.13192476	12.44065655	12.35285604	10.77612173	1	1	1.744361721	0.802699237
Q6ZWU9	40S ribosomal protein S27 OS=Mus musculus OX=10090 GN=Rps27 PE=1 SV=3 - [RS27_MOUSE]	Rps27	140.7226473	0	0	128.2868452	118.6632668	0	46.90754909	82.31670398	81.24625829	71.45056424	1	1	1.754871136	0.811365094
Q99K95	Replication termination factor 2 OS=Mus musculus OX=10090 GN=Rtf2 PE=1 SV=1 - [RTF2_MOUSE]	Rtf2	10.18726198	0	0	9.595795831	8.314409851	0	3.395753993	5.970068561	5.881618446	5.209777008	1	1	1.758098076	0.814015554
P62322	U6 snRNA-associated Sm-like protein LSm5 OS=Mus musculus OX=10090 GN=Lsm5 PE=3 SV=2 - [LSM5_MOUSE]	Lsm5	5.827286702	0	0	0	10.25355019	0	1.942428901	3.417850065	3.364385546	5.919889965	1	1	1.759575377	0.815227318
Q3UHN9	Bifunctional heparan sulfate N-deacetylase/N-sulfotransferase 1 OS=Mus musculus OX=10090 GN=Ndst1 PE=1 SV=2 - [NDST1_MOUSE]	Ndst1	0	0	14.207769	13.66674376	0	11.37170364	4.735923001	8.346149135	8.202859258	7.318500962	1	1	1.762306763	0.817465075
Q9CRA8	Exosome complex component RRP46 OS=Mus musculus OX=10090 GN=Exosc5 PE=1 SV=1 - [EXOS5_MOUSE]	Exosc5	23.02476661	0	0	40.64784906	0	0	7.674922202	13.54928302	13.2933552	23.4680466	1	1	1.76539679	0.81999248
P70274	Selenoprotein P OS=Mus musculus OX=10090 GN=Selenop PE=1 SV=3 - [SEPP1_MOUSE]	Selenop	18.34022501	0	0	32.75149816	0	0	6.113408337	10.91716605	10.58873385	18.90908628	1	1	1.785774065	0.836549563

O35344	Importin subunit alpha-4 OS=Mus musculus OX=10090 GN=Kpna3 PE=1 SV=1 - [IMA4_MOUSE]	Kpna3	29.29329245	0	0	0	52.62893532	0	9.764430817	17.54297844	16.91249028	30.38532997	1	1	1.796620691	0.845285854
Q8CI95	Oxysterol-binding protein-related protein 11 OS=Mus musculus OX=10090 GN=Osbpl11 PE=1 SV=2 - [OSB11_MOUSE]	Osbpl11	4.379693982	0	0	4.018005577	3.851923003	0	1.459897994	2.623309527	2.528617499	2.273369858	1	1	1.796912892	0.845520474
Q71LX4	Talin-2 OS=Mus musculus OX=10090 GN=Tln2 PE=1 SV=3 - [TLN2_MOUSE]	Tln2	100.58569	0	0	87.46324155	93.55860742	0	33.52856332	60.34061632	58.07317518	52.34530404	1	1	1.799677957	0.847738767
Q8VE70	Programmed cell death protein 10 OS=Mus musculus OX=10090 GN=Pdcd10 PE=1 SV=1 - [PDC10_MOUSE]	Pdcd10	65.36118084	0	0	55.29850775	62.35606387	0	21.78706028	39.21819054	37.73629535	34.1467733	1	1	1.800068024	0.848051426
P42669	Transcriptional activator protein Pur-alpha OS=Mus musculus OX=10090 GN=Pura PE=1 SV=1 - [PURA_MOUSE]	Pura	43.2202438	0	0	36.16420467	41.70112239	0	14.40674793	25.95510902	24.95321939	22.64762962	1	1	1.801593888	0.849273838
Q99NB8	Ubiquilin-4 OS=Mus musculus OX=10090 GN=Ubqln4 PE=1 SV=1 - [UBQL4_MOUSE]	Ubqln4	33.91999587	0	0	31.31928178	29.8379505	0	11.30666529	20.38574409	19.58371875	17.67010207	1	1	1.802984661	0.850387123
Q63844	Mitogen-activated protein kinase 3 OS=Mus musculus OX=10090 GN=Mapk3 PE=1 SV=5 - [MK03_MOUSE]	Mapk3	345.2951928	0	0	333.239108	293.7896802	0	115.0983976	209.0095961	199.3562725	182.0791662	1	1	1.815920989	0.860701432
Q3TVI8	Pre-B-cell leukemia transcription factor-interacting protein 1 OS=Mus musculus OX=10090 GN=Pbxip1 PE=1 SV=2 - [PBIP1_MOUSE]	Pbxip1	41.46210371	0	0	38.76597224	37.30626533	0	13.82070124	25.35741252	23.93815674	21.97228854	1	1	1.834741385	0.875576723
Q6WVG3	BTB/POZ domain-containing protein KCTD12 OS=Mus musculus OX=10090 GN=Kctd12 PE=1 SV=1 - [KCD12_MOUSE]	Kctd12	31.05349693	0	0	27.36967996	29.67059994	0	10.35116564	19.01342663	17.92874481	16.50625192	1	1	1.836839182	0.877225322
P63276	40S ribosomal protein S17 OS=Mus musculus OX=10090 GN=Rps17 PE=1 SV=2 - [RS17_MOUSE]	Rps17	100.3959156	0	0	87.75960907	97.8700726	0	33.46530521	61.87656056	57.96360891	53.82459408	1	1	1.84897643	0.886726834
P70362	Ubiquitin recognition factor in ER-associated degradation protein 1 OS=Mus musculus OX=10090 GN=Ufd1 PE=1 SV=2 - [UFD1_MOUSE]	Ufd1	227.7266091	0	0	212.4530929	210.9137942	0	75.9088697	141.1222957	131.4780191	122.2179165	1	1	1.859101529	0.89460556
Q8BVY0	Ribosomal L1 domain-containing protein 1 OS=Mus musculus OX=10090 GN=Rsl1d1 PE=1 SV=1 - [RL1D1_MOUSE]	Rsl1d1	21.88911929	0	0	19.18763821	21.68768226	0	7.296373095	13.62510682	12.63768891	11.86571562	1	1	1.867380772	0.901016133
O35593	26S proteasome non-ATPase regulatory subunit 14 OS=Mus musculus OX=10090 GN=Psmd14 PE=1 SV=2 - [PSDE_MOUSE]	Psmd14	66.56642738	0	0	66.11504296	60.31020579	0	22.18880913	42.14174958	38.43214477	36.61105471	1	1	1.8992344	0.925417971
Q9R0Y5	Adenylate kinase isoenzyme 1 OS=Mus musculus OX=10090 GN=Ak1 PE=1 SV=1 - [KAD1_MOUSE]	Ak1	28.44527655	0	0	27.50986521	26.87374678	0	9.48175885	18.12787066	16.42288807	15.70241806	1	1	1.91186793	0.934982867
Q9ERF3	WD repeat-containing protein 61 OS=Mus musculus OX=10090 GN=Wdr61 PE=1 SV=1 - [WDR61_MOUSE]	Wdr61	4.989869469	0	0	9.545053006	0	0	1.663289823	3.181684335	2.880902481	5.510838923	1	1	1.912886312	0.935751133
P53811	Phosphatidylinositol transfer protein beta isoform OS=Mus musculus OX=10090 GN=Pitpnb PE=1 SV=2 - [PIPNB_MOUSE]	Pitpnb	136.4535265	0	0	128.5025698	133.2803562	0	45.48450884	87.26097532	78.78148027	75.60797029	1	1	1.918476808	0.939961324
Q9D1P4	Cysteine and histidine-rich domain-containing protein 1 OS=Mus musculus OX=10090 GN=Chordc1 PE=1 SV=1 - [CHRD1_MOUSE]	Chordc1	59.70569898	0	0	58.43509623	56.27922632	0	19.90189966	38.23810752	34.47110138	33.13271184	1	1	1.92132953	0.942104979
Q9CWL8	Beta-catenin-like protein 1 OS=Mus musculus OX=10090 GN=Ctnnbl1 PE=1 SV=1 - [CTBL1_MOUSE]	Ctnnbl1	3.979044489	0	0	0	7.727524489	0	1.326348163	2.575841496	2.297302407	4.461488344	1	1	1.942055313	0.957584291
Q9JM14	5'(3')-deoxyribonucleotidase, cytosolic type OS=Mus musculus OX=10090 GN=Nt5c PE=1 SV=1 - [NT5C_MOUSE]	Nt5c	71.31846855	0	0	70.8859382	67.8799352	0	23.77282285	46.25529113	41.17573701	40.08644386	1	1	1.945721441	0.960305182
Q61239	Protein farnesyltransferase/geranylgeranyltransferase type-1 subunit alpha OS=Mus musculus OX=10090 GN=Fnta PE=1 SV=1 - [FNTA_MOUSE]	Fnta	10.9904186	0	0	21.49701064	0	0	3.663472867	7.165670213	6.345321137	12.41130488	1	1	1.955977422	0.967889717
Q569Z5	Probable ATP-dependent RNA helicase DDX46 OS=Mus musculus OX=10090 GN=Ddx46 PE=1 SV=2 - [DDX46_MOUSE]	Ddx46	25.11595138	0	0	24.69700675	24.52842526	0	8.371983793	16.40847734	14.50070129	14.2104082	1	1	1.959927031	0.970799943
O08759	Ubiquitin-protein ligase E3A OS=Mus musculus OX=10090 GN=Ube3a PE=1 SV=2 - [UBE3A_MOUSE]	Ube3a	19.06696041	0	0	38.96492531	0	0	6.355653469	12.98830844	11.00831472	22.49641011	1	1	2.04358348	1.031101178
O35343	Importin subunit alpha-3 OS=Mus musculus OX=10090 GN=Kpna4 PE=1 SV=1 - [IMA3_MOUSE]	Kpna4	10.79662593	0	0	0	22.0856942	0	3.598875311	7.361898067	6.233434889	12.75118149	1	1	2.045610762	1.032531656
Q8VHN8	Tudor-interacting repair regulator protein OS=Mus musculus OX=10090 GN=Nudt16l1 PE=1 SV=2 - [TIRR_MOUSE]	Nudt16l1	9.145818019	0	0	0	19.18806448	0	3.048606006	6.396021495	5.280340495	11.07823419	1	1	2.098015119	1.069025075
Q99J08	SEC14-like protein 2 OS=Mus musculus OX=10090 GN=Sec14l2 PE=1 SV=1 - [S14L2_MOUSE]	Sec14l2	11.34398022	0	0	0	25.05876816	0	3.78132674	8.352922719	6.549450033	14.46768654	1	1	2.208992582	1.143388574
Q9Z100	Probable carboxypeptidase X1 OS=Mus musculus OX=10090 GN=Cpxm1 PE=2 SV=2 - [CPXM1_MOUSE]	Cpxm1	0	11.94831091	0	0	26.51007666	0	3.982770302	8.83669222	6.898360518	15.3055999	1	1	2.218730067	1.149734159
Q9CS42	Ribose-phosphate pyrophosphokinase 2 OS=Mus musculus OX=10090 GN=Prps2 PE=1 SV=4 - [PRPS2_MOUSE]	Prps2	0	25.98326336	0	0	0	58.52530151	8.661087788	19.50843384	15.0014441	33.78959858	1	1	2.252423058	1.171477825
Q5SWU9	Acetyl-CoA carboxylase 1 OS=Mus musculus OX=10090 GN=Acaca PE=1 SV=1 - [ACACA_MOUSE]	Acaca	4.675796451	0	0	0	11.14004596	0	1.558598817	3.713348654	2.69957234	6.431708536	1	1	2.382491642	1.252471153
Q3TJZ6	Protein FAM98A OS=Mus musculus OX=10090 GN=Fam98a PE=1 SV=1 - [FA98A_MOUSE]	Fam98a	0.944201609	0	0	2.362896455	0	0	0.31473387	0.787632152	0.545135053	1.364218904	1	1	2.502533817	1.323389564
P62849	40S ribosomal protein S24 OS=Mus musculus OX=10090 GN=Rps24 PE=1 SV=1 - [RS24_MOUSE]	Rps24	48.20497996	0	0	0	121.0033751	0	16.06832665	40.33445836	27.83115816	69.86133117	1	1	2.510184117	1.327793187
Q8CG76	Aflatoxin B1 aldehyde reductase member 2 OS=Mus musculus OX=10090 GN=Akr7a2 PE=1 SV=3 - [ARK72_MOUSE]	Akr7a2	14.7086607	0	0	45.32536624	0	0	4.902886899	15.10845541	8.492049212	26.1686124	1	1	3.081542717	1.62365279
O70258	Epsilon-sarcoglycan OS=Mus musculus OX=10090 GN=Sgce PE=1 SV=2 - [SGCE_MOUSE]	Sgce	0	0	7.442651811	0	0	25.62014638	2.480883937	8.540048794	4.297017026	14.79179841	1	1	3.442341122	1.78339007
Q80TS3	Adhesion G protein-coupled receptor L3 OS=Mus musculus OX=10090 GN=Adgrl3 PE=1 SV=3 - [AGRL3_MOUSE]	Adgrl3	23.06926368	0	0	0	0	87.80535507	7.689754559	29.26845169	13.31904559	50.69444539	1	1	3.806162013	1.928336973
Q8BRF7	Sec1 family domain-containing protein 1 OS=Mus musculus OX=10090 GN=Scfd1 PE=1 SV=1 - [SCFD1_MOUSE]	Scfd1	6.418955689	0	0	0	30.07012483	0	2.139651896	10.02337494	3.705985795	17.36099466	1	1	4.684582085	2.22792035
Q9D0S9	Histidine triad nucleotide-binding protein 2, mitochondrial OS=Mus musculus OX=10090 GN=Hint2 PE=1 SV=1 - [HINT2_MOUSE]	Hint2	3.807122557	0	0	75.6328544	0	0	1.269040852	25.21095147	2.198043233	43.66664885	1	1	19.86614648	4.312240149
